# Differential impact of preventive cognitive therapy while tapering antidepressants versus maintenance antidepressant treatment on affect fluctuations and individual affect networks and impact on relapse: a secondary analysis of a randomised controlled trial

**DOI:** 10.1016/j.eclinm.2023.102329

**Published:** 2023-11-22

**Authors:** Junus M. van der Wal, Claudia D. van Borkulo, Jonas M.B. Haslbeck, Christien Slofstra, Nicola S. Klein, Tessa F. Blanken, Marie K. Deserno, Anja Lok, Maaike H. Nauta, Claudi L. Bockting

**Affiliations:** a Department of Psychiatry, Amsterdam UMC, location AMC, Meibergdreef 5, 1105 AZ, Amsterdam, the Netherlands; b Centre for Urban Mental Health, University of Amsterdam, Amsterdam, the Netherlands; c Department of Public and Occupational Health, Amsterdam UMC, location VUmc, Van der Boechorststraat 7, 1081 BT, Amsterdam, the Netherlands; d Department of Psychological Methods, University of Amsterdam, Nieuwe Achtergracht 129-B, 1018 WS, Amsterdam, the Netherlands; e Maastricht University, Faculty of Psychology and Neuroscience, Universiteitssingel 40, 6229 ER, Maastricht, the Netherlands; f Lentis Psychiatric Institute, Centre for Integrative Psychiatry Research, Hereweg 80, 9725 AG, Groningen, the Netherlands; g Department Trauma Centre, GGZ Drenthe Mental Health Institute, Altingerweg 1, 9411 PA, Beilen, the Netherlands; h Department of Clinical Psychology and Experimental Psychopathology, University of Groningen, Bloemstraat 36, 9712 LE, Groningen, the Netherlands

**Keywords:** Recurrent depression, Relapse prevention, Affect fluctuations, Network theory, Personalisation, Ecological momentary assessment

## Abstract

**Background:**

There is an urgent need to better understand and prevent relapse in major depressive disorder (MDD). We explored the differential impact of various MDD relapse prevention strategies (pharmacological and/or psychological) on affect fluctuations and individual affect networks in a randomised setting, and their predictive value for relapse.

**Methods:**

We did a secondary analysis using experience sampling methodology (ESM) data from individuals with remitted recurrent depression that was collected alongside a randomised controlled trial that ran in the Netherlands, comparing: (I) tapering antidepressants while receiving preventive cognitive therapy (PCT), (II) combining antidepressants with PCT, or (III) continuing antidepressants without PCT, for the prevention of depressive relapse, as well as ESM data from 11 healthy controls. Participants had multiple past depressive episodes, but were remitted for at least 8 weeks and on antidepressants for at least six months. Exclusion criteria were: current (hypo)mania, current alcohol or drug abuse, anxiety disorder that required treatment, psychological treatment more than twice per month, a diagnosis of organic brain damage, or a history of bipolar disorder or psychosis. Fluctuations (within-person variance, root mean square of successive differences, autocorrelation) in negative and positive affect were calculated. Changes in individual affect networks during treatment were modelled using time-varying vector autoregression, both with and without applying regularisation. We explored whether affect fluctuations or changes in affect networks over time differed between treatment conditions or relapse outcomes, and predicted relapse during 2-year follow-up. This ESM study was registered at ISRCTN registry, ISRCTN15472145.

**Findings:**

Between Jan 1, 2014, and Jan 31, 2015, 72 study participants were recruited, 42 of whom were included in the analyses. We found no indication that affect fluctuations differed between treatment groups, nor that they predicted relapse. We observed large individual differences in affect network structure across participants (irrespective of treatment or relapse status) and in healthy controls. We found no indication of group-level differences in how much networks changed over time, nor that changes in networks over time predicted time to relapse (regularised models: hazard ratios [HR] 1063, 95% CI <0.0001–>10 000, p = 0.65; non-regularised models: HR 2.54, 95% CI 0.23–28.7, p = 0.45) or occurrence of relapse (regularised models: odds ratios [OR] 22.84, 95% CI <0.0001–>10 000, p = 0.90; non-regularised models: OR 7.57, 95% CI 0.07–3709.54, p = 0.44) during complete follow-up.

**Interpretation:**

Our findings should be interpreted with caution, given the exploratory nature of this study and wide confidence intervals. While group-level differences in affect dynamics cannot be ruled out due to low statistical power, visual inspection of individual affect networks also revealed no meaningful patterns in relation to MDD relapse. More studies are needed to assess whether affect dynamics as informed by ESM may predict relapse or guide personalisation of MDD relapse prevention in daily practice.

**Funding:**

The 10.13039/501100001826Netherlands Organisation for Health Research and Development, 10.13039/501100003246Dutch Research Council, 10.13039/501100001827University of Amsterdam.

**Research in context**

**Evidence before this study**

We searched PubMed and Google Scholar, without language restrictions, for randomised studies on affect dynamics (ie, affect fluctuations and/or affect networks) that examined the impact of different treatment conditions on these dynamics, as well as their predictive value for depressive relapse, in individuals with remitted recurrent depression. We used search terms such as “affect fluctuation”, “affect dynamics”, “affect instability”, “affect variability”, “affect inertia”, “major depressive disorder”, “depressive relapse” and “relapse prevention”. We additionally searched for studies that examined change over time in affect networks in a clinical sample, using search terms such as “network analysis”, “network theory”, “affect networks”, “time-varying vector autoregressive models”, and “temporal networks”. We found no studies that explored the differential impact of different treatments on affect fluctuations or individual affect networks in a randomised setting. Also, we did not find publications that examined change over time in individual affect networks in a clinical sample of individuals with remitted recurrent depression at high risk of relapse. While there are some studies that examine the role of affect dynamics in depressive relapse, most of these studies comprise small samples or single case-studies and mostly focus on depressive symptomatology rather than clinically diagnosed depressive relapse. Furthermore, evidence compiled through these studies is inconclusive, pointing at both higher levels of fluctuation (eg, affect variability) as well as lower levels of fluctuation (eg, affect inertia) to precede increase in depressive symptoms. Moreover, there is a dearth of knowledge on the impact of current treatment and/or relapse prevention strategies for major depressive disorder (MDD) on affect dynamics and in turn how these dynamics impact risk of relapse.

**Added value of this study**

This is the first randomised experience sampling methodology (ESM) study to explore the differential impact of various MDD relapse prevention strategies (ie, preventive cognitive therapy while tapering antidepressants versus maintenance antidepressant treatment versus their combination) on affect fluctuations and individual affect networks, and examine the subsequent impact on occurrence of—or time to–MDD relapse, in a sample (n = 42) individuals with remitted recurrent depression. Also, this is the first study exploring change over time in individual affect networks (using time-varying vector auto-regressive models) in a clinical sample of individuals with remitted recurrent depression at high risk of relapse, in line with the network theory of mental disorders. While results of this exploratory study should be interpreted with caution, our findings provide no indication of a differential impact of various relapse prevention strategies with distinctly different target points on temporal affect dynamics (ie, both affect fluctuations and changes over time in individual affect networks), nor for the predictive value of these dynamics for MDD relapse.

**Implications of all the available evidence**

Altogether, evidence to date casts some doubt on the clinical relevance of ESM-informed affect fluctuations and individual affect networks for predicting relapse or personalising relapse prevention in recurrent MDD. More studies are needed to assess whether affect dynamics, as informed by ESM, may predict relapse or guide personalisation of MDD relapse prevention in daily practice.

## Introduction

Understanding and effectively addressing the high relapse rates in major depressive disorder (MDD) remains one of the most urgent challenges in the treatment of MDD, as 40–60% of individuals who suffer from a depressive episode experience relapse.^1^ Current relapse prevention strategies include continuation of antidepressant medication (ADM), and psychological interventions such as Preventive Cognitive Therapy (PCT) or Mindfulness-Based Cognitive Therapy (MBCT).^2^ While ADM continuation has been shown to reduce the risk of relapse with approximately 20% when compared to discontinuation during follow-up up to 1 year,^3^ PCT and MBCT have been demonstrated to mitigate the increased risk of MDD relapse associated with ADM tapering.^2^^,^^4^ Furthermore, PCT has been found to further decrease the relative risk of relapse with around 40% when administered alongside ADM maintenance therapy, compared to ADM alone.^4^

The rationale behind psychological relapse prevention interventions lies in part in the notion that some psychological disturbances associated with MDD, such as dysfunctional cognitions and beliefs or affect dysregulation, can persist after recovery.^1^^,^^5^, ^6^, ^7^ For example, latent dysfunctional beliefs and schemata in remitted individuals may be reactivated by adverse life events or momentary negative mood states, which could lead to negative thoughts and subsequently evoke MDD relapse.^8^, ^9^, ^10^ Additionally, affect dysregulation, measured by fluctuations in negative and positive affect (ie, NA and PA), has also been suggested to play a role in MDD relapse. Studies in this field have mostly focussed on affect variability (ie, overall changes in affect), affect instability (ie, moment-to-moment fluctuations in affect), and affect inertia (ie, resistance to affective change).^11^ However, current evidence on the role and clinical relevance of affect fluctuations in MDD relapse is inconclusive. For example, while a joined increase in affect variability and inertia (ie, ‘critical slowing down’), has been shown to precede an increase in depressive symptoms in two individual case-studies^12^^,^^13^ as well as in a group-level analysis,^14^ a more recent study only found a rise in affect inertia to be associated with worsening of depressive symptoms.^15^ In contrast, one study (n = 42) looking at clinically diagnosed MDD relapse, found no relationship between increase in NA inertia and risk of relapse, and also no clear relationship between individual NA trajectories and risk of relapse during 15 month follow-up.^16^ Moreover, there is a dearth of knowledge on how current relapse prevention strategies influence affect fluctuations, and how this in turn impacts the risk of MDD relapse. Also, this has not yet been studied from a network perspective, even though conceptualising psychopathology as a dynamic network of interacting elements has gained considerable traction over the past decade.^17^ Therefore, we extend past research by examining both affect fluctuation (ie, instability, variability, and inertia), as well as changes in how different affect items may influence each other over time in temporal affect networks (eg, sad mood predicting hopelessness at a next time point), in individuals with remitted recurrent depression in the context of different randomised relapse prevention treatment conditions and in relation to MDD relapse. Doing so may shed light on the differential impact of current MDD relapse prevention strategies on individual affect dynamics, and in turn whether this has a meaningful impact on subsequent risk of relapse. For the remainder of this article we will refer to ‘affect fluctuations’ as an umbrella term for instability, variability, and inertia, and ‘temporal affect dynamics’ as an umbrella term that includes both affect fluctuations and changes over time in temporal affect networks.

This study utilises experience sampling methodology (ESM) data on positive and negative affect items (n = 42 participants) collected alongside the pragmatic three-arm randomised-controlled Disrupting the Rhythm of Depression (DRD) trial, which tested the efficacy of eight weekly sessions of PCT for preventing MDD relapse.^4^ The DRD-trial randomised n = 289 individuals with remitted recurrent depression using ADM to either: (I) receive PCT while tapering ADM, (II) receive PCT while continuing ADM, or (III) continue ADM without PCT (treatment-as-usual). In short, PCT focusses on identification of dysfunctional attitudes and schemas and evaluation using specific techniques such as identification of wishful beliefs, positive mental imagery and enhancing positive affect,^18^ enhancing recall and specificity of positive autobiographical memory, and formulation of a personalised prevention strategy.^19^ Thus, while the treatment-as-usual group witnessed no change in treatment, the other groups received additional psychological treatment with a distinctly different target point, either with or without tapering ADM. The DRD-trial found that, during 2-year follow-up, ADM alone was not superior to receiving PCT while tapering ADM in preventing MDD relapse, while adding PCT to ADM continuation was superior to ADM alone and decreased risk of relapse with 41%.^4^

The present study has three aims. First, we explore the differential impact of the different relapse prevention strategies on affect fluctuations, as well as the predictive value of these fluctuations for (time to) MDD relapse. Second, we will descriptively explore whether changes over time in individual temporal affect networks are differentially impacted by treatment conditions, and whether there may be specific affect network trajectories that lead up to MDD relapse. Finally, we explore whether the average change in affect network structure over time differs between treatment conditions, between participants who relapse versus those who remain in remission, and whether this is predictive of (time to) MDD relapse.

While this is an exploratory study, we formulate some tentative expectations based on past findings. First, since affect instability is common while tapering ADM,^20^ it is reasonable to expect higher levels of affect instability and possibly more change over time in temporal affect network structure in participants who taper ADM, even though this effect may be (partly) mitigated by PCT over the course of the therapy. Also, given that PCT in part aims to activate positive affect, it could result in increases in PA over the course of the study period and thus higher PA variability in groups receiving PCT.^4^ Given that the group randomised to continue ADM without PCT (treatment-as-usual) sees no change in treatment, we do not expect to find high levels of affective change or fluctuation, nor major changes in temporal affect network structure over the course of the study period. Lastly, we formulate no specific expectations with regard to how affect fluctuations relate to MDD relapse, given the inconclusive previous findings in the field.

## Methods

### Study design and participants

This study utilised ESM data collected among a subset of participants in the pragmatic three-arm randomised-controlled DRD trial, which tested the efficacy of eight weekly sessions of PCT in preventing MDD relapse. The rationale, methodology (including randomisation and masking of participants), and results of this trial have been described in more detail elsewhere.^4^^,^^21^^,^^22^ In short, the DRD trial randomised 289 participants who experienced multiple past depressive episodes, but were remitted for at least 8 weeks and on ADM for the past six months, between three relapse prevention strategies: (I) receiving PCT while tapering ADM, (II) combining PCT with ADM continuation, or (III) continuing their ADM without receiving PCT (treatment-as-usual). Exclusion criteria were: current (hypo)mania, current alcohol or drug abuse, anxiety disorder that required treatment, psychological treatment more than twice per month, a diagnosis of organic brain damage, or a history of bipolar disorder or psychosis. The primary endpoint of the DRD trial was time-related proportion of participants experiencing MDD relapse, as assessed by trained assessors blinded to treatment allocation at 3, 9, 15, and 24 months past baseline, using the Structured Clinical Interview for DSM-IV Axis-I Disorders (SCID) and retrospective parts of information from monthly ratings on the Inventory of Depressive Symptomatology Self-Report.^4^ In addition, during the last phase of trial inclusion, all participants that were included in the DRD trial after screening were additionally asked to participate in an ESM study. Inclusion for the DRD trial at large took place between July 14, 2009, and April 30, 2015, while recruitment for the added ESM study took place between January 1, 2014 and January 31 2015. While the ESM study protocol foresaw the inclusion of 15 participants per treatment arm, considerable early drop-out resulted in additional recruitment, amounting in a total 72 participants who were recruited for ESM data alongside the DRD trial. The number of participants was chosen to be able to examine individual-level patterns of i.a. affect fluctuation in participants at high risk of MDD relapse undergoing different relapse prevention strategies and explore possible differences between randomised treatment conditions, although the sample size was not powered to test for group-level differences. Participants in the ESM study followed the same treatment protocol as other participants, although PCT was always administered individually (rather than in group sessions). Participants randomised to taper their ADM were instructed to gradually do so over a 4-week period guided by their general practitioner or psychiatrist, in accordance with international guidelines at the time.^23^ In addition, 15 matched controls without history of depression (ie, matched on age, sex, and level of education) were recruited to complete the ESM procedure to enable post-hoc comparisons between individuals with remitted recurrent depression and individuals without history of depression.^22^

### Ethics

Ethical approval for the DRD trial and the ESM data collection was provided by University Medical Centre Groningen (METc 2009/158). The DRD trial at large was conducted in accordance with CONSORT guidelines, as can be read in the main outcome paper.^4^ Ethical approval for including individuals without a history of depression was obtained from the University of Groningen Ethical Committee of the Psychology Department (ppo-014-043). All participants provided written informed consent. This ESM study was registered at ISRCTN registry, ISRCTN15472145.^24^ In line with the exploratory nature, the registered protocol, which is available in the Supplementary Materials, articulates the overall aims of the ESM study, without detailing an a-priori analysis plan.

### ESM procedure

The ESM data collection started upon enrolment in the DRD trial and lasted eight weeks (ie, the duration of the PCT intervention). Participants received random notifications to immediately complete a questionnaire via a mobile phone app ten times per day, for three consecutive days per week (ie, Thursday, Friday, Saturday, chosen to reflect both weekdays and the weekend), yielding a maximum of 240 responses. Sampling took place three days per week, since initial piloting to test the feasibility of the ESM regimen indicated that more days would be too burdensome for participants, given the number of daily notifications and length of the ESM period. Participants were not informed of a 5-min time limit. Adherence to the ESM procedure and treatment protocol was monitored through weekly phone interviews. The questionnaire included items on positive and negative affect, momentary thoughts and visual imagery, and current activity.^22^ For this study, we only used data on positive and negative affect items (scored from 0 to 100), which included anxious, angry, lonely, irritated, feeling down, suspicious, helpless, guilty, and insecure as negative affect items and cheerful, enthusiastic, hopeful, content, and energetic as positive affect items. Affect items in the ESM questionnaire were based on the PANAS scale, as well as on previous research on daily affect fluctuation and the consideration to include affect items that are reflective of presence or absence of depressive symptomatology (eg, feeling down, guilty, irritated, enthusiasm, energetic).^22^^,^^25^^,^^26^ Aggregated NA and PA were calculated by computing the mean score of all aforementioned negative and positive affect items, respectively.

### Statistical analysis

Descriptive statistics provided include sex, age, 17-item Hamilton Depression Ratings Scale (HDRS) score at baseline, types of ADM used, proportion of participants who experienced relapse during short-term (<3 months) or complete follow-up (24 months), and treatment protocol adherence. As a general description of the affect scores throughout the study period, we calculated the means of all separate affect items and aggregated NA and PA per week throughout the study period, both for the entire sample as well as per treatment group.

For all affect items and aggregated NA and PA, we calculated the within-person variance (WPV, a measure of affect variability), root mean square of successive difference (RMSSD, a measure of affect instability), and autocorrelation (AC, a measure of affect inertia).^11^ Participants were excluded if they missed >70% of the notifications. When calculating the successive difference between consecutive time points (necessary to compute the RMSSD and AC) we excluded differences between measurements that were two or more time points apart, including the last measurement on Saturday and the first measurement on Thursday. Fluctuation measures were used in three ways. First, to explore the differential impact of a change in treatment relative to baseline, we compared mean WPV, RMSSD, and AC in aggregated NA and PA in the two groups receiving PCT (with or without ADM tapering), to the treatment-as-usual group that did not receive PCT and continued ADM, using Dunnett's multiple comparison test and a significance level of 0.05. Second, logistic regression analysis was used to explore whether WPV, RMSSD, or AC in aggregated NA or PA or in individual affect items (independent variables) were predictive of the occurrence of relapse during 24-month follow-up (dependent variable), and whether there was an interaction effect with treatment condition. If multiple measures were a significant predictor, they were simultaneously added to the models to explore whether different fluctuation measures together accounted for the same variance in relapse, given their suggested overlap in measuring fluctuation.^27^ To account for possible multicollinearity between fluctuation measures, predictors were not introduced in a combined logistic regression model if the variance inflation factor (VIF) exceeded the rule of thumb score of 10.^28^ Third, cox proportional hazard models were used to explore whether WPV, RMSSD, or AC in aggregated NA or PA or individual affect items (independent variables) were predictive of time to relapse (dependant variable, measured in days), and whether there was an interaction effect with treatment condition. For right-censored participants, the total days of participating in the study was taken as dependent variable. In the latter two analyses, fluctuation measures were standardised to allow a closer comparison of the effect size and significance level alpha was subject to Bonferroni correction (p = 0.003).

We explored, for each participant, how individual affect items influenced each other over time in a temporal network (ie, in line with the network theory of mental disorders),^17^ how these networks changed over the course of treatment, and how this related to MDD relapse. To this end, we estimated individual lag-1 time-varying vector-autoregressive (TV-VAR) models for all participants, using the R-package *mgm.*^29^ In these models, variables (ie, the affect item scores) are predicted by the values of all variables at a previous time point (eg sad mood predicting hopelessness at a next time point). The resulting parameters can be visualised in a temporal network, as positive arrows (ie, solid blue arrows) or negative arrows (ie, dashed red arrows) between the different network nodes (ie, affect items), thus visualising conditional dependence between different affect items over a time lag of 1. We utilised TV-VAR models since they are, in contract to standard VAR models, able to account for changes in networks over time, which is plausible in the case of a change in treatment (ie, addition of PCT with or without tapering ADM) or possible occurrence of MDD relapse. For statistical and computational reasons, we limited the number of affect items in our networks to seven and excluded participants who responded to <30% of the notifications.^30^ Affect items in the networks were chosen to reflect positive and negative affect dimensions, high and low arousal, and key symptoms of MDD and included: *feeling down*, *anxious*, *irritated*, *guilty*, *cheerful*, *energetic*, and *hopeful*.^31^ For each participant, eight temporal networks were estimated evenly spread throughout the ESM period.

The output of the TV-VAR models is reported in several ways. First, we visually inspected the resulting individual networks to explore if specific trajectories in temporal affect dynamics could be identified, either per treatment group or in participants who relapsed versus those who remained in remission. Second, we quantified the average change over time in network structure both at the individual-level and group-level (ie, per treatment group or relapse status). We used these values to explore if change over time in affect network structure differed between treatment groups or between participants who relapsed versus those who remained in remission, using an ANOVA or Kruskal–Wallis test (when a Shapiro–Wilks test indicated violation of normality). Logistic regression and cox proportional hazard models were estimated to explore whether individual change over time in affect network structure (independent variable) was predictive of MDD relapse or days to relapse (dependent variable). Total days of participating in the study was taken as dependent variable for right-censored participants. We considered a p-value of 0.05 to be statistically significant.

We conducted two sensitivity analyses to check the robustness of our findings. First, following the pragmatic design of the DRD trial, participants who did not fully adhere to the treatment protocol were still included for analyses. Since not all participants were able to complete ADM tapering during the ESM period, we conducted all analyses where exploratory comparisons between treatment groups were drawn both in accordance with treatment randomisation (ie, intention-to-treat), as well as with the actual treatment received (ie, as-treated), and reported both outcomes.^32^ Second, for every regression in the TV-VAR analyses, least absolute shrinkage and selection operator (LASSO) regularisation can be applied, which sets small estimates to zero to minimise the number of false positives.^33^ Since this is an exploratory study, we estimated models both with and without regularisation and report the results of both analyses.

For additional details on TV-VAR models or how average change in network structure was calculated, we refer to the methodological appendix. The full R script of all the analyses is available in a public repository on the Open Science Framework. Data are not publicly available due to privacy reasons, but are available for research purposes upon reasonable request with the corresponding author. All analyses were performed in R version 4.0.3.^34^

### Role of the funding source

The study funders had no role in study design; data collection, data analysis, or data interpretation; or writing of the report.

## Results

**Fig. 1:**
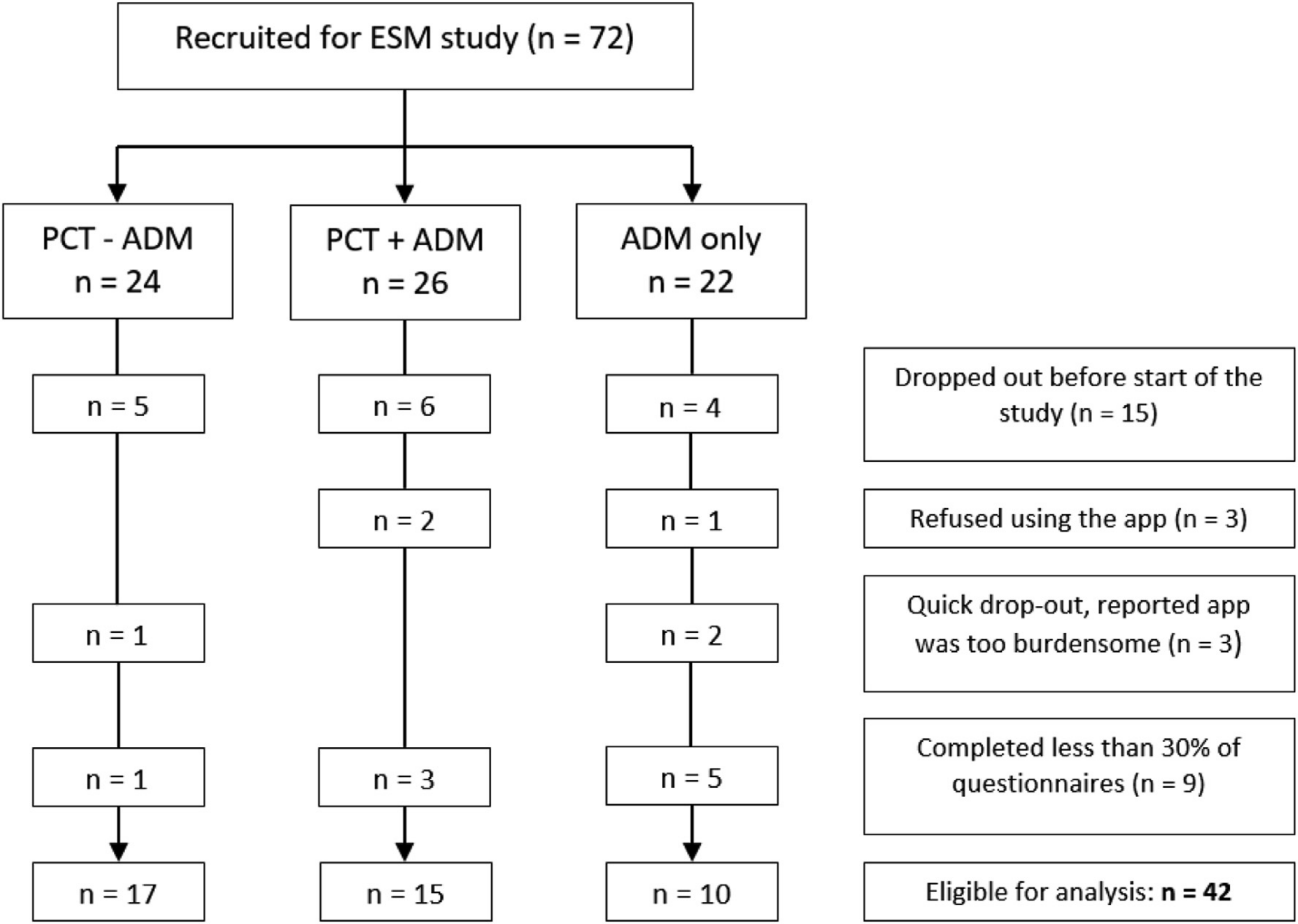
**Flowchart of treatment allocation and inclusion in final analyses**. ESM = experience sampling methodology, PCT = preventive cognitive therapy, ADM = antidepressant medication.

Between Jan 1, 2014 and Jan 31, 2015, 72 DRD trial participants were recruited to participate in the ESM study that ran alongside the trial. Of these 72 participants and the 15 never-depressed healthy controls, 42 and 11 participants respectively provided enough data to be included in the analyses. Fig. 1 provides an overview of the number of initially recruited participants, as well as the reasons for the differential number of drop-outs, per treatment arm. Baseline and follow-up characteristics of the final sample are shown in Table 1. The majority of the sample was female (69.05%), the mean age of the sample was 50.86 (standard deviation, SD: 11.21), and mean baseline HDRS was low in accordance with their remitted status (mean: 2.90, SD: 3.00). Notably, while all participants randomised to receive PCT completed the PCT protocol, almost half (n = 8 out of 17, 47%) of the participants randomised to taper ADM was not able to do so during the ESM period, while two out of 17 (12%) only partially tapered ADM and one participant (6%) increased ADM dosage during this period (Table 1). Of all the participants, 5 relapsed within the first 3 months, and 25 relapsed during the entire follow-up period. Mean affect scores throughout the ESM period are shown in Supplementary Table S7. Most group-level mean affect scores showed little change over the course of the study, except for a decrease in aggregated NA scores in the treatment-as-usual group that continued their ADM and did not receive PCT.

**Table 1:** Baseline and follow-up characteristics.

	Total group (n = 42)	PCT taper ADM (n = 17)	PCT continue ADM (n = 15)	Continue ADM only (n = 10)
Sex				
No. female, %	29, 69.05%	9, 52.94%	11, 73.33%	9, 90%
No. males, %	13, 30.95%	8, 47.06%	4, 26.67%	1, 10%
Age (mean, SD)	50.86, 11.21	42.53, 11.36	50.13, 11.00	49.10, 12.04
Baseline HDRS (mean, SD)	2.90, 3.00	3.24, 2.97	2.67, 3.00	2.70, 3.34
Past episodes (mean, SD)	5.48, 5.12	4.76, 2.44	6.87, 8.12	4.60, 1.07
Type of ADM				
SSRI	35	16	12	7
SNRI	2	–	1	1
TCA	3	–	1	2
Atypical	1	1	–	–
Combination	1	–	1	–
Protocol adherence				
Completed PCT^a^ (n, %)	–	17 (100%)	15 (100%)	–
ADM tapering (n, %)				
Complete tapering	–	6 (35%)	–	–
Partial tapering	–	2 (12%)	1 (7%)	–
No change	–	8 (47%)	13 (87%)	9 (90%)
Increase	–	1 (6%)	1 (7%)	1 (10%)
Relapse <3 months FU (n, %)	5 (11.90%)	3 (17.65%)	2 (13.33%)	–
Relapse <24 months FU (n, %)	25 (59.52%)	11 (64.71%)	9 (60.00%)	5 (50%)

PCT = preventive cognitive therapy, ADM = antidepressant medication, SD = standard deviation, HDRS = 17-item Hamilton Depression Rating Scale, FU = follow-up, SSRI = selective serotonin reuptake inhibitor, SNRI = serotonin–norepinephrine reuptake inhibitor, TCA = tricyclic antidepressant.

a completed >5 out of 8 sessions of PCT.

We found no differences in variability (WPV), instability (RMSSD), or inertia (AC), in aggregated NA or PA between treatment groups that witnessed a change in treatment (ie, addition of PCT with or without tapering ADM) versus the treatment-as-usual group (ie, ADM only), both in the intention-to-treat and as-treated analyses, although confidence intervals were wide (see Table 2 for group differences, 95% confidence intervals, and p-values). None of the fluctuation measures for any of the affect items nor aggregated NA or PA was predictive of occurrence of relapse or time to relapse (see Supplementary Tables S1 and S2 for corresponding odds ratios [OR] and hazard ratios [HR], including 95% confidence intervals), neither was there a significant interaction effect with treatment group allocation.

**Table 2:** Differences in mean NA and PA fluctuations between treatment groups.

*Fluc. measure*	PCT tapering ADM (n = 17) *(Mean, SD)*	ADM only (n = 10) *(Mean, SD)*	Difference (95% CI, p-value)	PCT plus ADM (n = 15) *(Mean, SD)*	ADM only (n = 10) *(Mean, SD)*	Difference (95% CI, p-value)
**Intention-to-treat**						
**WPV**						
PA	186.38, 137.30	134.69, 37.48	51.69 (−65.77 to 169.15, 0.49)	208.00, 155.62	134.69, 37.48	73.31 (−47.02 to 193.64, 0.28)
NA	118.28, 103.89	52.12, 37.12	66.16 (−1.38 to 133.69, 0.06)	51.99, 46.96	52.12, 37.12	−0.13 (−69.31 to 69.05, 1.00)
**AC**						
PA	0.4837, 0.1683	0.4817, 0.1214	0.0020 (−0.1425 to 0.1464, 1.00)	0.4947, 0.1696	0.4817, 0.1214	0.0130 (−0.1350 to 0.1610, 0.97)
NA	0.5163, 0.2318	0.5525, 0.2076	−0.0362 (−0.2451 to 0.1727, 0.88)	0.3655, 0.2419	0.5525, 0.2076	−0.1869 (−0.4009 to 0.0271, 0.09)
**RMSSD**						
PA	12.21, 4.65	10.84, 1.65	1.38 (−2.66 to 5.13, 0.60)	12.03, 4.61	10.84, 1.65	1.19 (−2.66 to 5.03, 0.69)
NA	7.93, 3.57	5.31, 2.05	2.62 (−0.29 to 5.53, 0.08)	6.57, 3.37	5.31, 2.05	1.26 (−1.72 to 4.24, 0.51)
***Fluc. measure***	**PCT tapering ADM (n = 9)*****(Mean, SD)***	**ADM only (n = 10)*****(Mean, SD)***	**Difference (p-value)**	**PCT plus ADM (n = 23)*****(Mean, SD)***	**ADM only (n = 10)*****(Mean, SD)***	**Difference (p-value)**
**As-treated**						
**WPV**						
PA	177.61, 151.35	134.69, 37.48	42.91 (−92.85 to 178.68, 0.68)	203.92, 144.08	134.69, 37.48	69.22 (−42.70 to 181.15, 0.27)
NA	78.49, 97.86	52.12, 37.12	26.37 (−57.66 to 110.40, 0.69)	90.62, 85.55	52.12, 37.12	38.50 (−30.78 to 107.77, 0.34)
**AC**						
PA	0.4463, 0.1343	0.4817, 0.1214	−0.0354 (−0.20 to 0.13, 0.83)	0.5055, 0.1771	0.4817, 0.1214	0.0238 (−0.11 to 0.16, 0.89)
NA	0.4190, 0.2141	0.5525, 0.2076	−0.1335 (−0.3849 to 0.1180, 0.37)	0.4560, 0.2598	0.5525, 0.2076	−0.0964 (−0.3037 to 0.1109, 0.46)
**RMSSD**						
PA	11.88, 4.39	10.84, 1.65	1.04 (−3.30 to 5.38, 0.80)	12.22, 4.71	10.84, 1.65	1.39 (−2.19 to 4.96, 0.57)
NA	7.14, 3.64	5.31, 2.05	1.83 (−1.59 to 5.25, 0.37)	7.35, 3.51	5.31, 2.05	2.05 (−0.78 to 4.87, 0.18)

PCT = preventive cognitive therapy, ADM = antidepressant medication, 95% CI = 95% family-wise confidence level, PA = aggregated positive affect, NA = aggregated negative affect, fluc. = fluctuation, SD = standard deviation, WPV = within-person variance, AC = autocorrelation, RMSSD = root mean square of successive differences.

**Fig. 2:**
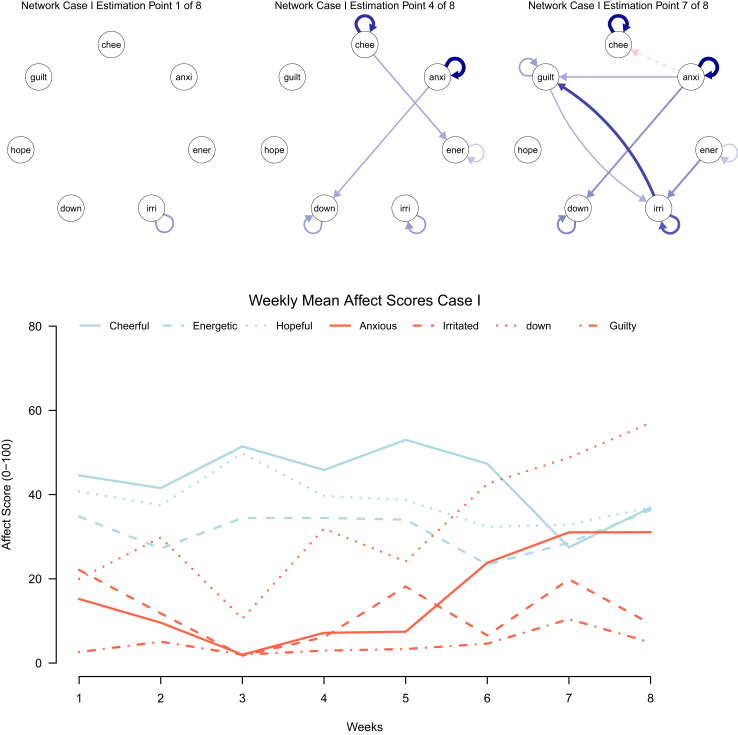
**Affect networks and weekly mean affect scores for case I**. chee = cheerful, anxi = anxious, ener = energetic, irri = irritated, down = feeling down, hope = hopeful, guilt = feeling guilty.

**Fig. 3:**
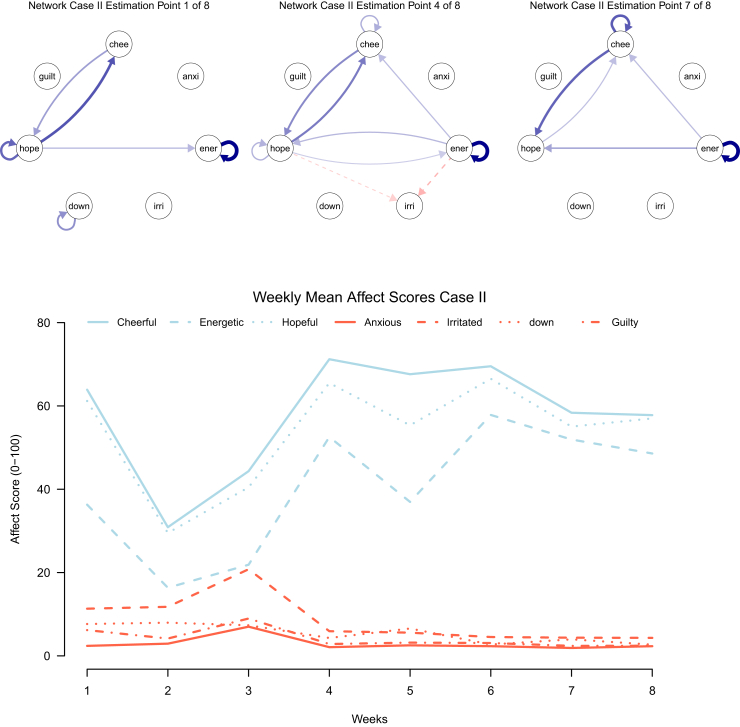
**Affect networks and weekly mean affect scores for case II**. chee = cheerful, anxi = anxious, ener = energetic, irri = irritated, down = feeling down, hope = hopeful, guilt = feeling guilty.

We estimated TV-VAR models for all 42 participants, both with and without applying LASSO regularisation. The corresponding temporal affect networks can be found in the Supplementary Figures. Upon visual inspection of the networks, no distinct trajectories in how temporal affect networks changed over time were observed for individuals within the same treatment groups, nor for participants who relapsed versus participants who remained in remission (including five participants who experienced relapse during or shortly after the ESM period). Instead, we observed large individual differences in network structure between participants across different groups. To showcase what type of information the TV-VAR models recovered from the ESM data, we describe two individual cases below (Figs. 2 and 3).

We found no differences in average change over time in temporal affect network structure between different groups, either according to treatment condition (both intention-to-treat and as-treated) or relapse status at follow-up, in both the regularised and non-regularised TV-VAR models (see Supplementary Tables S3 and S4 for average change in temporal affect networks per group and Supplementary Table S5 for all group-level comparisons). As shown in Table 3, average change in affect network structure over time (ie, mean SD) was not predictive of time to relapse (regularised models: HR 1063, 95% CI <0.0001–>10,000, p = 0.65; non-regularised models: HR 2.54, 95% CI 0.23–28.7, p = 0.45), nor for occurrence of relapse at the short term (ie, within three months; regularised models: OR >10,000, 95% CI <0.0001–>10,000, p = 0.47; non-regularised models: OR 14.81, 95% CI 0.03–3375.99, p = 0.32) or during complete follow-up (ie, up to 24 months; regularised models: OR 22.84, 95% CI <0.0001–>10,000, p = 0.90; non-regularised models: OR 7.57, 95% CI 0.07–3709.54, p = 0.44).

**Table 3:** Logistic regression models and cox proportional hazard models relating non-regularised and regularised mean SDs^a^ to occurrence of–and time to–relapse.

	Logistic regression model I: relapse <3 months (n = 5)			Logistic regression model II: relapse <2 years (n = 25)			Cox proportional hazard model: time to relapse		
	Beta (SE)	OR (95% CI)	p	Beta (SE)	OR (95% CI)	p	HR	95% CI	p
Non-reg TV-VAR mean SDs	2.70 (2.72)	14.81 (0.03–3375.99)	0.32	2.02 (2.62)	7.57 (0.07–3709.54)	0.44	2.54	0.23–28.7	0.45
Regularised TV-VAR mean SDs	26.63 (37.00)	>10,000 (<0.0001–>10,000)	0.47	3.13 (25.53)	22.84 (<0.0001–>10,000)	0.90	1063	<0.0001–>10,000	0.65

SD = standard deviation, SE = standard error, OR = odds radio, HR = hazard ratio, CI = confidence interval; TV-VAR = time-varying vector autoregression.

a Mean SD: reflects the average change in network structure over time, see methodological appendix for more details.

### Two case descriptions showcasing output of the TV-VAR models

The networks shown for Case I (Fig. 2) are three of the eight networks recovered from a regularised TV-VAR model of an individual who was allocated to receive PCT while continuing ADM, and who relapsed during the ESM period at day 16. The bottom graph (recovered from ESM responses) shows the mean affect scores per week for this participant. We observe an increase in feeling down from week three onward, as well as an increase in anxiety between week five and week seven, which may be a reflection of the relapse in MDD. The first network shows little temporal interaction, with only a positive self-loop (ie, solid blue arrow) for irritation. In the following weeks, the number of interactions start to increase, with most notably strong positive self-loops for cheerfulness and anxiety, as well as positive links from cheerfulness to feeling energetic and from anxiety to feeling down. The third network shown, at the fore last estimation point 7, shows a positive link from anxiety to guilt and to feeling down, as well as a negative link (ie, dashed red arrow) from anxiety to cheerfulness. In addition, there are reciprocal positive links between irritation and guilt, as well as self-loops for these affect items.

The networks shown for Case II (Fig. 3) are three of the eight networks recovered from a regularised TV-VAR model of an individual who remained in remission during the ESM period and the follow-up period, and received PCT while continuing ADM. The graph below (recovered from ESM responses) displays the mean affect scores per week for this participant. We observe stable low negative affectivity, possibly reflective of the favourable clinical status of this participant. While the positive affect measures seem to take a dip at week 2, they subsequently increase and seem to remain stable. Notably, most interactions are positive reciprocal or self-loop (ie, solid blue arrows) interaction between the three positive affect items in the network (ie, energetic, hopeful, cheerful). In addition, the second network, from estimation point 4 (out of 8), shows negative links (ie, dashed red arrow) from energetic and hopeful to irritation, indicating that higher levels of the first two positive affect items predict lower levels of irritation at the next time point.

## Discussion

This exploratory study provides no indication of a differential impact of various relapse prevention interventions on temporal affect dynamics (ie, both affect fluctuations and changes over time in individual affect networks), nor for the predictive value of these dynamics for MDD relapse. To our knowledge, this is the first study to exploit a randomised setting to explore whether temporal affect dynamics are differentially impacted by various distinct relapse prevention strategies (ie, psychological vs. pharmacological vs. their combination) and predict relapse during long-term follow-up (24 months), in participants with remitted recurrent MDD (n = 42). In addition, this is the first study in a sample of individuals with remitted recurrent depression at high risk of relapse to estimate change over time in temporal affect networks using TV-VAR models, which allowed us to explore within-individual changes in affect network structure over the course of the study period.

In our sample, we found no indication that fluctuations in NA or PA were differentially impacted by different treatment strategies in a randomised pragmatic setting, nor that any of the fluctuation measures (ie, WPV, RMSSD, AC of positive or negative affect items) were predictive of occurrence of–or time to–relapse, even for affect items reflective of core MDD symptoms (eg, feeling down, cheerful, or enthusiastic). Upon visual inspection of the individual temporal affect networks, we identified no distinct patterns or trajectories in how the networks changed over time at a group-level (eg, in participants who experienced short-term relapse or in participants receiving similar treatment). Instead, we observed large individual differences in network structure across all participants, and even healthy controls (n = 11, Supplementary Figures). In addition, we found no indication of a difference in how much the network structure on average changed over time between the randomised treatment conditions or between the participants who relapsed versus those who remained in remission, nor that average change in affect network structure over time was predictive of occurrence of–or time to–relapse.

Our findings appear to be in contrast with previous studies suggesting that affect fluctuations may predict an increase in depressive symptoms. For example, two ESM case-studies suggested that an increase in affect score variance and autocorrelation (ie, ‘critical slowing down’) precedes an increase in depressive symptoms.^12^^,^^13^ However, in larger samples, evidence regarding affect fluctuation as predictor of increase in depressive symptoms remains mixed. Indeed, in a study in pregnant women (n = 19), fluctuation in NA or PA at the beginning of the pregnancy was not predictive of depressive symptomatology at 36 weeks gestation.^35^

Also, the fact that we found no indication of a significant difference in NA or PA fluctuation between the different treatment conditions was not in line with our tentative expectation of an increase of PA (and thus higher PA variability) in the PCT groups, or that tapering of ADM could lead to higher levels of affect fluctuation.^20^ With regard to the latter, it could be that fluctuations were (partly) mitigated by components of PCT that were aimed at emotion regulation.^19^ However, we did observe a decrease of mean NA in the treatment-as-usual group, in contrast with our expectation that this group would not see major changes in affect scores. It may be that PCT temporarily increased negative affect, for example due to reflecting on potential negative beliefs and cognitions. Notably, it has been argued that, from a dynamic system perspective, a temporary increase in eg NA is necessary to facilitate a transition of a system into a more healthy state.^36^ Nonetheless, our findings raise the question to what extent different treatment modalities with distinct target points (ie, pharmacological versus psychological) have meaningful impact on affect fluctuations as measured by ESM in individuals with remitted recurrent depression.

Our finding of substantial heterogeneity in temporal affect network structure across all participants, including never-depressed healthy controls, extends previous findings in the same sample of large individual differences in temporal NA trajectories.^16^ How to explain this heterogeneity remains an open question. It may be that individuals interpret questions and response scales differently,^37^ or the relationship between the networks and MDD outcome could be so person-specific that idiographic approaches (such as illustrated by the two case descriptions in this article) might be more appropriate than approaches aimed at identifying group-level patterns in affect dynamics. However, our results also stress the need to first establish the clinical benefit of such ideographic approaches in a prospective setting, especially when these are implemented in the context of individualised treatment.^17^ Given our non-significant results, future prospective studies on this topic may benefit from expanding on the methodology and measurements used in this study, for example by not only examining predictive value of fluctuation measures in themselves, but also relate changes in these measures (eg a change in variance and/or inertia over time) to future clinical course of MDD.^12^, ^13^, ^14^ In addition, when considering ideographic approaches in the personalisation of treatment, it may be interesting to examine the dynamic interaction between affect dynamics and other possible individual predictors of treatment response, such as self-reported self-efficacy.^38^

Additionally, we found no indication of a difference in average change in temporal affect network structure over time between the different treatment groups or between participants who relapsed versus those who remained in remission. This is not in line with our tentative expectation that tapering ADM may lead to higher levels of change in affect dynamics over time.^20^ It could be that such changes were mitigated by components of PCT. However, if this were the case, the positive impact of adding PCT to continuation of ADM, observed in the DRD trial, might lead to the expectation that this group would significantly differ from the other treatment groups in this ESM study as well. Hence, while the positive impact of PCT through affect regulation is supported by a recent neuroimaging study,^39^ the question whether PCT also has a positive impact via changes in temporal affect dynamics remains unanswered. Additionally, our findings cast some doubt on the relevance of individual affect networks in informing risk of MDD relapse. This is further supported by a post-hoc analyses where we tentatively compared, but found no difference in, average change in affect network structure of participants who relapsed (n = 25) with a group of never-depressed healthy controls (n = 11) (Supplementary Table S5).

This was an exploratory study with several limitations and these results should therefore be interpreted with caution. First, only 47% of participants randomised to taper ADM were able to (fully or partially) do so during the ESM period, which could impact the results. However, as shown in the results section, the intention-to-treat an as-treated analysis amounted to comparable conclusions. Nonetheless, the tapering protocol, in line with international guidelines at the time,^23^ proved not feasible for most participants, as 41% of the participants randomised to the tapering group (n = 7 out of 17) was using their baseline ADM dosage after six months. Thus, more research is needed to establish the most optimal ADM tapering strategy in recurrent MDD, which may be person-specific. Second, this was an exploratory study with a relatively small number of participants, although the sample size was larger than in most studies looking at affect fluctuations and MDD relapse. Still, between-group differences cannot be ruled out given limited power and we should therefore be cautious to draw firm conclusions. Third, as pointed out in Fig. 1, there was differential dropout between the three treatment groups, both with regard to early dropout (ie, n = 21 before start of data collection) as well as during ESM data collection (ie, n = 9 excluded from analyses due to low number of responses). This may introduce bias, especially if participants who dropped out showed a different clinical profile with regard to risk of relapse. We have no indication that there was a difference in baseline severity characteristics between the n = 9 participants who were excluded from this ESM study due to low number of responses and n = 42 participants included in the analyses, both with regard to baseline HDRS-17 (medians 4 and 2, respectively; W = 261.5; p = 0.0714) as well as number of previous depressive episodes (medians 3 and 4.5, respectively; W = 123.5, p = 0.1028). However, since were are unable to draw comparisons to the n = 21 participants who dropped out before the start of data collection, it remains unclear exactly how differential dropout influenced the results. Fourth, our choice to sample three days per week (ie, Thursday, Friday, and Saturday) may have influenced the results, since certain affect dynamics may have been missed by not sampling on the other days. However, after initial pilots to test the feasibility of the ESM regimen, expanding the number of sampling days was deemed too burdensome given the number of ESM questionnaires (ie, ten per day) and the eight-week length of the ESM period. Indeed, the notion that too many ESM questionnaires can be too burdensome and have a negative impact on participants has since been corroborated in several qualitative studies.^40^^,^^41^ Moreover, the sampling days were selected to still reflect both week- and weekend days.^16^ Fifth, imputation methods for missing data in psychological time-series are currently only available for stationary data.^42^ However, in line with a recent simulation study,^28^ by excluding participants with >70% missing values, we were able to include enough data points to estimate TV-VAR models for the remaining participants, as indicated by the fact that all estimations resulted in models that were time-varying to various degrees (Supplementary Table S6). While more data points may have increased the chance of identifying potentially interesting affect dynamics, expanding data collection could have overburdened participants, who were already responding receiving 10 notifications per day for 24 days in total. Future studies could benefit from exploring how missing data in non-stationary psychological time-series can be handled.

In conclusion, we observed large individual differences in affect network structure within our sample of individuals with remitted recurrent depression, irrespective of relapse status (including short-term relapse) or randomised treatment condition, and even across a group of healthy controls. Also, we found no indication of group-level differences in the average change in temporal affect network structure over time, nor was this predictive of (time to) relapse. Fluctuations in NA or PA did not seem to differ between treatment conditions in a pragmatic randomised setting, nor were affect fluctuations predictive of relapse. The non-significant results of this exploratory study stress the need for more prospective research examining the use of temporal affect dynamics (as measured by ESM), either to inform risk of relapse or personalise relapse prevention in recurrent MDD, before potential utilisation in clinical practice.

## Contributors

CLB, MHN, NSK, and CS conceptualised the study, CLB and CS acquired funding for the DRD trial and ESM data collection. JvdW, CDvB, CS, TFB, MKD, and CLB curated and prepared the data for the current analyses. JvdW, CDvB, and JMBH conducted the formal analyses. JvdW, CDvB, JMBH, AL, and CLB wrote the first drafts of the manuscript. JvdW, CvB, and CLB accessed and verified the underlying data. All authors had final responsibility for the decision to submit for publication; reviewed and edited the later version of the manuscript; and approved the final version of the manuscript.

## Data sharing statement

Data are not publicly available due to privacy reasons, but are available for research purposes upon reasonable request with the corresponding author. The full R script of all the analyses is available in a public repository on the Open Science Framework.

## Declaration of interests

CLB is co-editor of Clinical Psychology Europe and Proceedings of the European Academy of Sciences and Arts. CLB is co-developer of the Dutch multidisciplinary clinical guideline for anxiety and depression (non-remunerated) and member of the scientific advisory board of the National Insure Institute, for which she receives an honorarium (no direct relation to this study). CLB has presented keynote addresses at conferences, such as the European Psychiatry Association and the European Conference Association, for which she sometimes receives an honorarium. She has presented clinical training workshops, some of which include a fee. CLB receives royalties from her books and co-edited books, and she developed PCT on the basis of the cognitive model of AT Beck. MHN reports travel expenses, some subsistence, and speaker honoraria for lectures and clinical training workshops, and is a member of the workgroup of the Dutch multi-disciplinary guideline for anxiety (non-remunerated). All other authors declare no competing interests.

## Appendix A Supplementary data

Supplementary Figures “Temporal affect networks of all n = 42 study participants and n = 11 healthy never-depressed controls”.

Supplementary Material

Study protocol

DRD design and protocol

Trial Registration DRD study

## References

[1] Bockting C.L., Hollon S.D., Jarrett R.B., Kuyken W., Dobson K. (2015). A lifetime approach to major depressive disorder: the contributions of psychological interventions in preventing relapse and recurrence. Clin Psychol Rev.

[2] Breedvelt J.J.F., Warren F.C., Segal Z., Kuyken W., Bockting C.L. (2021). Continuation of antidepressants vs sequential psychological interventions to prevent relapse in depression. JAMA Psychiatr.

[3] Kato M., Hori H., Inoue T. (2021). Discontinuation of antidepressants after remission with antidepressant medication in major depressive disorder: a systematic review and meta-analysis. Mol Psychiatry.

[4] Bockting C.L.H., Klein N.S., Elgersma H.J. (2018). Effectiveness of preventive cognitive therapy while tapering antidepressants versus maintenance antidepressant treatment versus their combination in prevention of depressive relapse or recurrence (DRD study): a three-group, multicentre, randomised controlled trial. Lancet Psychiatr.

[5] Brouwer M.E., Williams A.D., Kennis M. (2019). Psychological theories of depressive relapse and recurrence: a systematic review and meta-analysis of prospective studies. Clin Psychol Rev.

[6] Kuyken W., Watkins E., Holden E. (2010). How does mindfulness-based cognitive therapy work?. Behav Res Ther.

[7] Schoevers R.A., van Borkulo C.D., Lamers F. (2021). Affect fluctuations examined with ecological momentary assessment in patients with current or remitted depression and anxiety disorders. Psychol Med.

[8] Beck A.T., Bredemeier K. (2016). A unified model of depression: integrating clinical, cognitive, biological, and evolutionary perspectives. Clin Psychol Sci.

[9] van Rijsbergen G.D., Bockting C.L.H., Burger H. (2013). Mood reactivity rather than cognitive reactivity is predictive of depressive relapse: a randomized study with 5.5-year follow-up. J Consult Clin Psychol.

[10] Segal Z.V., Kennedy S., Gemar M., Hood K., Pedersen R., Buis T. (2006). Cognitive reactivity to sad mood provocation and the prediction of depressive relapse. Arch Gen Psychiatry.

[11] Koval P., Pe M.L., Meers K., Kuppens P. (2013). Affect dynamics in relation to depressive symptoms: variable, unstable or inert?. Emotion.

[12] Wichers M., Groot P.C. (2016). Critical slowing down as a personalized early warning signal for depression. Psychother Psychosom.

[13] Wichers M., Smit A.C., Snippe E. (2020). Early warning signals based on momentary affect dynamics can expose nearby transitions in depression: a confirmatory single-subject time-series study. J Pers Res.

[14] van de Leemput I.A., Wichers M., Cramer A.O.J. (2014). Critical slowing down as early warning for the onset and termination of depression. Proc Natl Acad Sci U S A.

[15] Curtiss J.E., Mischoulon D., Fisher L.B. (2021). Rising early warning signals in affect associated with future changes in depression: a dynamical systems approach. Psychol Med.

[16] Slofstra C., Nauta M.H., Bringmann L.F. (2018). Individual negative affective trajectories can be detected during different depressive relapse prevention strategies. Psychother Psychosom.

[17] Bringmann L.F., Albers C., Bockting C. (2022). Psychopathological networks: theory, methods and practice. Behav Res Ther.

[18] Padesky C.A., Mooney K.A. (2012). Strengths-based cognitive-behavioural therapy: a four-step model to build resilience. Clin Psychol Psychother.

[19] Bockting C. (2009).

[20] Cosci F., Chouinard G. (2020). Acute and persistent withdrawal syndromes following discontinuation of psychotropic medications. Psychother Psychosom.

[21] Bockting C.L.H., Elgersma H.J., van Rijsbergen G.D. (2011). Disrupting the rhythm of depression: design and protocol of a randomized controlled trial on preventing relapse using brief cognitive therapy with or without antidepressants. BMC Psychiatr.

[22] Slofstra C., Klein N.S., Nauta M.H., Wichers M., Batalas N., Bockting C.L.H. (2017). Imagine your mood: study design and protocol of a randomized controlled micro-trial using app-based experience sampling methodology to explore processes of change during relapse prevention interventions for recurrent depression. Contemp Clin Trials Commun.

[23] NICE (2009). https://www.nice.org.uk/guidance/cg90.

[24] Imagine your mood: a step towards personalized prevention in depression.

[25] Wichers M., Kasanova Z., Bakker J. (2015). From affective experience to motivated action: tracking reward-seeking and punishment-avoidant behaviour in real-life. PLoS One.

[26] Watson D., Clark L.A., Tellegen A. (1988). Development and validation of brief measures of positive and negative affect: the PANAS scales. J Pers Soc Psychol.

[27] Bos E.H., de Jonge P., Cox R.F.A. (2019). Affective variability in depression: revisiting the inertia–instability paradox. Br J Psychol.

[28] Thompson C.G., Kim R.S., Aloe A.M., Becker B.J. (2017). Extracting the variance in flation factor and other multicollinearity diagnostics from typical regression results. Basic Appl Soc Psych.

[29] Haslbeck J.M.B., Waldorp L.J. (2020). MGM: estimating time-varying mixed graphical models in high-dimensional data. J Stat Softw.

[30] Haslbeck J.M.B., Bringmann L.F., Waldorp L.J. (2020). A tutorial on estimating time-varying vector autoregressive models. Multivariate Behav Res.

[31] Tellegen A., Watson D. (1985). Toward a consensual structure of mood. Psychol Bull.

[32] Smith V.A., Coffman C.J., Hudgens M.G. (2021). Interpreting the results of intention-to-treat, per-protocol, and as-treated analyses of clinical trials. JAMA.

[33] Hastie T., Tibshirani R., Wainwright M. (2015).

[34] RStudio Team (2020).

[35] Brouwer M.E., Molenaar N.M., Burger H. (2020). Tapering antidepressants while receiving digital preventive cognitive therapy during pregnancy: an experience sampling methodology trial. Front Psychiatry.

[36] Hayes A.M., Yasinski C., Ben Barnes J., Bockting C.L.H. (2015). Network destabilization and transition in depression: new methods for studying the dynamics of therapeutic change. Clin Psychol Rev.

[37] Haslbeck J., Ryan O., Dablander F. (2023). Multimodality and skewness in emotion time series. Emotion.

[38] Müller-Bardorff M., Schulz A., Paersch C. (2022).

[39] van Tol M., van Kleef R., Eike R. (2021).

[40] Bos F.M., Snippe E., Bruggeman R., Doornbos B., Wichers M., van der Krieke L. (2020). Recommendations for the use of long-term experience sampling in bipolar disorder care: a qualitative study of patient and clinician experiences. Int J Bipolar Disord.

[41] Bos F.M., Snippe E., Bruggeman R., Wichers M., van der Krieke L. (2019). Insights of patients and clinicians on the promise of the experience sampling method for psychiatric care. Psychiatr Serv.

[42] Mansueto A.C., Wiers R.W., van Weert J.C.M., Schouten B.C., Epskamp S. (2022). Investigating the feasibility of idiographic network models. Psychol Methods.

