# Supplementary material for: Differential impact of preventive cognitive therapy while tapering antidepressants versus maintenance antidepressant treatment on affect fluctuations and individual affect networks and impact on relapse: a secondary analysis of a randomised controlled trial
Source: eClinicalMedicine. 2023 Nov 22;66:102329. doi: 10.1016/j.eclinm.2023.102329 (PMC10700372; doi:10.1016/j.eclinm.2023.102329)

PCT plus ADM non−reg Pt 288 Estpoint 1

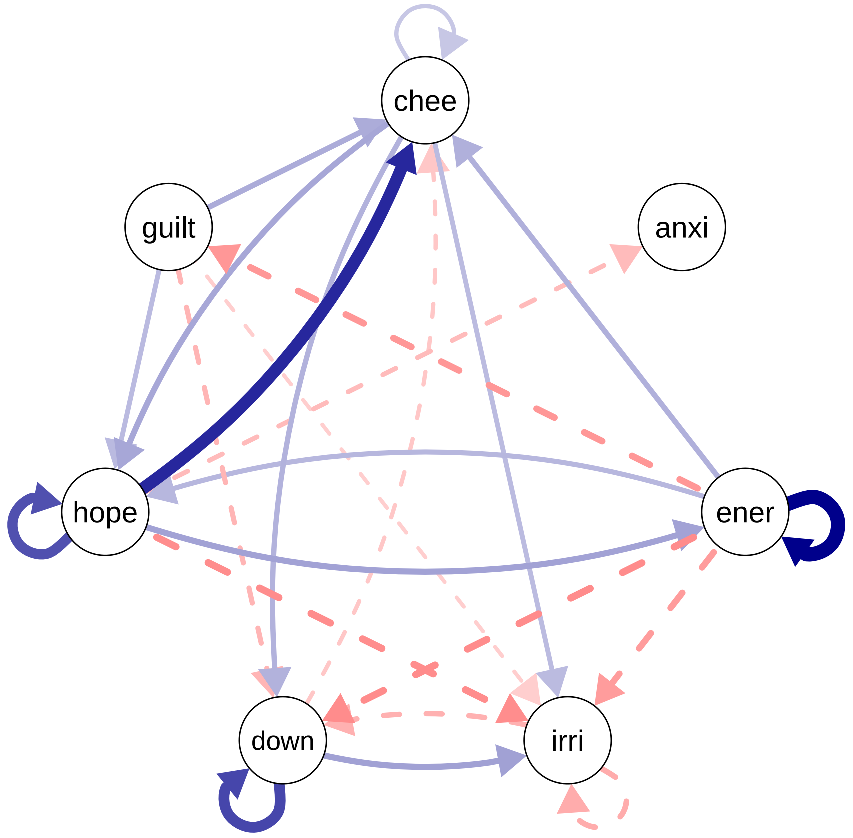

PCT plus ADM non−reg Pt 288 Estpoint 2

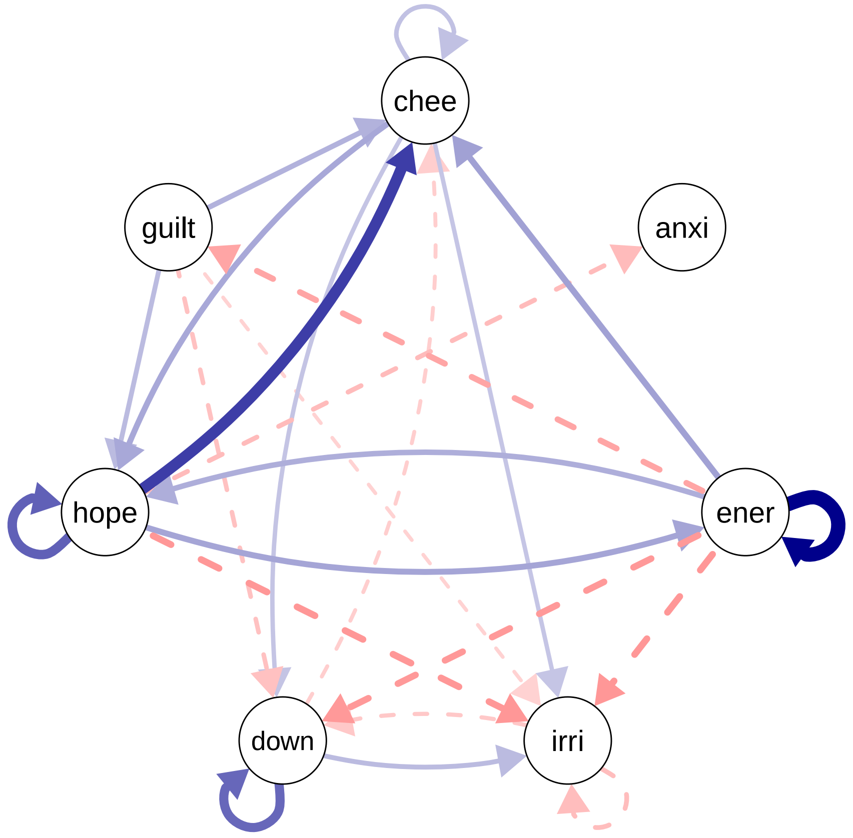

PCT plus ADM non−reg Pt 288 Estpoint 3

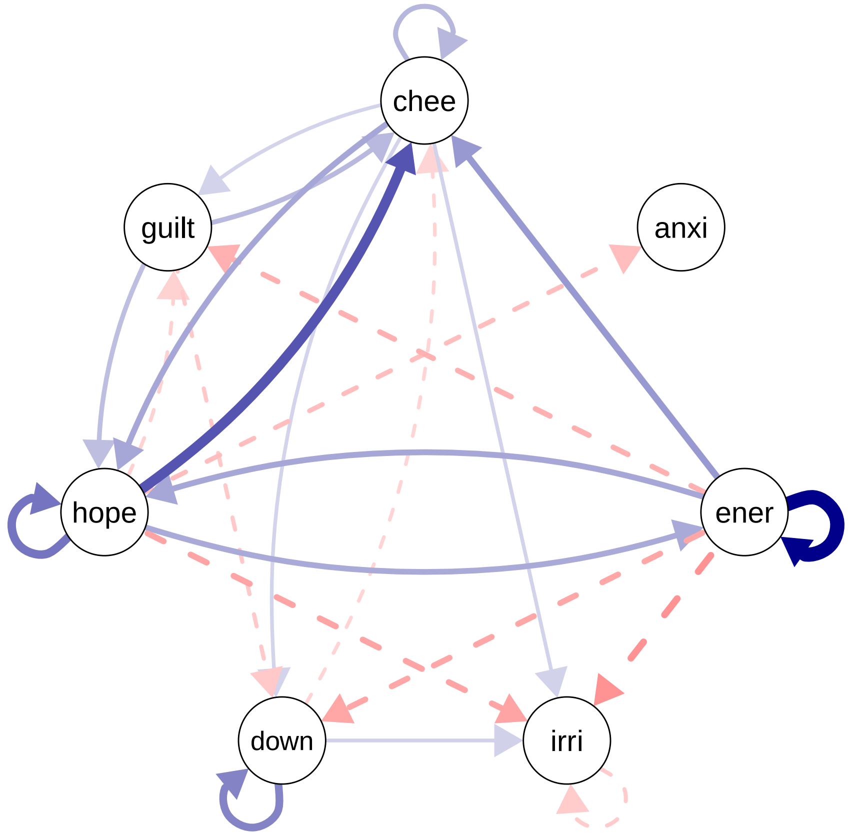

PCT plus ADM non−reg Pt 288 Estpoint 4

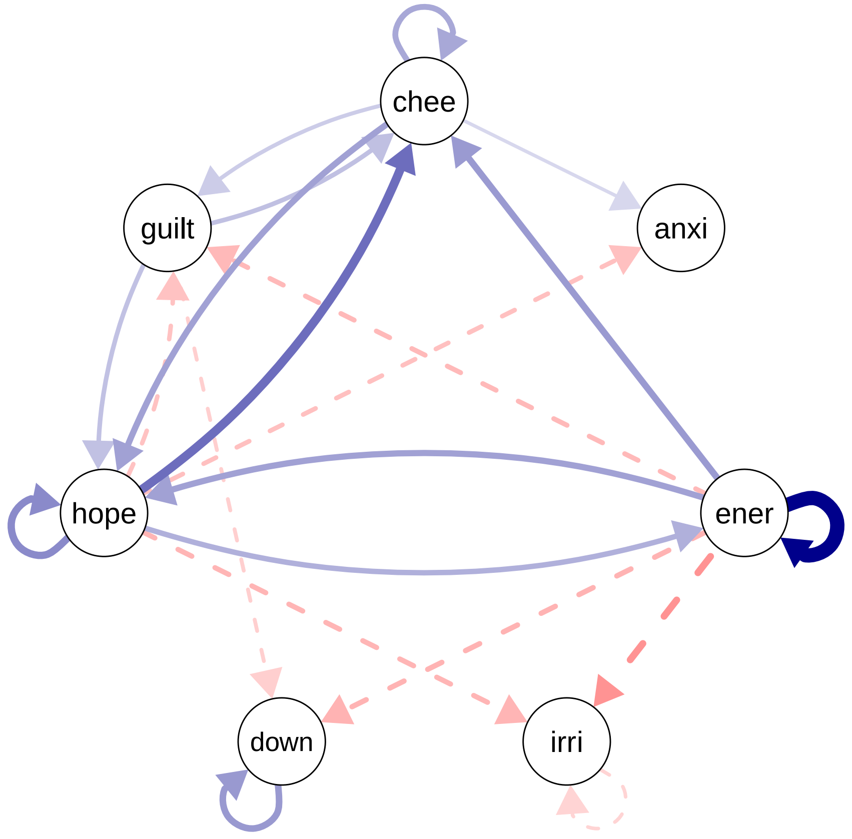

PCT plus ADM non−reg Pt 288 Estpoint 5

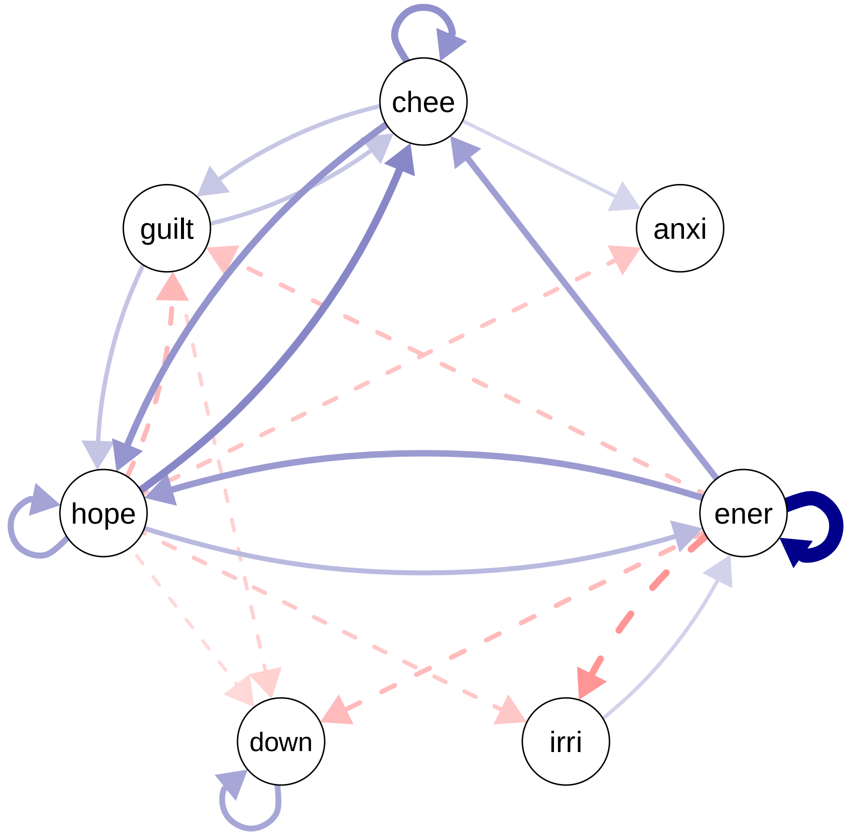

PCT plus ADM non−reg Pt 288 Estpoint 6

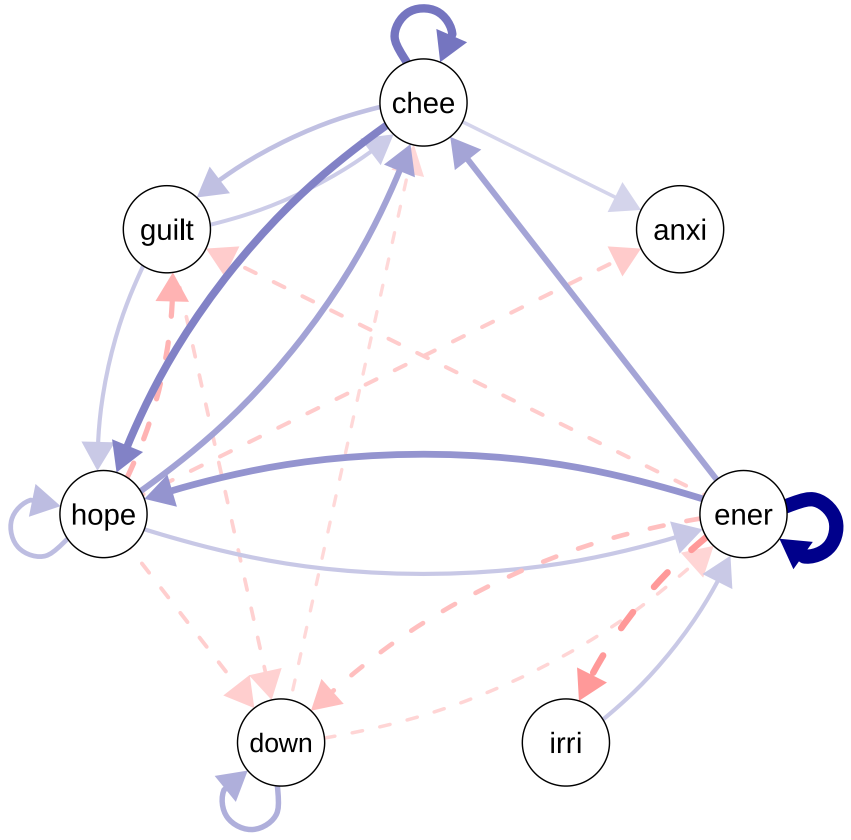

PCT plus ADM non−reg Pt 288 Estpoint 7

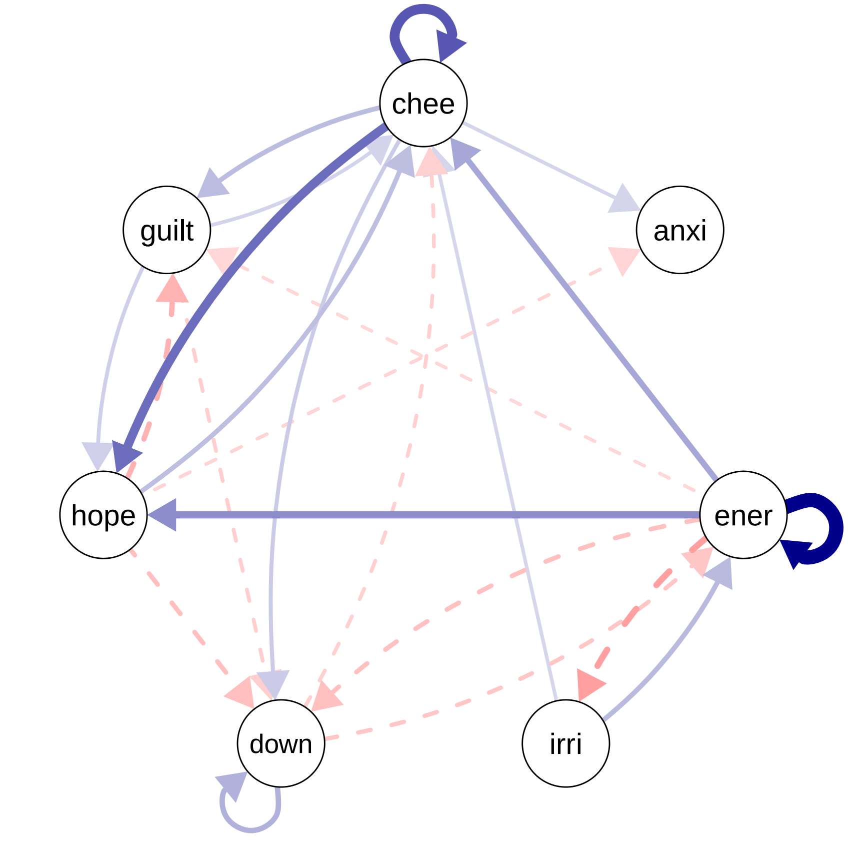

PCT plus ADM non−reg Pt 288 Estpoint 8

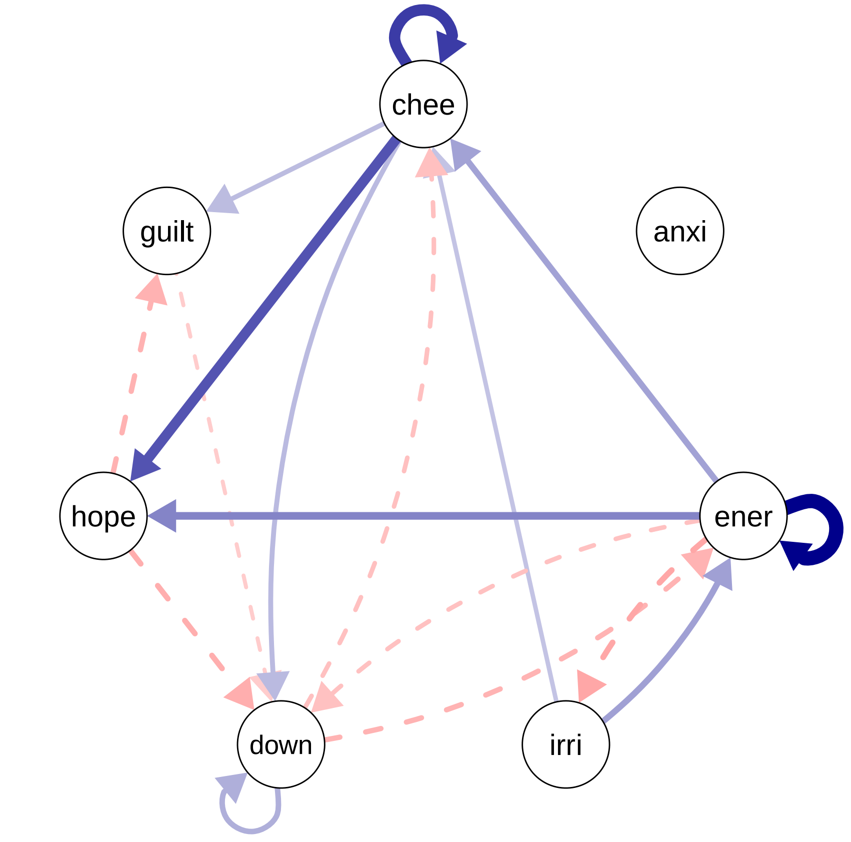

PCT plus ADM non−reg Pt 291 Estpoint 1

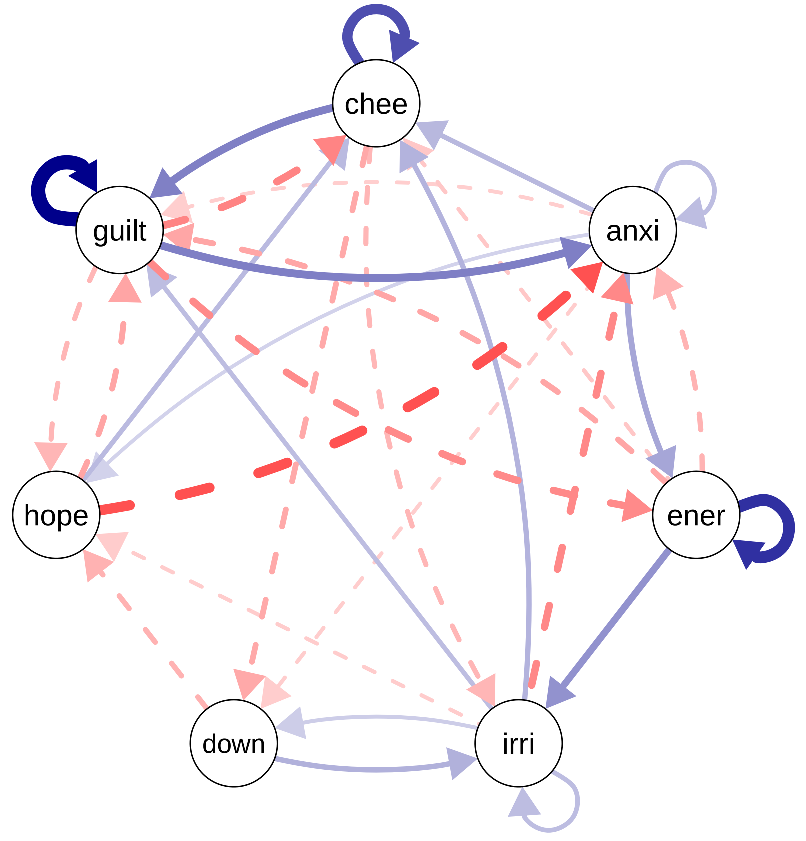

PCT plus ADM non−reg Pt 291 Estpoint 2

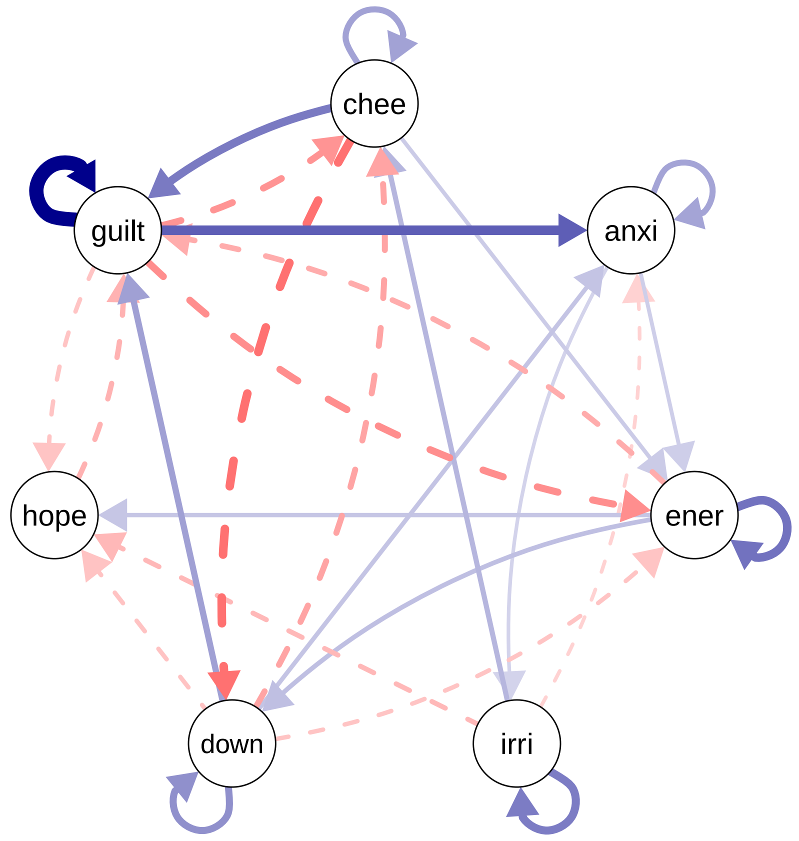

PCT plus ADM non−reg Pt 291 Estpoint 3

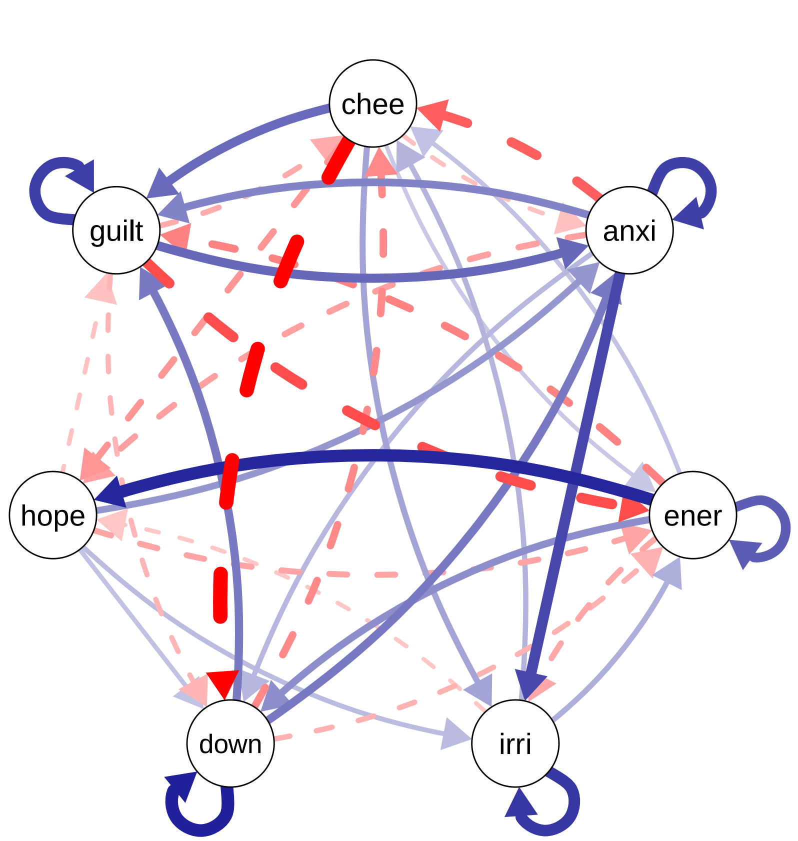

PCT plus ADM non−reg Pt 291 Estpoint 4

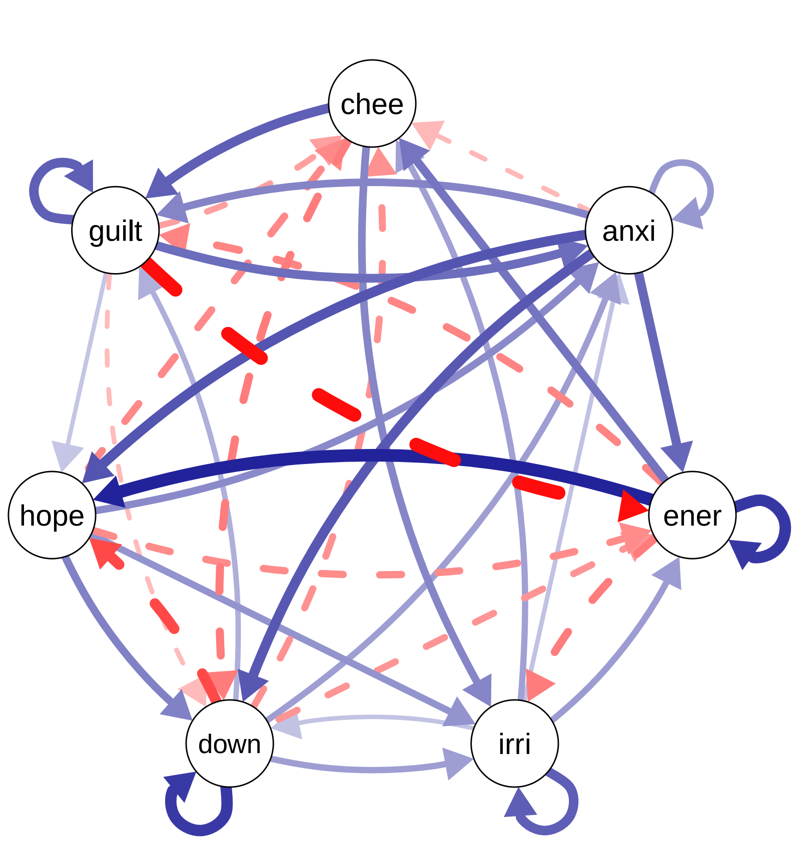

PCT plus ADM non−reg Pt 291 Estpoint 5

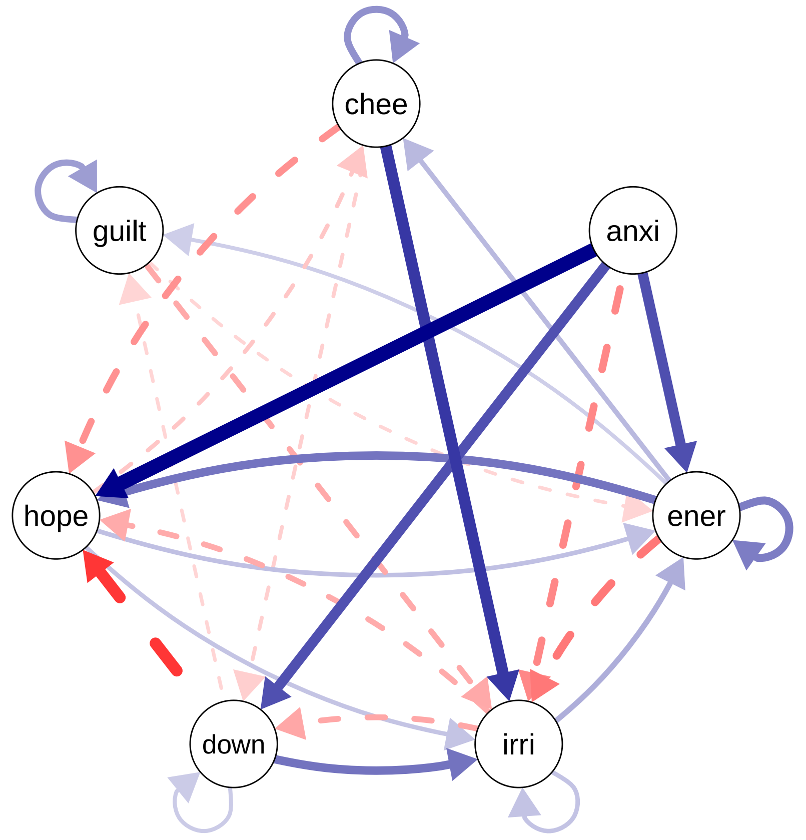

PCT plus ADM non−reg Pt 291 Estpoint 6

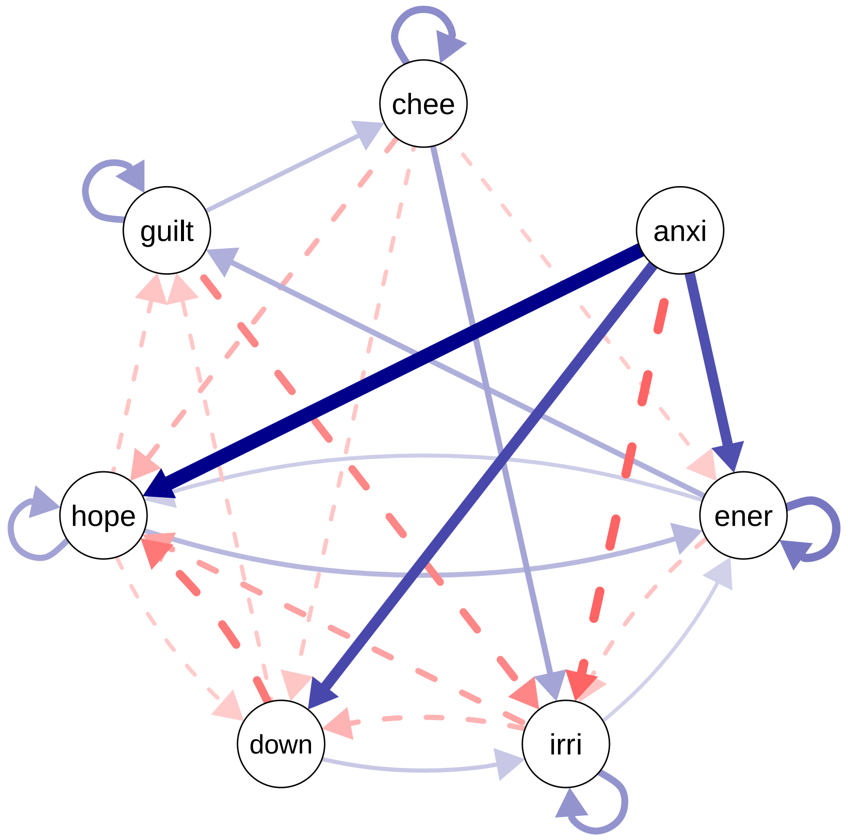

PCT plus ADM non−reg Pt 291 Estpoint 7

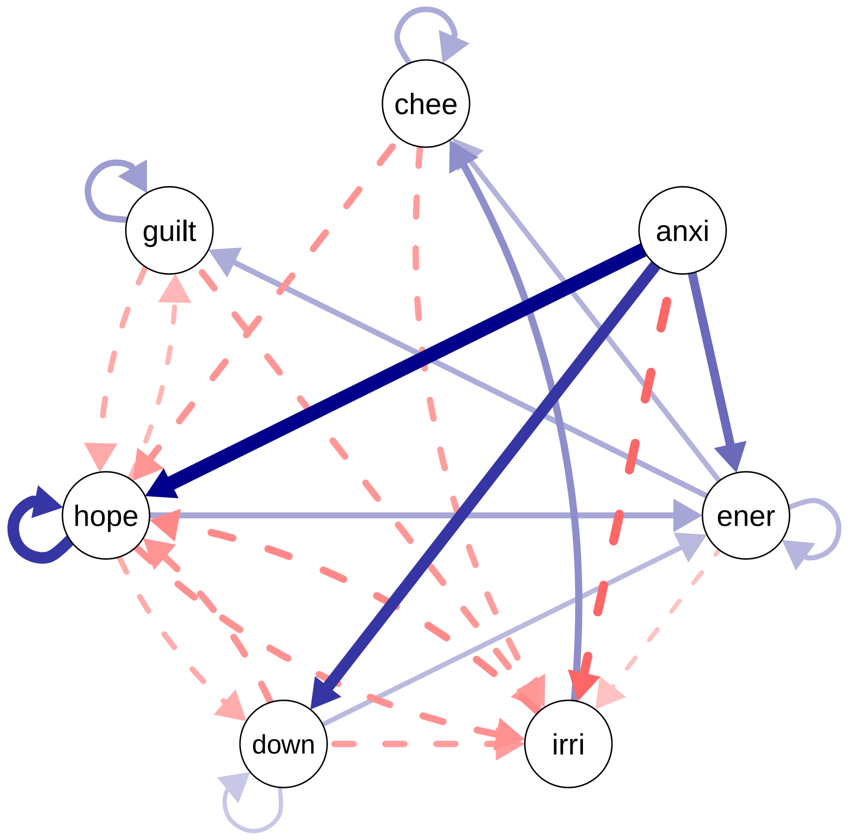

PCT plus ADM non−reg Pt 291 Estpoint 8

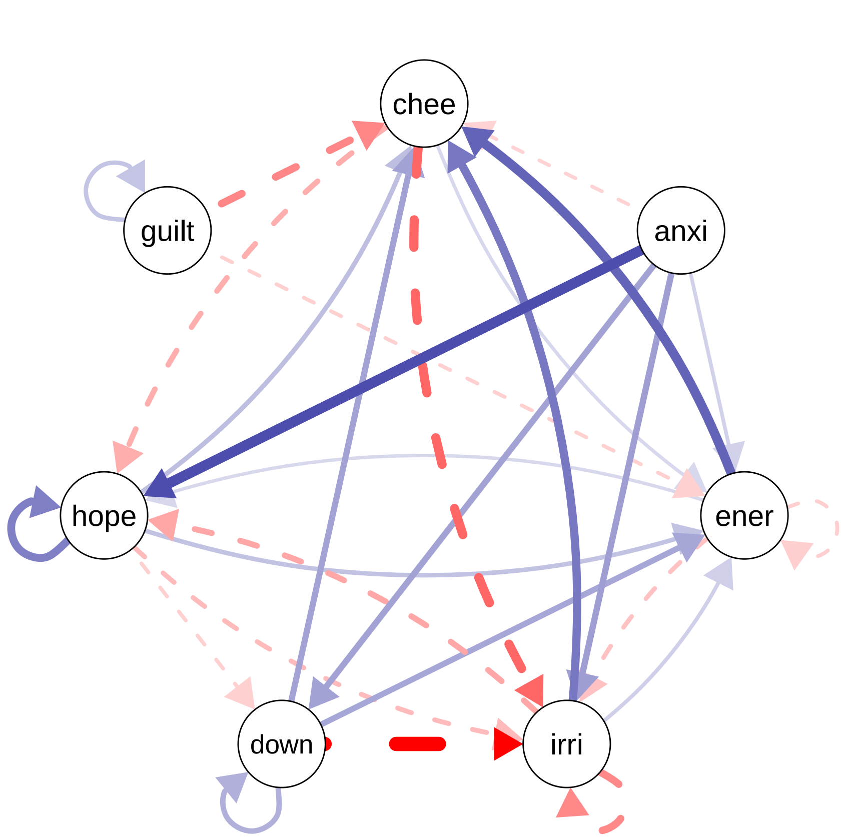

PCT plus ADM non−reg Pt 234 Estpoint 1

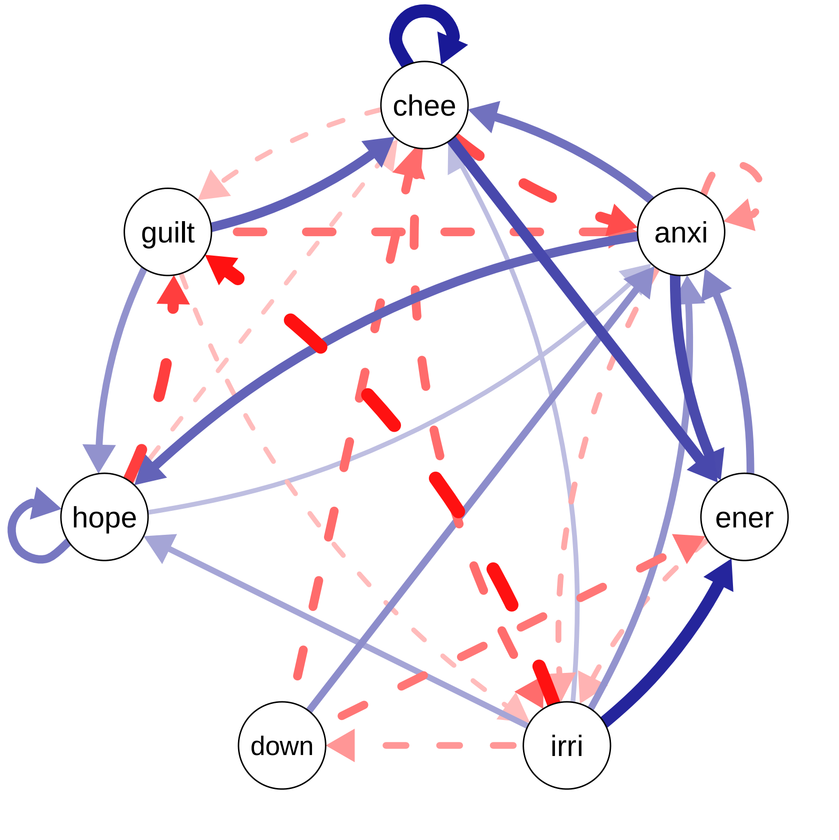

PCT plus ADM non−reg Pt 234 Estpoint 2

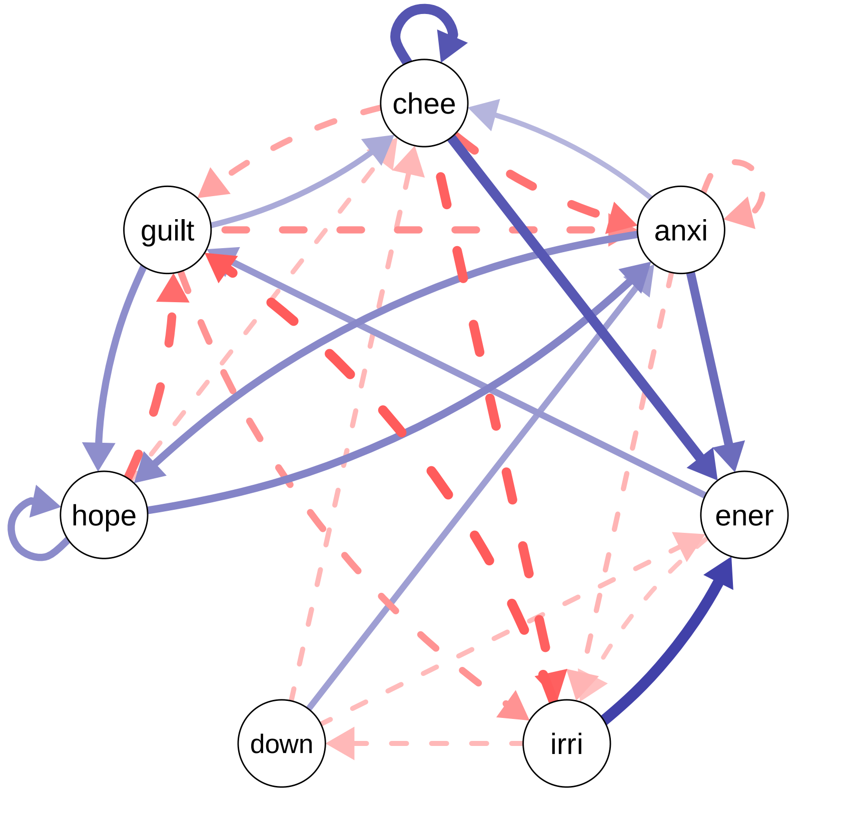

PCT plus ADM non−reg Pt 234 Estpoint 3

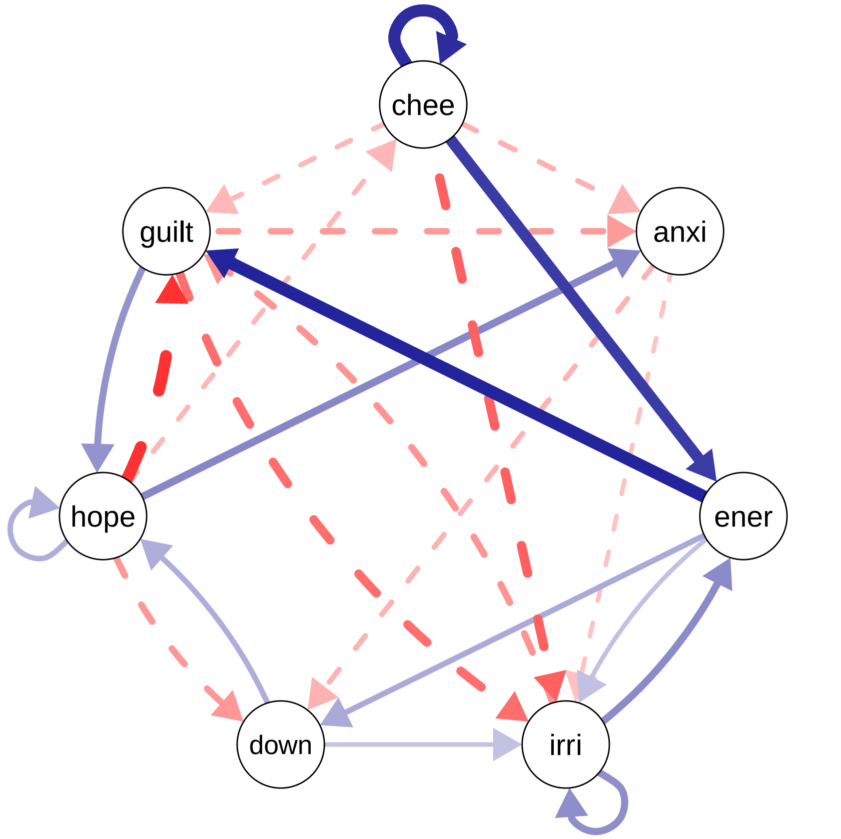

PCT plus ADM non−reg Pt 234 Estpoint 4

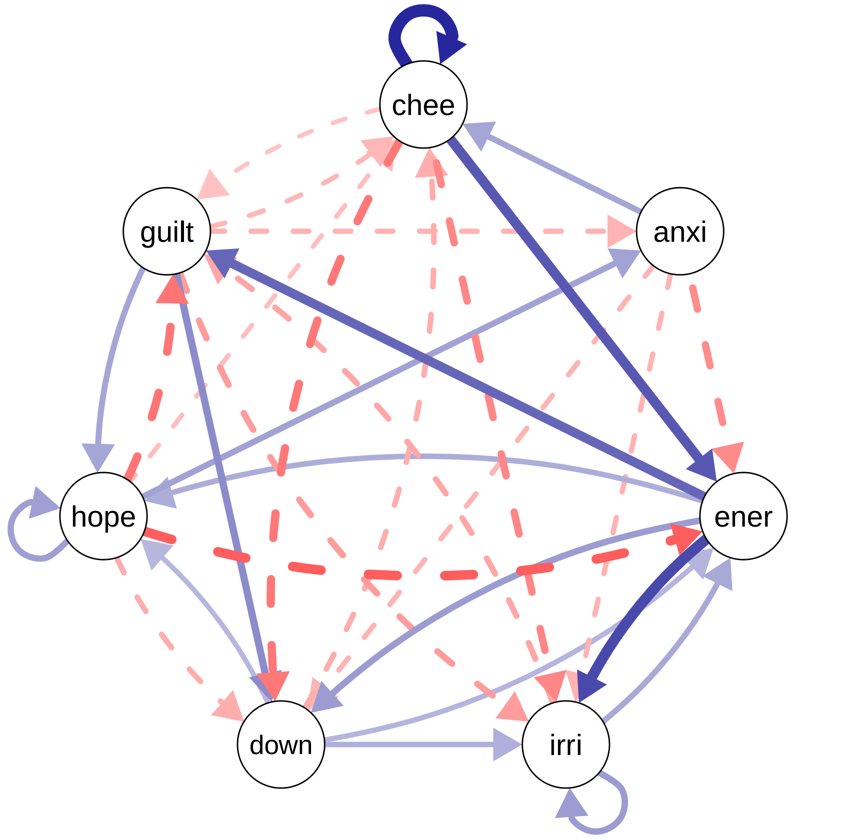

PCT plus ADM non−reg Pt 234 Estpoint 5

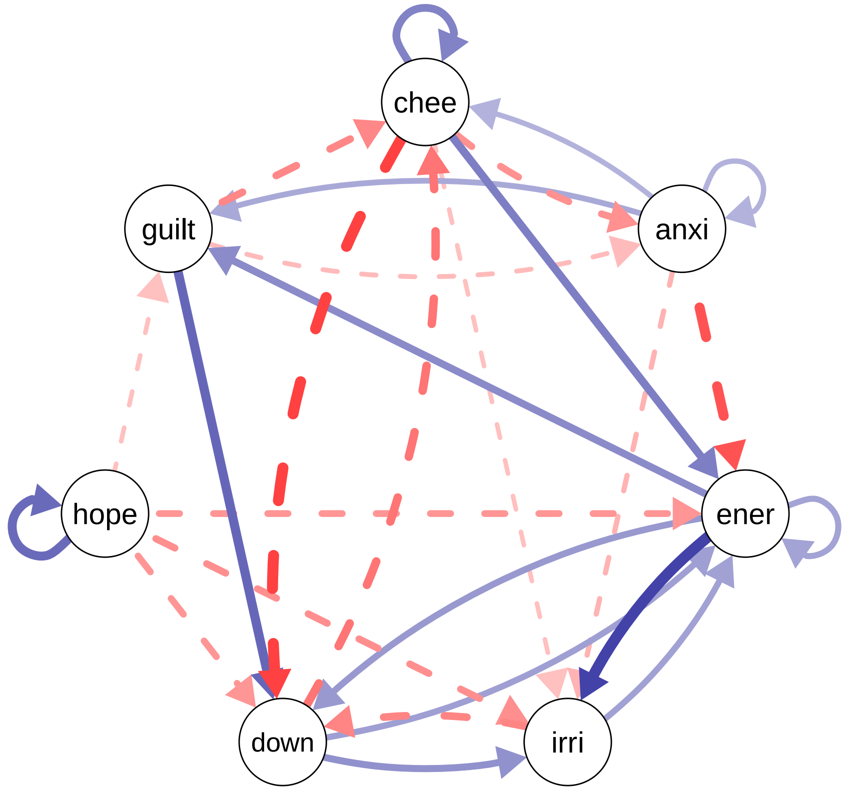

PCT plus ADM non−reg Pt 234 Estpoint 6

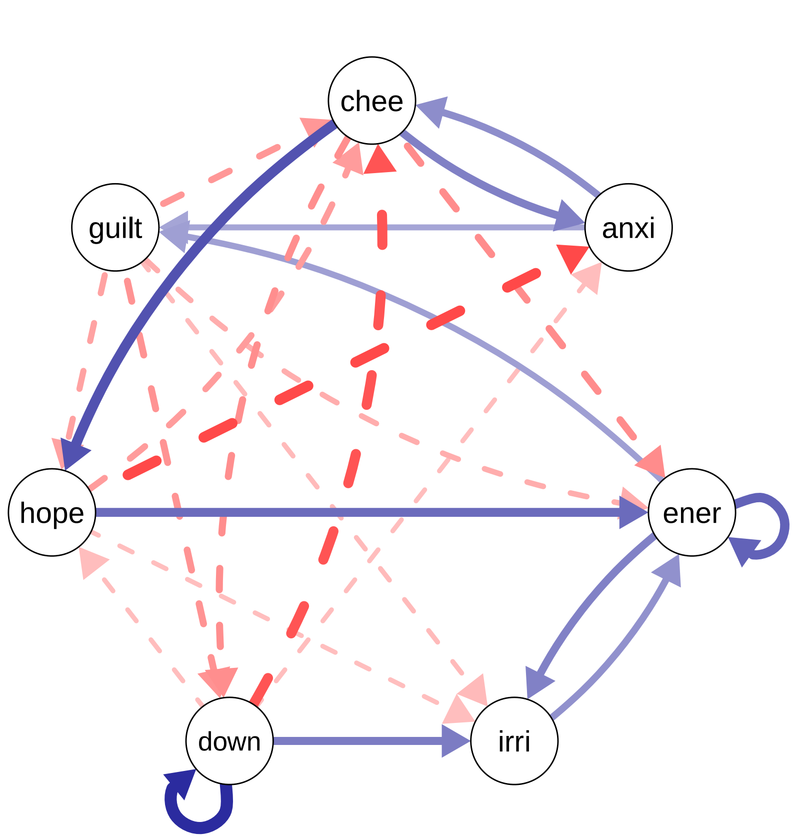

PCT plus ADM non−reg Pt 234 Estpoint 7

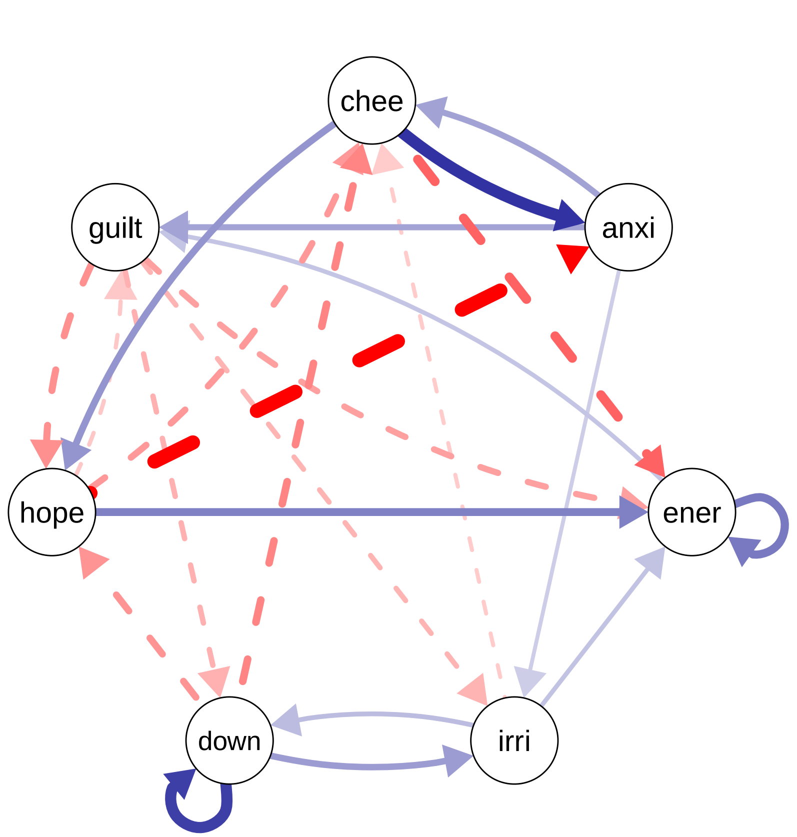

PCT plus ADM non−reg Pt 234 Estpoint 8

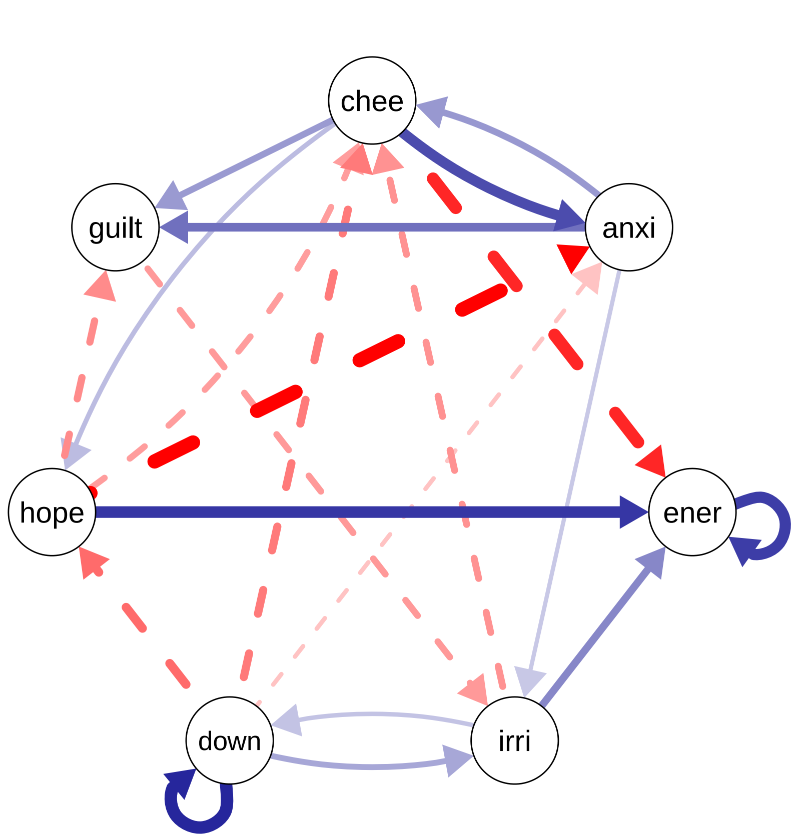

PCT plus ADM non−reg Pt 250 Estpoint 1

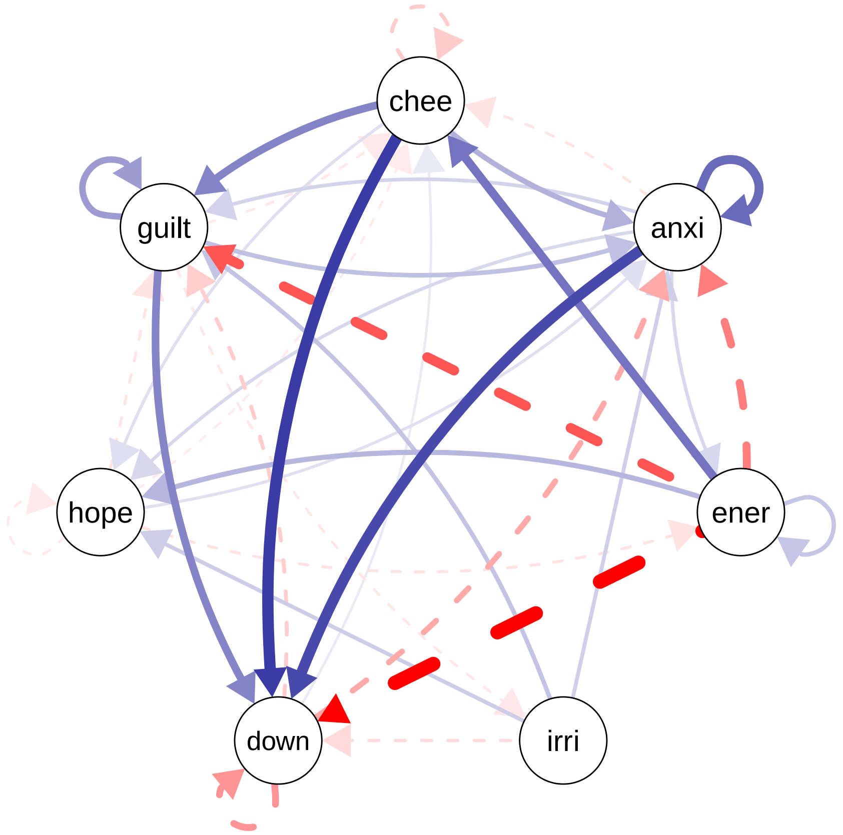

PCT plus ADM non−reg Pt 250 Estpoint 2

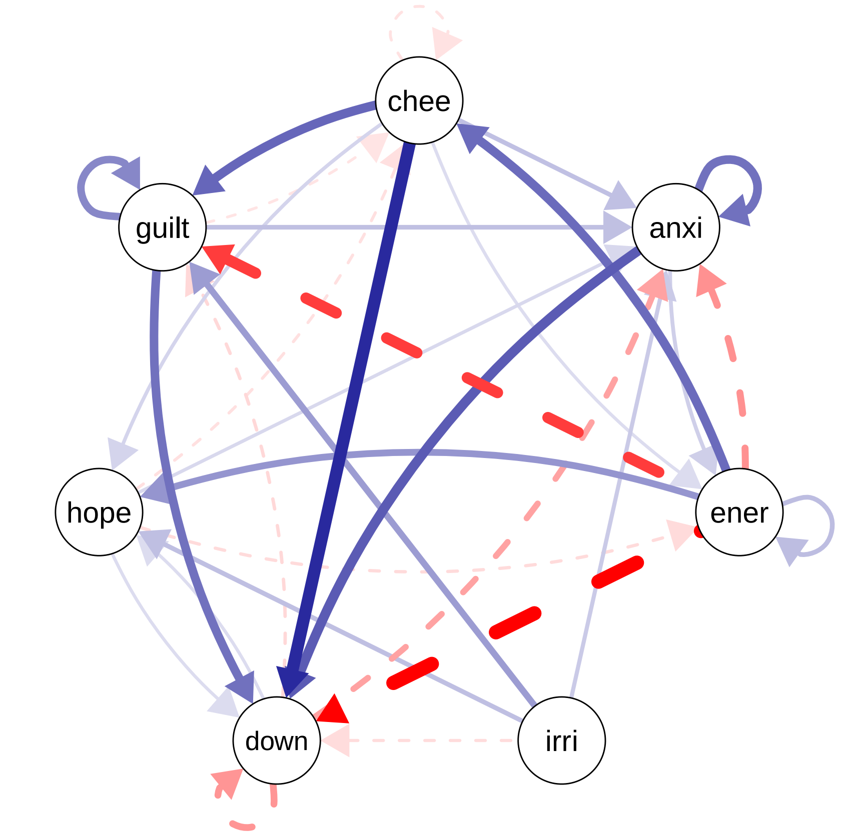

PCT plus ADM non−reg Pt 250 Estpoint 3

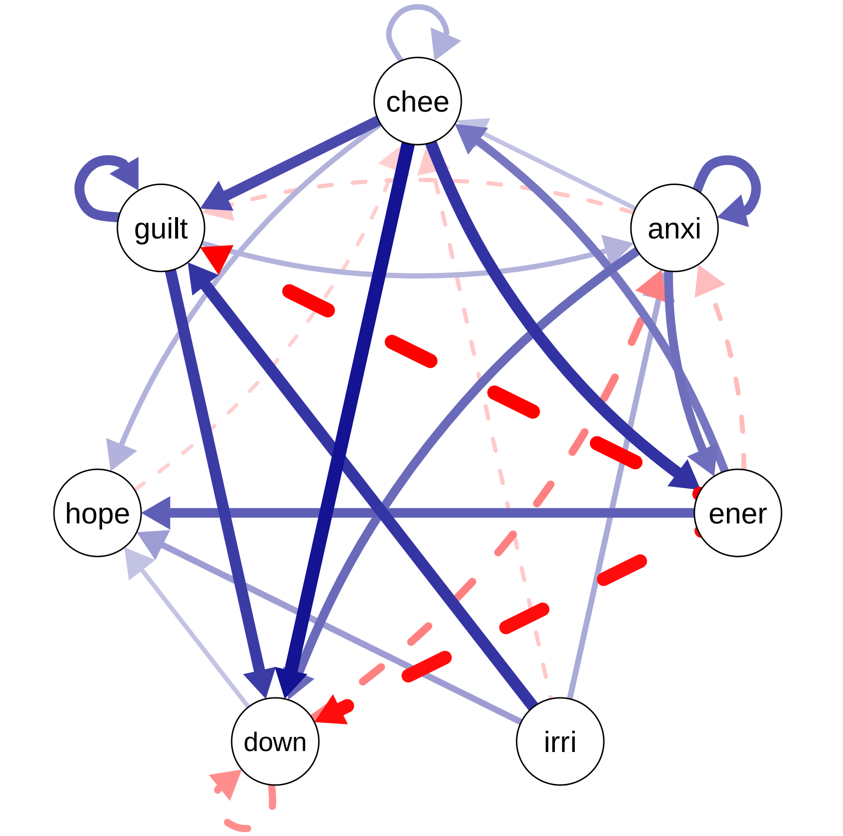

PCT plus ADM non−reg Pt 250 Estpoint 4

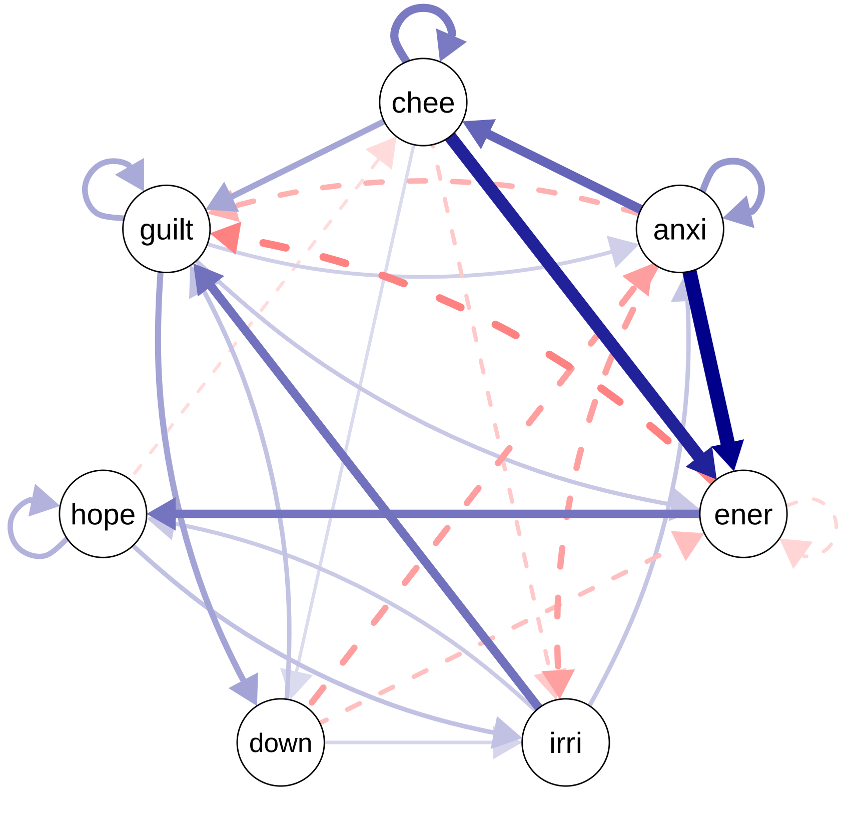

PCT plus ADM non−reg Pt 250 Estpoint 5

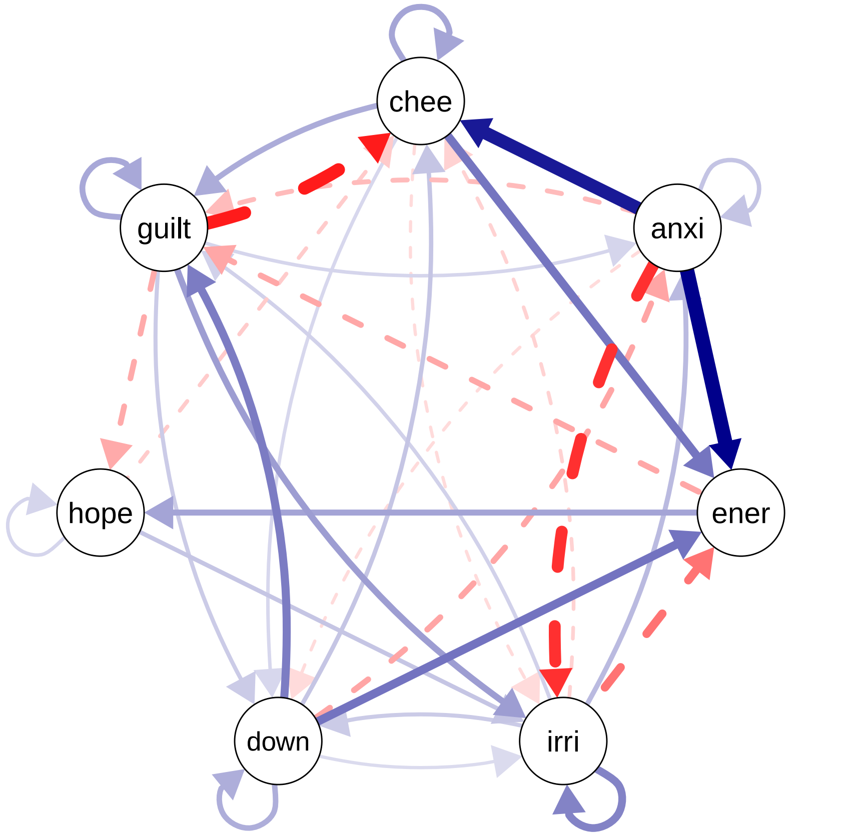

PCT plus ADM non−reg Pt 250 Estpoint 6

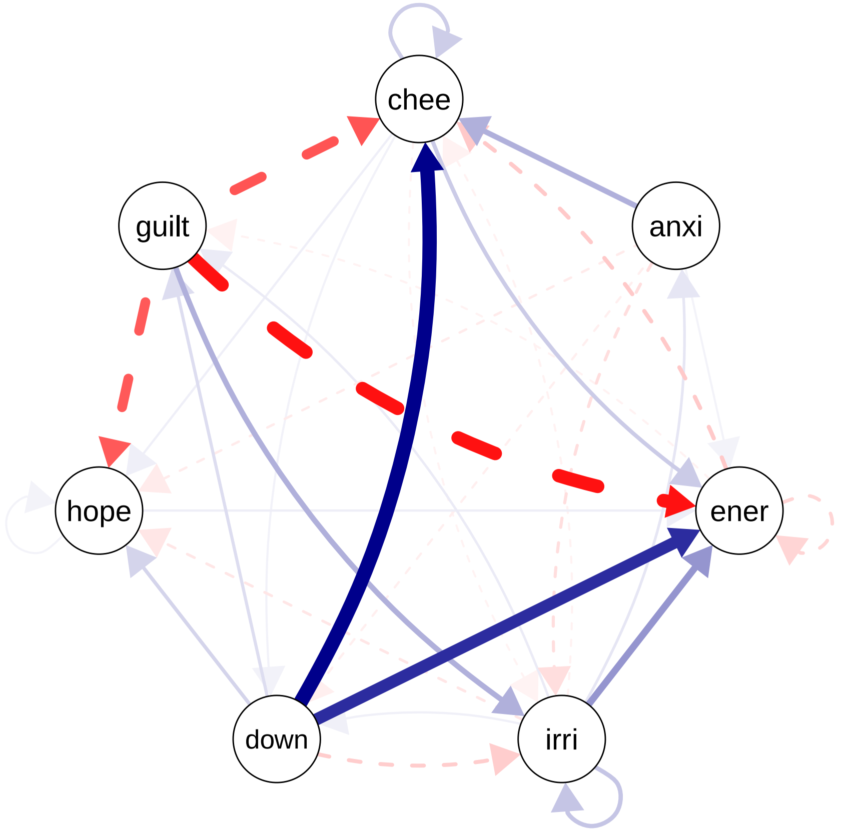

PCT plus ADM non−reg Pt 250 Estpoint 7

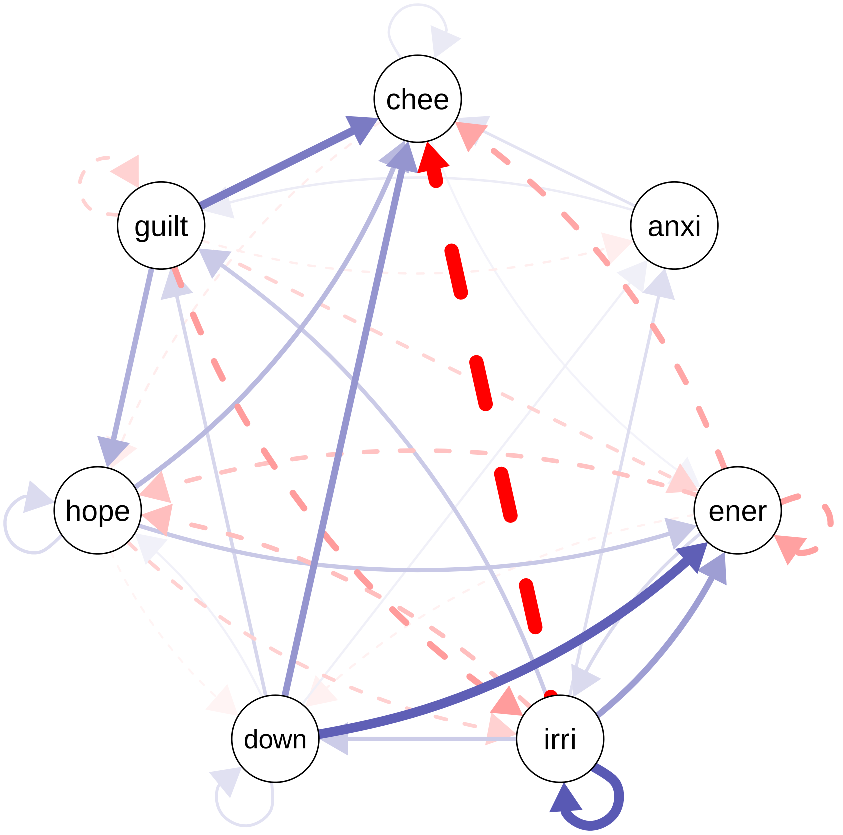

PCT plus ADM non−reg Pt 250 Estpoint 8

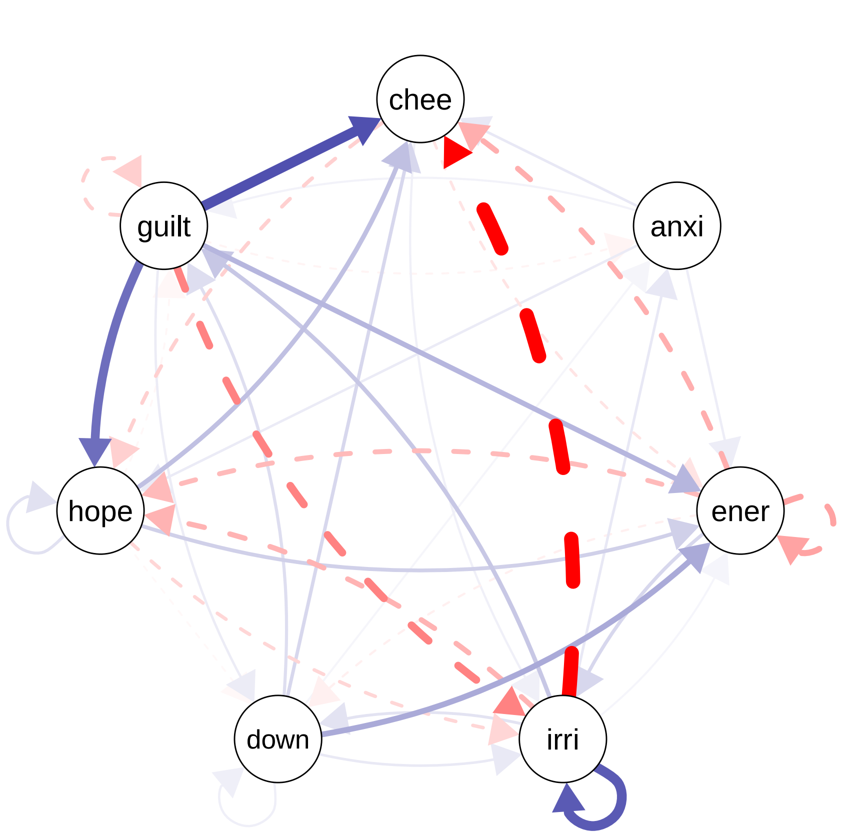

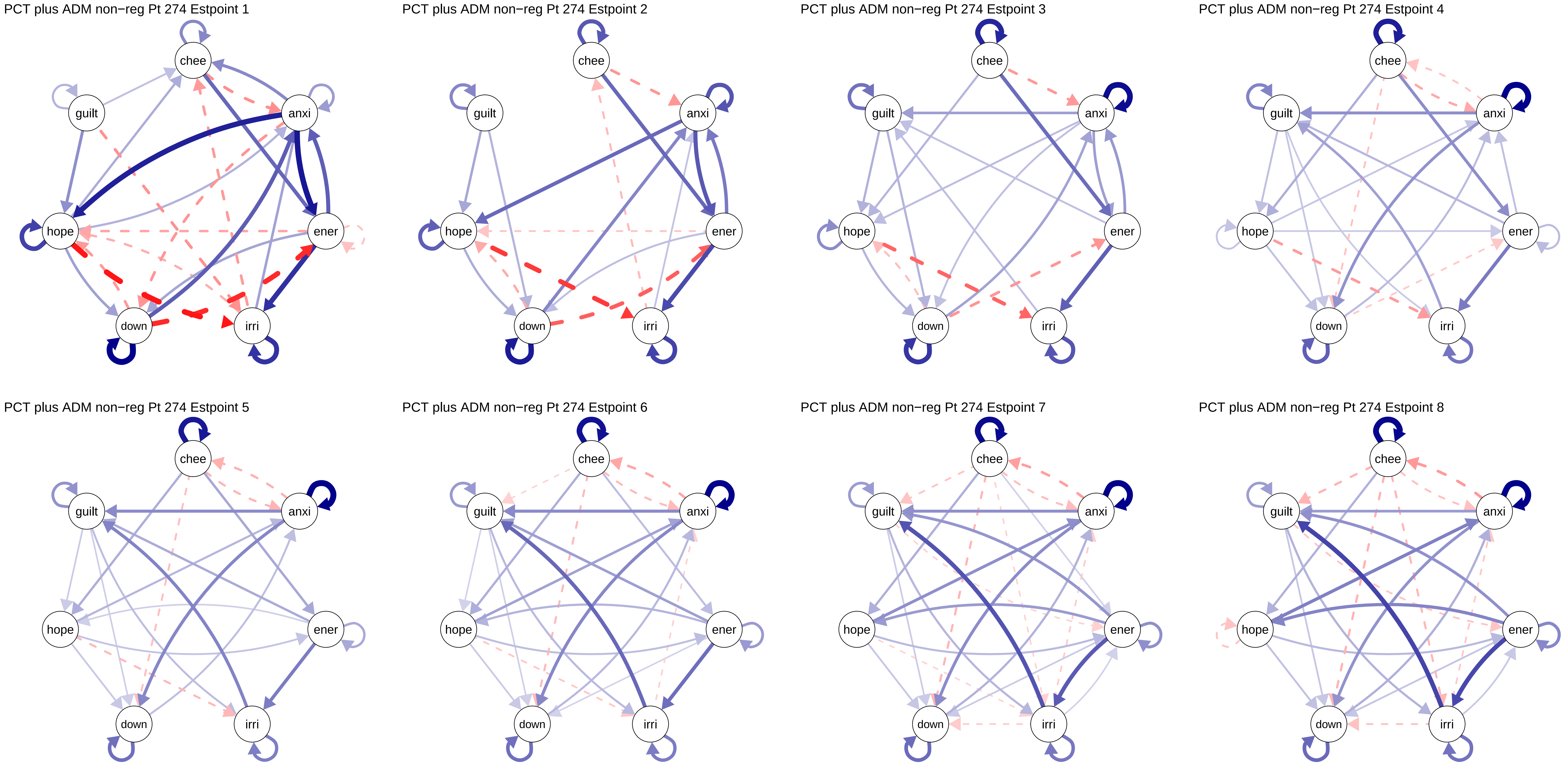
PCT plus ADM non−reg Pt 274 Estpoint 1
PCT plus ADM non−reg Pt 274 Estpoint 2
PCT plus ADM non−reg Pt 274 Estpoint 3
PCT plus ADM non−reg Pt 274 Estpoint 4
PCT plus ADM non−reg Pt 274 Estpoint 5
PCT plus ADM non−reg Pt 274 Estpoint 6
PCT plus ADM non−reg Pt 274 Estpoint 7
PCT plus ADM non−reg Pt 274 Estpoint 8
chee
guilt
anxi
hope
ener
down
irri

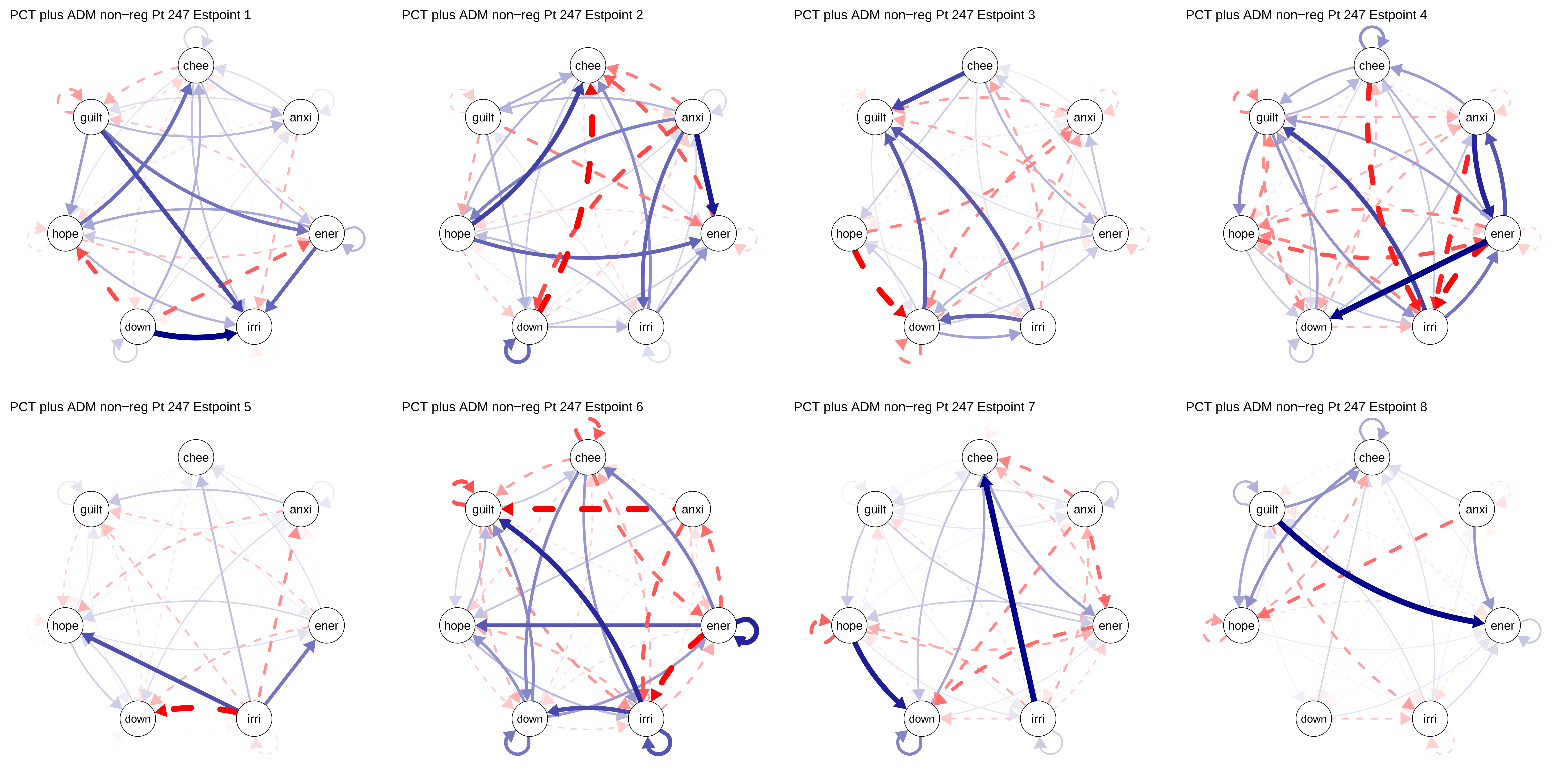
PCT plus ADM non−reg Pt 247 Estpoint 1
PCT plus ADM non−reg Pt 247 Estpoint 2
PCT plus ADM non−reg Pt 247 Estpoint 3
PCT plus ADM non−reg Pt 247 Estpoint 4
PCT plus ADM non−reg Pt 247 Estpoint 5
PCT plus ADM non−reg Pt 247 Estpoint 6
PCT plus ADM non−reg Pt 247 Estpoint 7
PCT plus ADM non−reg Pt 247 Estpoint 8
chee
guilt
anxi
hope
ener
down
irri

PCT plus ADM non−reg Pt 225 Estpoint 1

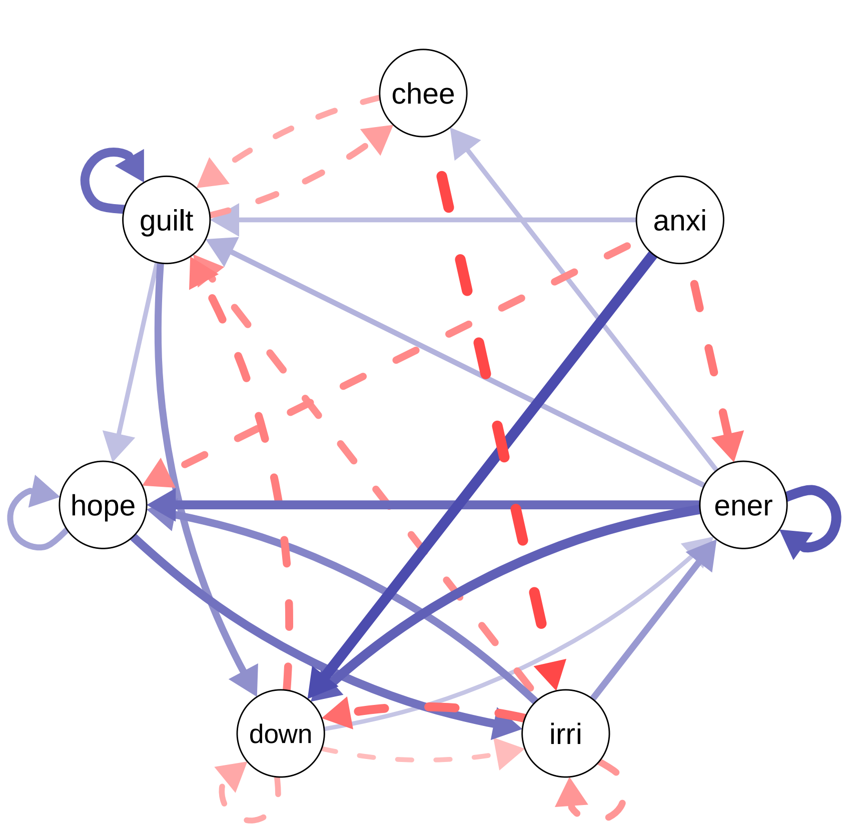

PCT plus ADM non−reg Pt 225 Estpoint 2

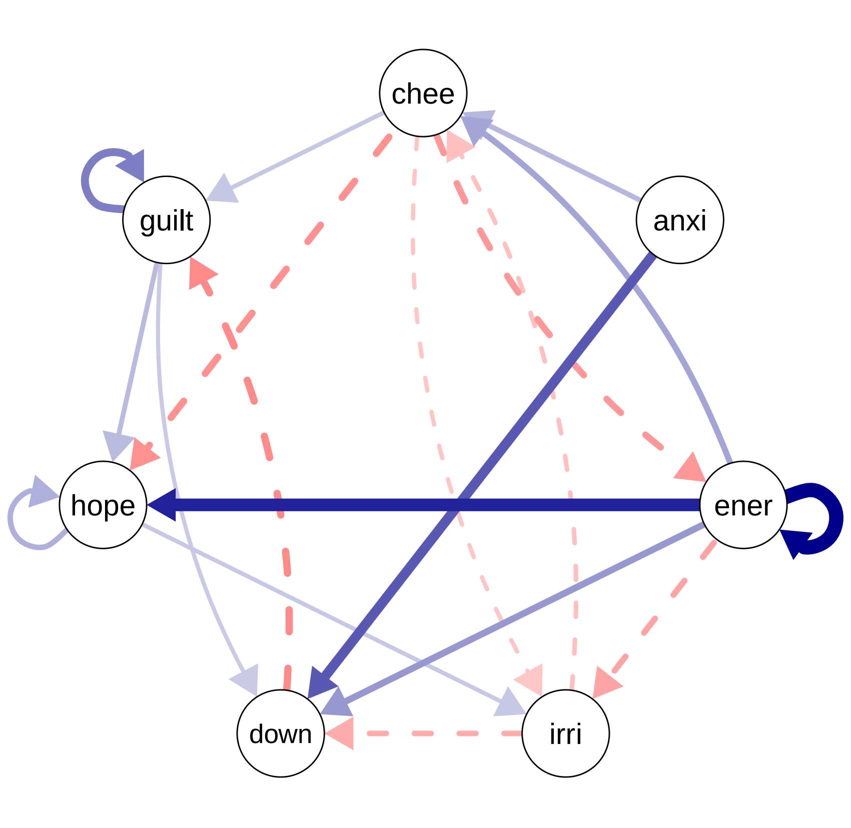

PCT plus ADM non−reg Pt 225 Estpoint 3

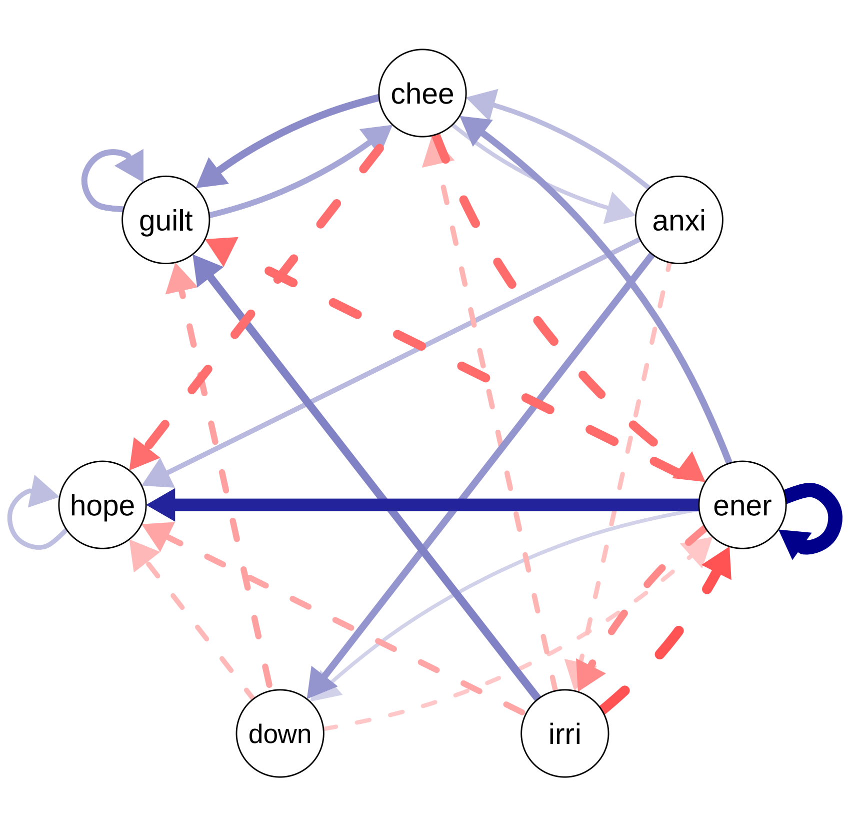

PCT plus ADM non−reg Pt 225 Estpoint 4

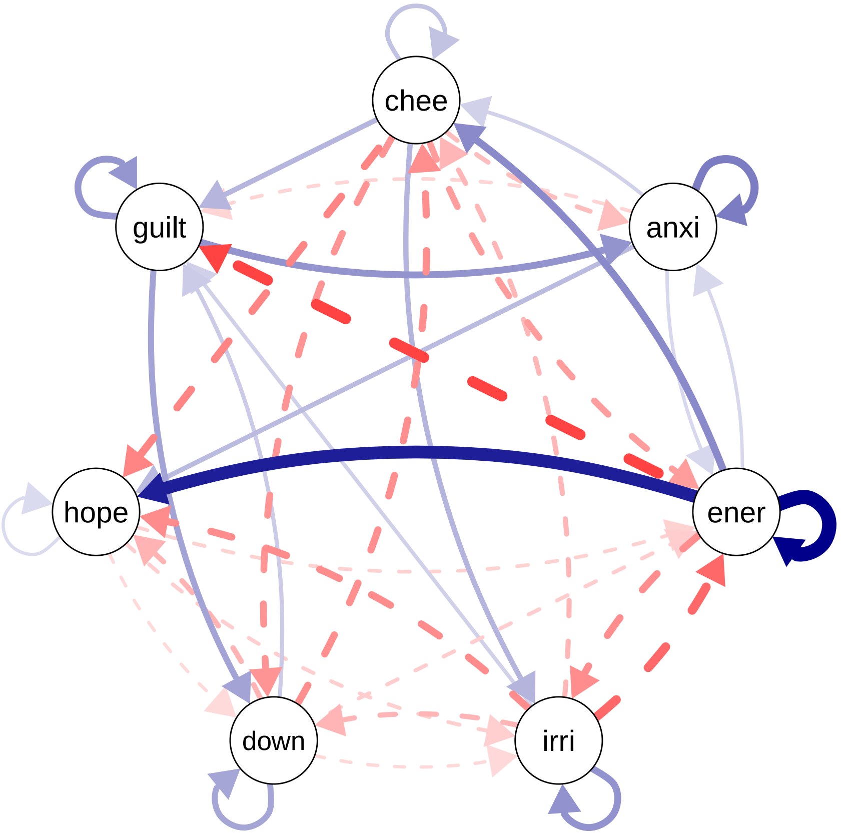

PCT plus ADM non−reg Pt 225 Estpoint 5

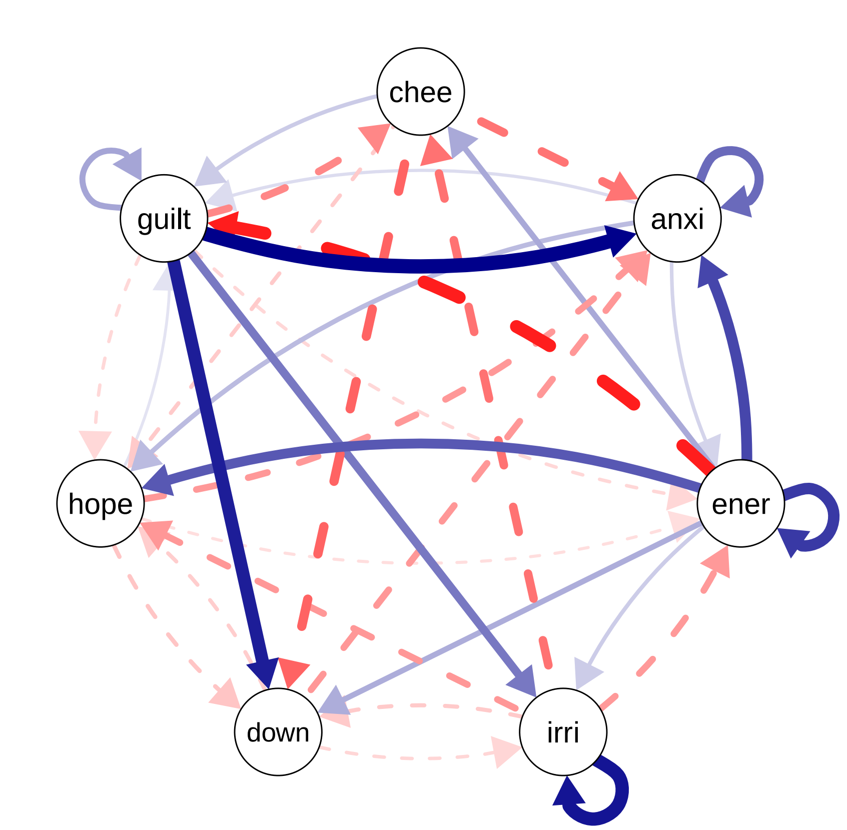

PCT plus ADM non−reg Pt 225 Estpoint 6

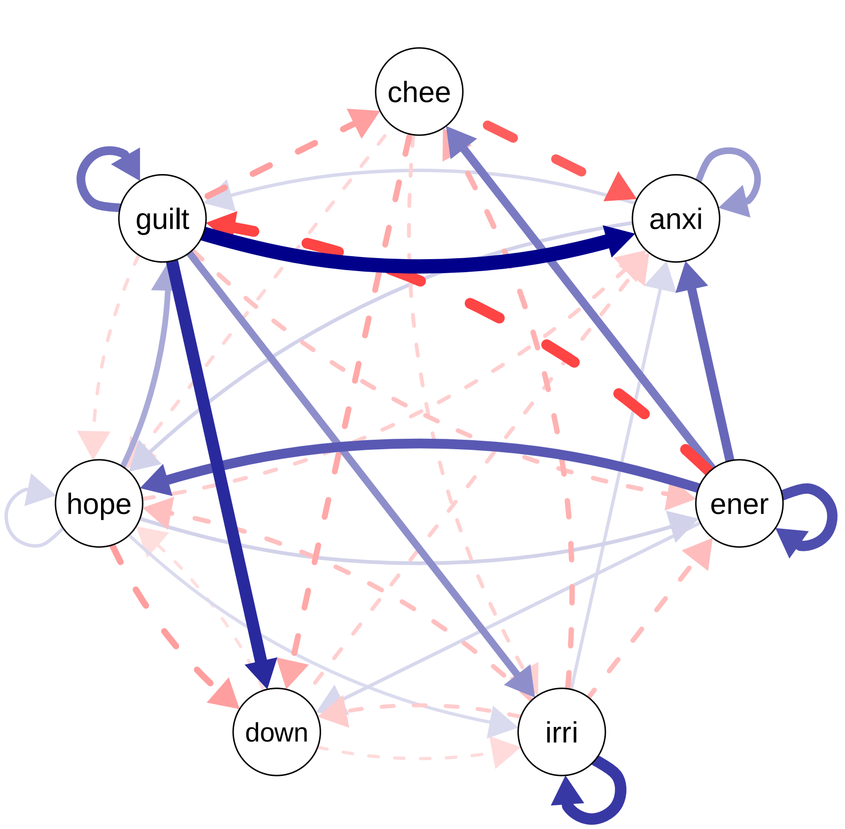

PCT plus ADM non−reg Pt 225 Estpoint 7

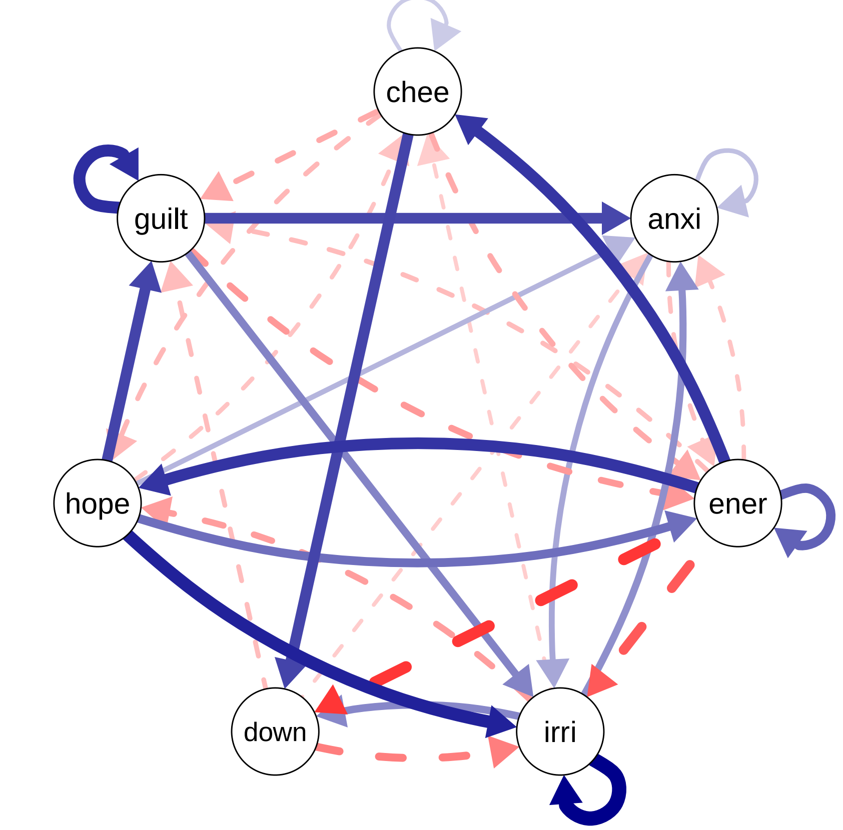

PCT plus ADM non−reg Pt 225 Estpoint 8

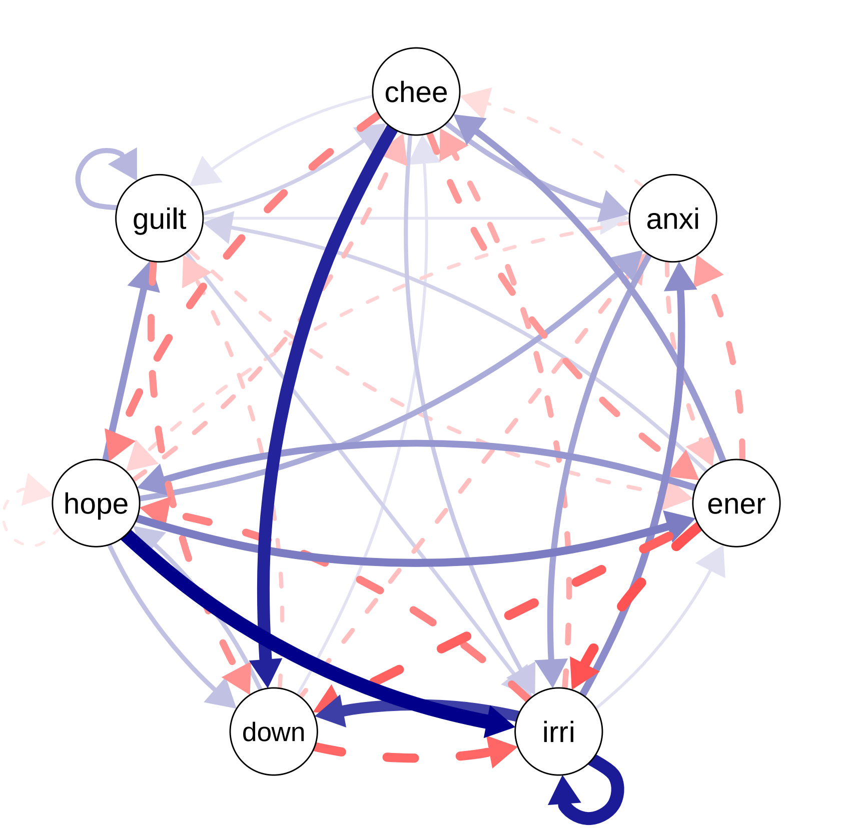

PCT plus ADM non−reg Pt 248 Estpoint 1

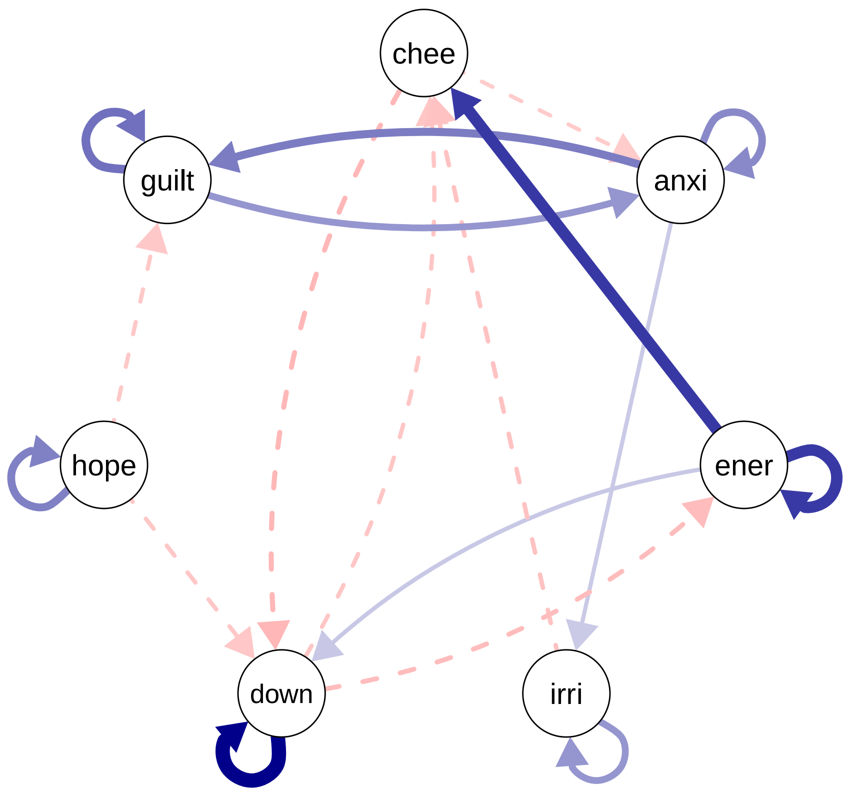

PCT plus ADM non−reg Pt 248 Estpoint 2

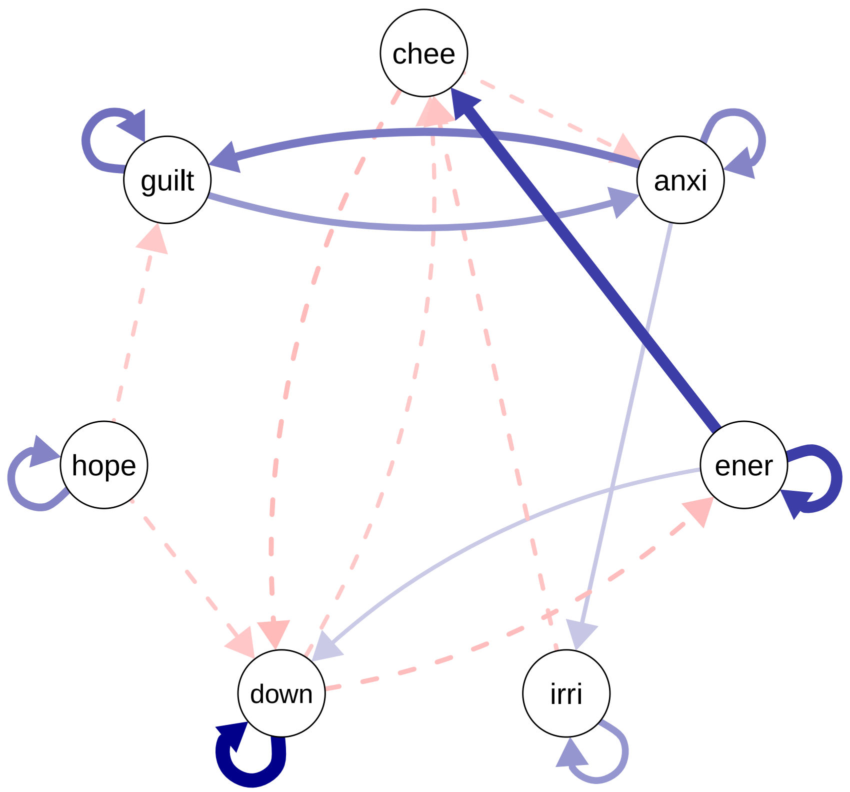

PCT plus ADM non−reg Pt 248 Estpoint 3

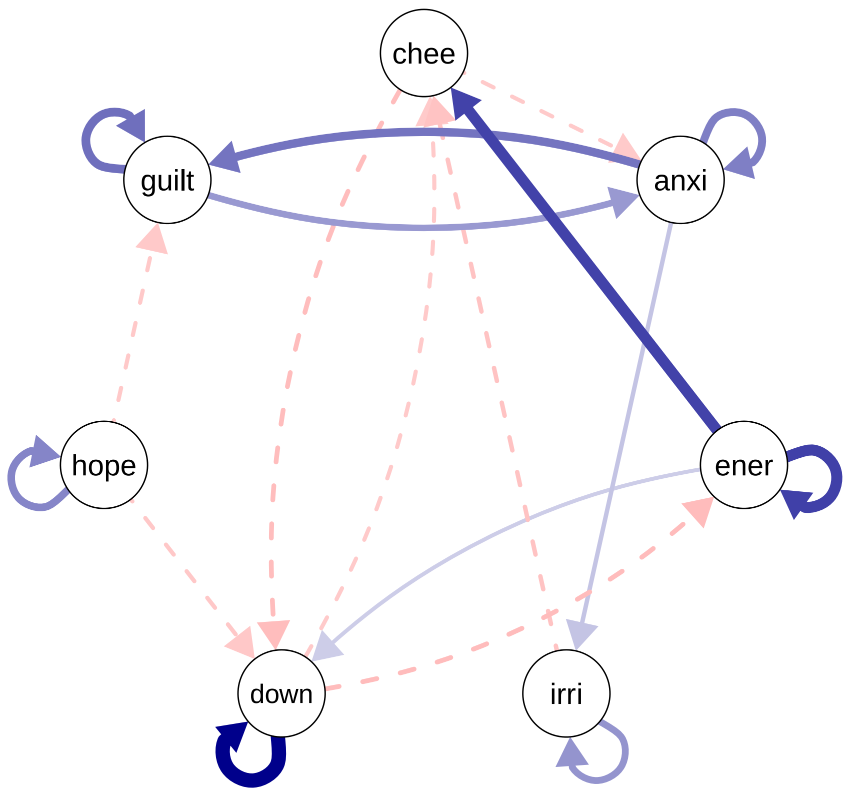

PCT plus ADM non−reg Pt 248 Estpoint 4

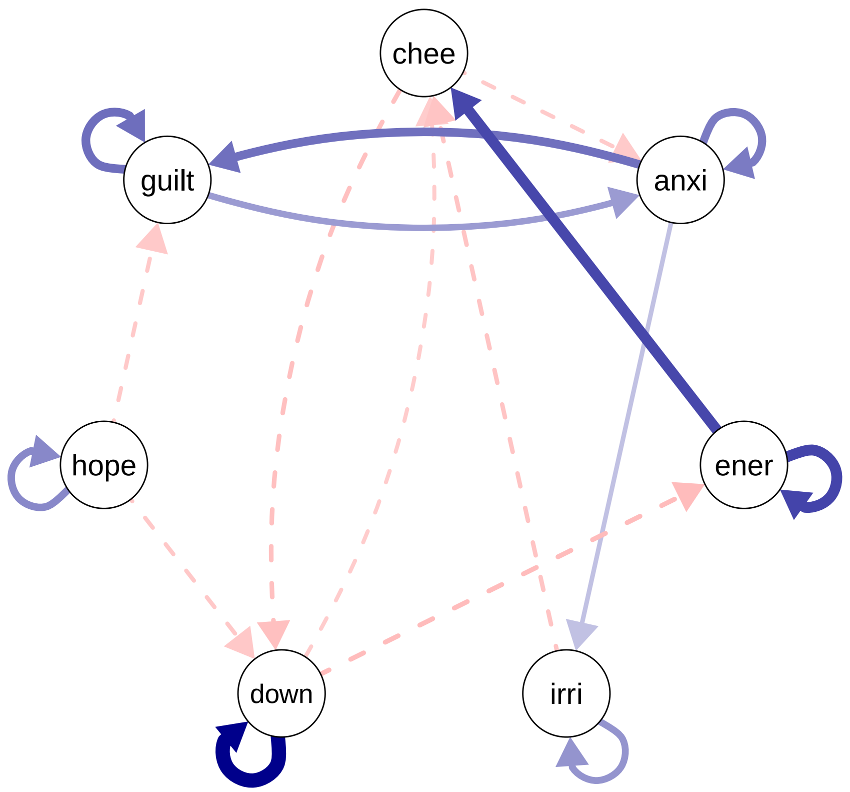

PCT plus ADM non−reg Pt 248 Estpoint 5

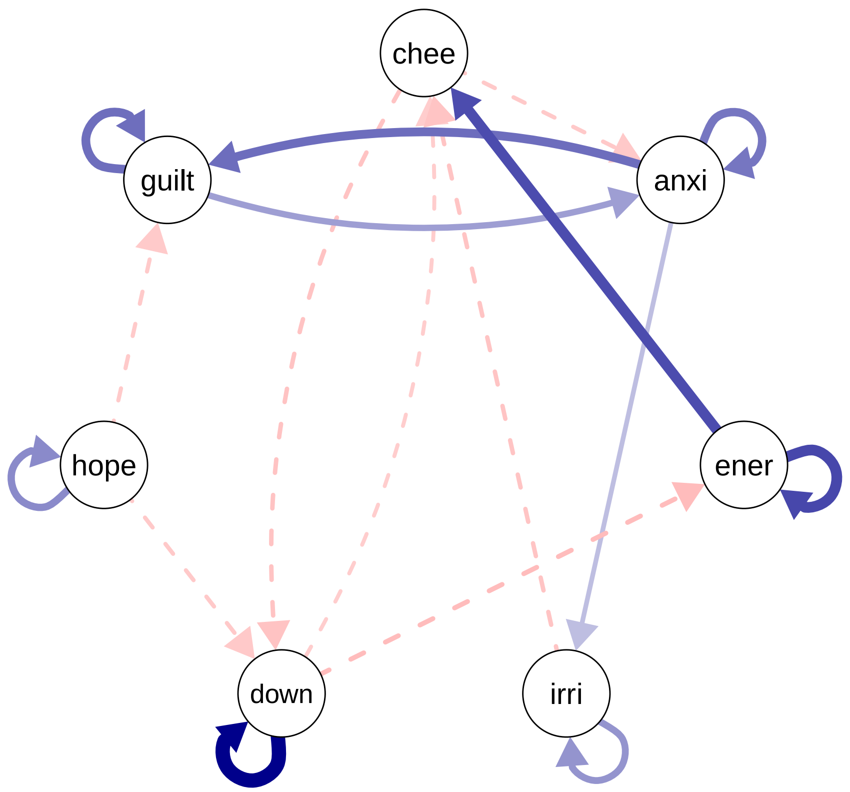

PCT plus ADM non−reg Pt 248 Estpoint 6

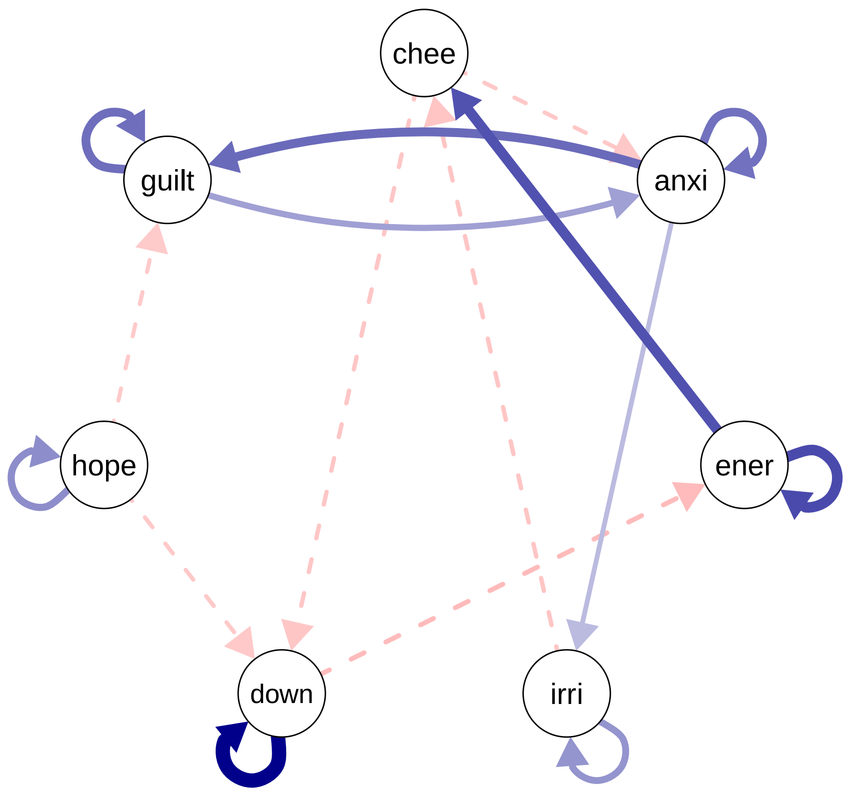

PCT plus ADM non−reg Pt 248 Estpoint 7

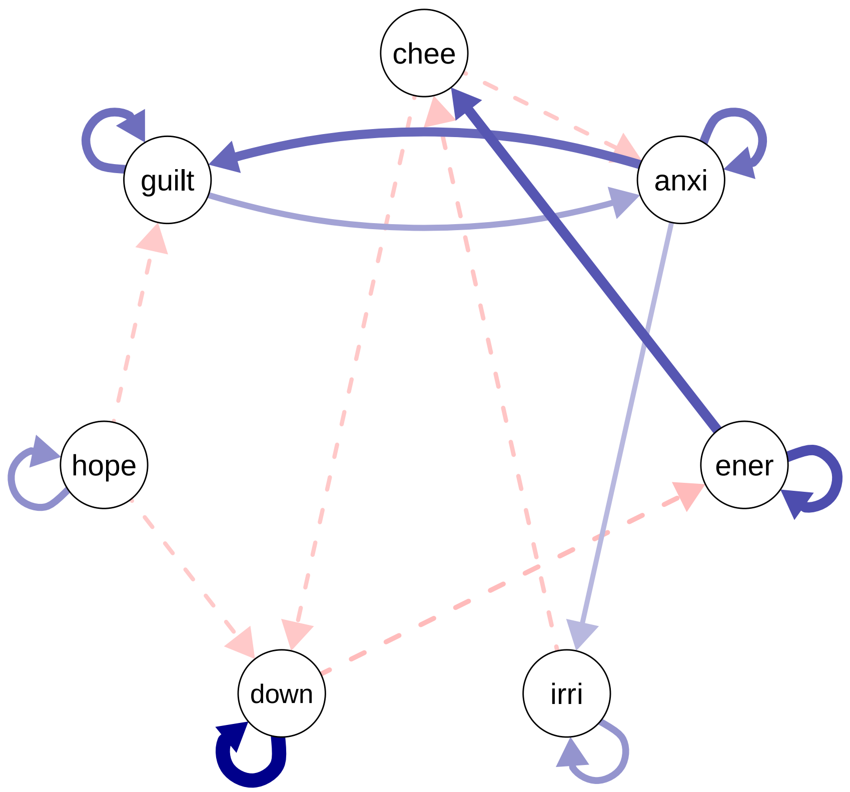

PCT plus ADM non−reg Pt 248 Estpoint 8

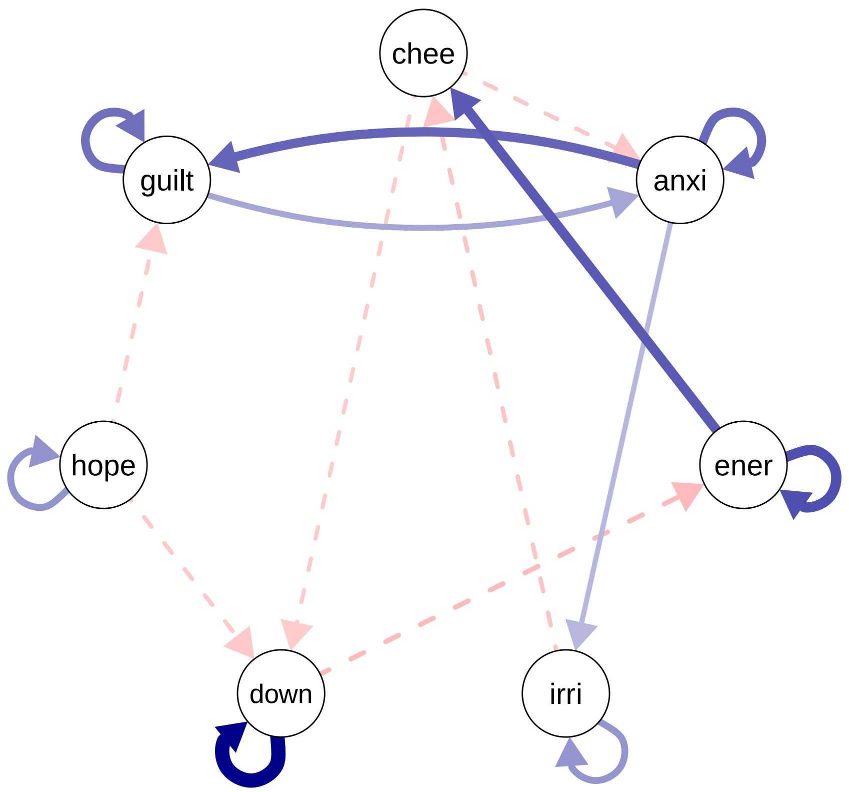

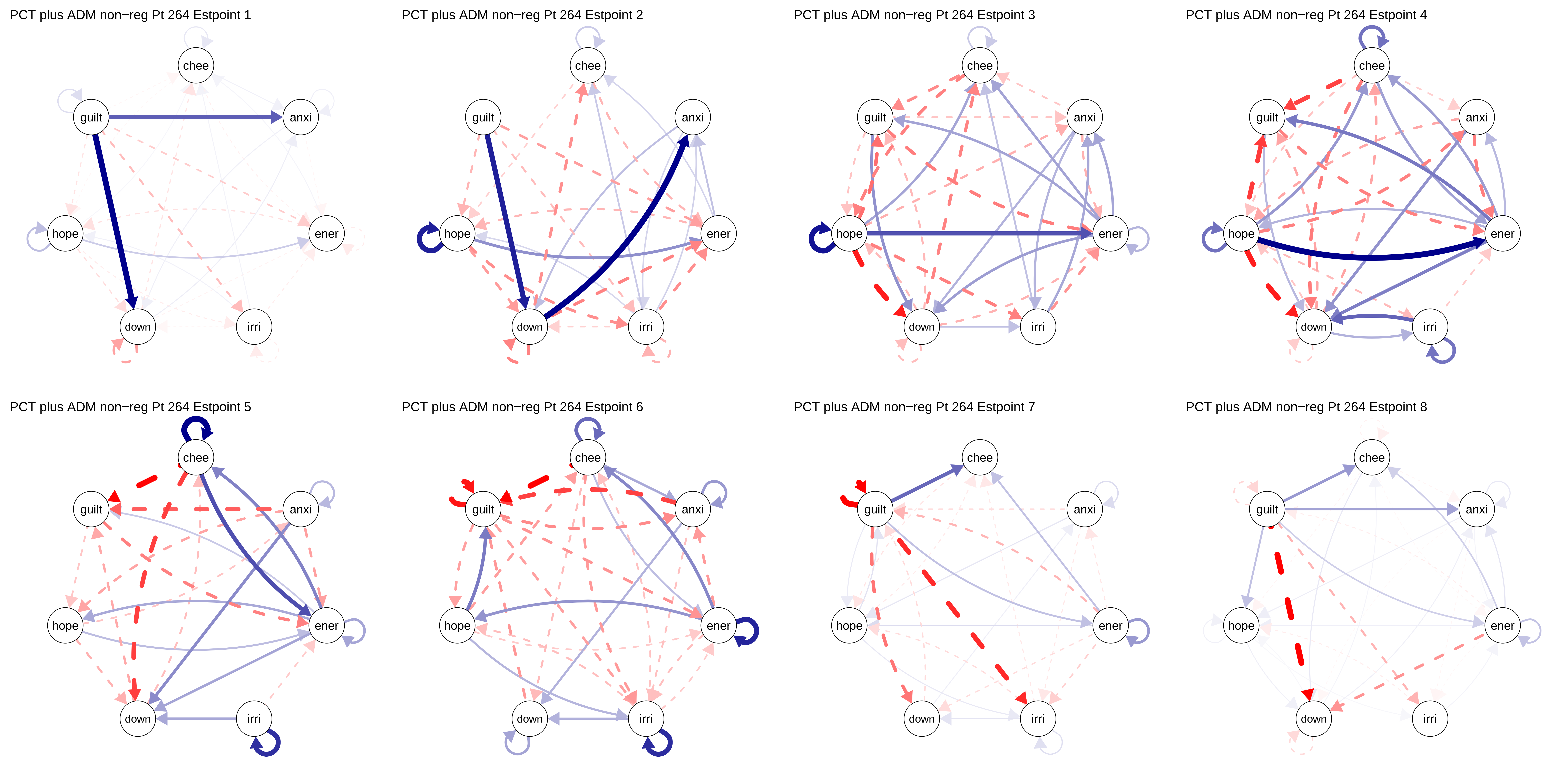
PCT plus ADM non−reg Pt 264 Estpoint 1
chee
guilt
anxi
hope
ener
down
irri
PCT plus ADM non−reg Pt 264 Estpoint 2
chee
guilt
anxi
hope
ener
down
irri
PCT plus ADM non−reg Pt 264 Estpoint 3
chee
guilt
anxi
hope
ener
down
irri
PCT plus ADM non−reg Pt 264 Estpoint 4
chee
guilt
anxi
hope
ener
down
irri
PCT plus ADM non−reg Pt 264 Estpoint 5
chee
guilt
anxi
hope
ener
down
irri
PCT plus ADM non−reg Pt 264 Estpoint 6
chee
guilt
anxi
hope
ener
down
irri
PCT plus ADM non−reg Pt 264 Estpoint 7
chee
guilt
anxi
hope
ener
down
irri
PCT plus ADM non−reg Pt 264 Estpoint 8
chee
guilt
anxi
hope
ener
down
irri

PCT plus ADM non−reg Pt 255 Estpoint 1

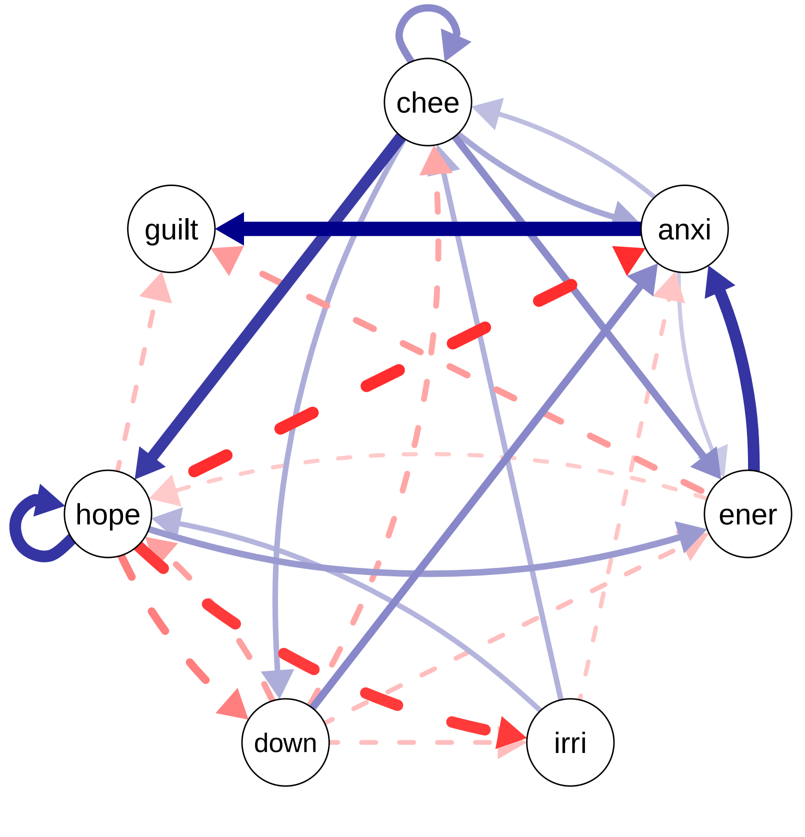

PCT plus ADM non−reg Pt 255 Estpoint 2

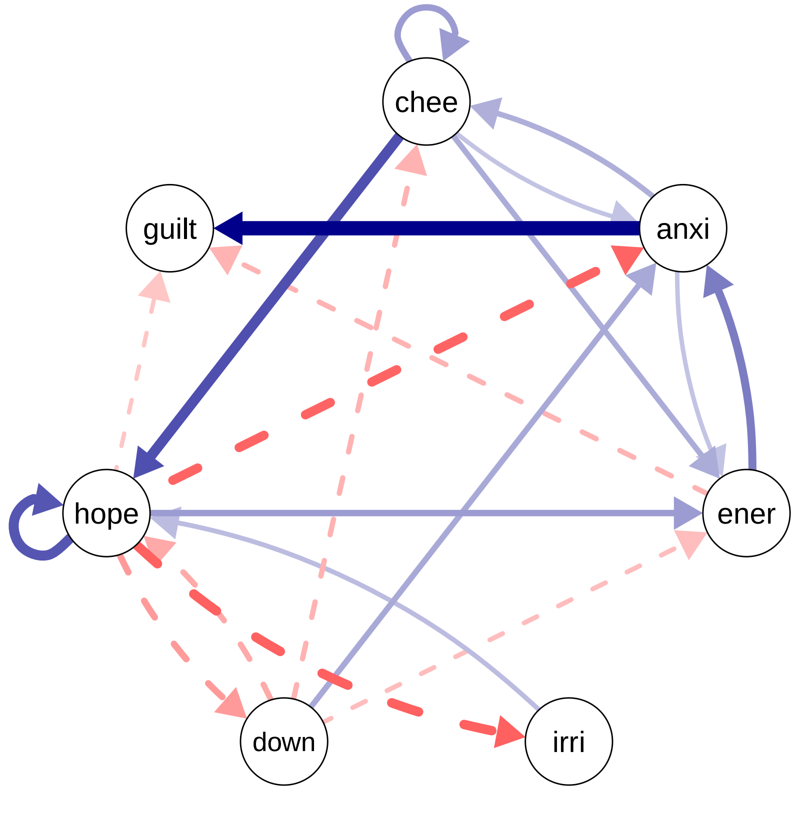

PCT plus ADM non−reg Pt 255 Estpoint 3

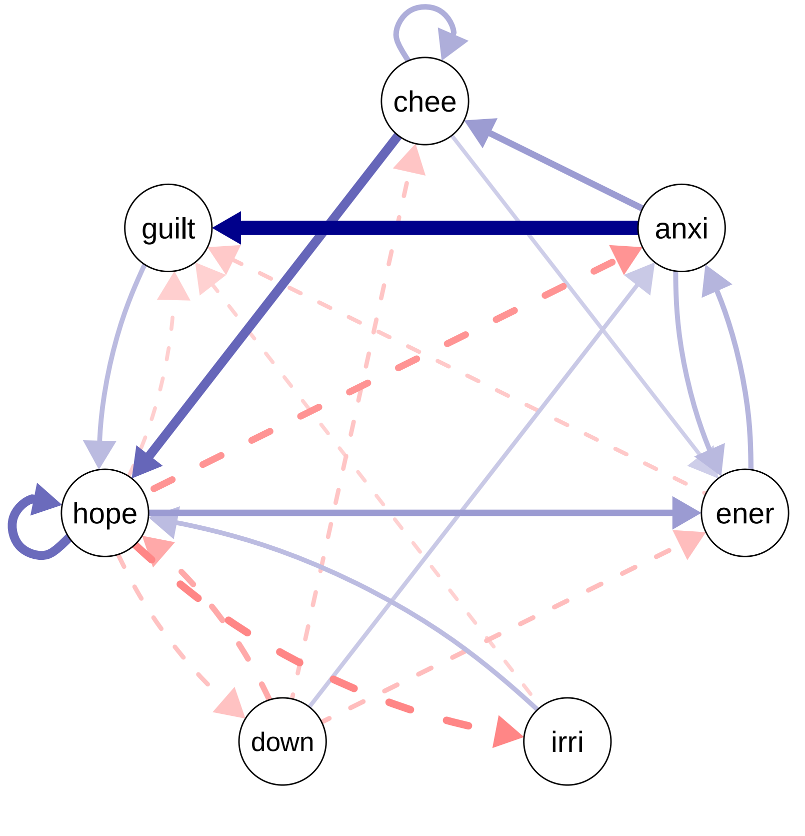

PCT plus ADM non−reg Pt 255 Estpoint 4

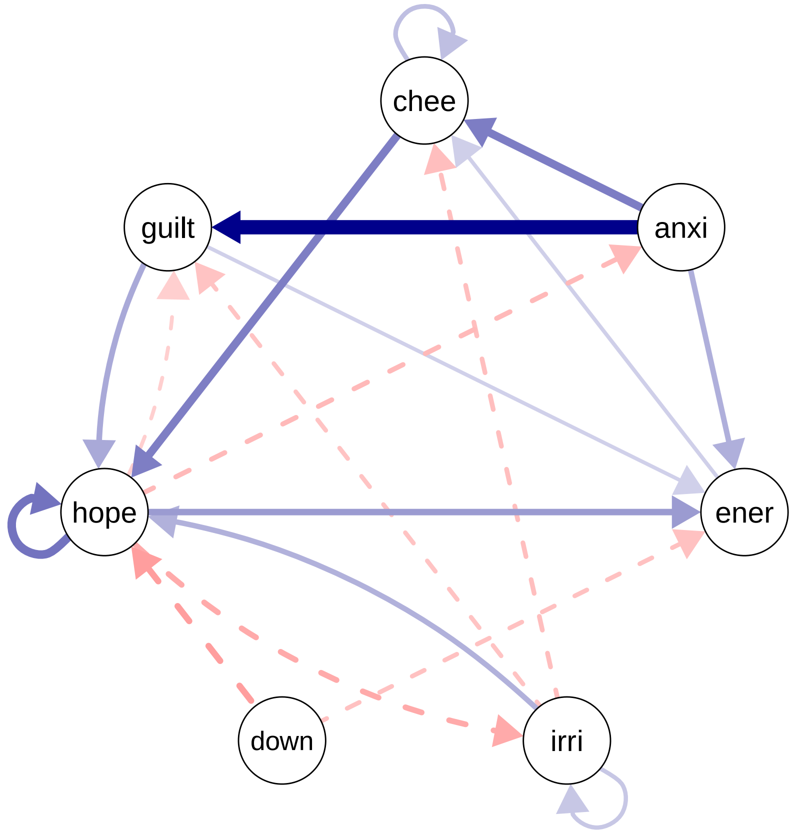

PCT plus ADM non−reg Pt 255 Estpoint 5

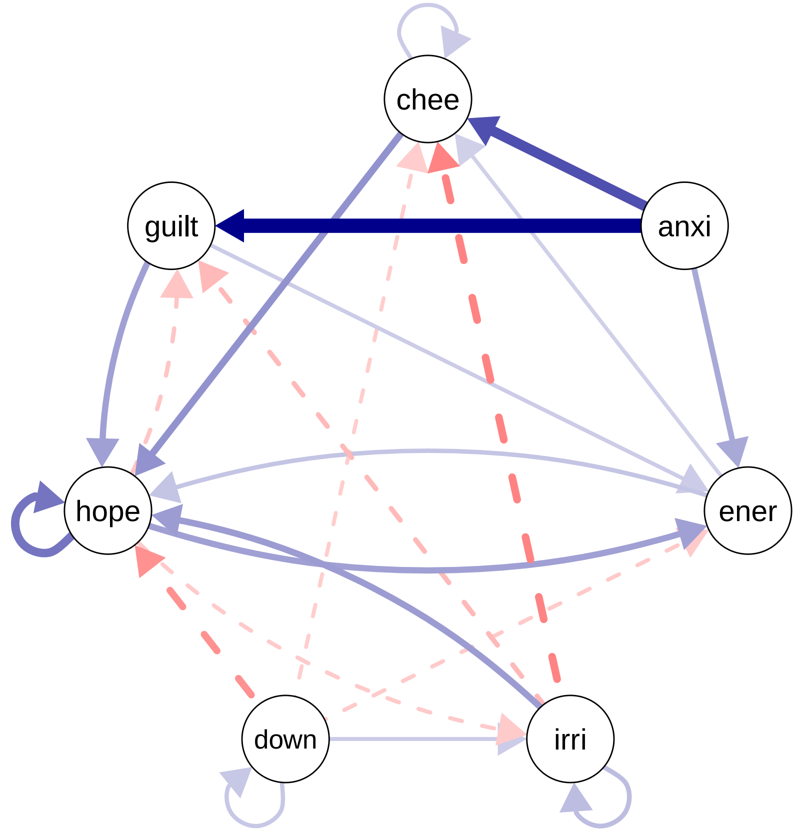

PCT plus ADM non−reg Pt 255 Estpoint 6

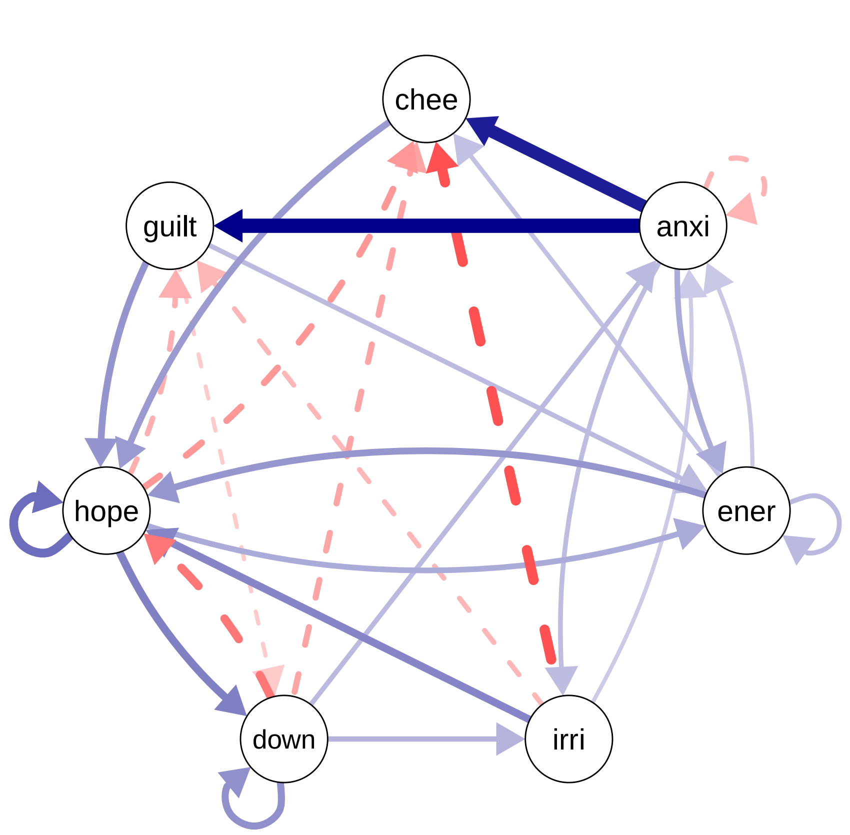

PCT plus ADM non−reg Pt 255 Estpoint 7

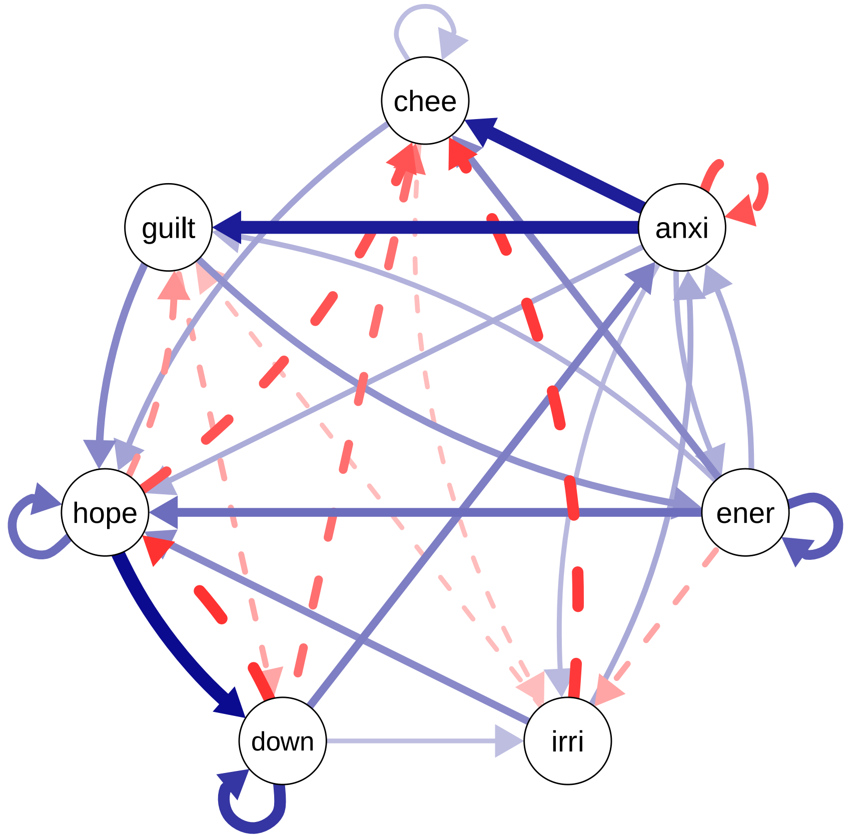

PCT plus ADM non−reg Pt 255 Estpoint 8

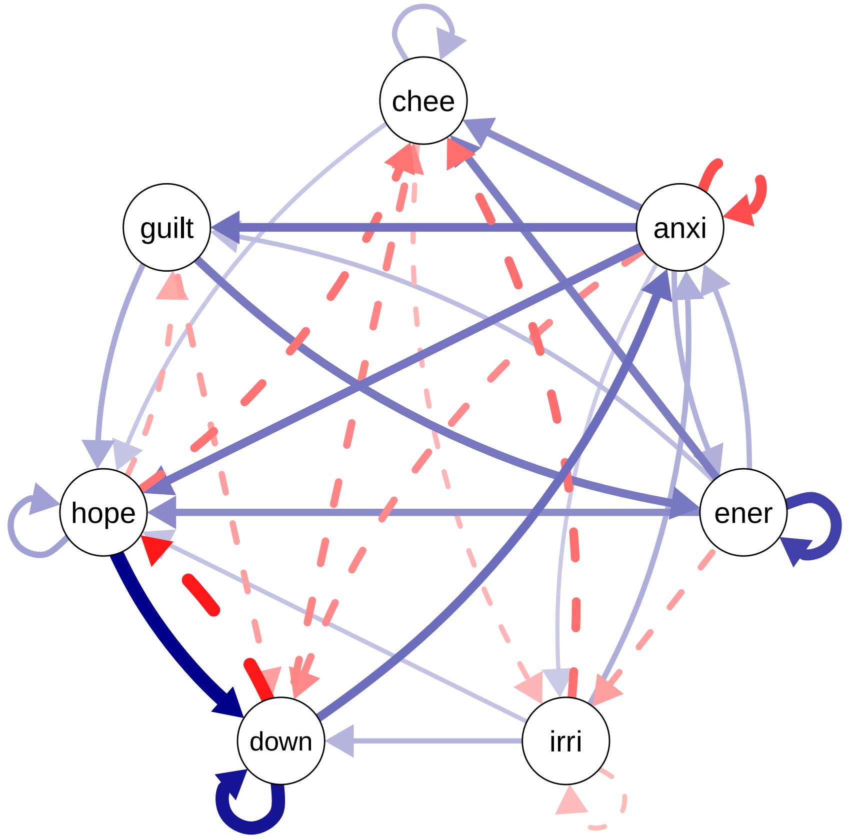

PCT plus ADM non−reg Pt 261 Estpoint 1

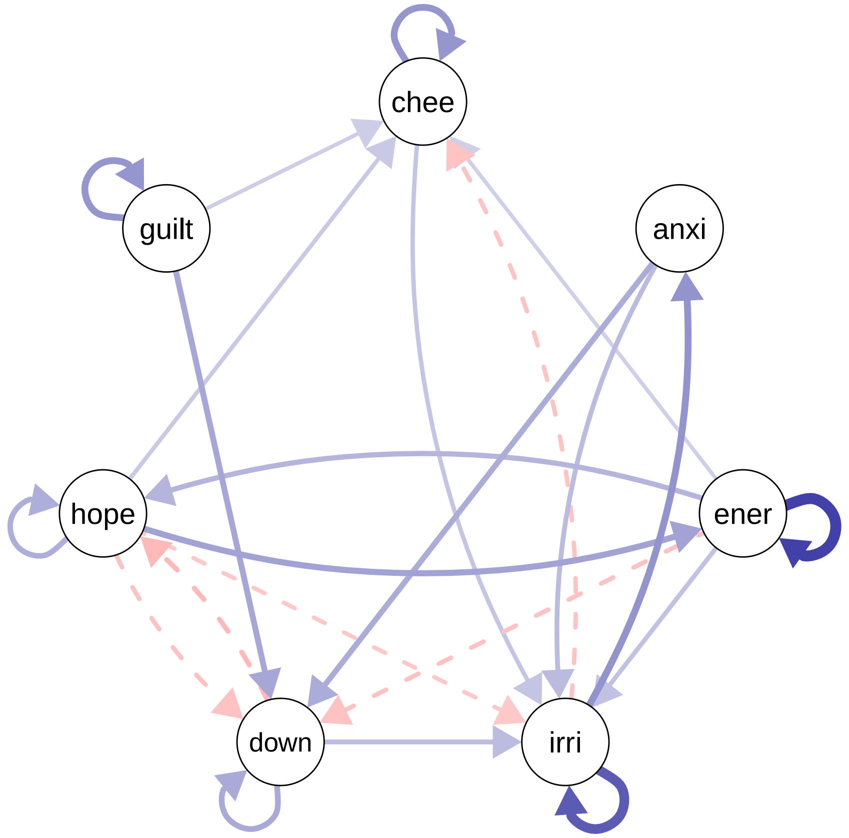

PCT plus ADM non−reg Pt 261 Estpoint 2

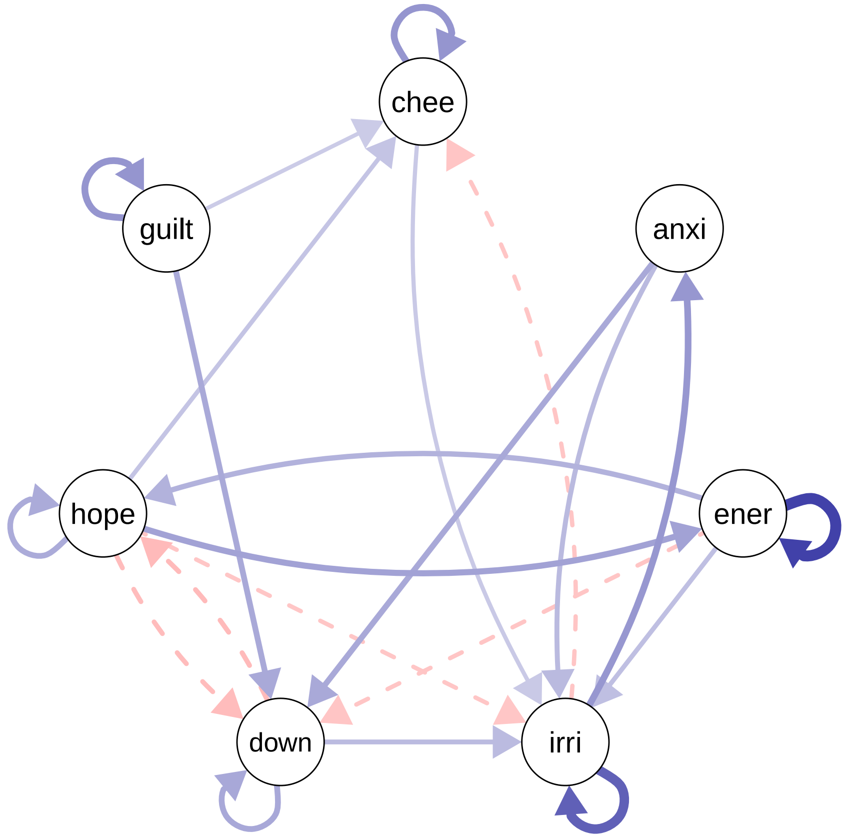

PCT plus ADM non−reg Pt 261 Estpoint 3

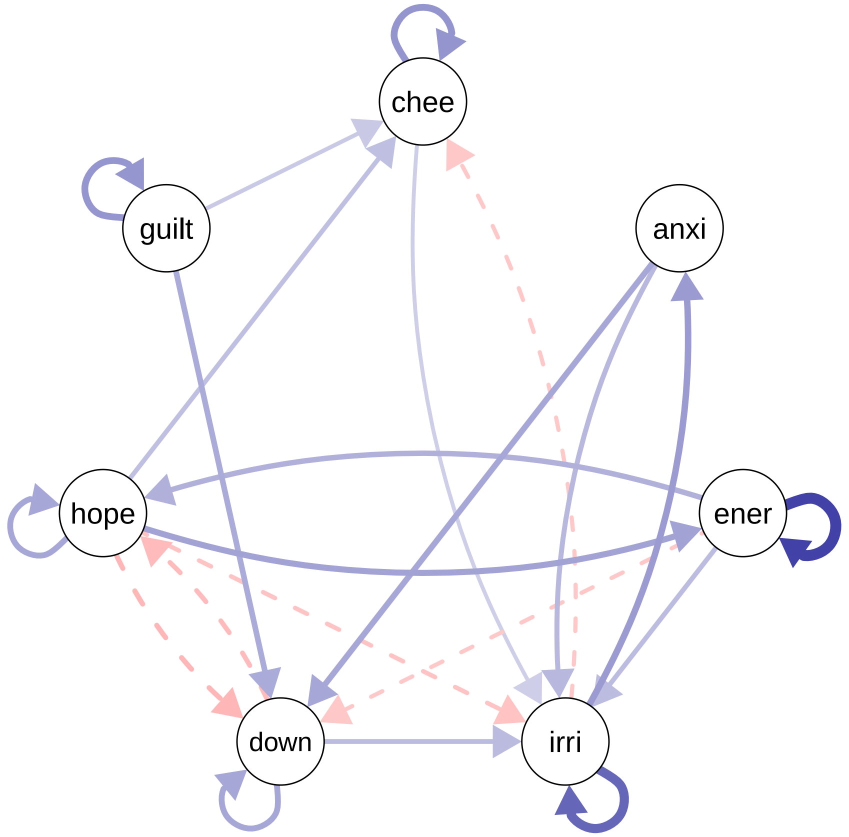

PCT plus ADM non−reg Pt 261 Estpoint 4

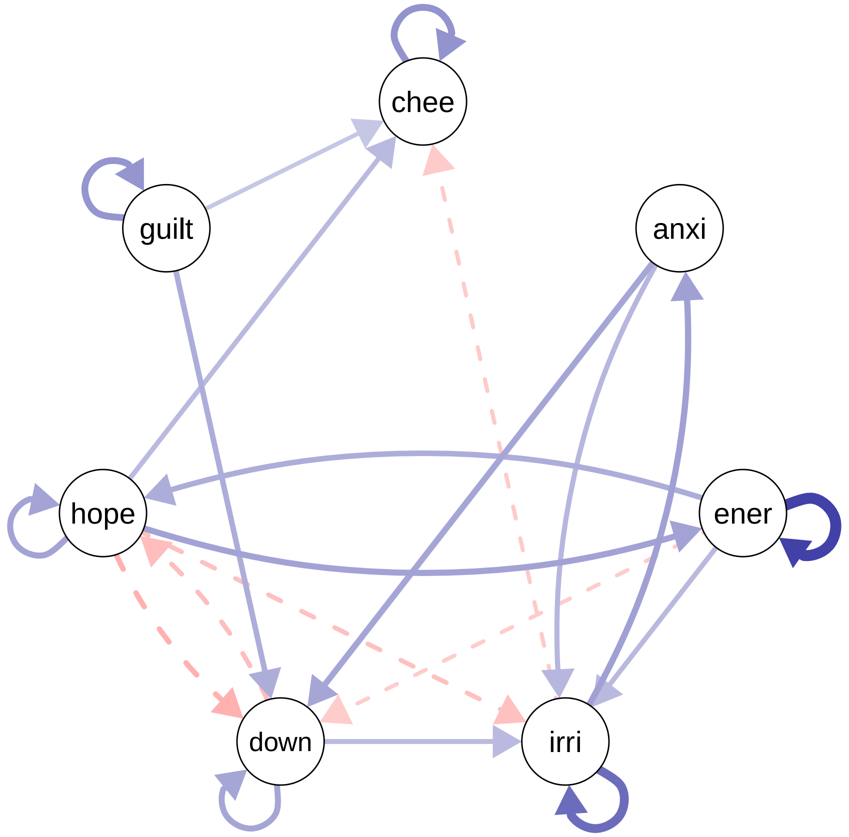

PCT plus ADM non−reg Pt 261 Estpoint 5

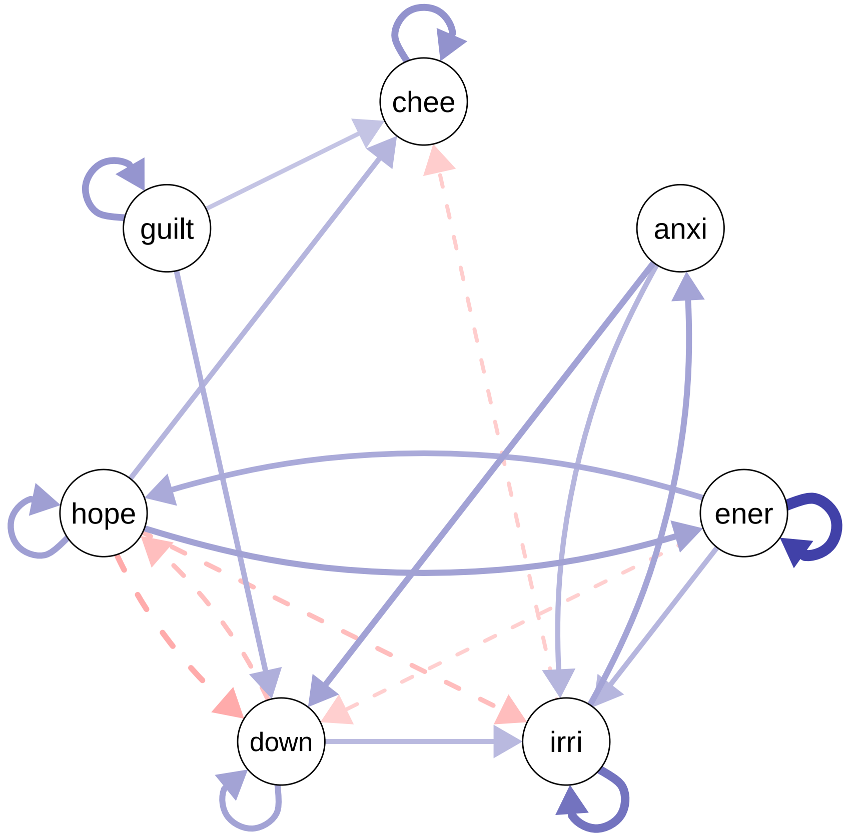

PCT plus ADM non−reg Pt 261 Estpoint 6

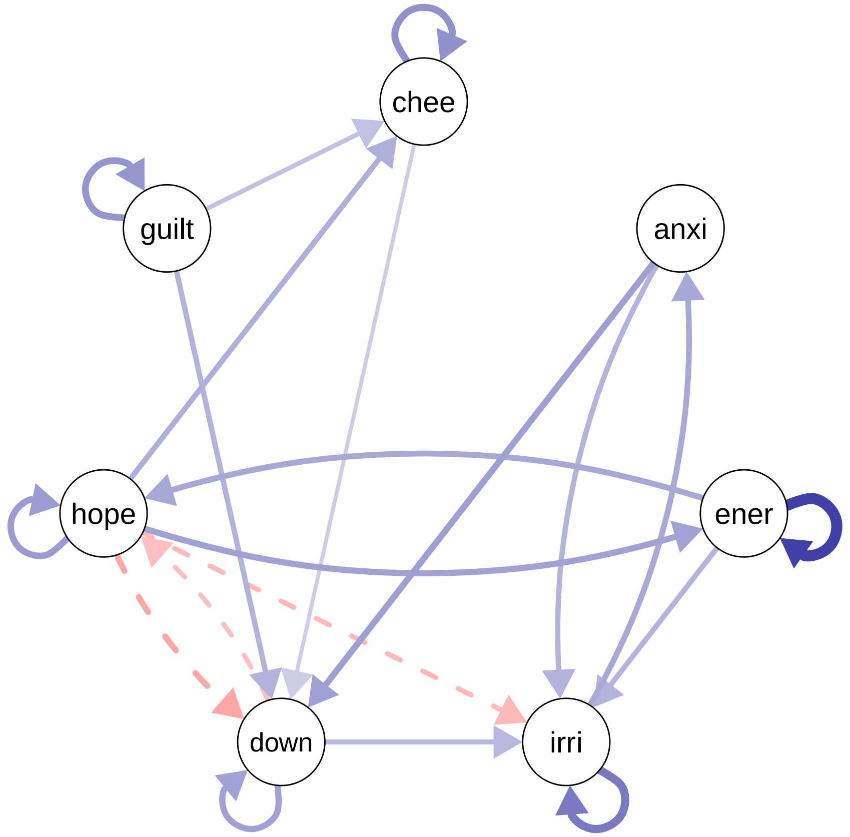

PCT plus ADM non−reg Pt 261 Estpoint 7

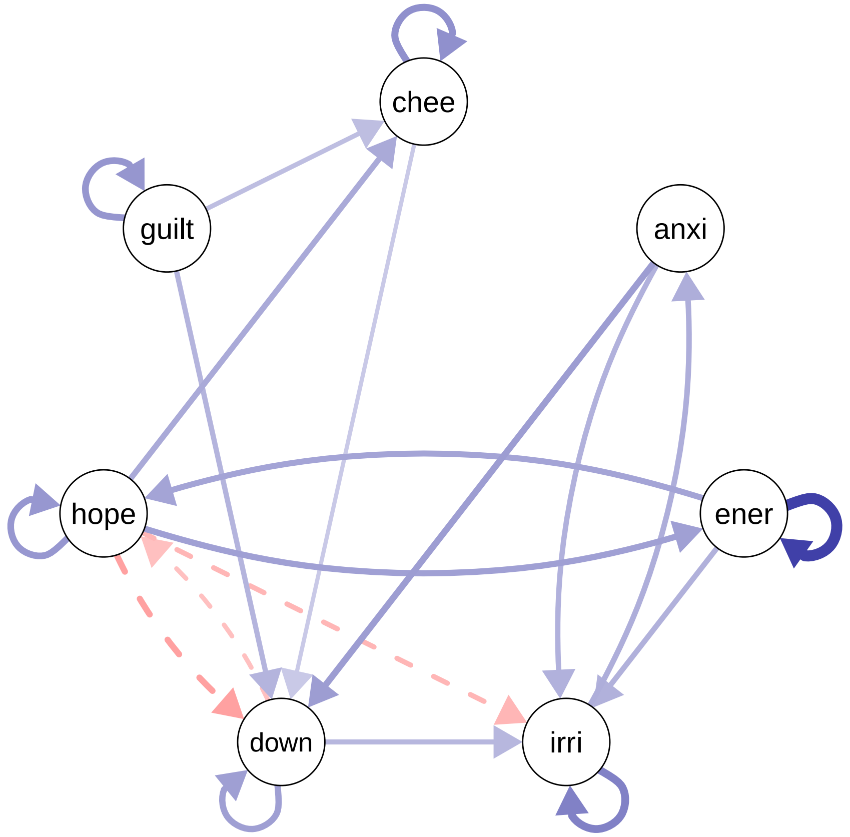

PCT plus ADM non−reg Pt 261 Estpoint 8

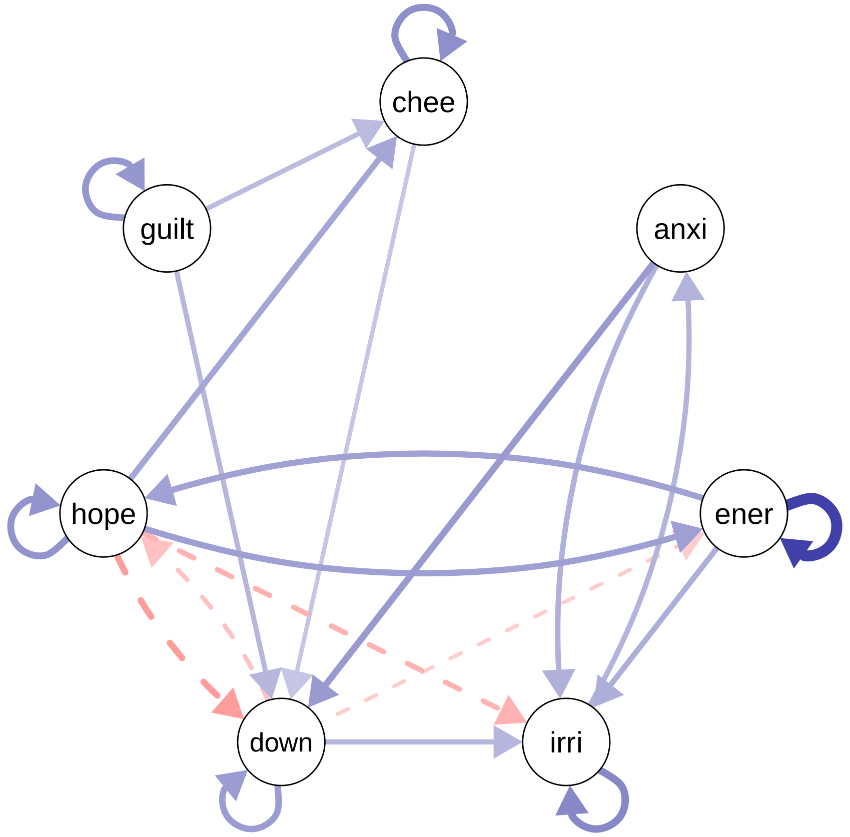

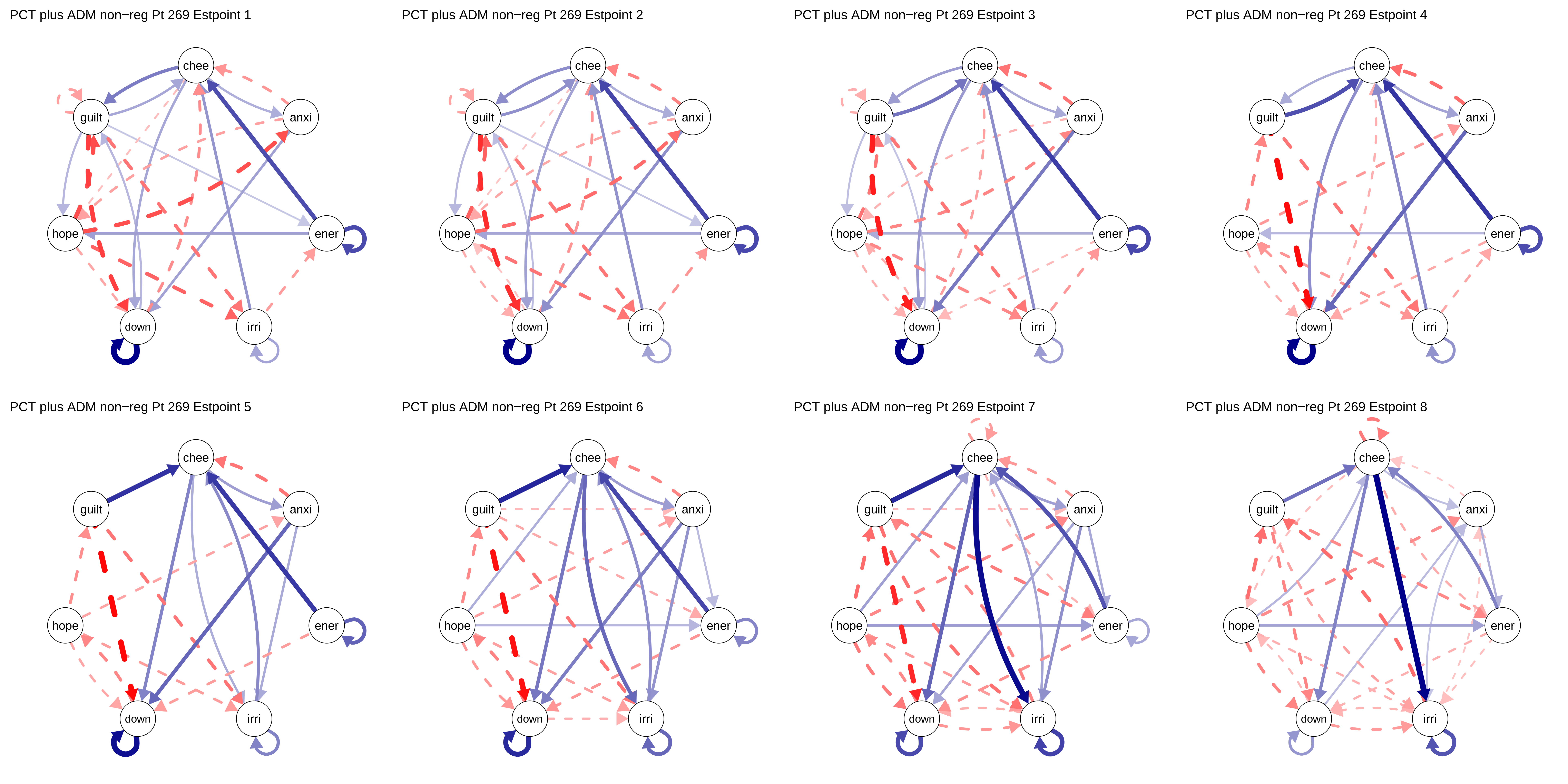
PCT plus ADM non−reg Pt 269 Estpoint 1
chee
guilt
anxi
hope
ener
down
irri
PCT plus ADM non−reg Pt 269 Estpoint 2
chee
guilt
anxi
hope
ener
down
irri
PCT plus ADM non−reg Pt 269 Estpoint 3
chee
guilt
anxi
hope
ener
down
irri
PCT plus ADM non−reg Pt 269 Estpoint 4
chee
guilt
anxi
hope
ener
down
irri
PCT plus ADM non−reg Pt 269 Estpoint 5
chee
guilt
anxi
hope
ener
down
irri
PCT plus ADM non−reg Pt 269 Estpoint 6
chee
guilt
anxi
hope
ener
down
irri
PCT plus ADM non−reg Pt 269 Estpoint 7
chee
guilt
anxi
hope
ener
down
irri
PCT plus ADM non−reg Pt 269 Estpoint 8
chee
guilt
anxi
hope
ener
down
irri

PCT plus ADM non−reg Pt 258 Estpoint 1

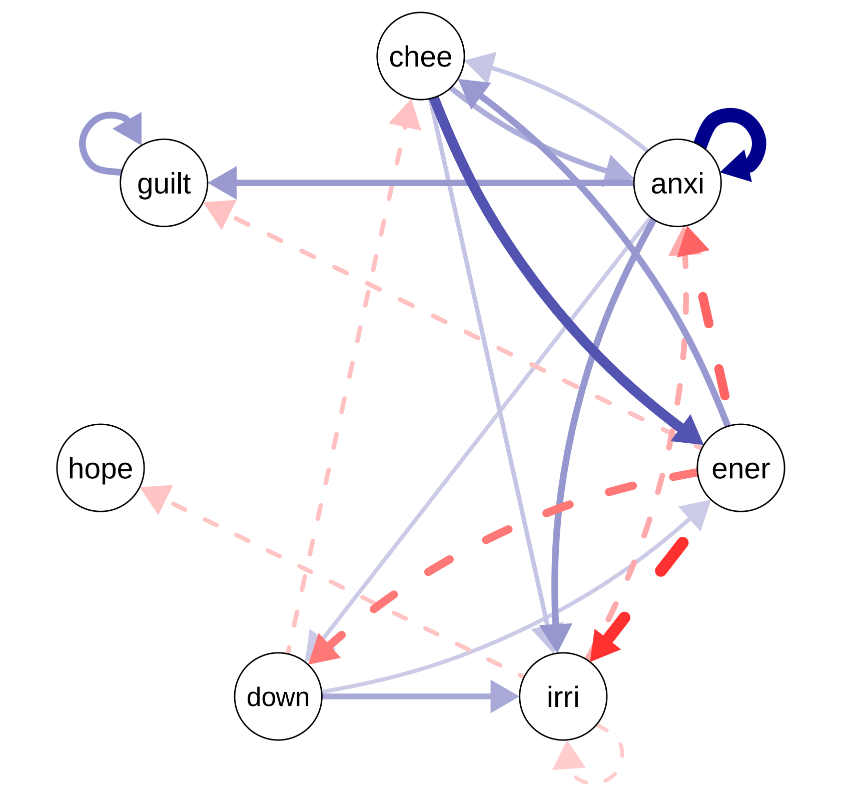

PCT plus ADM non−reg Pt 258 Estpoint 2

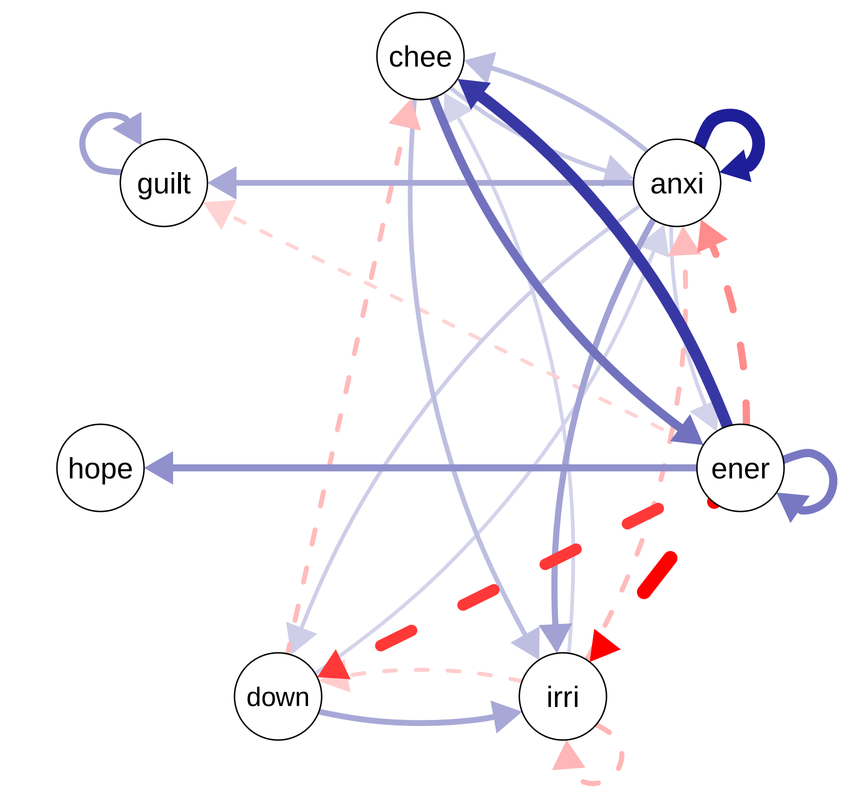

PCT plus ADM non−reg Pt 258 Estpoint 3

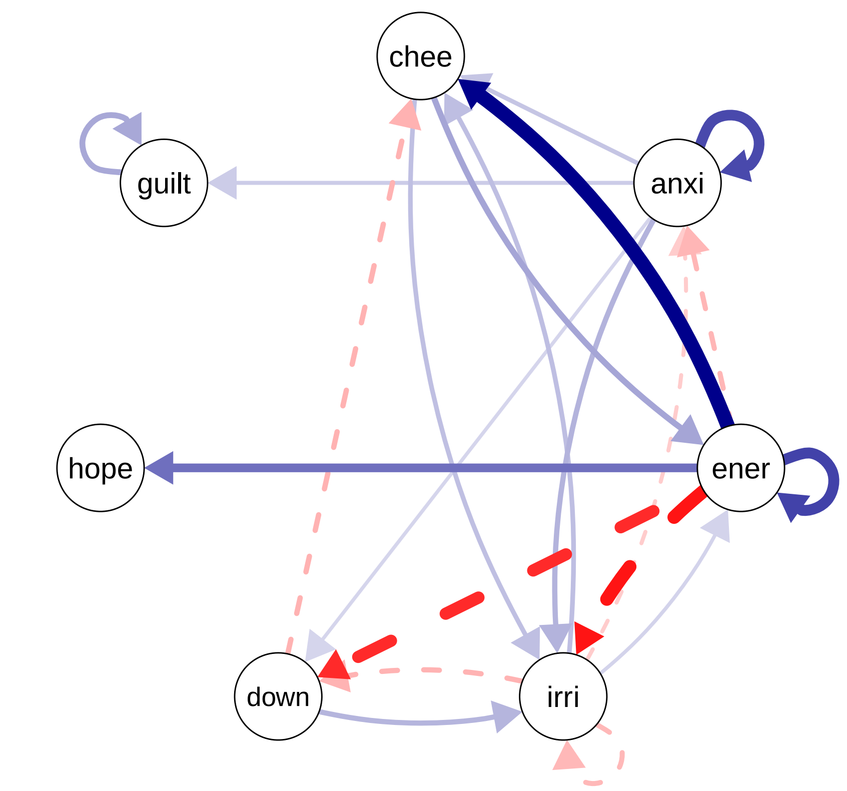

PCT plus ADM non−reg Pt 258 Estpoint 4

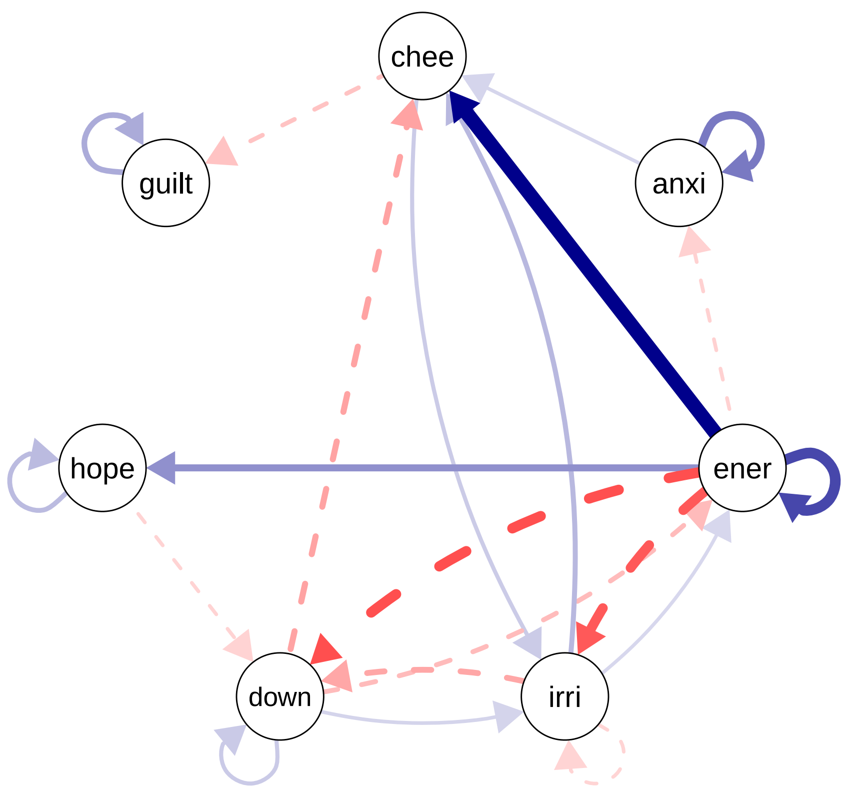

PCT plus ADM non−reg Pt 258 Estpoint 5

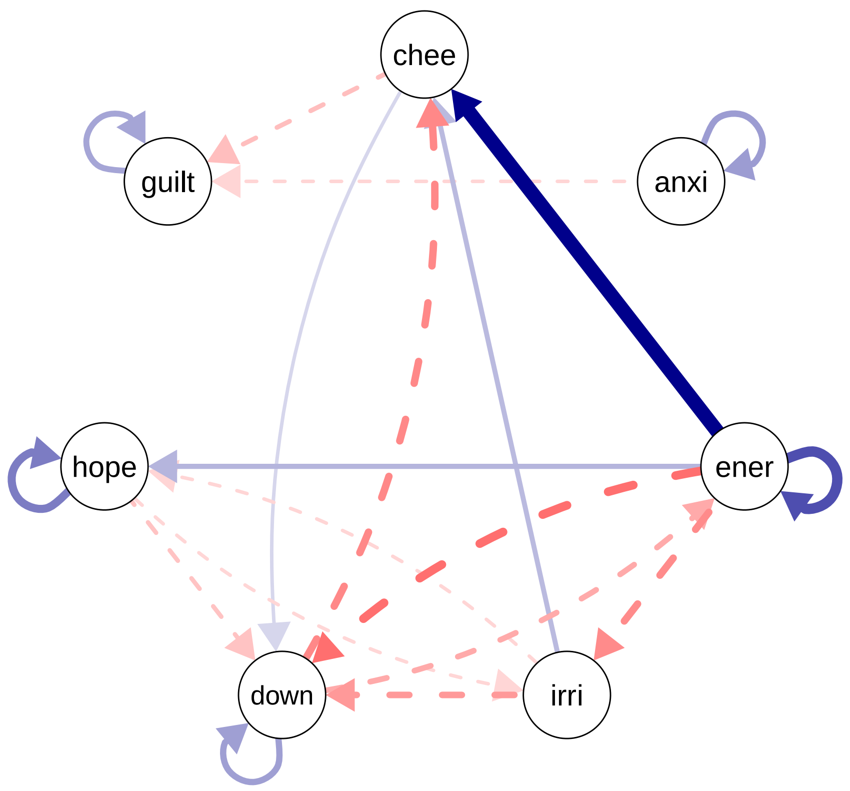

PCT plus ADM non−reg Pt 258 Estpoint 6

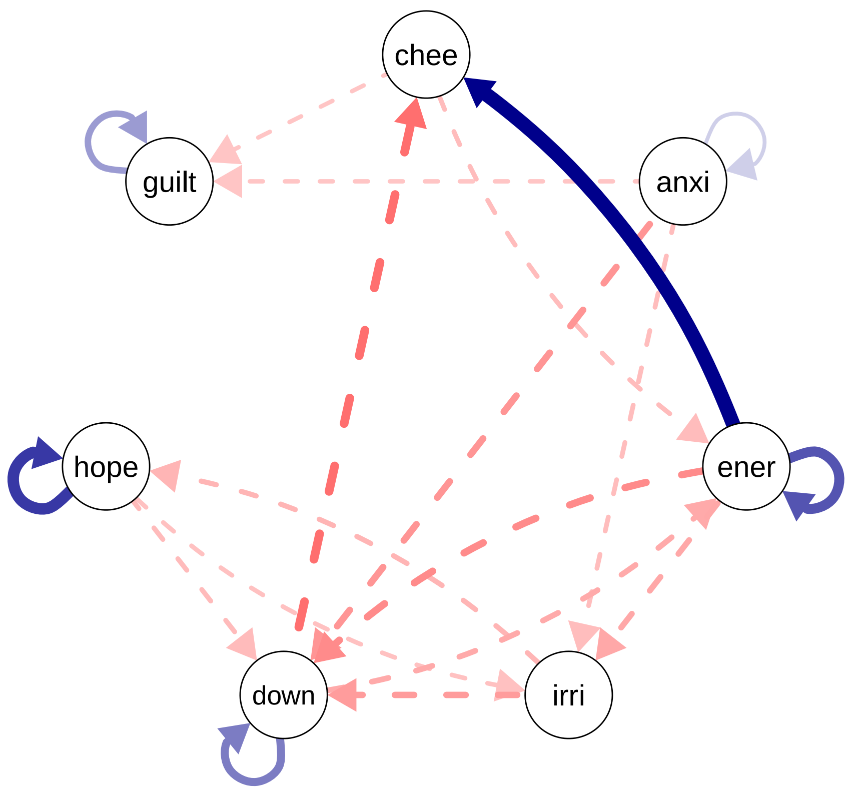

PCT plus ADM non−reg Pt 258 Estpoint 7

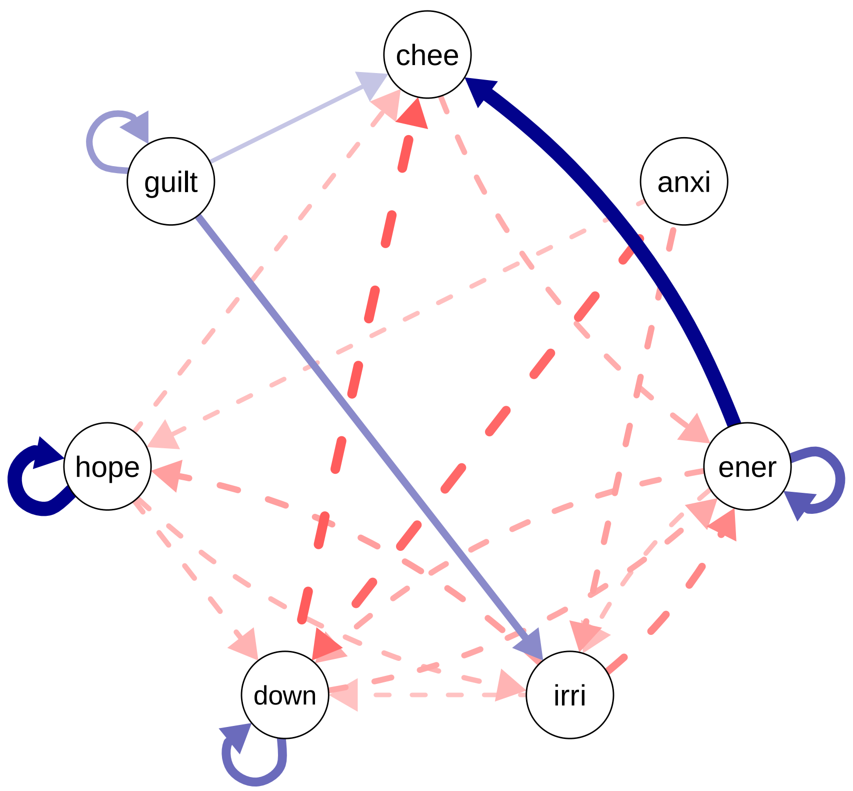

PCT plus ADM non−reg Pt 258 Estpoint 8

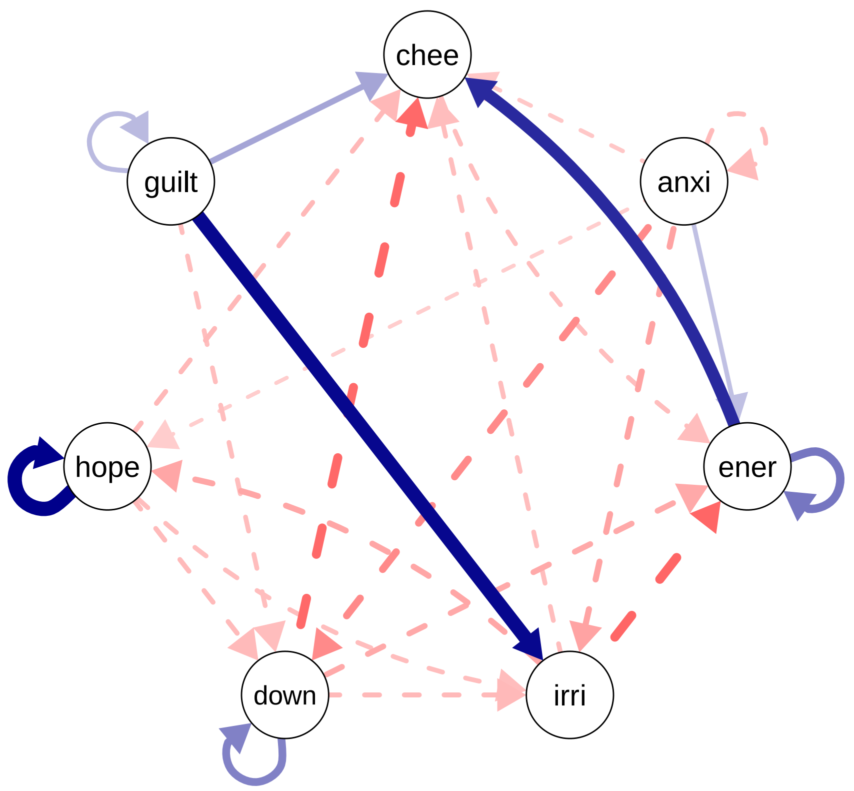

PCT plus ADM non−reg Pt 290 Estpoint 1

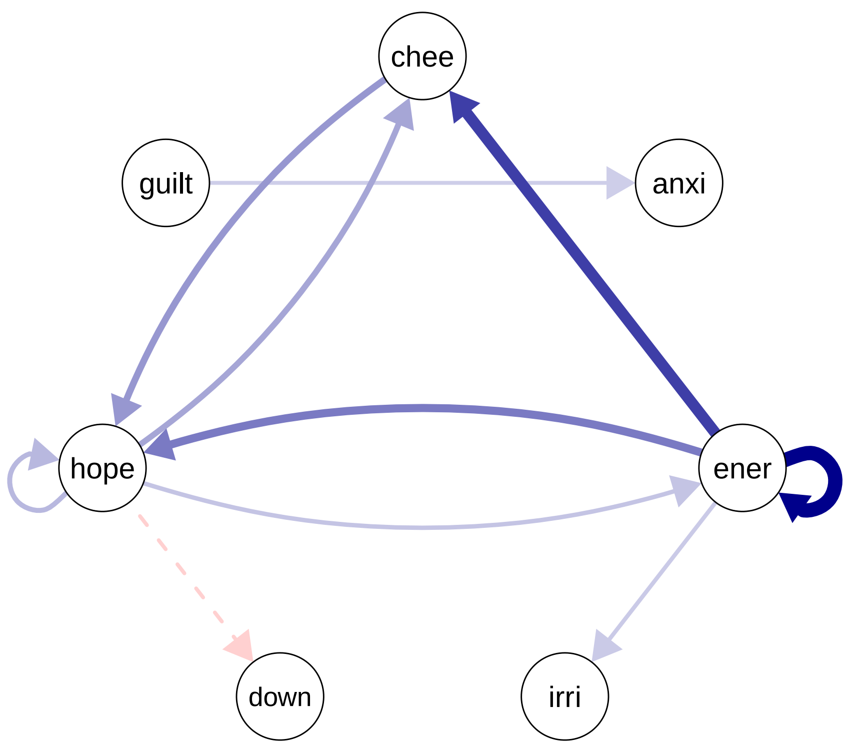

PCT plus ADM non−reg Pt 290 Estpoint 2

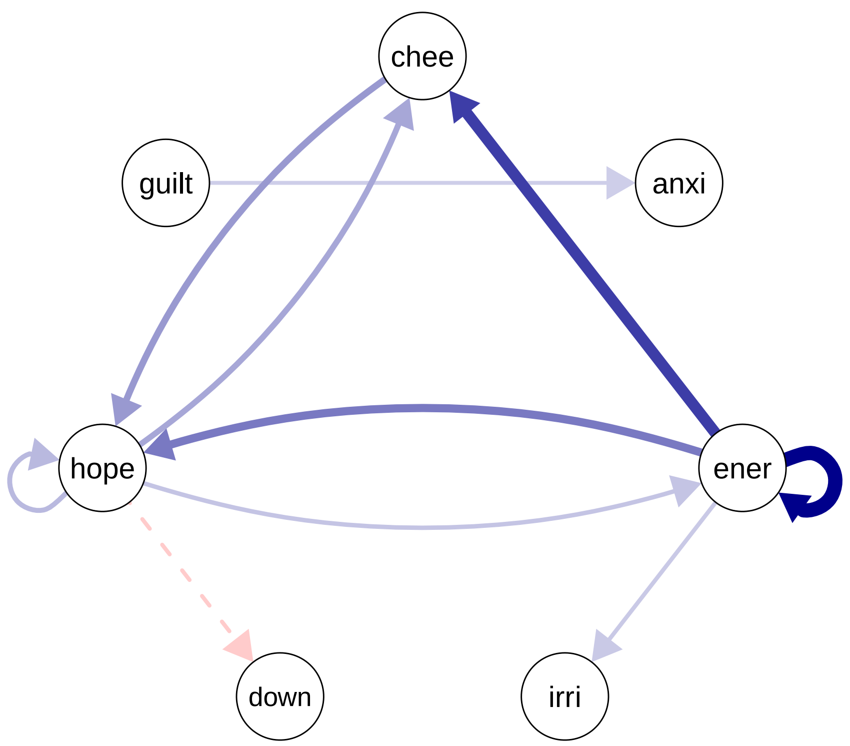

PCT plus ADM non−reg Pt 290 Estpoint 3

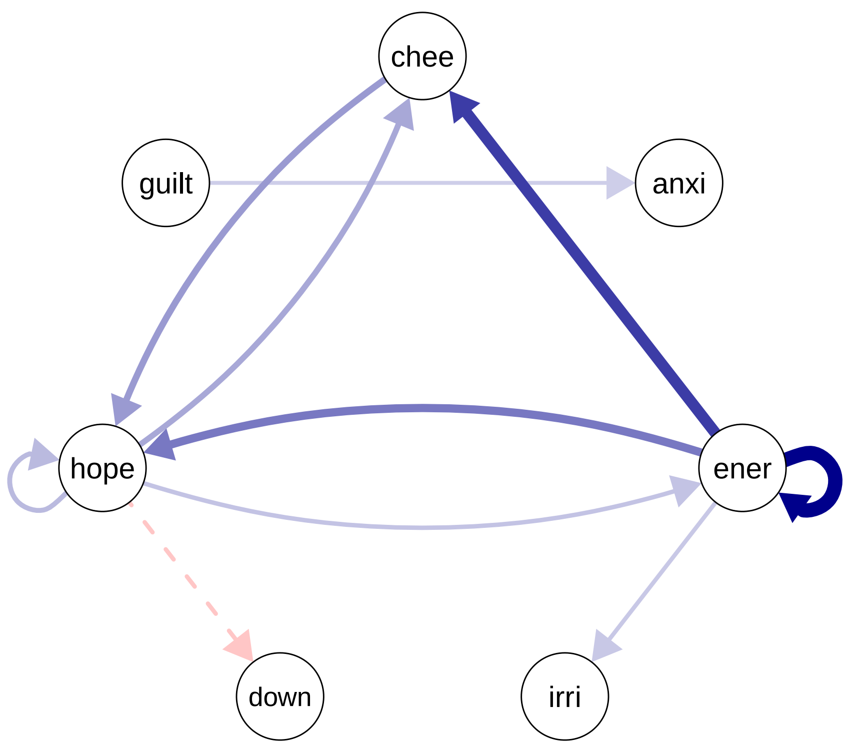

PCT plus ADM non−reg Pt 290 Estpoint 4

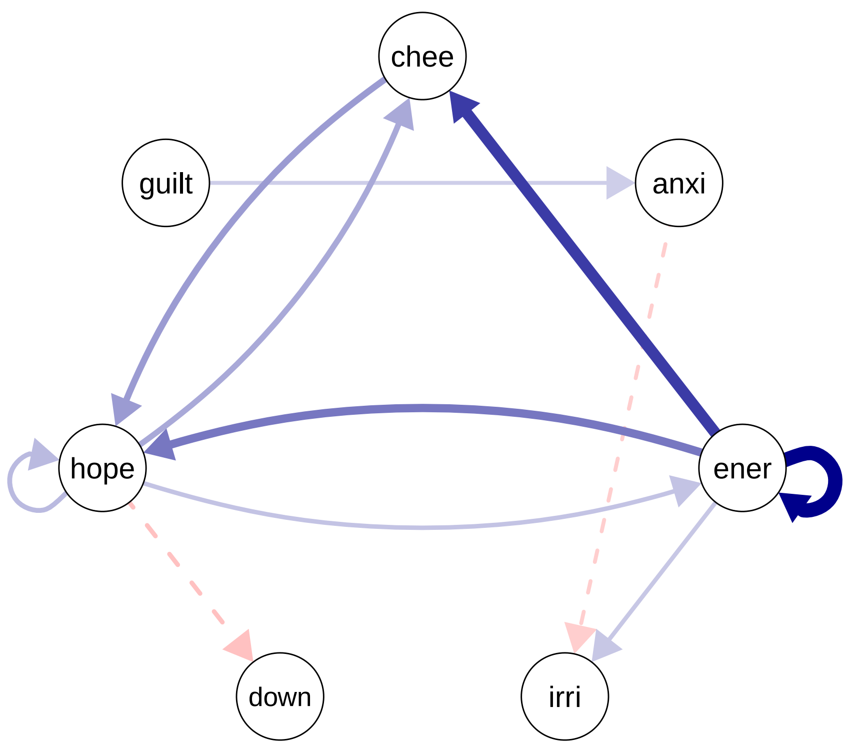

PCT plus ADM non−reg Pt 290 Estpoint 5

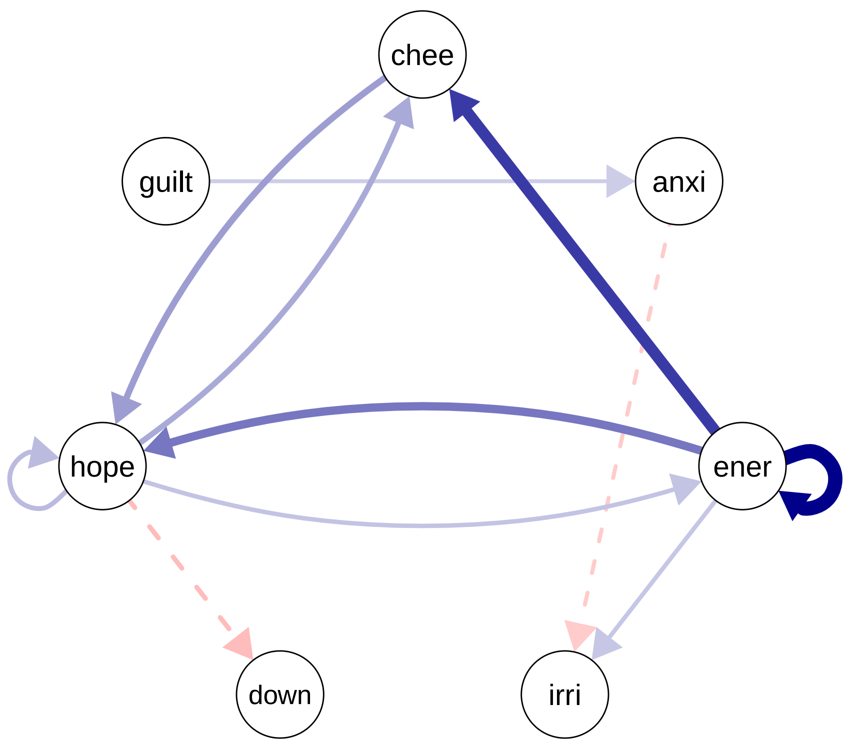

PCT plus ADM non−reg Pt 290 Estpoint 6

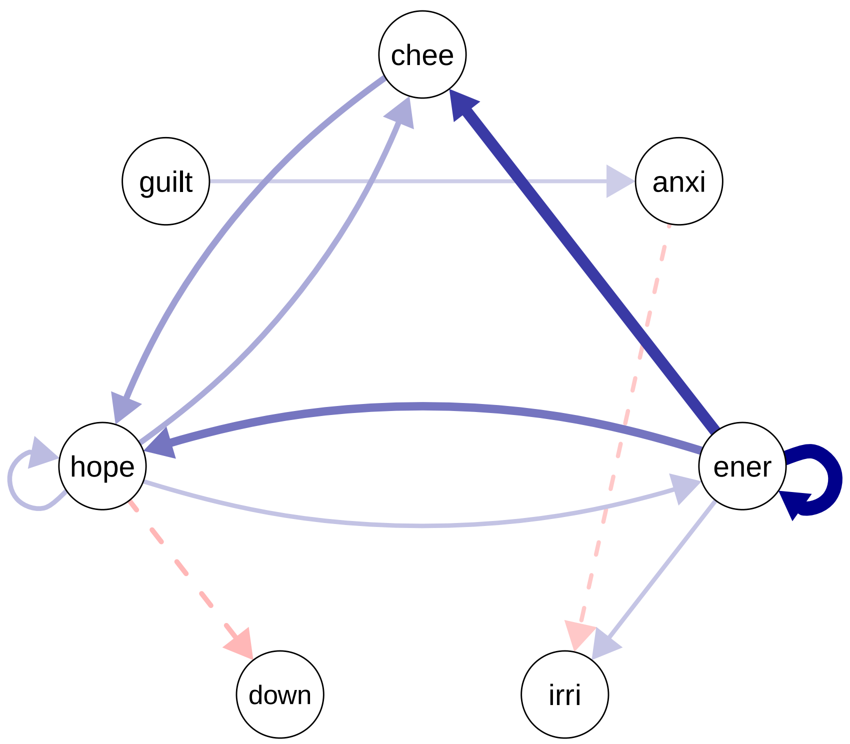

PCT plus ADM non−reg Pt 290 Estpoint 7

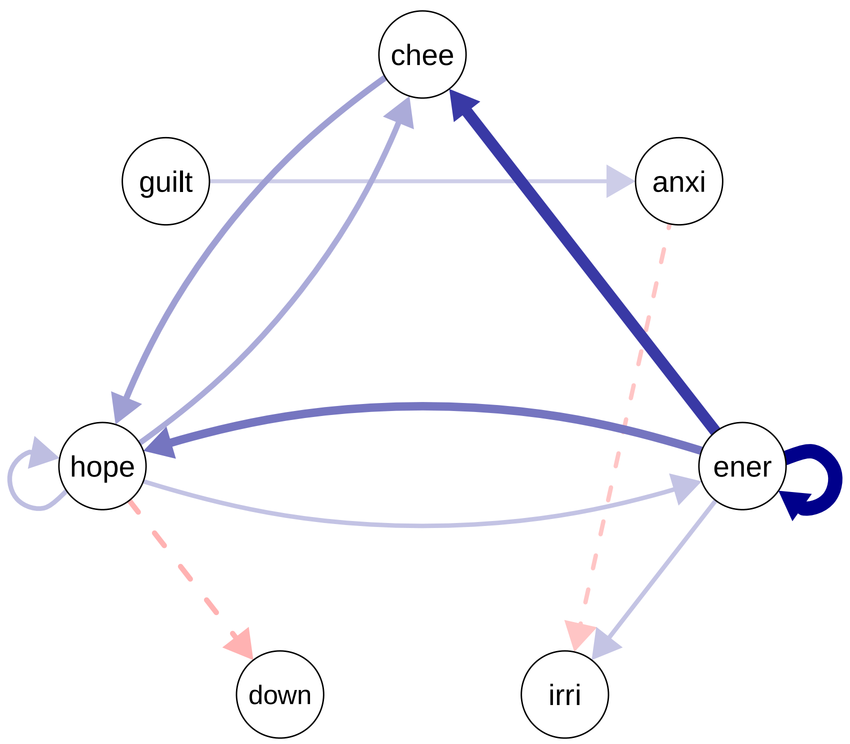

PCT plus ADM non−reg Pt 290 Estpoint 8

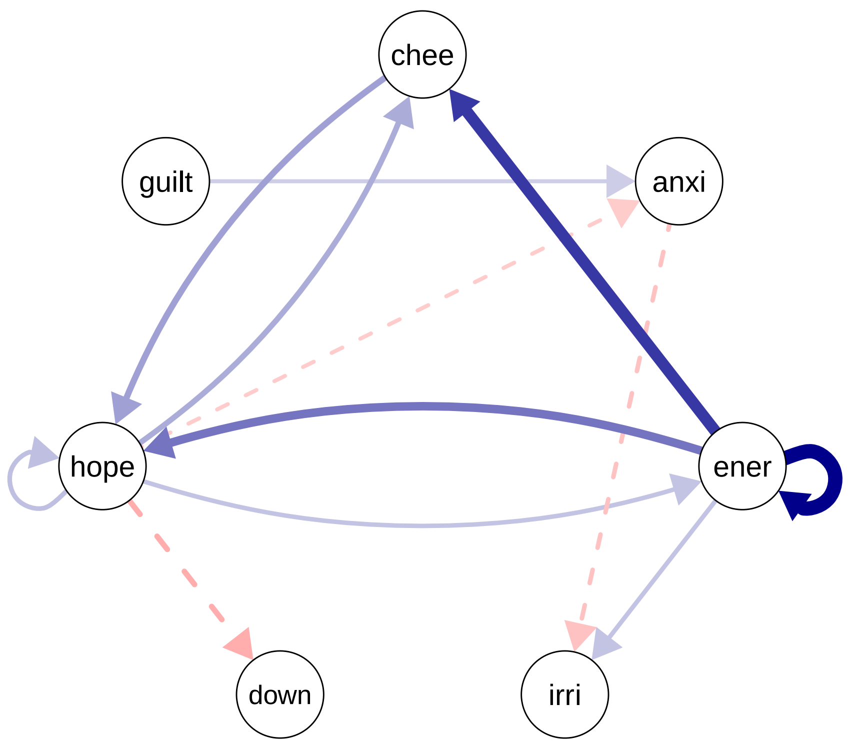

PCT plus ADM non−reg Pt 232 Estpoint 1

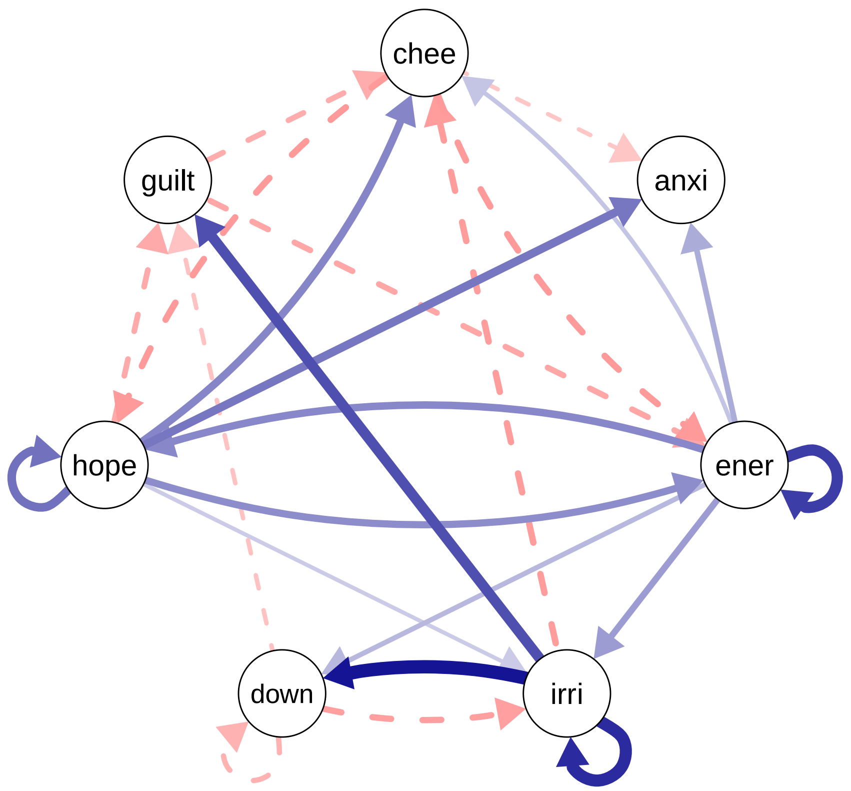

PCT plus ADM non−reg Pt 232 Estpoint 2

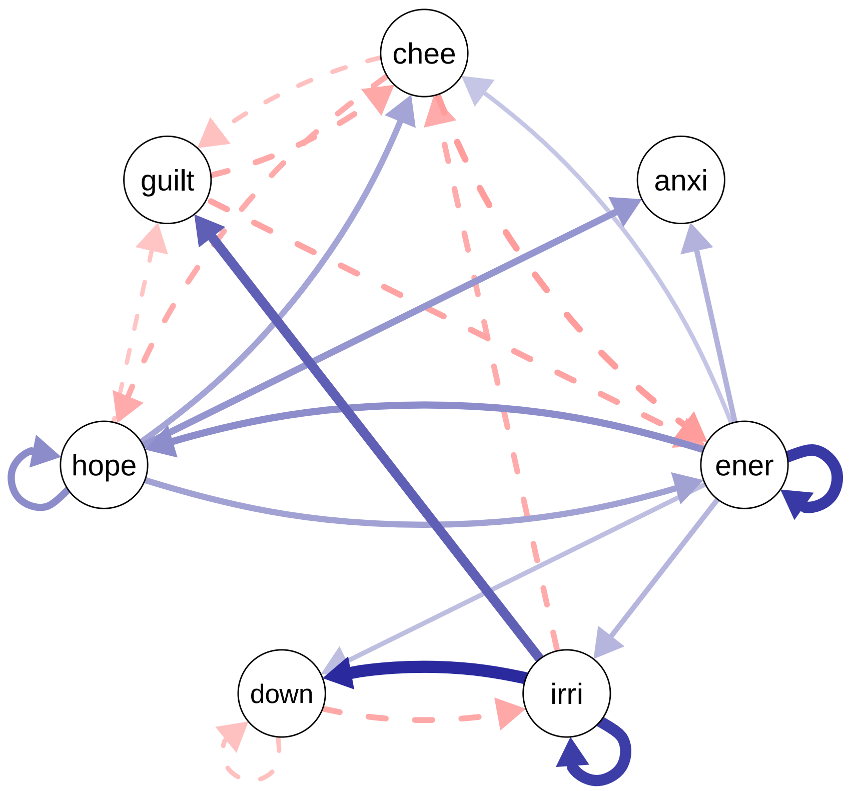

PCT plus ADM non−reg Pt 232 Estpoint 3

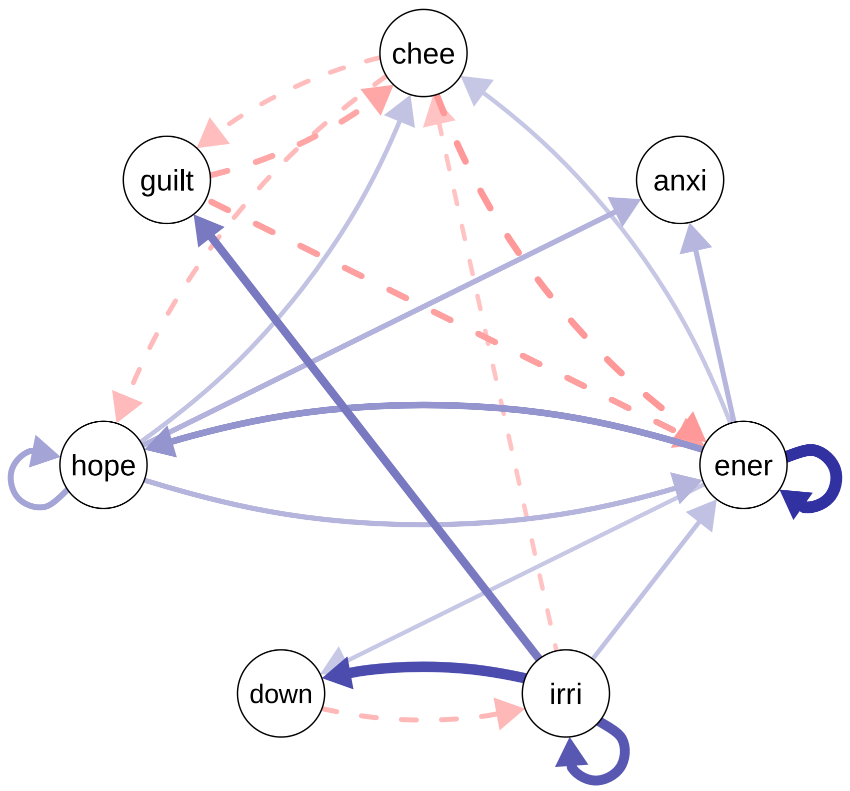

PCT plus ADM non−reg Pt 232 Estpoint 4

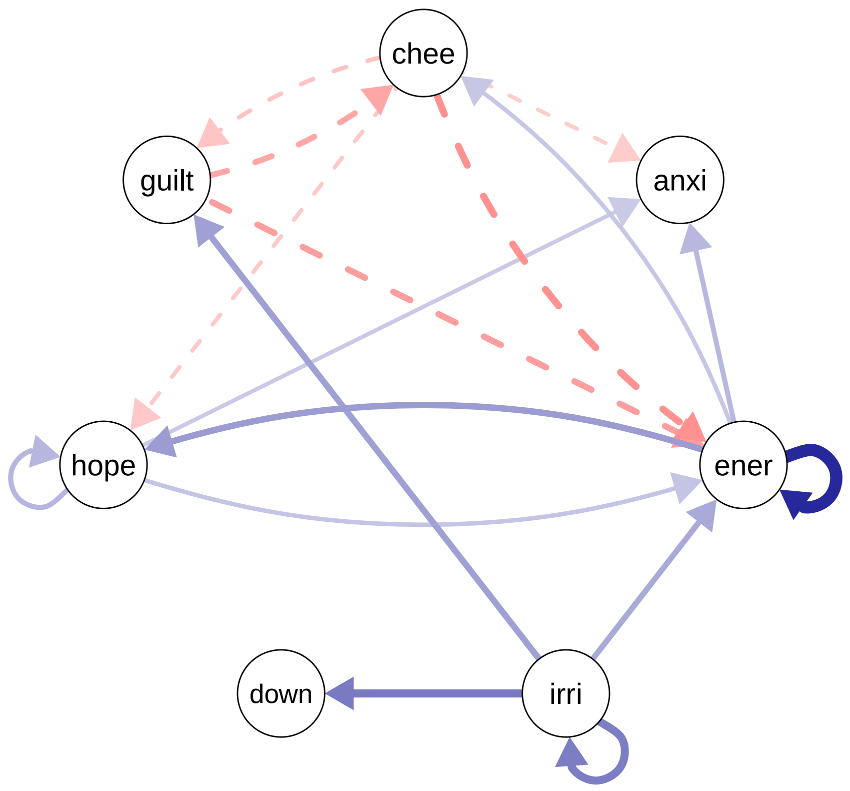

PCT plus ADM non−reg Pt 232 Estpoint 5

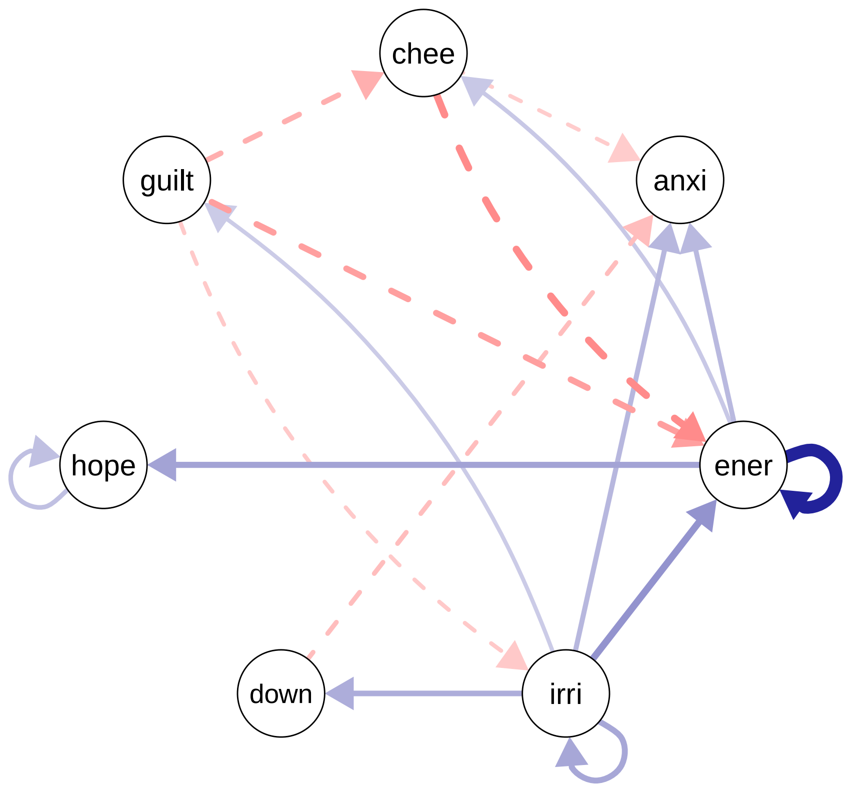

PCT plus ADM non−reg Pt 232 Estpoint 6

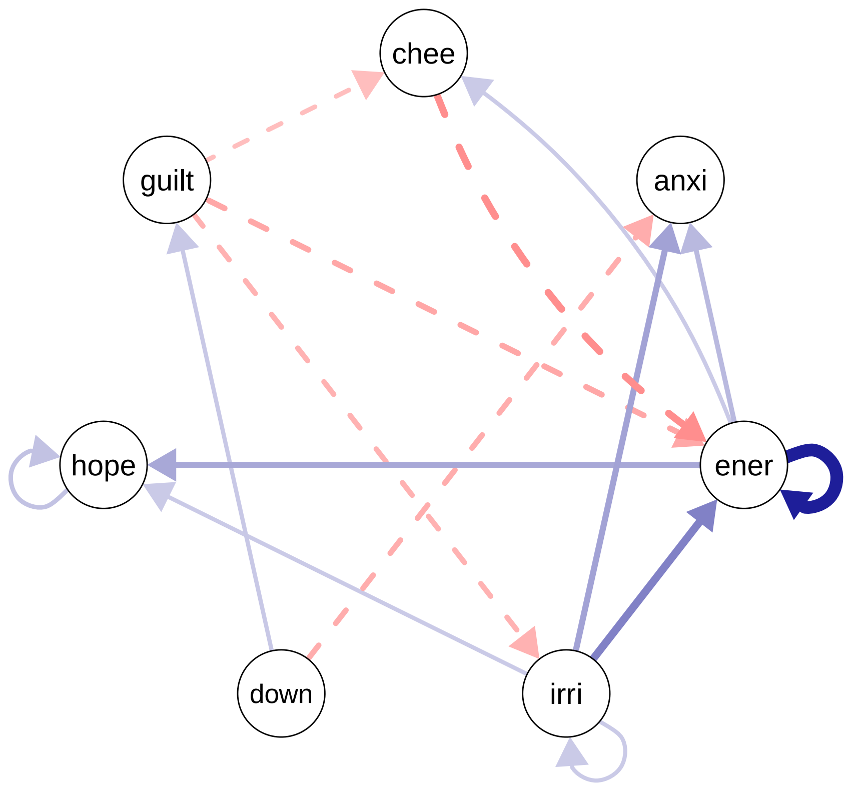

PCT plus ADM non−reg Pt 232 Estpoint 7

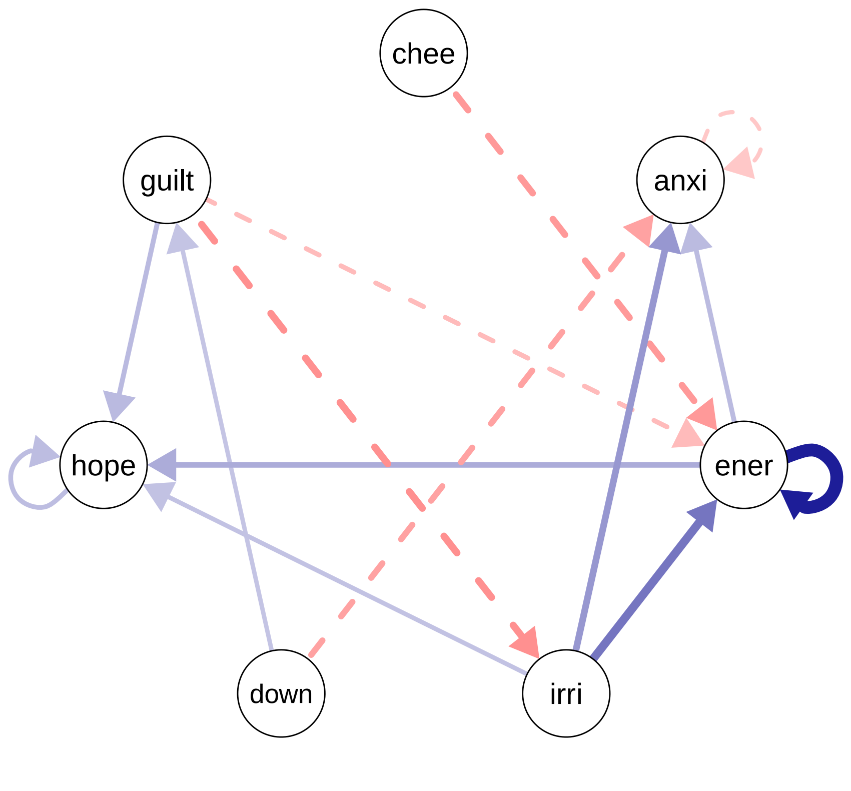

PCT plus ADM non−reg Pt 232 Estpoint 8

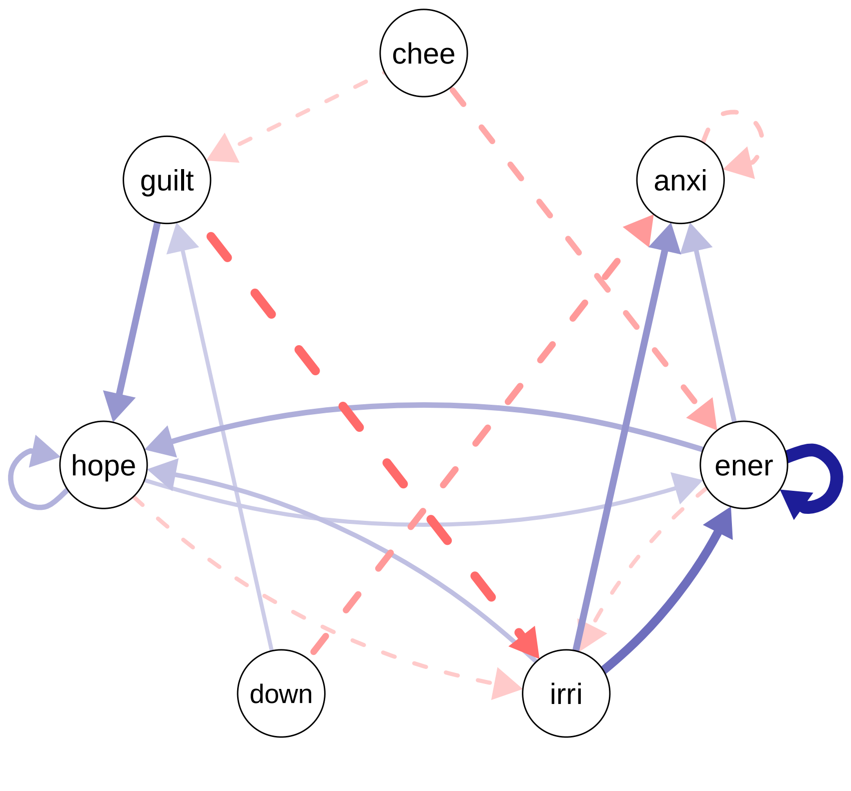

PCT tap ADM non−reg Pt 266 Estpoint 1

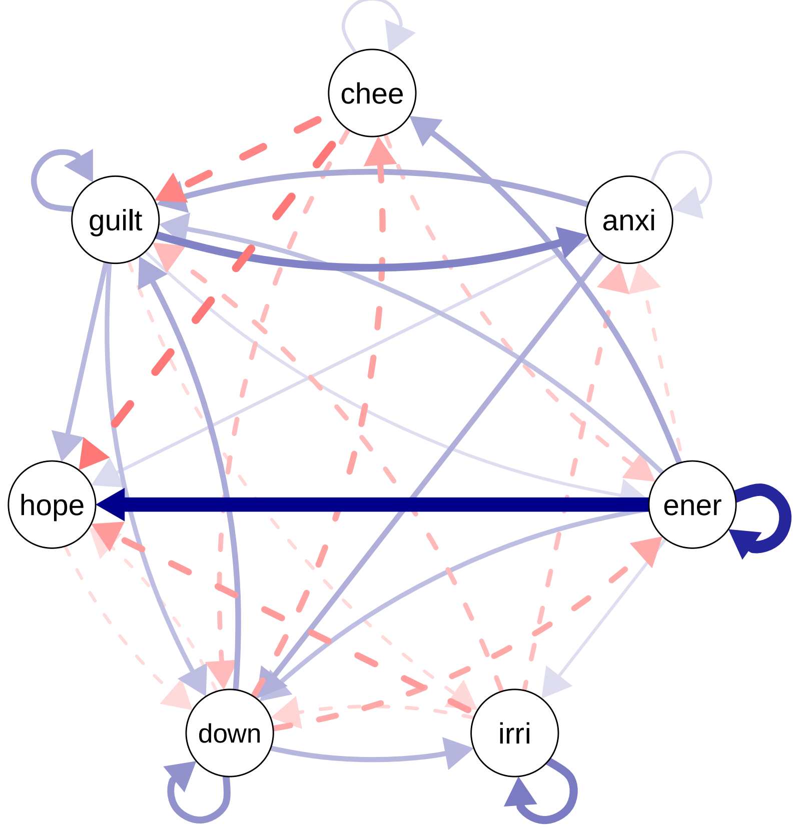

PCT tap ADM non−reg Pt 266 Estpoint 2

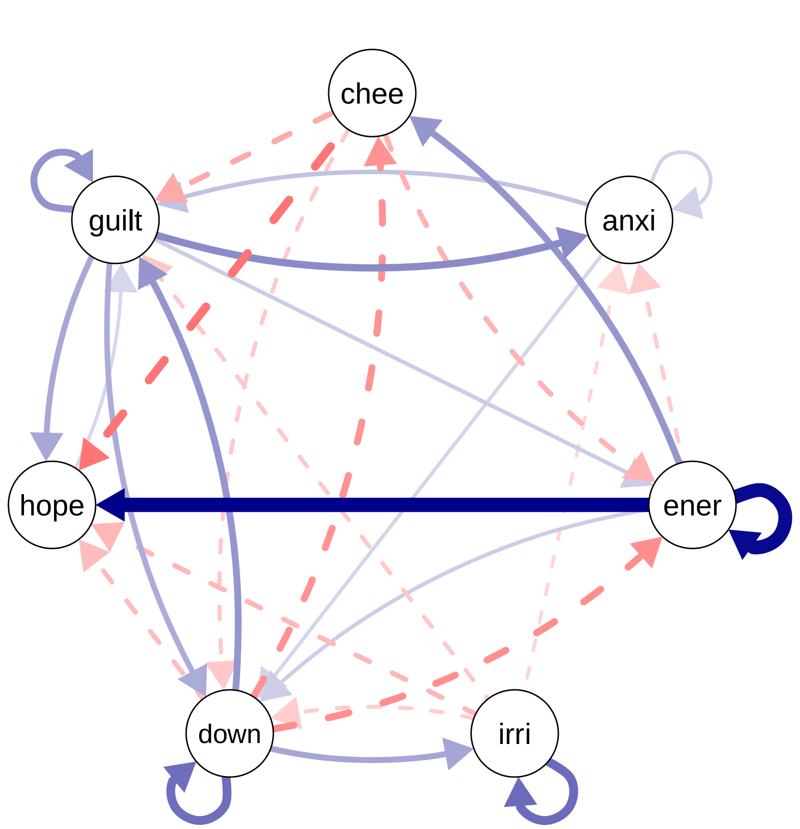

PCT tap ADM non−reg Pt 266 Estpoint 3

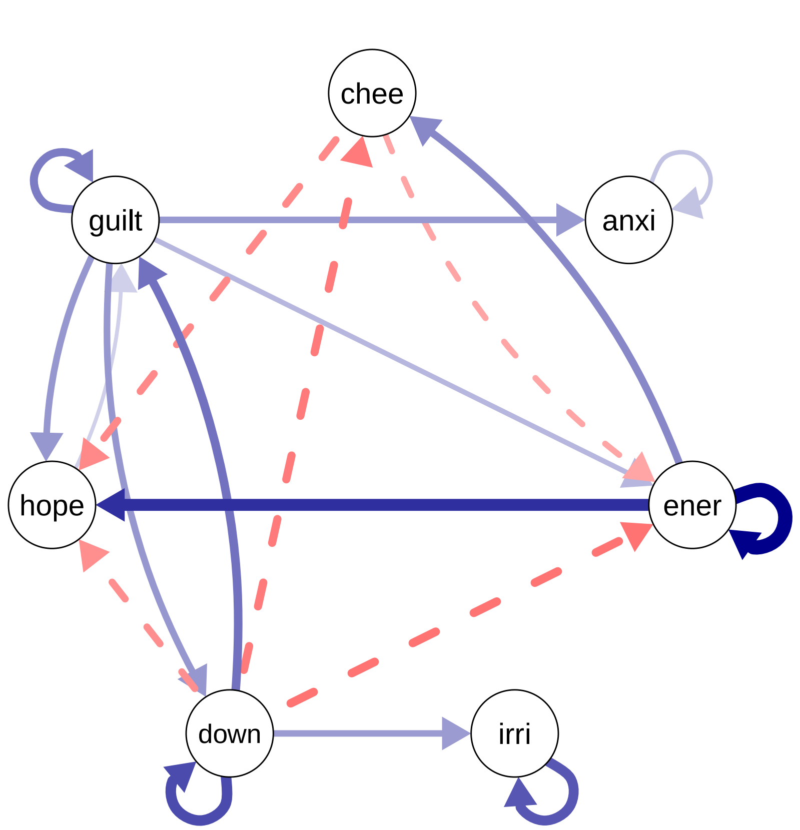

PCT tap ADM non−reg Pt 266 Estpoint 4

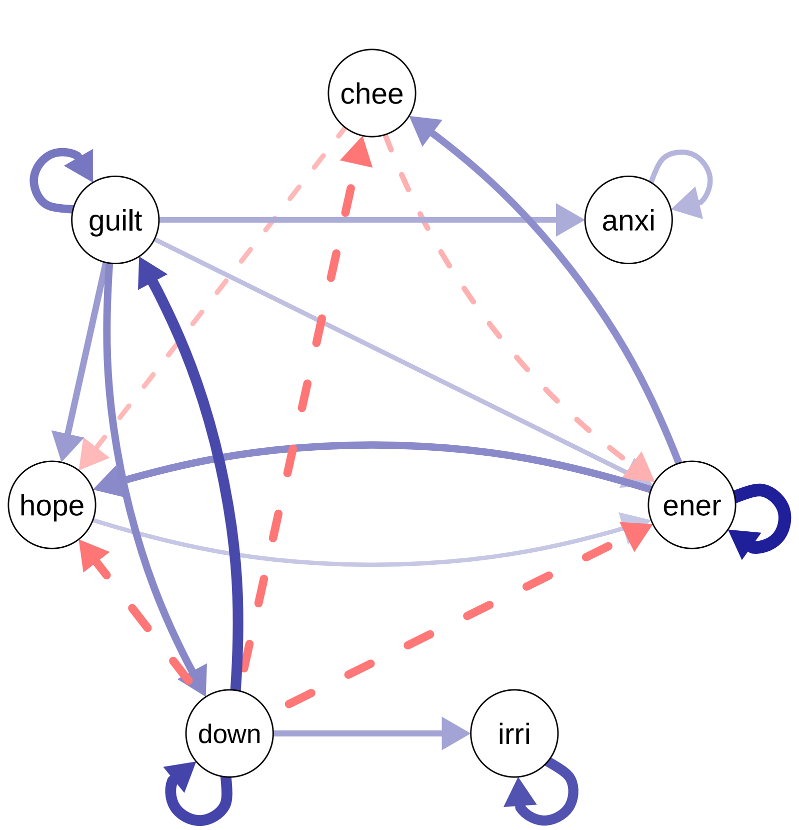

PCT tap ADM non−reg Pt 266 Estpoint 5

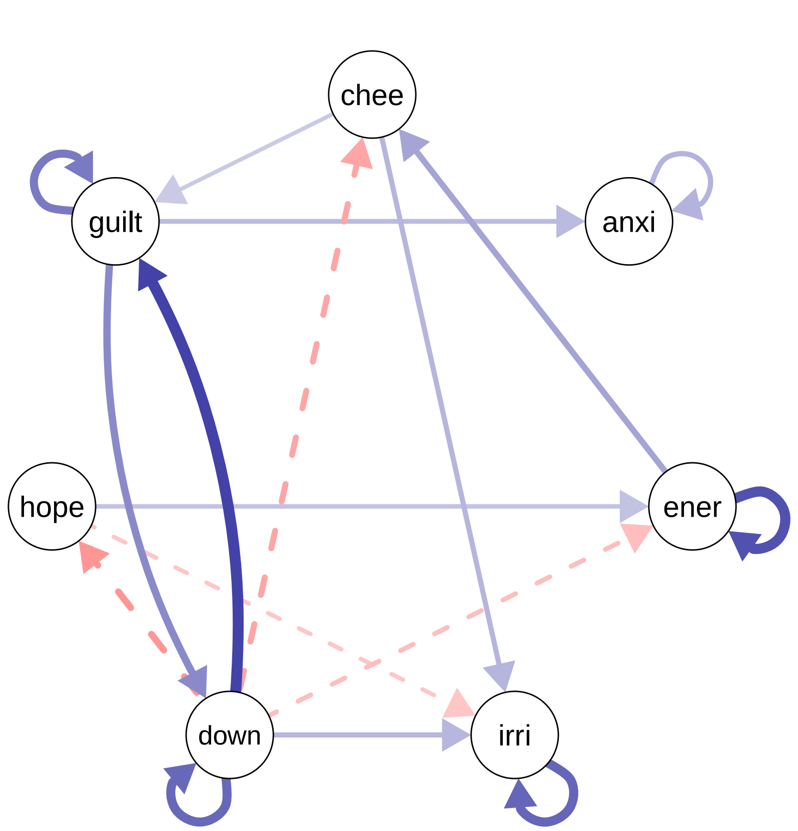

PCT tap ADM non−reg Pt 266 Estpoint 6

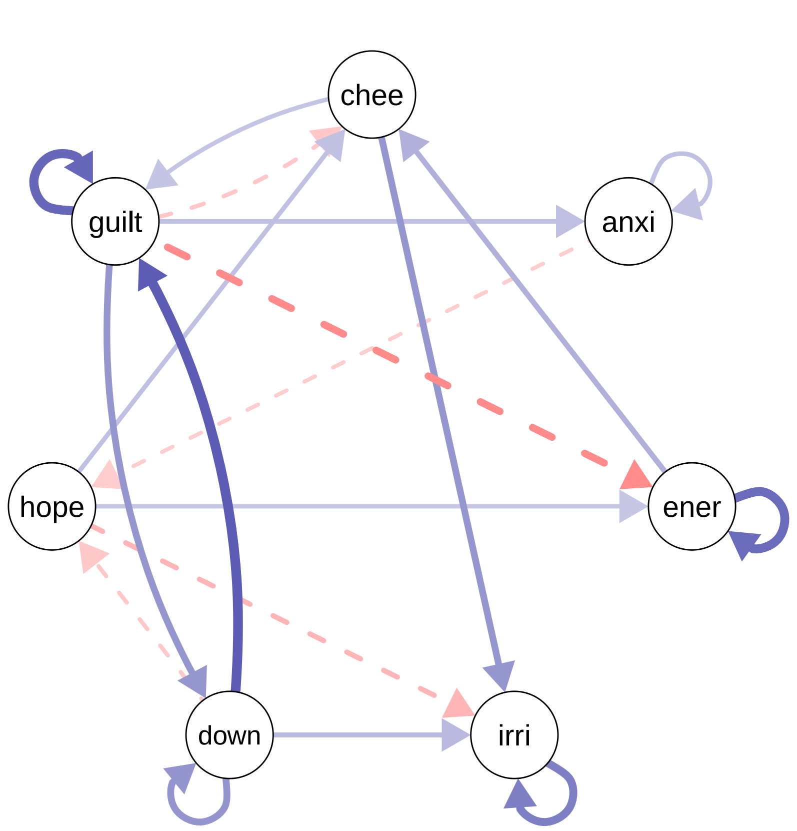

PCT tap ADM non−reg Pt 266 Estpoint 7

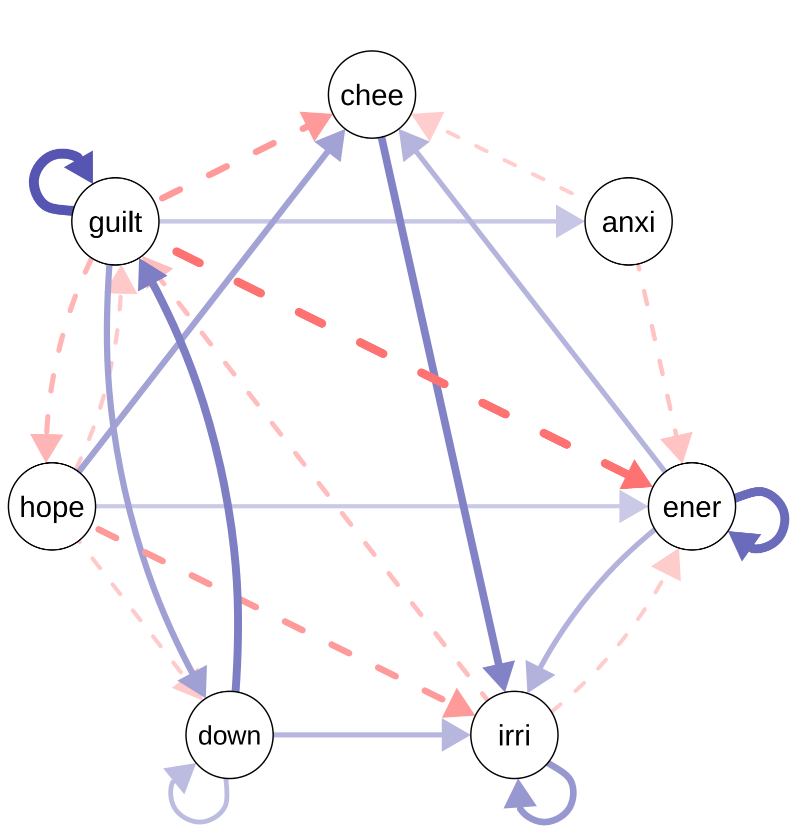

PCT tap ADM non−reg Pt 266 Estpoint 8

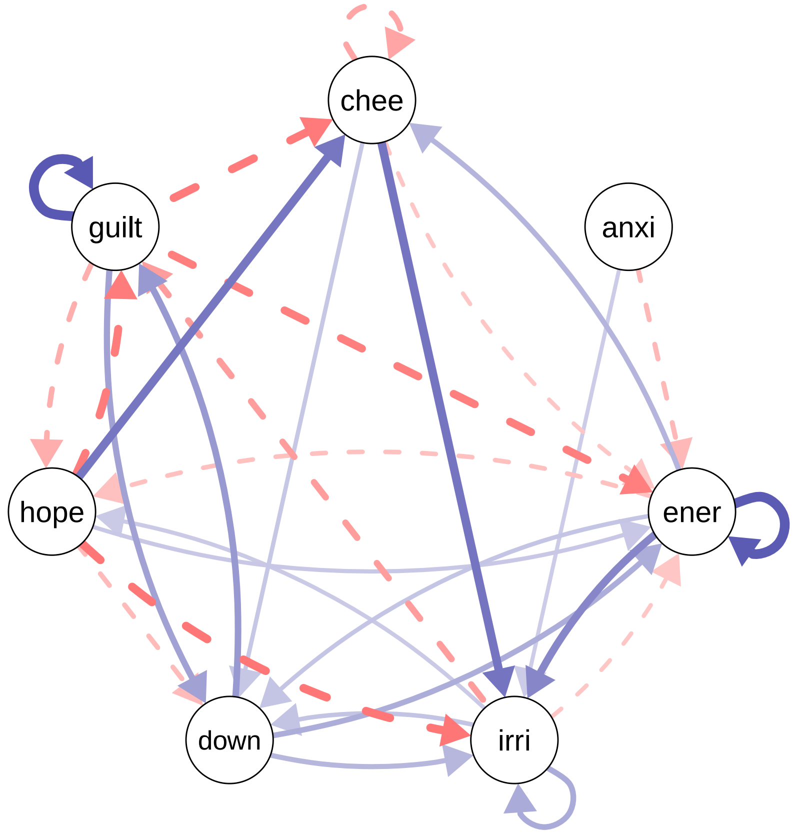

PCT tap ADM non−reg Pt 277 Estpoint 1

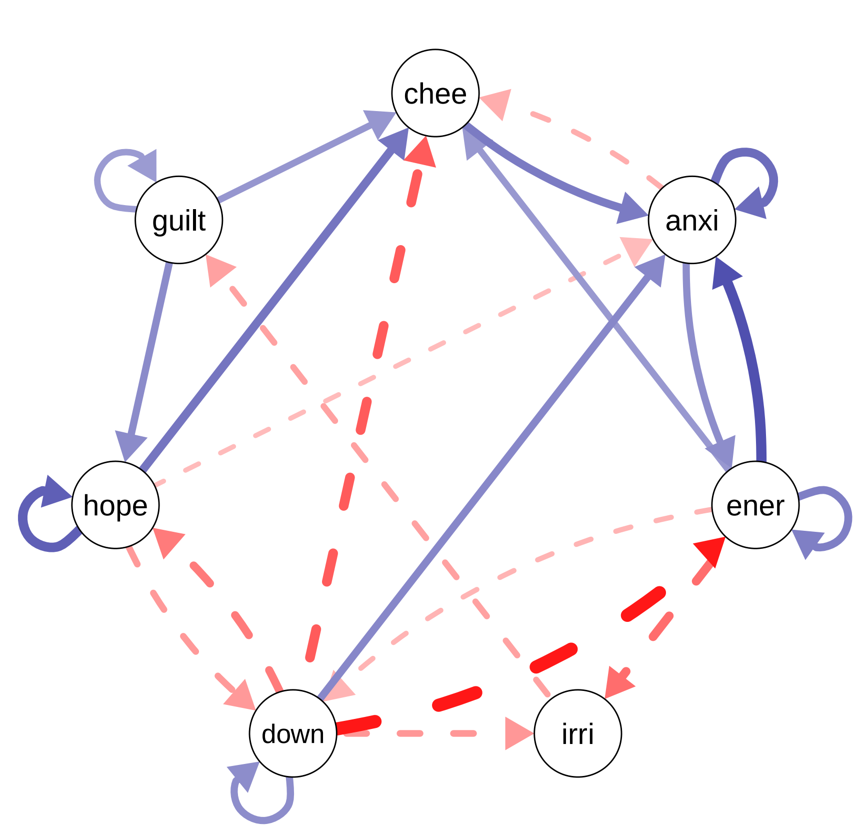

PCT tap ADM non−reg Pt 277 Estpoint 2

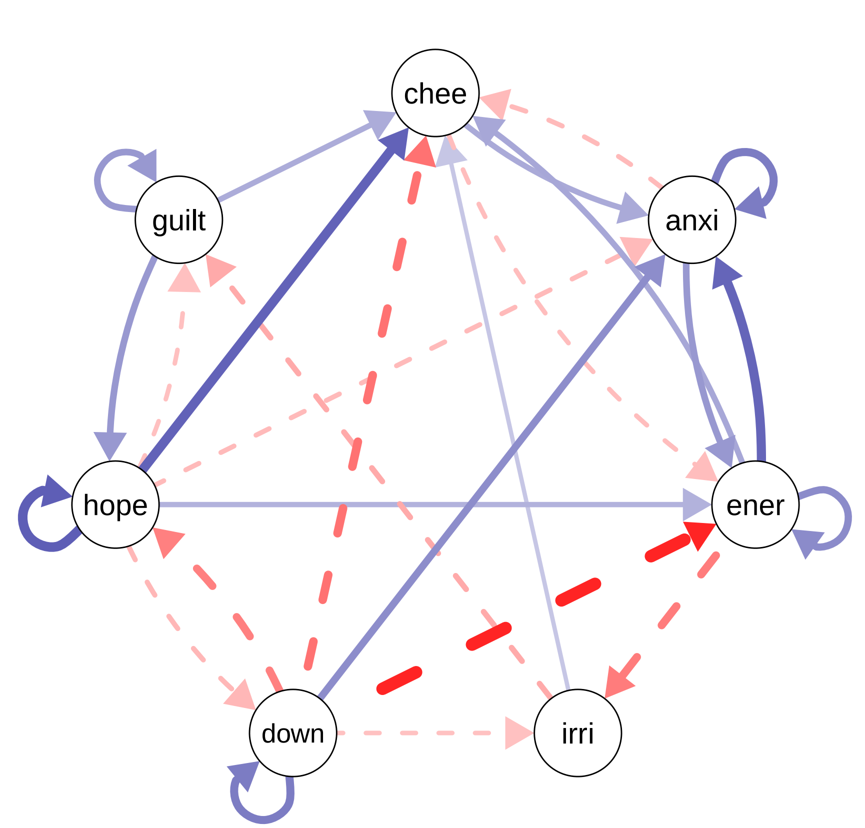

PCT tap ADM non−reg Pt 277 Estpoint 3

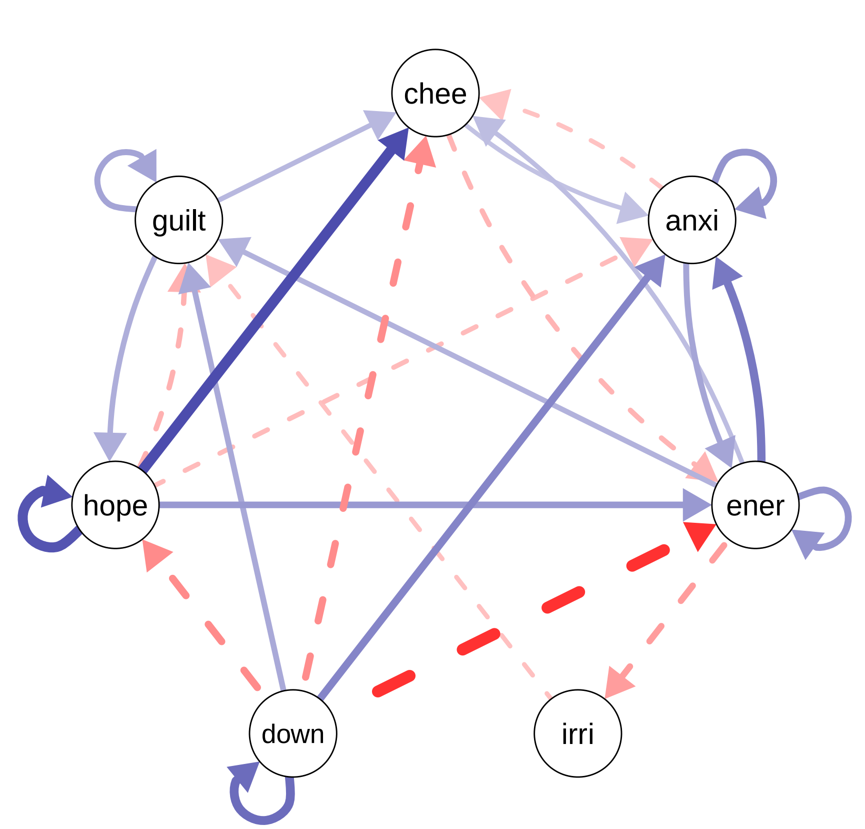

PCT tap ADM non−reg Pt 277 Estpoint 4

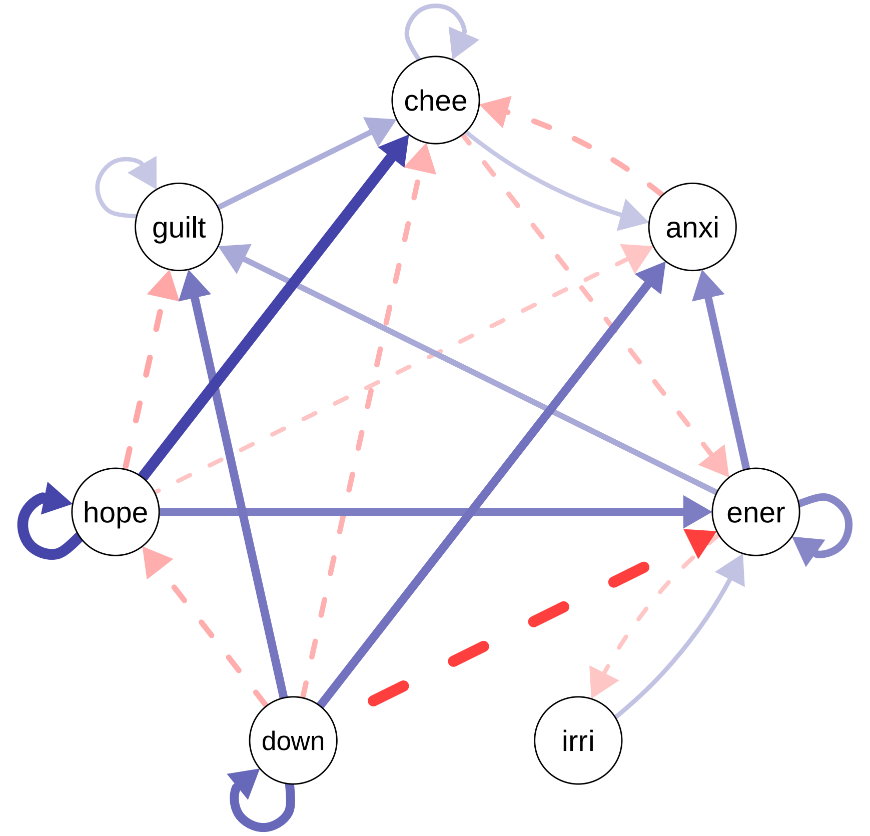

PCT tap ADM non−reg Pt 277 Estpoint 5

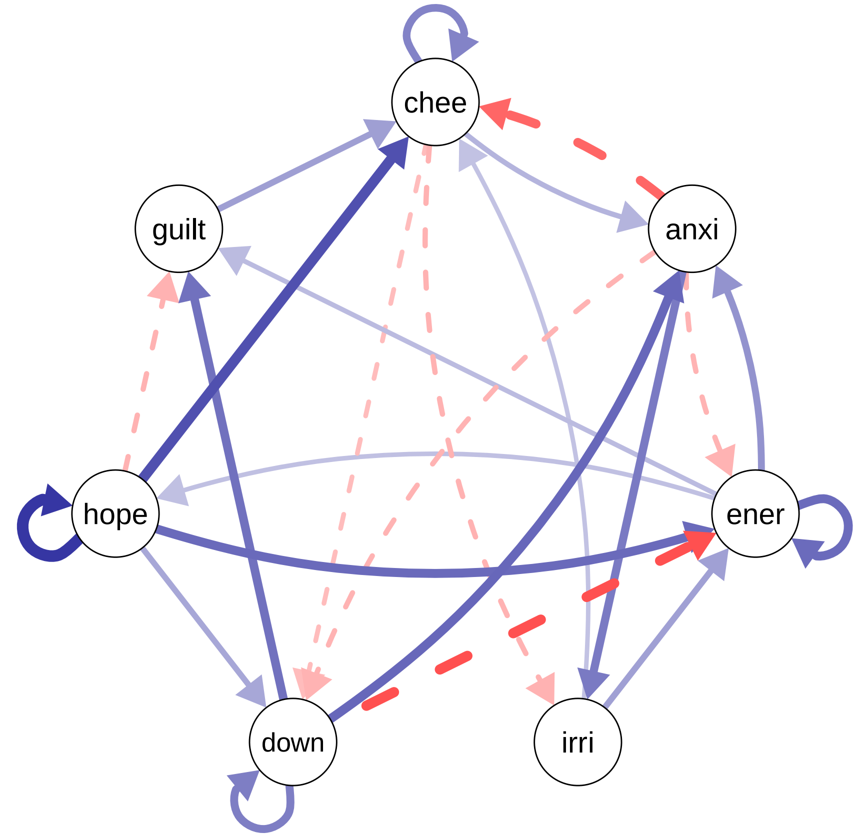

PCT tap ADM non−reg Pt 277 Estpoint 6

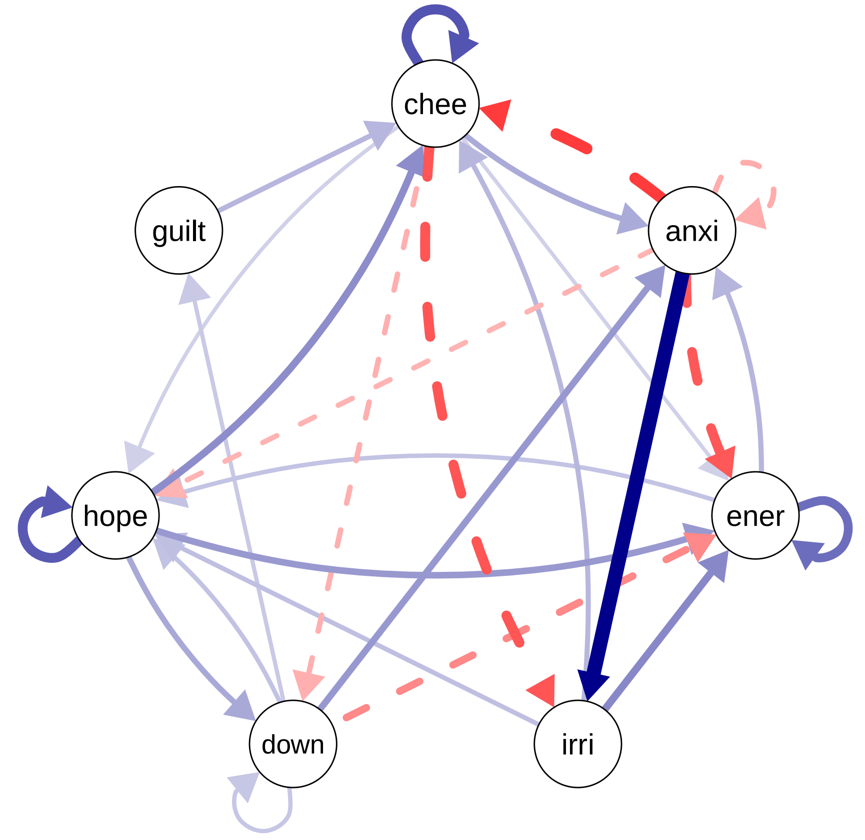

PCT tap ADM non−reg Pt 277 Estpoint 7

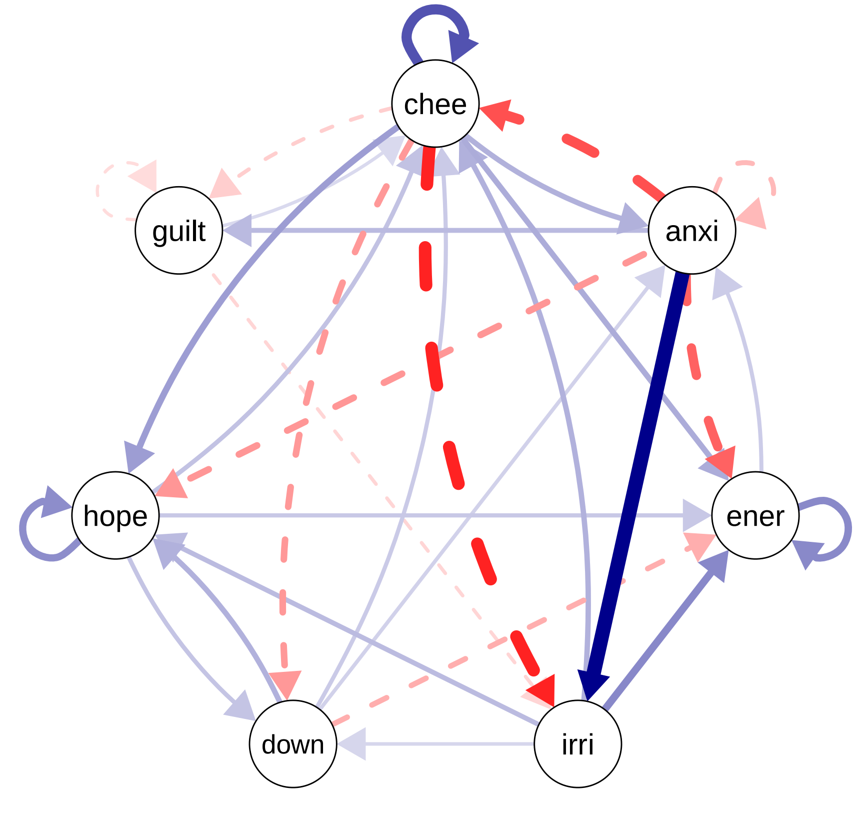

PCT tap ADM non−reg Pt 277 Estpoint 8

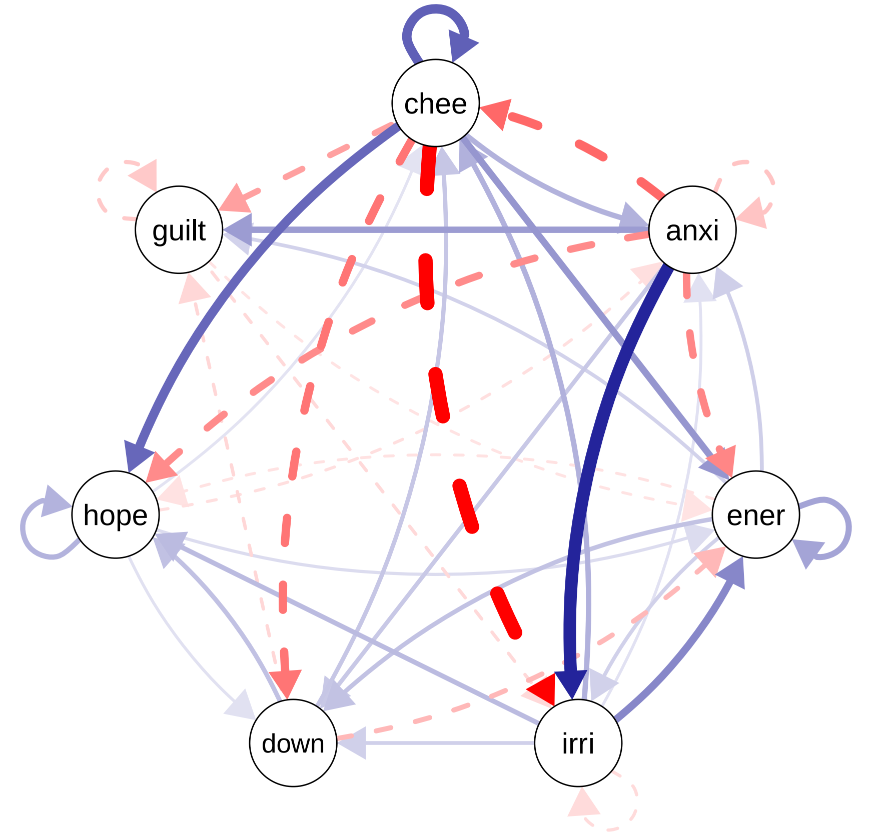

PCT tap ADM non−reg Pt 260 Estpoint 1

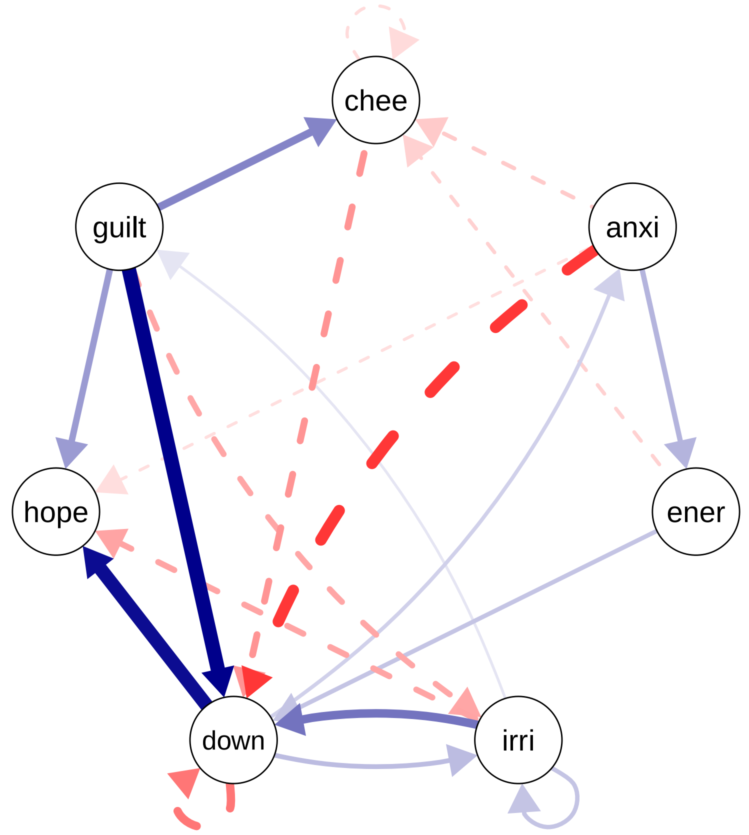

PCT tap ADM non−reg Pt 260 Estpoint 2

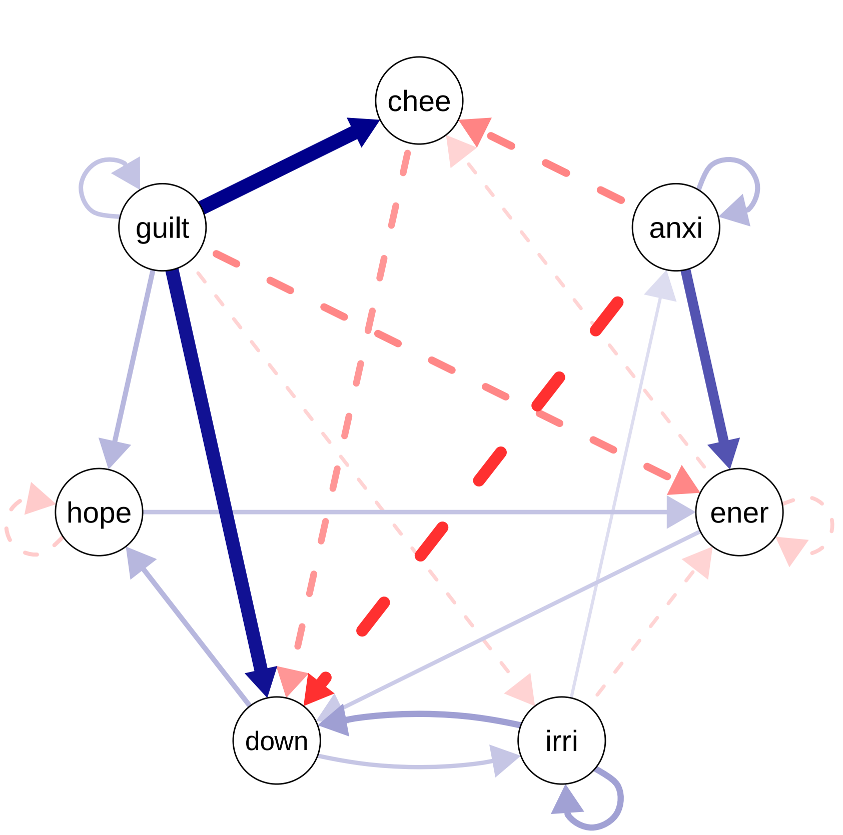

PCT tap ADM non−reg Pt 260 Estpoint 3

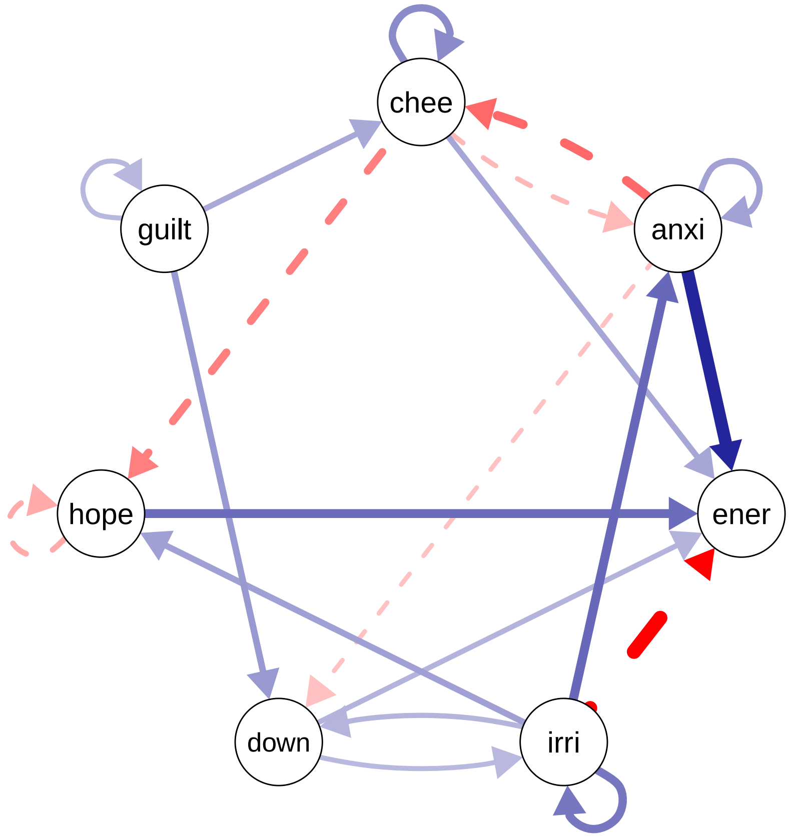

PCT tap ADM non−reg Pt 260 Estpoint 4

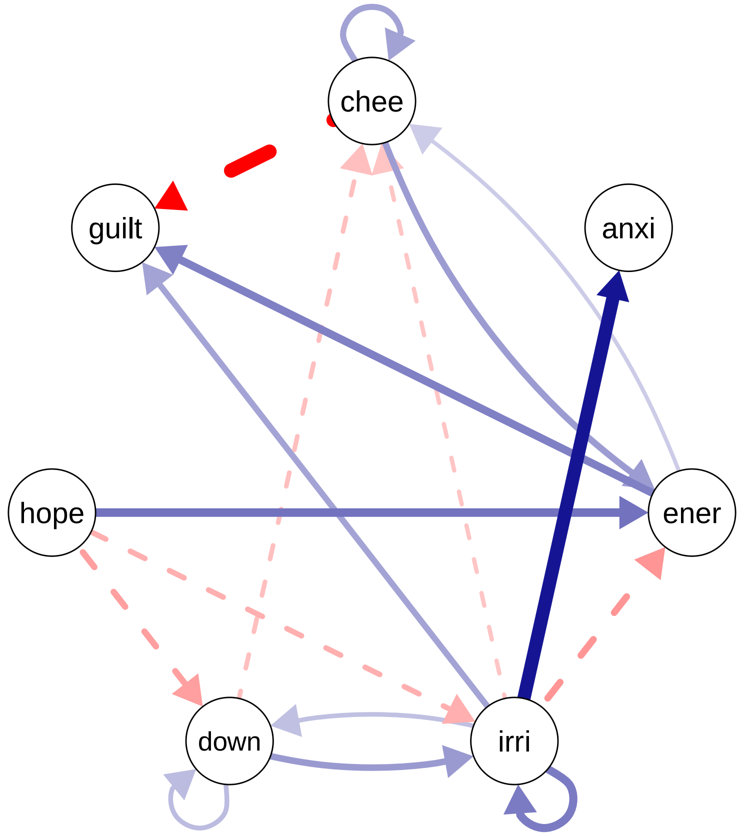

PCT tap ADM non−reg Pt 260 Estpoint 5

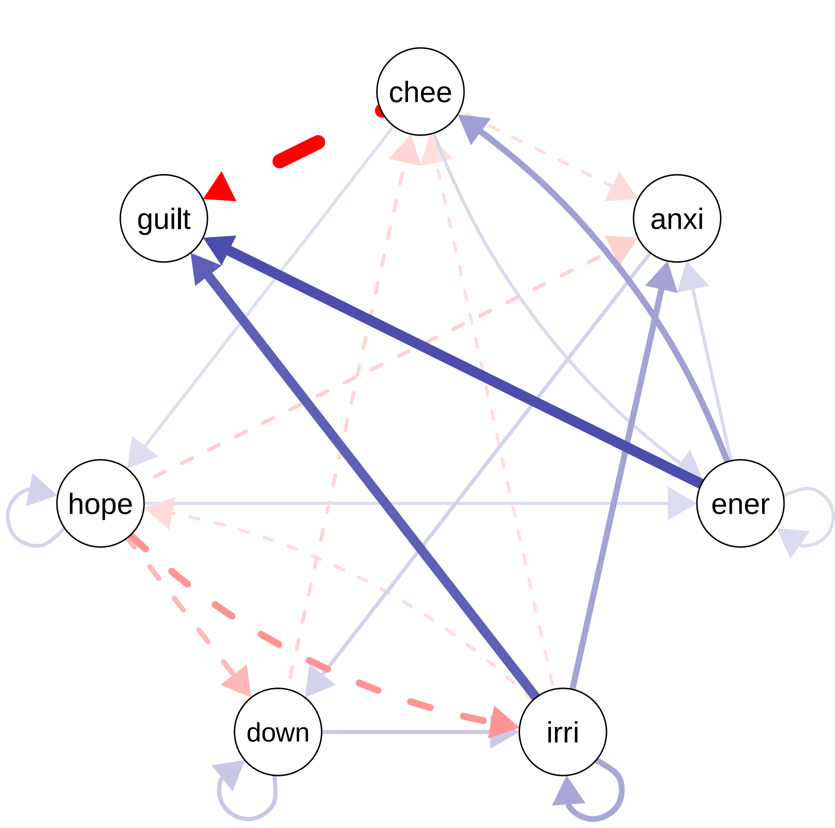

PCT tap ADM non−reg Pt 260 Estpoint 6

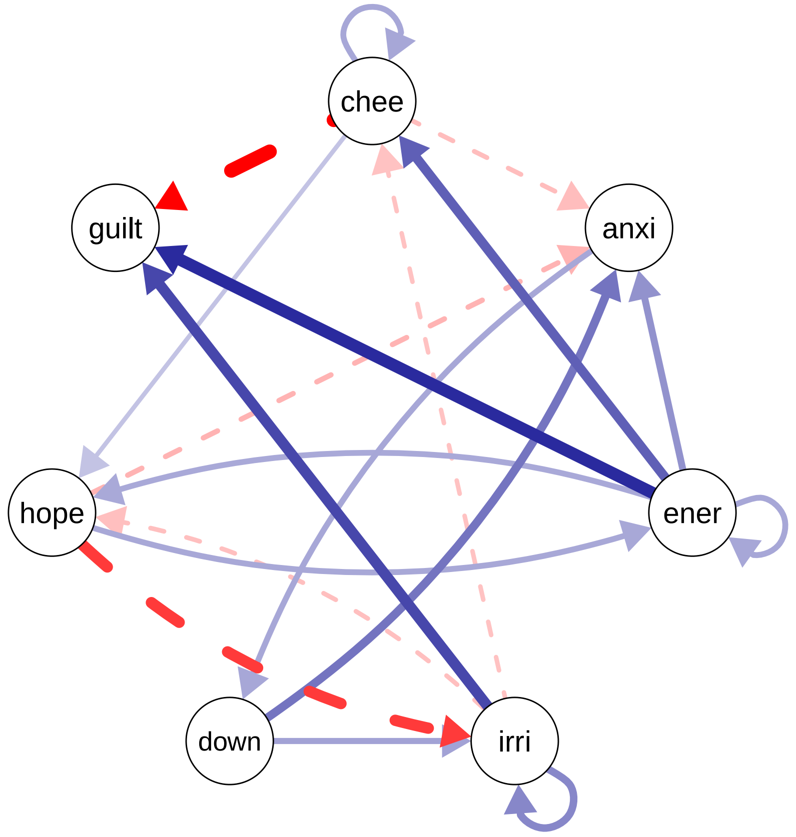

PCT tap ADM non−reg Pt 260 Estpoint 7

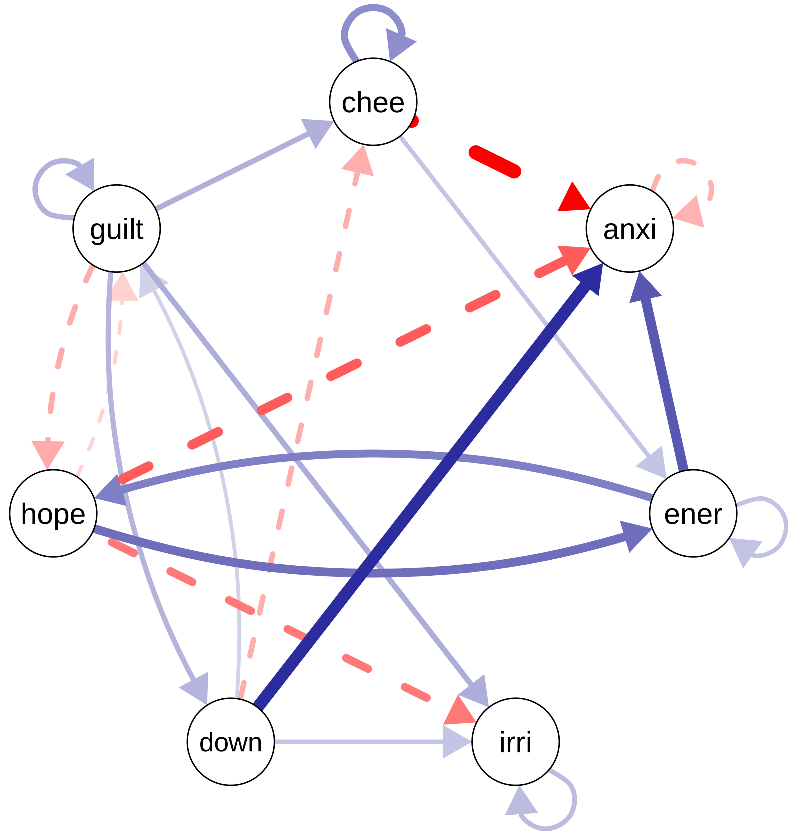

PCT tap ADM non−reg Pt 260 Estpoint 8

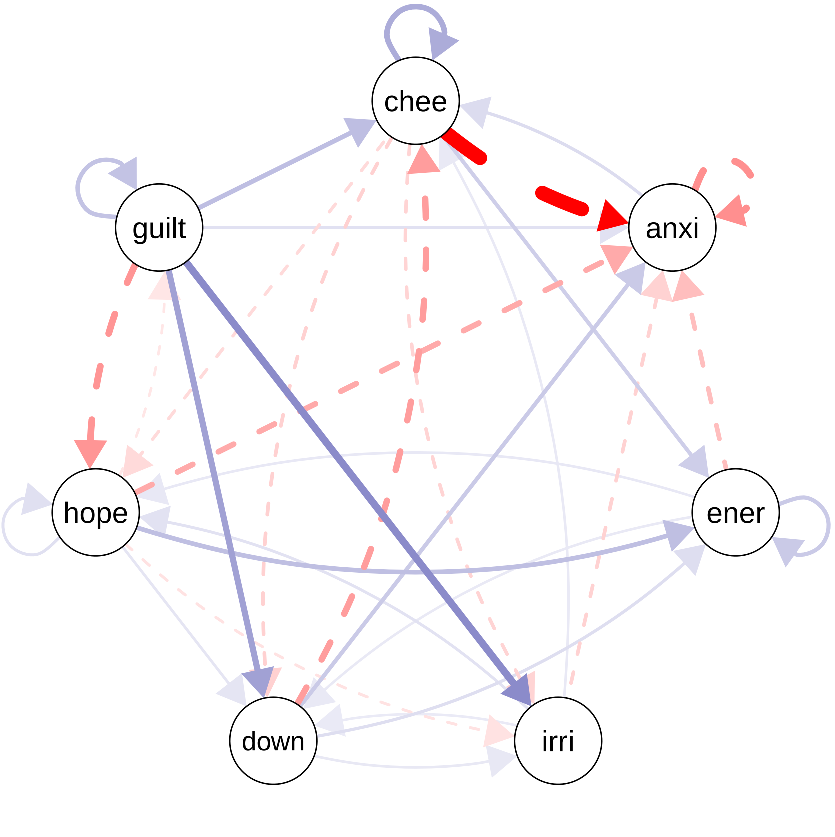

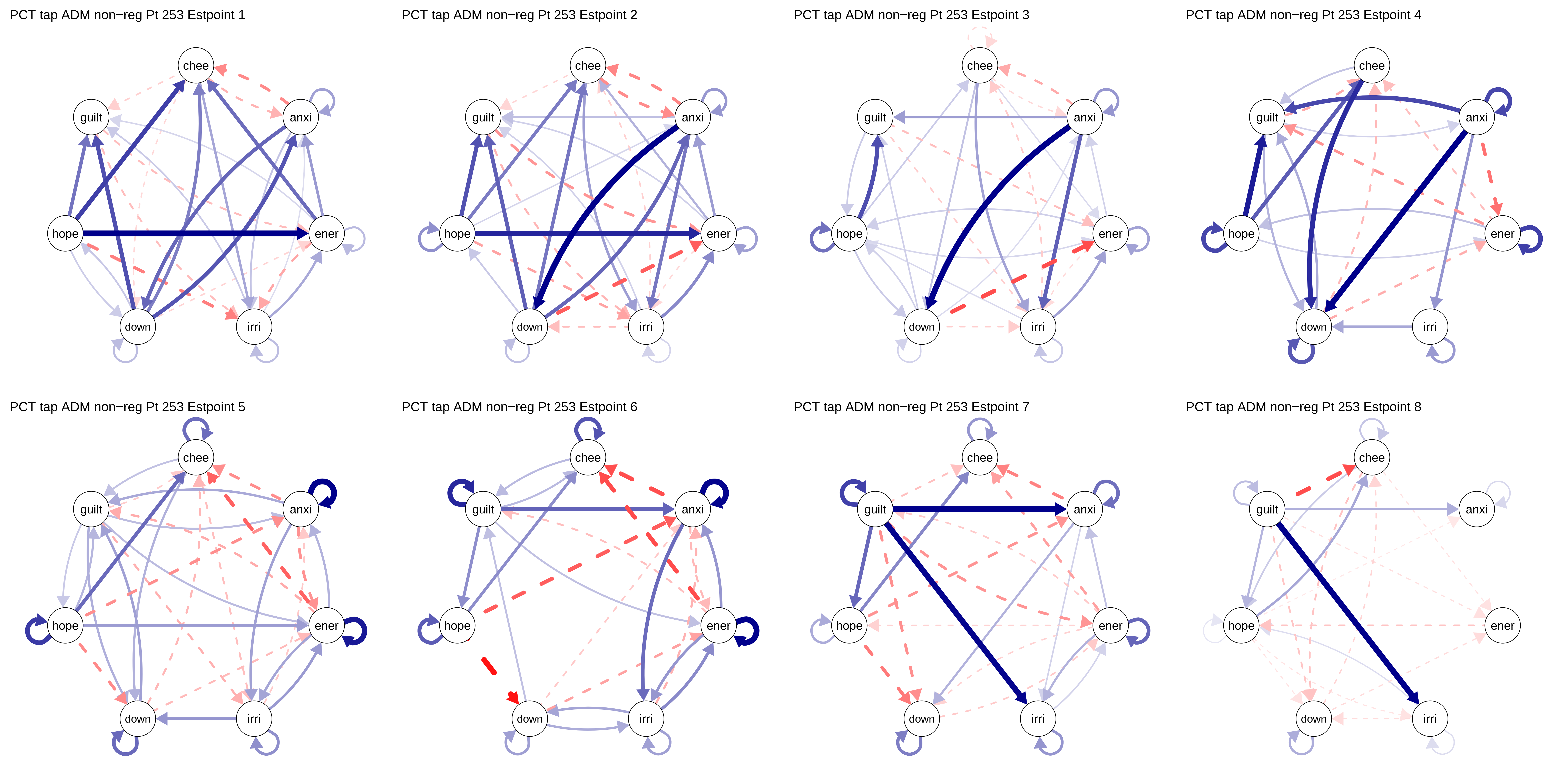
PCT tap ADM non−reg Pt 253 Estpoint 1
PCT tap ADM non−reg Pt 253 Estpoint 2
PCT tap ADM non−reg Pt 253 Estpoint 3
PCT tap ADM non−reg Pt 253 Estpoint 4
PCT tap ADM non−reg Pt 253 Estpoint 5
PCT tap ADM non−reg Pt 253 Estpoint 6
PCT tap ADM non−reg Pt 253 Estpoint 7
PCT tap ADM non−reg Pt 253 Estpoint 8
chee
guilt
anxi
hope
ener
down
irri

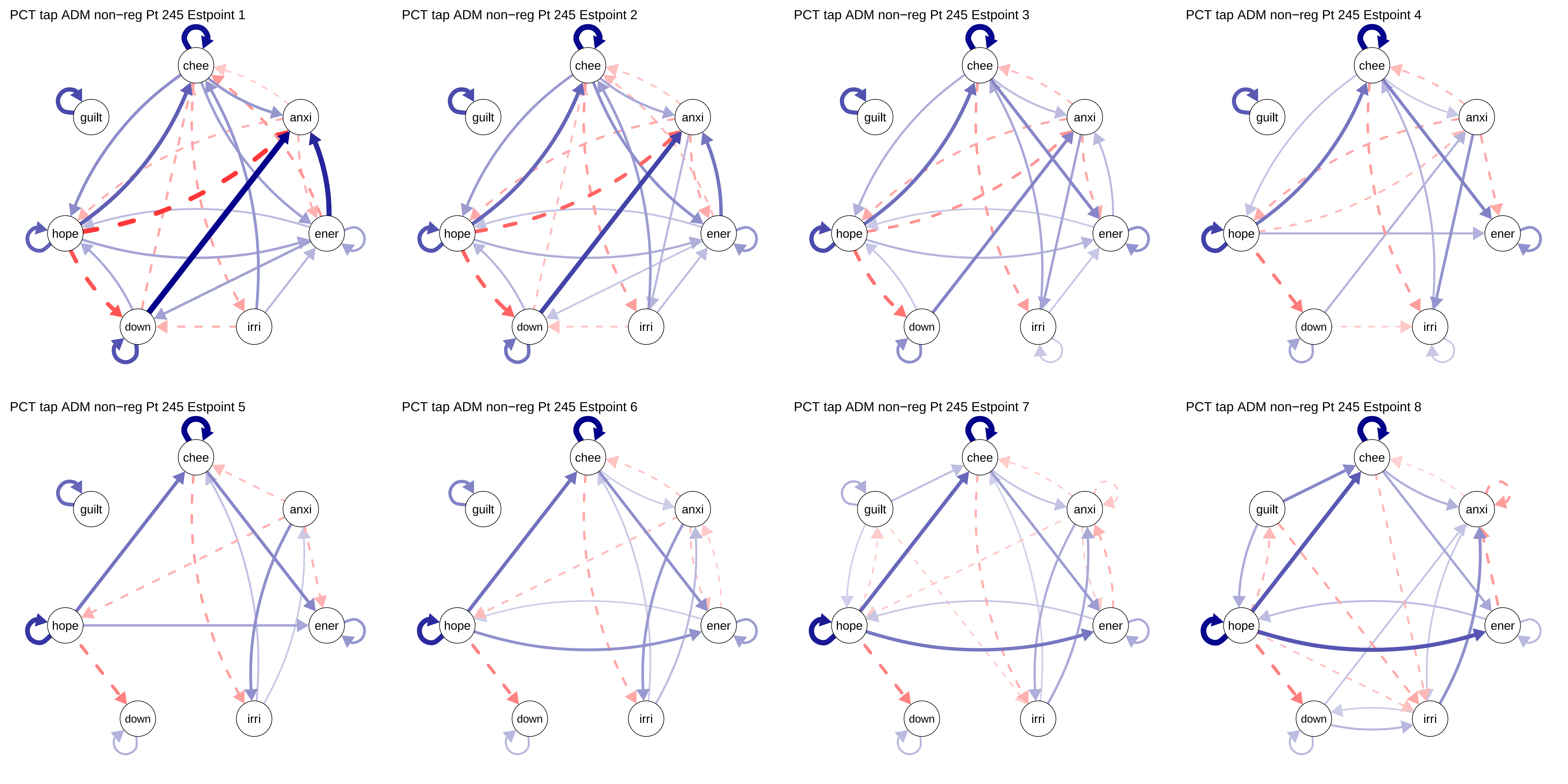
PCT tap ADM non−reg Pt 245 Estpoint 1
PCT tap ADM non−reg Pt 245 Estpoint 2
PCT tap ADM non−reg Pt 245 Estpoint 3
PCT tap ADM non−reg Pt 245 Estpoint 4
PCT tap ADM non−reg Pt 245 Estpoint 5
PCT tap ADM non−reg Pt 245 Estpoint 6
PCT tap ADM non−reg Pt 245 Estpoint 7
PCT tap ADM non−reg Pt 245 Estpoint 8
chee
guilt
anxi
hope
ener
down
irri

PCT tap ADM non−reg Pt 286 Estpoint 1

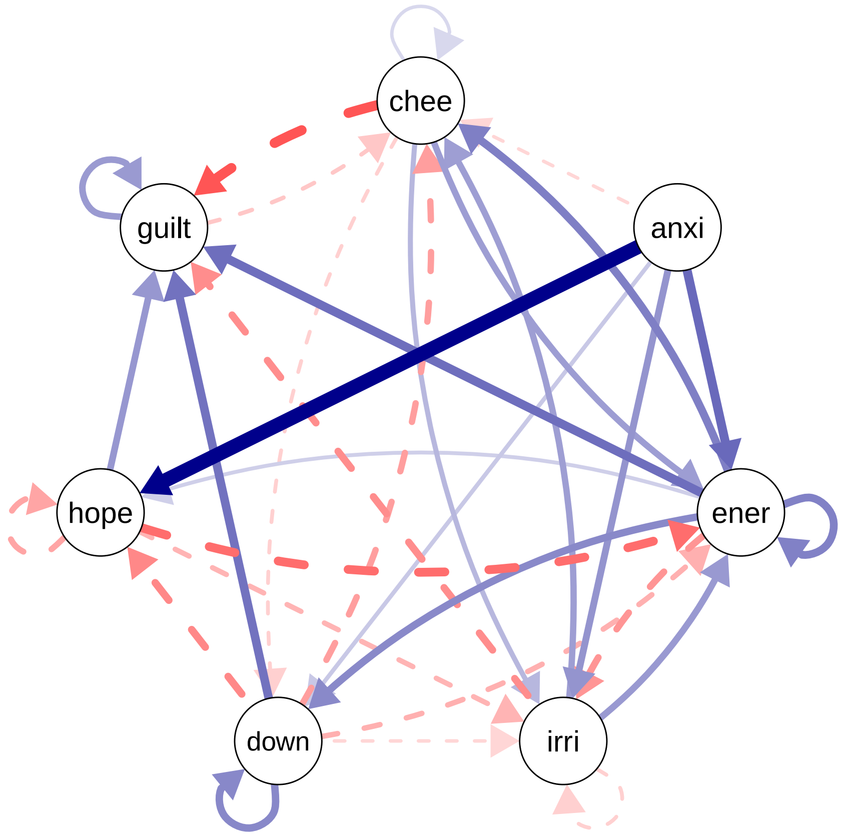

PCT tap ADM non−reg Pt 286 Estpoint 2

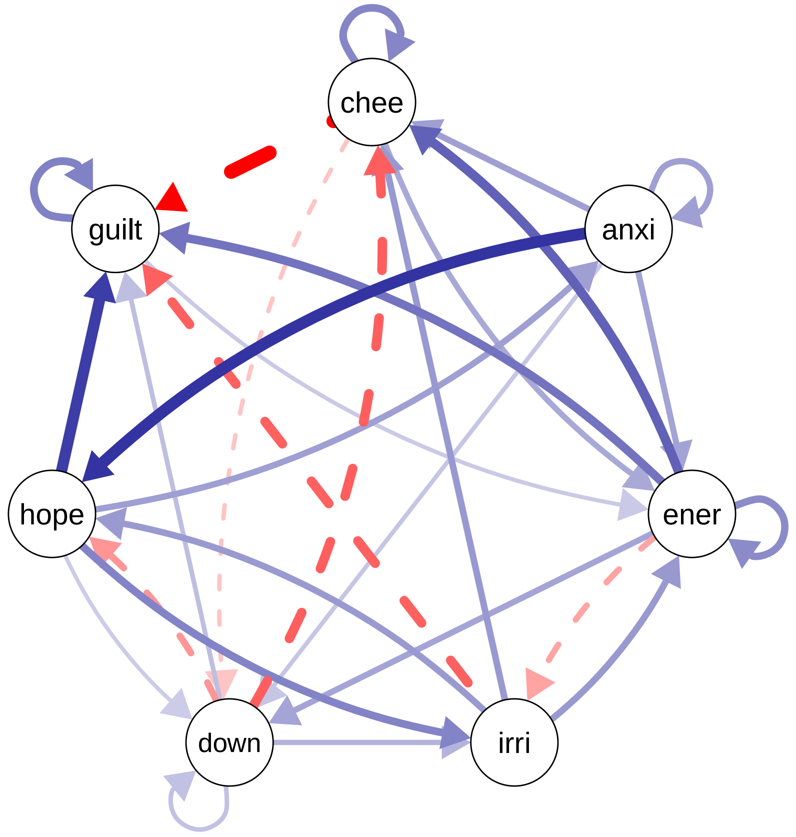

PCT tap ADM non−reg Pt 286 Estpoint 3

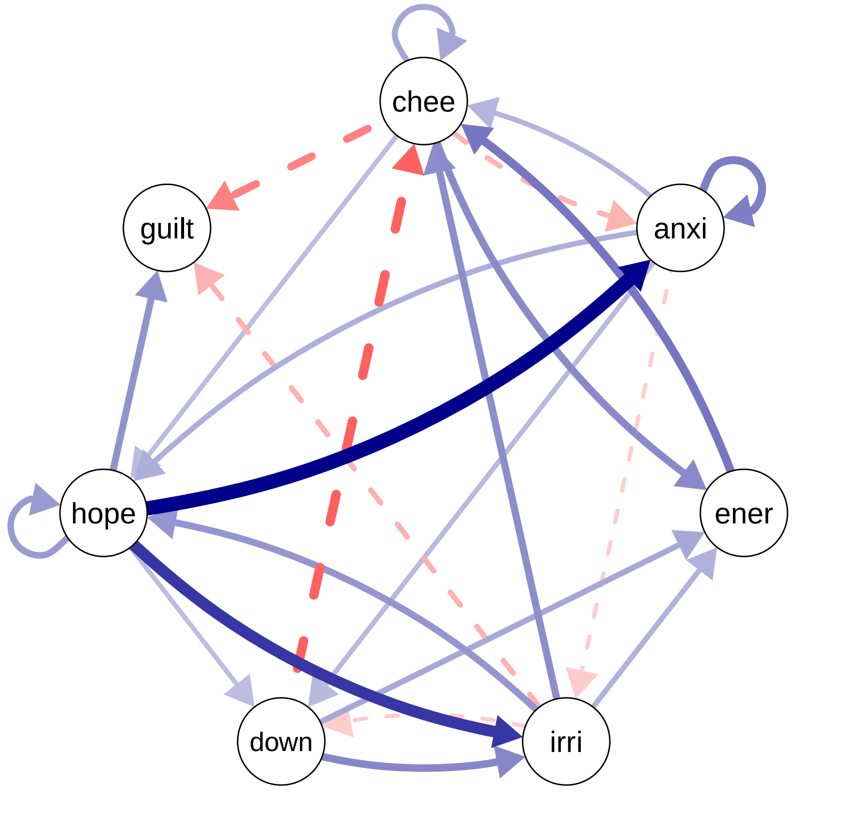

PCT tap ADM non−reg Pt 286 Estpoint 4

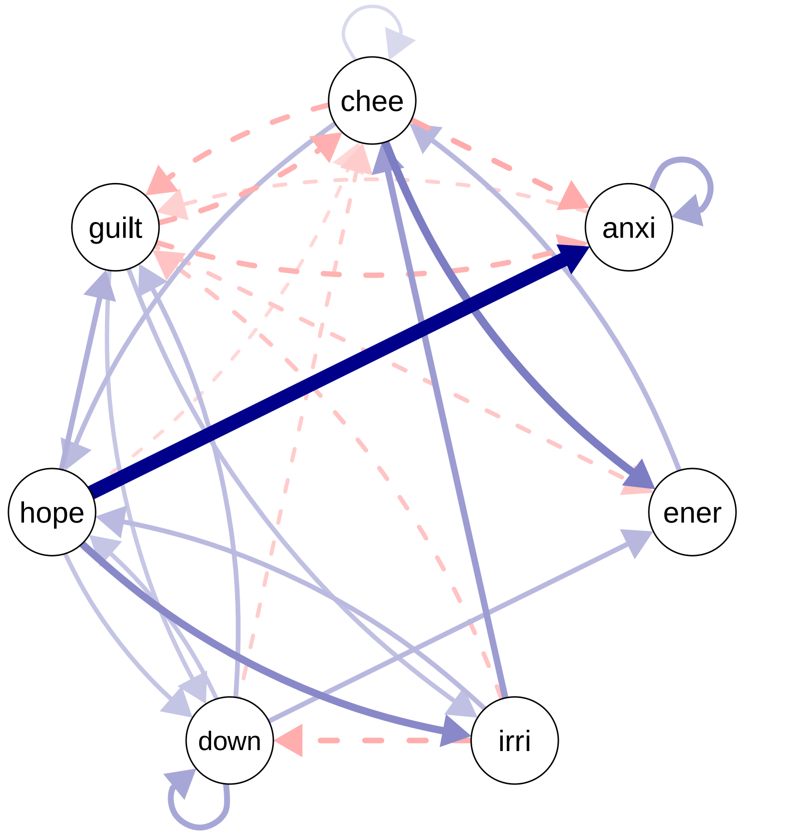

PCT tap ADM non−reg Pt 286 Estpoint 5

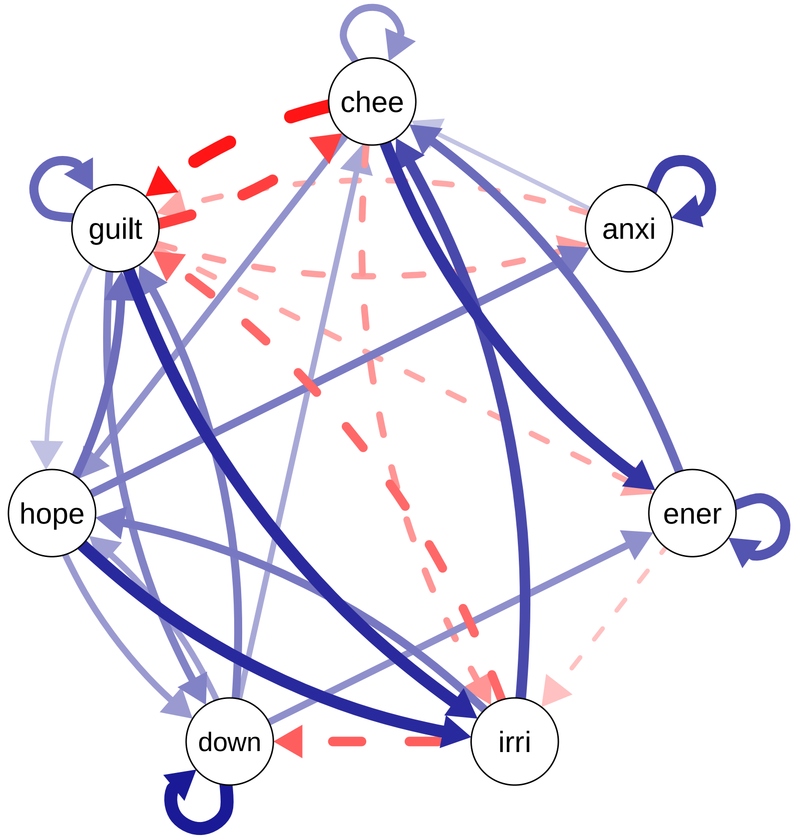

PCT tap ADM non−reg Pt 286 Estpoint 6

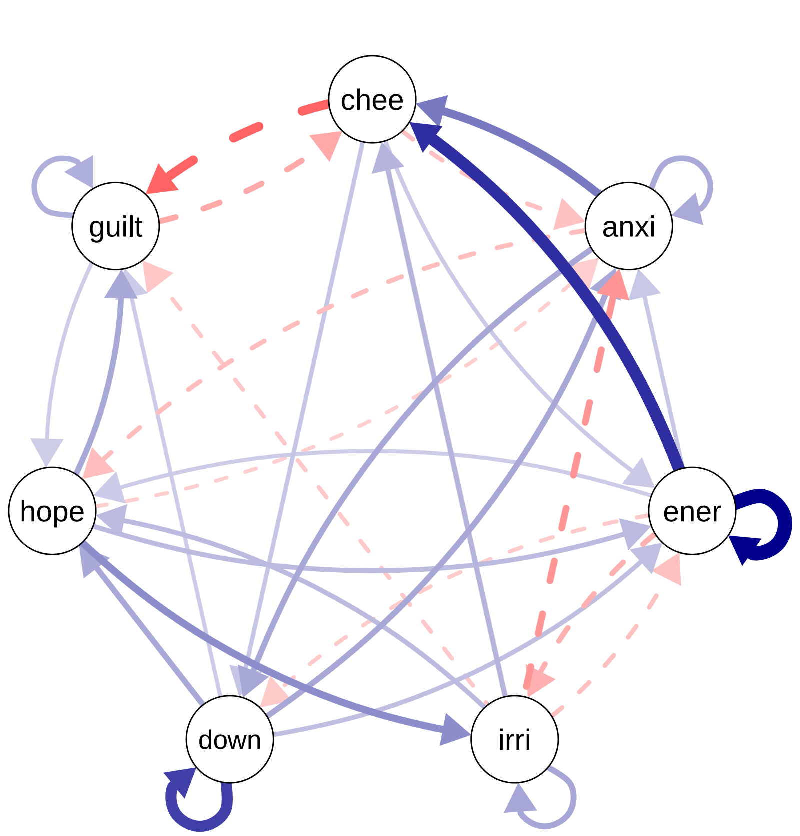

PCT tap ADM non−reg Pt 286 Estpoint 7

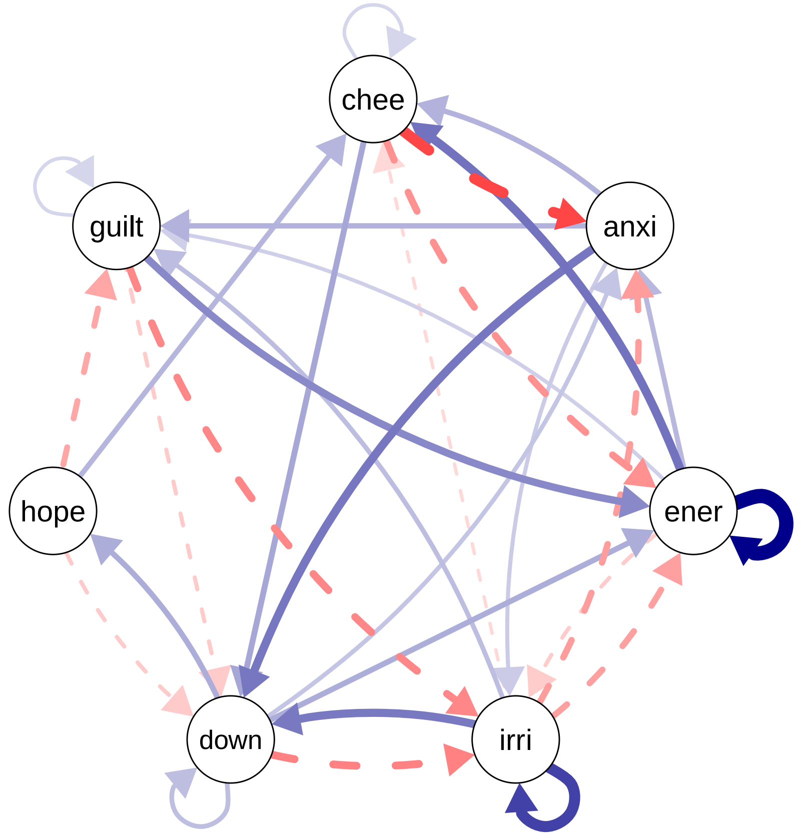

PCT tap ADM non−reg Pt 286 Estpoint 8

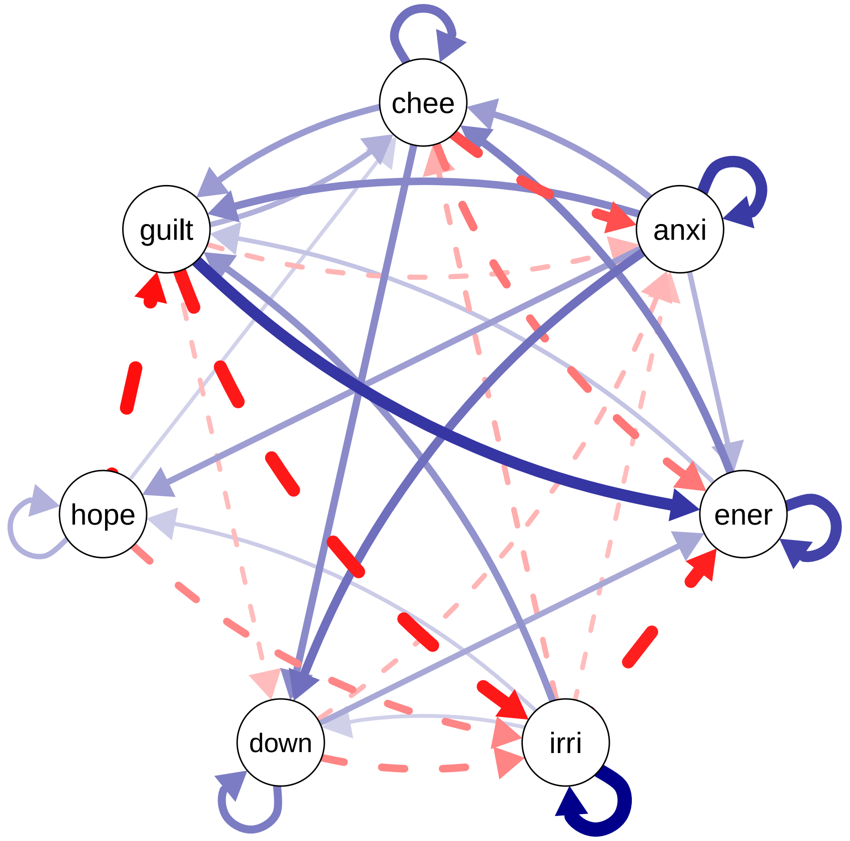

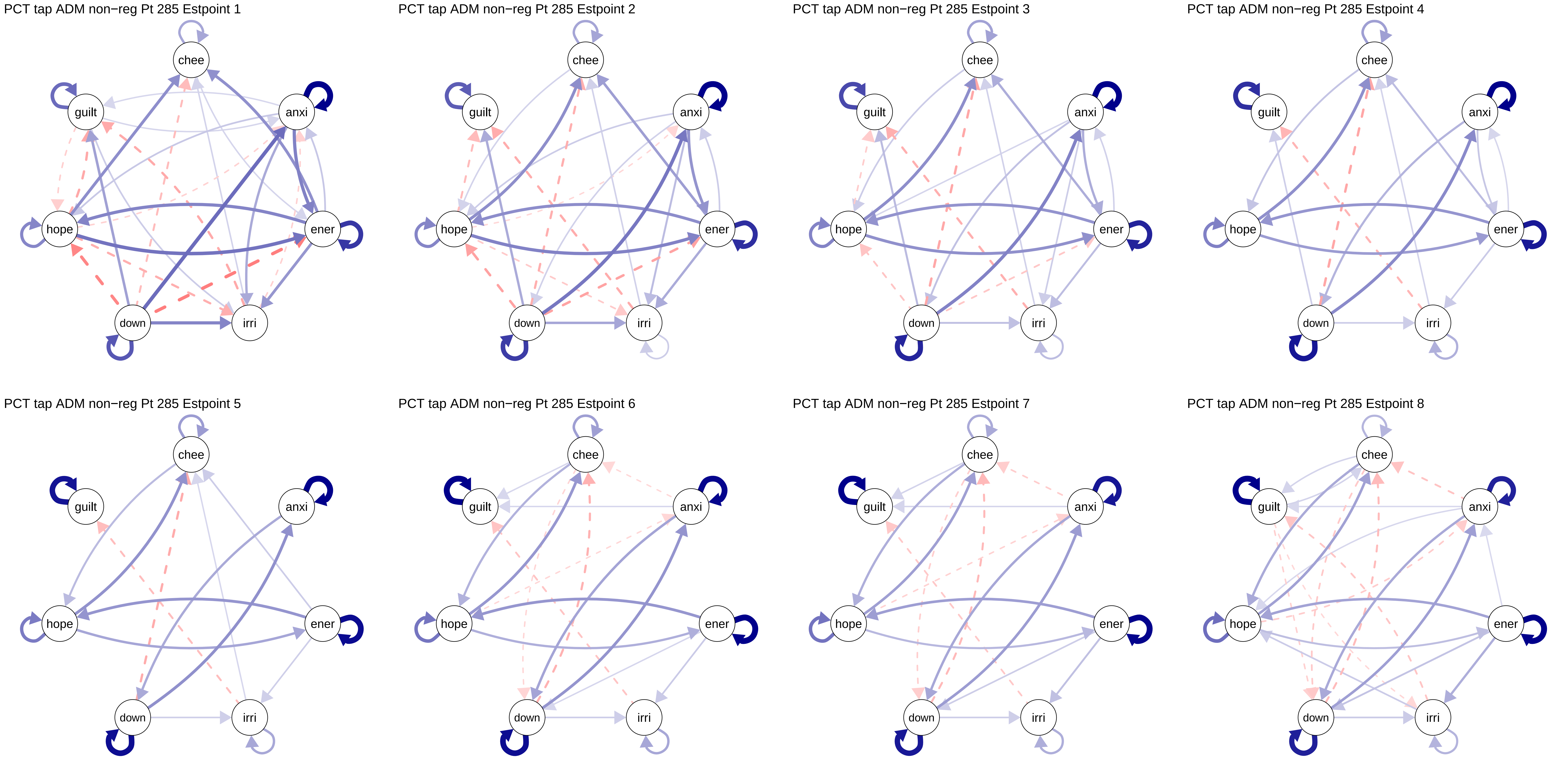
PCT tap ADM non−reg Pt 285 Estpoint 1
PCT tap ADM non−reg Pt 285 Estpoint 2
PCT tap ADM non−reg Pt 285 Estpoint 3
PCT tap ADM non−reg Pt 285 Estpoint 4
PCT tap ADM non−reg Pt 285 Estpoint 5
PCT tap ADM non−reg Pt 285 Estpoint 6
PCT tap ADM non−reg Pt 285 Estpoint 7
PCT tap ADM non−reg Pt 285 Estpoint 8
chee
guilt
anxi
hope
ener
down
irri

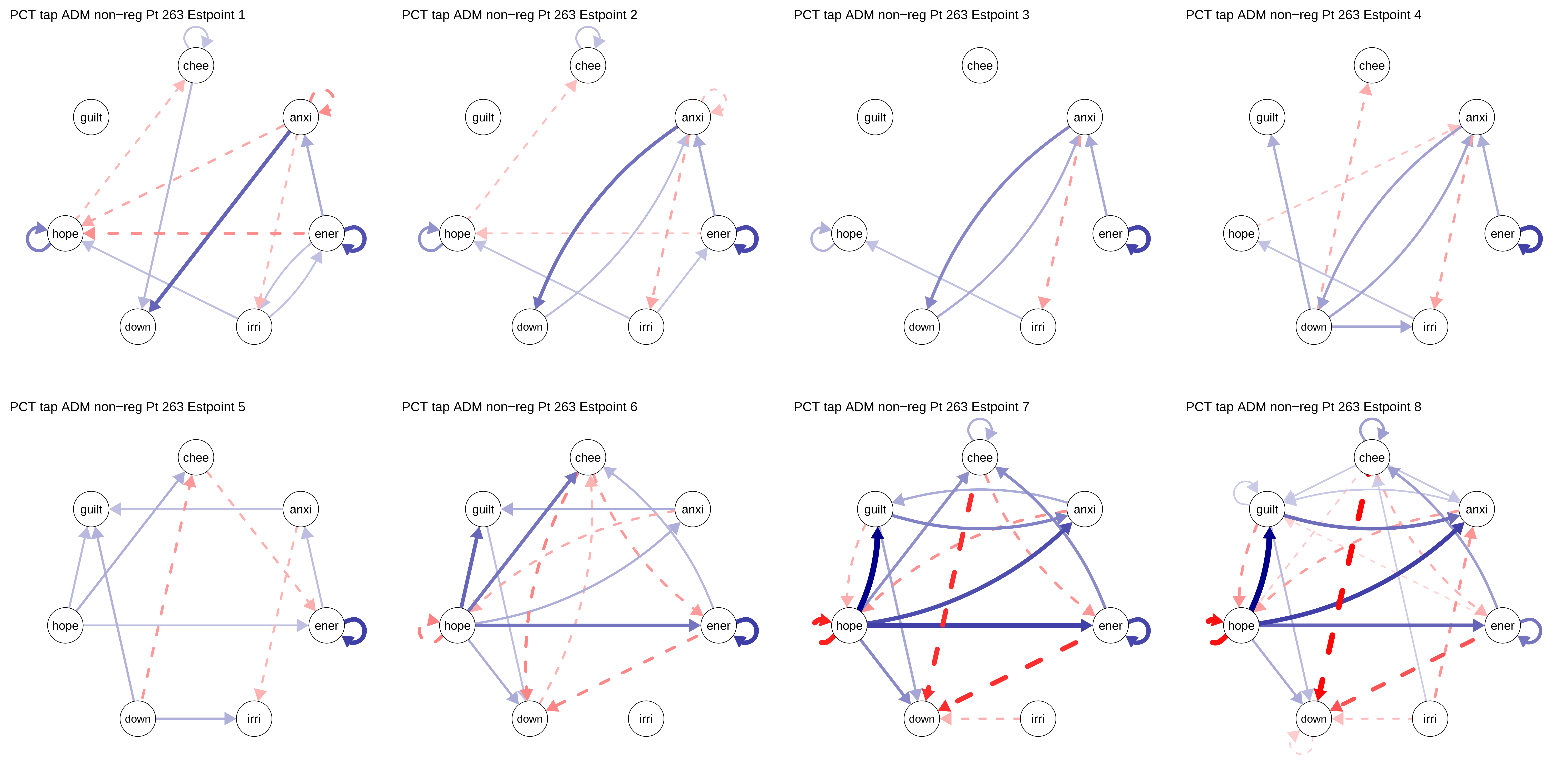
PCT tap ADM non−reg Pt 263 Estpoint 1
chee
guilt
anxi
hope
ener
down
irri
PCT tap ADM non−reg Pt 263 Estpoint 2
chee
guilt
anxi
hope
ener
down
irri
PCT tap ADM non−reg Pt 263 Estpoint 3
chee
guilt
anxi
hope
ener
down
irri
PCT tap ADM non−reg Pt 263 Estpoint 4
chee
guilt
anxi
hope
ener
down
irri
PCT tap ADM non−reg Pt 263 Estpoint 5
chee
guilt
anxi
hope
ener
down
irri
PCT tap ADM non−reg Pt 263 Estpoint 6
chee
guilt
anxi
hope
ener
down
irri
PCT tap ADM non−reg Pt 263 Estpoint 7
chee
guilt
anxi
hope
ener
down
irri
PCT tap ADM non−reg Pt 263 Estpoint 8
chee
guilt
anxi
hope
ener
down
irri

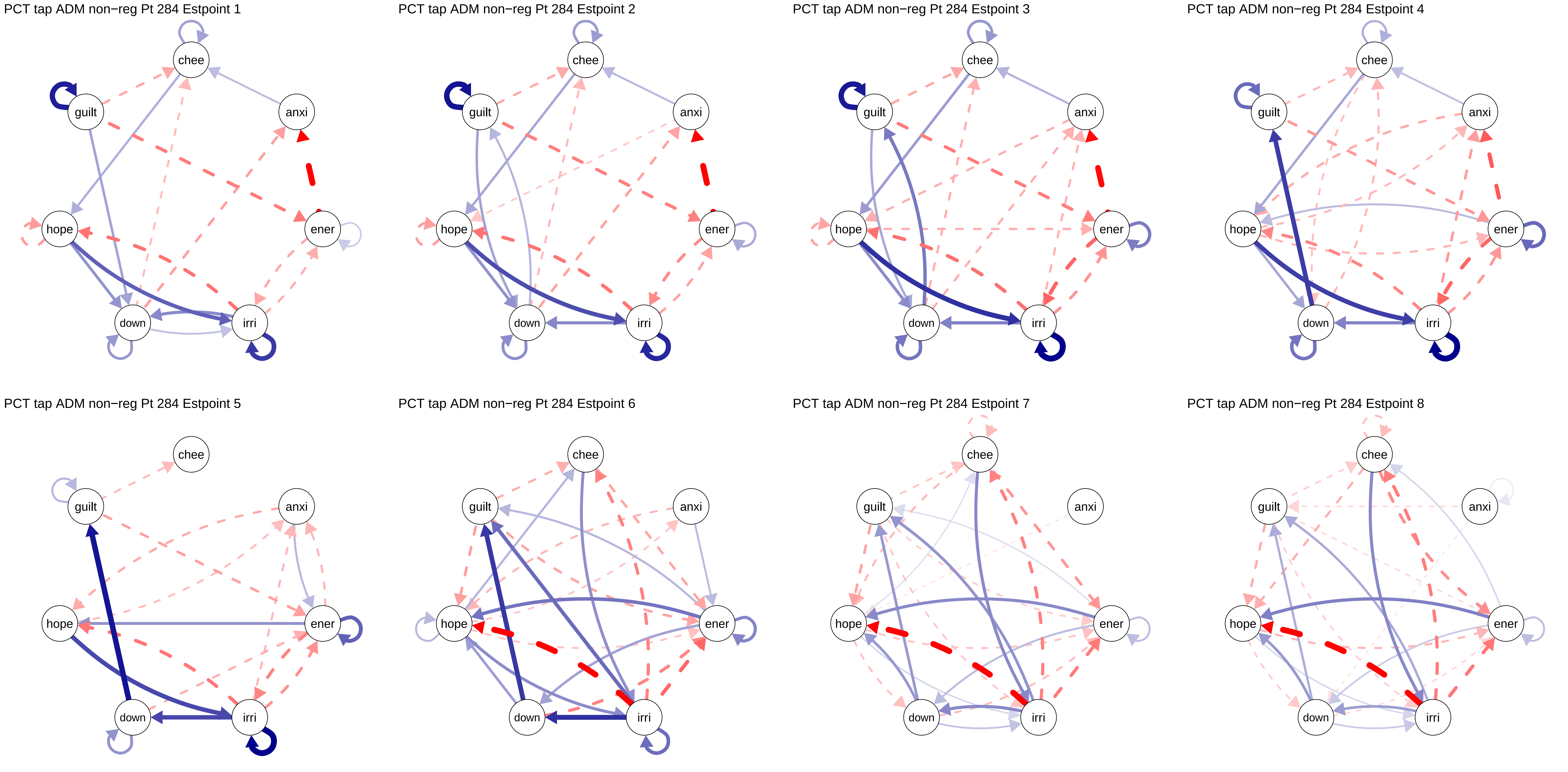
PCT tap ADM non−reg Pt 284 Estpoint 1
PCT tap ADM non−reg Pt 284 Estpoint 2
PCT tap ADM non−reg Pt 284 Estpoint 3
PCT tap ADM non−reg Pt 284 Estpoint 4
PCT tap ADM non−reg Pt 284 Estpoint 5
PCT tap ADM non−reg Pt 284 Estpoint 6
PCT tap ADM non−reg Pt 284 Estpoint 7
PCT tap ADM non−reg Pt 284 Estpoint 8
chee
guilt
anxi
hope
ener
down
irri

PCT tap ADM non−reg Pt 68 Estpoint 1

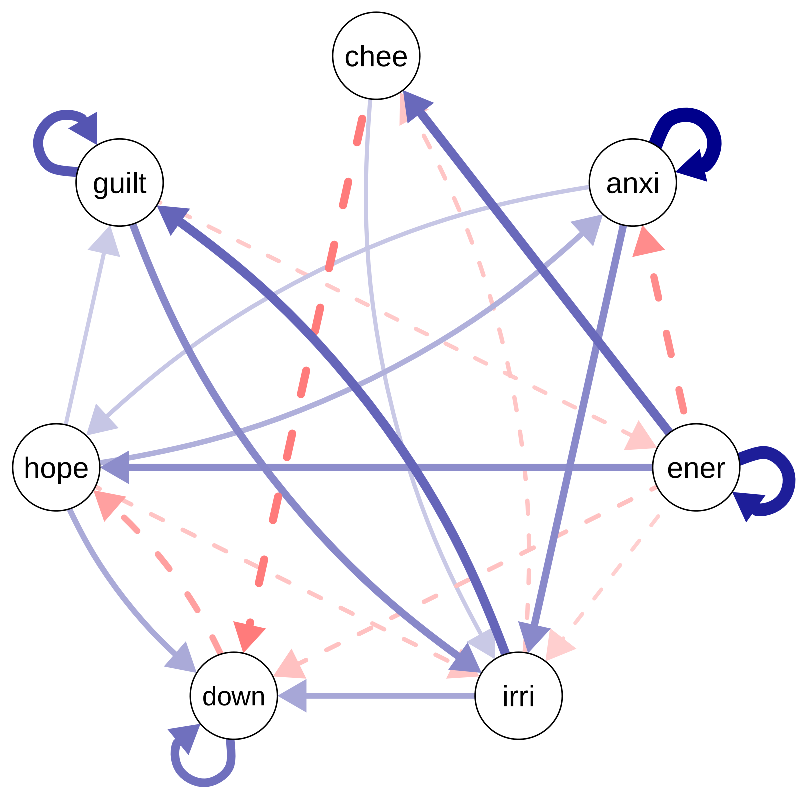

PCT tap ADM non−reg Pt 68 Estpoint 2

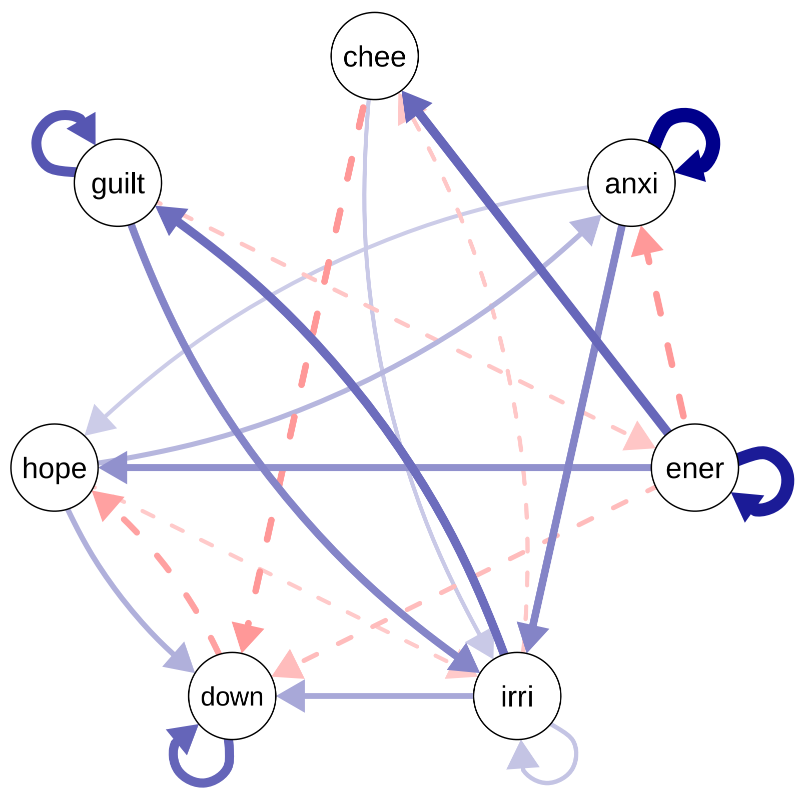

PCT tap ADM non−reg Pt 68 Estpoint 3

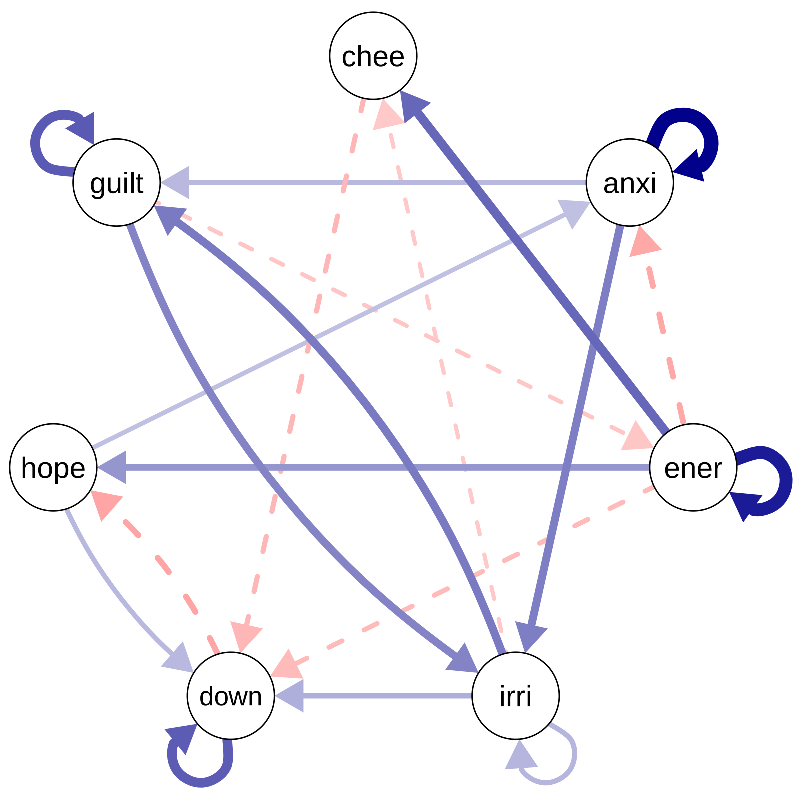

PCT tap ADM non−reg Pt 68 Estpoint 4

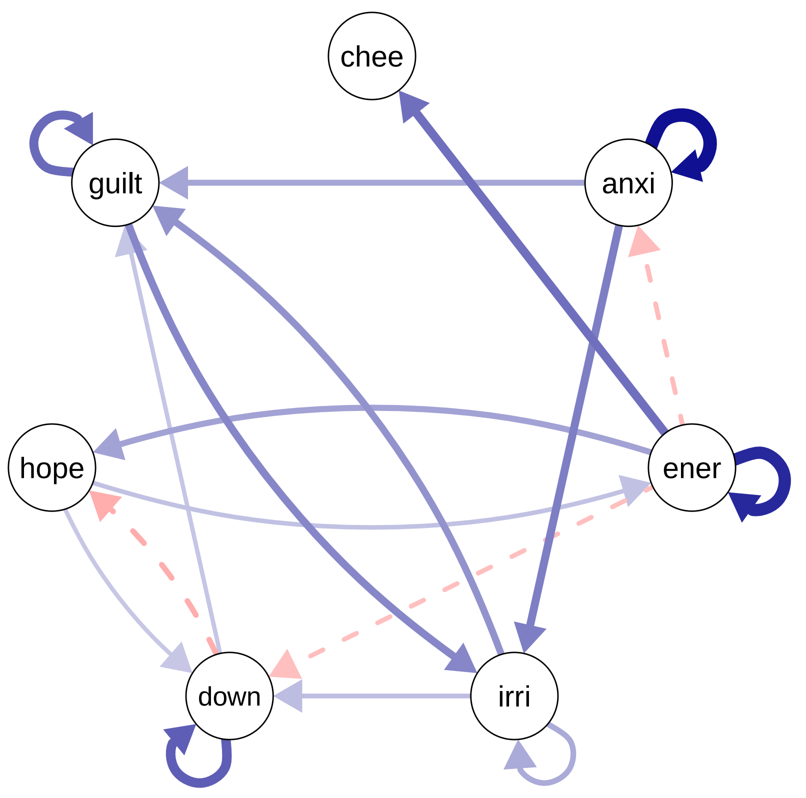

PCT tap ADM non−reg Pt 68 Estpoint 5

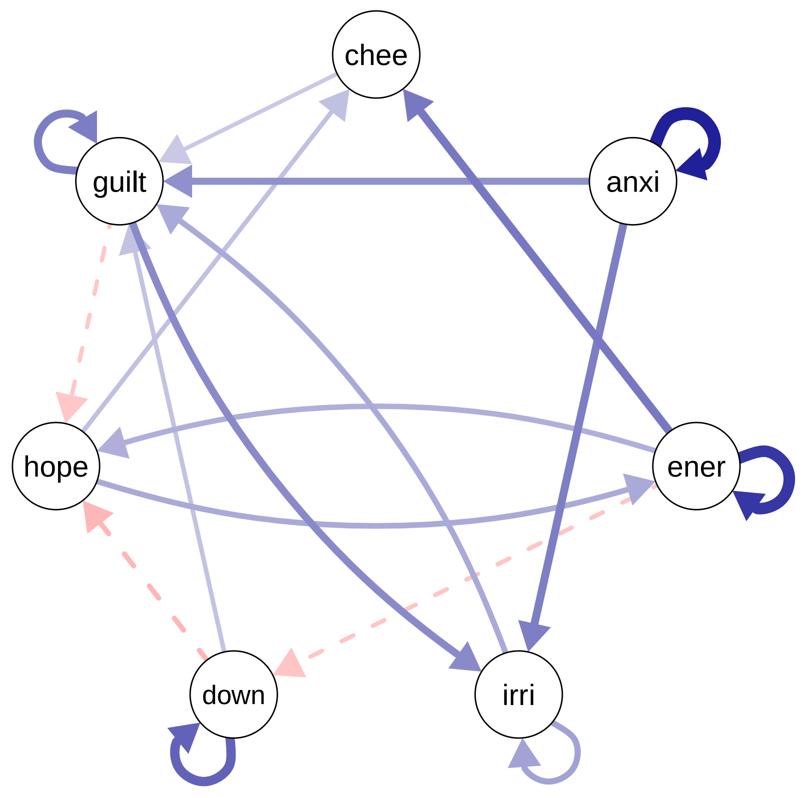

PCT tap ADM non−reg Pt 68 Estpoint 6

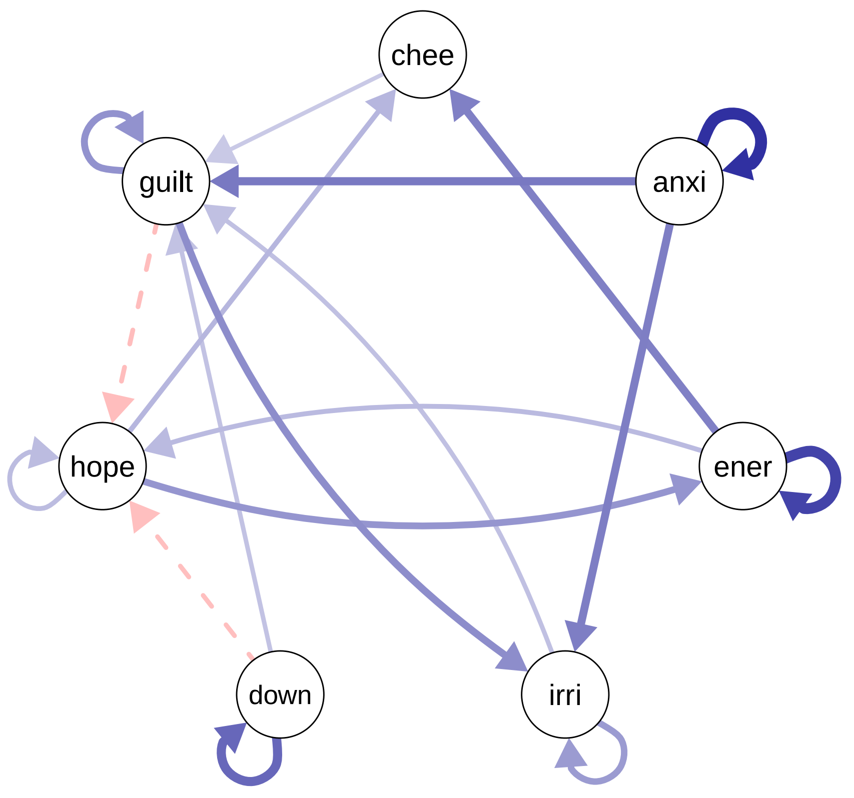

PCT tap ADM non−reg Pt 68 Estpoint 7

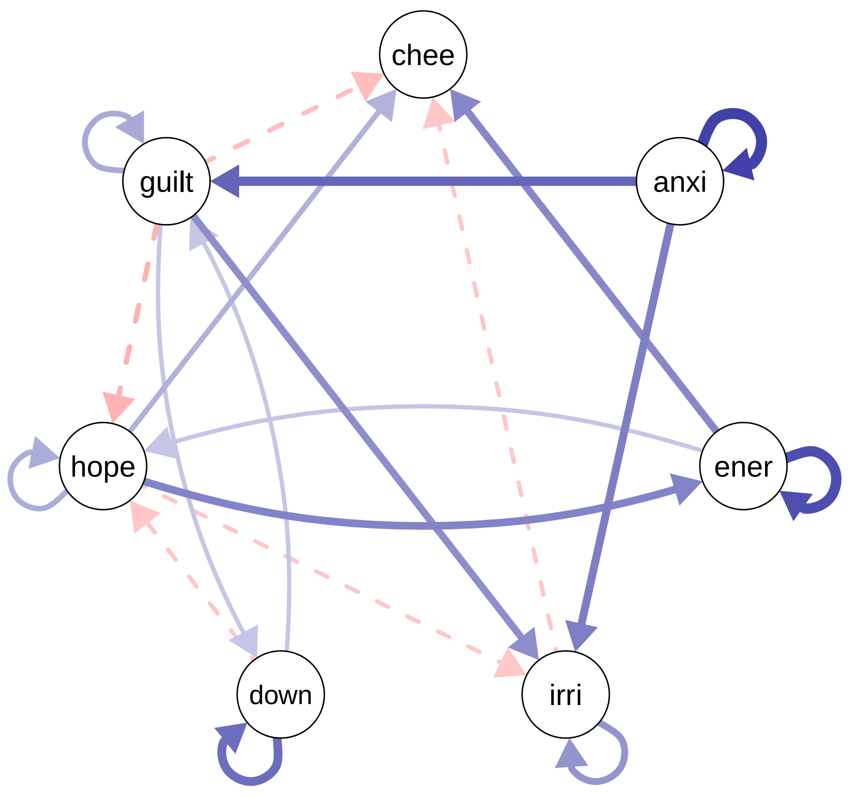

PCT tap ADM non−reg Pt 68 Estpoint 8

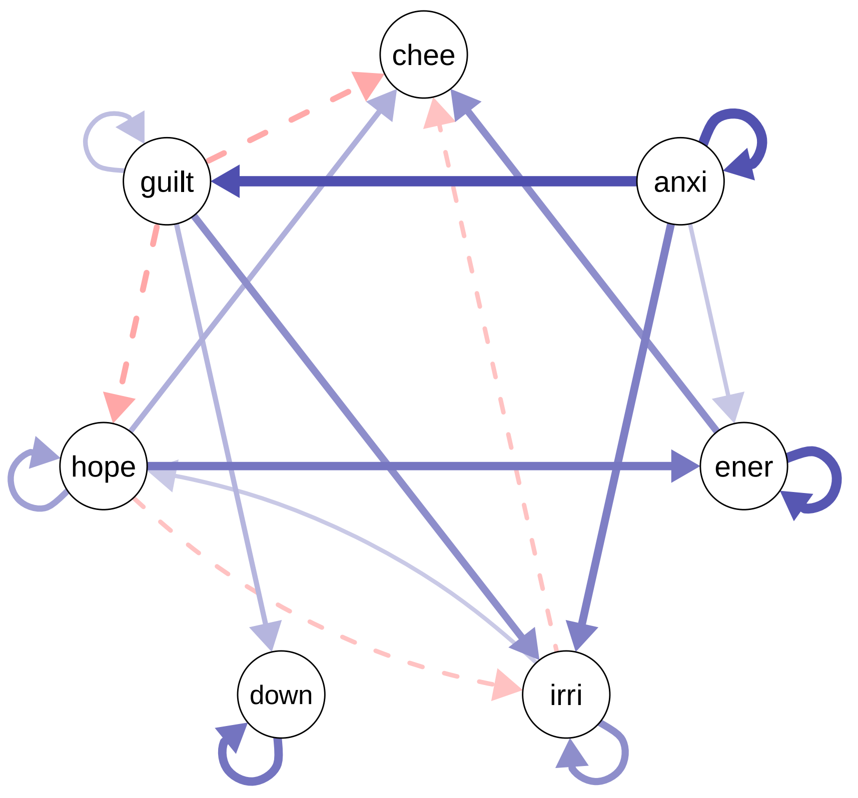

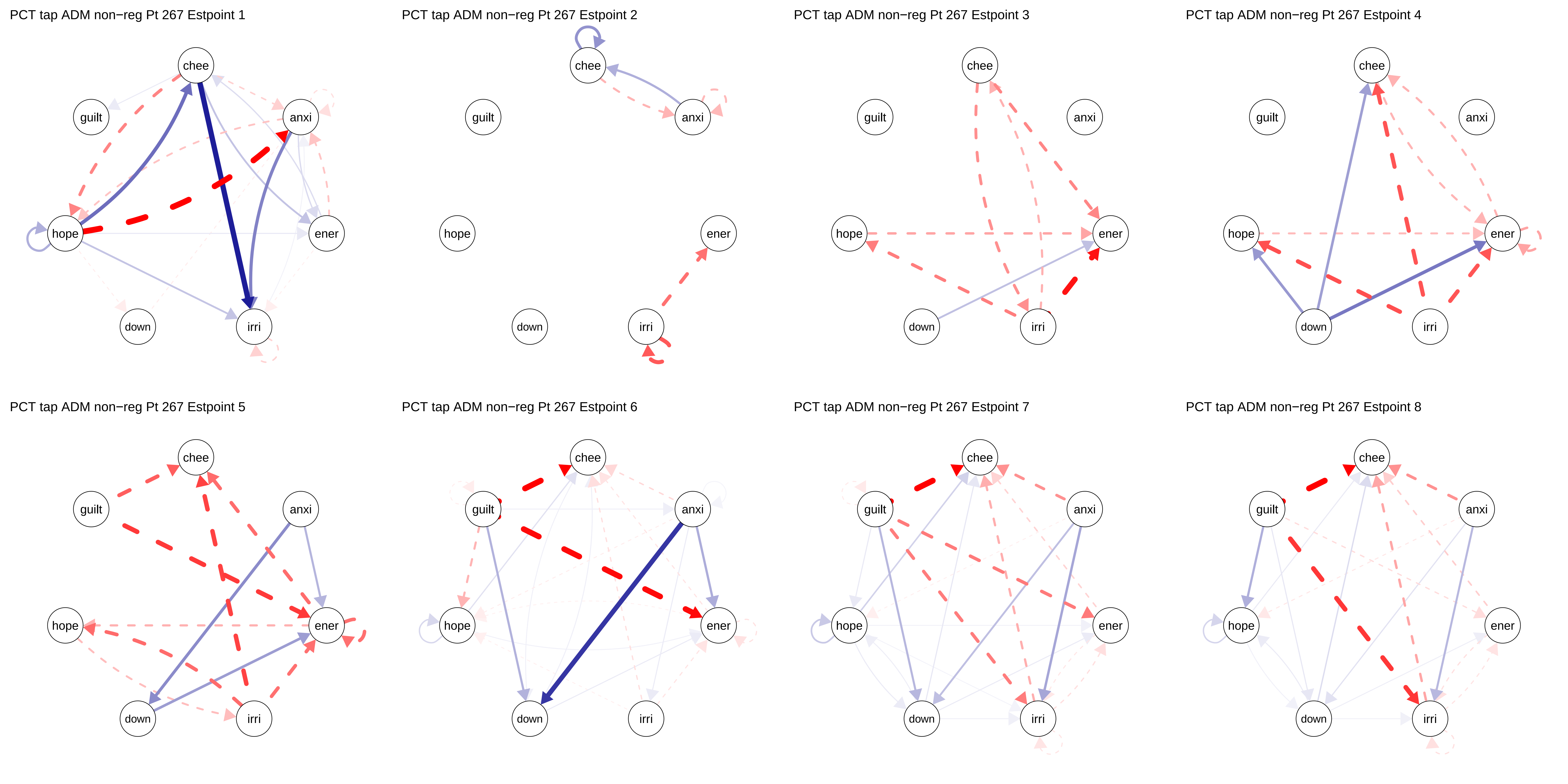
PCT tap ADM non−reg Pt 267 Estpoint 1
chee
guilt
anxi
hope
ener
down
irri
PCT tap ADM non−reg Pt 267 Estpoint 2
chee
guilt
anxi
hope
ener
down
irri
PCT tap ADM non−reg Pt 267 Estpoint 3
chee
guilt
anxi
hope
ener
down
irri
PCT tap ADM non−reg Pt 267 Estpoint 4
chee
guilt
anxi
hope
ener
down
irri
PCT tap ADM non−reg Pt 267 Estpoint 5
chee
guilt
anxi
hope
ener
down
irri
PCT tap ADM non−reg Pt 267 Estpoint 6
chee
guilt
anxi
hope
ener
down
irri
PCT tap ADM non−reg Pt 267 Estpoint 7
chee
guilt
anxi
hope
ener
down
irri
PCT tap ADM non−reg Pt 267 Estpoint 8
chee
guilt
anxi
hope
ener
down
irri

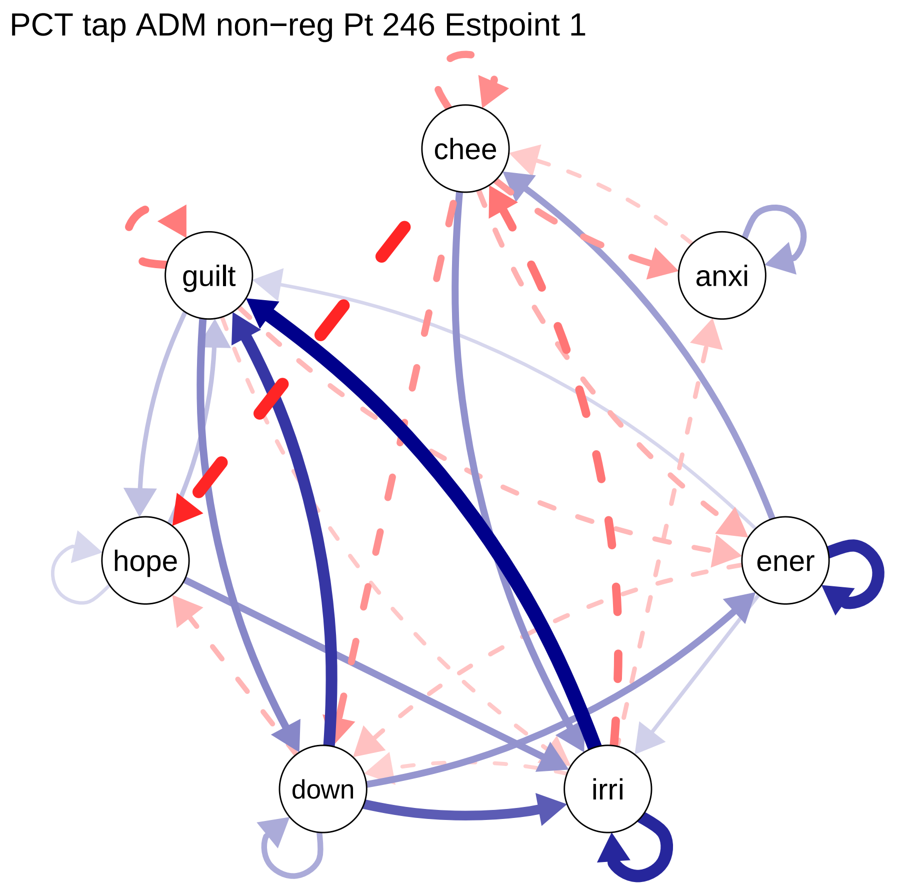
PCT tap ADM non−reg Pt 246 Estpoint 1
chee
guilt
anxi
hope
ener
down
irri

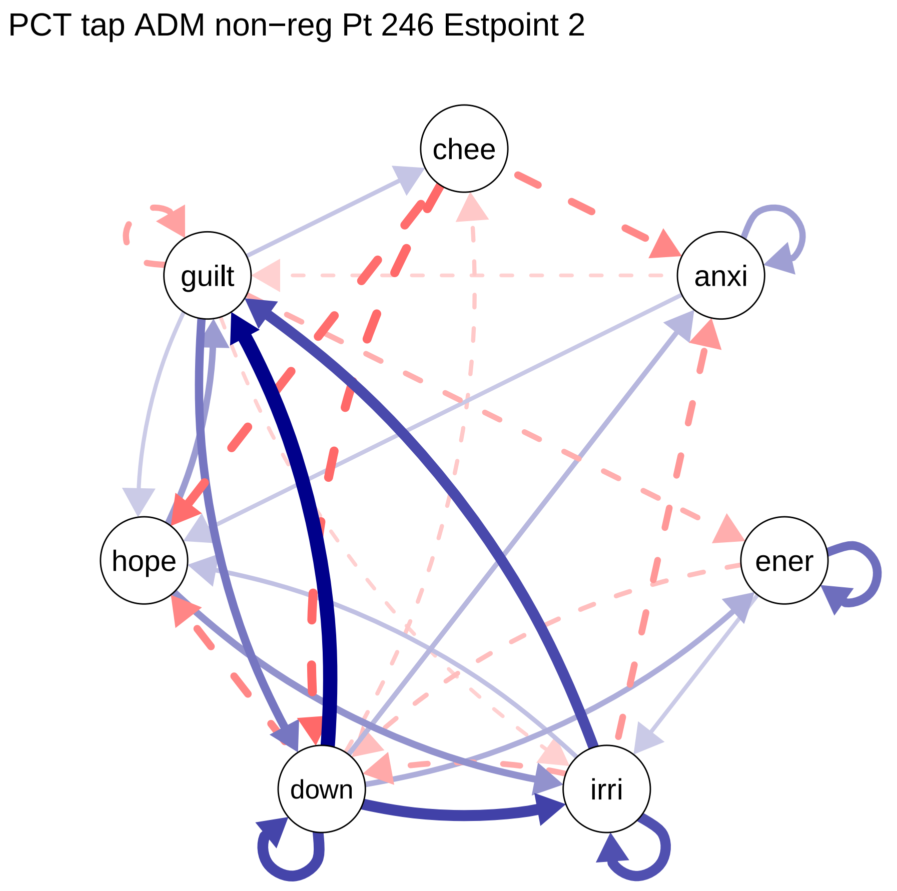
PCT tap ADM non−reg Pt 246 Estpoint 2
chee
guilt
anxi
hope
ener
down
irri

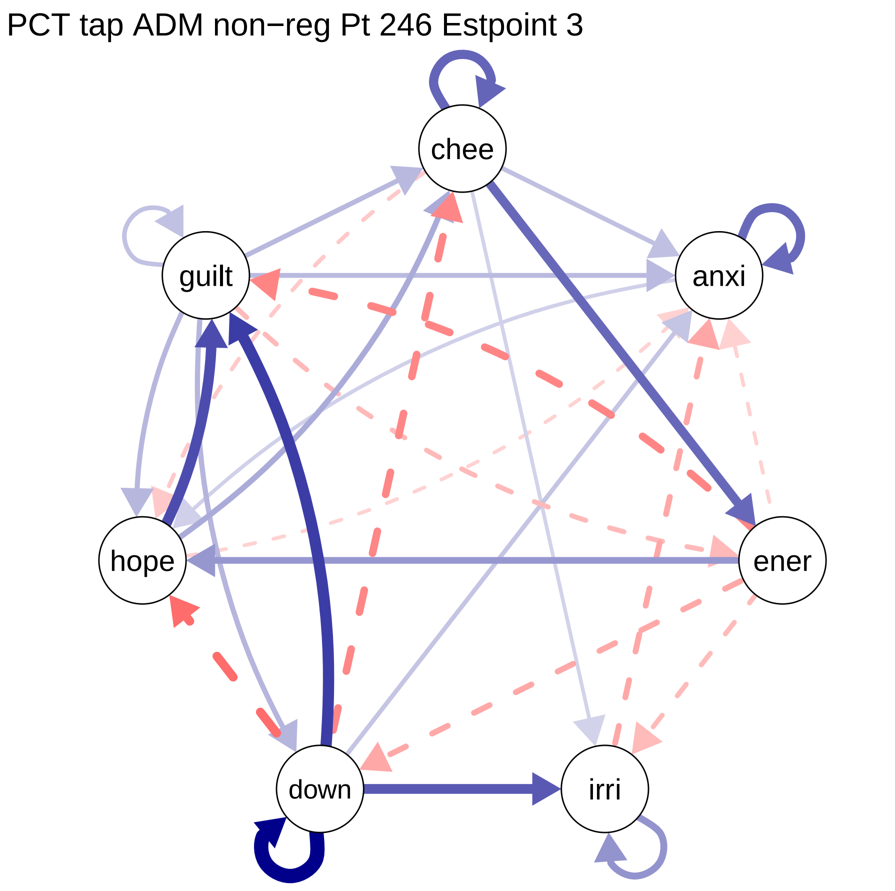
PCT tap ADM non−reg Pt 246 Estpoint 3
chee
guilt
anxi
hope
ener
down
irri

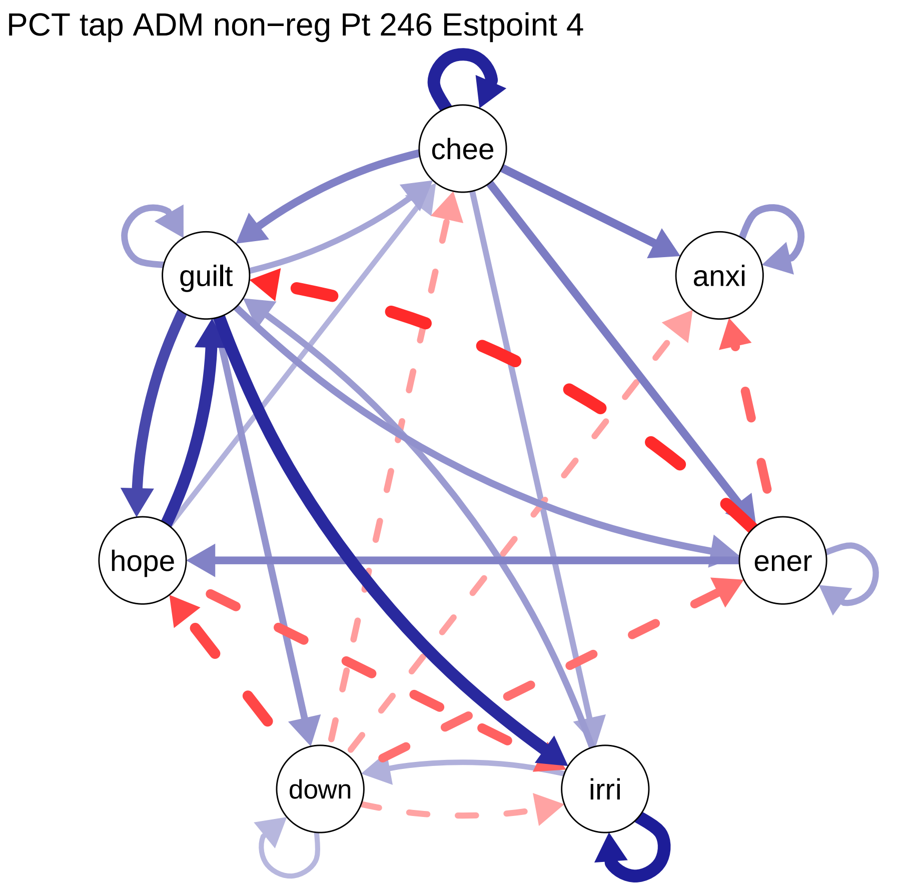
PCT tap ADM non−reg Pt 246 Estpoint 4
chee
guilt
anxi
hope
ener
down
irri

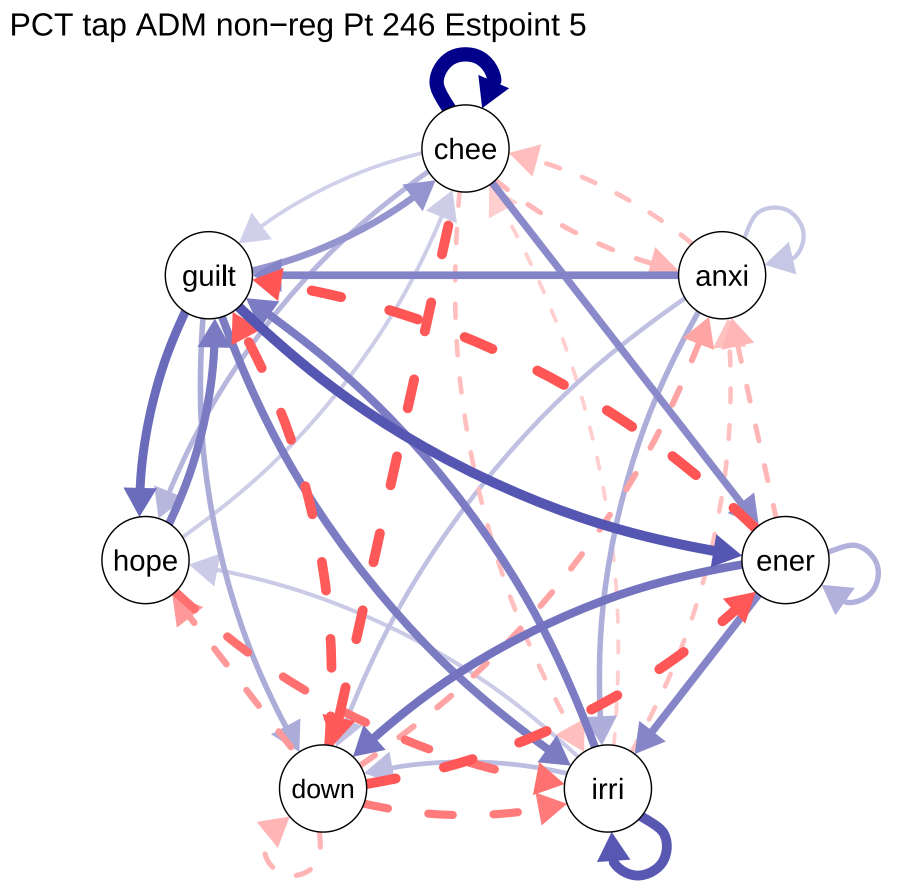
PCT tap ADM non−reg Pt 246 Estpoint 5
chee
guilt
anxi
hope
ener
down
irri

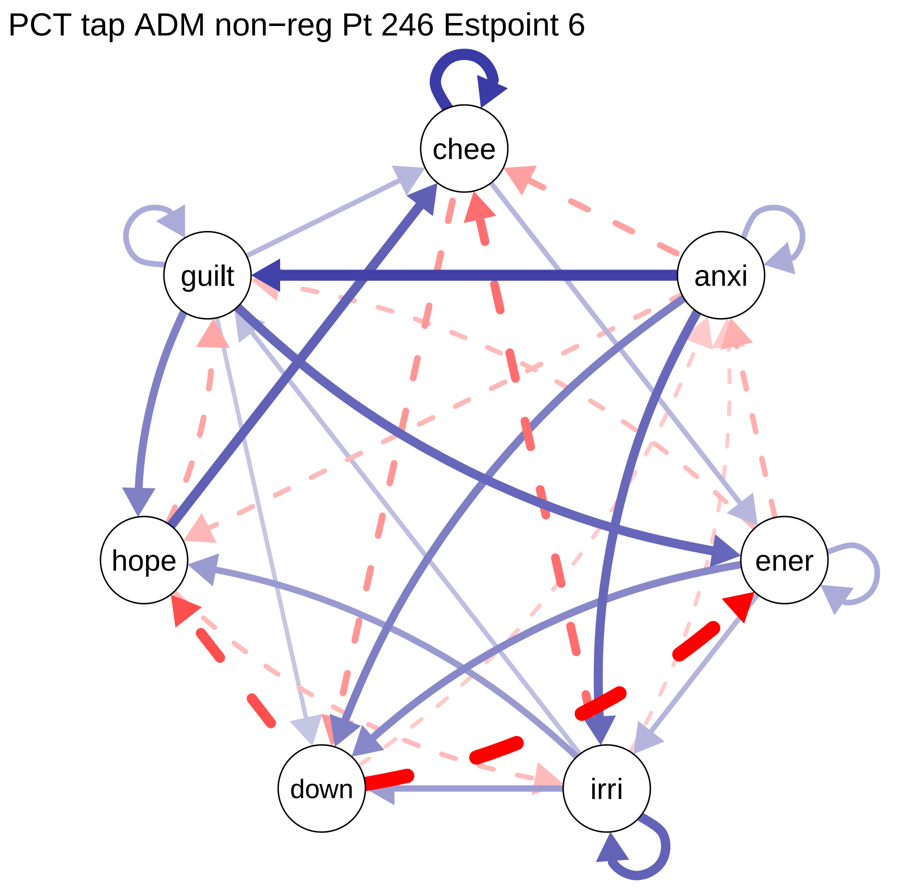
PCT tap ADM non−reg Pt 246 Estpoint 6
chee
guilt
anxi
hope
ener
down
irri

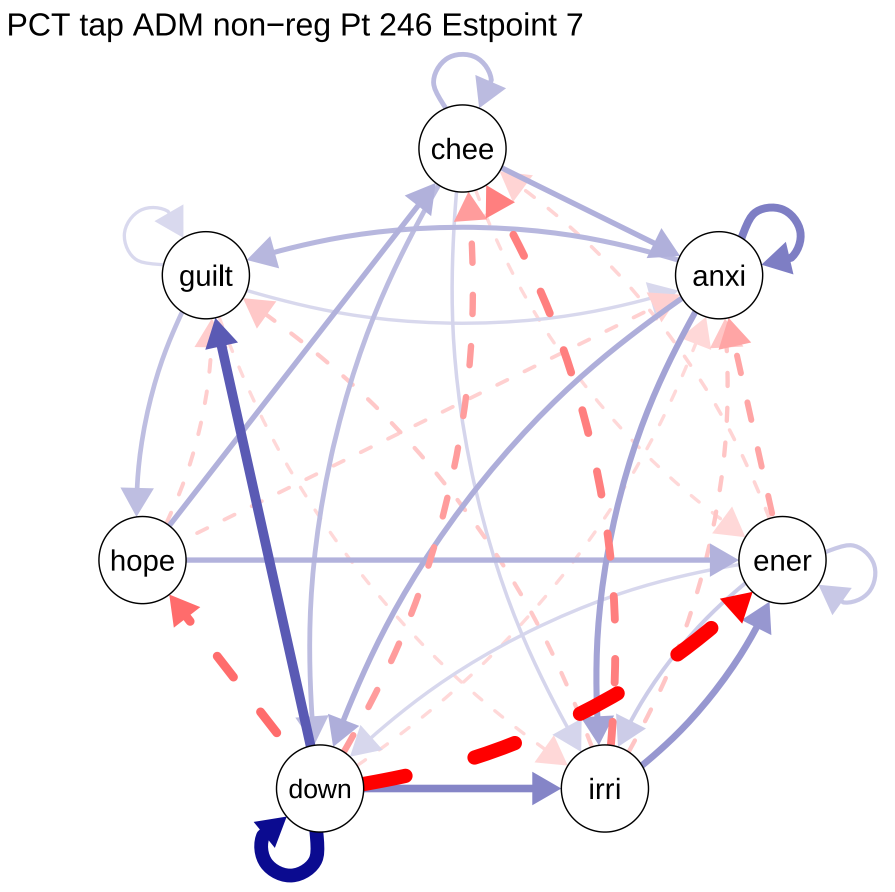
PCT tap ADM non−reg Pt 246 Estpoint 7
chee
guilt
anxi
hope
ener
down
irri

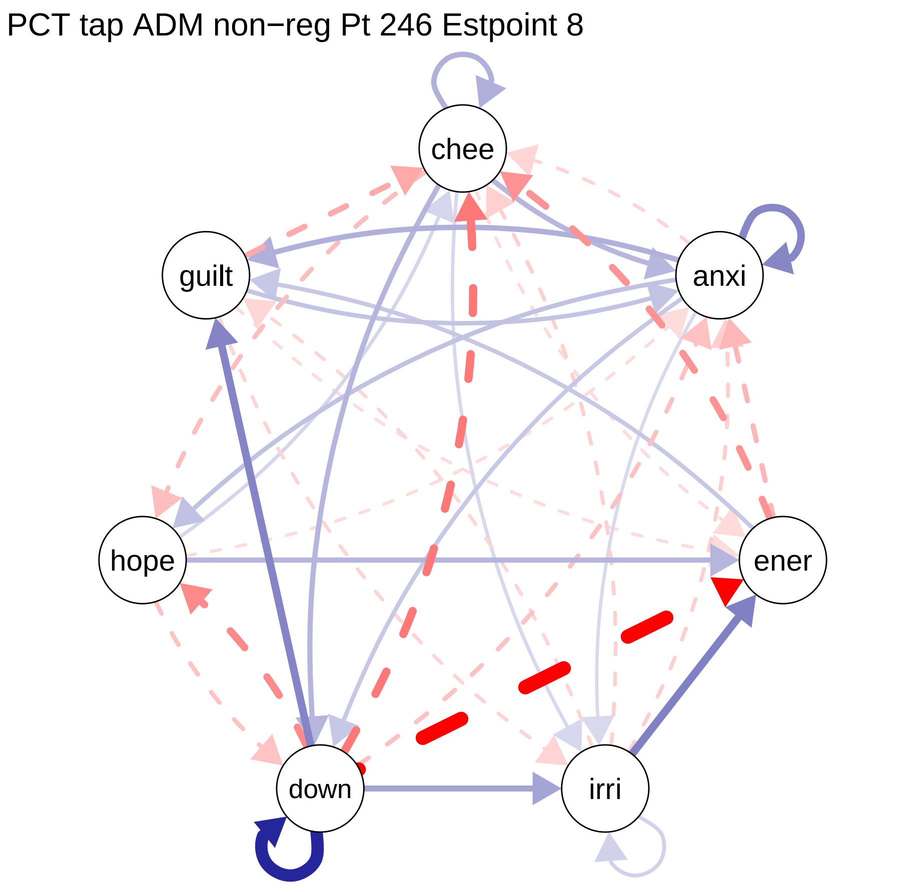
PCT tap ADM non−reg Pt 246 Estpoint 8
chee
guilt
anxi
hope
ener
down
irri

PCT tap ADM non−reg Pt 272 Estpoint 1

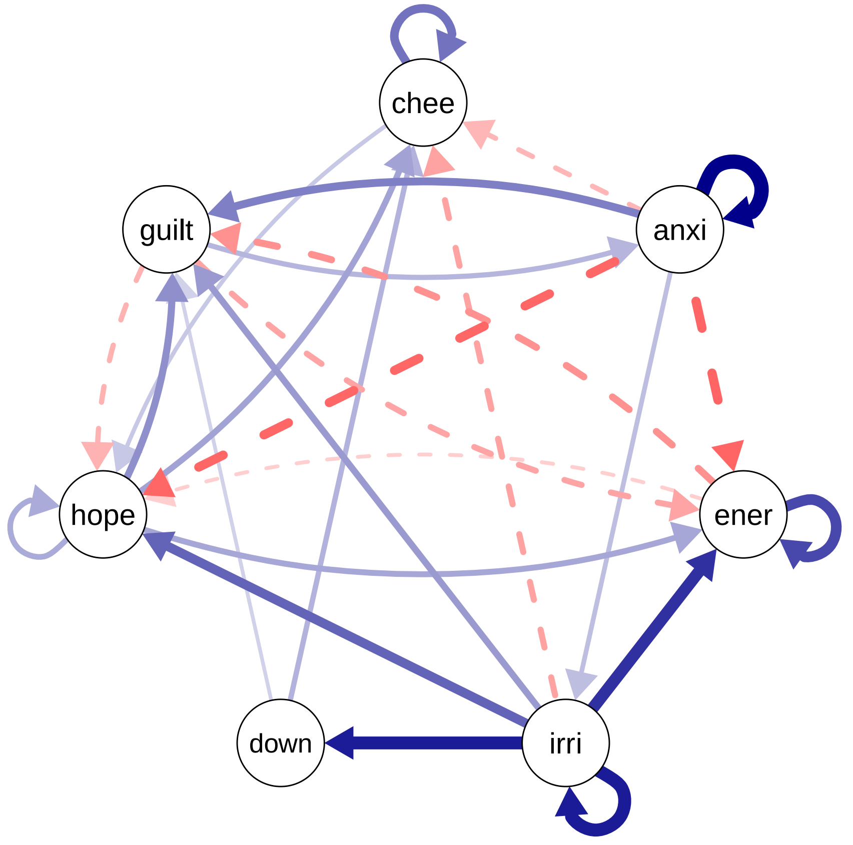

PCT tap ADM non−reg Pt 272 Estpoint 2

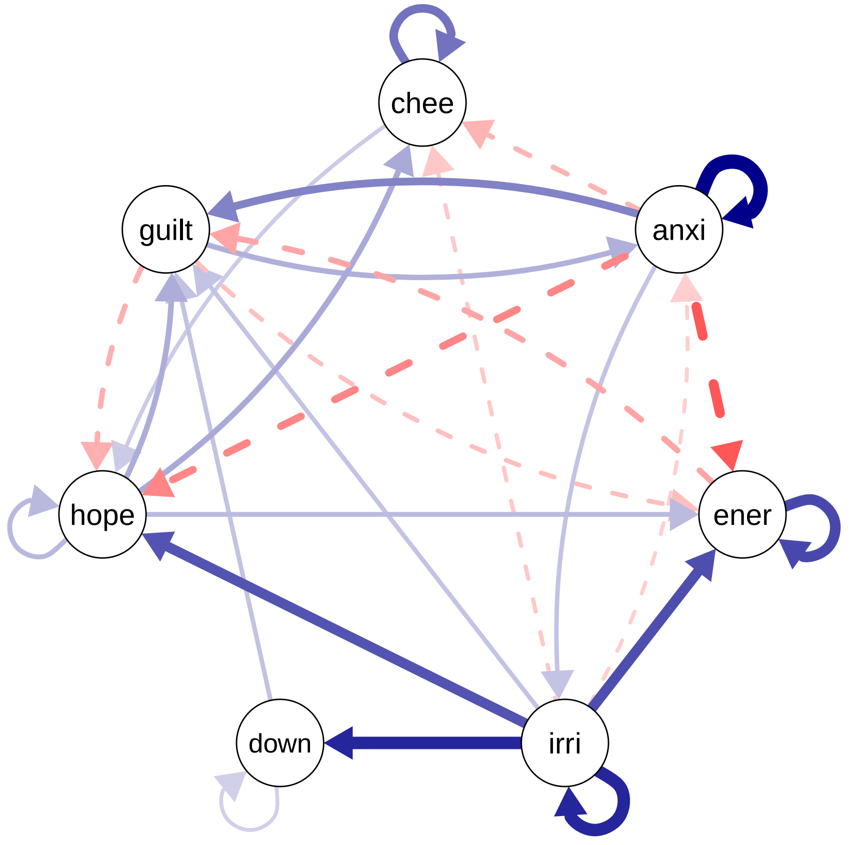

PCT tap ADM non−reg Pt 272 Estpoint 3

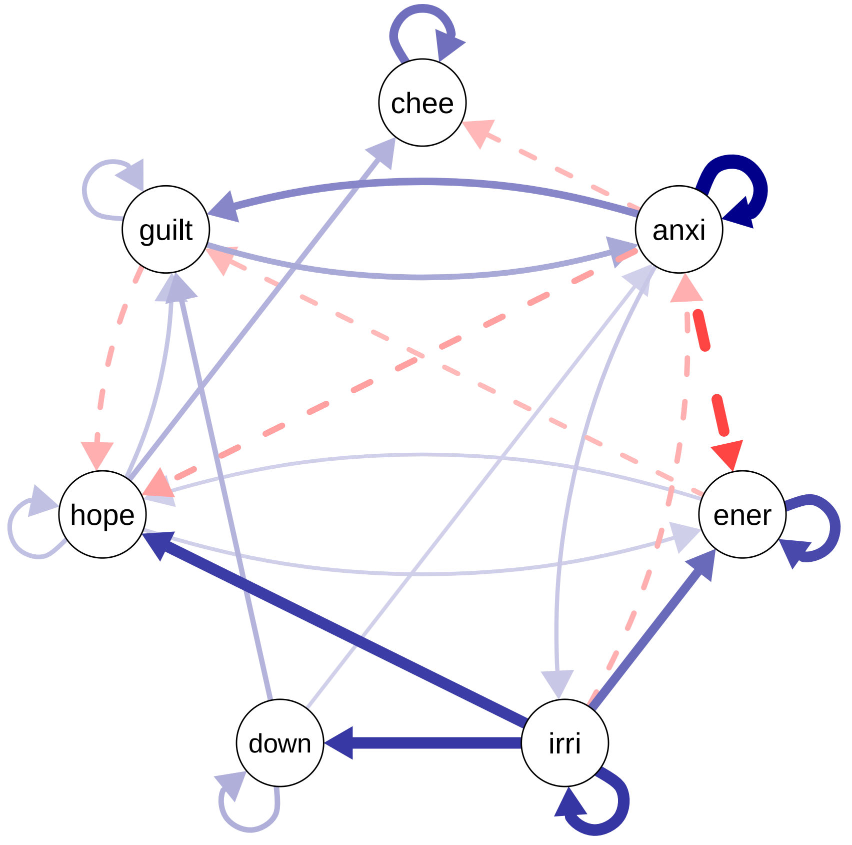

PCT tap ADM non−reg Pt 272 Estpoint 4

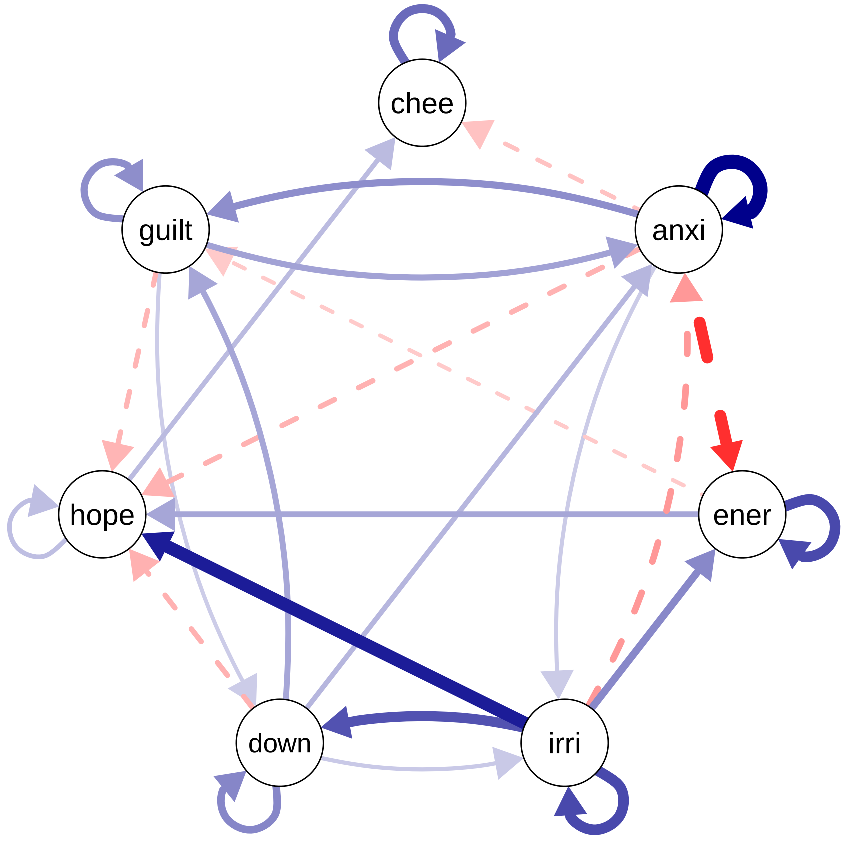

PCT tap ADM non−reg Pt 272 Estpoint 5

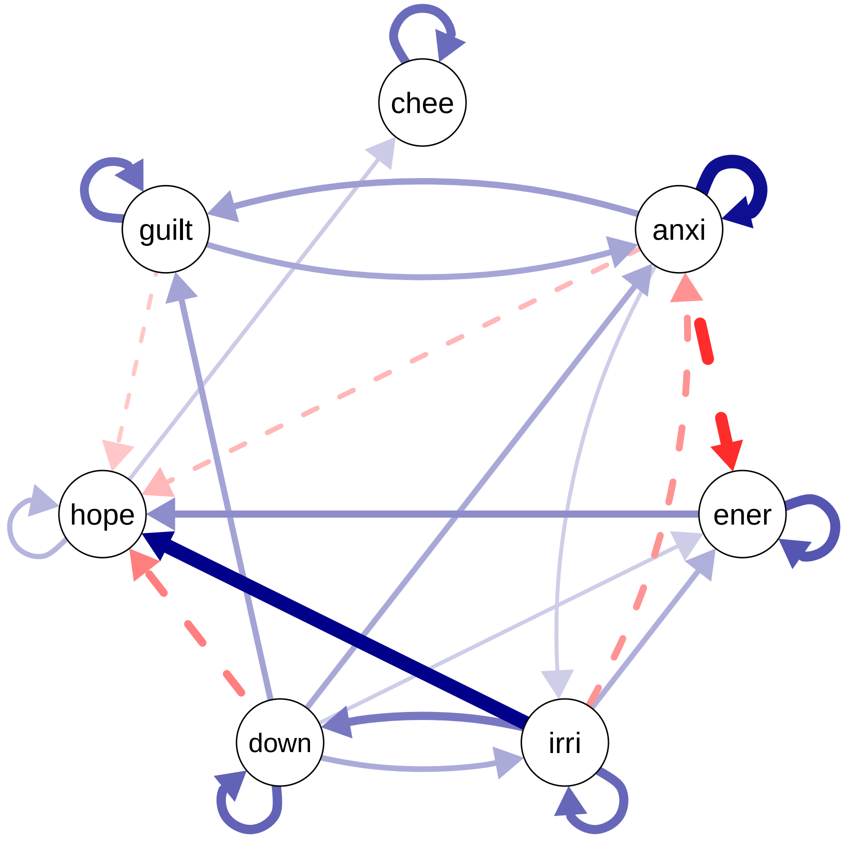

PCT tap ADM non−reg Pt 272 Estpoint 6

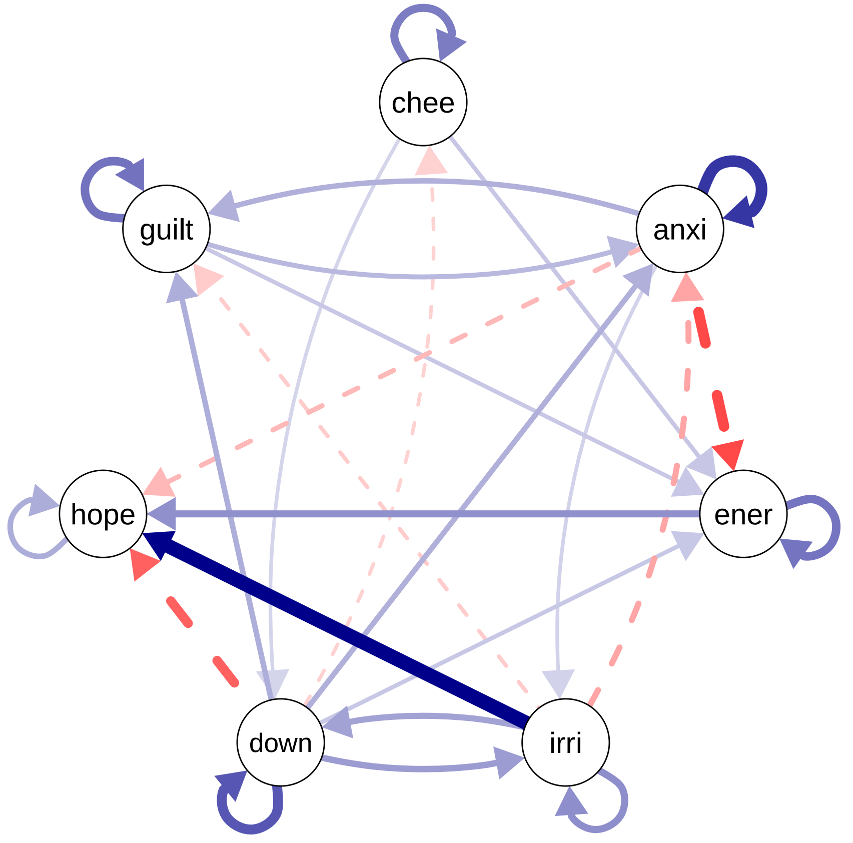

PCT tap ADM non−reg Pt 272 Estpoint 7

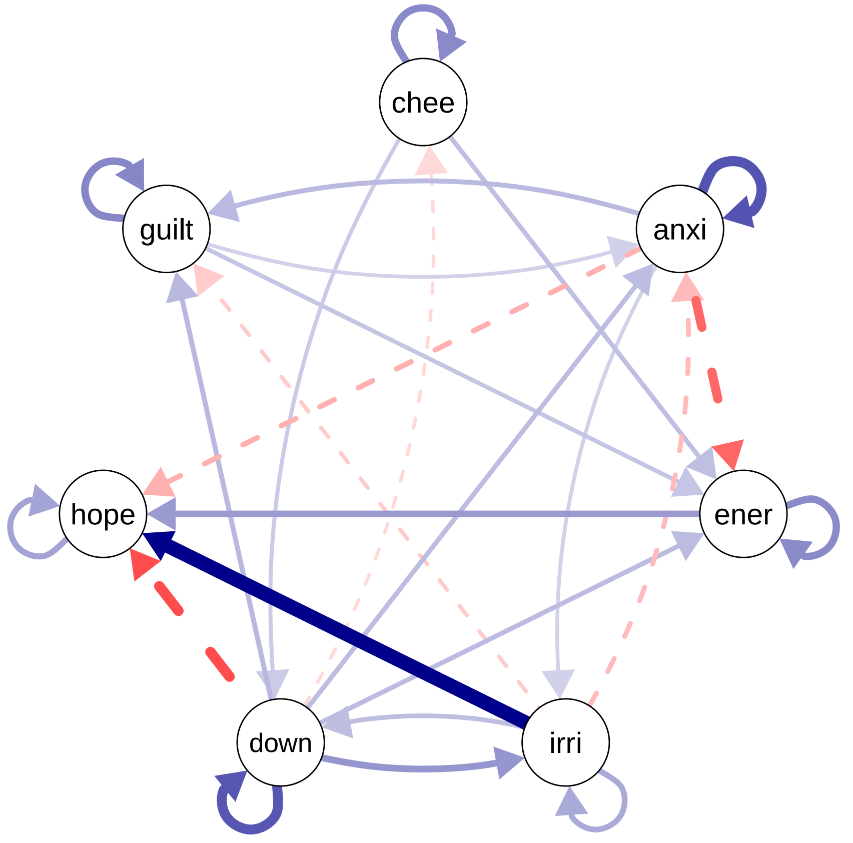

PCT tap ADM non−reg Pt 272 Estpoint 8

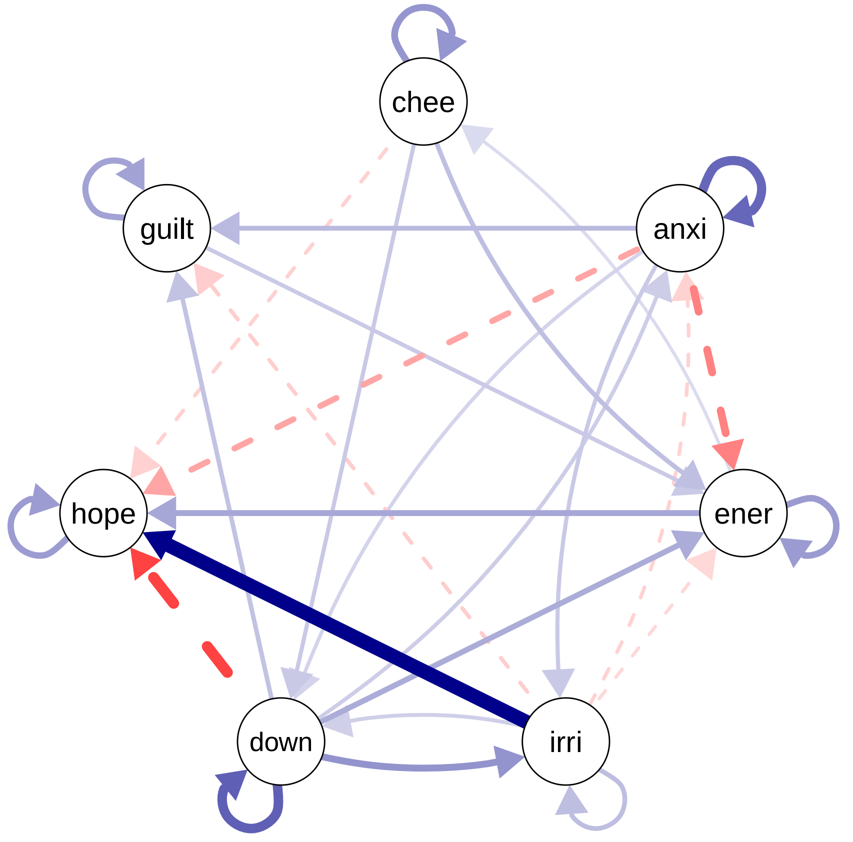

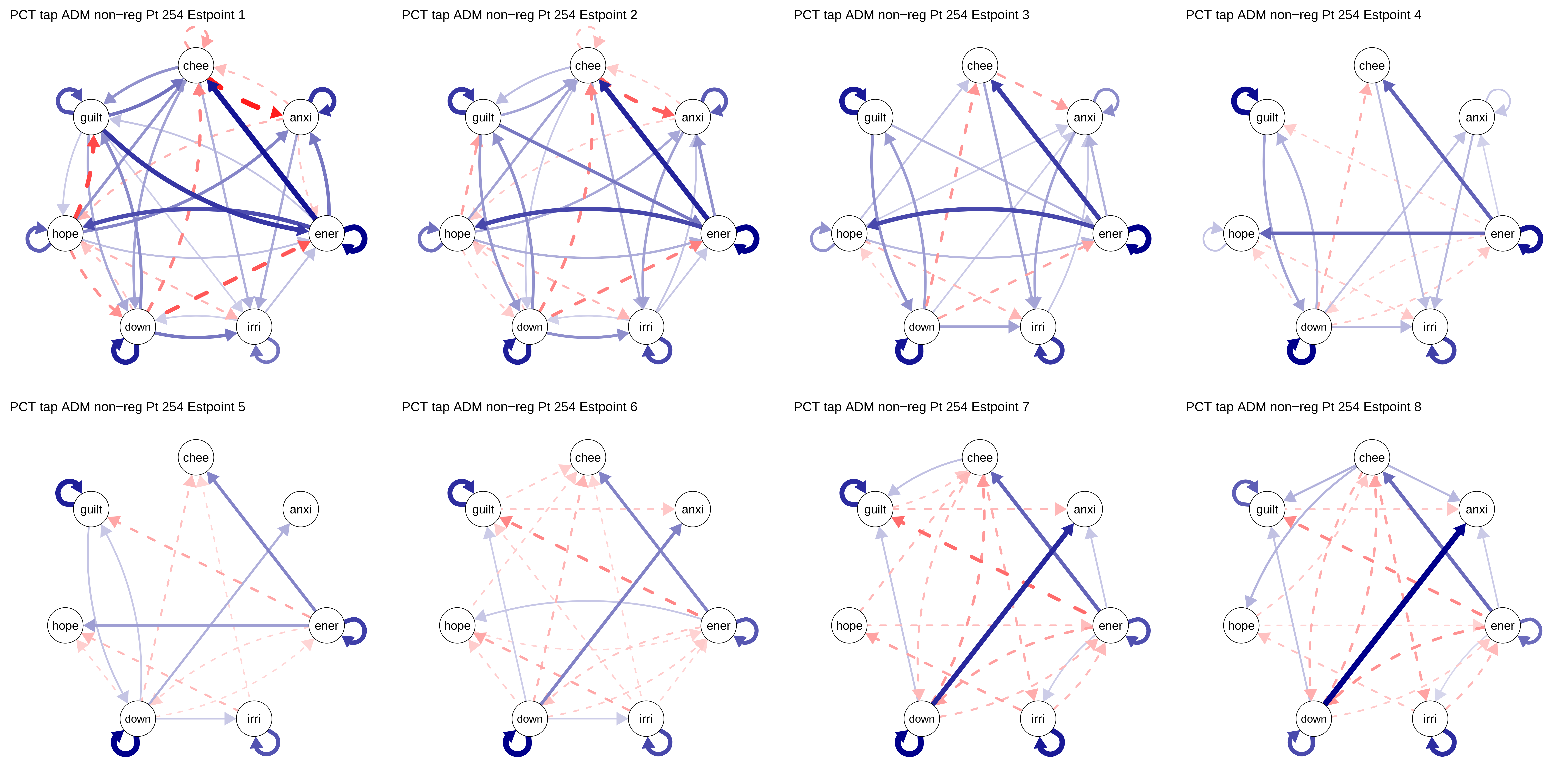
PCT tap ADM non−reg Pt 254 Estpoint 1
PCT tap ADM non−reg Pt 254 Estpoint 2
PCT tap ADM non−reg Pt 254 Estpoint 3
PCT tap ADM non−reg Pt 254 Estpoint 4
PCT tap ADM non−reg Pt 254 Estpoint 5
PCT tap ADM non−reg Pt 254 Estpoint 6
PCT tap ADM non−reg Pt 254 Estpoint 7
PCT tap ADM non−reg Pt 254 Estpoint 8
chee
guilt
anxi
hope
ener
down
irri

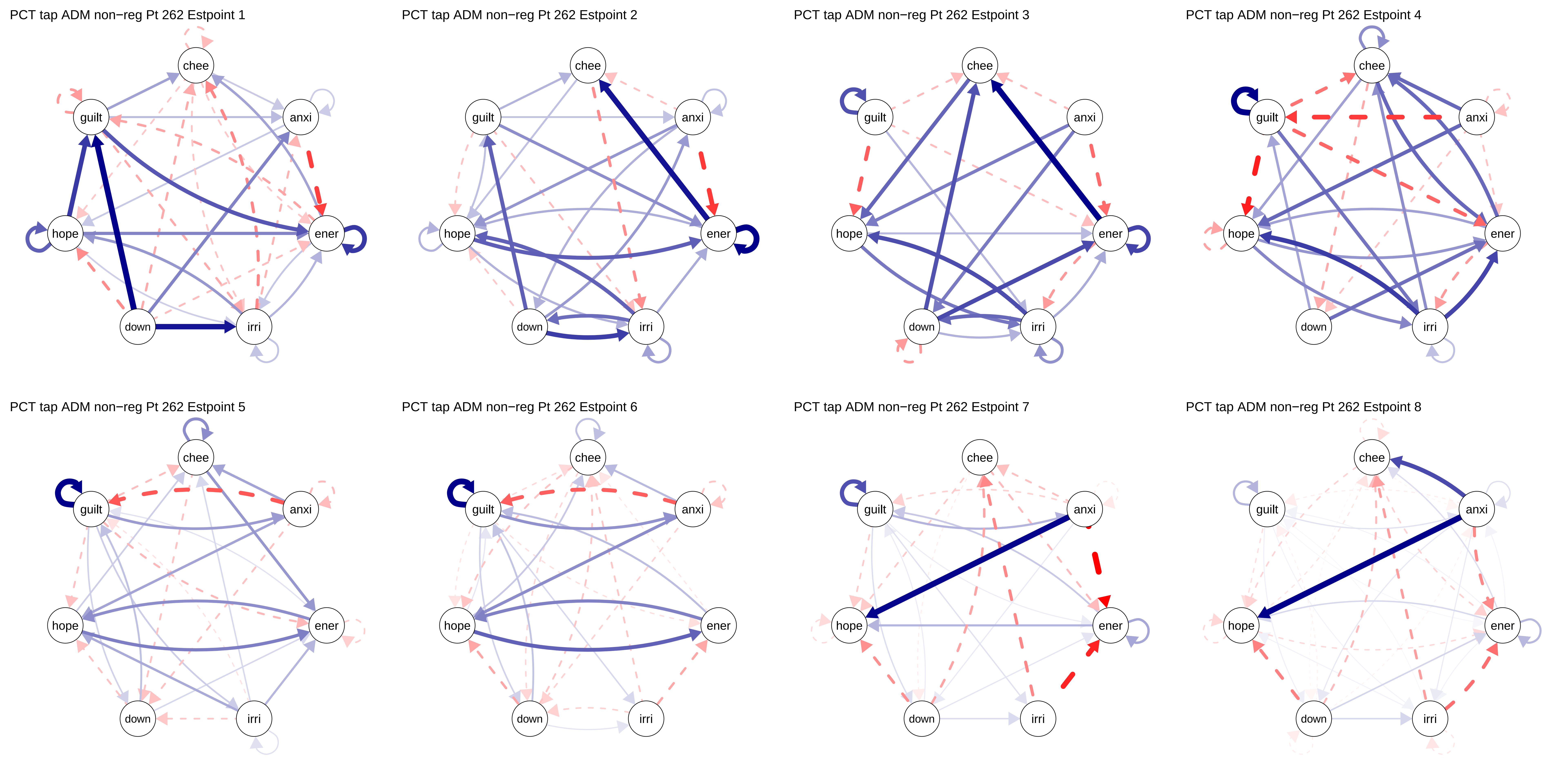
PCT tap ADM non−reg Pt 262 Estpoint 1
PCT tap ADM non−reg Pt 262 Estpoint 2
PCT tap ADM non−reg Pt 262 Estpoint 3
PCT tap ADM non−reg Pt 262 Estpoint 4
PCT tap ADM non−reg Pt 262 Estpoint 5
PCT tap ADM non−reg Pt 262 Estpoint 6
PCT tap ADM non−reg Pt 262 Estpoint 7
PCT tap ADM non−reg Pt 262 Estpoint 8
chee
guilt
anxi
hope
ener
down
irri

PCT tap ADM non−reg Pt 236 Estpoint 1

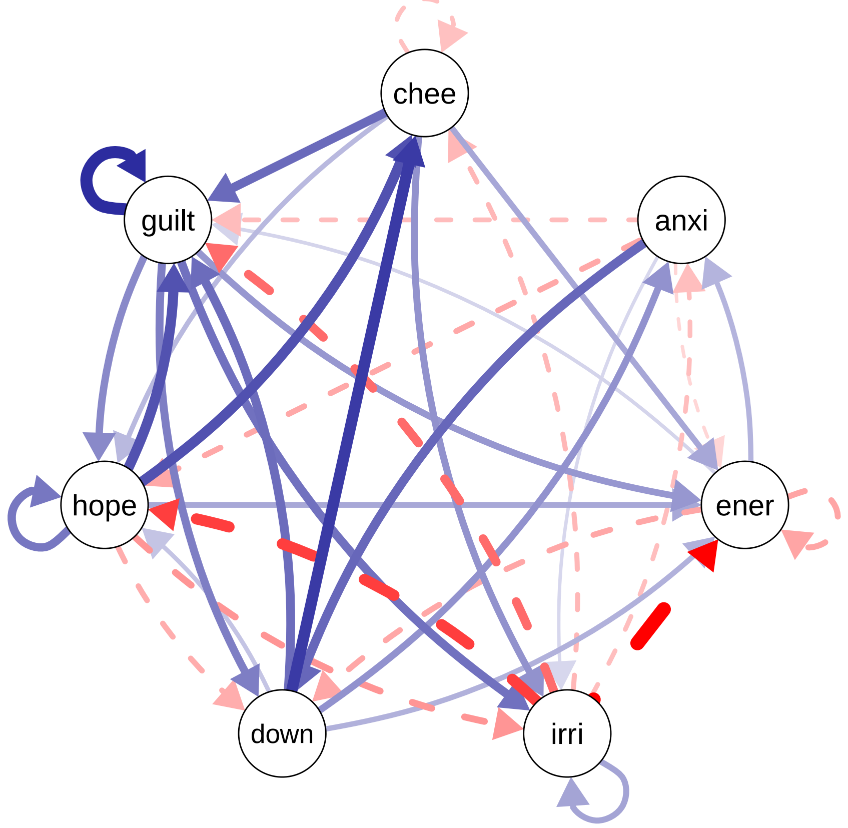

PCT tap ADM non−reg Pt 236 Estpoint 2

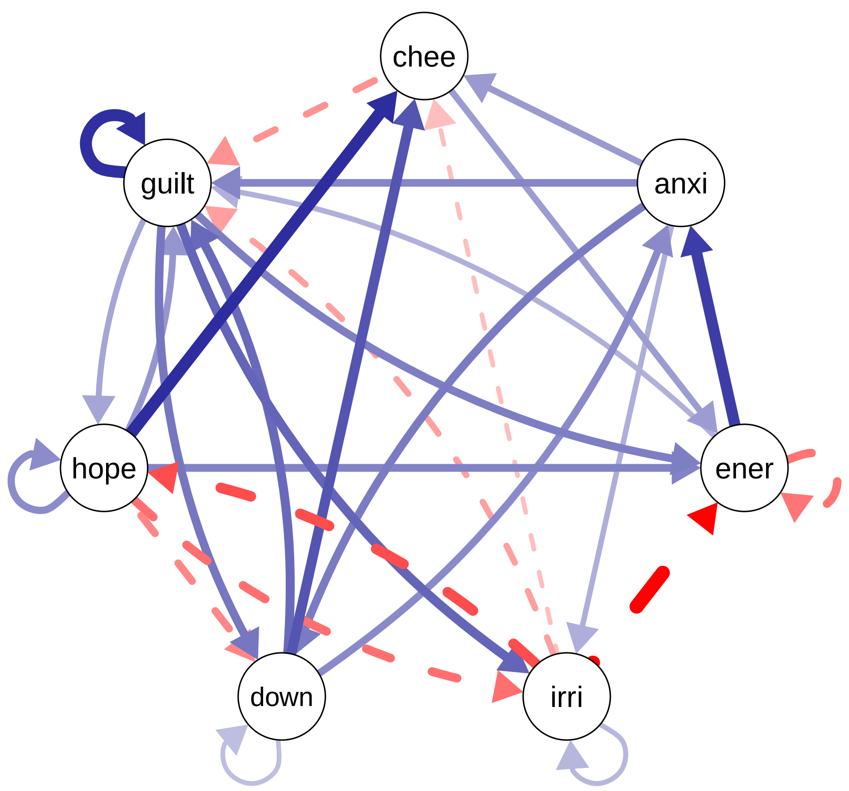

PCT tap ADM non−reg Pt 236 Estpoint 3

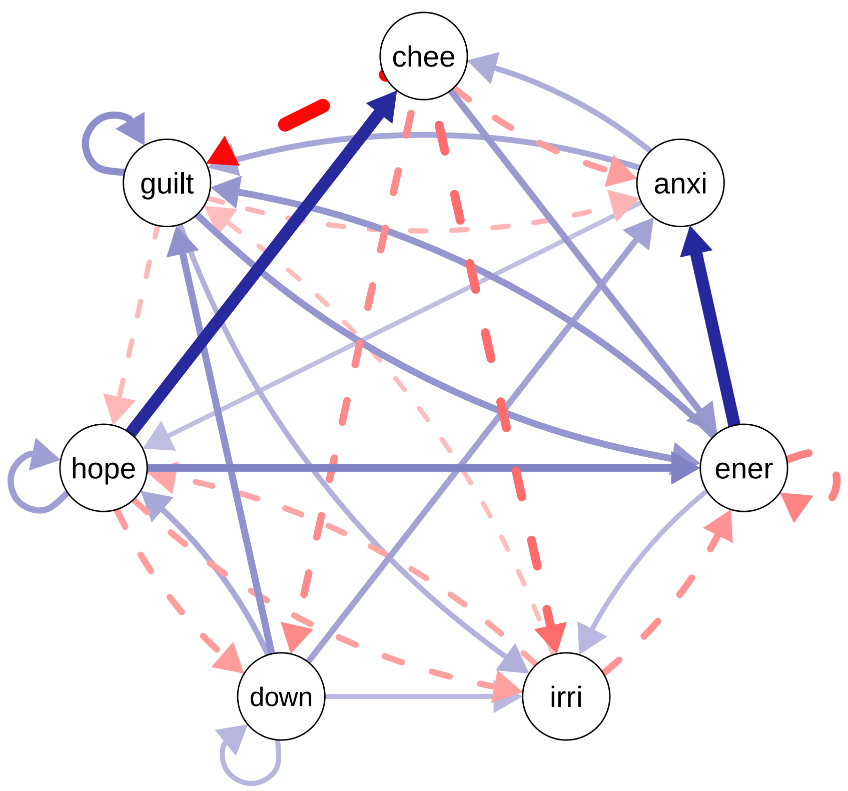

PCT tap ADM non−reg Pt 236 Estpoint 4

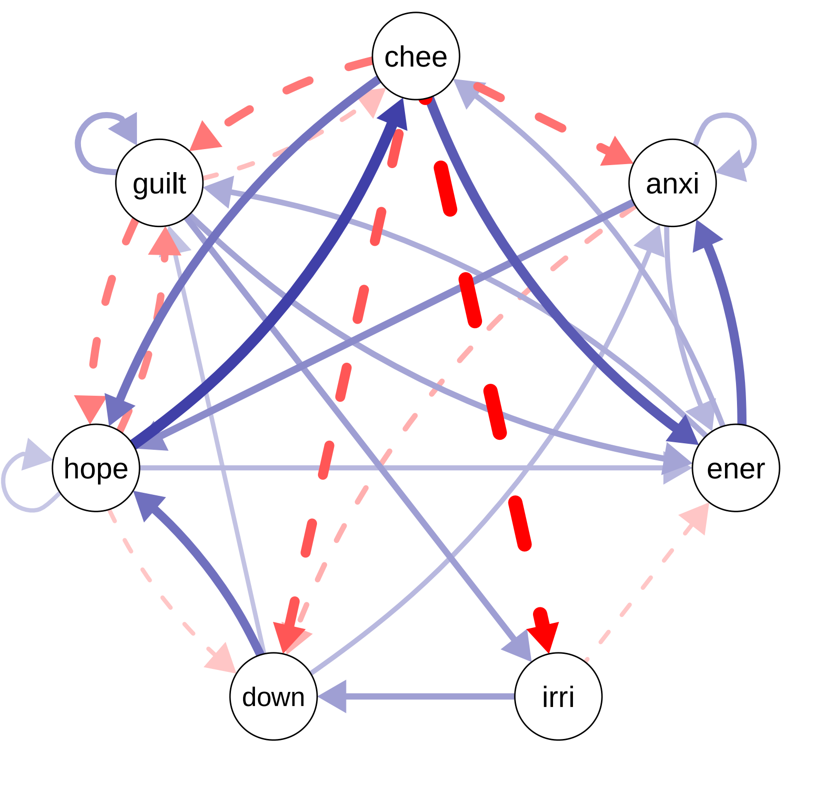

PCT tap ADM non−reg Pt 236 Estpoint 5

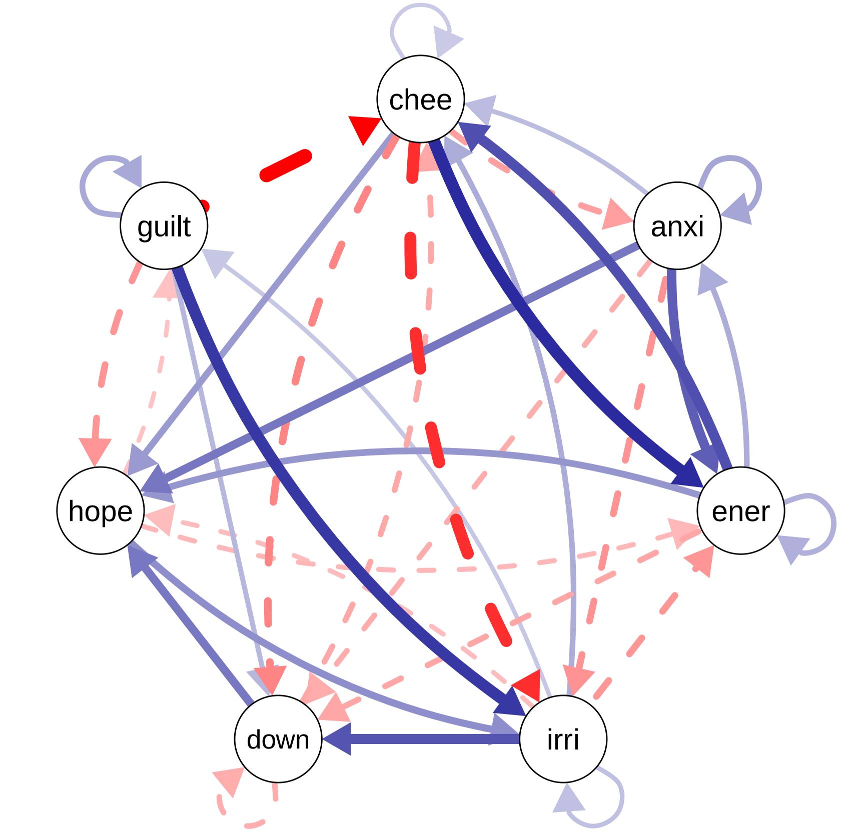

PCT tap ADM non−reg Pt 236 Estpoint 6

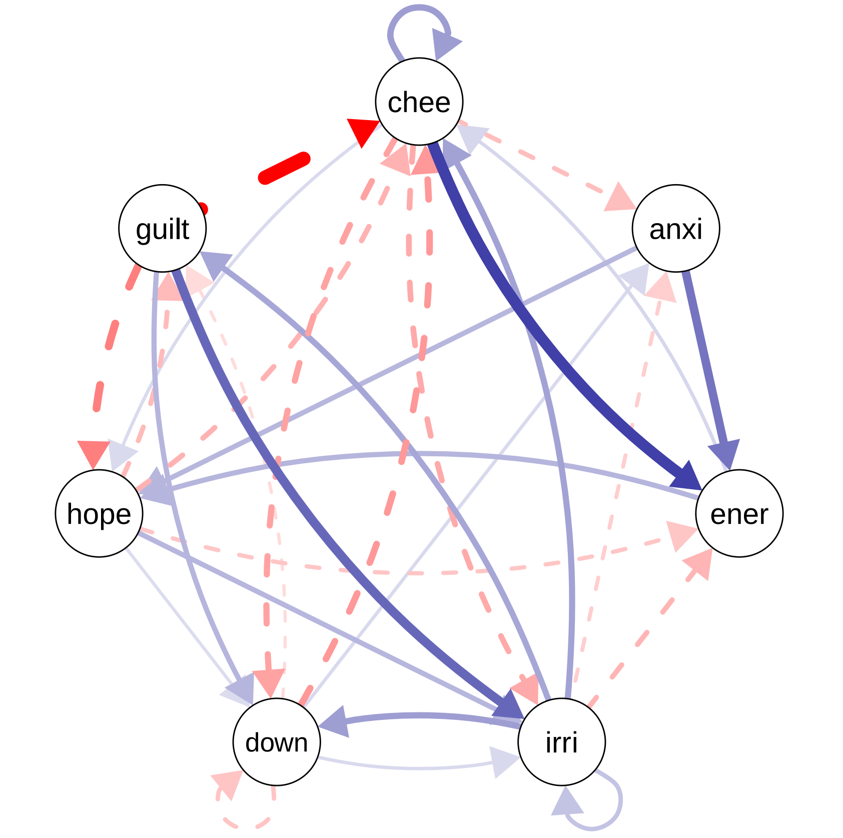

PCT tap ADM non−reg Pt 236 Estpoint 7

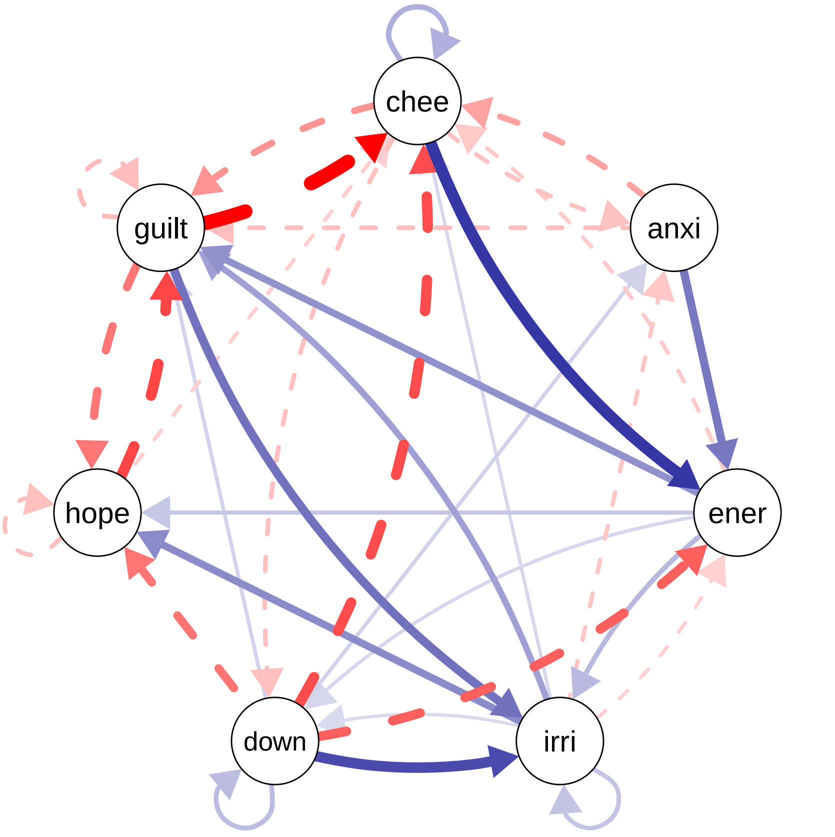

PCT tap ADM non−reg Pt 236 Estpoint 8

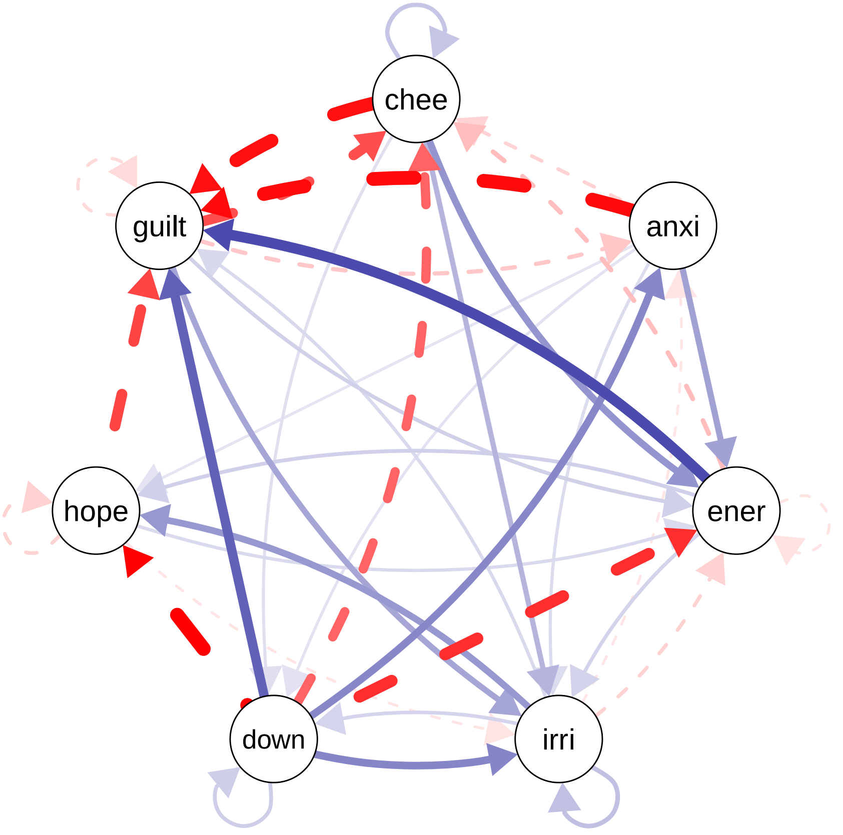

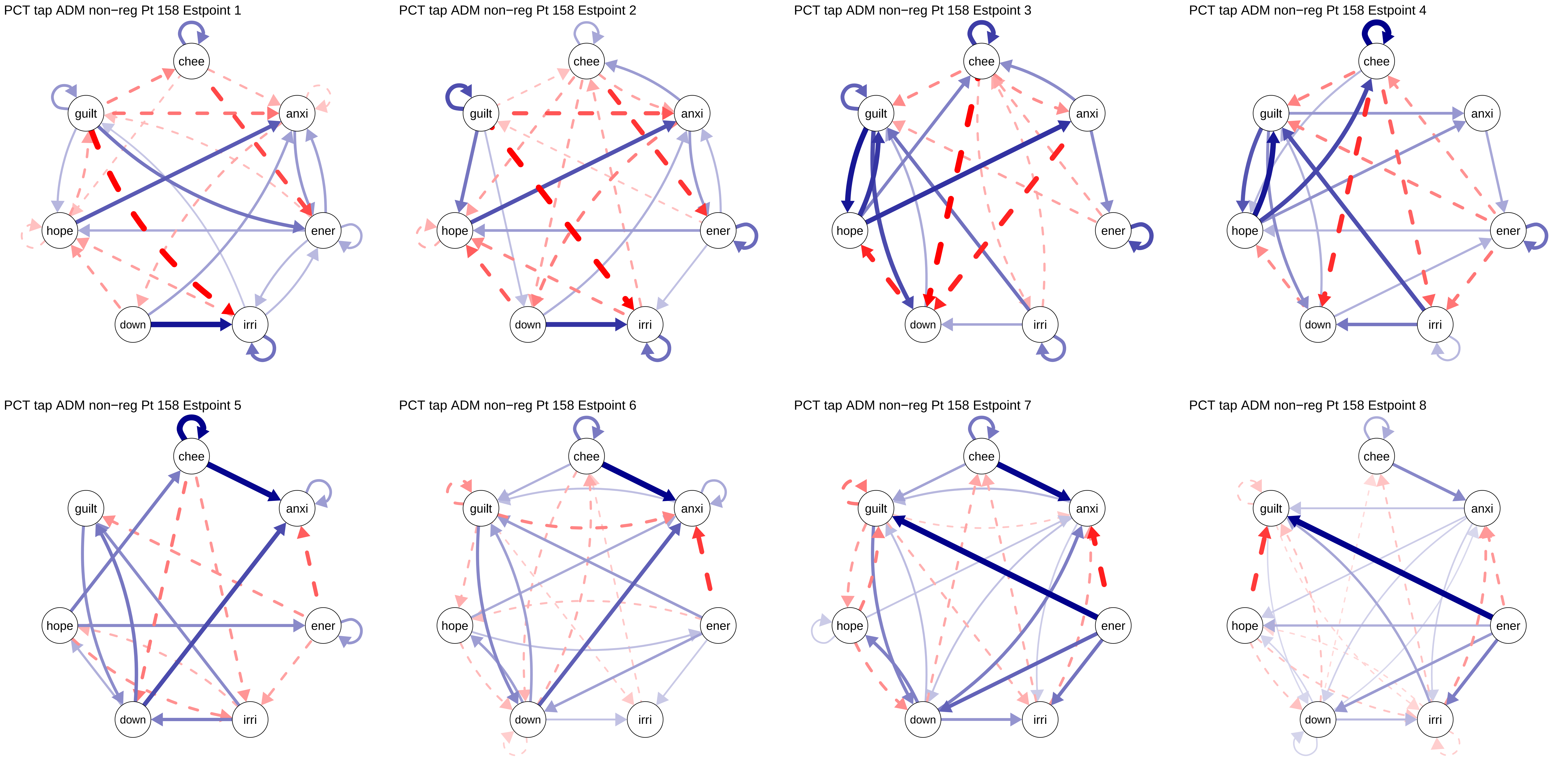
PCT tap ADM non−reg Pt 158 Estpoint 1
PCT tap ADM non−reg Pt 158 Estpoint 2
PCT tap ADM non−reg Pt 158 Estpoint 3
PCT tap ADM non−reg Pt 158 Estpoint 4
PCT tap ADM non−reg Pt 158 Estpoint 5
PCT tap ADM non−reg Pt 158 Estpoint 6
PCT tap ADM non−reg Pt 158 Estpoint 7
PCT tap ADM non−reg Pt 158 Estpoint 8
chee
guilt
anxi
hope
ener
down
irri

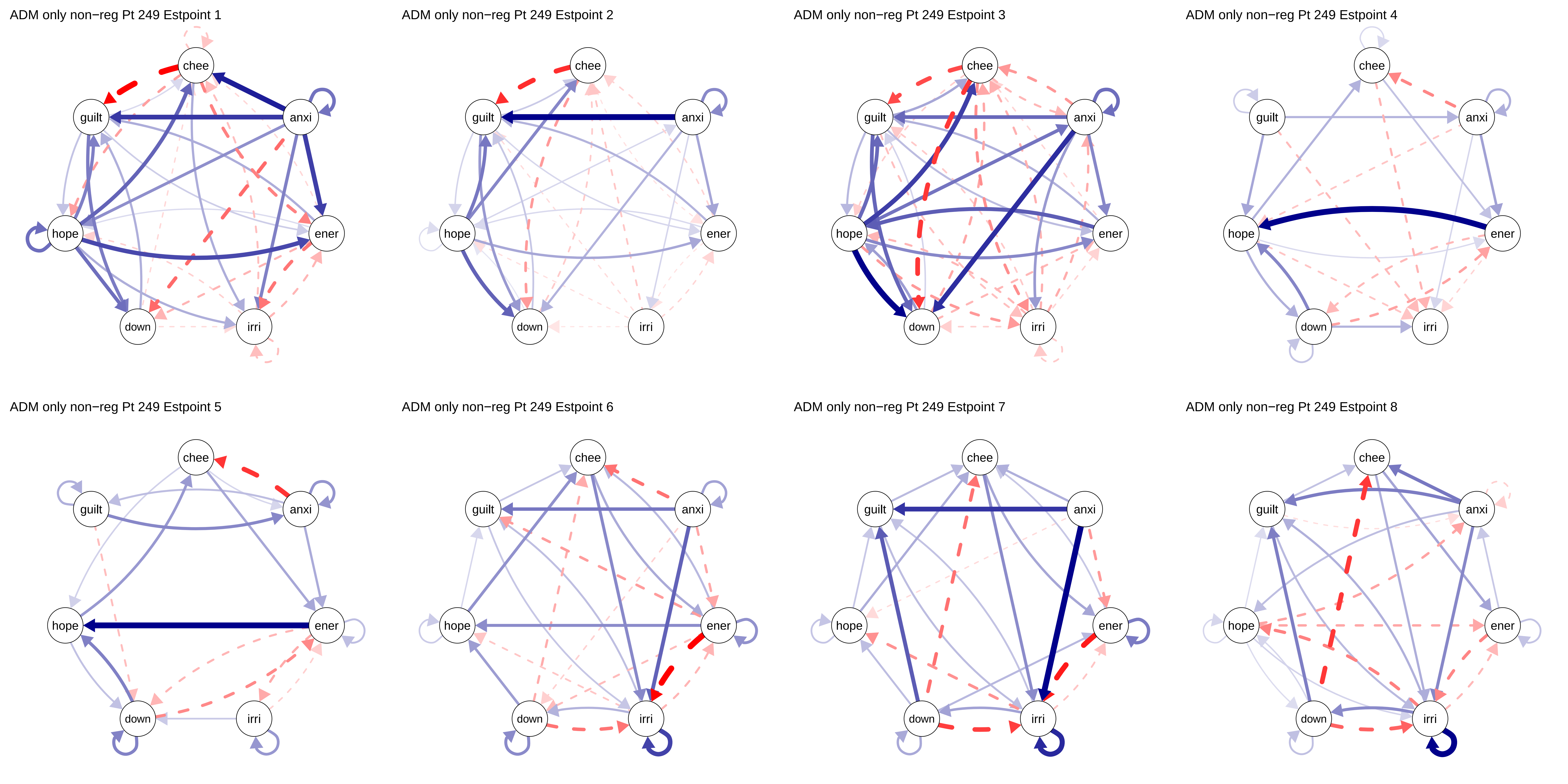
ADM only non−reg Pt 249 Estpoint 1
chee guilt anxi hope ener down irri
ADM only non−reg Pt 249 Estpoint 2
chee guilt anxi hope ener down irri
ADM only non−reg Pt 249 Estpoint 3
chee guilt anxi hope ener down irri
ADM only non−reg Pt 249 Estpoint 4
chee guilt anxi hope ener down irri
ADM only non−reg Pt 249 Estpoint 5
chee guilt anxi hope ener down irri
ADM only non−reg Pt 249 Estpoint 6
chee guilt anxi hope ener down irri
ADM only non−reg Pt 249 Estpoint 7
chee guilt anxi hope ener down irri
ADM only non−reg Pt 249 Estpoint 8
chee guilt anxi hope ener down irri

ADM only non−reg Pt 273 Estpoint 1

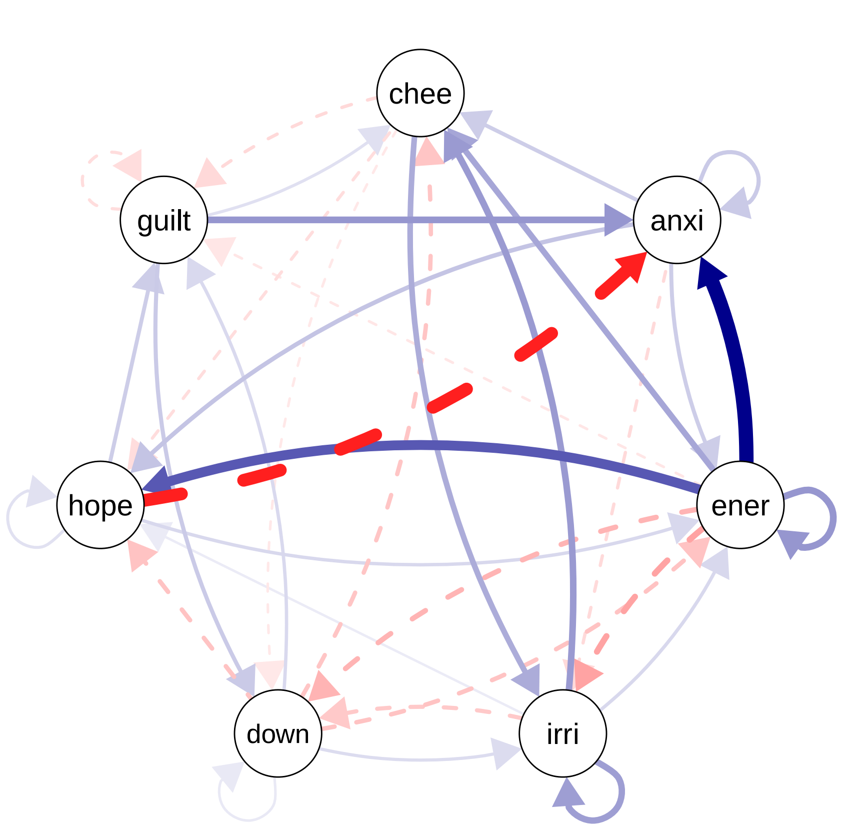

ADM only non−reg Pt 273 Estpoint 2

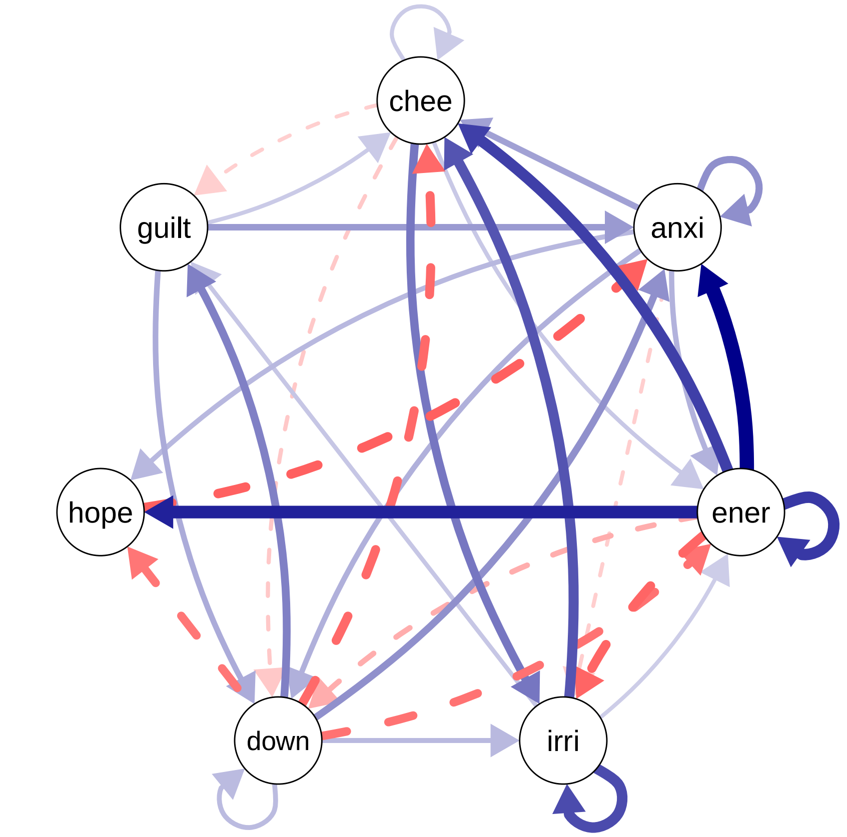

ADM only non−reg Pt 273 Estpoint 3

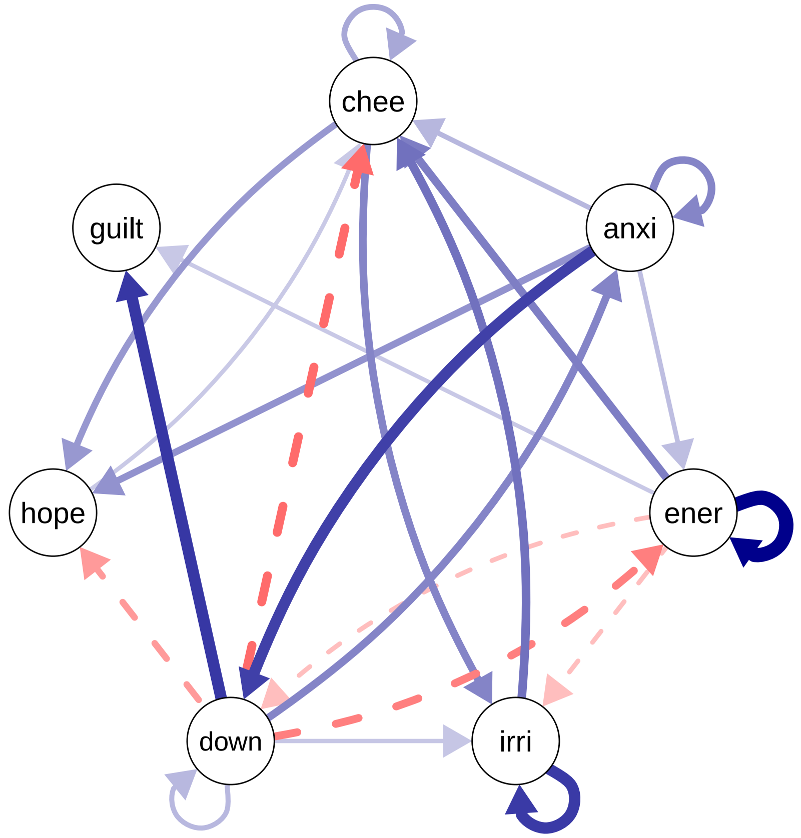

ADM only non−reg Pt 273 Estpoint 4

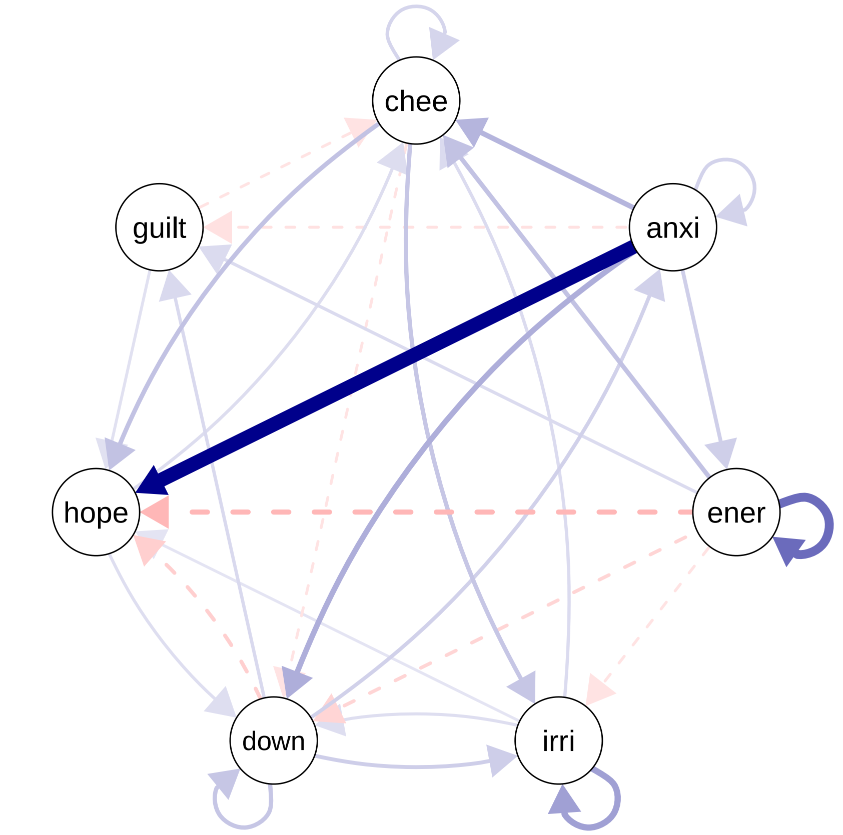

ADM only non−reg Pt 273 Estpoint 5

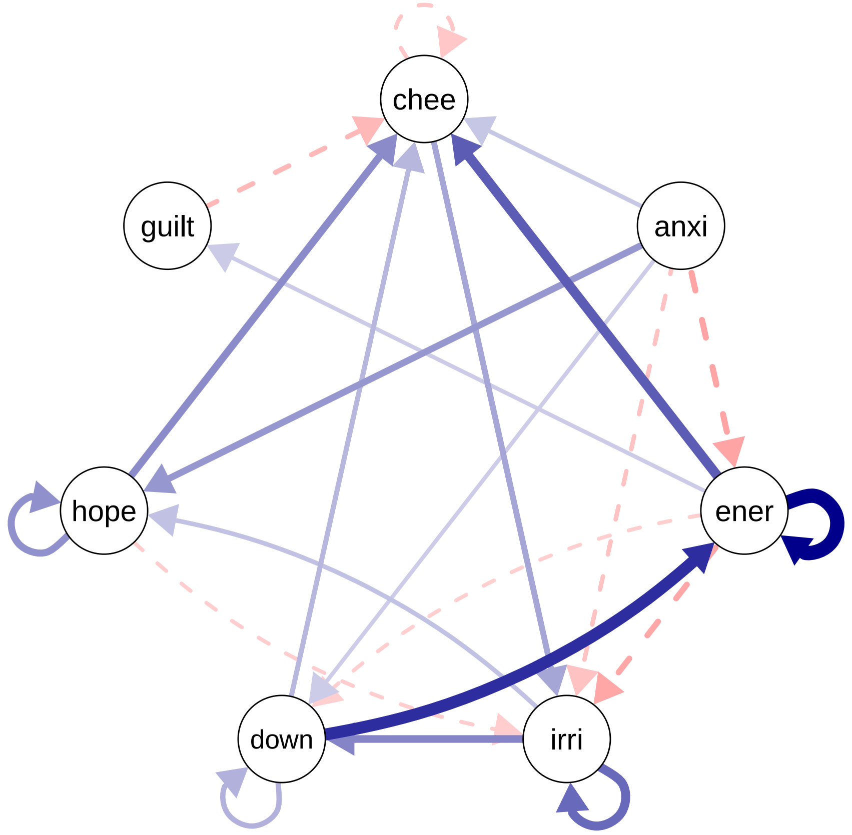

ADM only non−reg Pt 273 Estpoint 6

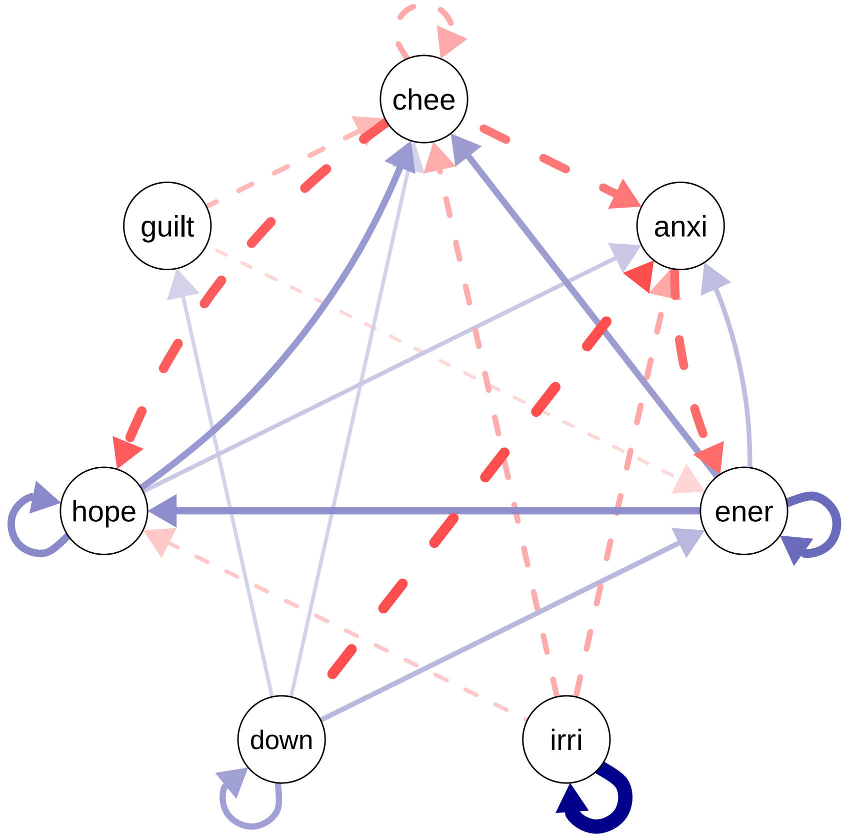

ADM only non−reg Pt 273 Estpoint 7

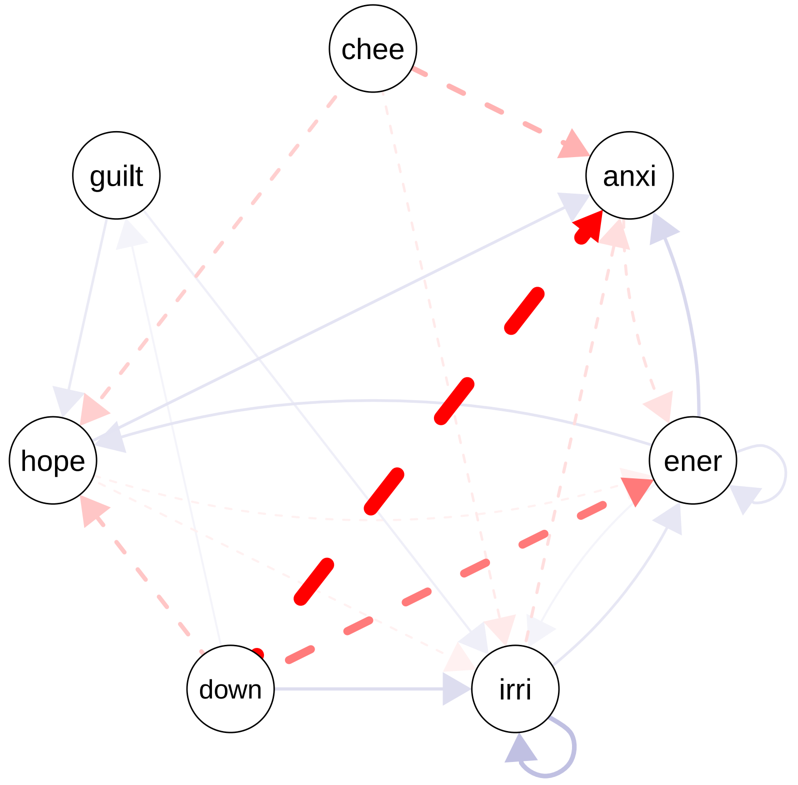

ADM only non−reg Pt 273 Estpoint 8

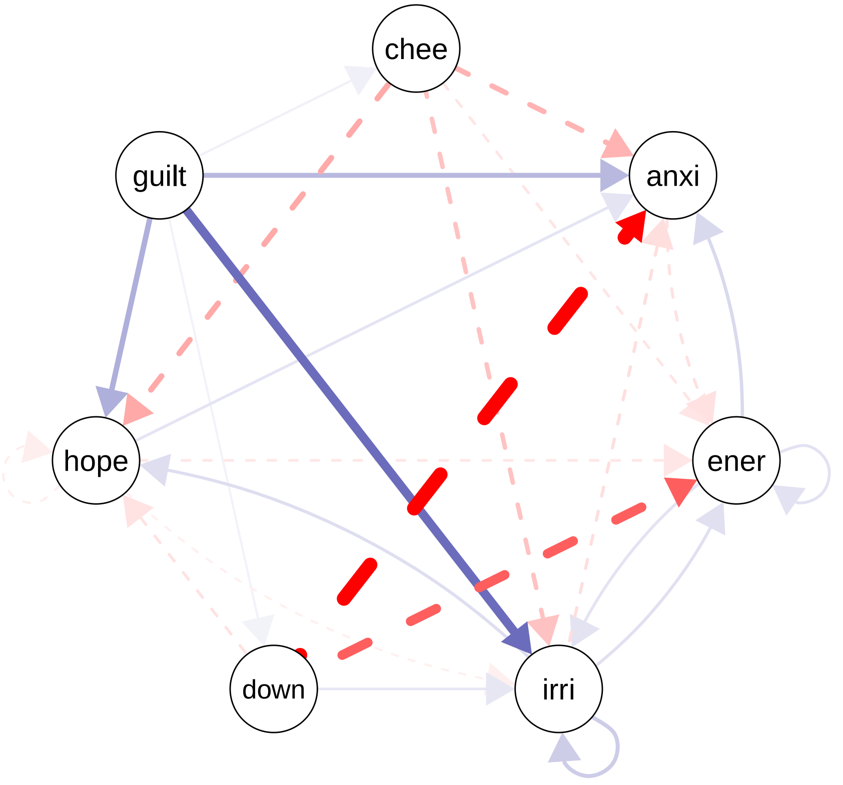

ADM only non−reg Pt 224 Estpoint 1

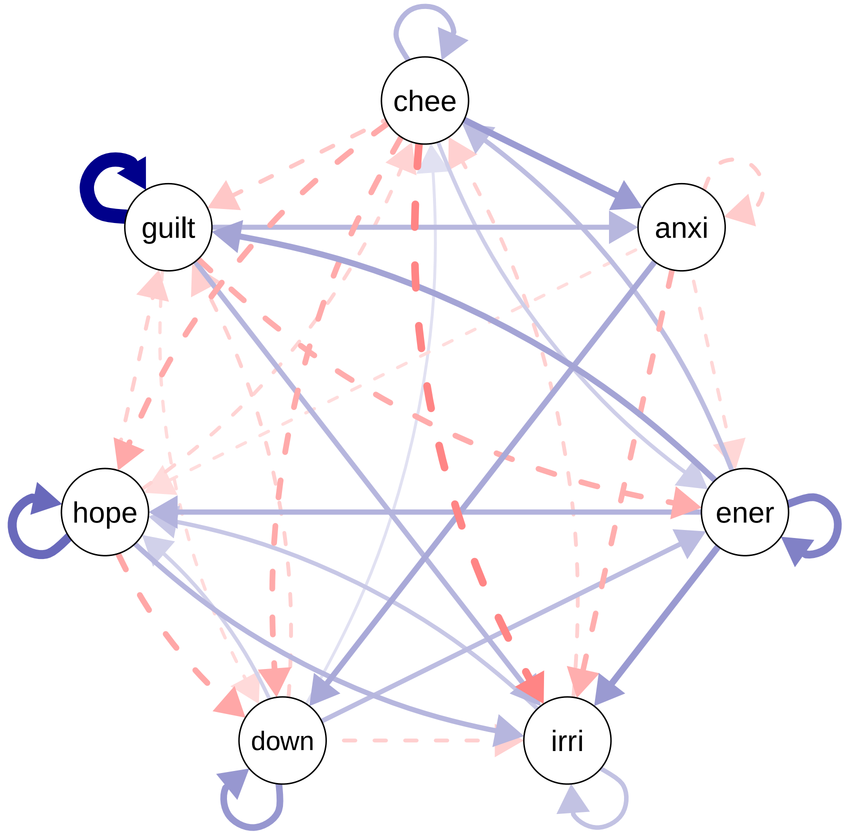

ADM only non−reg Pt 224 Estpoint 2

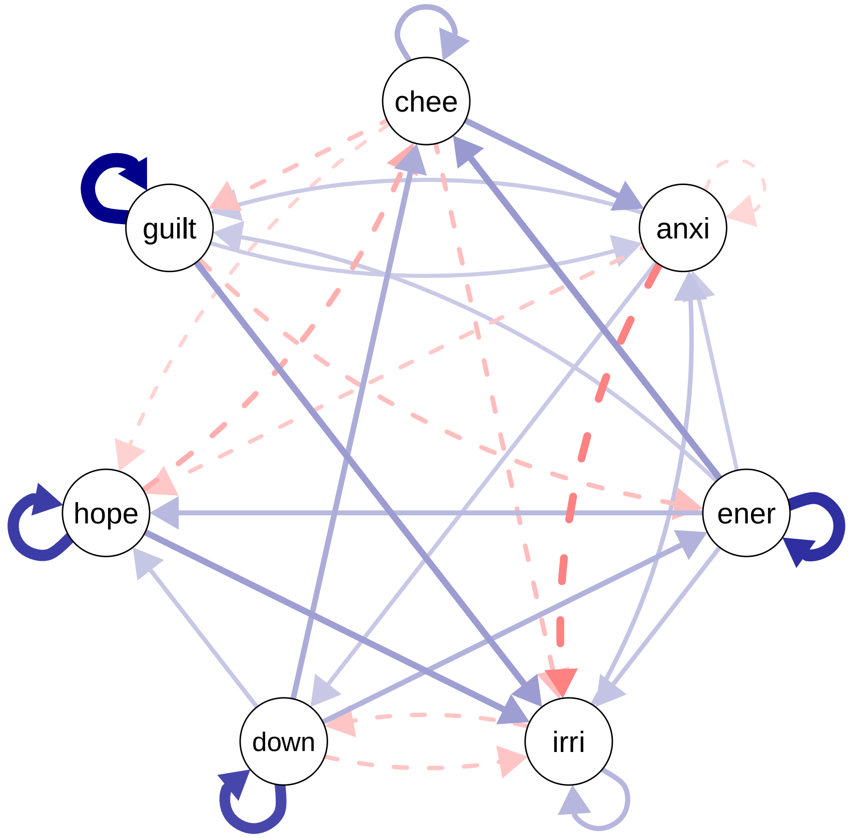

ADM only non−reg Pt 224 Estpoint 3

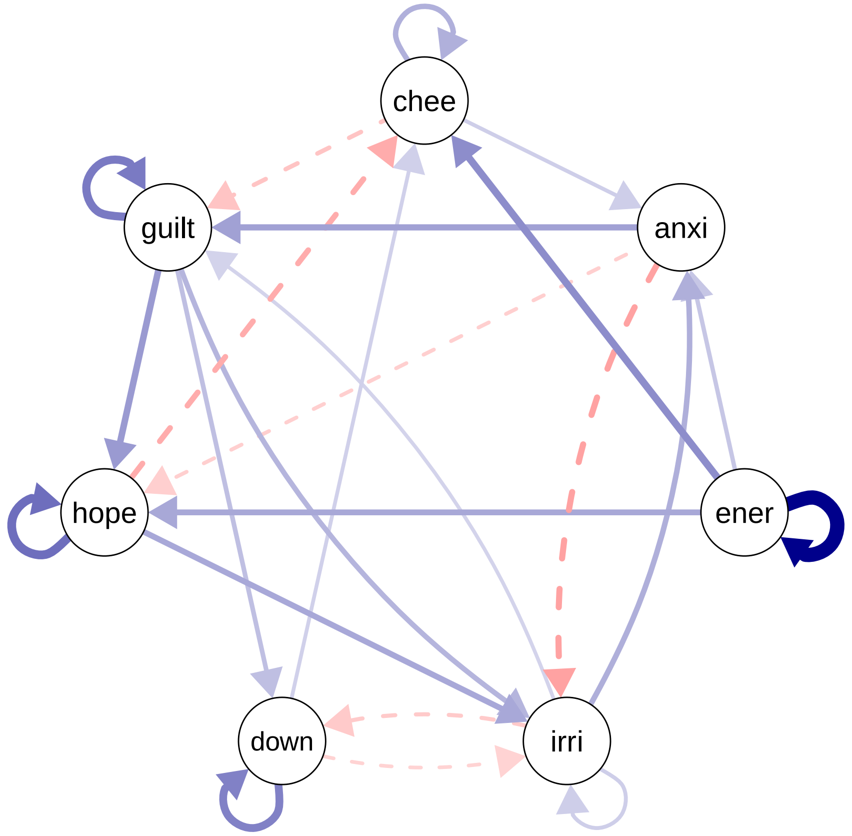

ADM only non−reg Pt 224 Estpoint 4

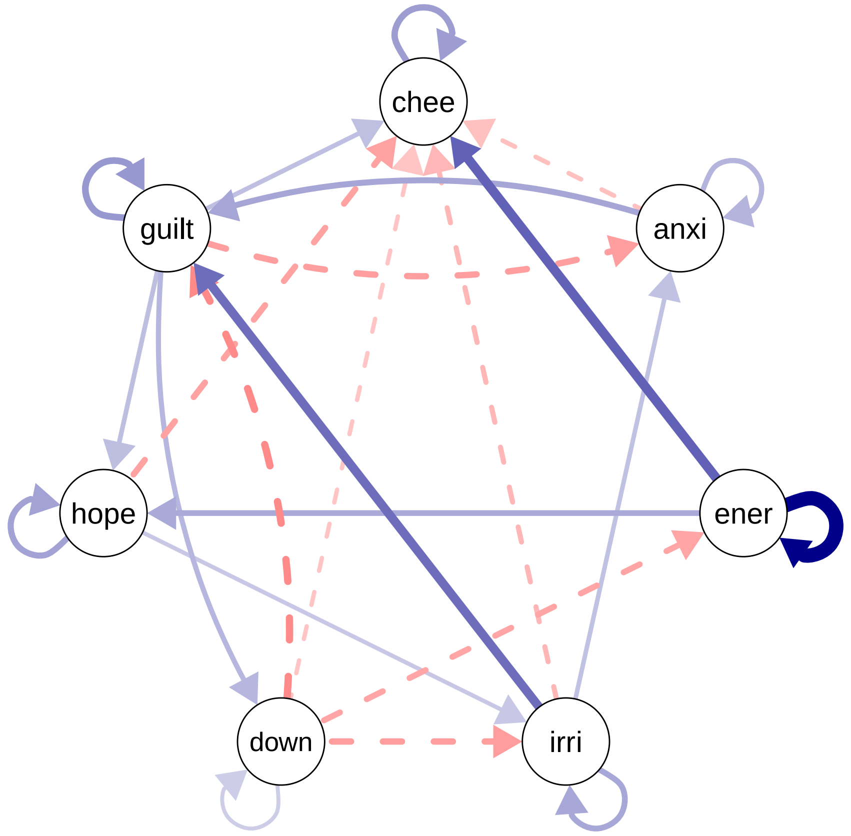

ADM only non−reg Pt 224 Estpoint 5

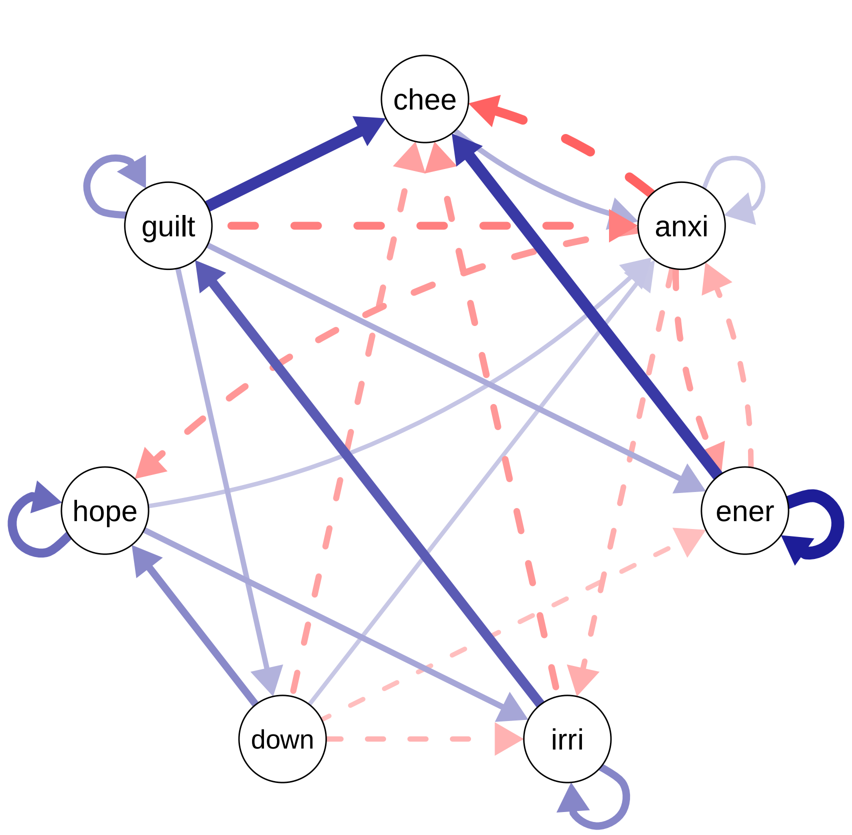

ADM only non−reg Pt 224 Estpoint 6

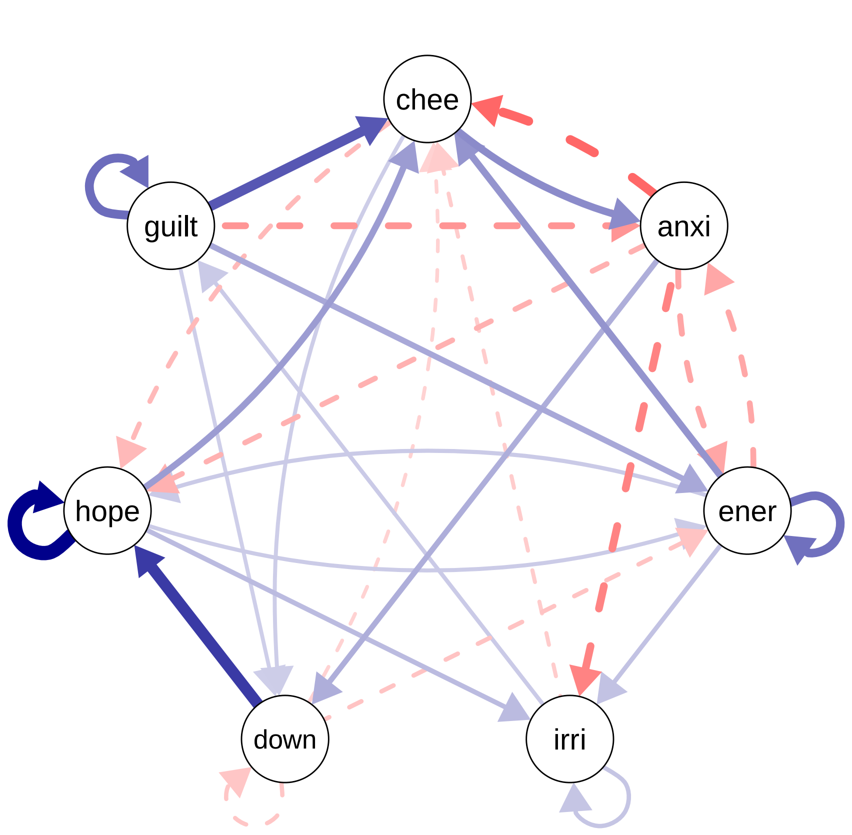

ADM only non−reg Pt 224 Estpoint 7

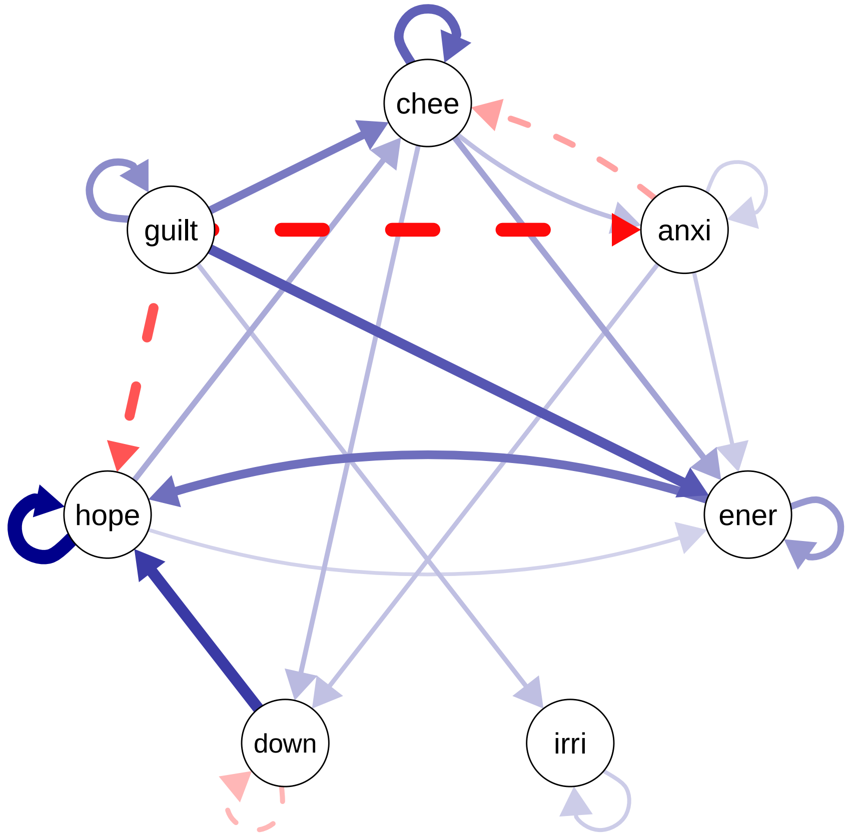

ADM only non−reg Pt 224 Estpoint 8

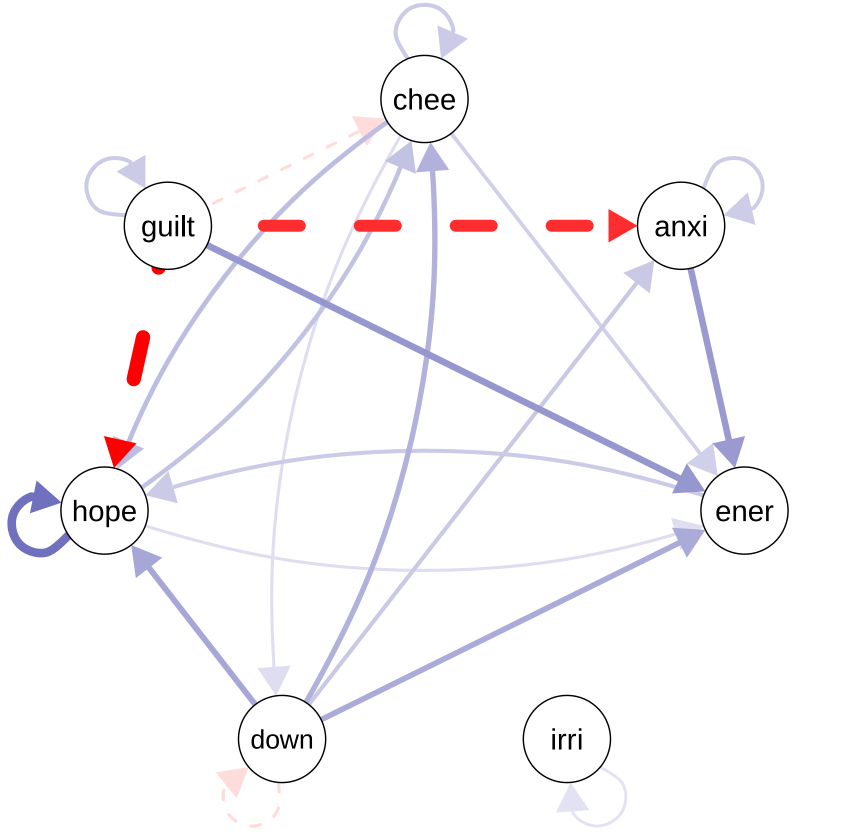

ADM only non−reg Pt 259 Estpoint 1

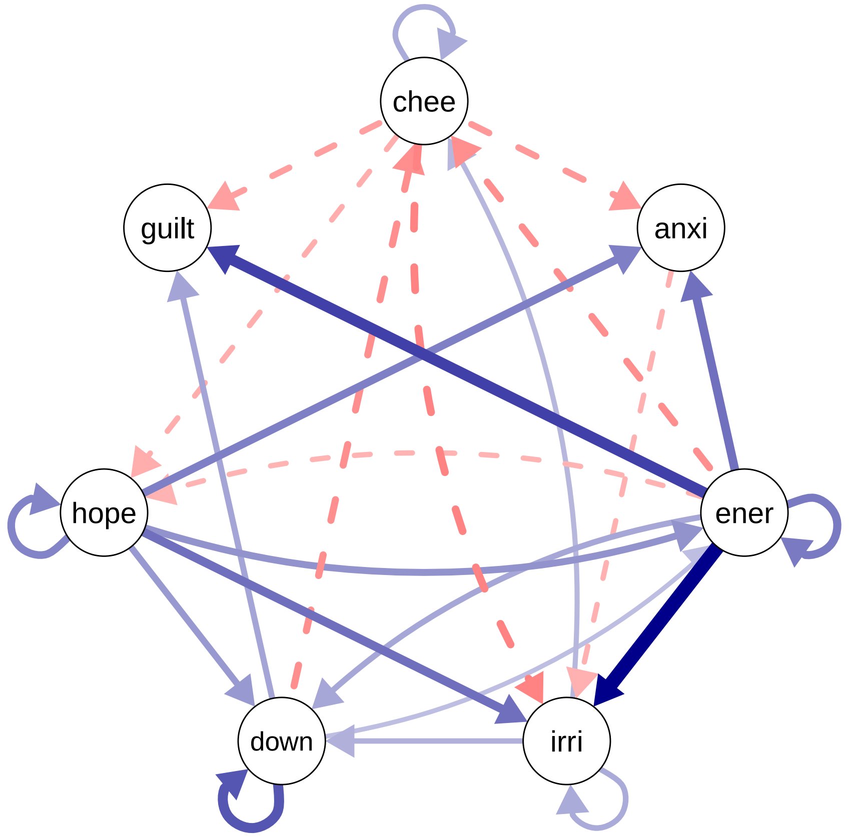

ADM only non−reg Pt 259 Estpoint 2

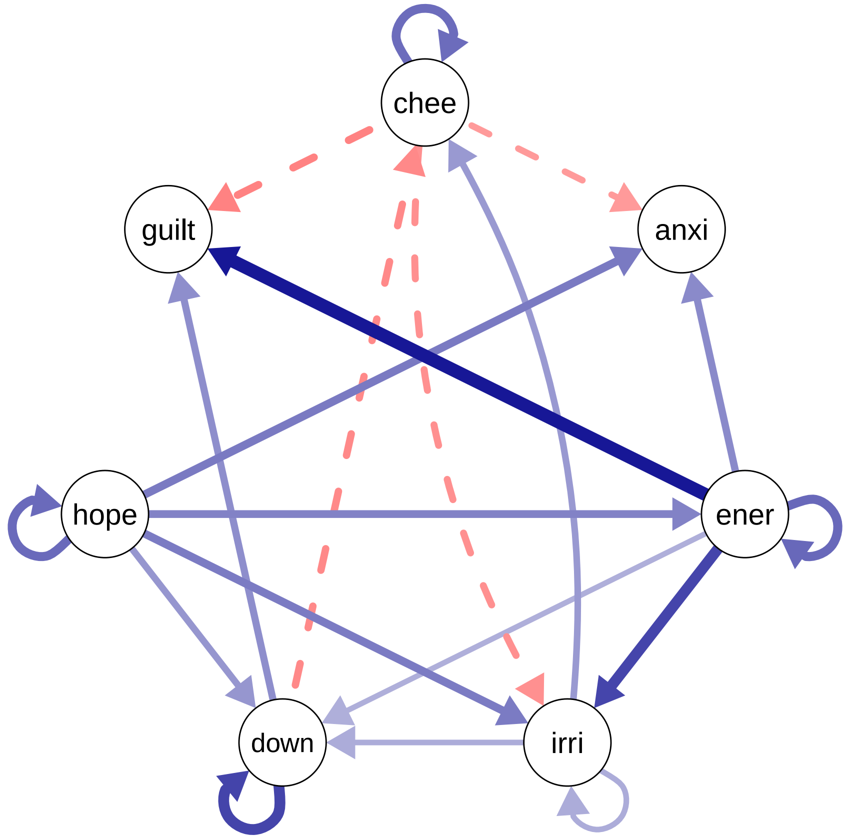

ADM only non−reg Pt 259 Estpoint 3

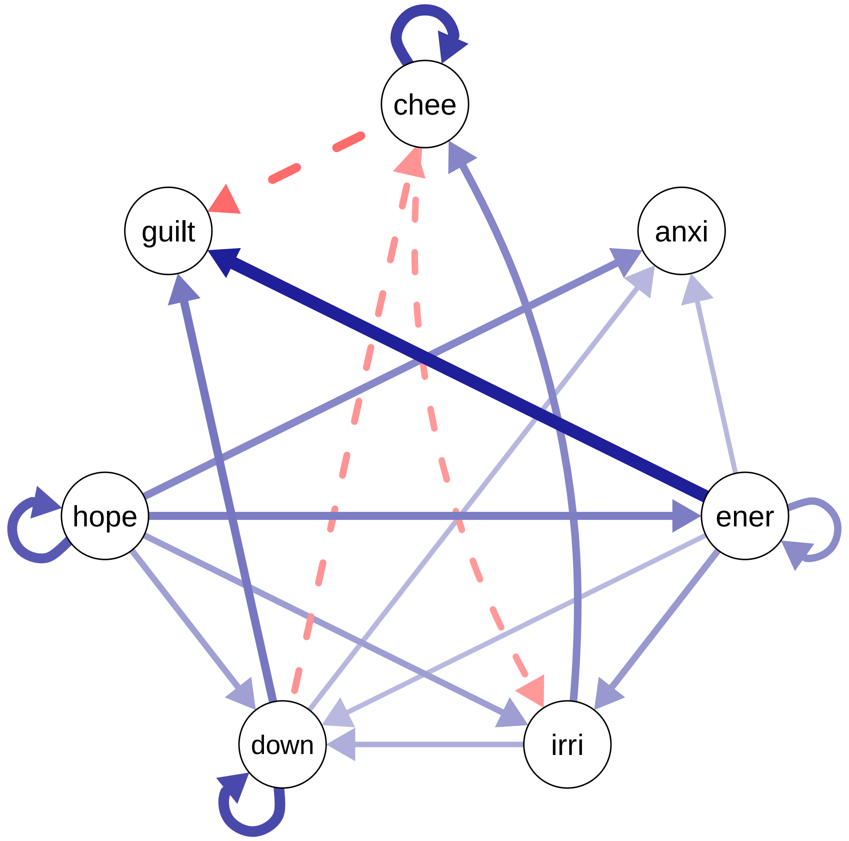

ADM only non−reg Pt 259 Estpoint 4

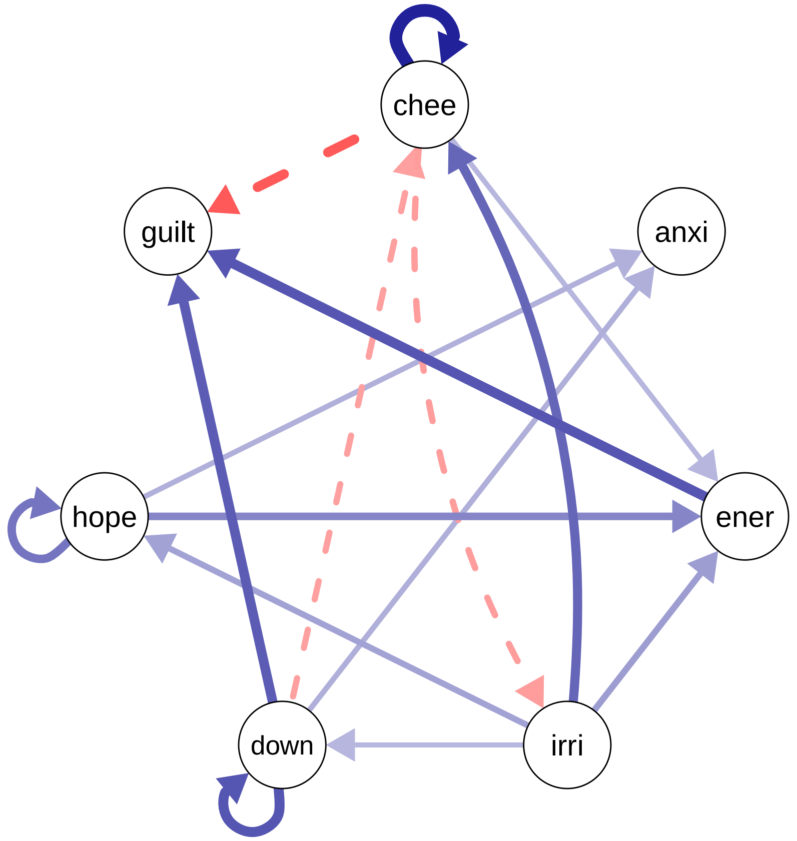

ADM only non−reg Pt 259 Estpoint 5

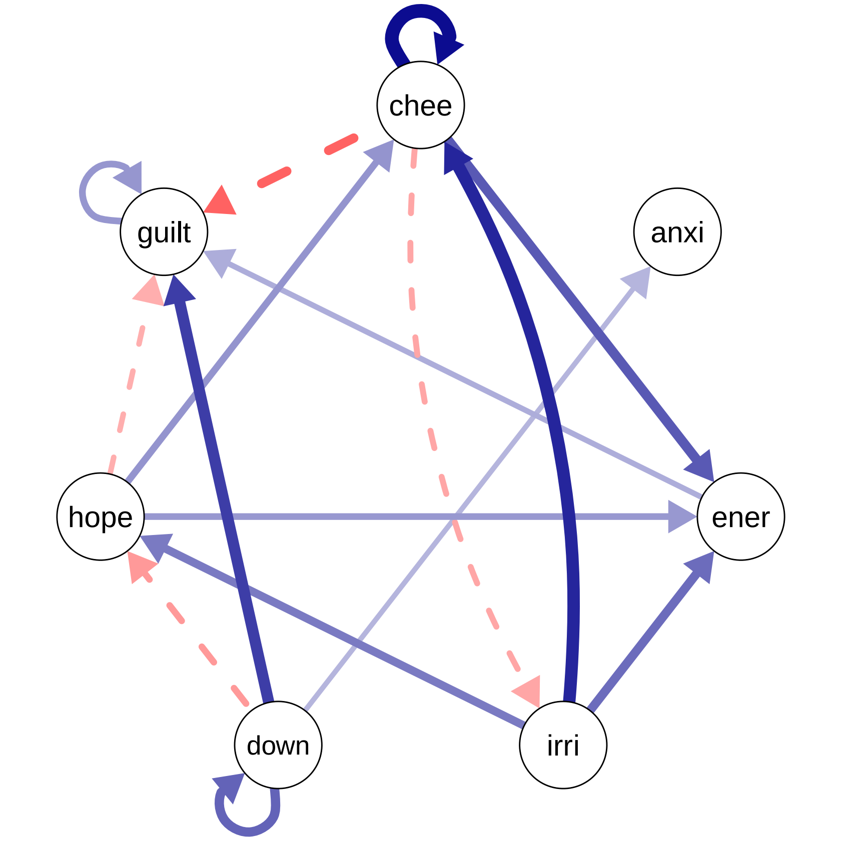

ADM only non−reg Pt 259 Estpoint 6

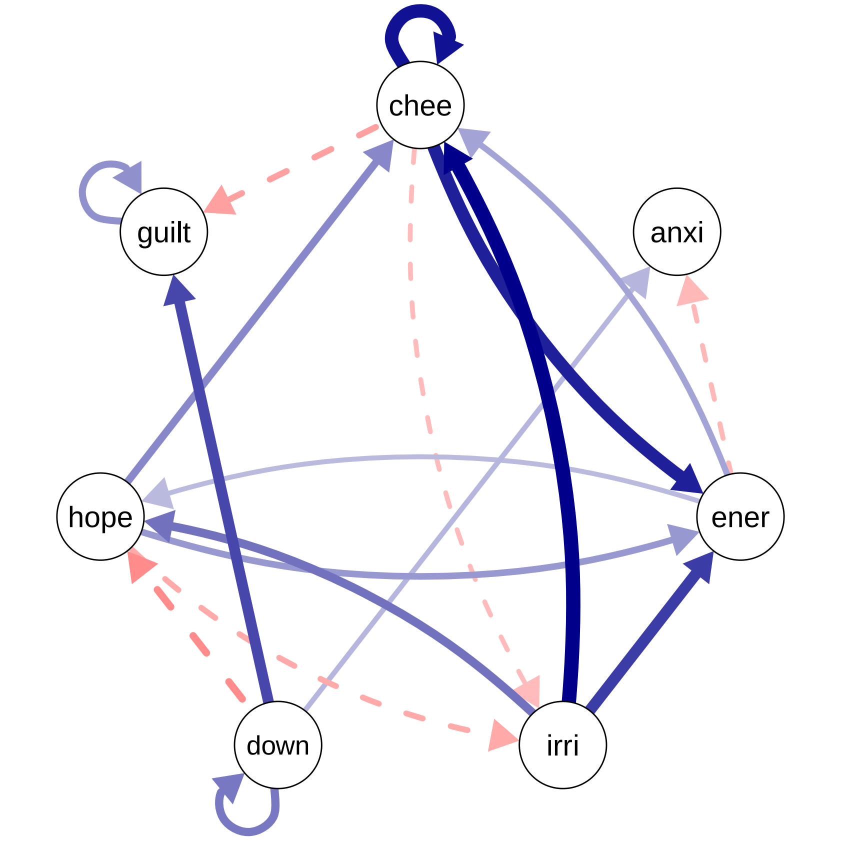

ADM only non−reg Pt 259 Estpoint 7

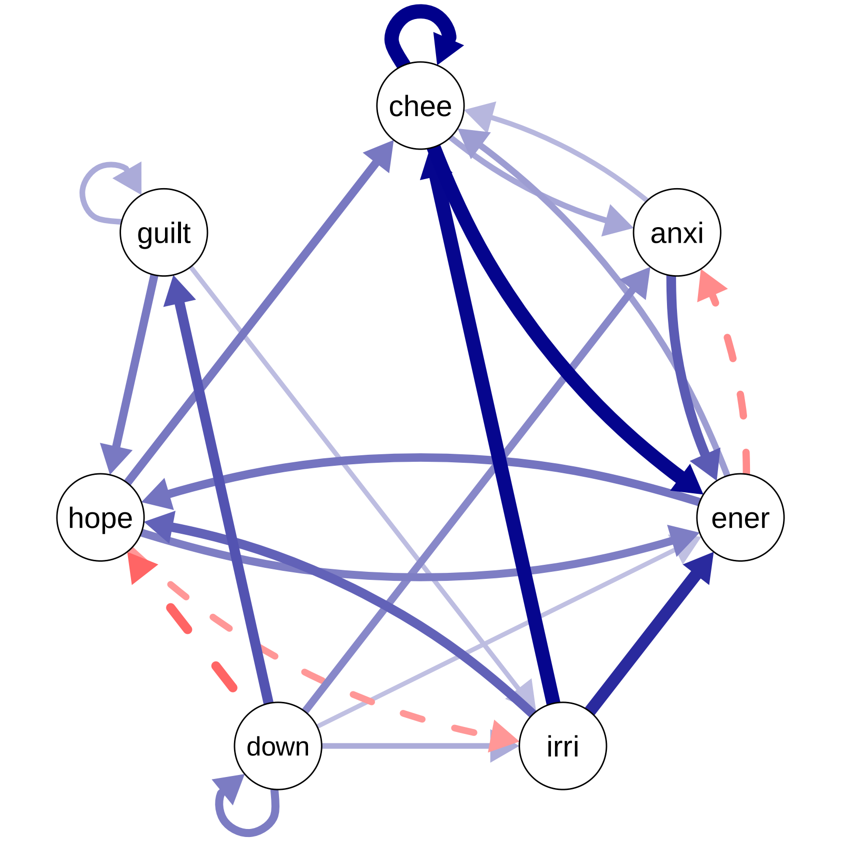

ADM only non−reg Pt 259 Estpoint 8

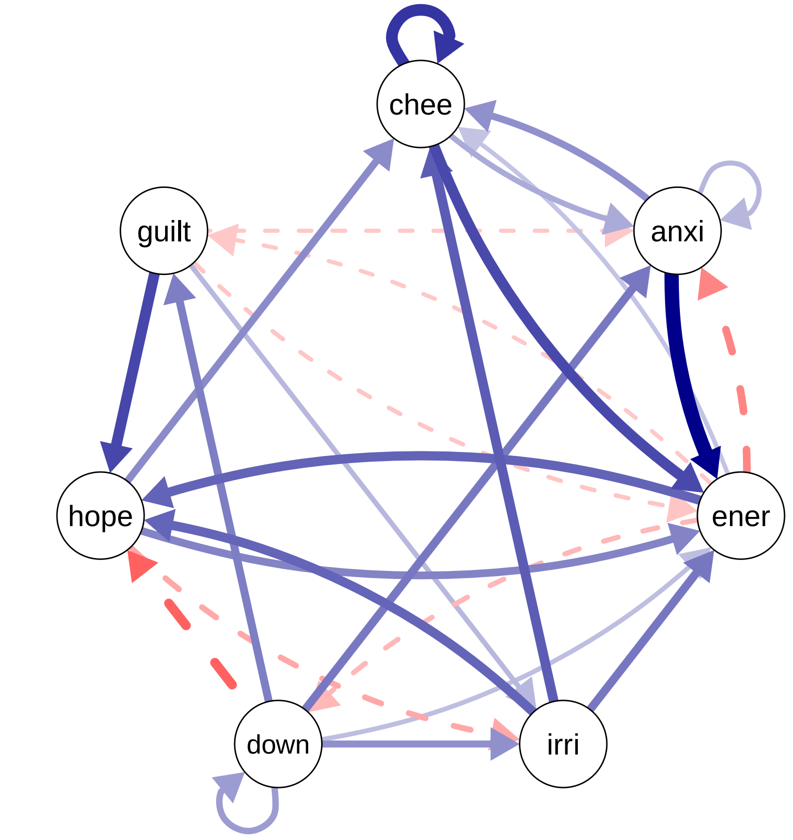

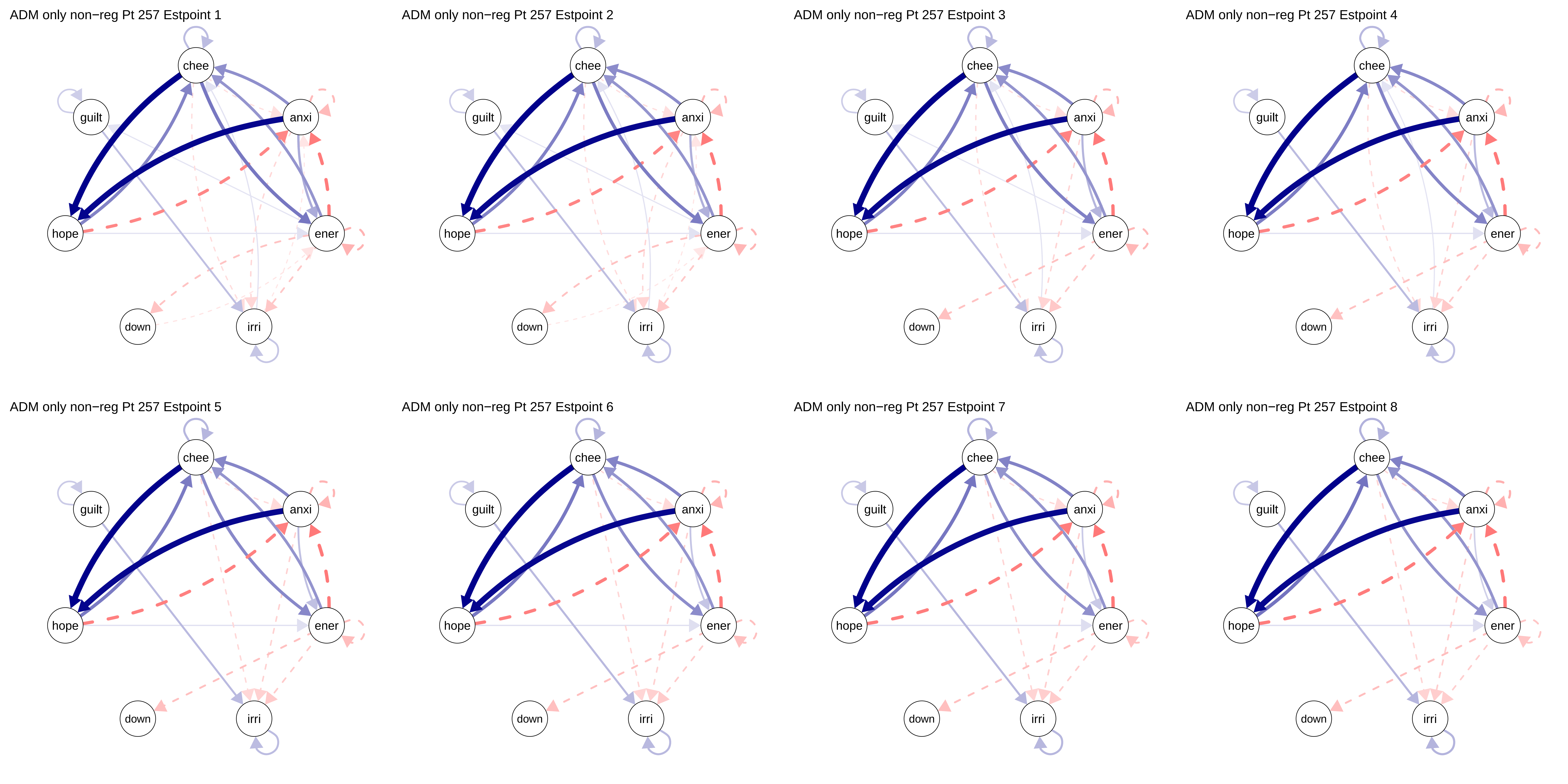
ADM only non−reg Pt 257 Estpoint 1
chee
guilt
anxi
hope
ener
down
irri
ADM only non−reg Pt 257 Estpoint 2
chee
guilt
anxi
hope
ener
down
irri
ADM only non−reg Pt 257 Estpoint 3
chee
guilt
anxi
hope
ener
down
irri
ADM only non−reg Pt 257 Estpoint 4
chee
guilt
anxi
hope
ener
down
irri
ADM only non−reg Pt 257 Estpoint 5
chee
guilt
anxi
hope
ener
down
irri
ADM only non−reg Pt 257 Estpoint 6
chee
guilt
anxi
hope
ener
down
irri
ADM only non−reg Pt 257 Estpoint 7
chee
guilt
anxi
hope
ener
down
irri
ADM only non−reg Pt 257 Estpoint 8
chee
guilt
anxi
hope
ener
down
irri

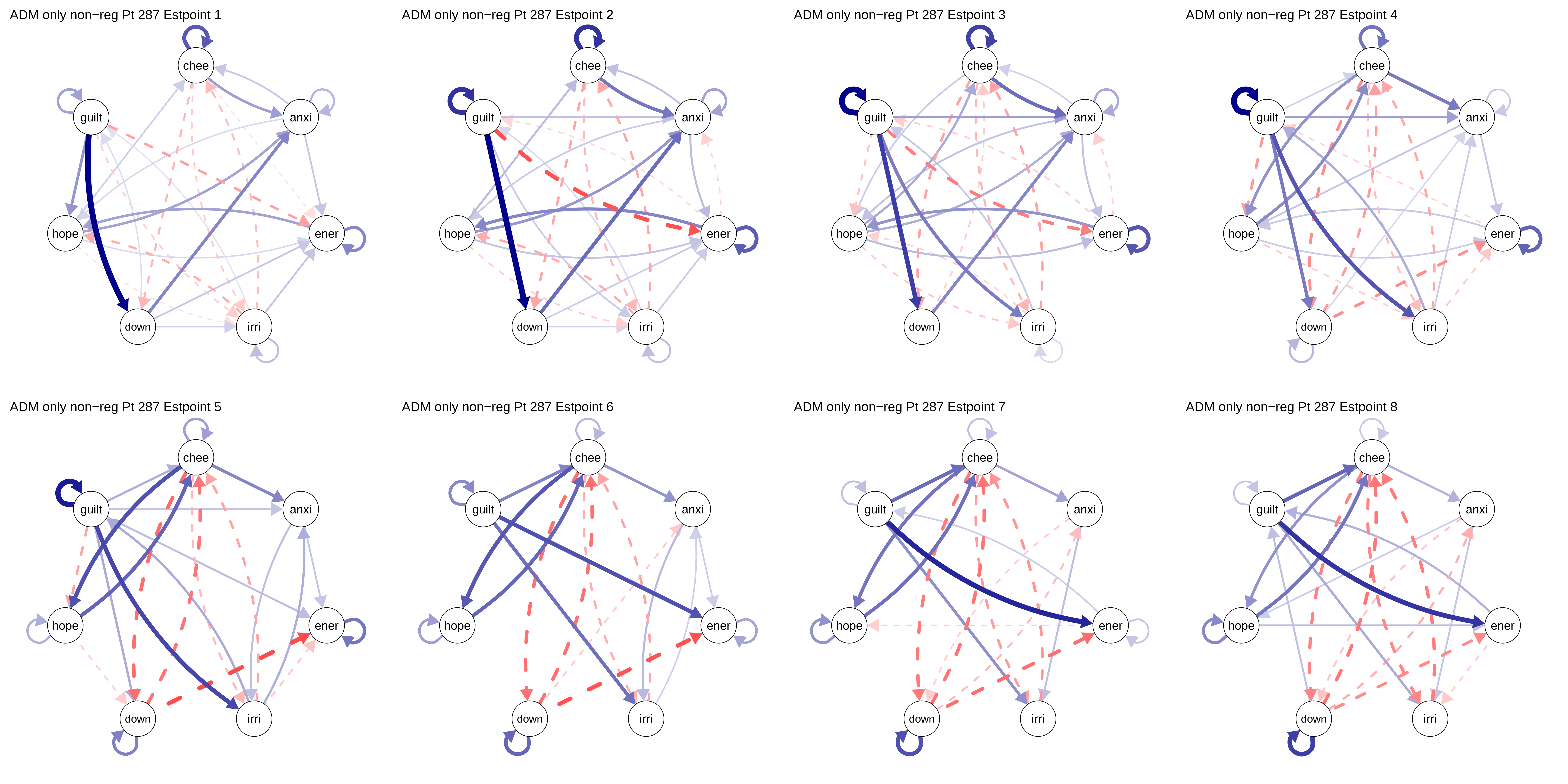
ADM only non−reg Pt 287 Estpoint 1
chee
guilt
anxi
hope
ener
down
irri
ADM only non−reg Pt 287 Estpoint 2
chee
guilt
anxi
hope
ener
down
irri
ADM only non−reg Pt 287 Estpoint 3
chee
guilt
anxi
hope
ener
down
irri
ADM only non−reg Pt 287 Estpoint 4
chee
guilt
anxi
hope
ener
down
irri
ADM only non−reg Pt 287 Estpoint 5
chee
guilt
anxi
hope
ener
down
irri
ADM only non−reg Pt 287 Estpoint 6
chee
guilt
anxi
hope
ener
down
irri
ADM only non−reg Pt 287 Estpoint 7
chee
guilt
anxi
hope
ener
down
irri
ADM only non−reg Pt 287 Estpoint 8
chee
guilt
anxi
hope
ener
down
irri

ADM only non−reg Pt 233 Estpoint 1

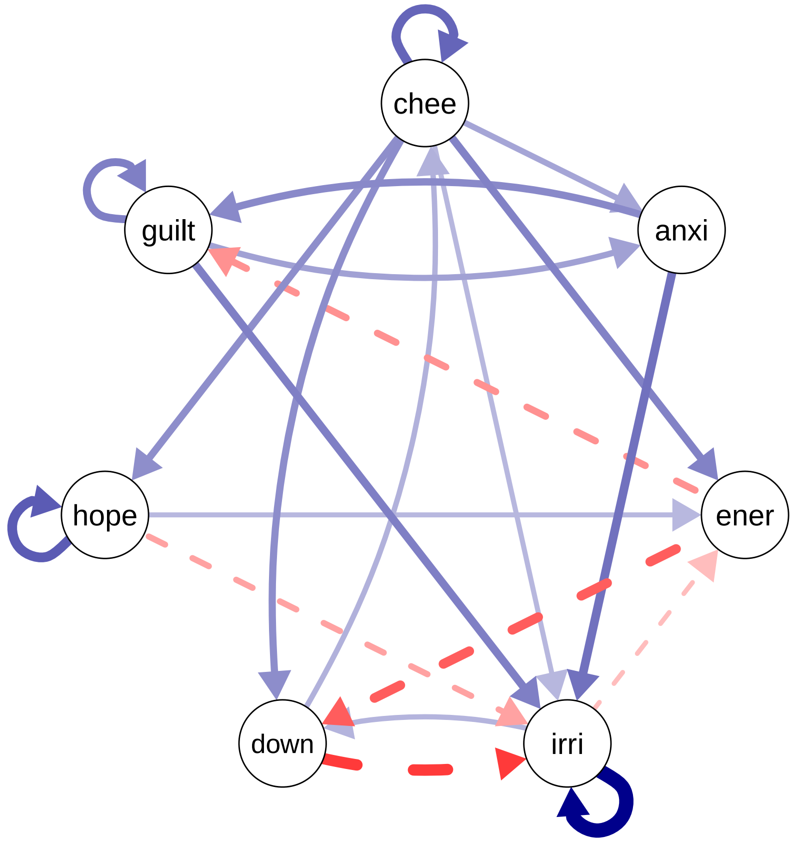

ADM only non−reg Pt 233 Estpoint 2

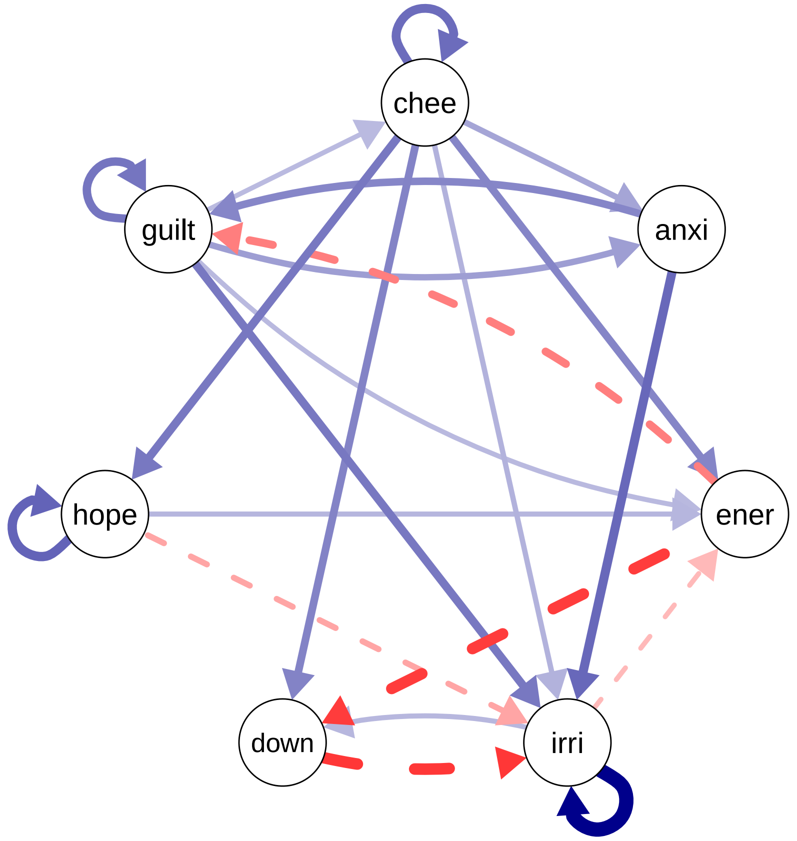

ADM only non−reg Pt 233 Estpoint 3

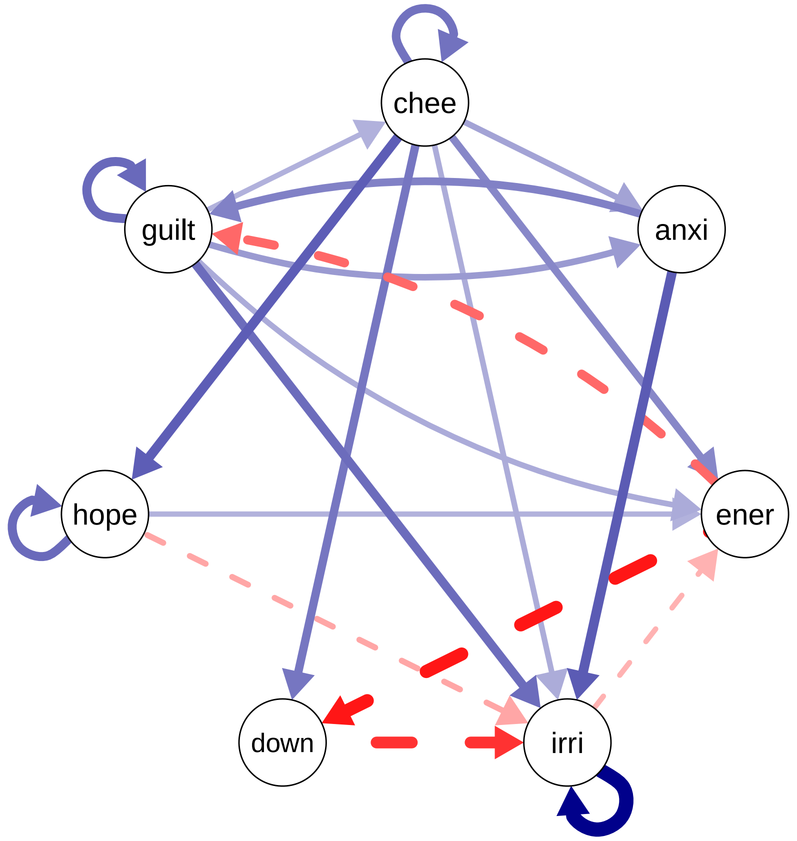

ADM only non−reg Pt 233 Estpoint 4

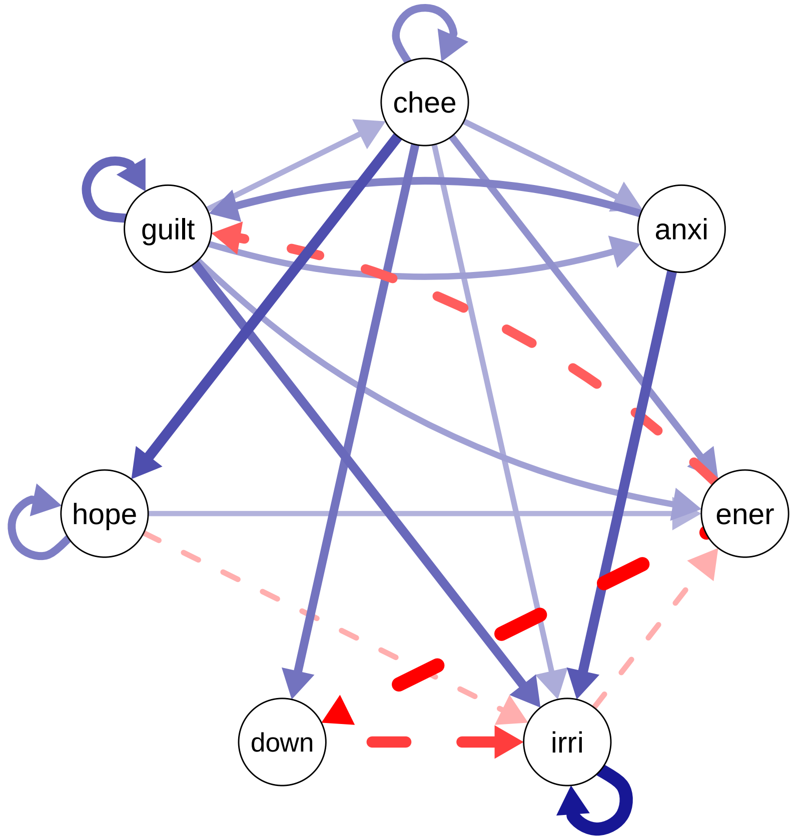

ADM only non−reg Pt 233 Estpoint 5

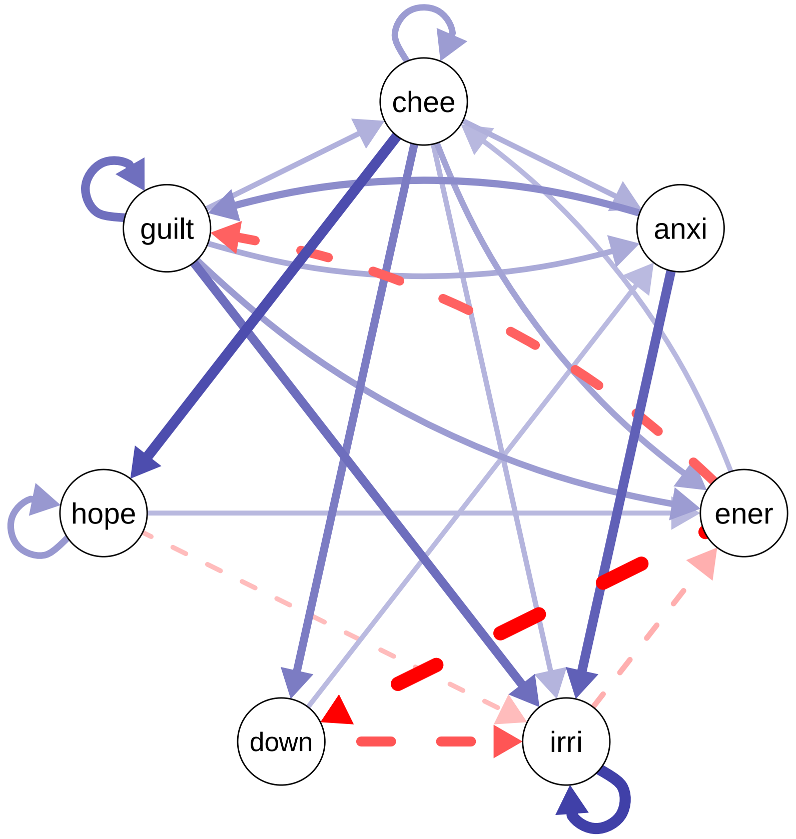

ADM only non−reg Pt 233 Estpoint 6

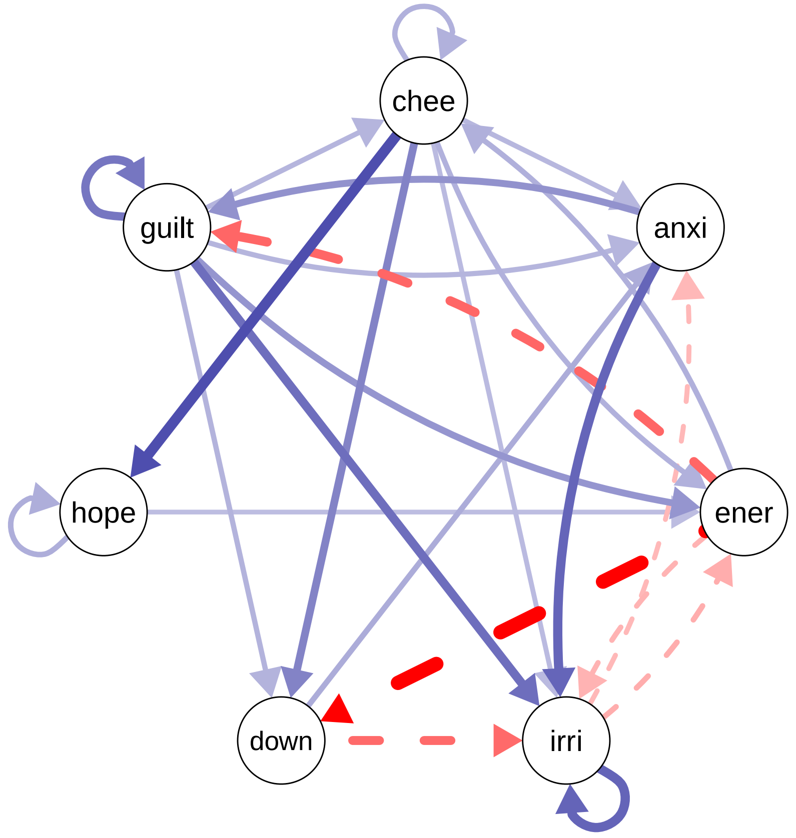

ADM only non−reg Pt 233 Estpoint 7

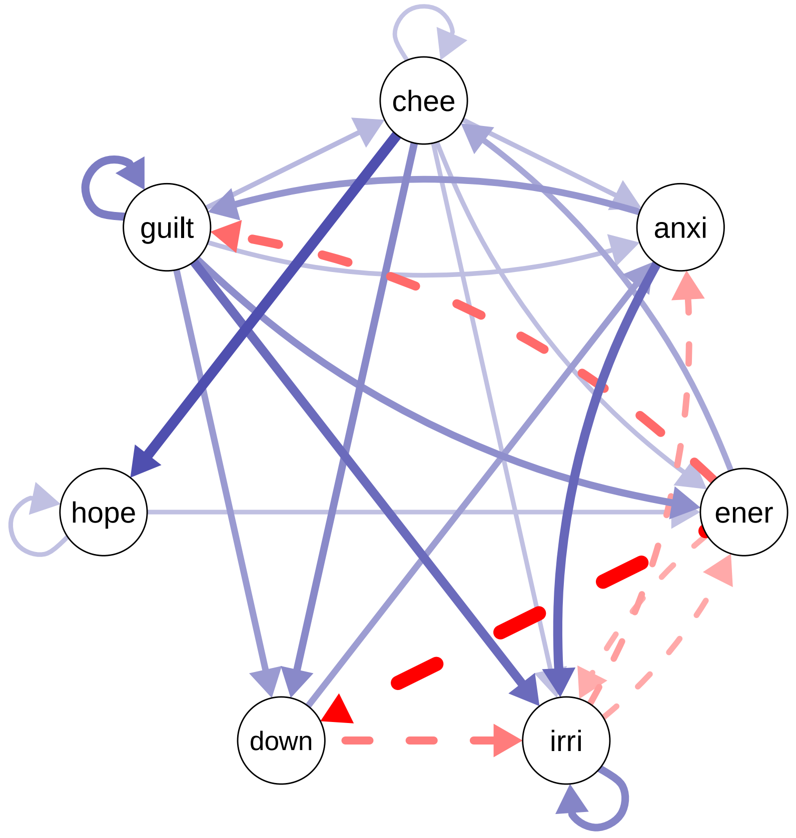

ADM only non−reg Pt 233 Estpoint 8

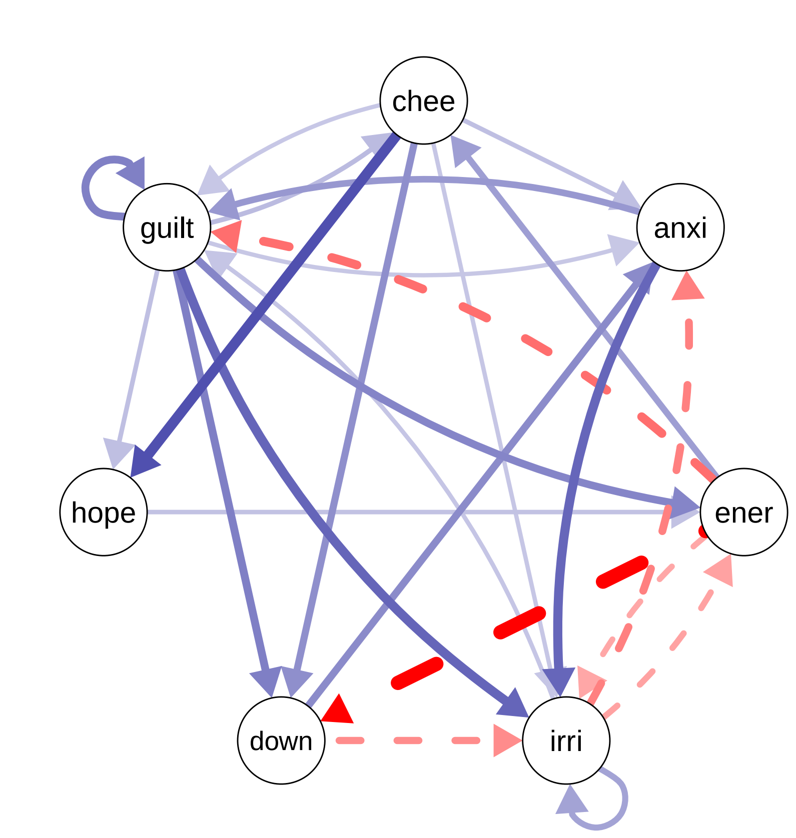

ADM only non−reg Pt 243 Estpoint 1

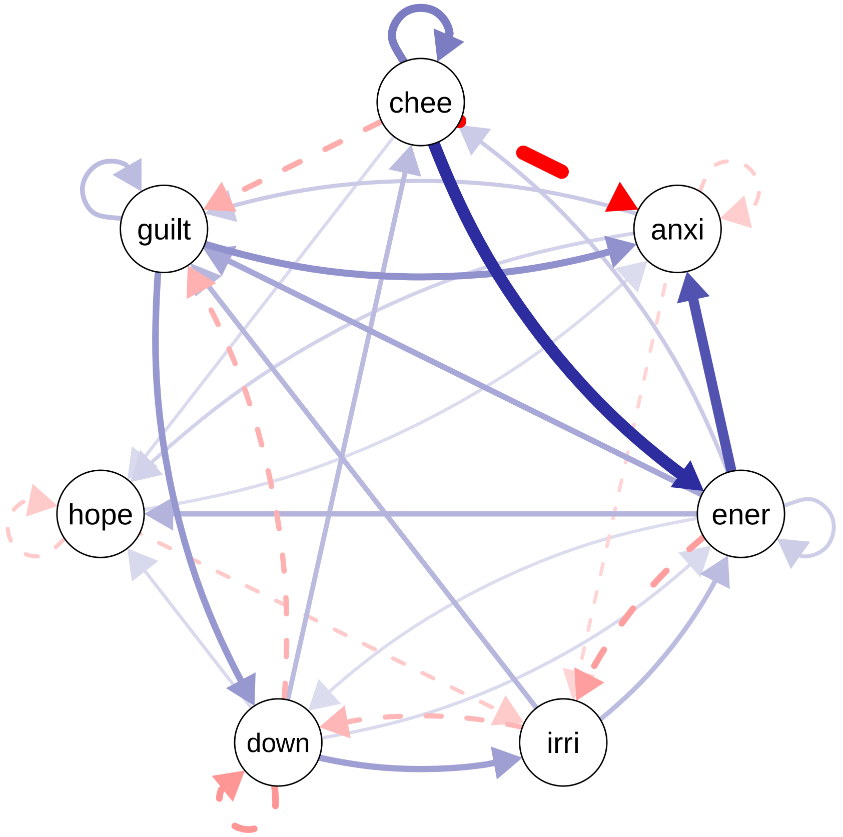

ADM only non−reg Pt 243 Estpoint 2

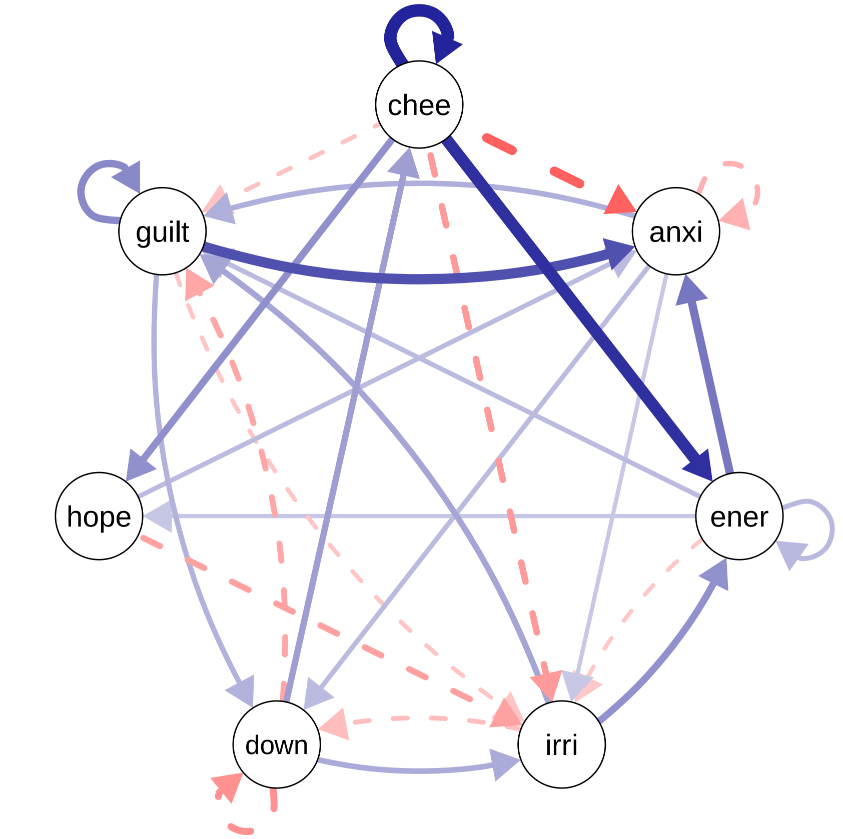

ADM only non−reg Pt 243 Estpoint 3

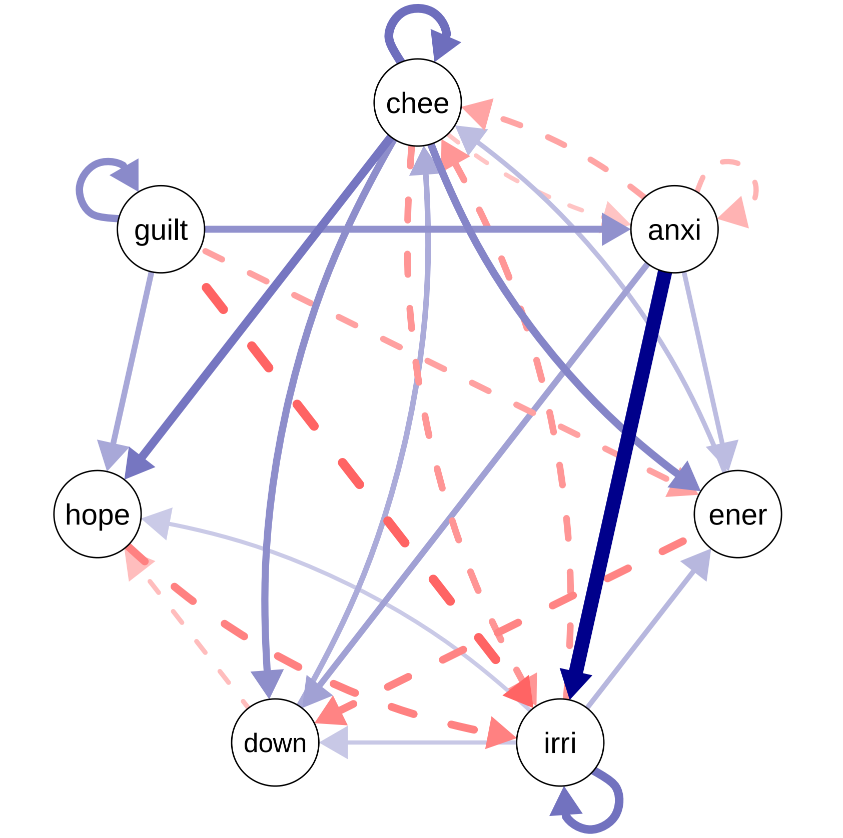

ADM only non−reg Pt 243 Estpoint 4

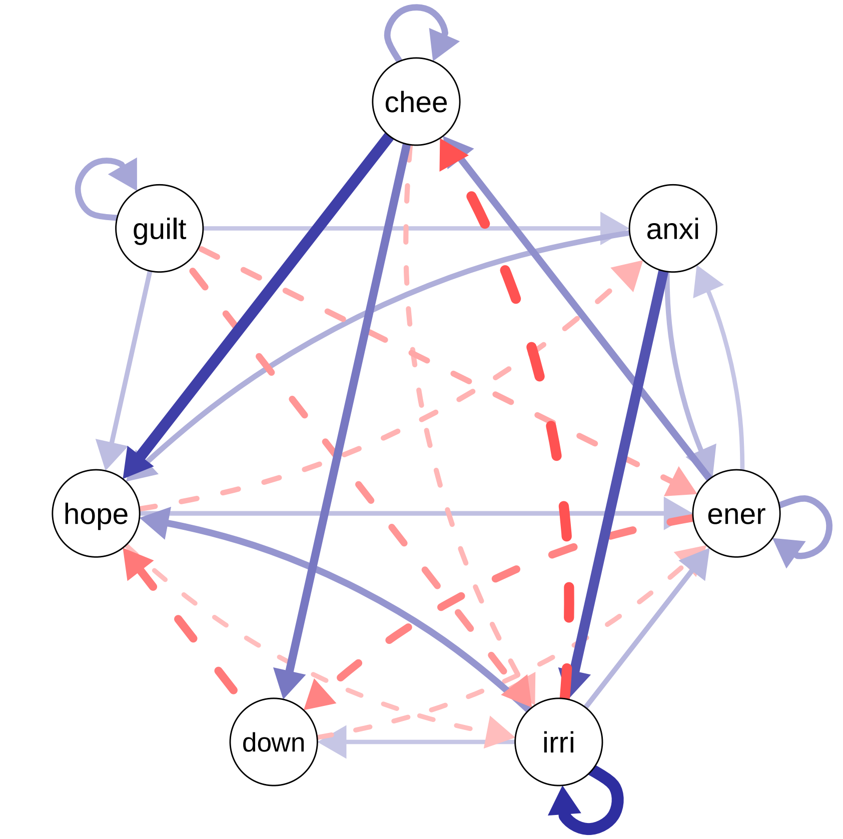

ADM only non−reg Pt 243 Estpoint 5

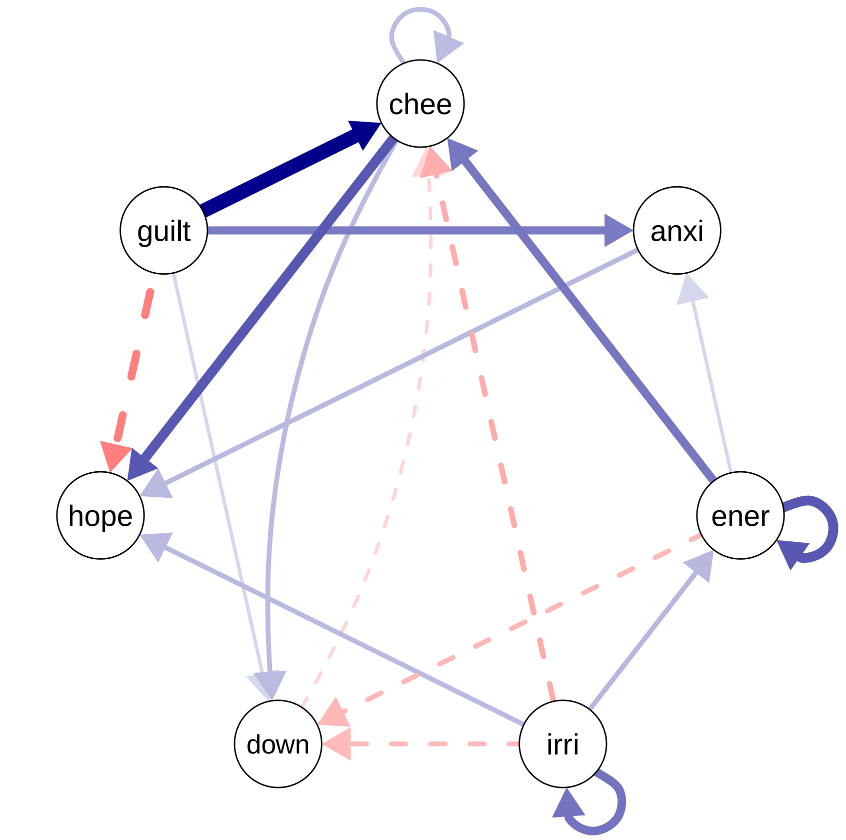

ADM only non−reg Pt 243 Estpoint 6

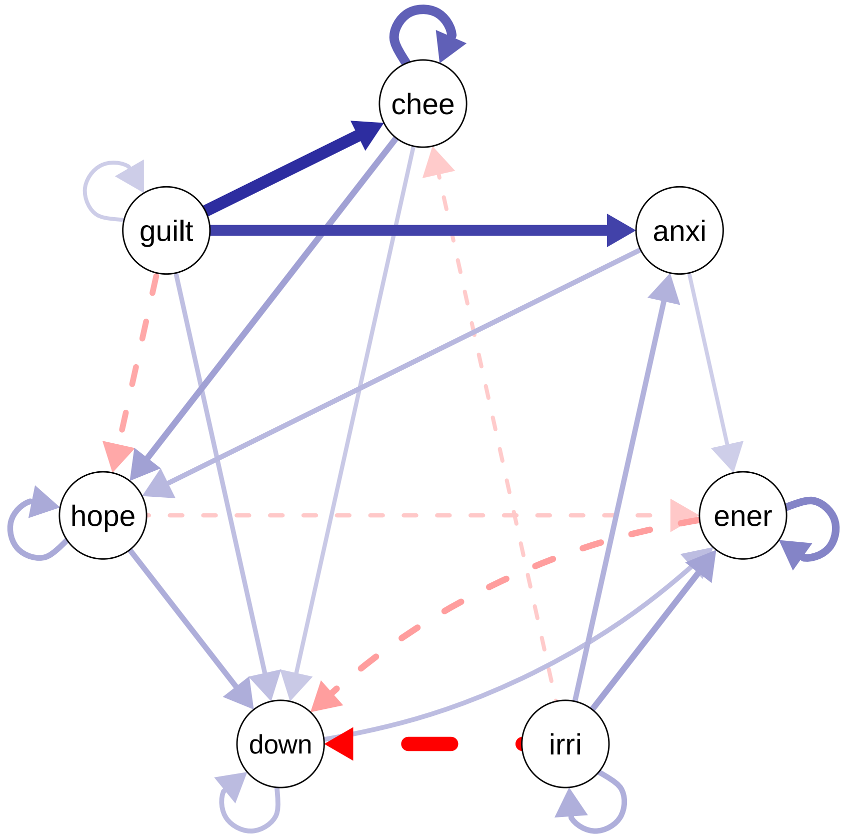

ADM only non−reg Pt 243 Estpoint 7

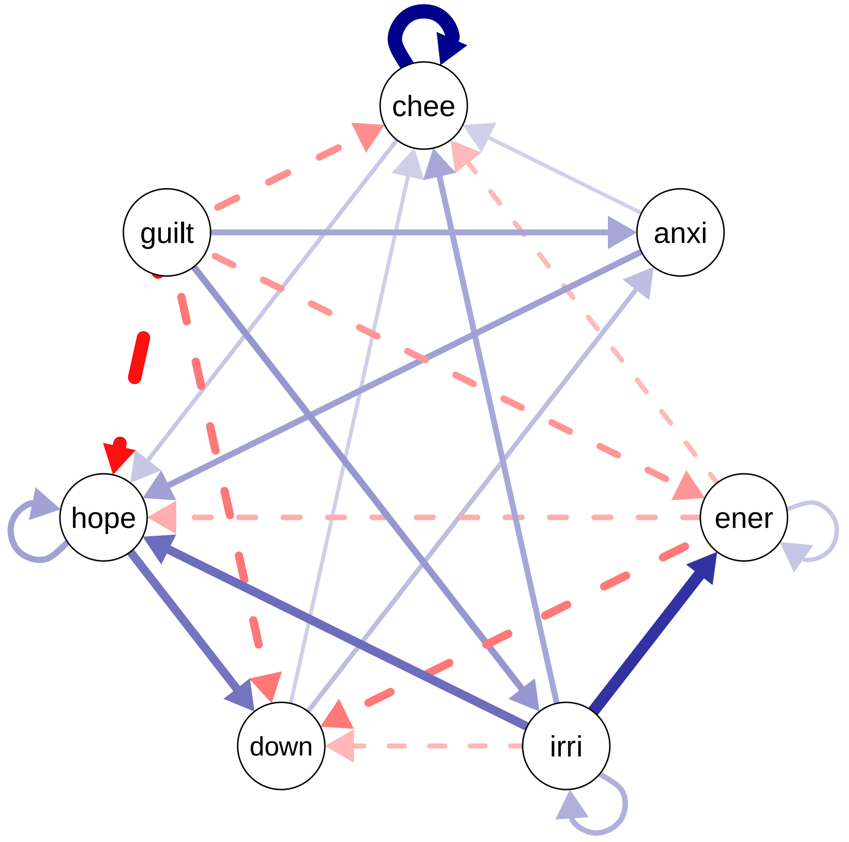

ADM only non−reg Pt 243 Estpoint 8

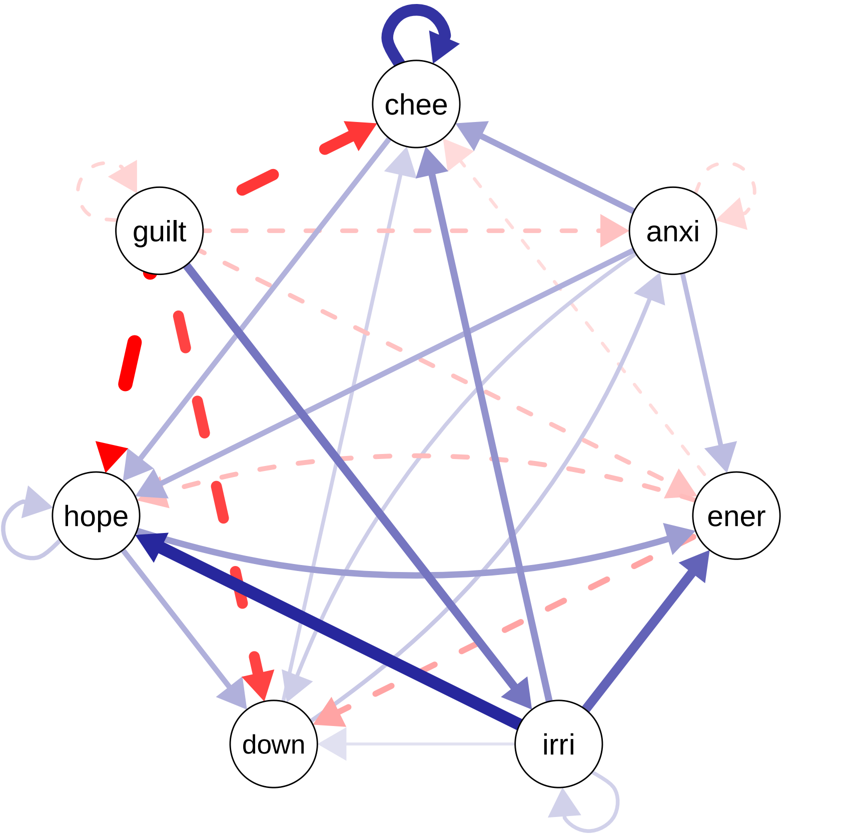

ADM only non−reg Pt 239 Estpoint 1

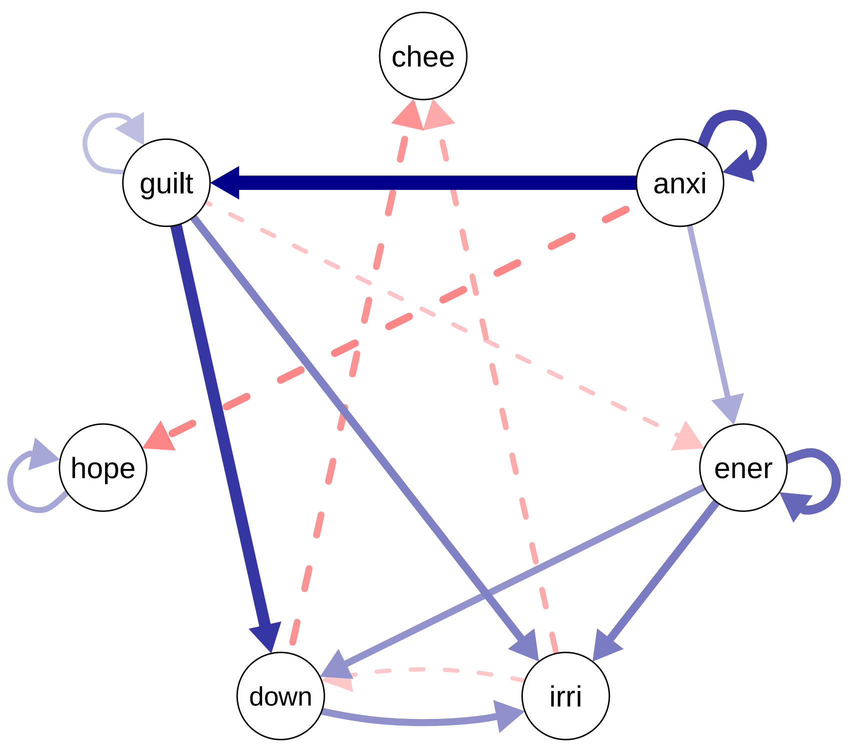

ADM only non−reg Pt 239 Estpoint 2

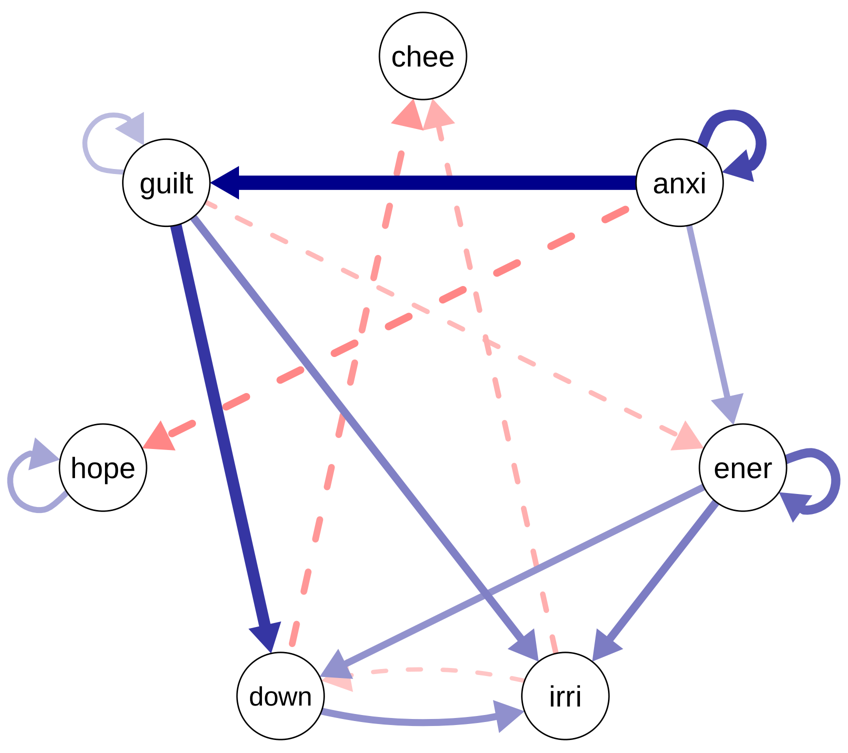

ADM only non−reg Pt 239 Estpoint 3

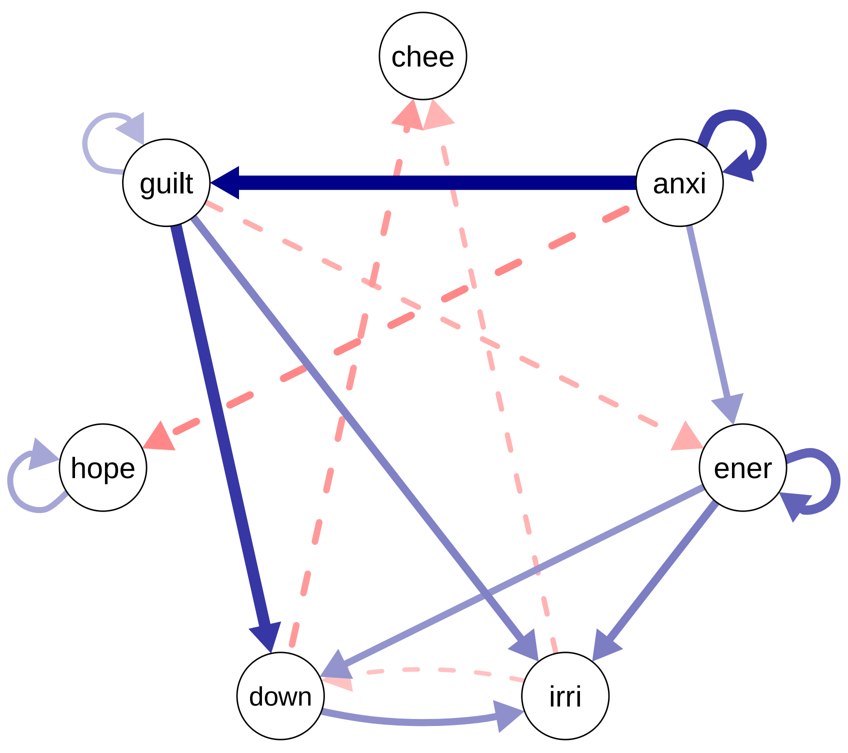

ADM only non−reg Pt 239 Estpoint 4

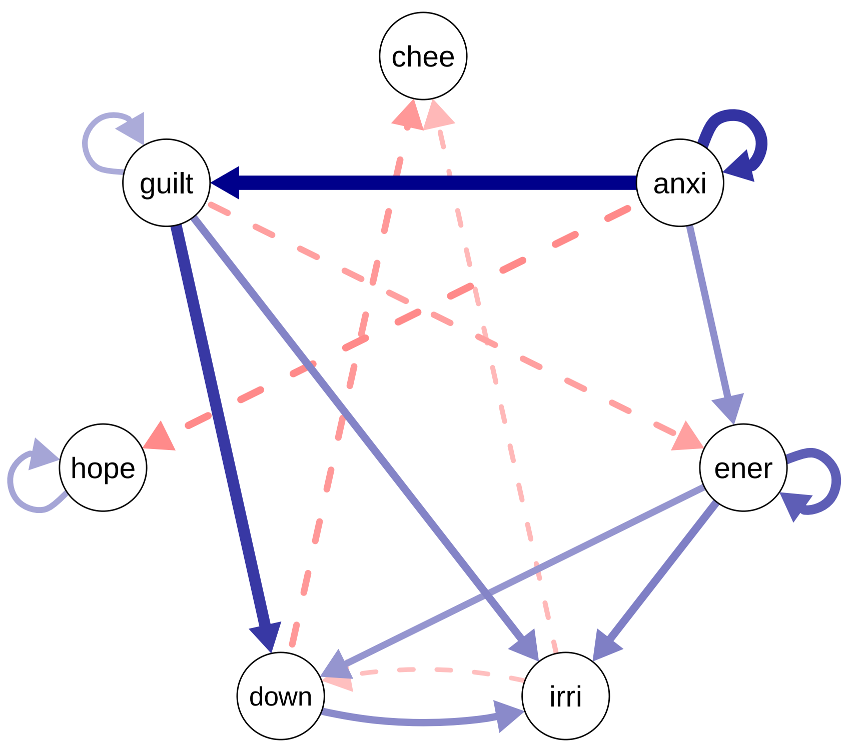

ADM only non−reg Pt 239 Estpoint 5

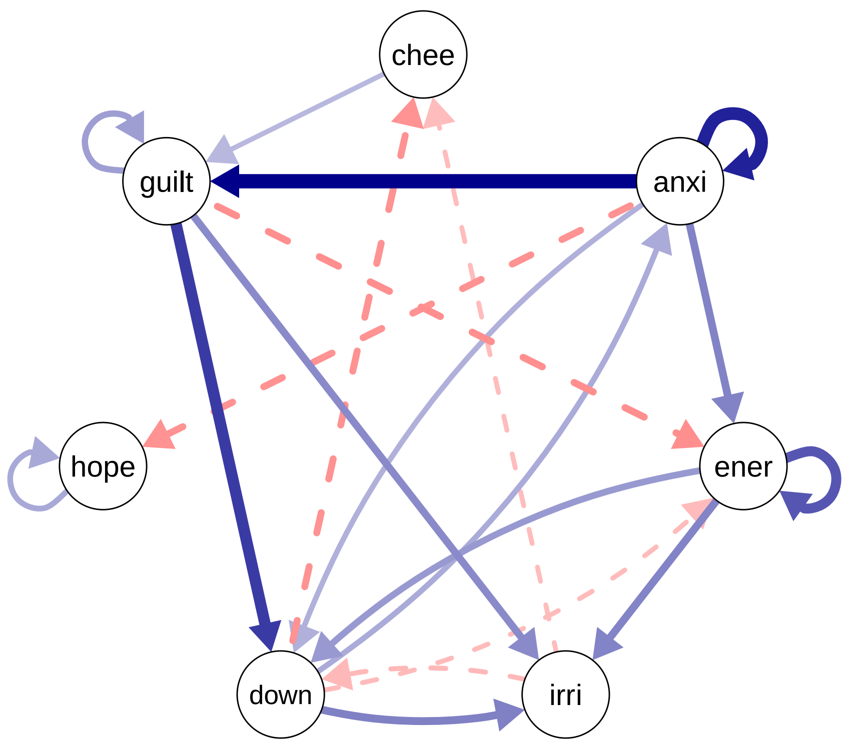

ADM only non−reg Pt 239 Estpoint 6

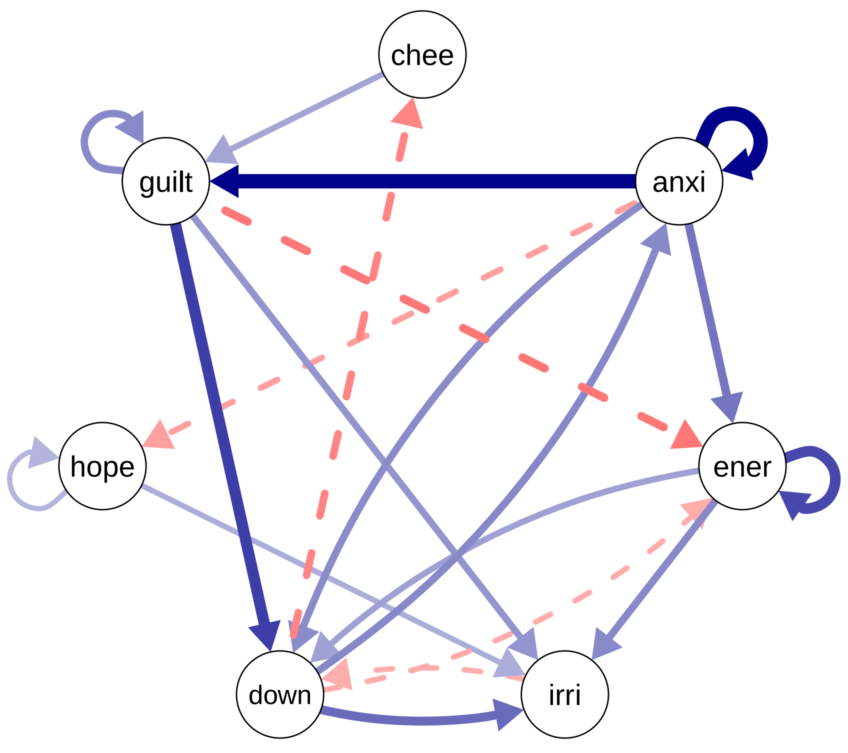

ADM only non−reg Pt 239 Estpoint 7

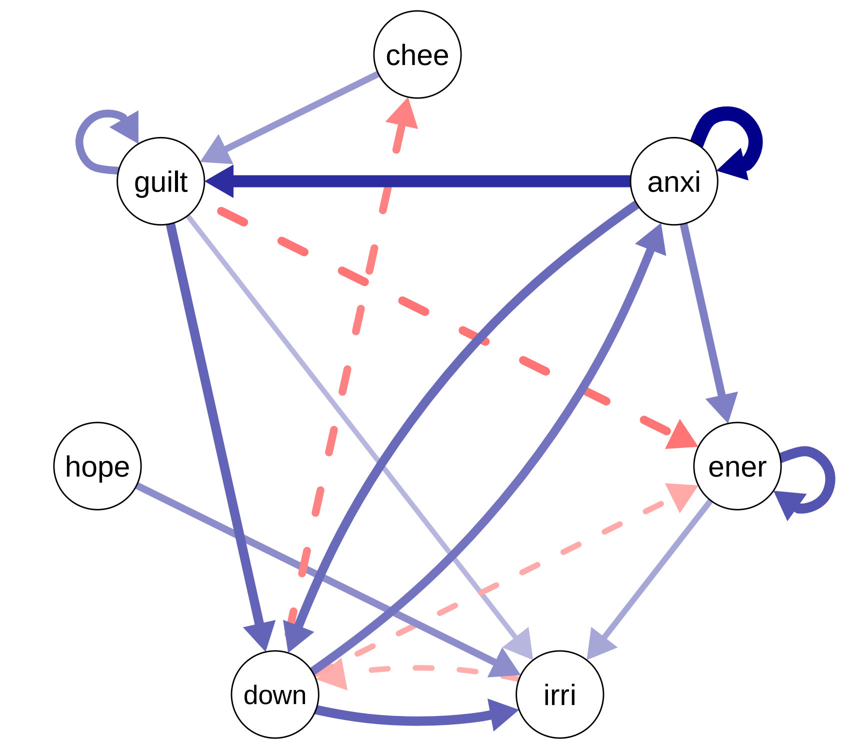

ADM only non−reg Pt 239 Estpoint 8

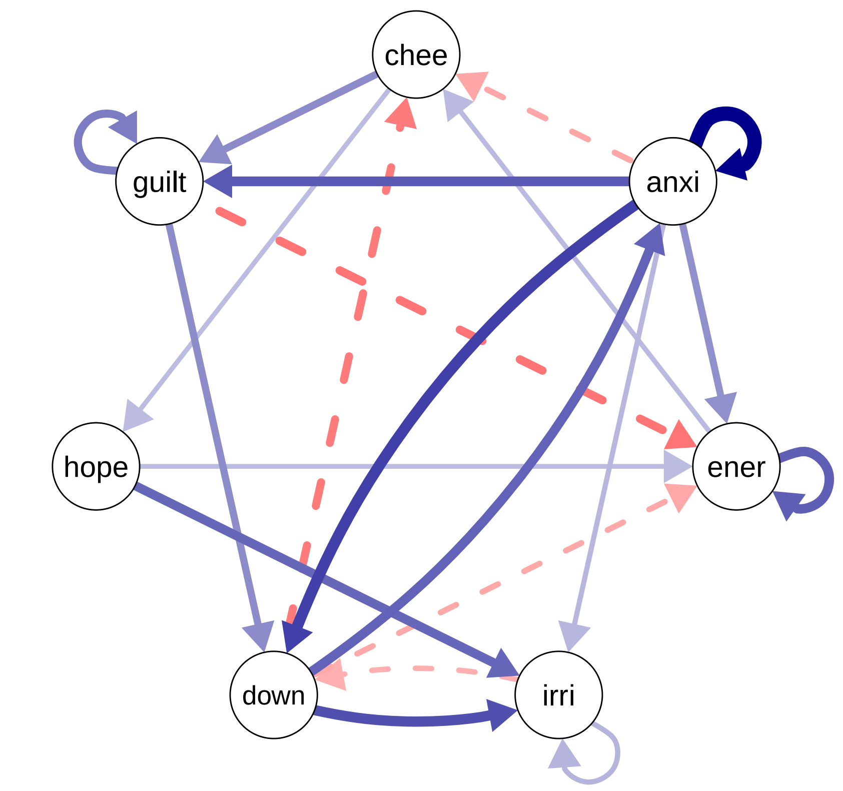

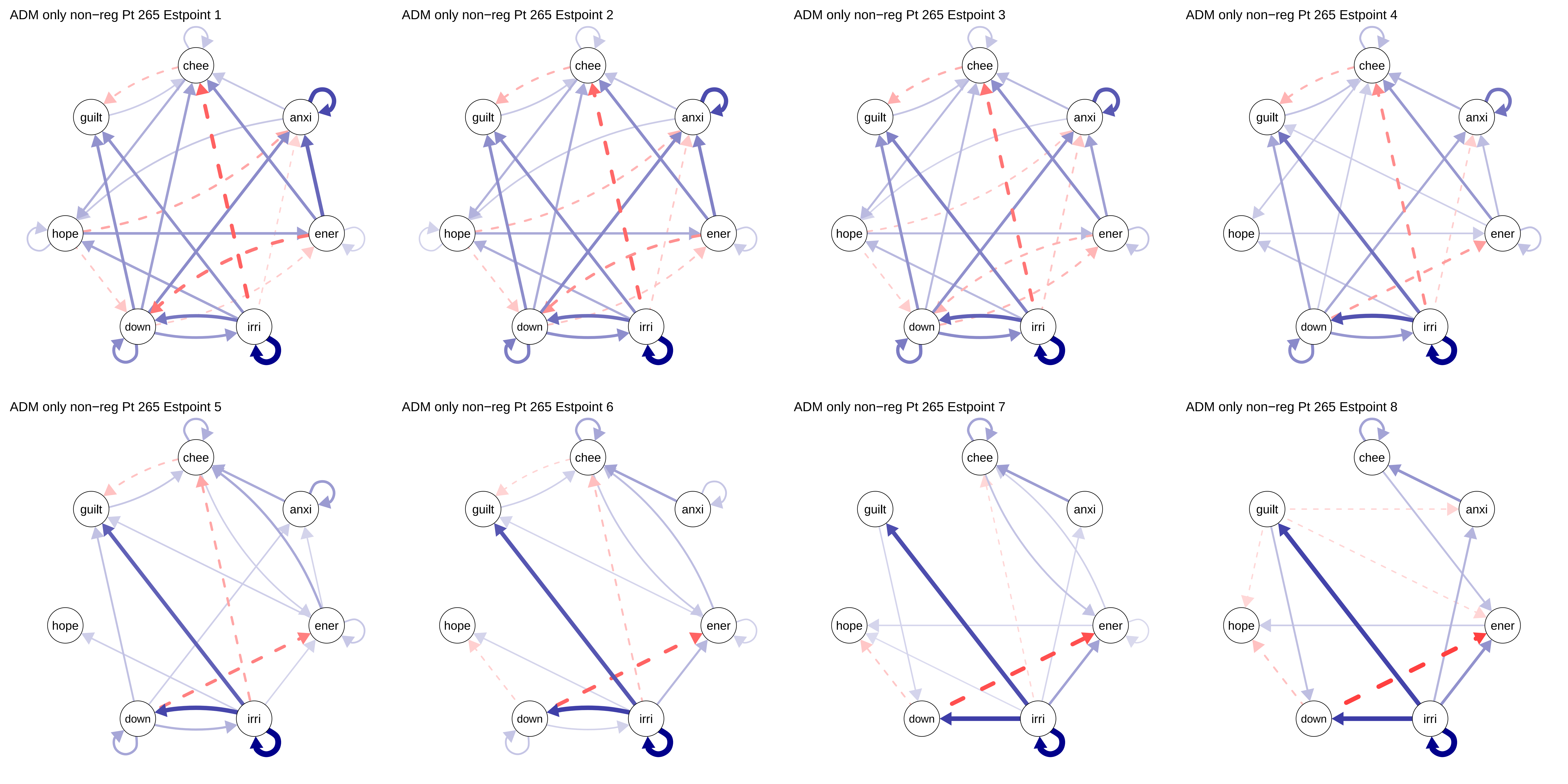
ADM only non−reg Pt 265 Estpoint 1
chee guilt anxi hope ener down irri
ADM only non−reg Pt 265 Estpoint 2
chee guilt anxi hope ener down irri
ADM only non−reg Pt 265 Estpoint 3
chee guilt anxi hope ener down irri
ADM only non−reg Pt 265 Estpoint 4
chee guilt anxi hope ener down irri
ADM only non−reg Pt 265 Estpoint 5
chee guilt anxi hope ener down irri
ADM only non−reg Pt 265 Estpoint 6
chee guilt anxi hope ener down irri
ADM only non−reg Pt 265 Estpoint 7
chee guilt anxi hope ener down irri
ADM only non−reg Pt 265 Estpoint 8
chee guilt anxi hope ener down irri

PCT plus ADM reg Pt 288 Estpoint 1

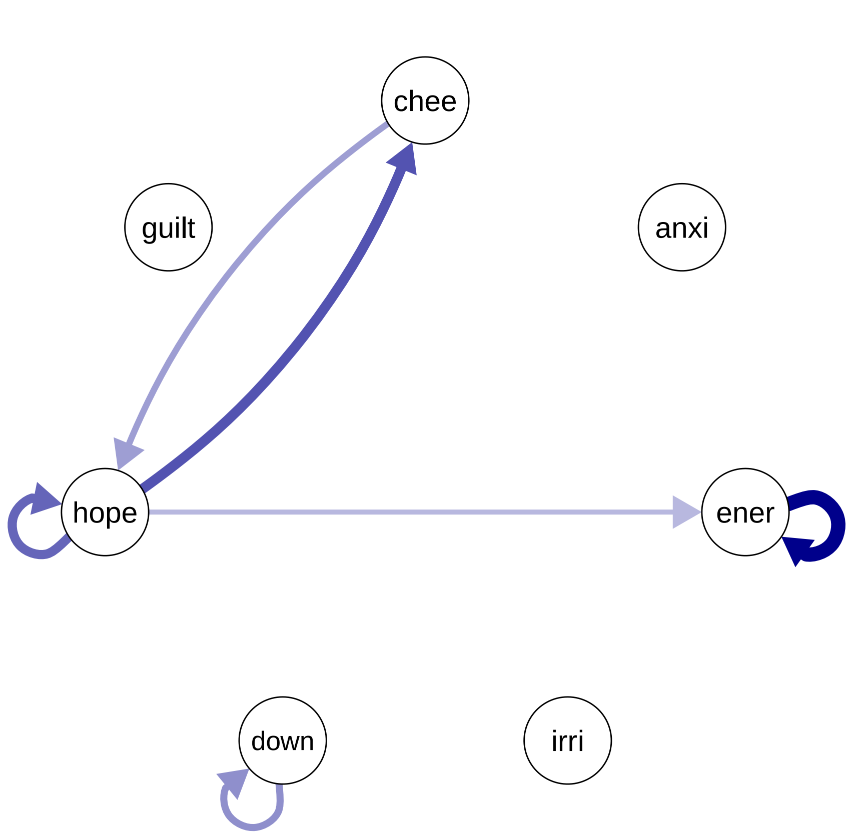

PCT plus ADM reg Pt 288 Estpoint 2

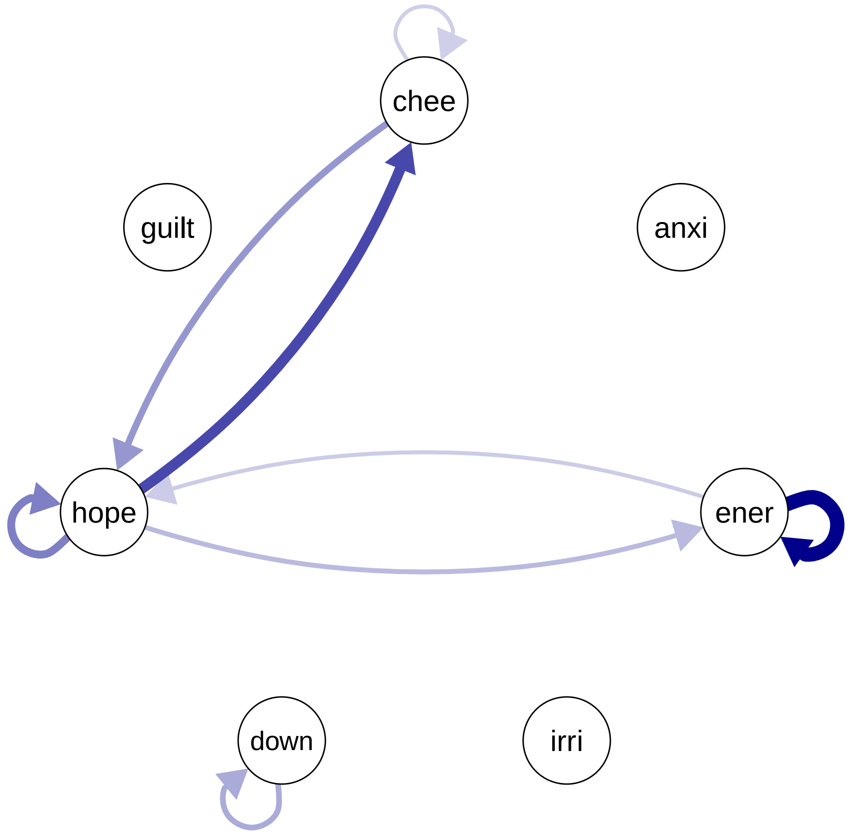

PCT plus ADM reg Pt 288 Estpoint 3

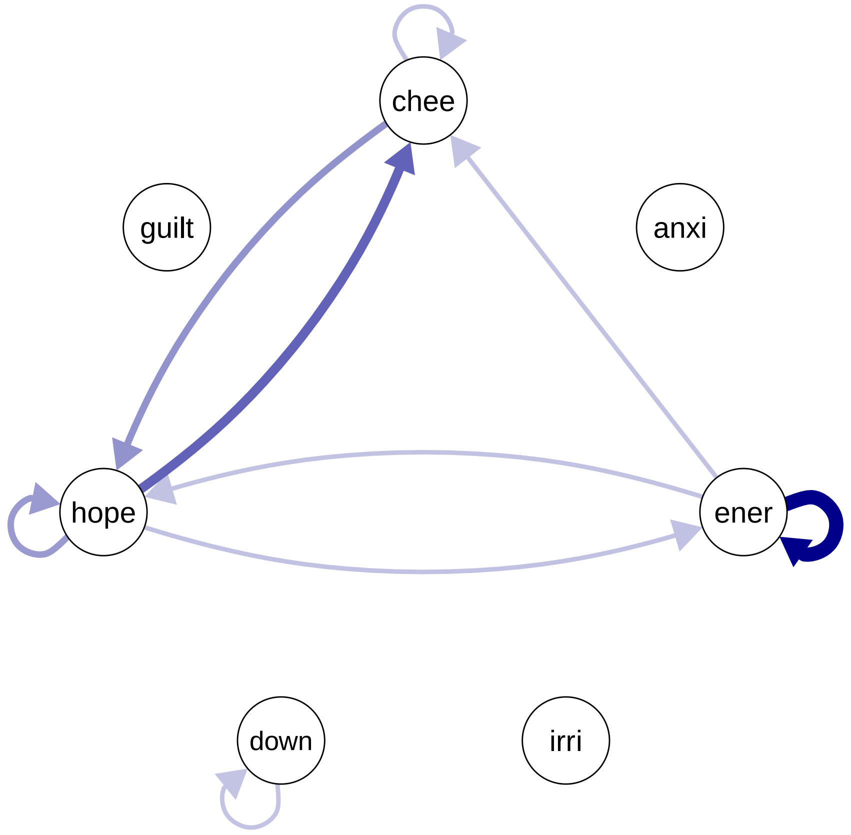

PCT plus ADM reg Pt 288 Estpoint 4

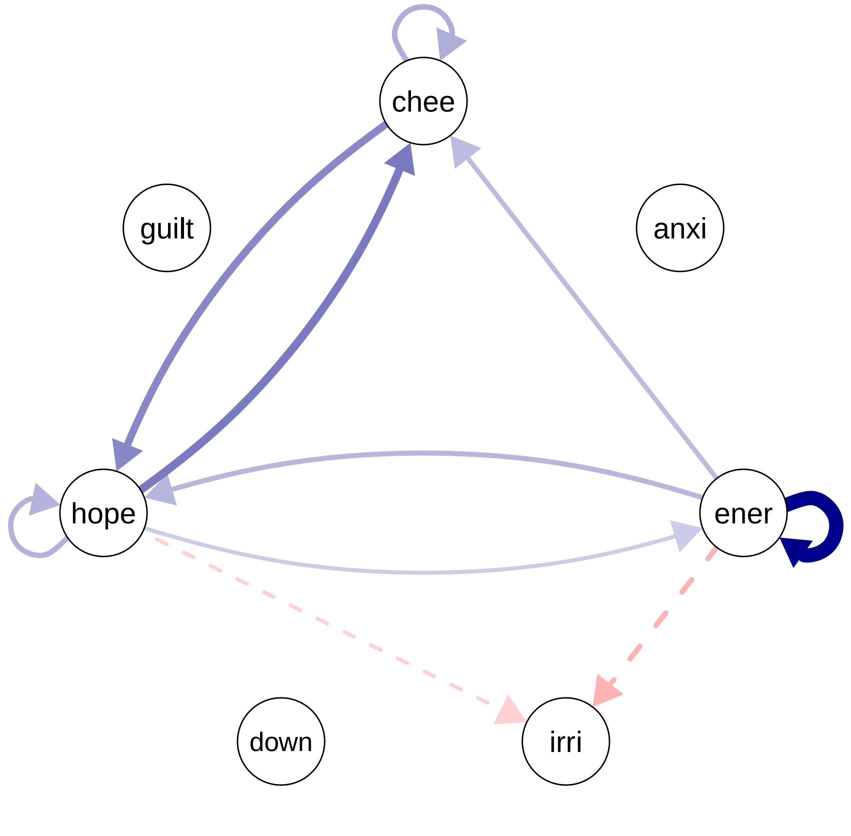

PCT plus ADM reg Pt 288 Estpoint 5

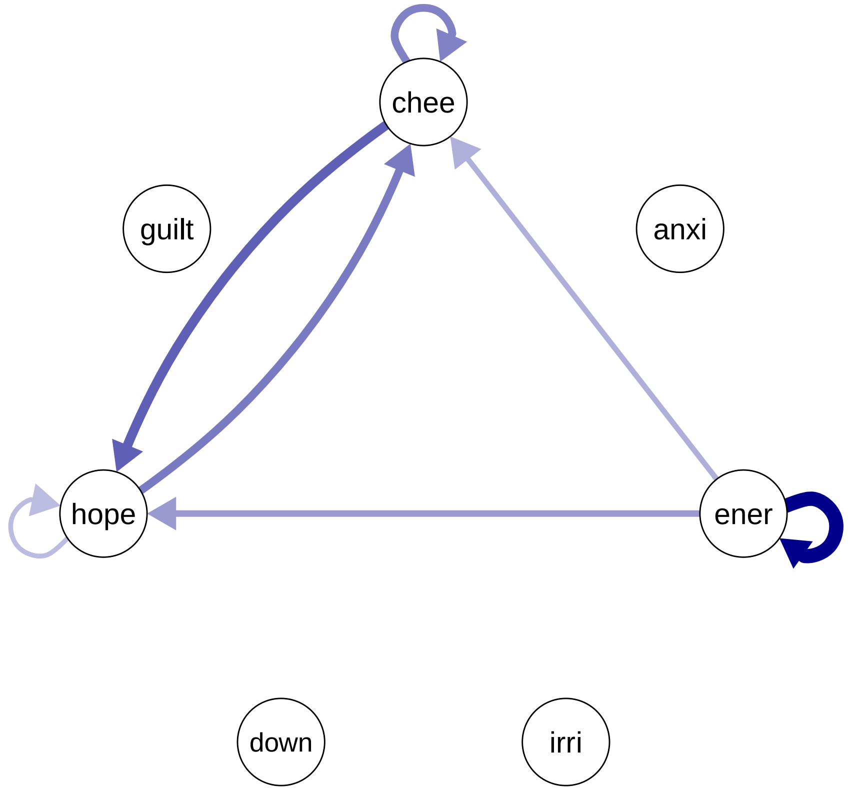

PCT plus ADM reg Pt 288 Estpoint 6

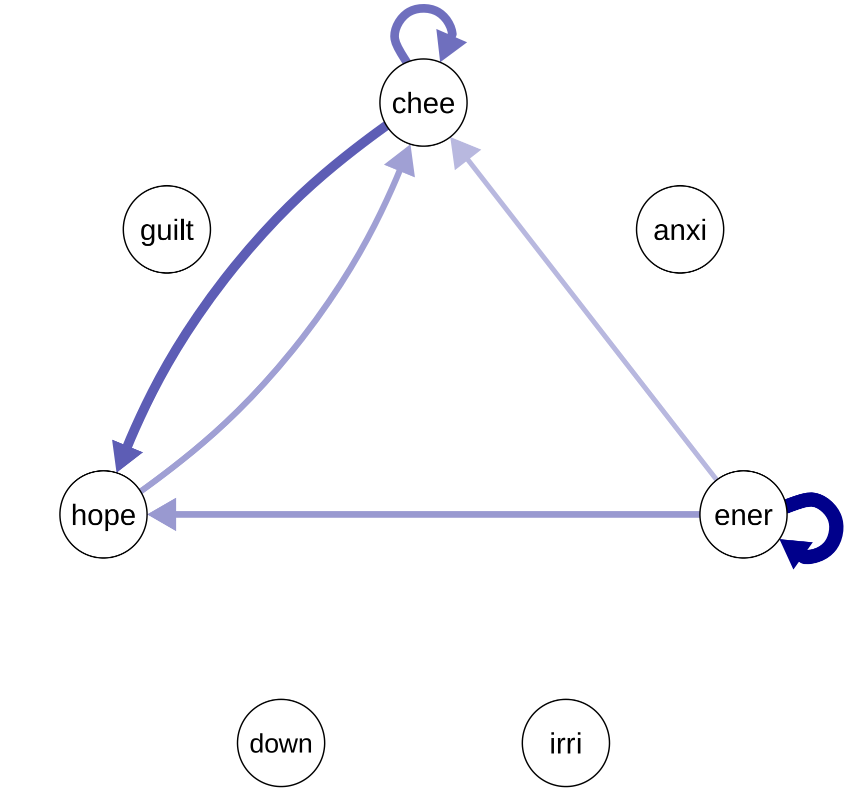

PCT plus ADM reg Pt 288 Estpoint 7

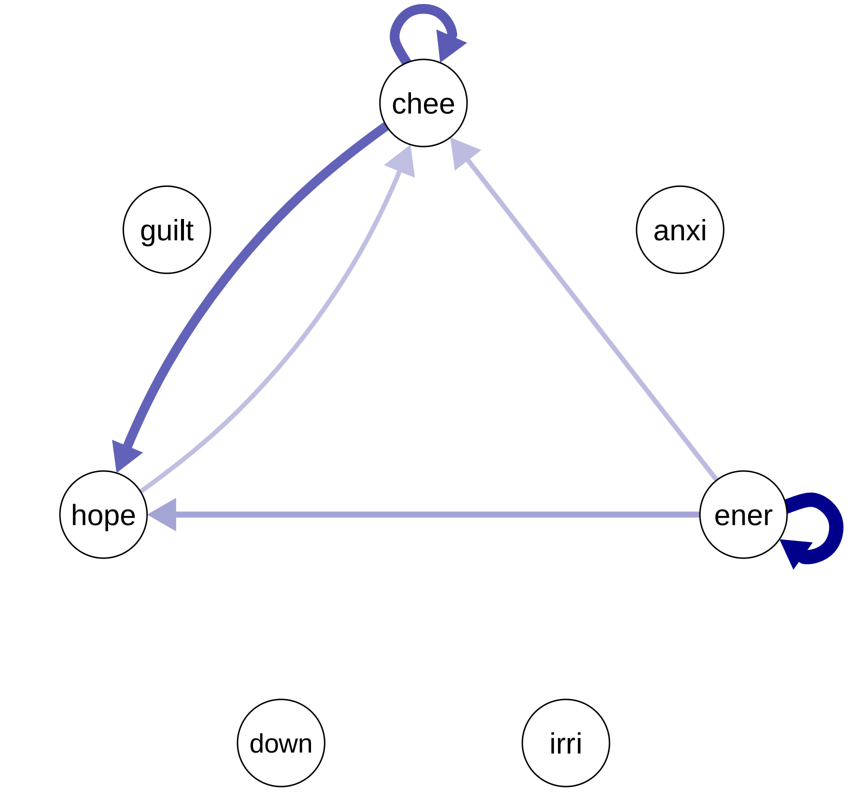

PCT plus ADM reg Pt 288 Estpoint 8

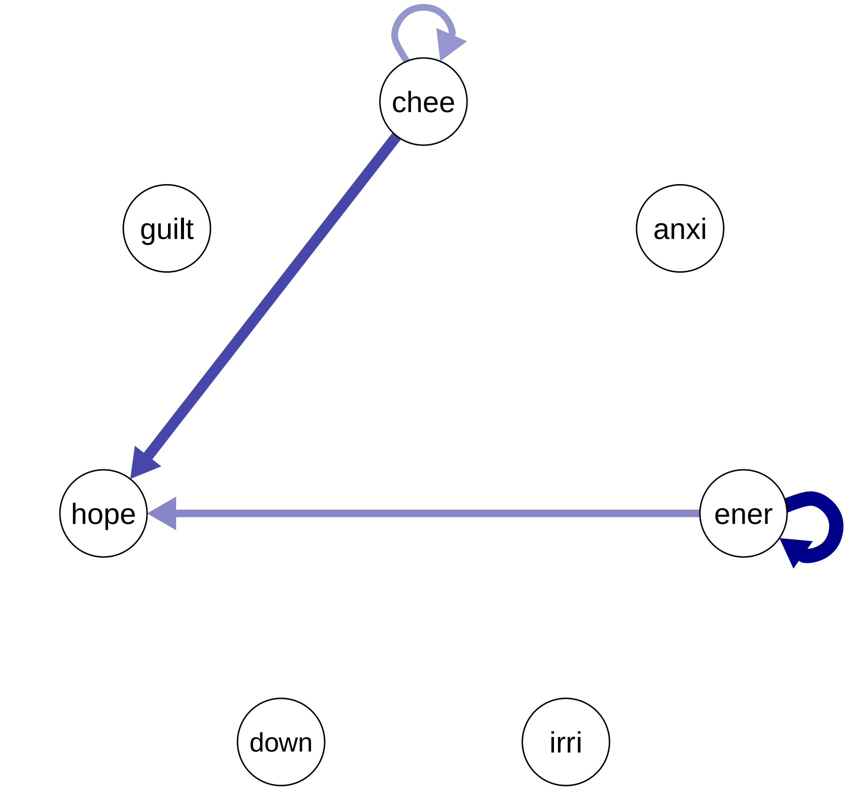

PCT plus ADM reg Pt 291 Estpoint 1

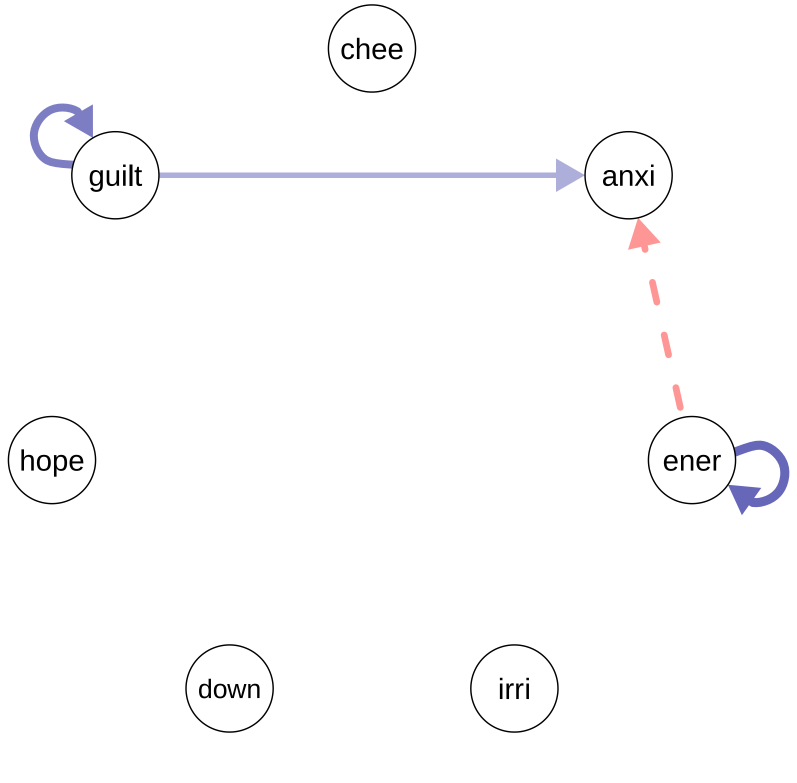

PCT plus ADM reg Pt 291 Estpoint 2

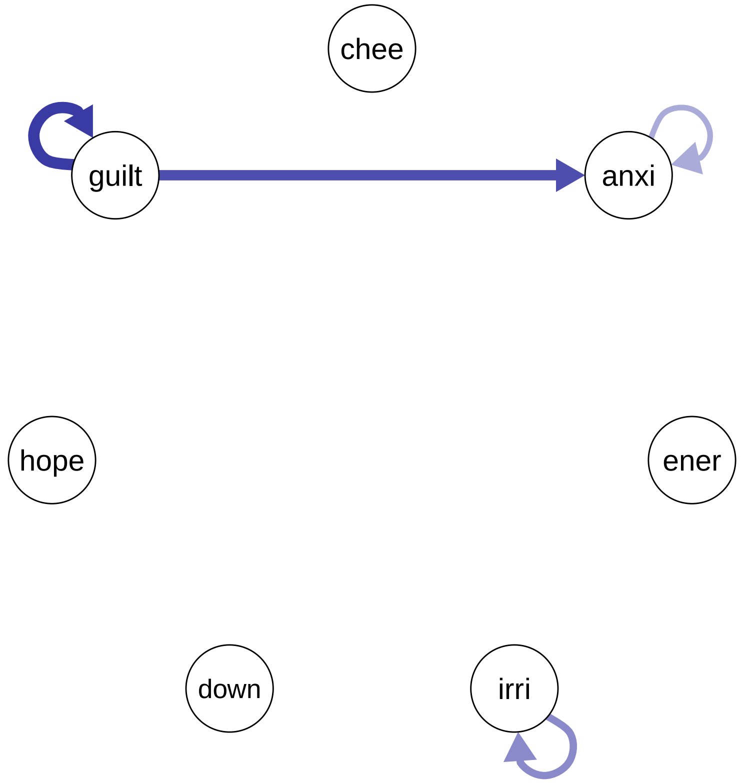

PCT plus ADM reg Pt 291 Estpoint 3

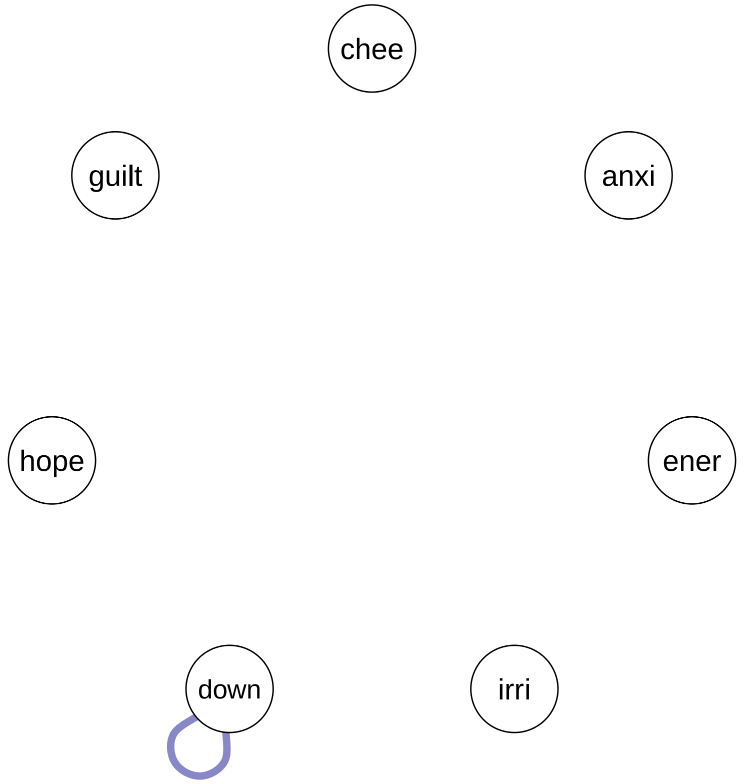

PCT plus ADM reg Pt 291 Estpoint 4

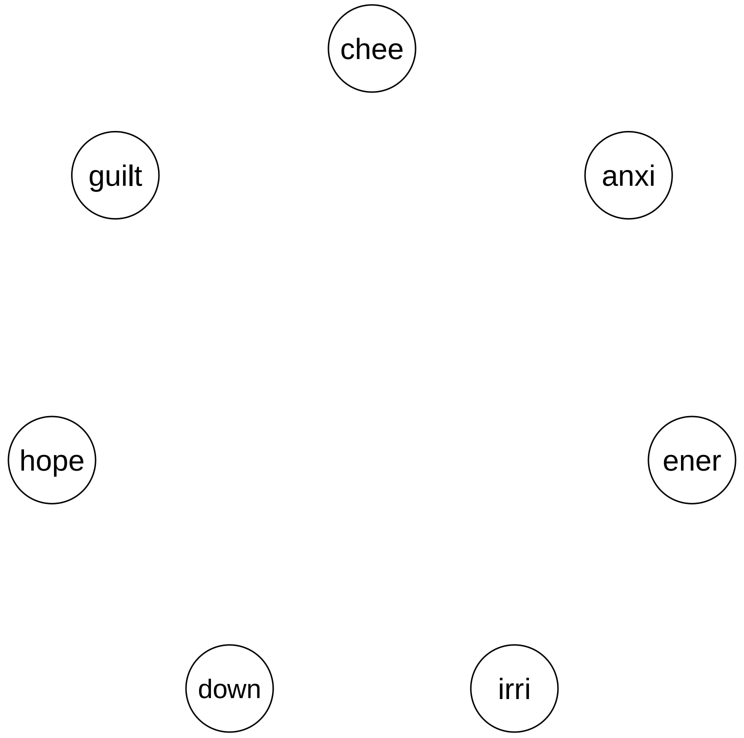

PCT plus ADM reg Pt 291 Estpoint 5

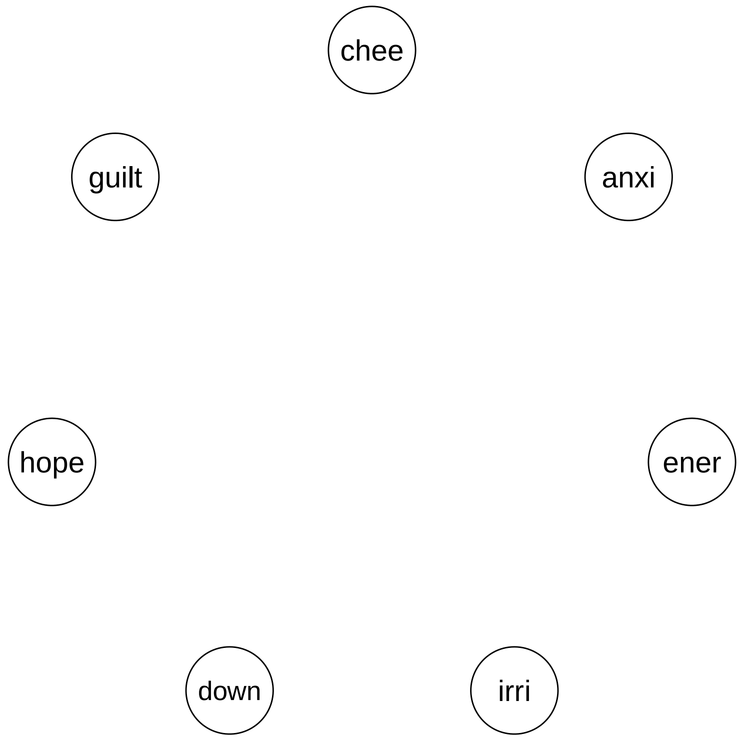

PCT plus ADM reg Pt 291 Estpoint 6

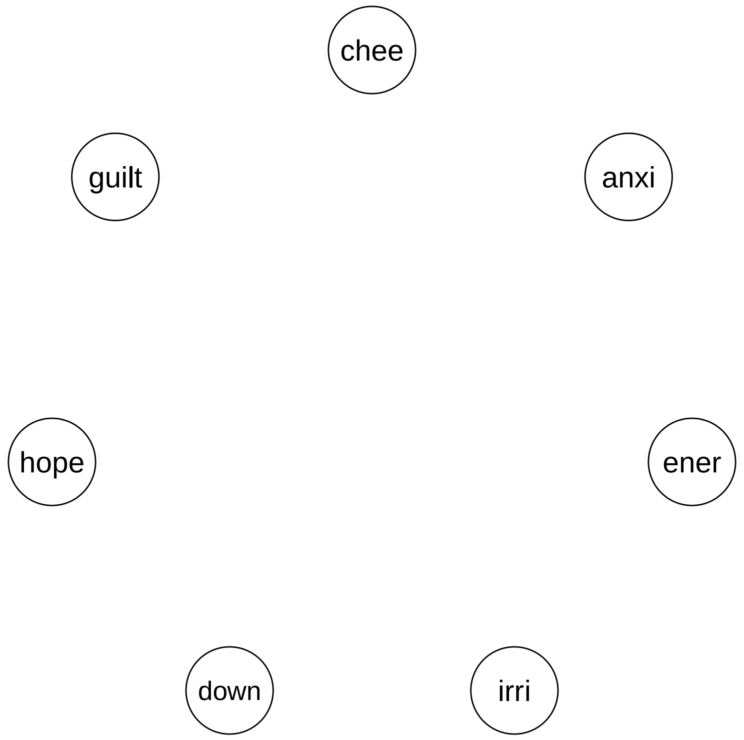

PCT plus ADM reg Pt 291 Estpoint 7

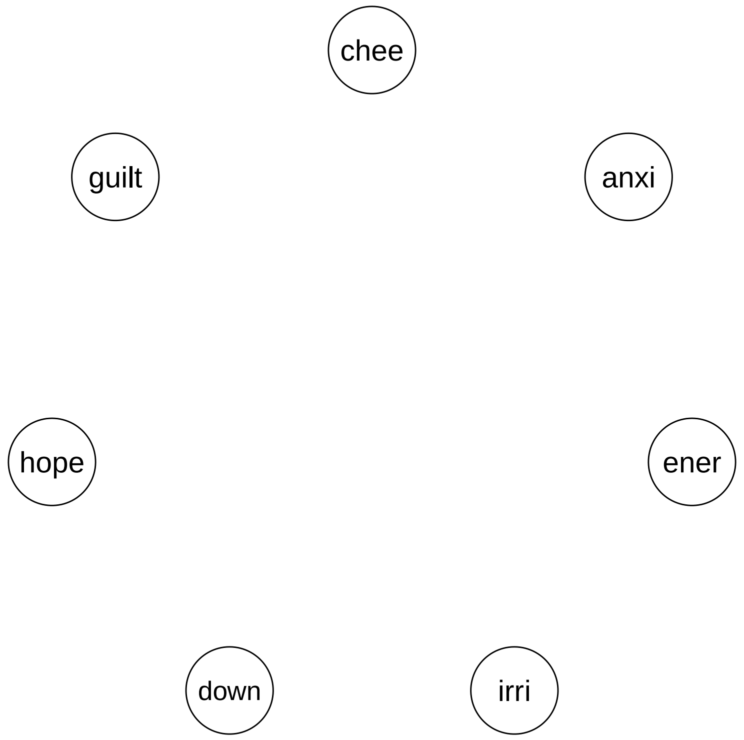

PCT plus ADM reg Pt 291 Estpoint 8

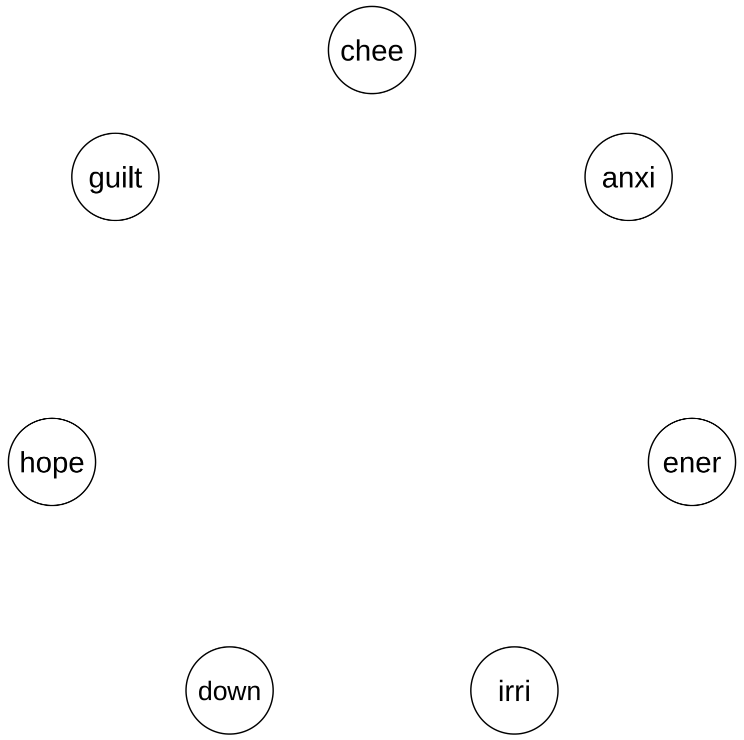

PCT plus ADM reg Pt 234 Estpoint 1

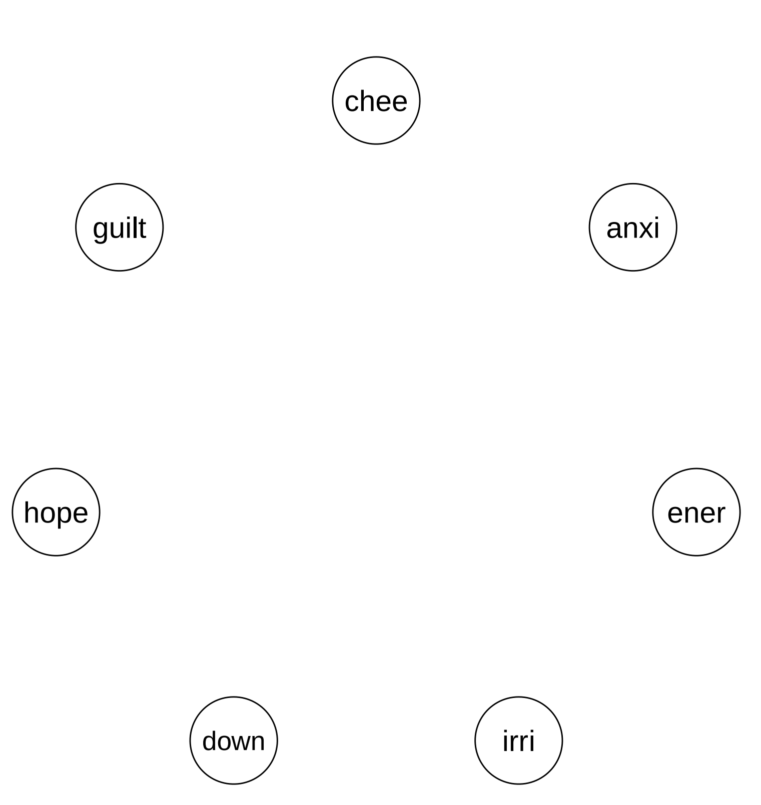

PCT plus ADM reg Pt 234 Estpoint 2

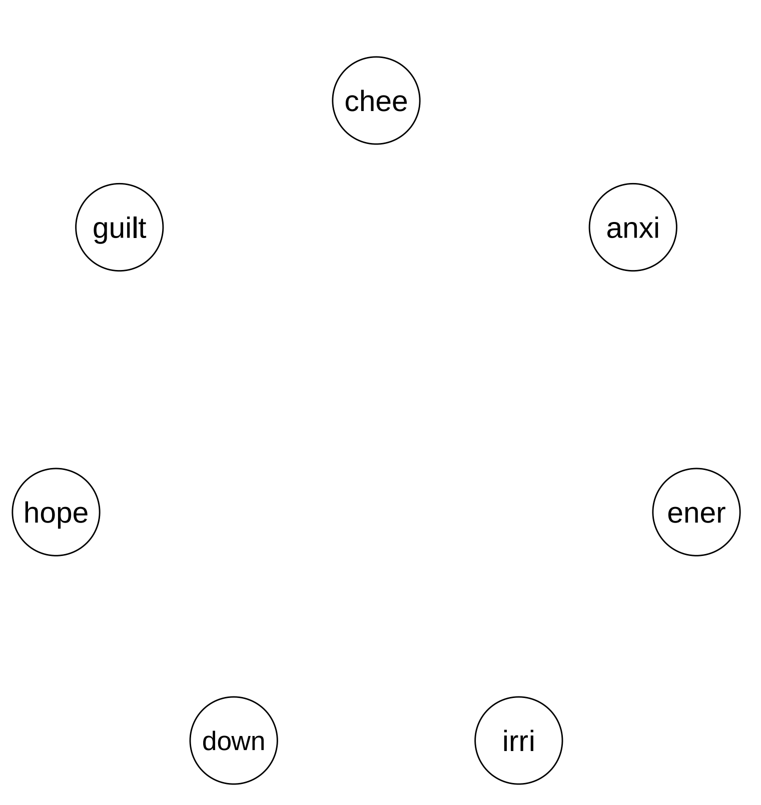

PCT plus ADM reg Pt 234 Estpoint 3

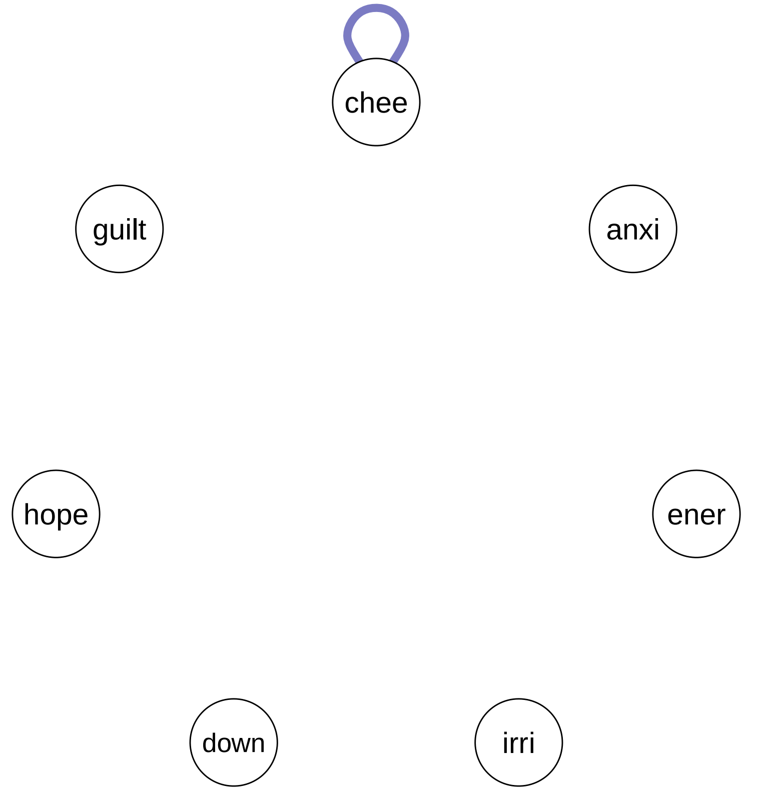

PCT plus ADM reg Pt 234 Estpoint 4

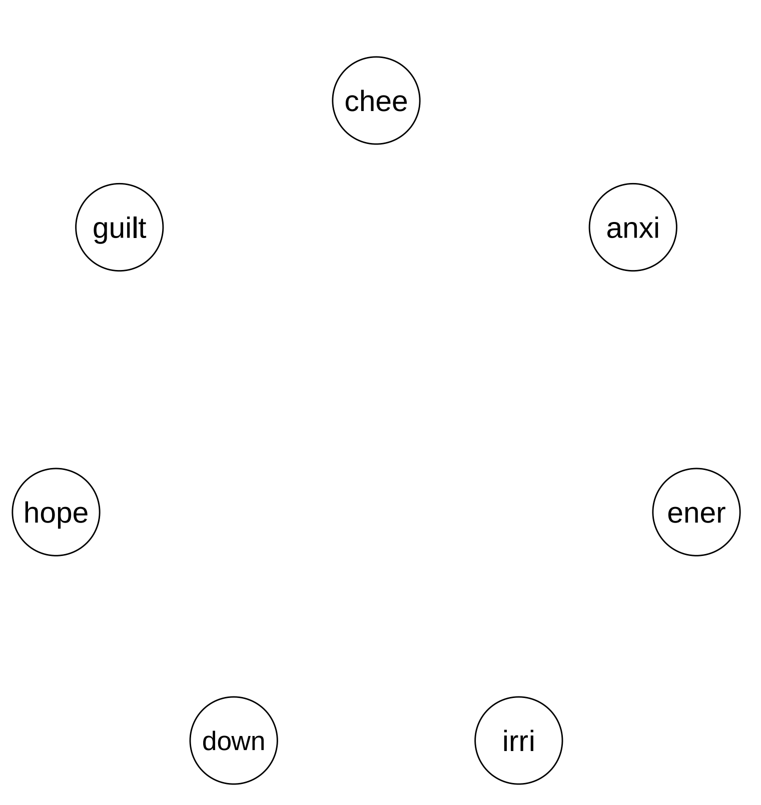

PCT plus ADM reg Pt 234 Estpoint 5

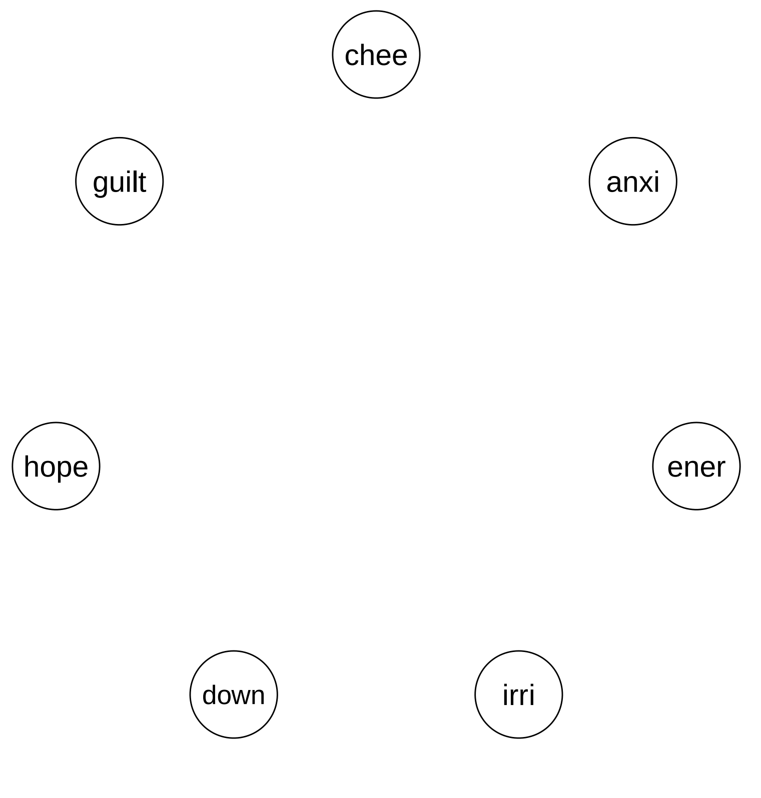

PCT plus ADM reg Pt 234 Estpoint 6

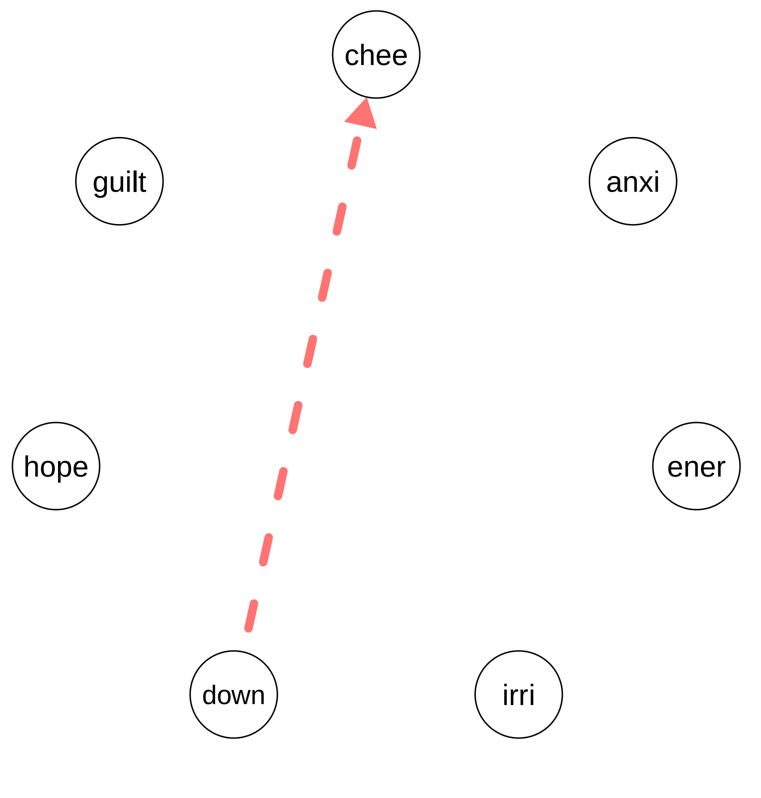

PCT plus ADM reg Pt 234 Estpoint 7

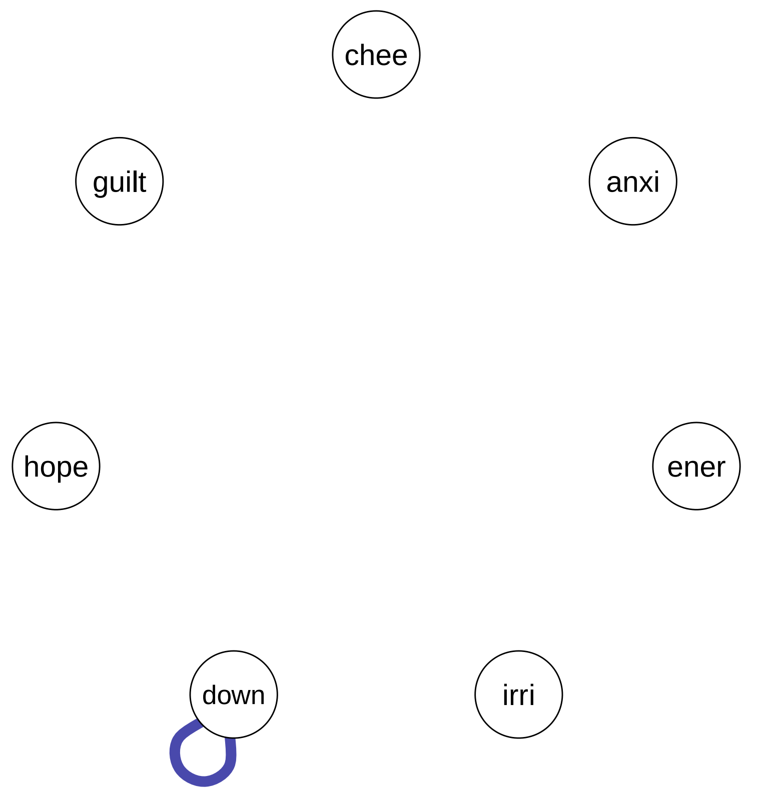

PCT plus ADM reg Pt 234 Estpoint 8

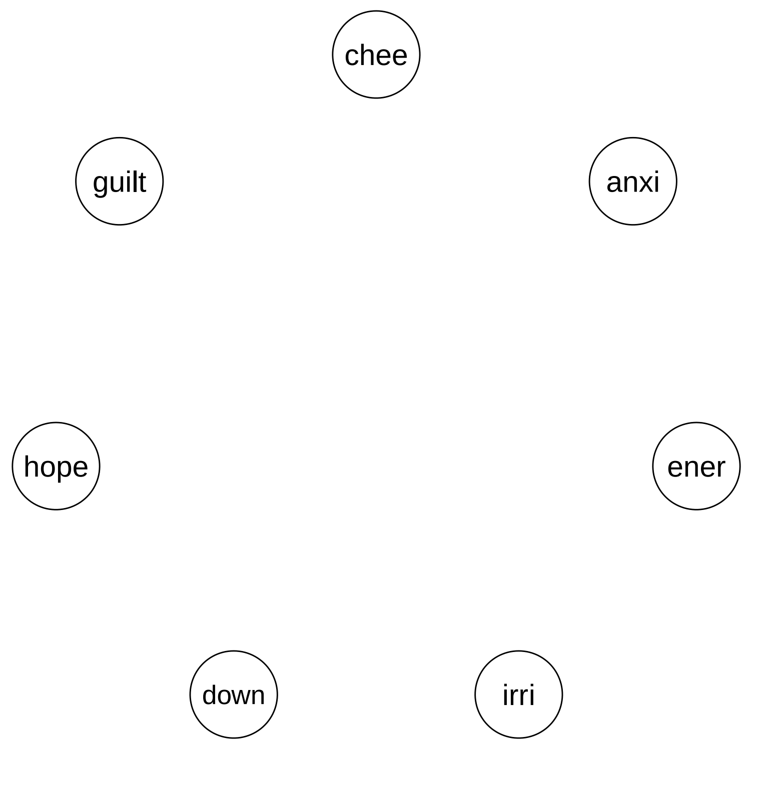

PCT plus ADM reg Pt 250 Estpoint 1

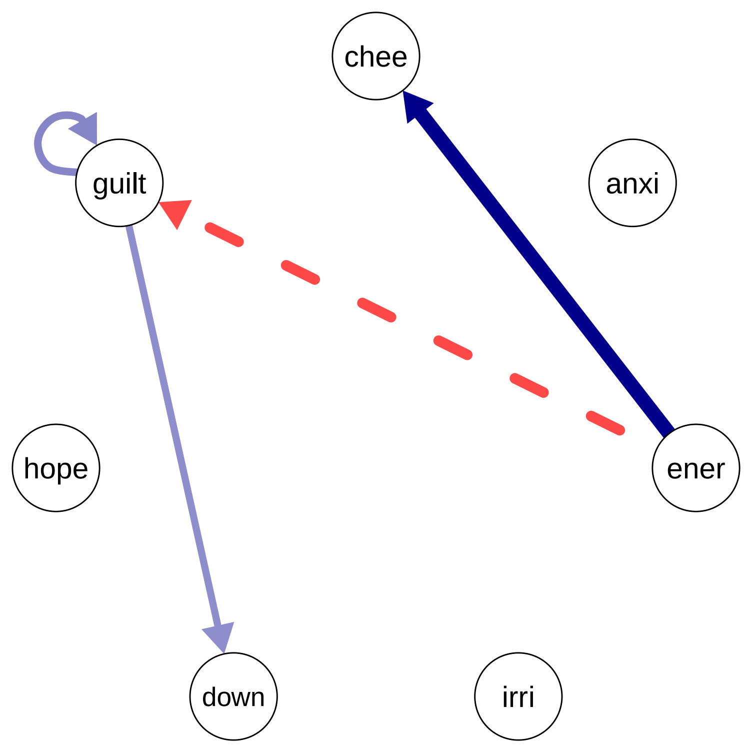

PCT plus ADM reg Pt 250 Estpoint 2

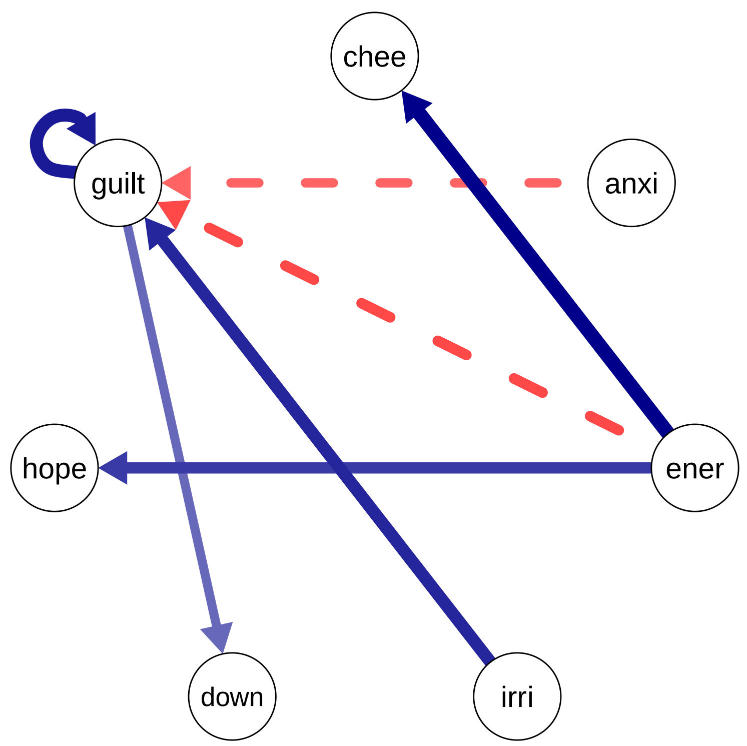

PCT plus ADM reg Pt 250 Estpoint 3

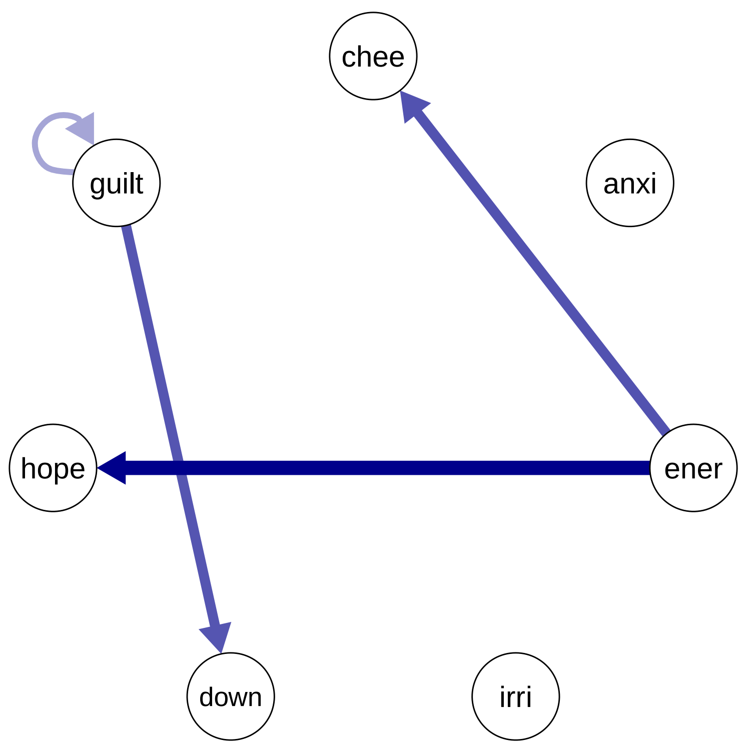

PCT plus ADM reg Pt 250 Estpoint 4

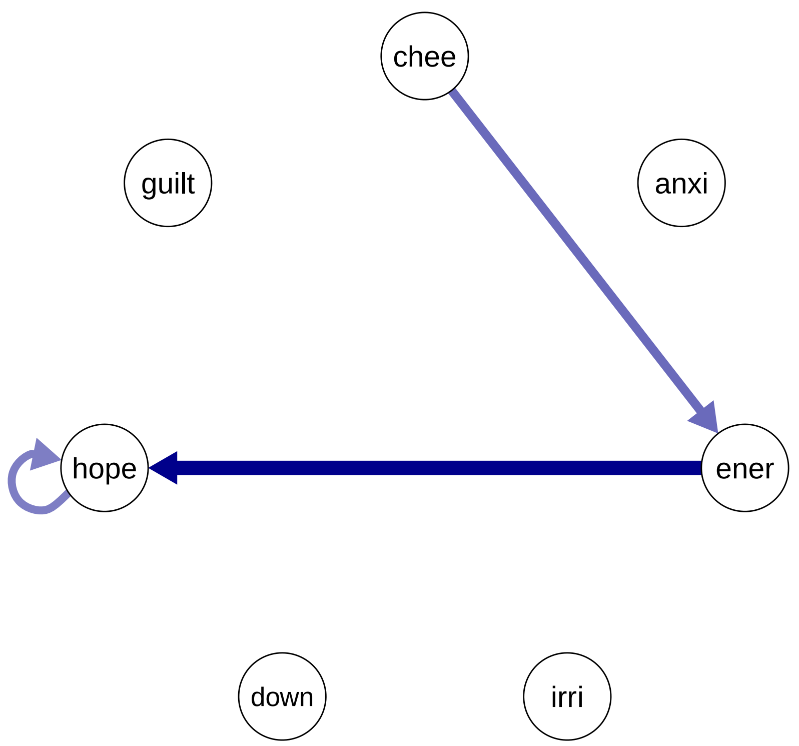

PCT plus ADM reg Pt 250 Estpoint 5

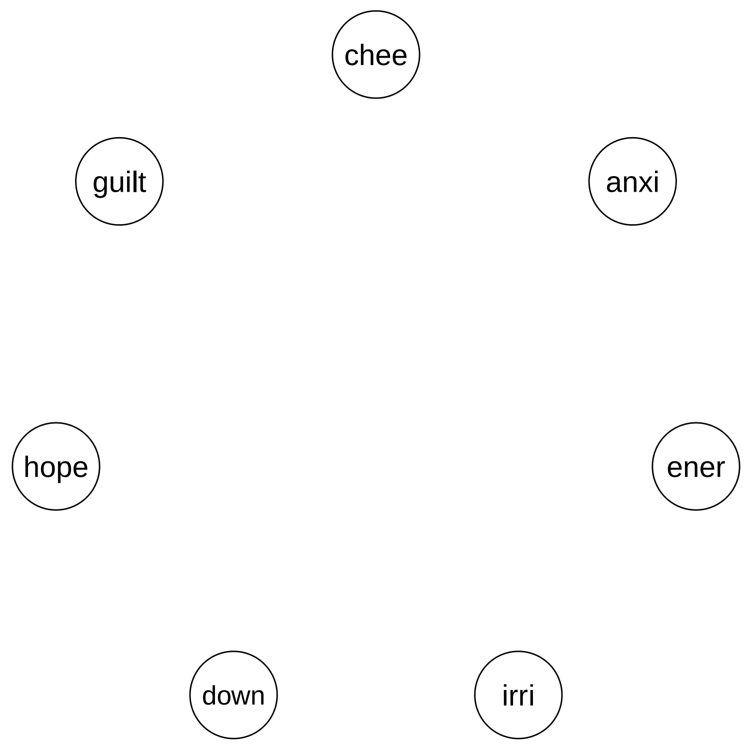

PCT plus ADM reg Pt 250 Estpoint 6

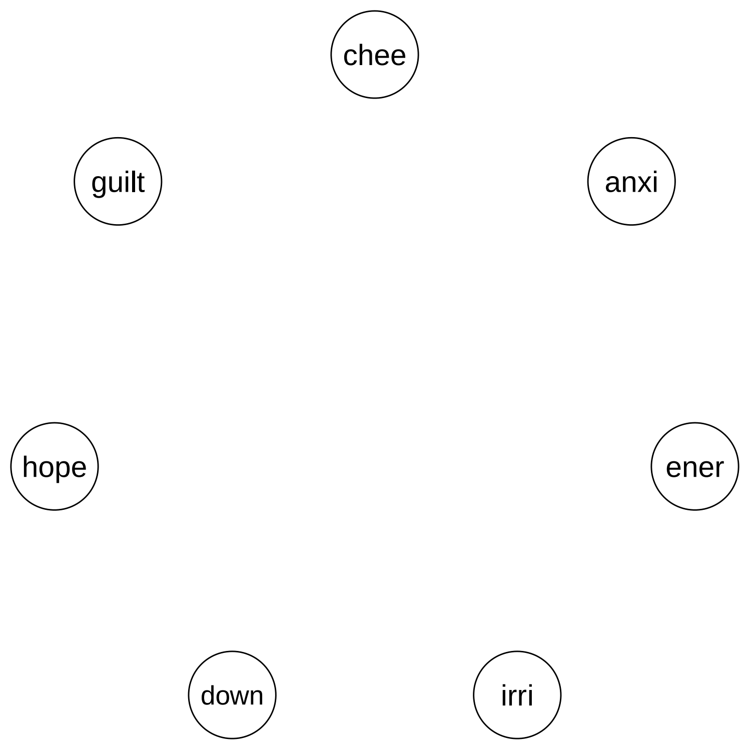

PCT plus ADM reg Pt 250 Estpoint 7

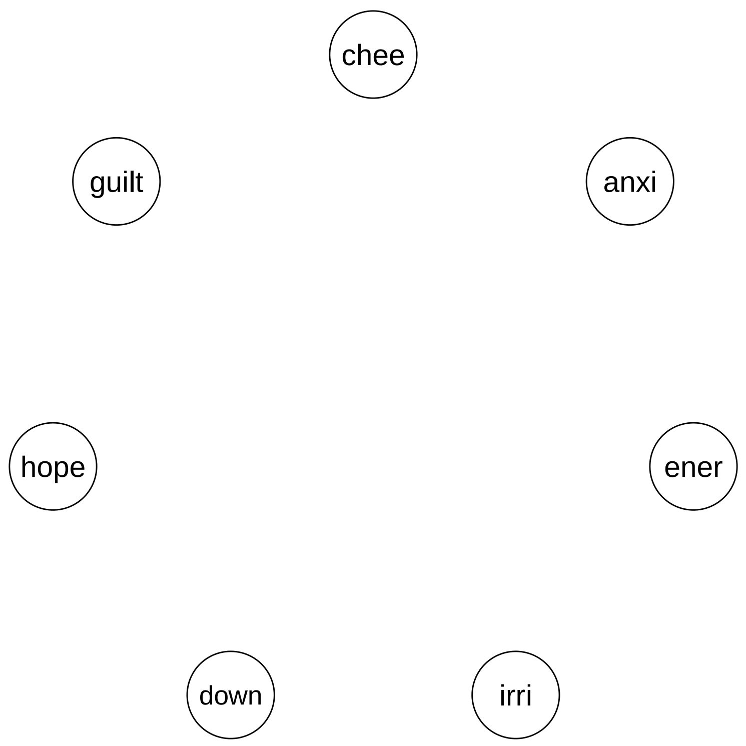

PCT plus ADM reg Pt 250 Estpoint 8

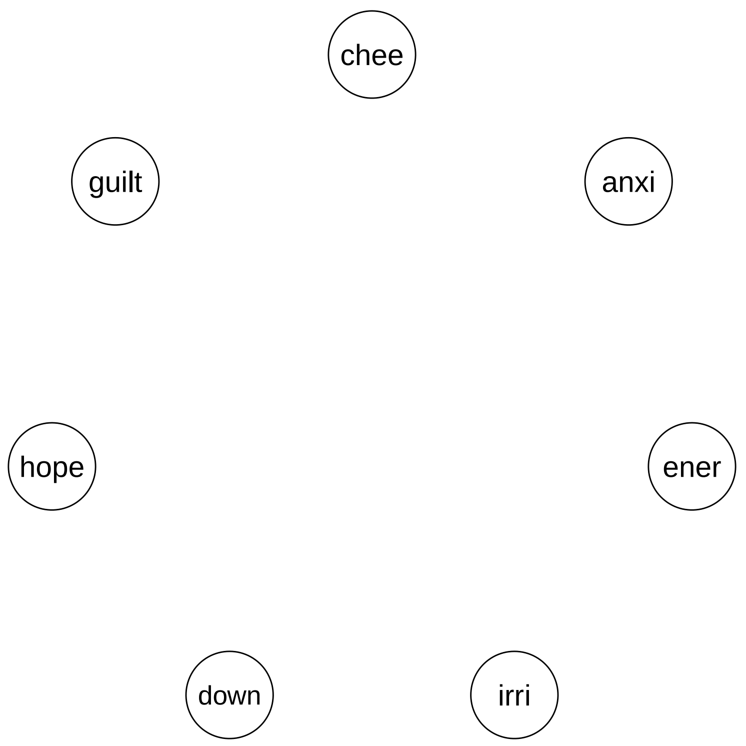

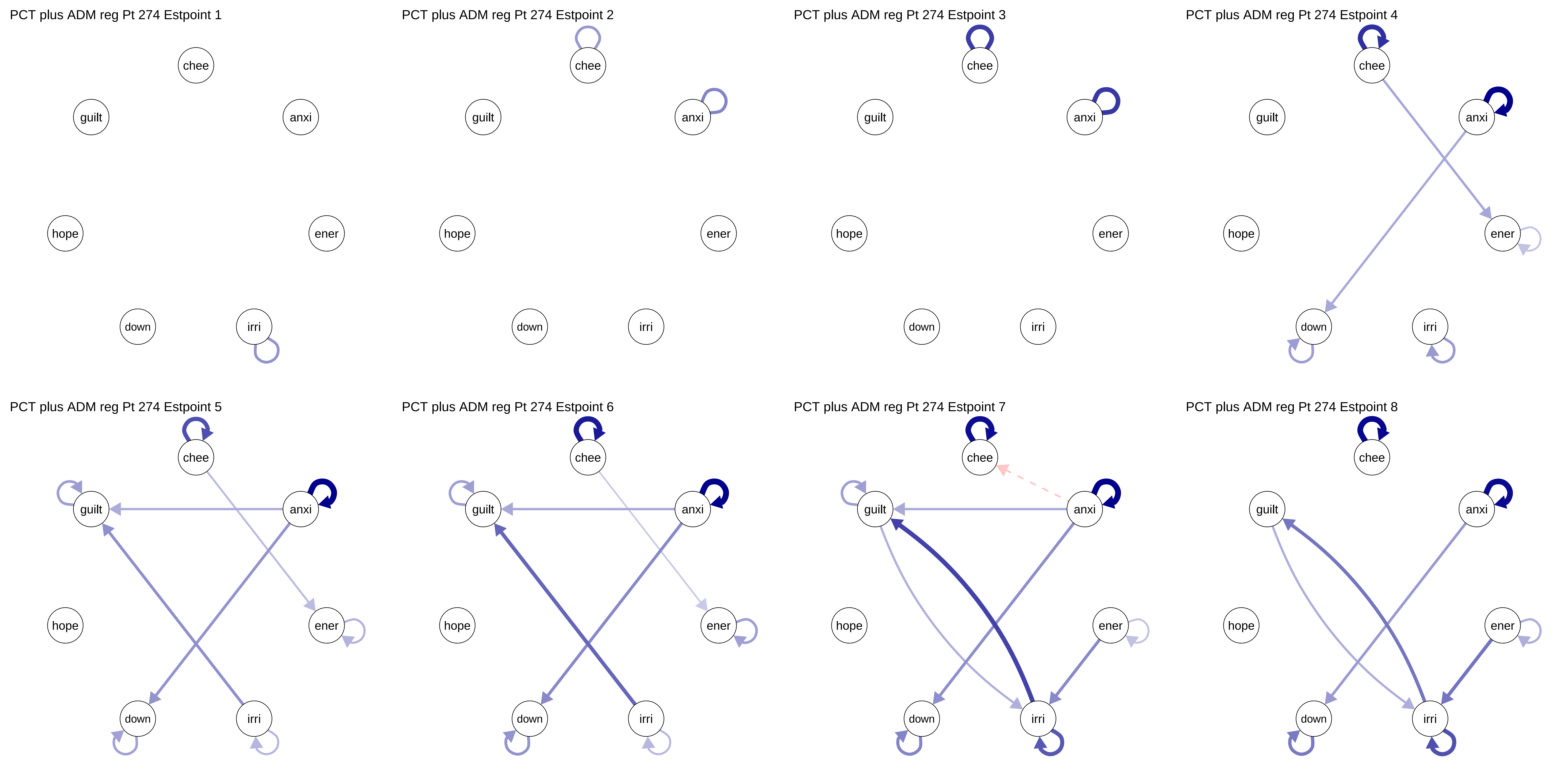
PCT plus ADM reg Pt 274 Estpoint 1
chee
guilt
anxi
hope
ener
down
irri
PCT plus ADM reg Pt 274 Estpoint 2
chee
guilt
anxi
hope
ener
down
irri
PCT plus ADM reg Pt 274 Estpoint 3
chee
guilt
anxi
hope
ener
down
irri
PCT plus ADM reg Pt 274 Estpoint 4
chee
guilt
anxi
hope
ener
down
irri
PCT plus ADM reg Pt 274 Estpoint 5
chee
guilt
anxi
hope
ener
down
irri
PCT plus ADM reg Pt 274 Estpoint 6
chee
guilt
anxi
hope
ener
down
irri
PCT plus ADM reg Pt 274 Estpoint 7
chee
guilt
anxi
hope
ener
down
irri
PCT plus ADM reg Pt 274 Estpoint 8
chee
guilt
anxi
hope
ener
down
irri

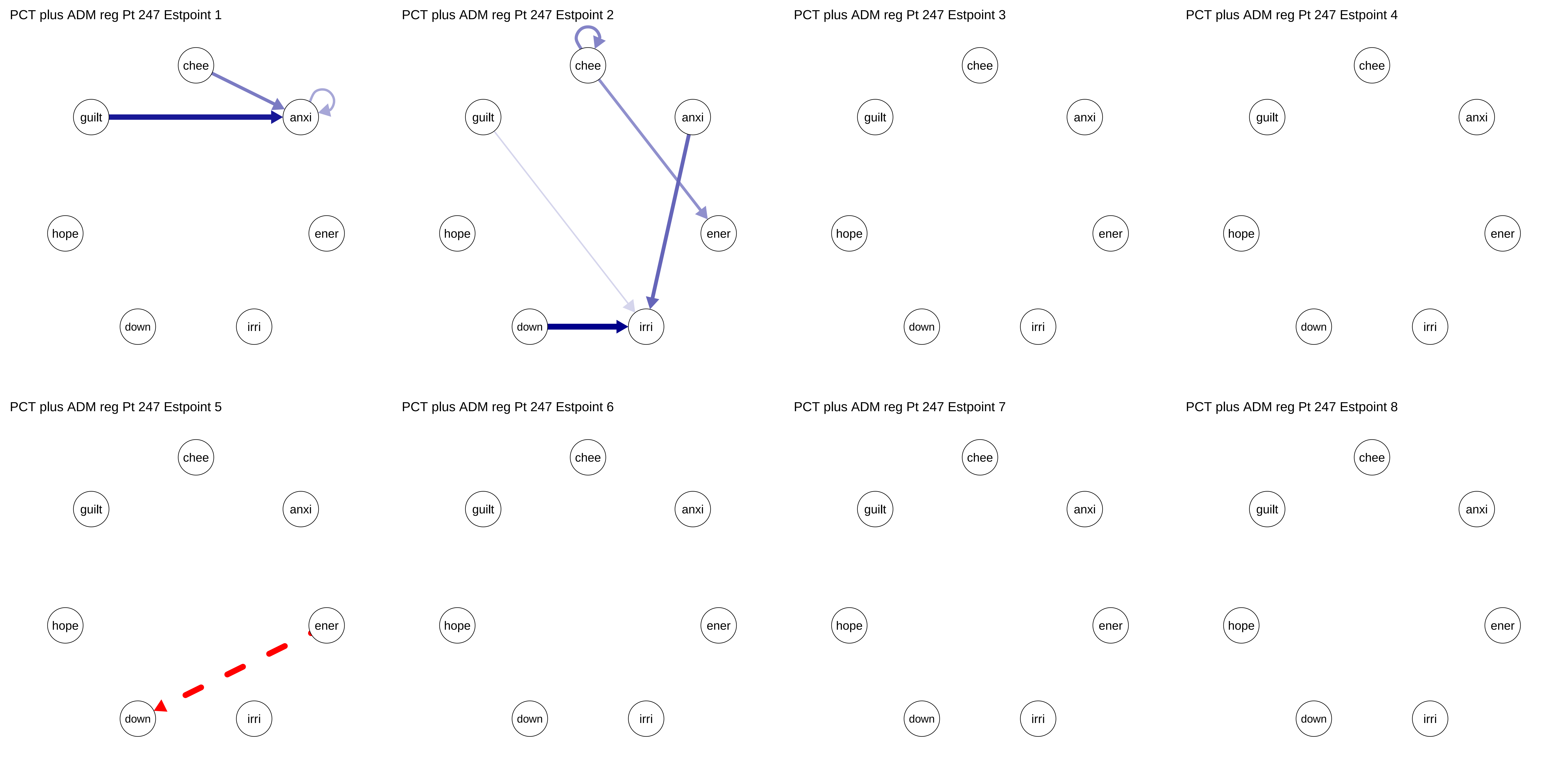
PCT plus ADM reg Pt 247 Estpoint 1
chee
guilt
anxi
hope
ener
down
irri
PCT plus ADM reg Pt 247 Estpoint 2
chee
guilt
anxi
hope
ener
down
irri
PCT plus ADM reg Pt 247 Estpoint 3
chee
guilt
anxi
hope
ener
down
irri
PCT plus ADM reg Pt 247 Estpoint 4
chee
guilt
anxi
hope
ener
down
irri
PCT plus ADM reg Pt 247 Estpoint 5
chee
guilt
anxi
hope
ener
down
irri
PCT plus ADM reg Pt 247 Estpoint 6
chee
guilt
anxi
hope
ener
down
irri
PCT plus ADM reg Pt 247 Estpoint 7
chee
guilt
anxi
hope
ener
down
irri
PCT plus ADM reg Pt 247 Estpoint 8
chee
guilt
anxi
hope
ener
down
irri

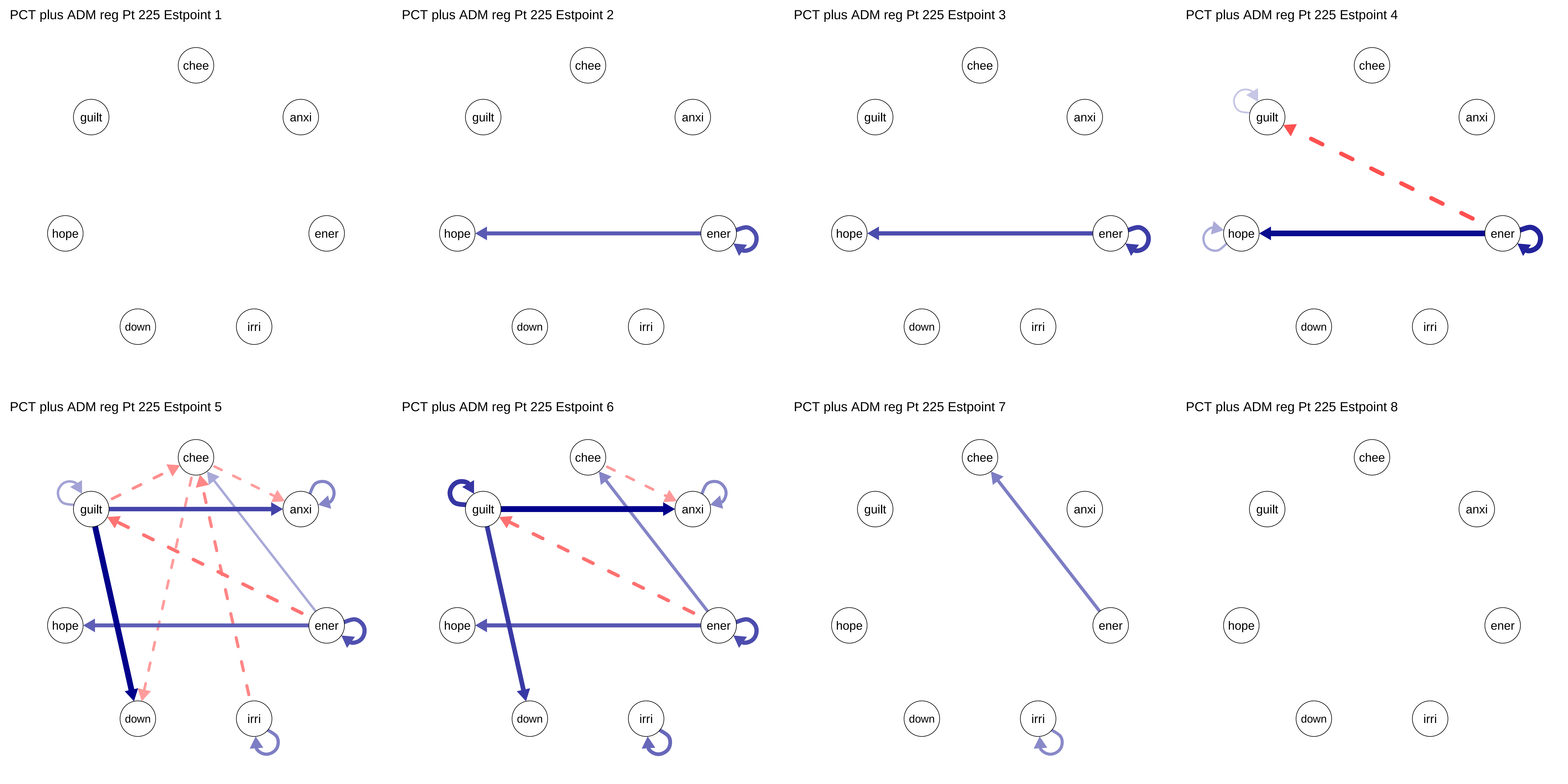
PCT plus ADM reg Pt 225 Estpoint 1
PCT plus ADM reg Pt 225 Estpoint 2
PCT plus ADM reg Pt 225 Estpoint 3
PCT plus ADM reg Pt 225 Estpoint 4
PCT plus ADM reg Pt 225 Estpoint 5
PCT plus ADM reg Pt 225 Estpoint 6
PCT plus ADM reg Pt 225 Estpoint 7
PCT plus ADM reg Pt 225 Estpoint 8
chee
guilt
anxi
hope
ener
down
irri

PCT plus ADM reg Pt 248 Estpoint 1

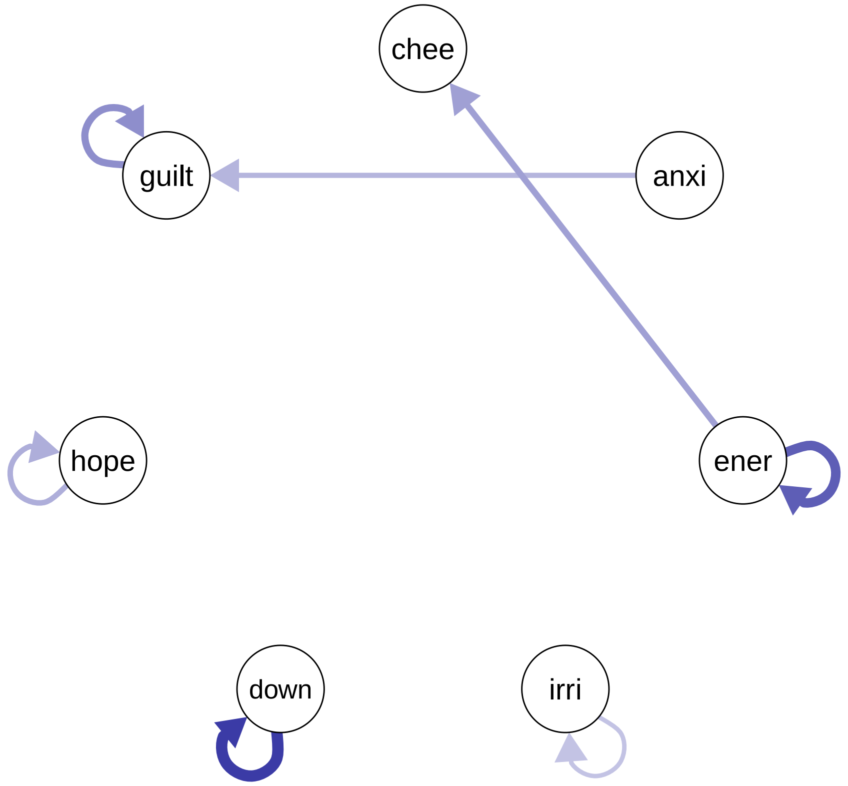

PCT plus ADM reg Pt 248 Estpoint 2

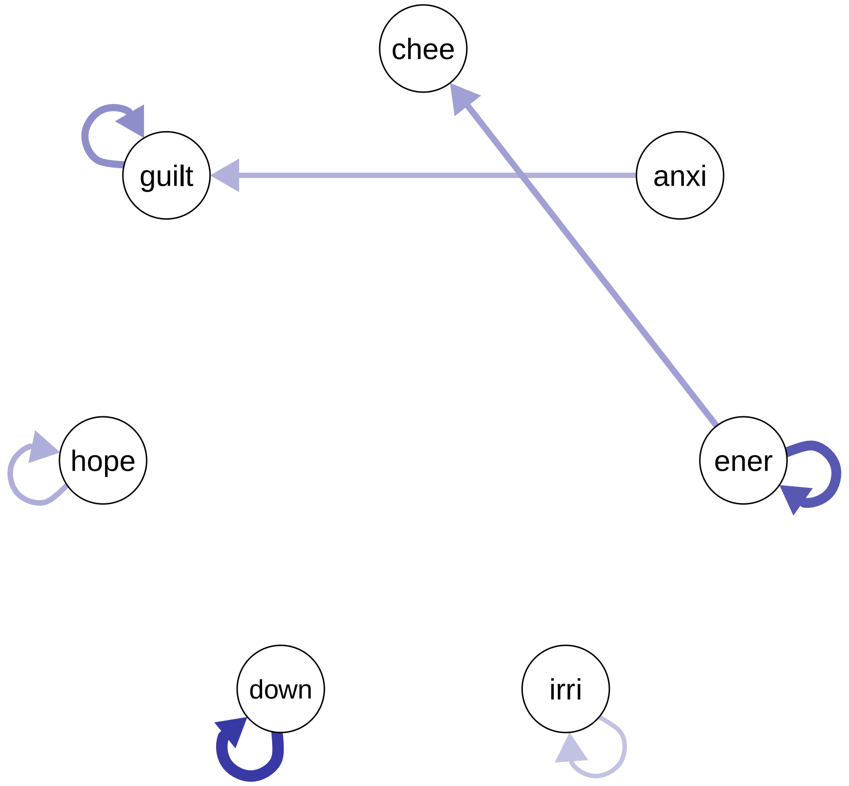

PCT plus ADM reg Pt 248 Estpoint 3

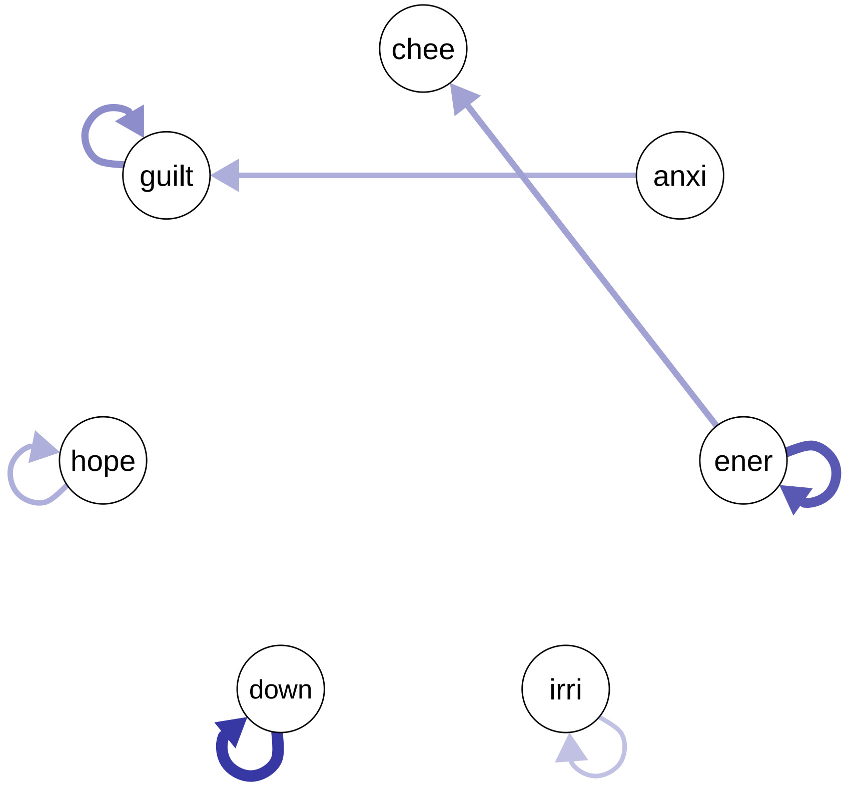

PCT plus ADM reg Pt 248 Estpoint 4

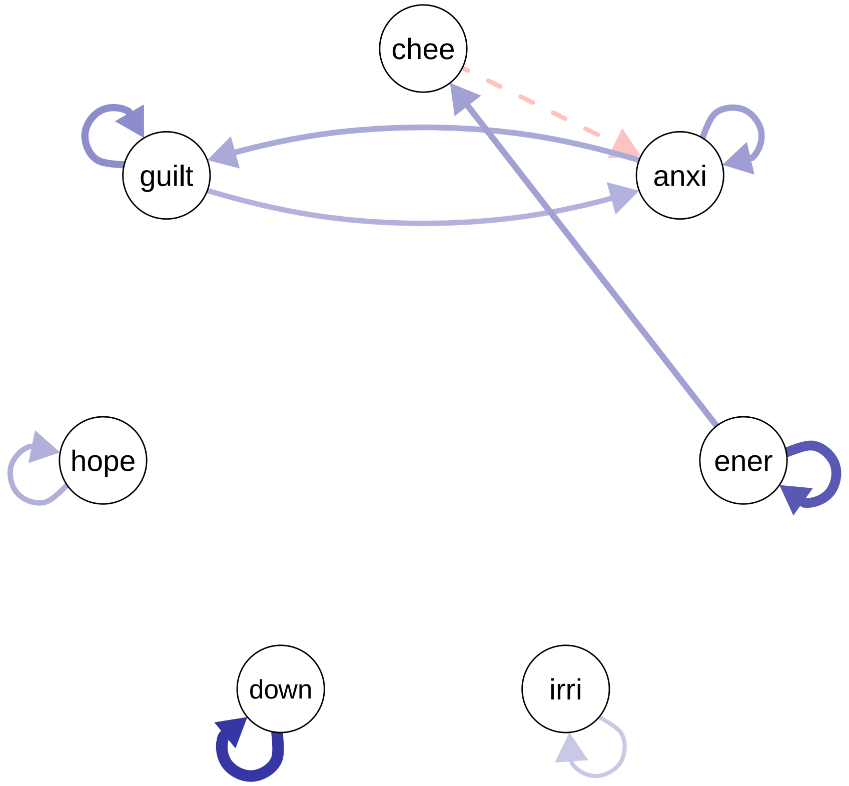

PCT plus ADM reg Pt 248 Estpoint 5

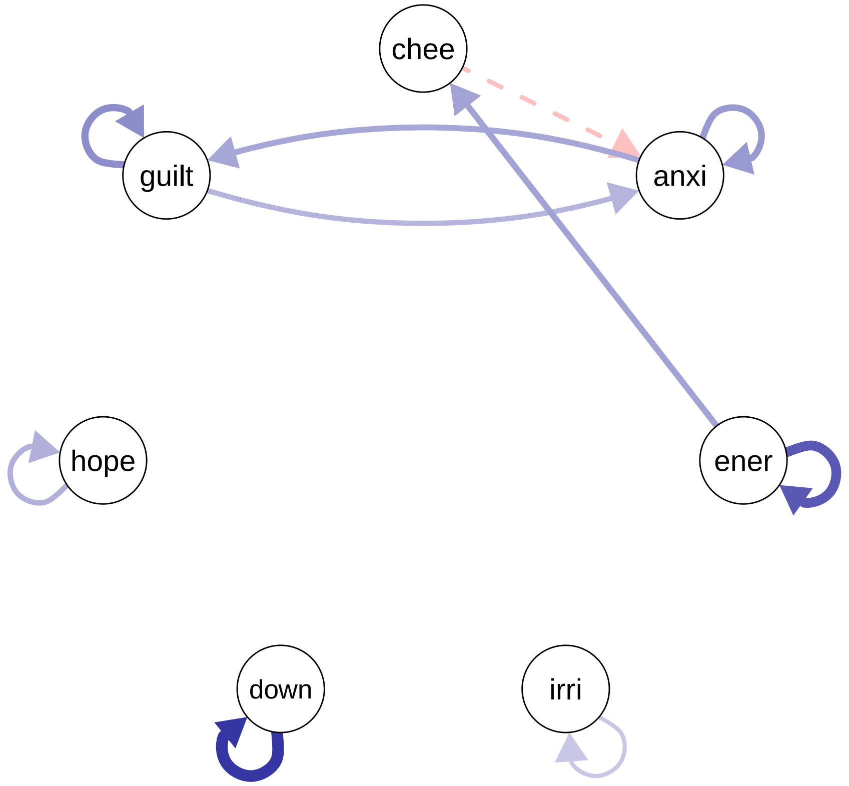

PCT plus ADM reg Pt 248 Estpoint 6

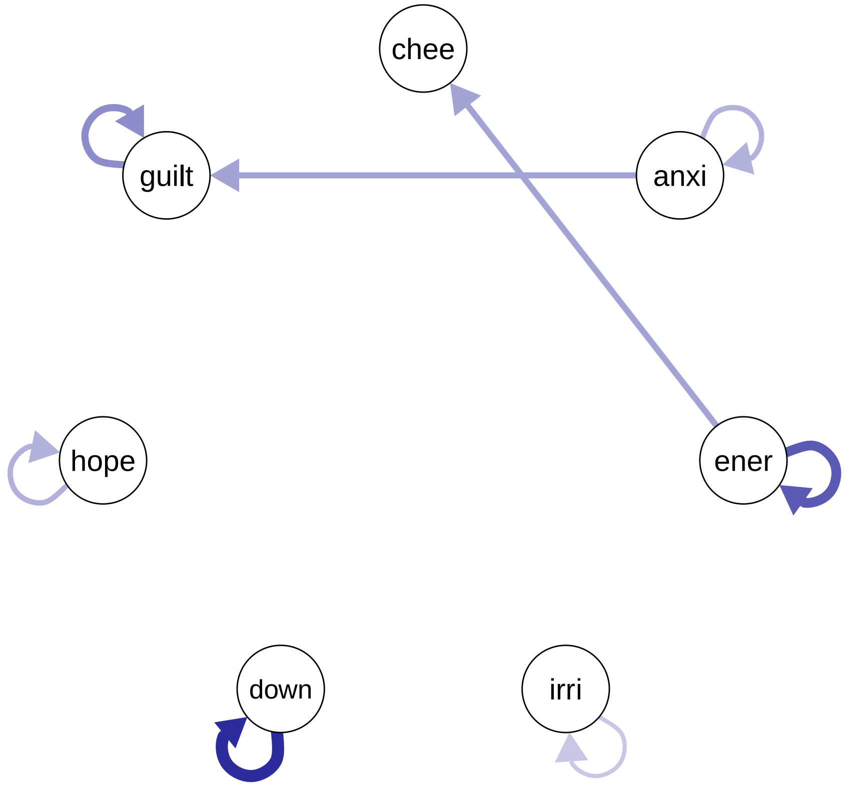

PCT plus ADM reg Pt 248 Estpoint 7

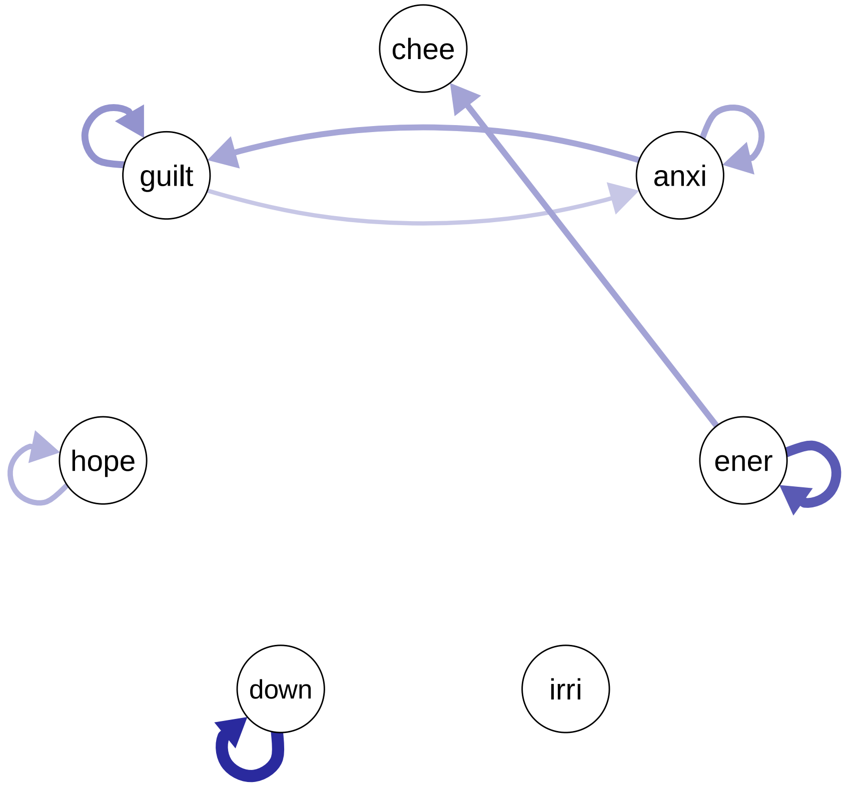

PCT plus ADM reg Pt 248 Estpoint 8

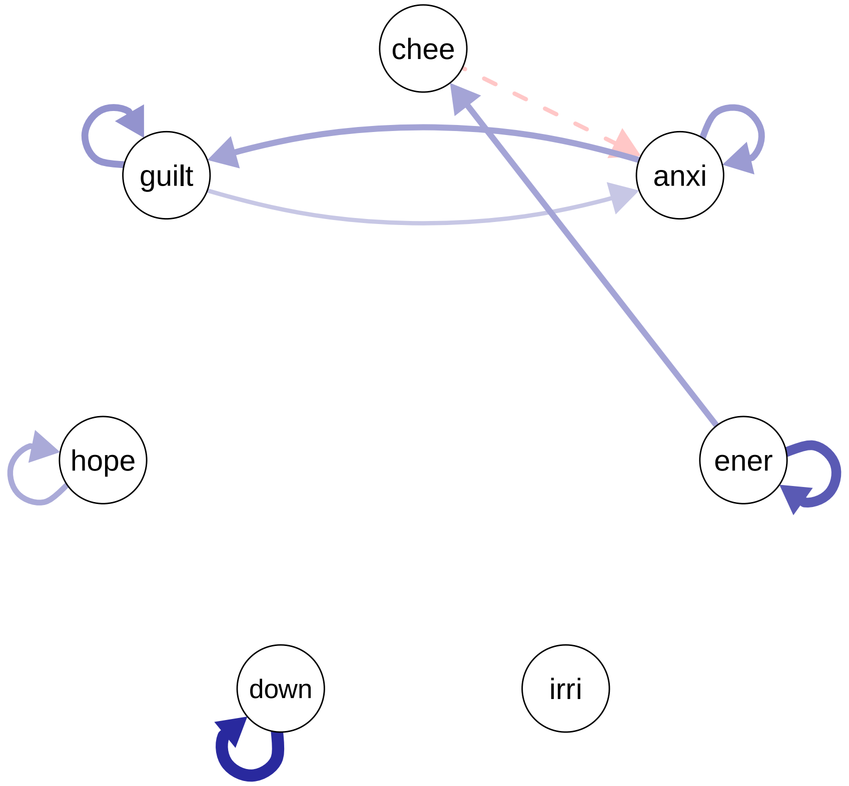

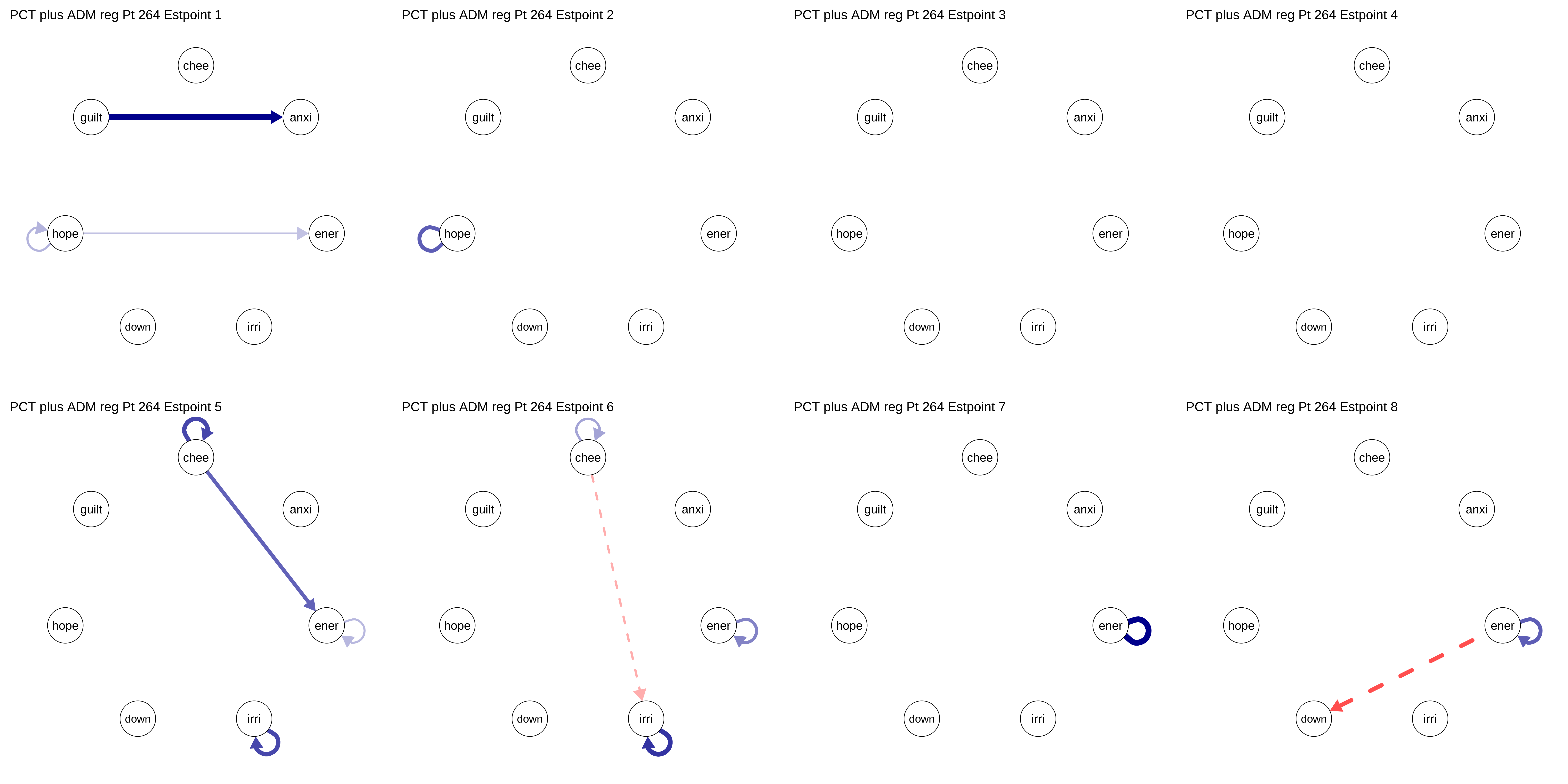
PCT plus ADM reg Pt 264 Estpoint 1
chee
guilt
anxi
hope
ener
down
irri
PCT plus ADM reg Pt 264 Estpoint 2
chee
guilt
anxi
hope
ener
down
irri
PCT plus ADM reg Pt 264 Estpoint 3
chee
guilt
anxi
hope
ener
down
irri
PCT plus ADM reg Pt 264 Estpoint 4
chee
guilt
anxi
hope
ener
down
irri
PCT plus ADM reg Pt 264 Estpoint 5
chee
guilt
anxi
hope
ener
down
irri
PCT plus ADM reg Pt 264 Estpoint 6
chee
guilt
anxi
hope
ener
down
irri
PCT plus ADM reg Pt 264 Estpoint 7
chee
guilt
anxi
hope
ener
down
irri
PCT plus ADM reg Pt 264 Estpoint 8
chee
guilt
anxi
hope
ener
down
irri

PCT plus ADM reg Pt 255 Estpoint 1

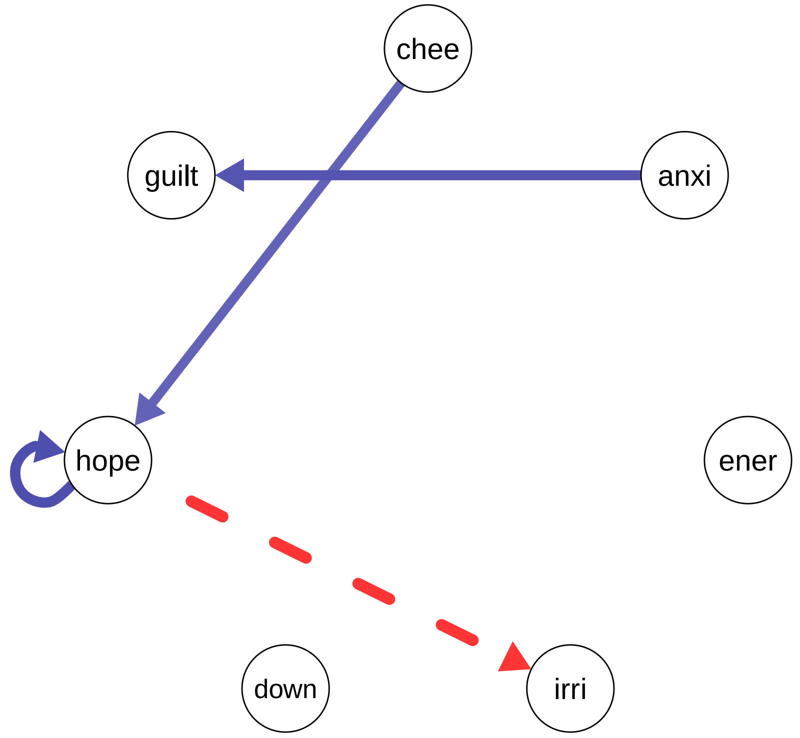

PCT plus ADM reg Pt 255 Estpoint 2

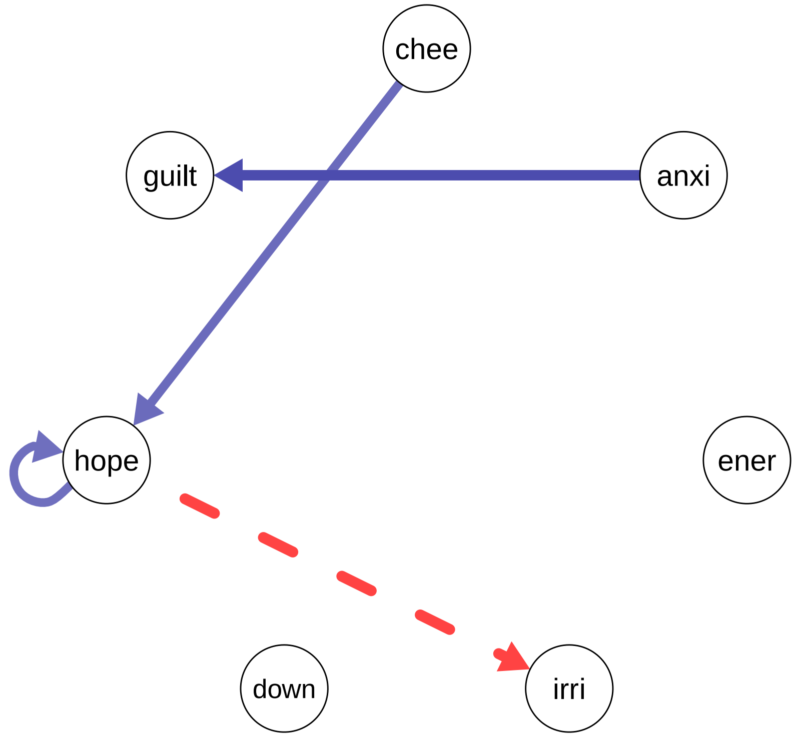

PCT plus ADM reg Pt 255 Estpoint 3

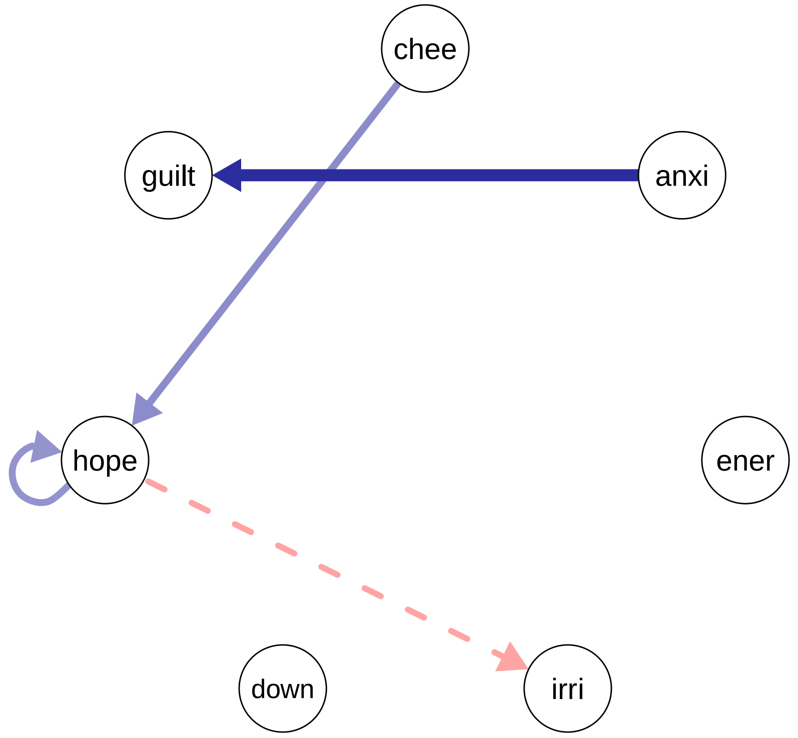

PCT plus ADM reg Pt 255 Estpoint 4

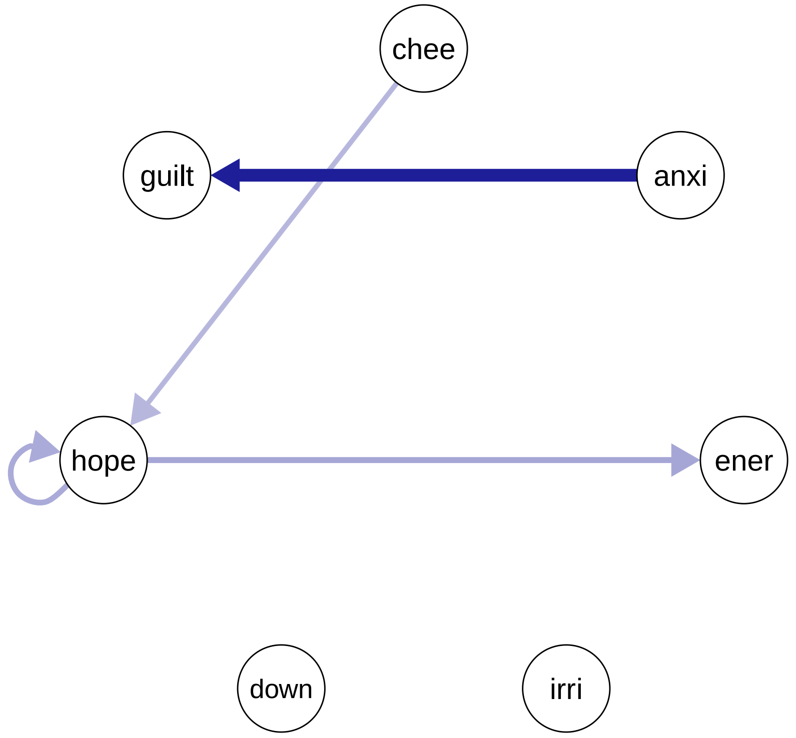

PCT plus ADM reg Pt 255 Estpoint 5

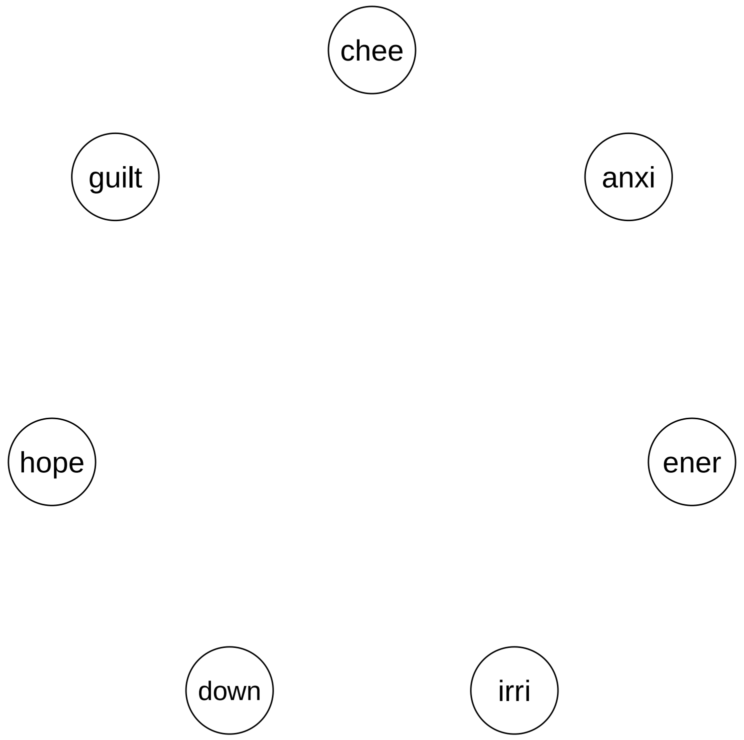

PCT plus ADM reg Pt 255 Estpoint 6

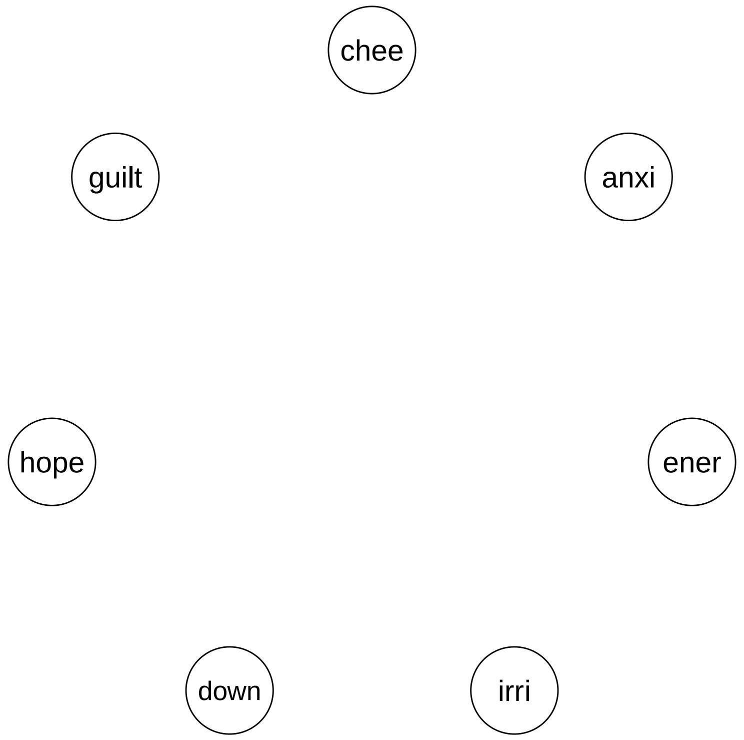

PCT plus ADM reg Pt 255 Estpoint 7

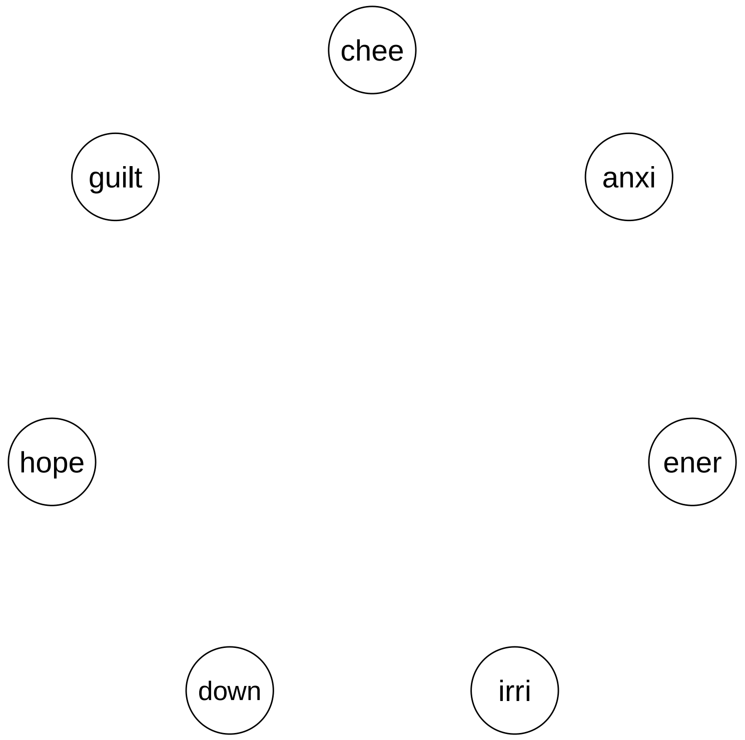

PCT plus ADM reg Pt 255 Estpoint 8

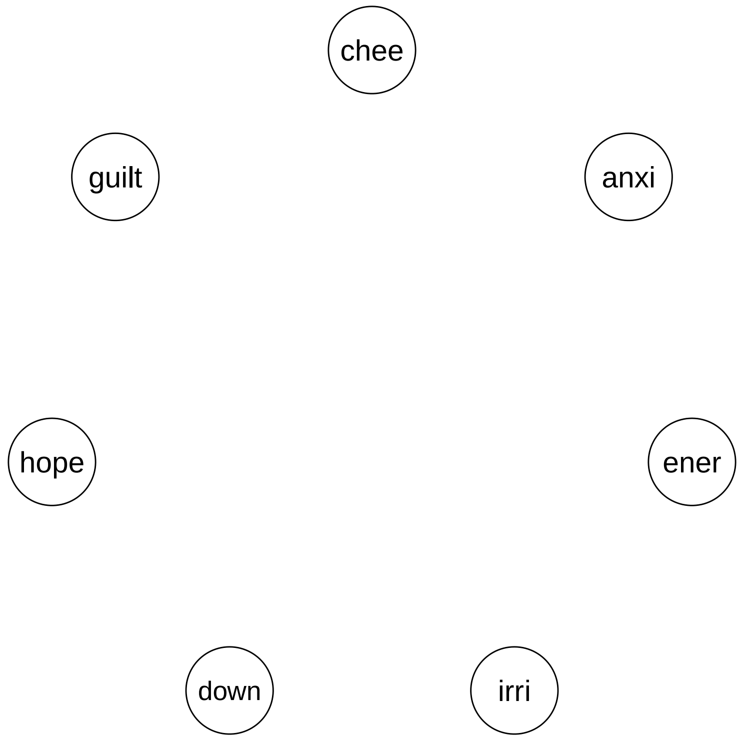

PCT plus ADM reg Pt 261 Estpoint 1

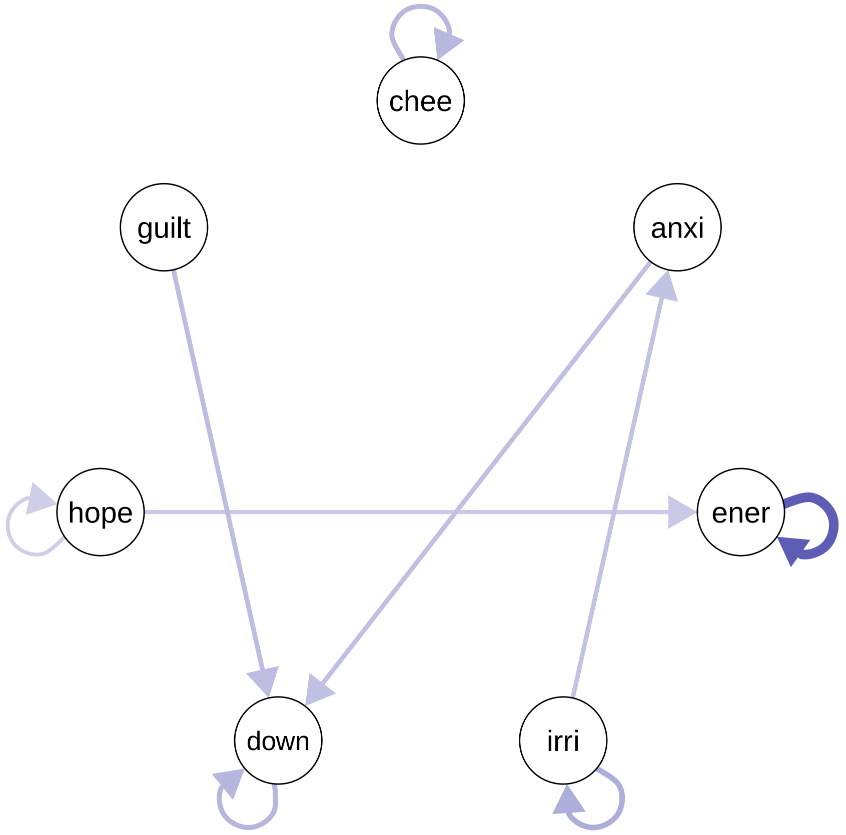

PCT plus ADM reg Pt 261 Estpoint 2

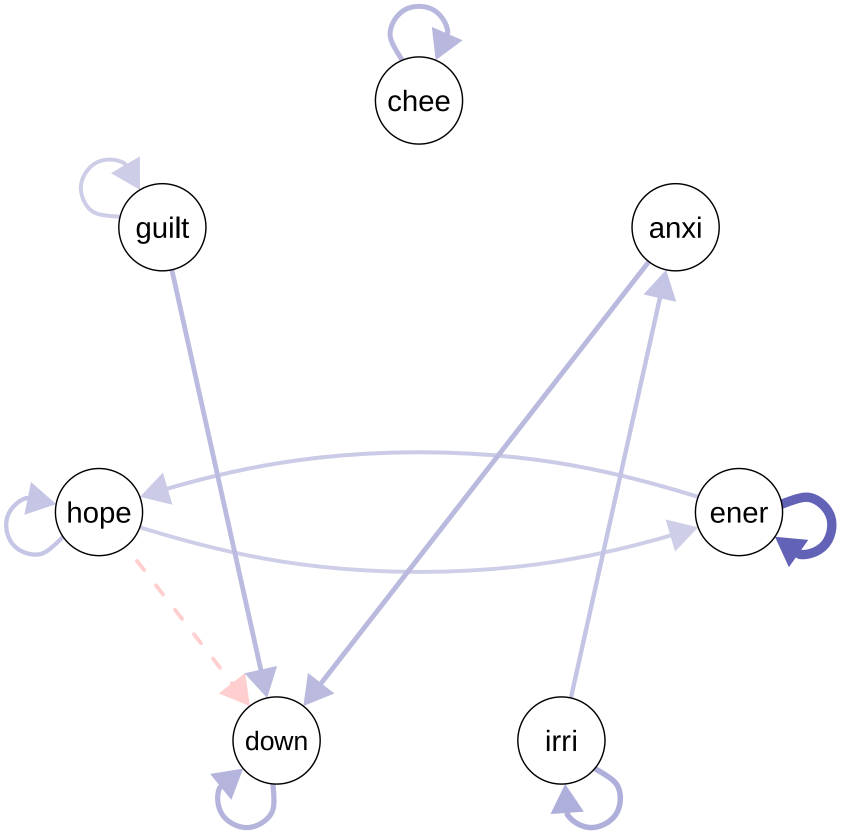

PCT plus ADM reg Pt 261 Estpoint 3

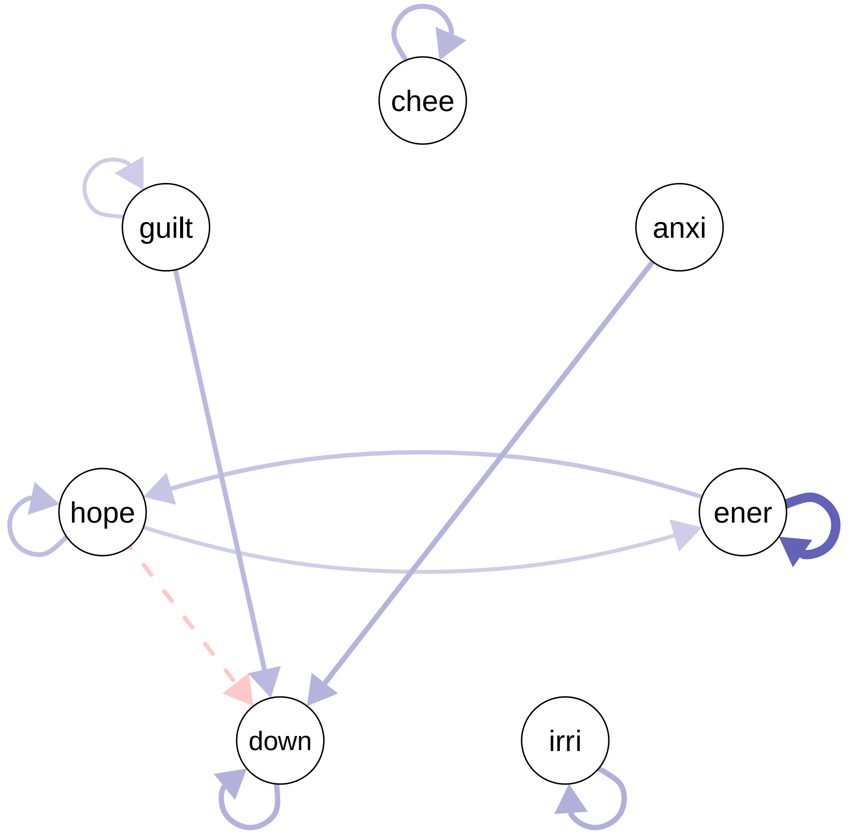

PCT plus ADM reg Pt 261 Estpoint 4

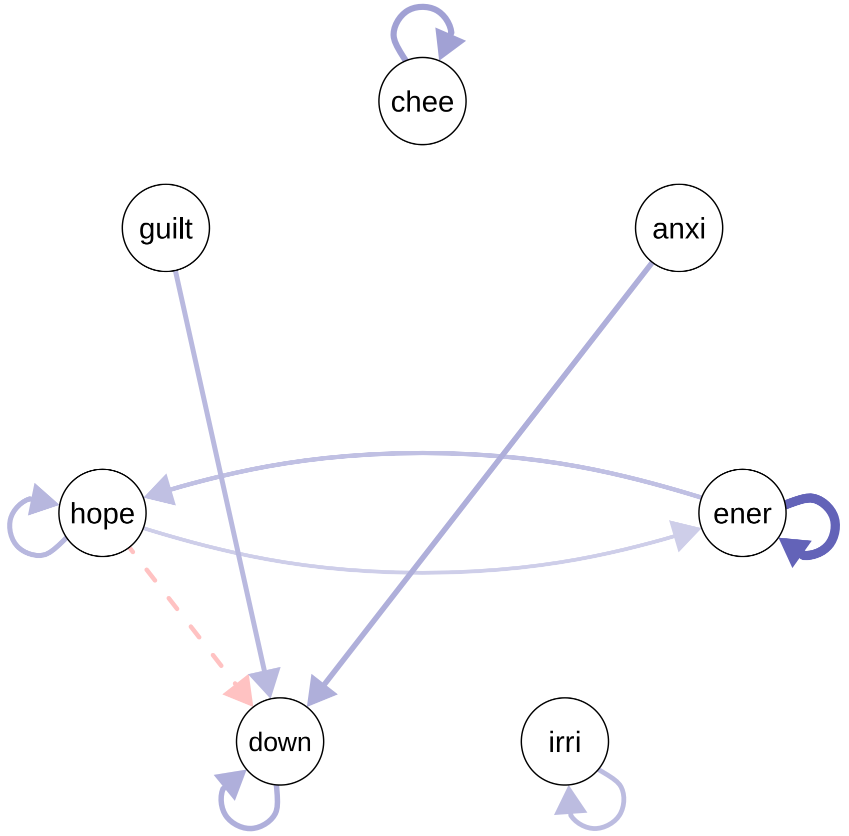

PCT plus ADM reg Pt 261 Estpoint 5

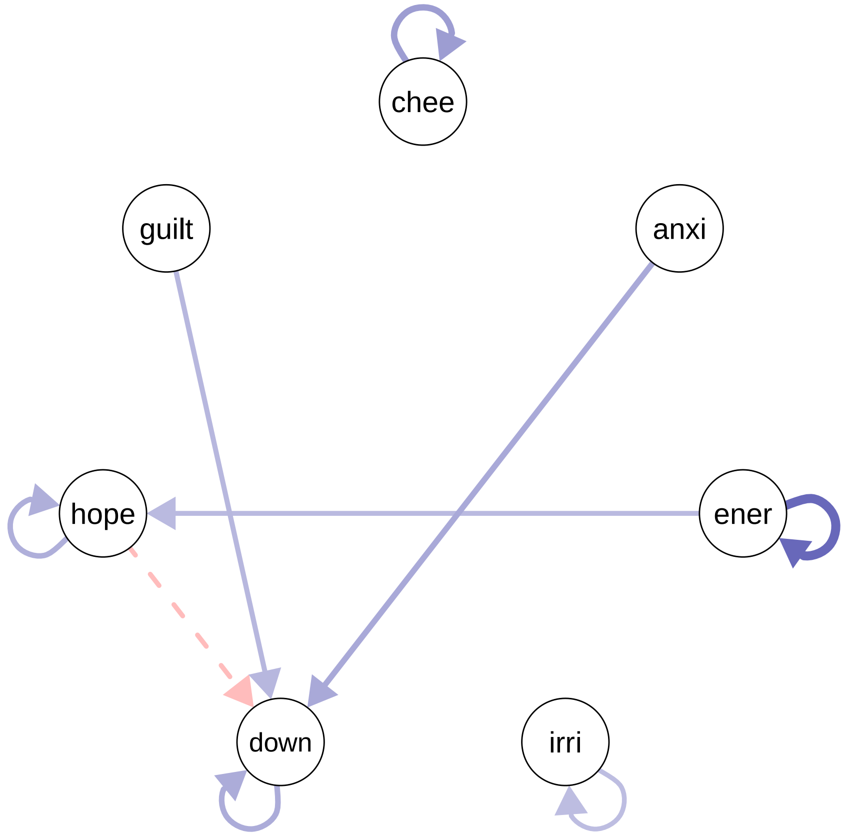

PCT plus ADM reg Pt 261 Estpoint 6

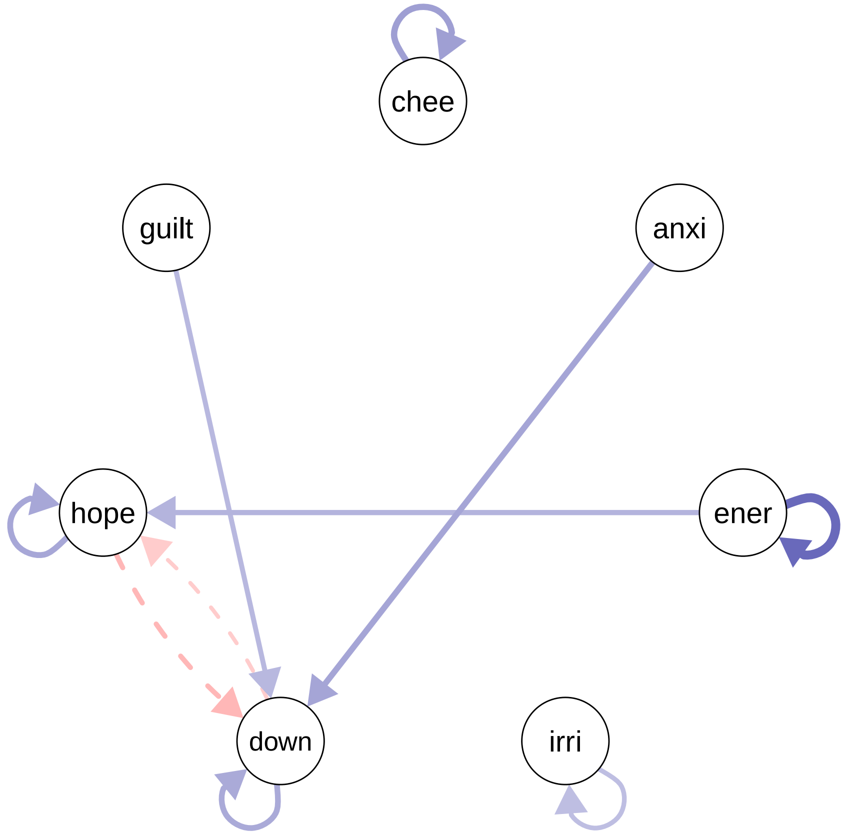

PCT plus ADM reg Pt 261 Estpoint 7

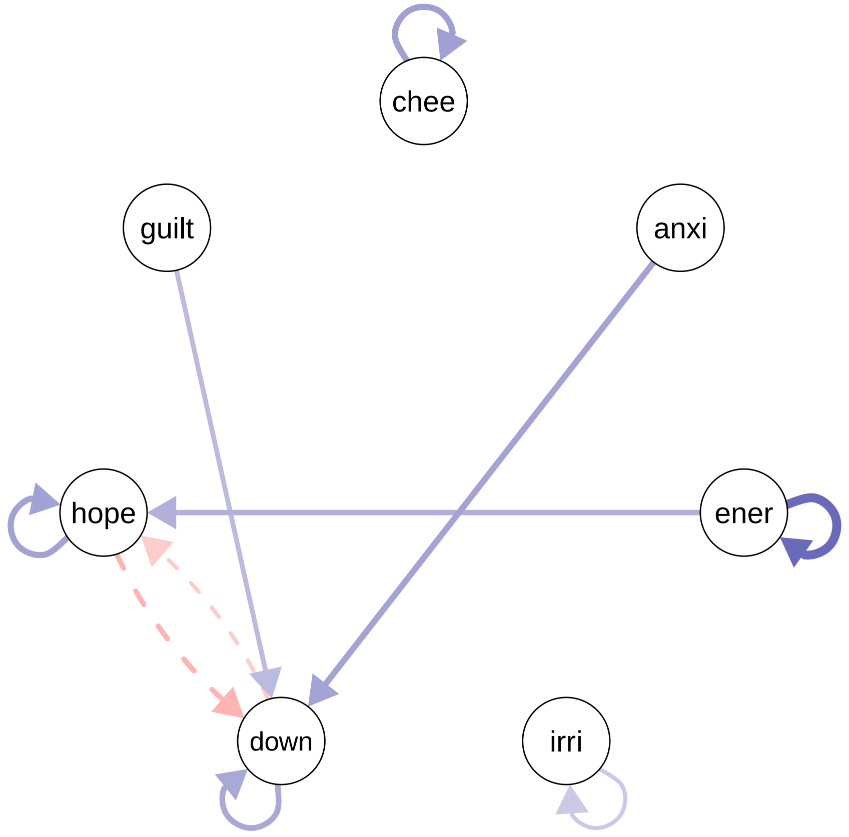

PCT plus ADM reg Pt 261 Estpoint 8

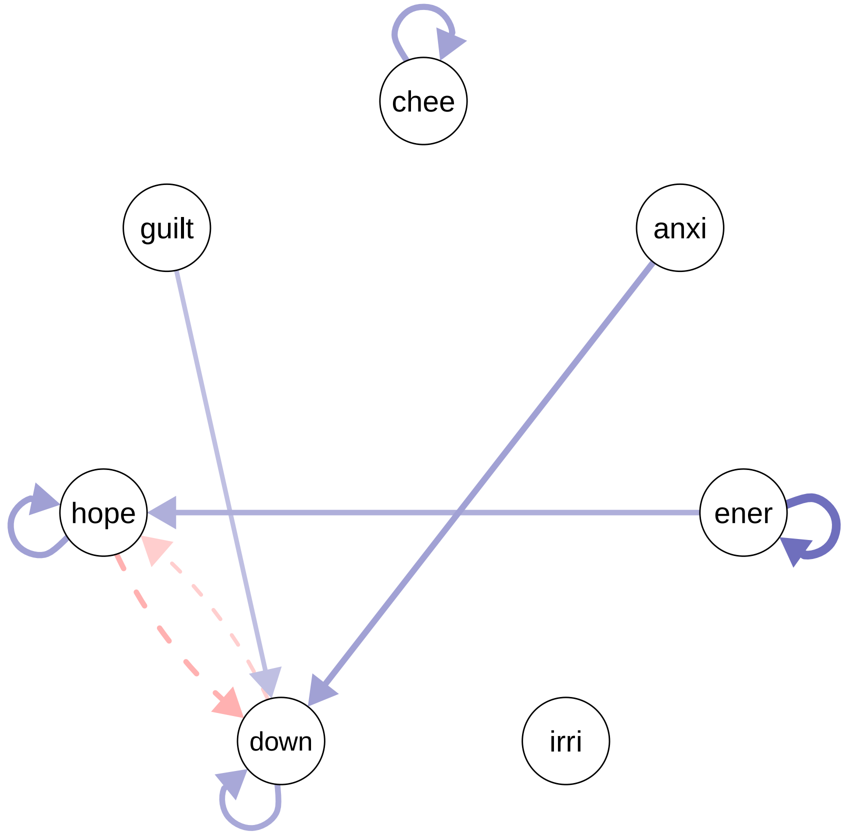

PCT plus ADM reg Pt 269 Estpoint 1

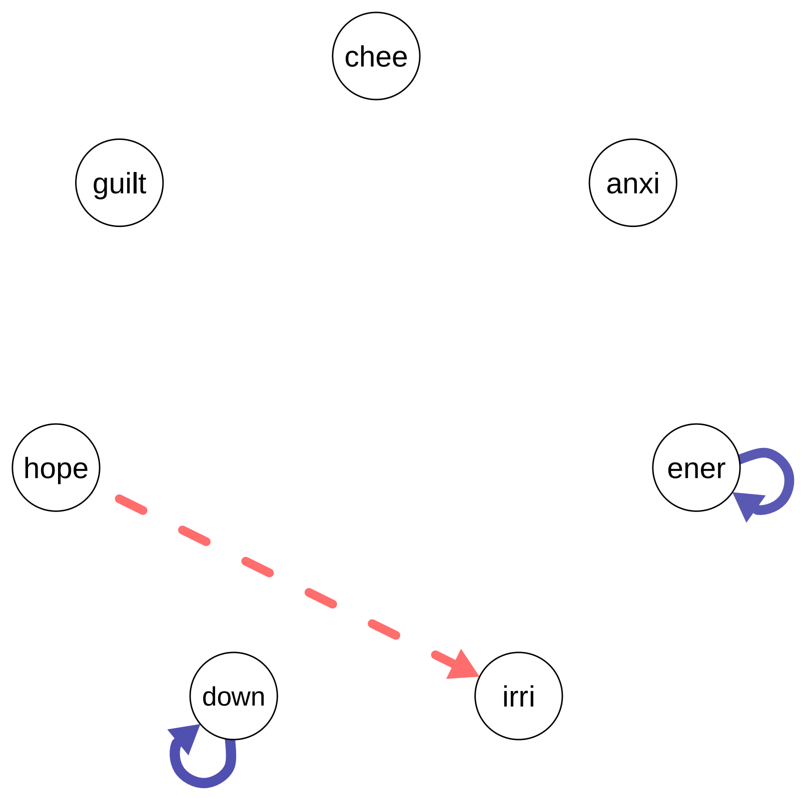

PCT plus ADM reg Pt 269 Estpoint 2

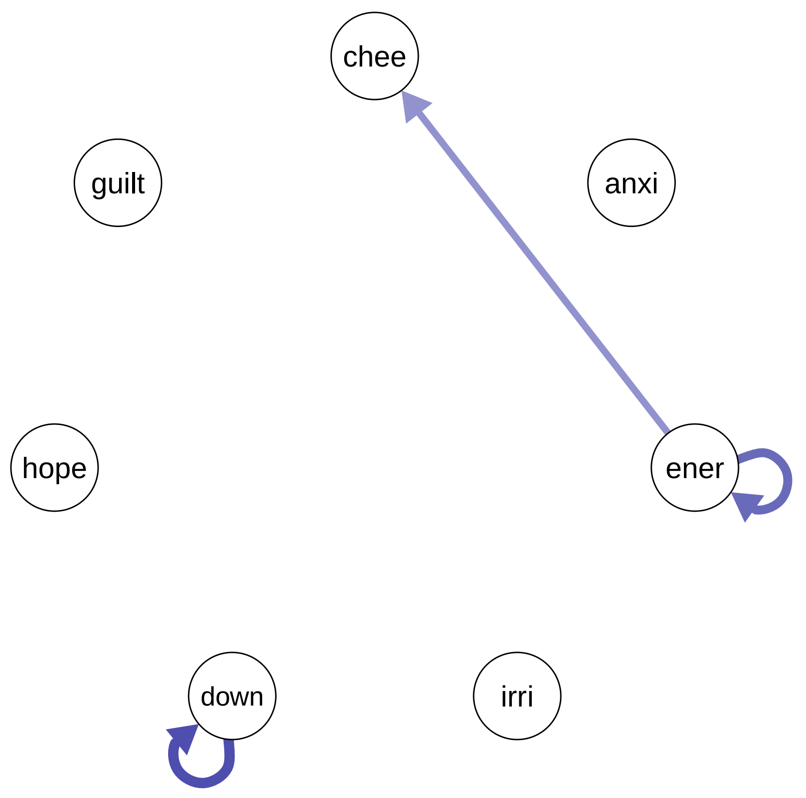

PCT plus ADM reg Pt 269 Estpoint 3

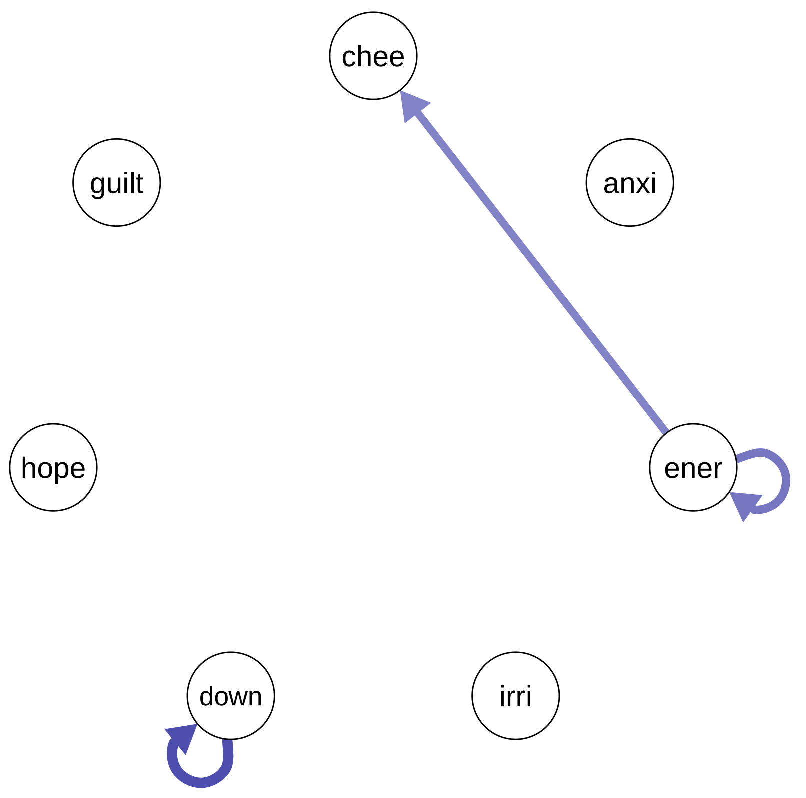

PCT plus ADM reg Pt 269 Estpoint 4

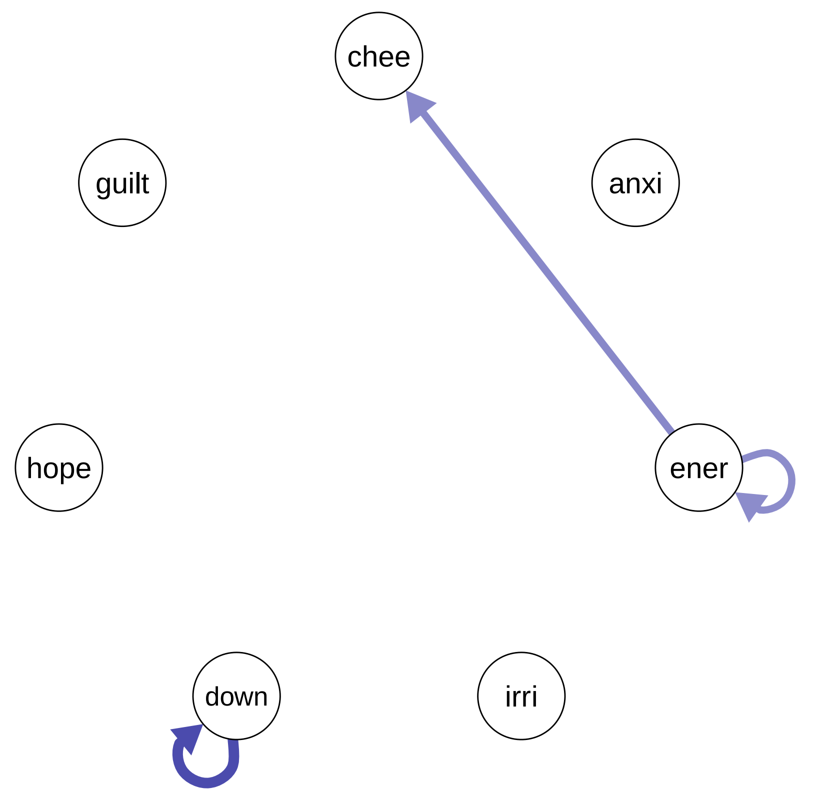

PCT plus ADM reg Pt 269 Estpoint 5

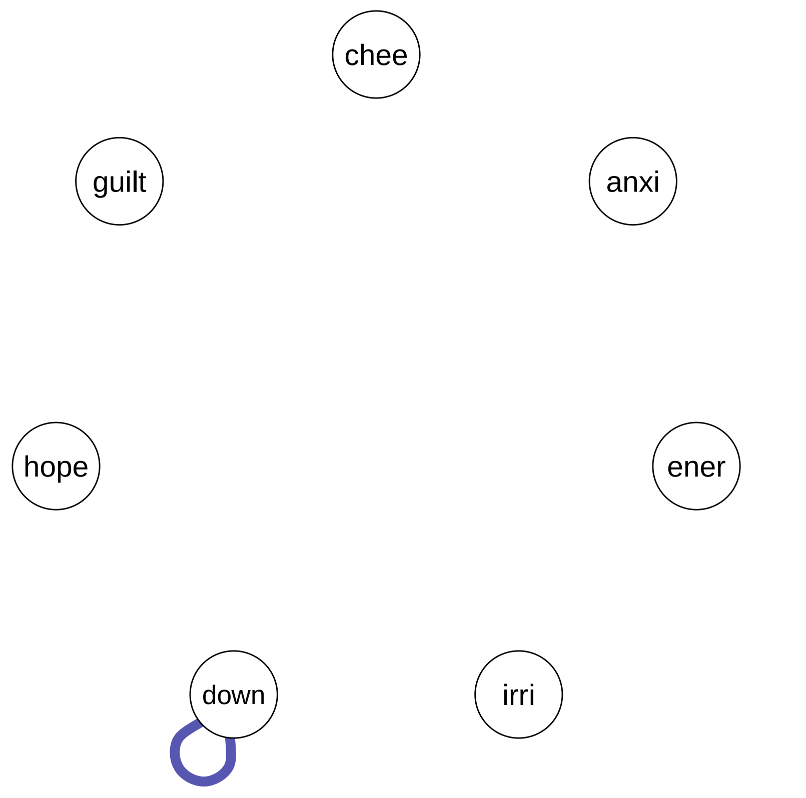

PCT plus ADM reg Pt 269 Estpoint 6

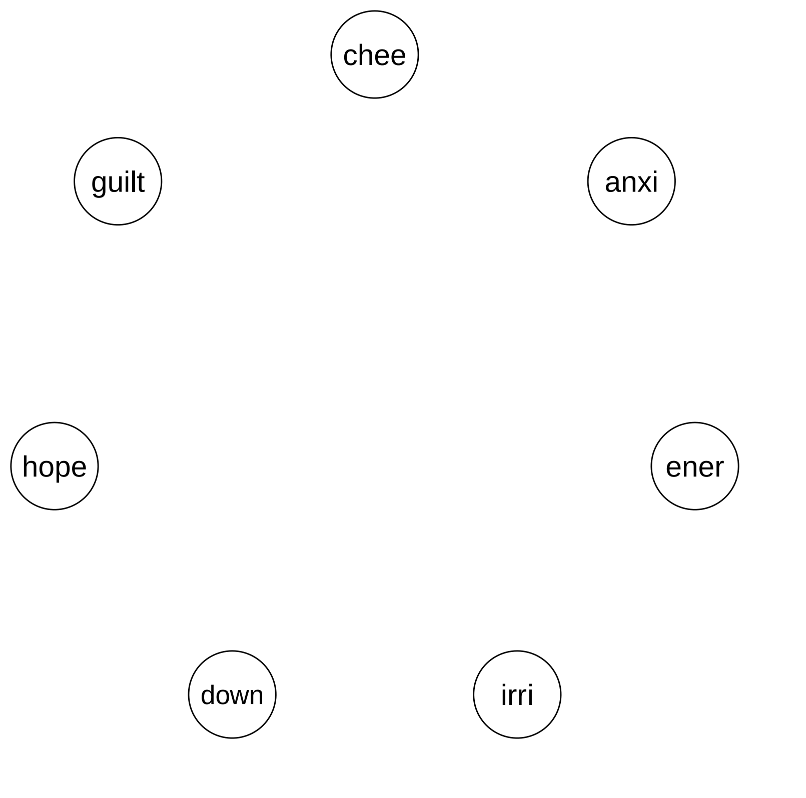

PCT plus ADM reg Pt 269 Estpoint 7

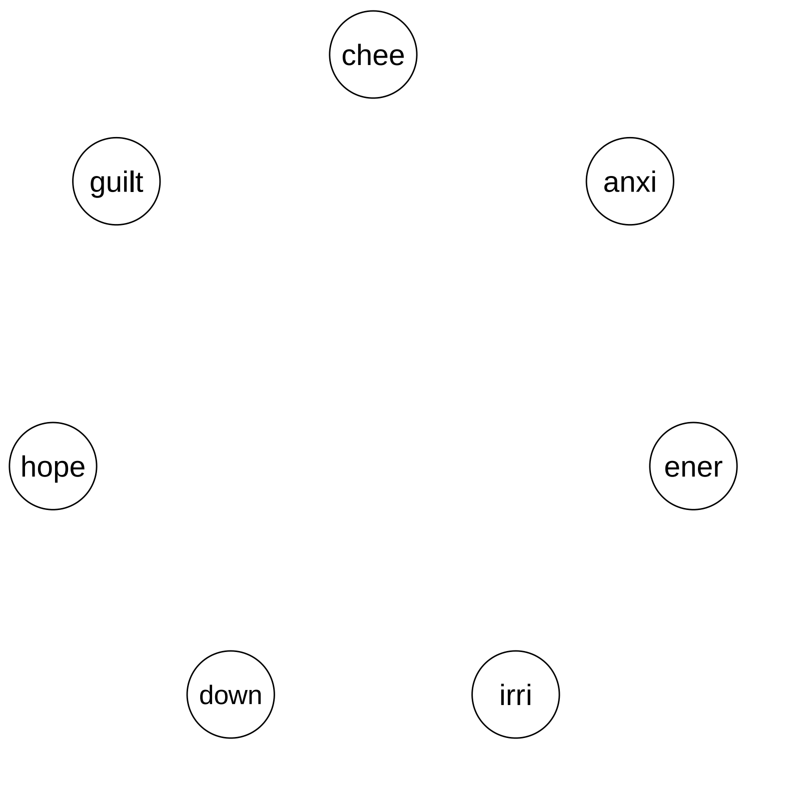

PCT plus ADM reg Pt 269 Estpoint 8

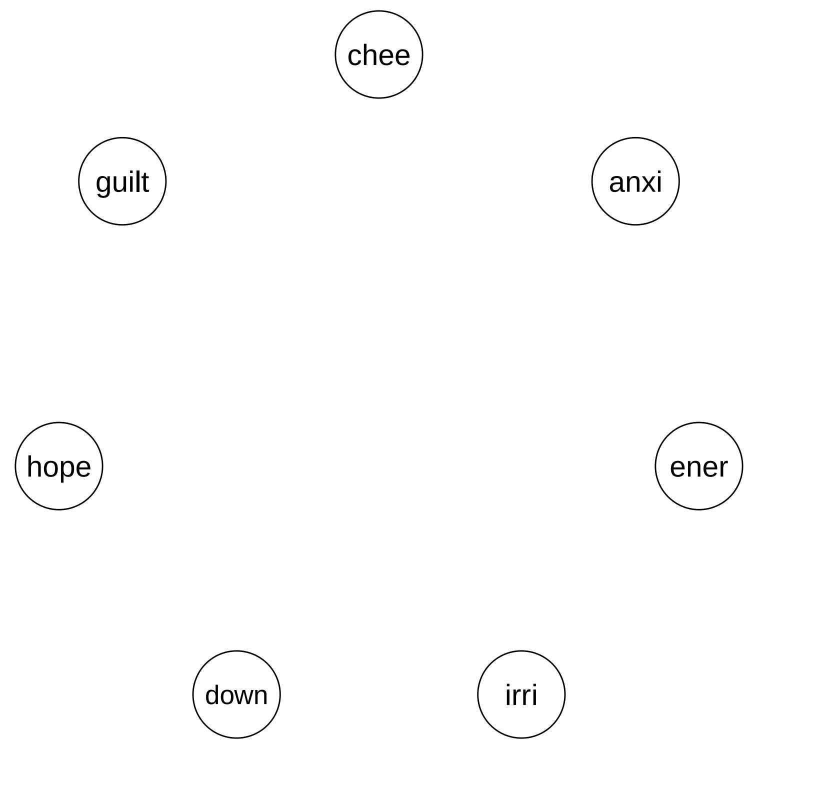

PCT plus ADM reg Pt 258 Estpoint 1

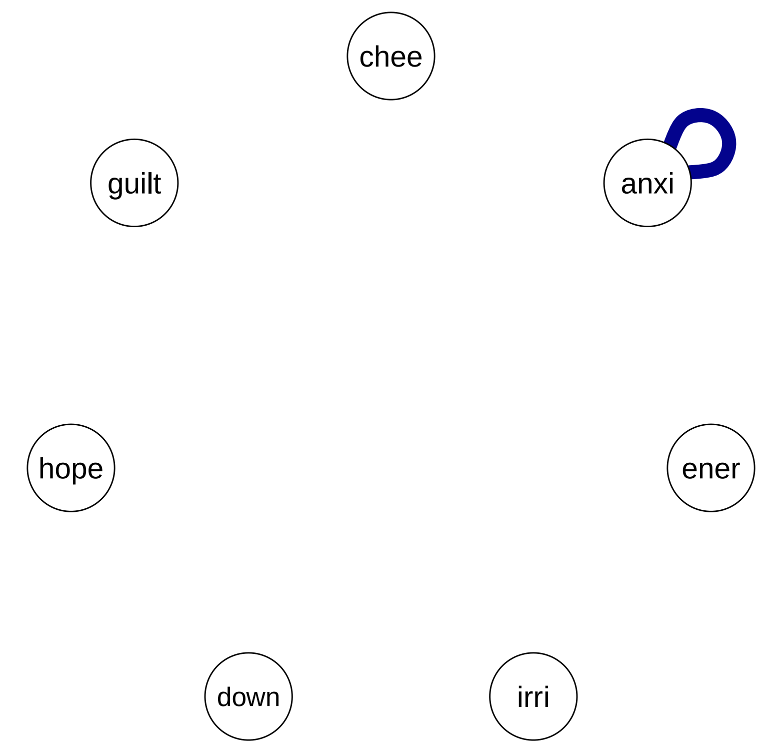

PCT plus ADM reg Pt 258 Estpoint 2

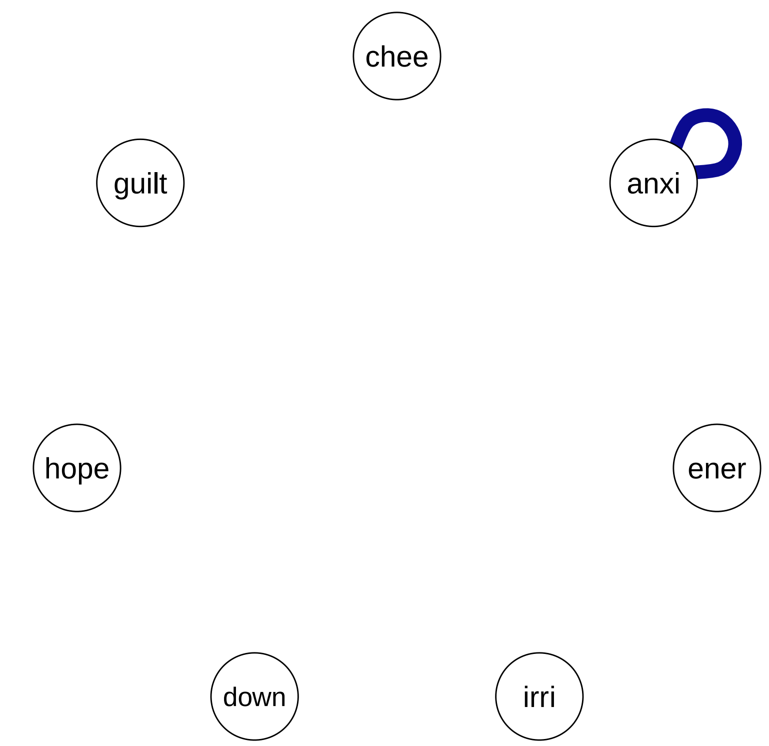

PCT plus ADM reg Pt 258 Estpoint 3

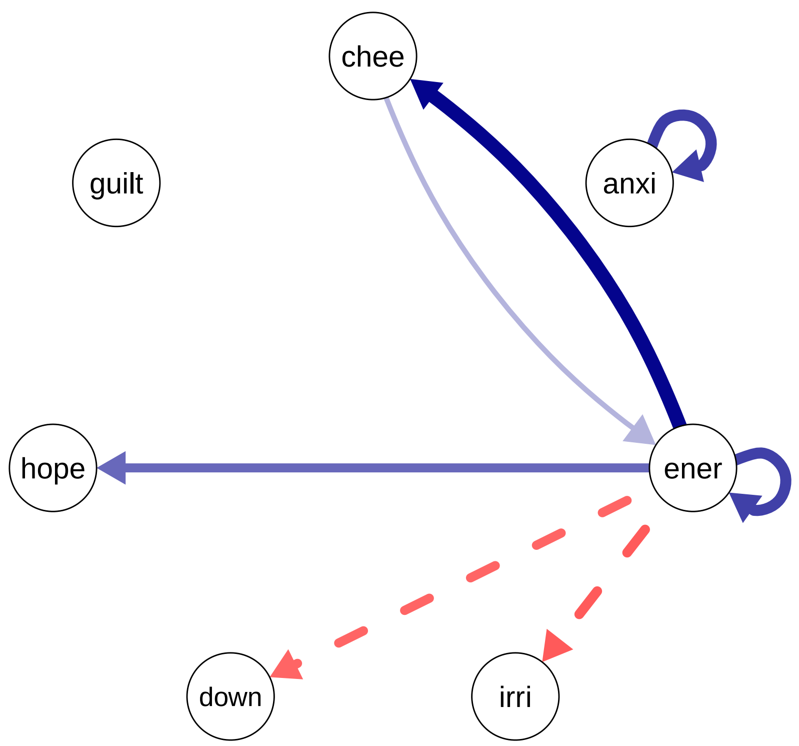

PCT plus ADM reg Pt 258 Estpoint 4

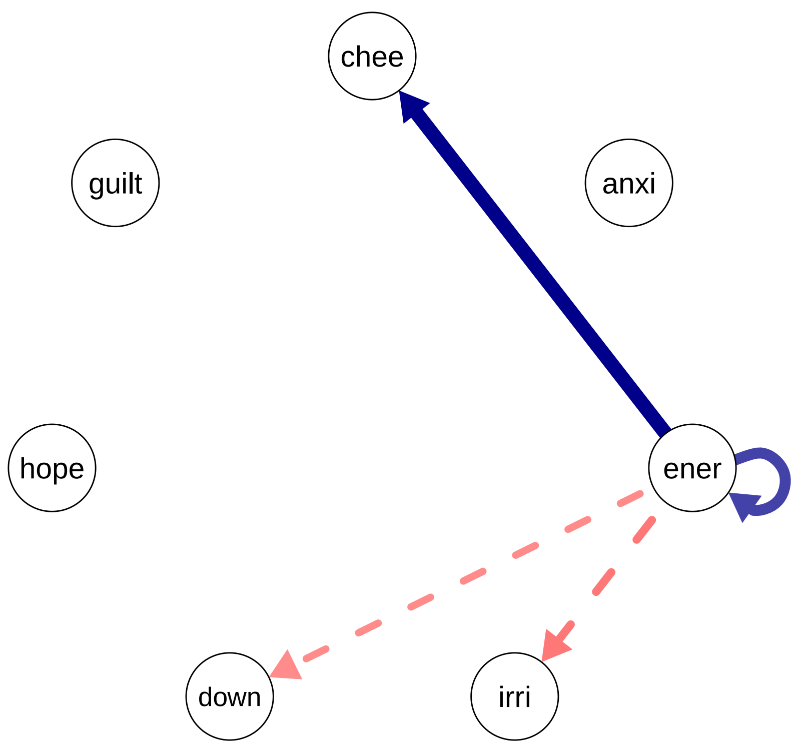

PCT plus ADM reg Pt 258 Estpoint 5

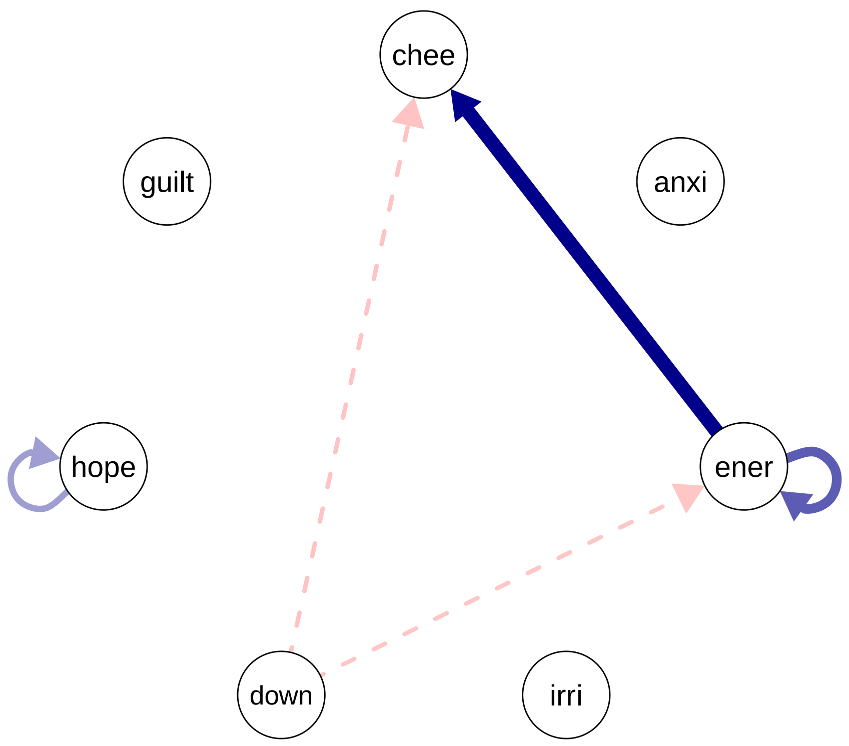

PCT plus ADM reg Pt 258 Estpoint 6

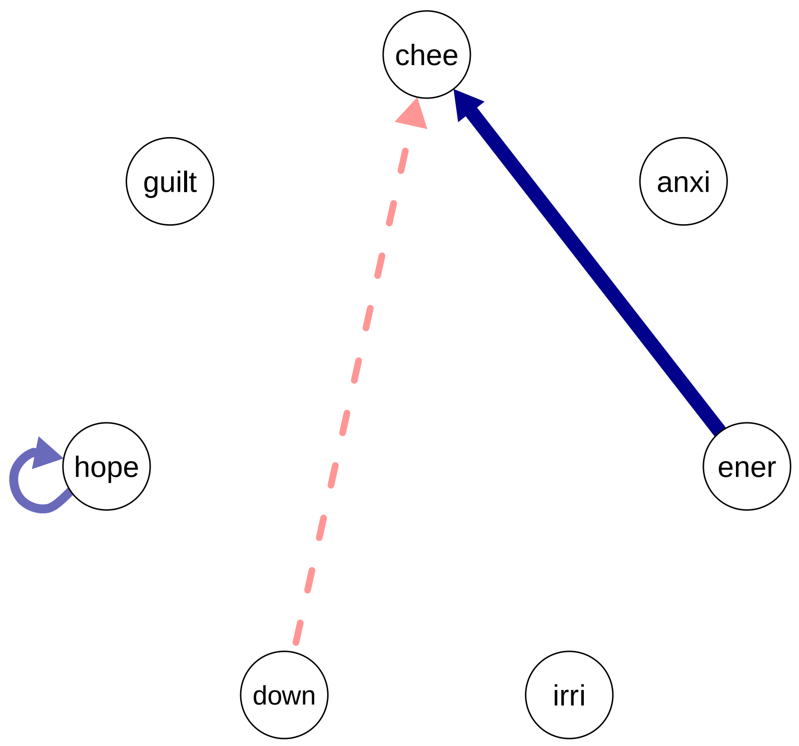

PCT plus ADM reg Pt 258 Estpoint 7

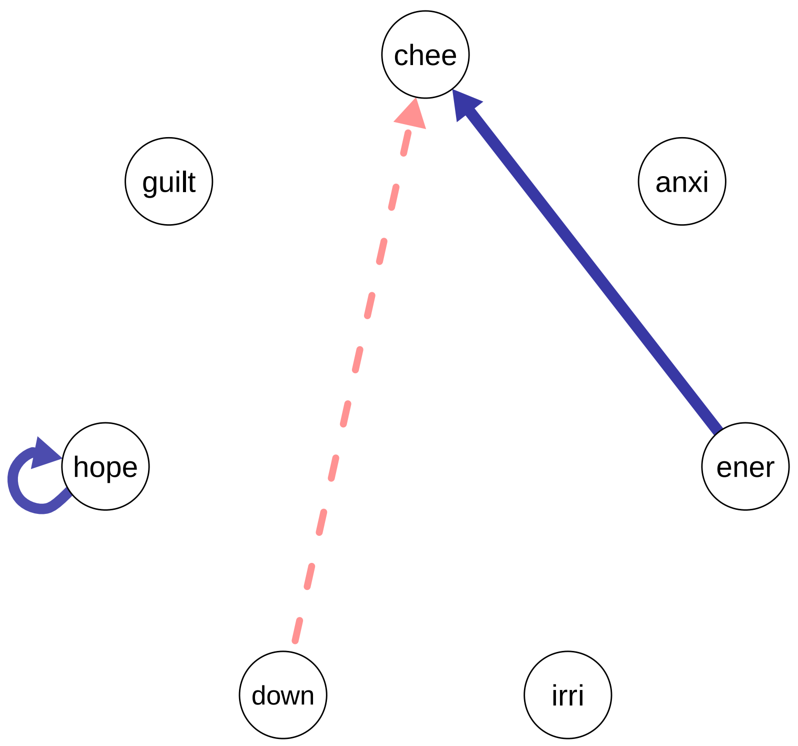

PCT plus ADM reg Pt 258 Estpoint 8

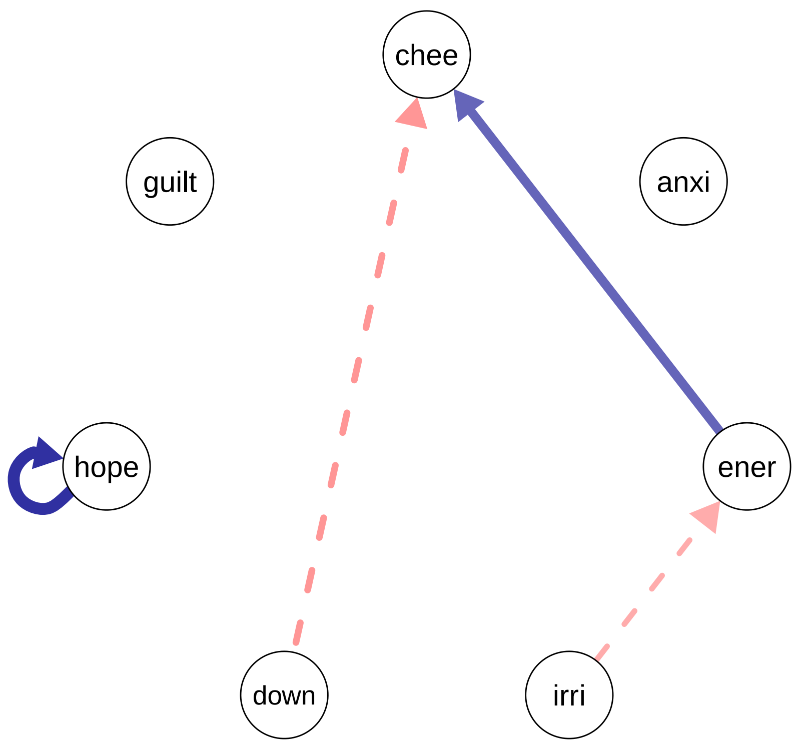

PCT plus ADM reg Pt 290 Estpoint 1

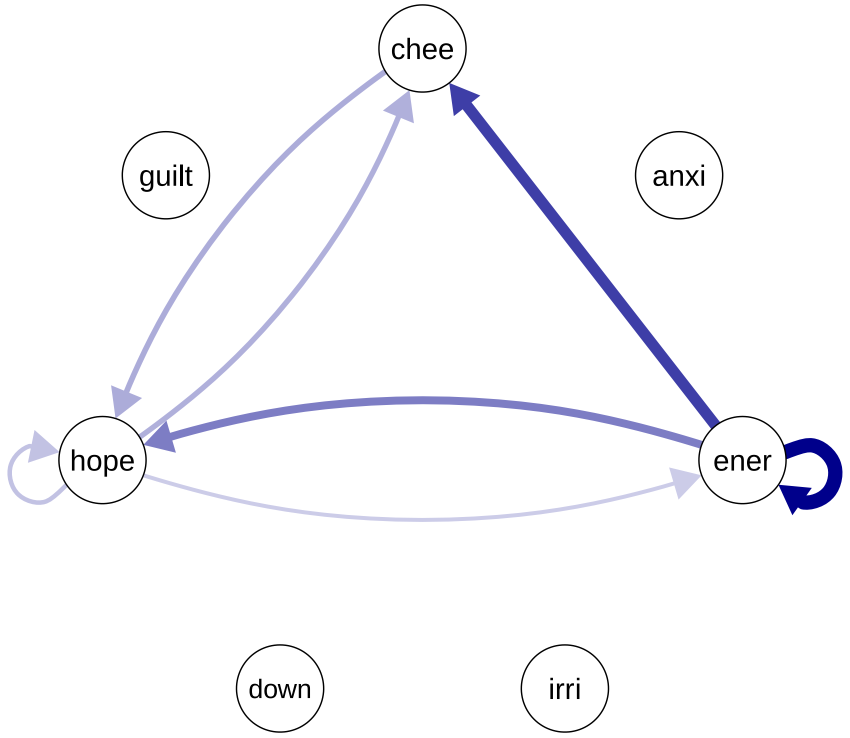

PCT plus ADM reg Pt 290 Estpoint 2

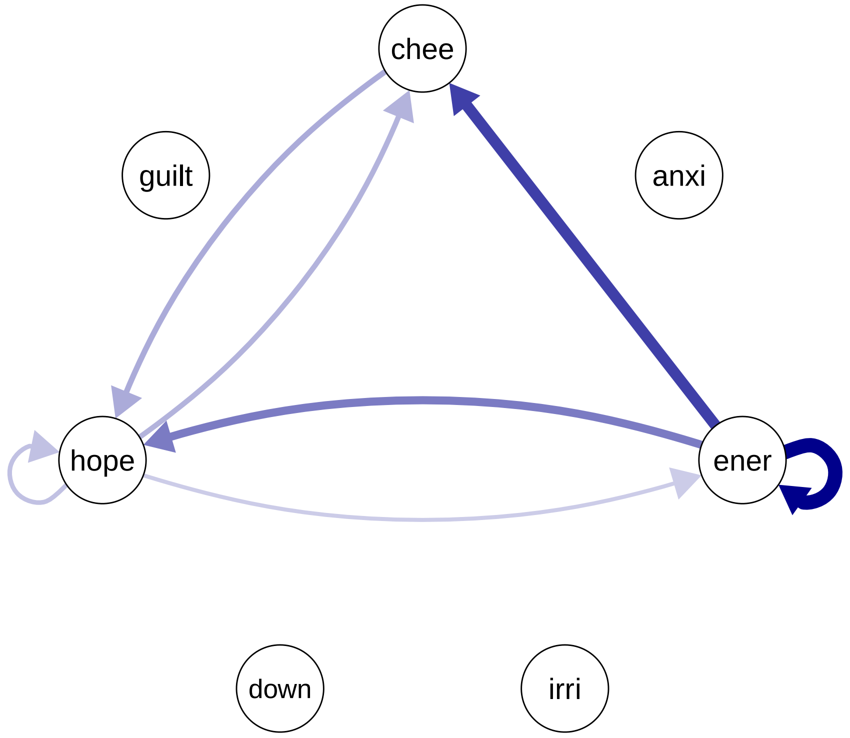

PCT plus ADM reg Pt 290 Estpoint 3

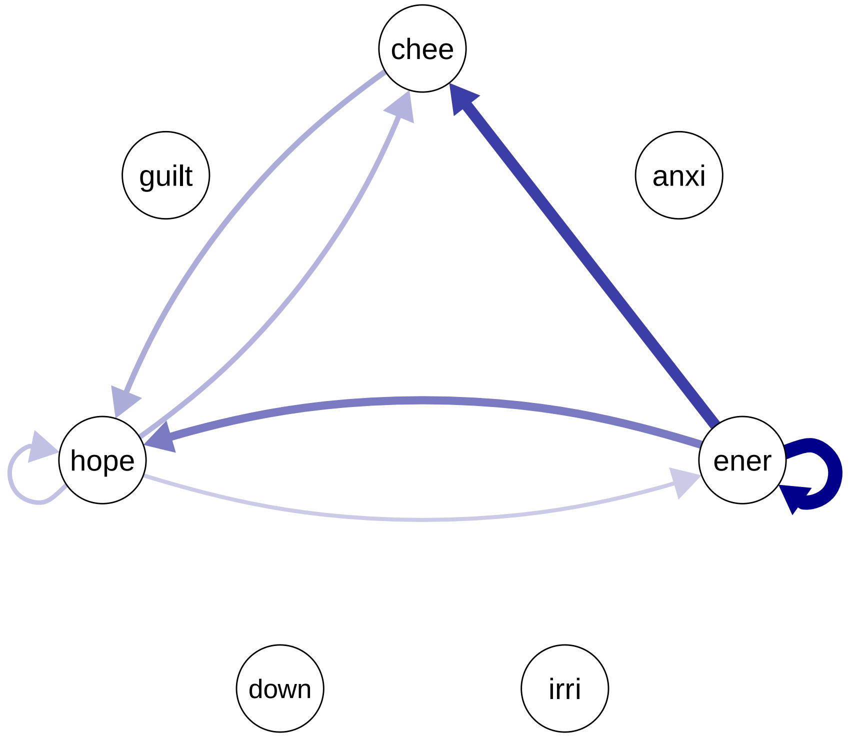

PCT plus ADM reg Pt 290 Estpoint 4

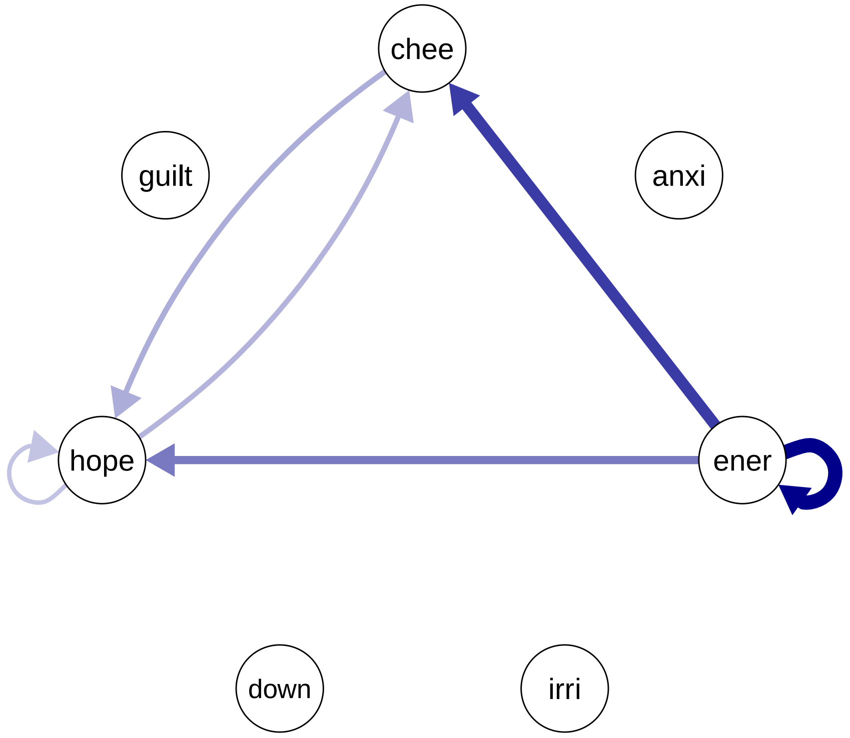

PCT plus ADM reg Pt 290 Estpoint 5

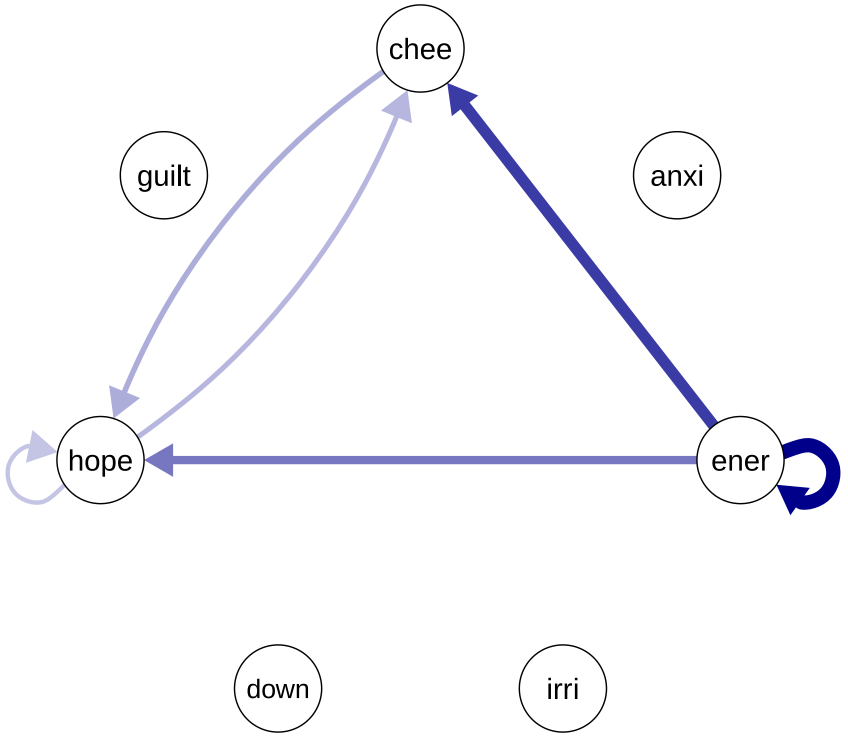

PCT plus ADM reg Pt 290 Estpoint 6

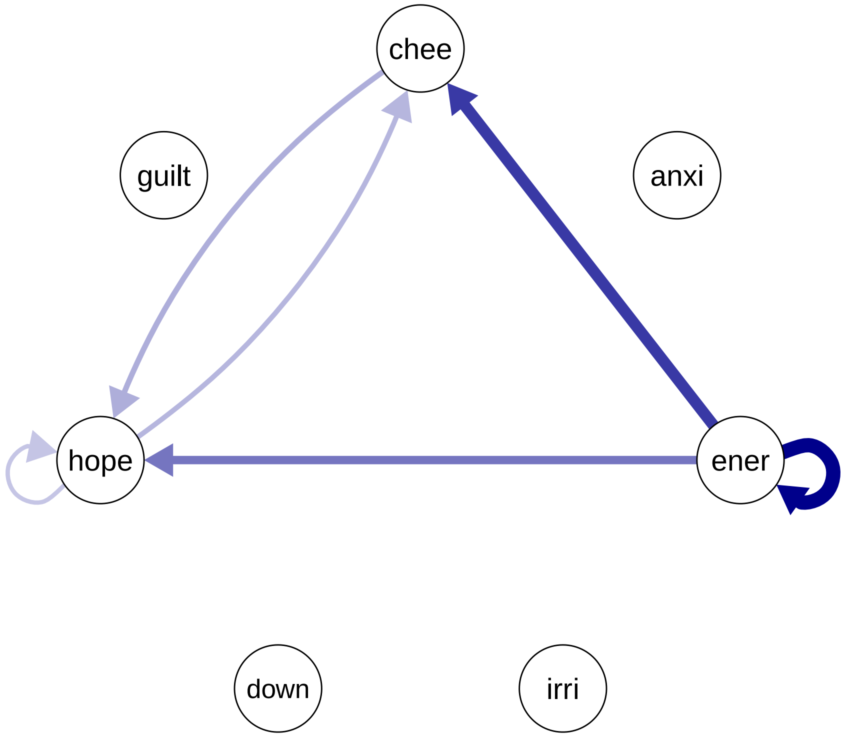

PCT plus ADM reg Pt 290 Estpoint 7

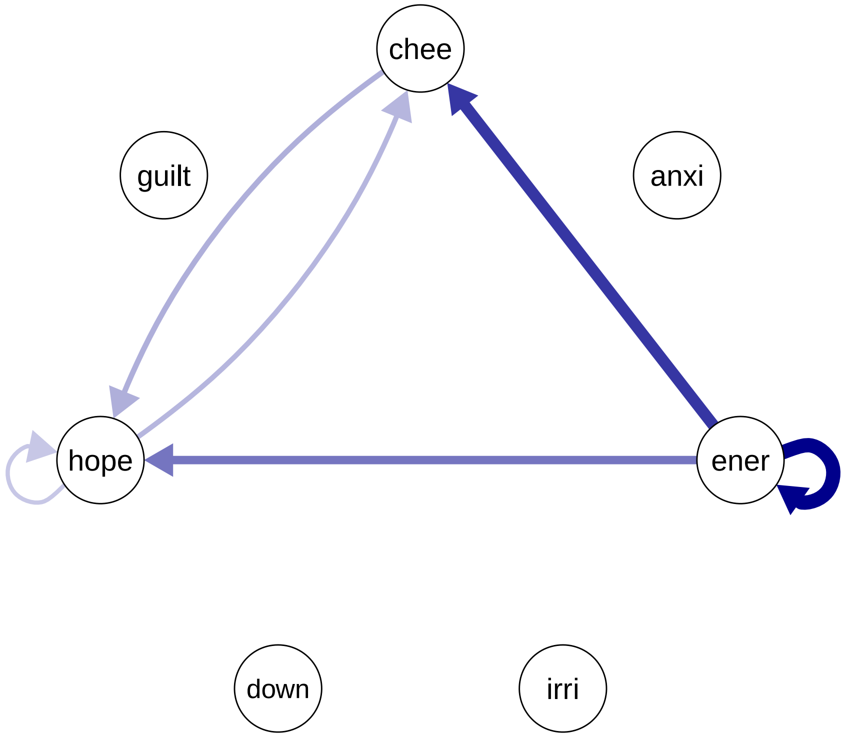

PCT plus ADM reg Pt 290 Estpoint 8

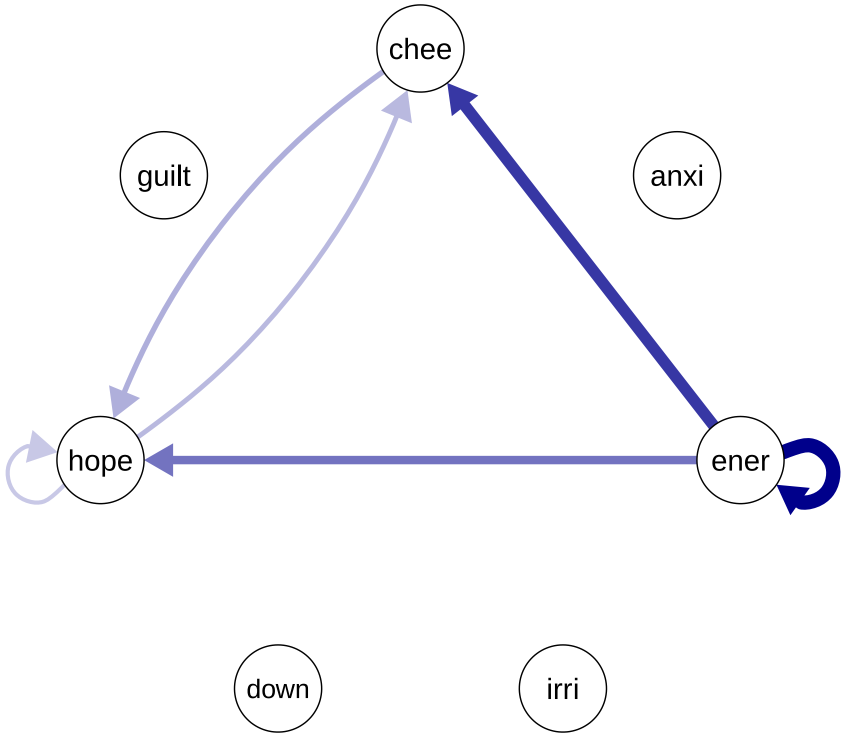

PCT plus ADM reg Pt 232 Estpoint 1

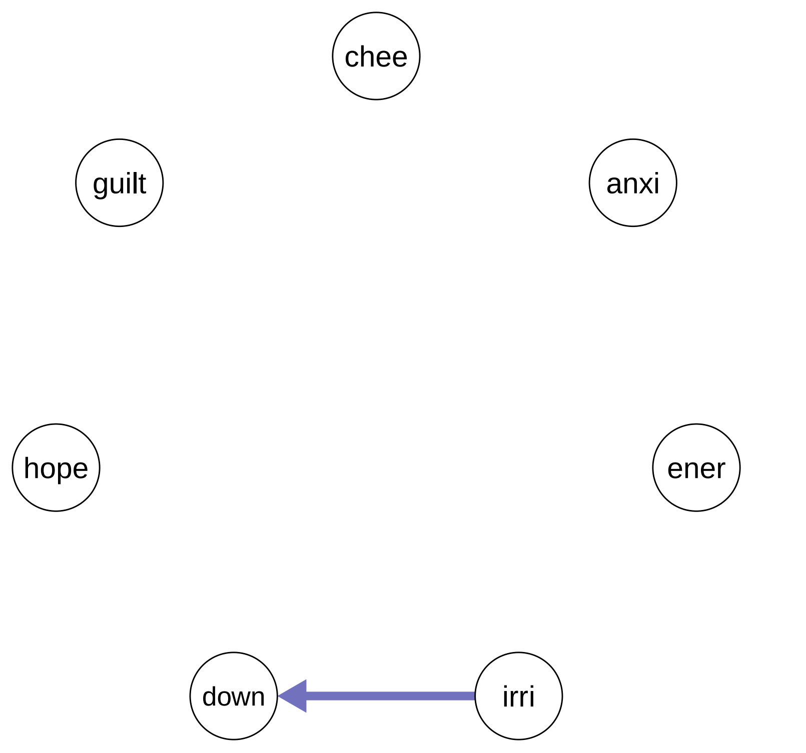

PCT plus ADM reg Pt 232 Estpoint 2

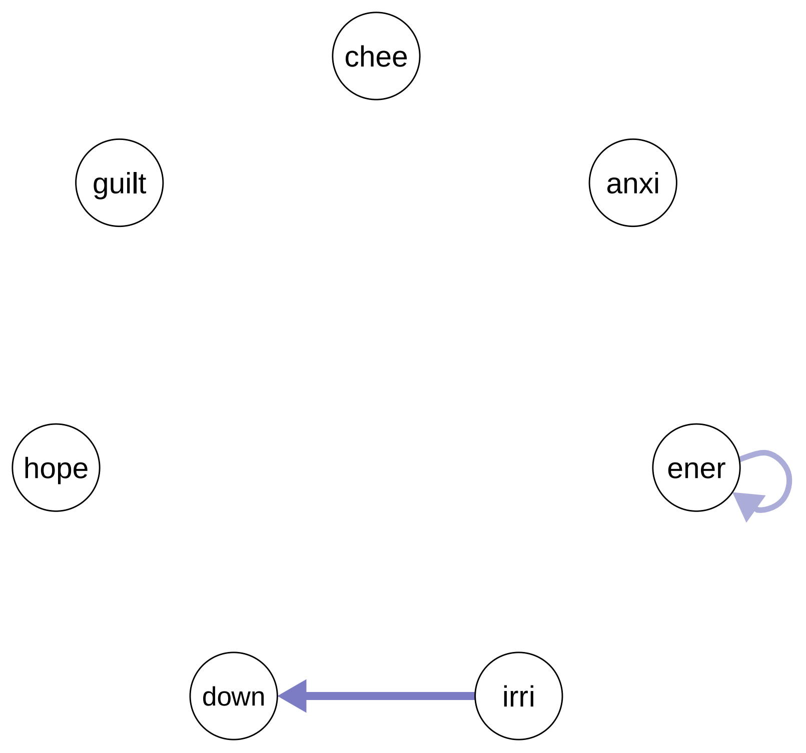

PCT plus ADM reg Pt 232 Estpoint 3

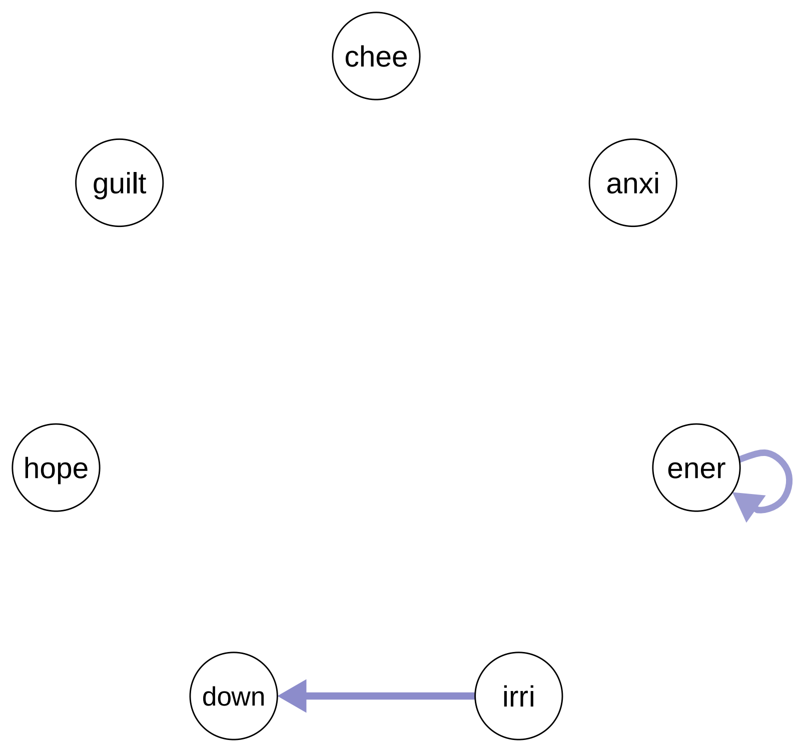

PCT plus ADM reg Pt 232 Estpoint 4

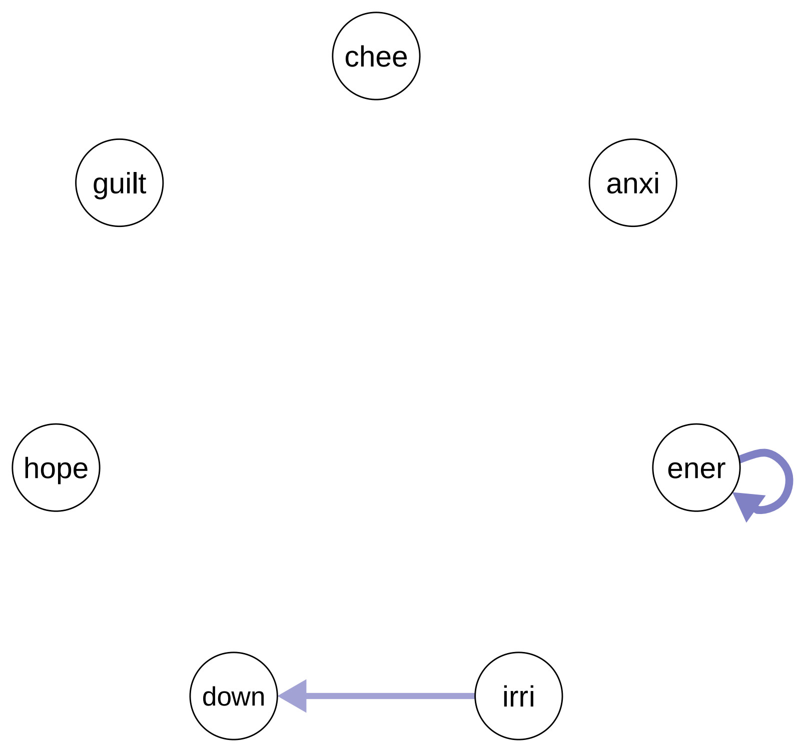

PCT plus ADM reg Pt 232 Estpoint 5

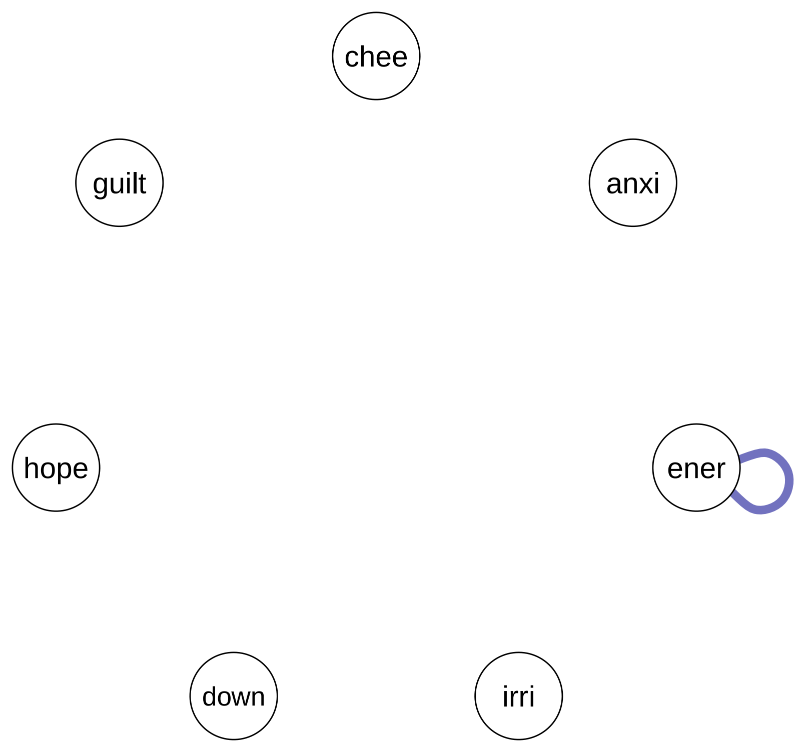

PCT plus ADM reg Pt 232 Estpoint 6

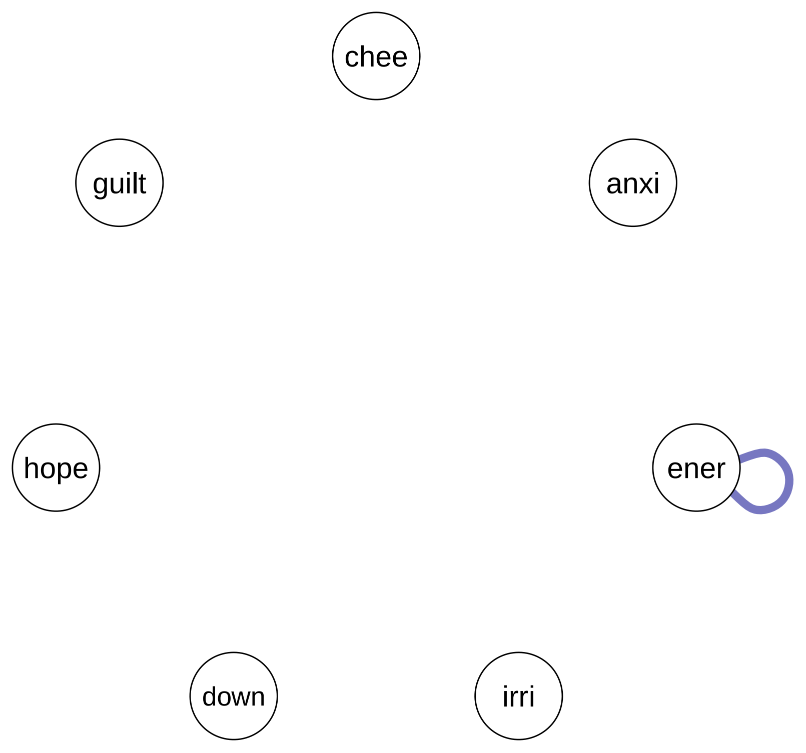

PCT plus ADM reg Pt 232 Estpoint 7

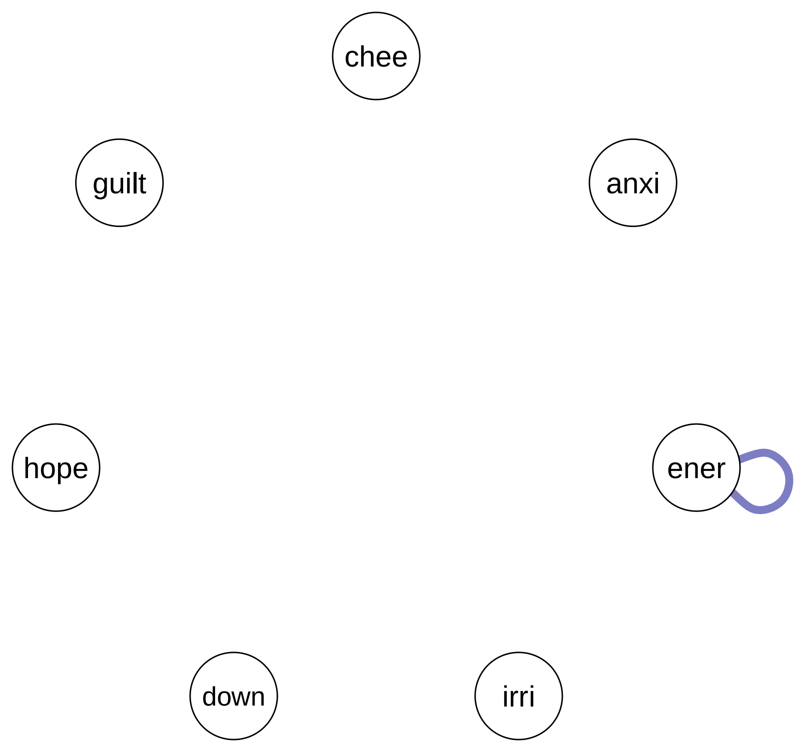

PCT plus ADM reg Pt 232 Estpoint 8

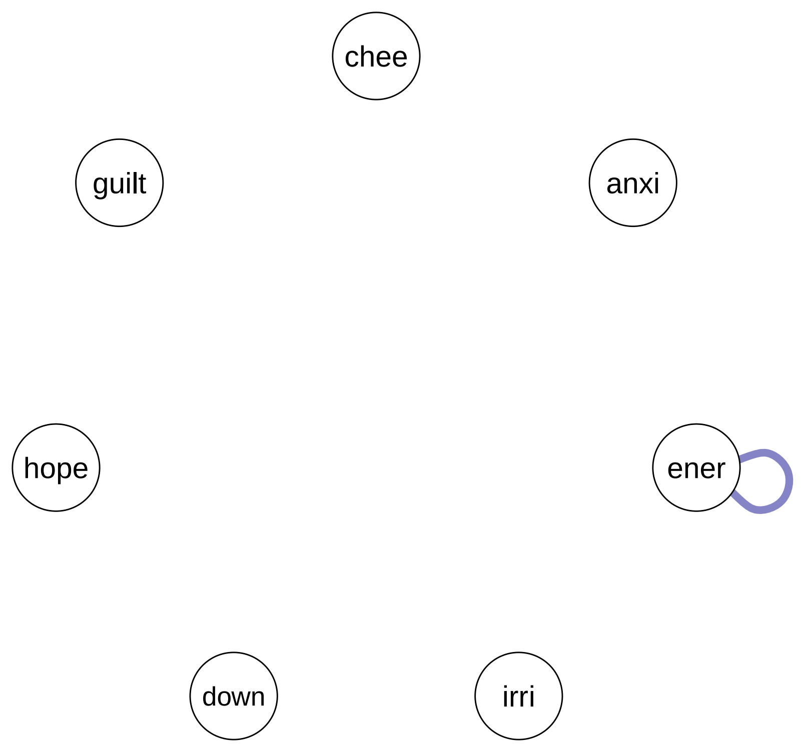

PCT tap ADM reg Pt 266 Estpoint 1

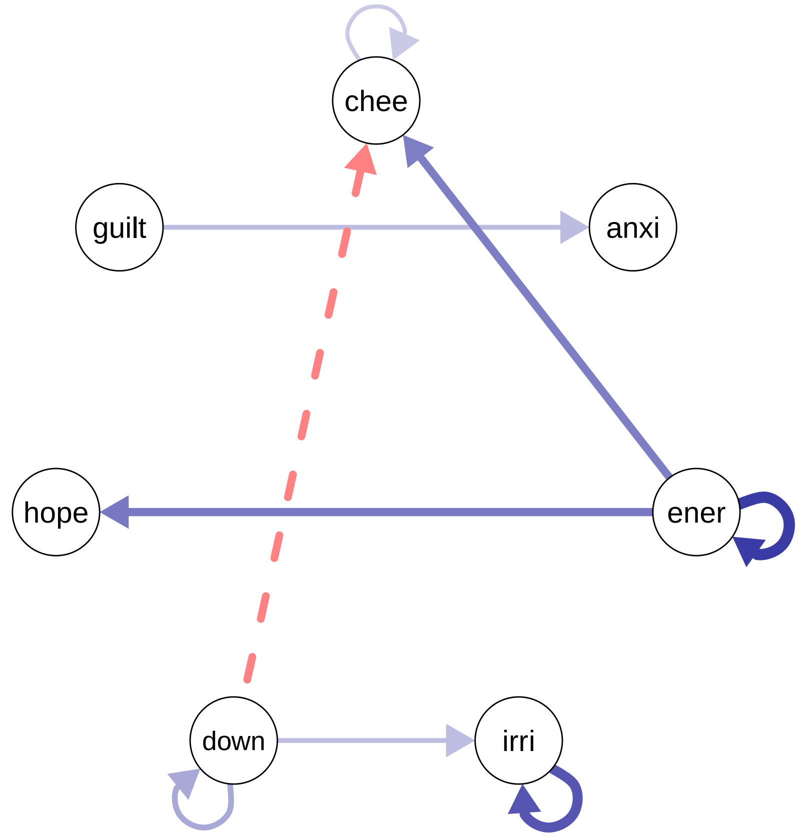

PCT tap ADM reg Pt 266 Estpoint 2

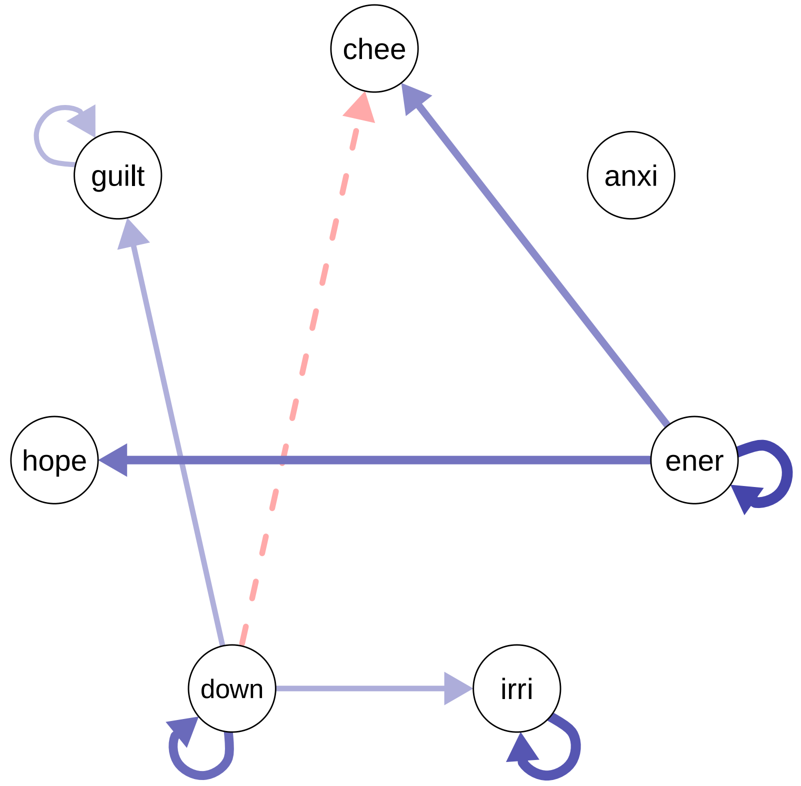

PCT tap ADM reg Pt 266 Estpoint 3

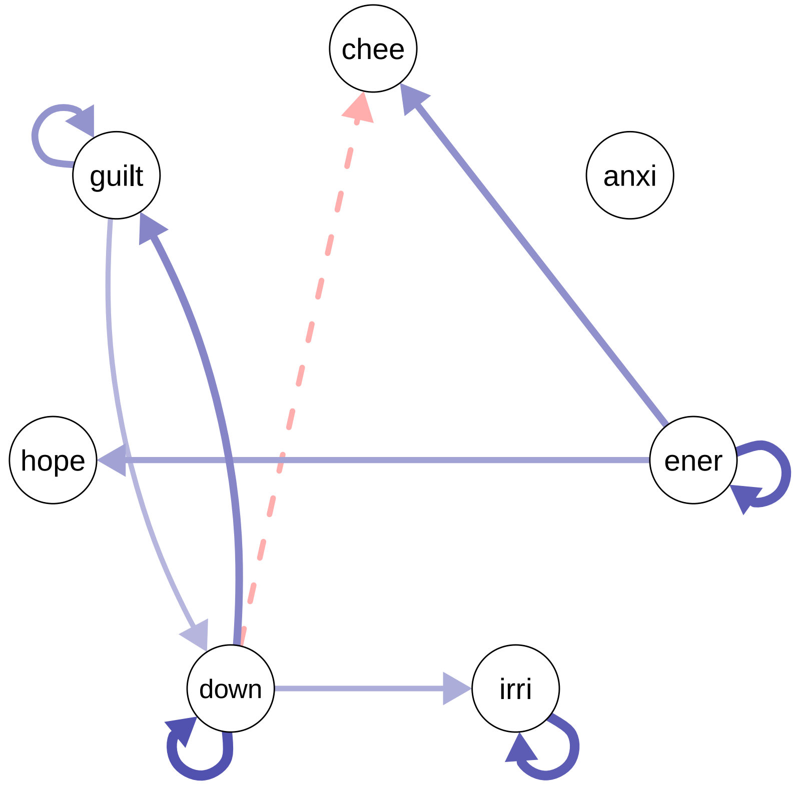

PCT tap ADM reg Pt 266 Estpoint 4

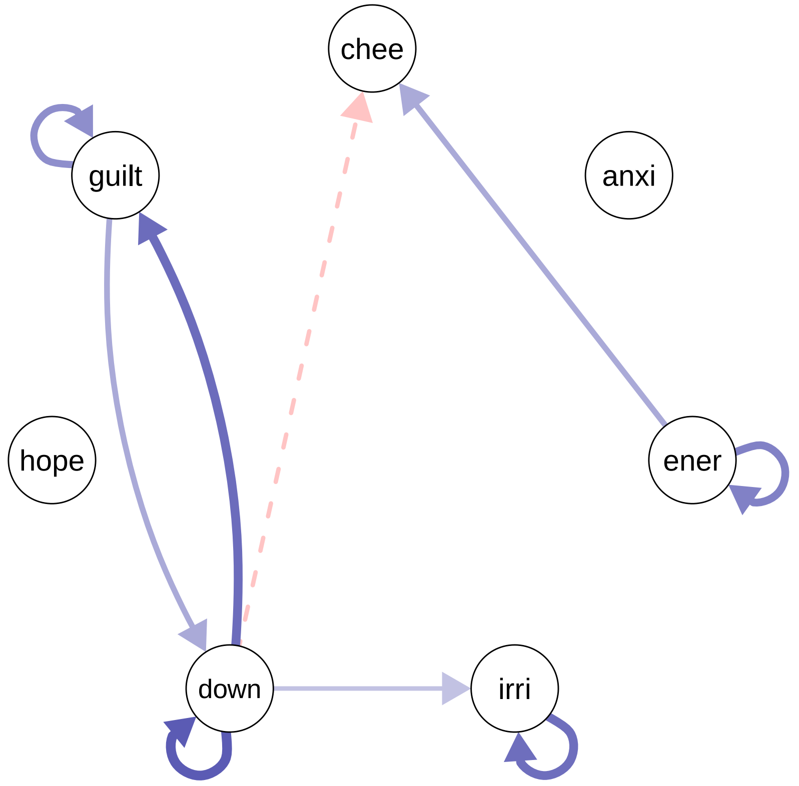

PCT tap ADM reg Pt 266 Estpoint 5

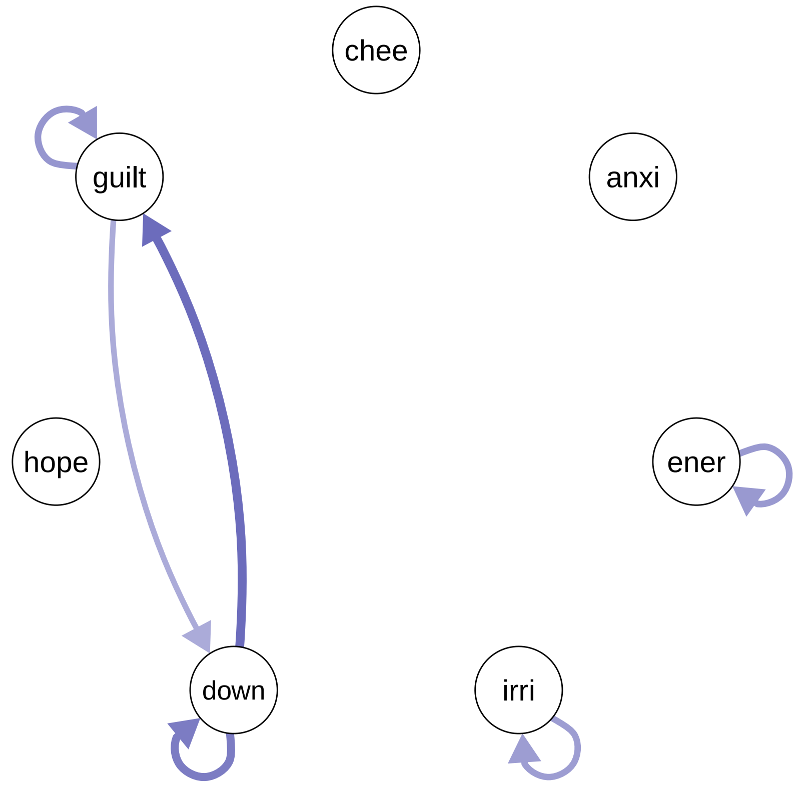

PCT tap ADM reg Pt 266 Estpoint 6

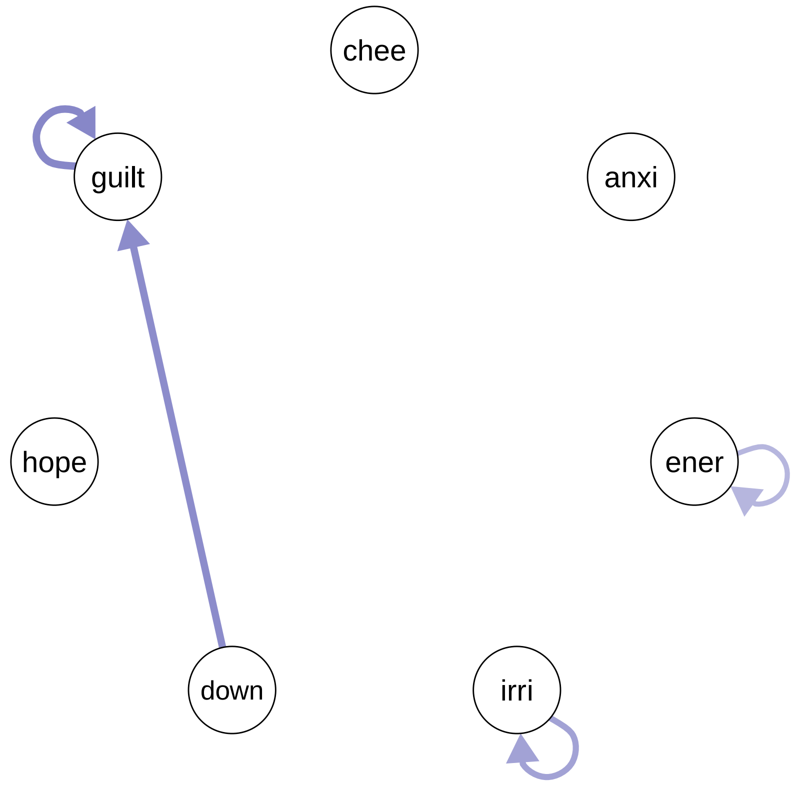

PCT tap ADM reg Pt 266 Estpoint 7

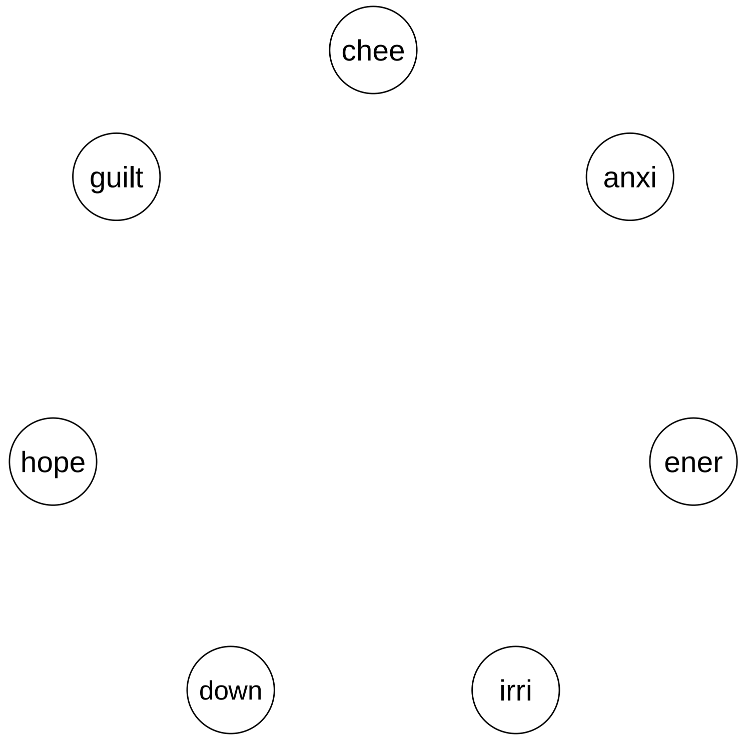

PCT tap ADM reg Pt 266 Estpoint 8

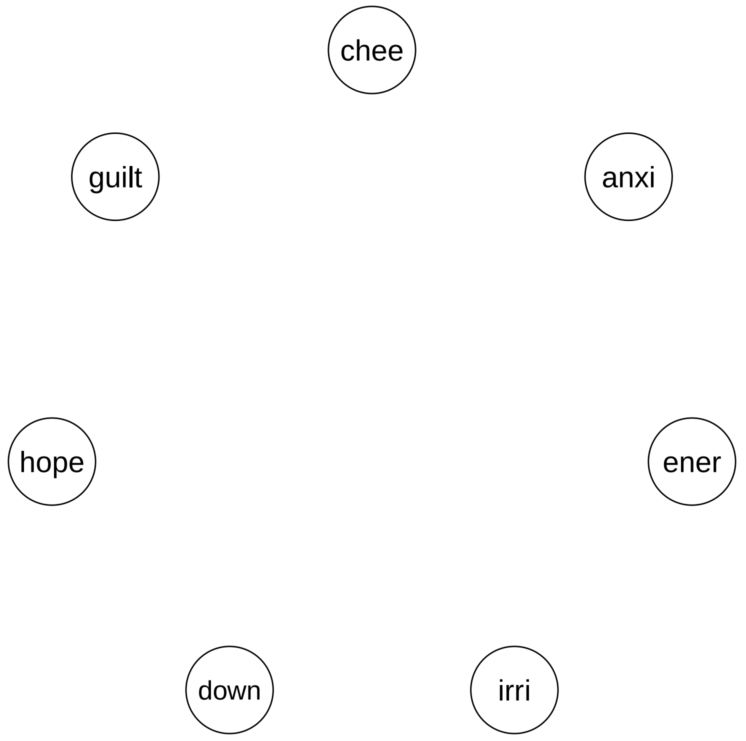

PCT tap ADM reg Pt 277 Estpoint 1

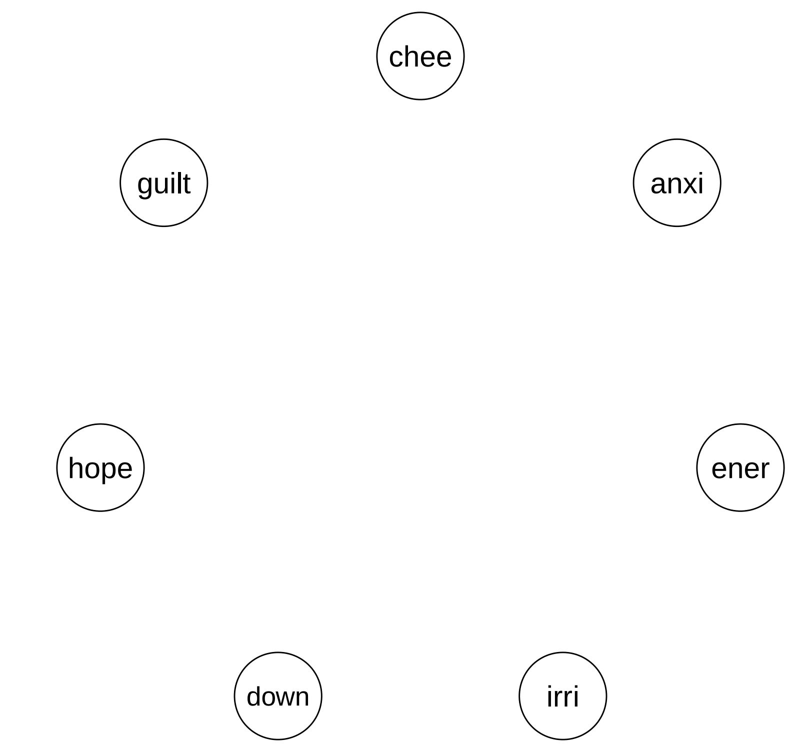

PCT tap ADM reg Pt 277 Estpoint 2

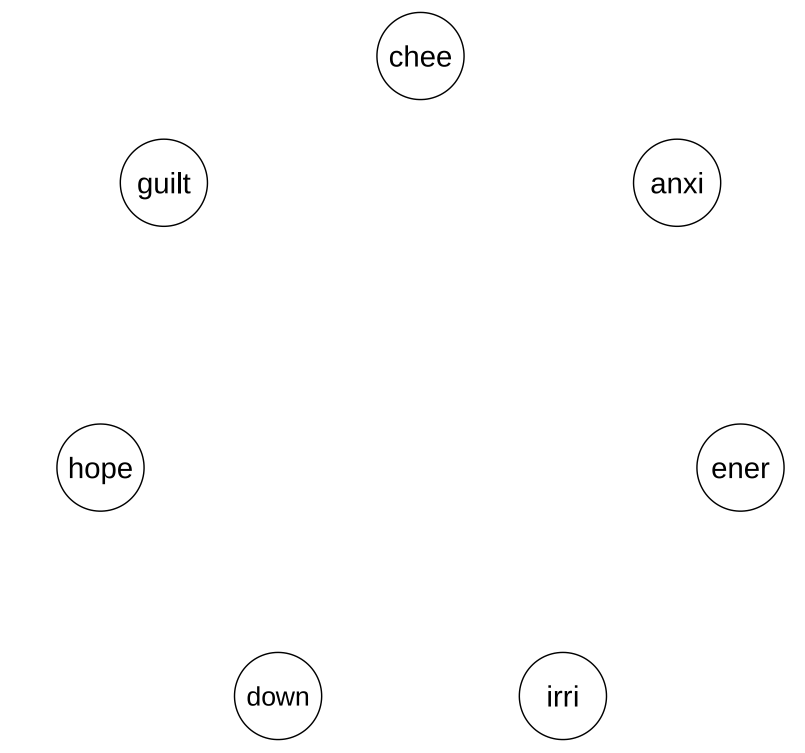

PCT tap ADM reg Pt 277 Estpoint 3

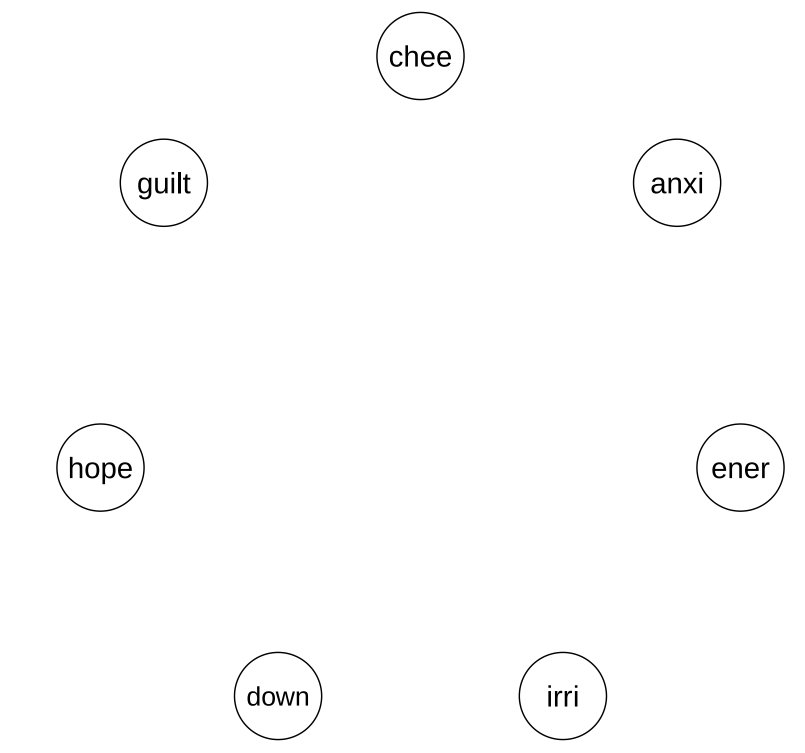

PCT tap ADM reg Pt 277 Estpoint 4

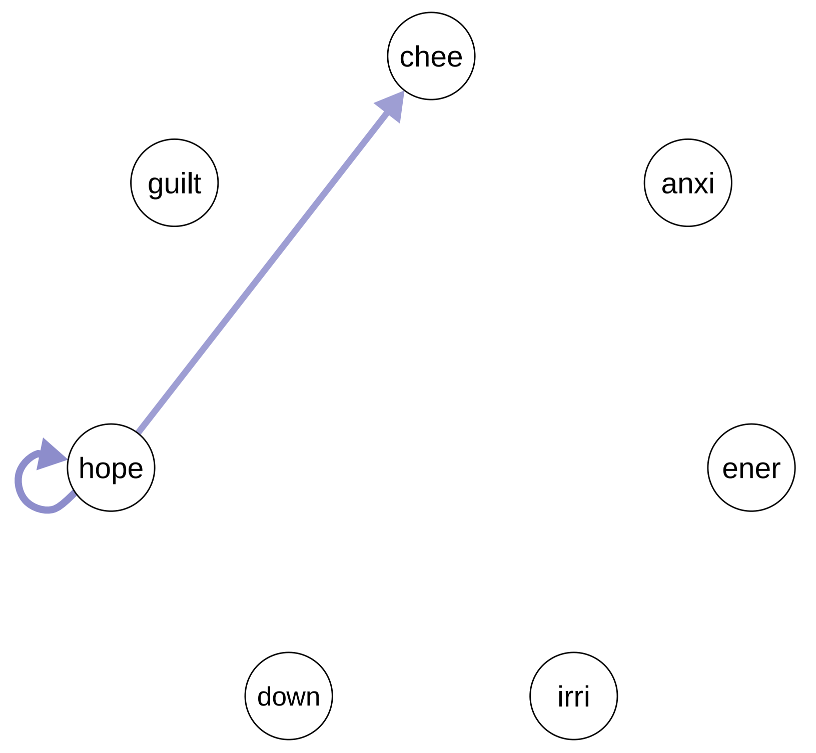

PCT tap ADM reg Pt 277 Estpoint 5

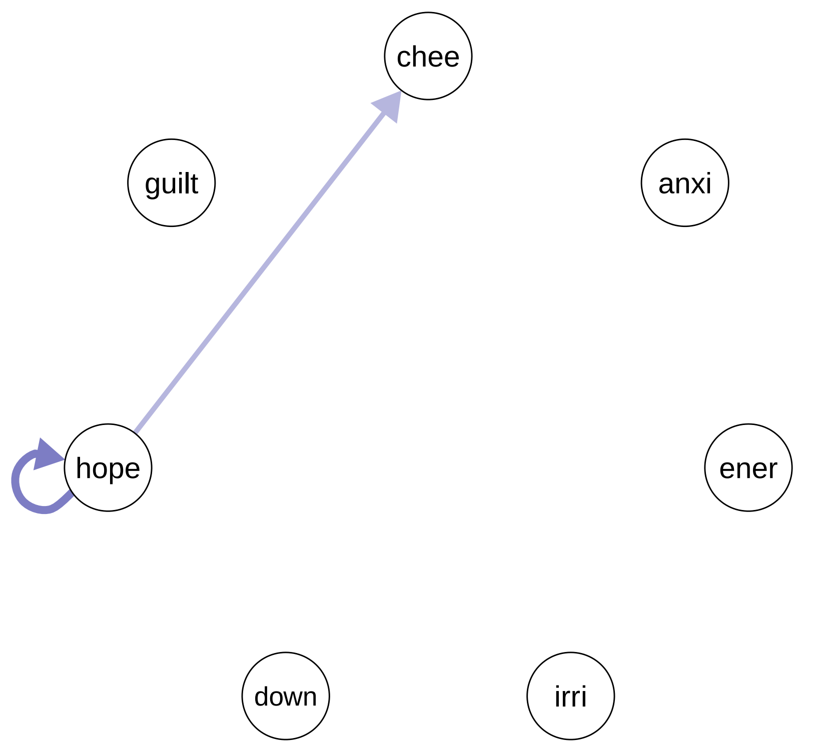

PCT tap ADM reg Pt 277 Estpoint 6

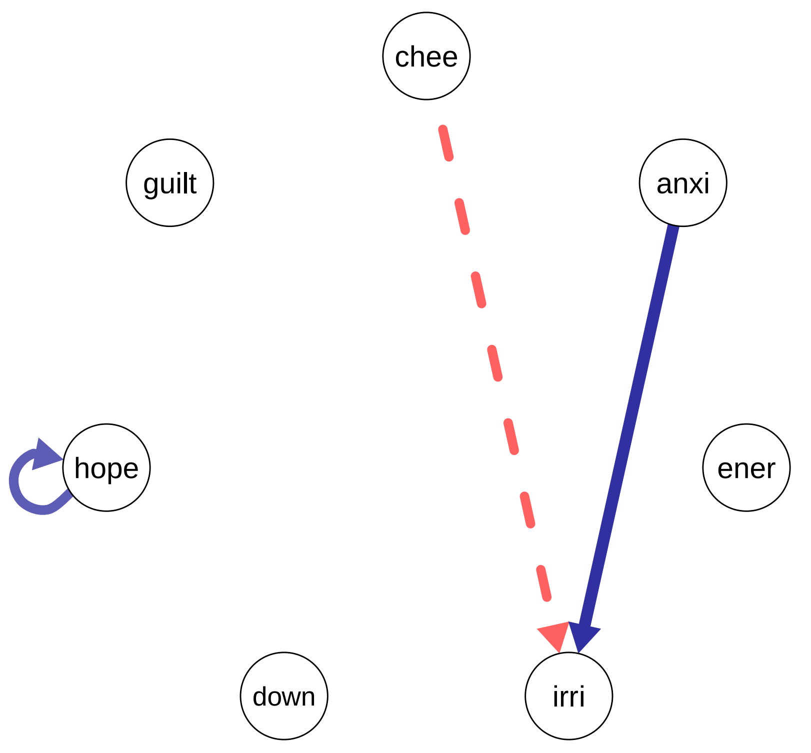

PCT tap ADM reg Pt 277 Estpoint 7

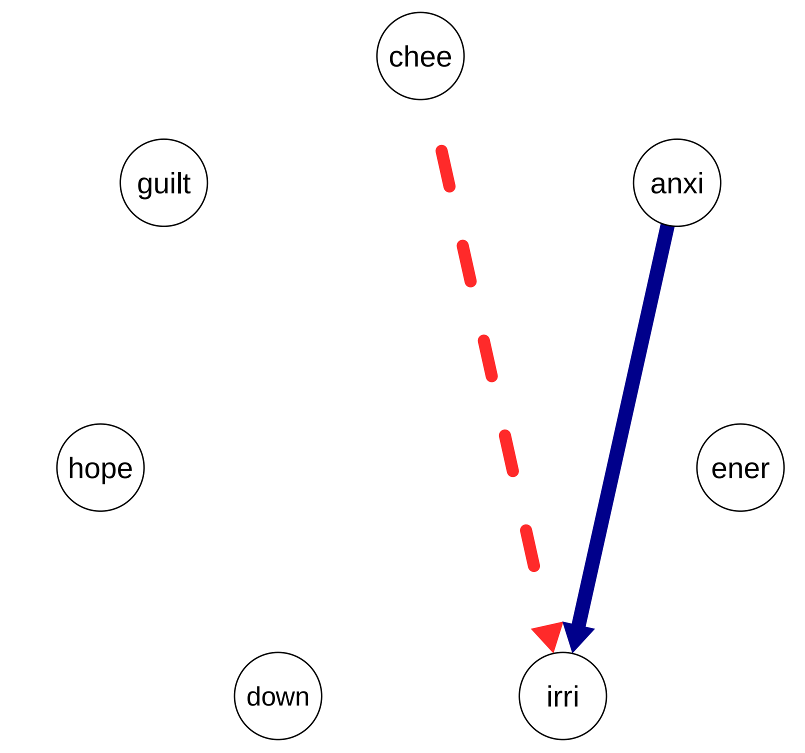

PCT tap ADM reg Pt 277 Estpoint 8

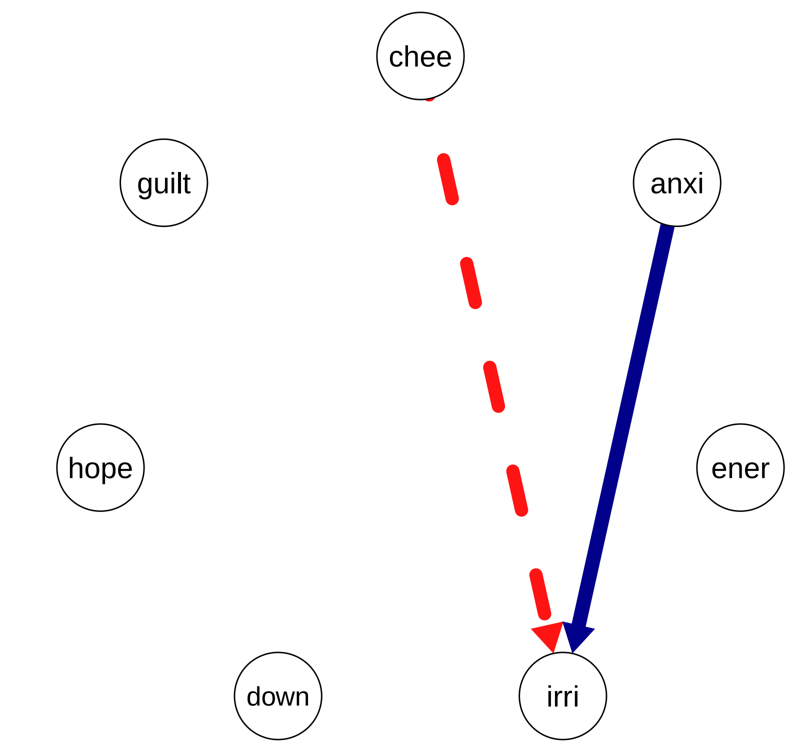

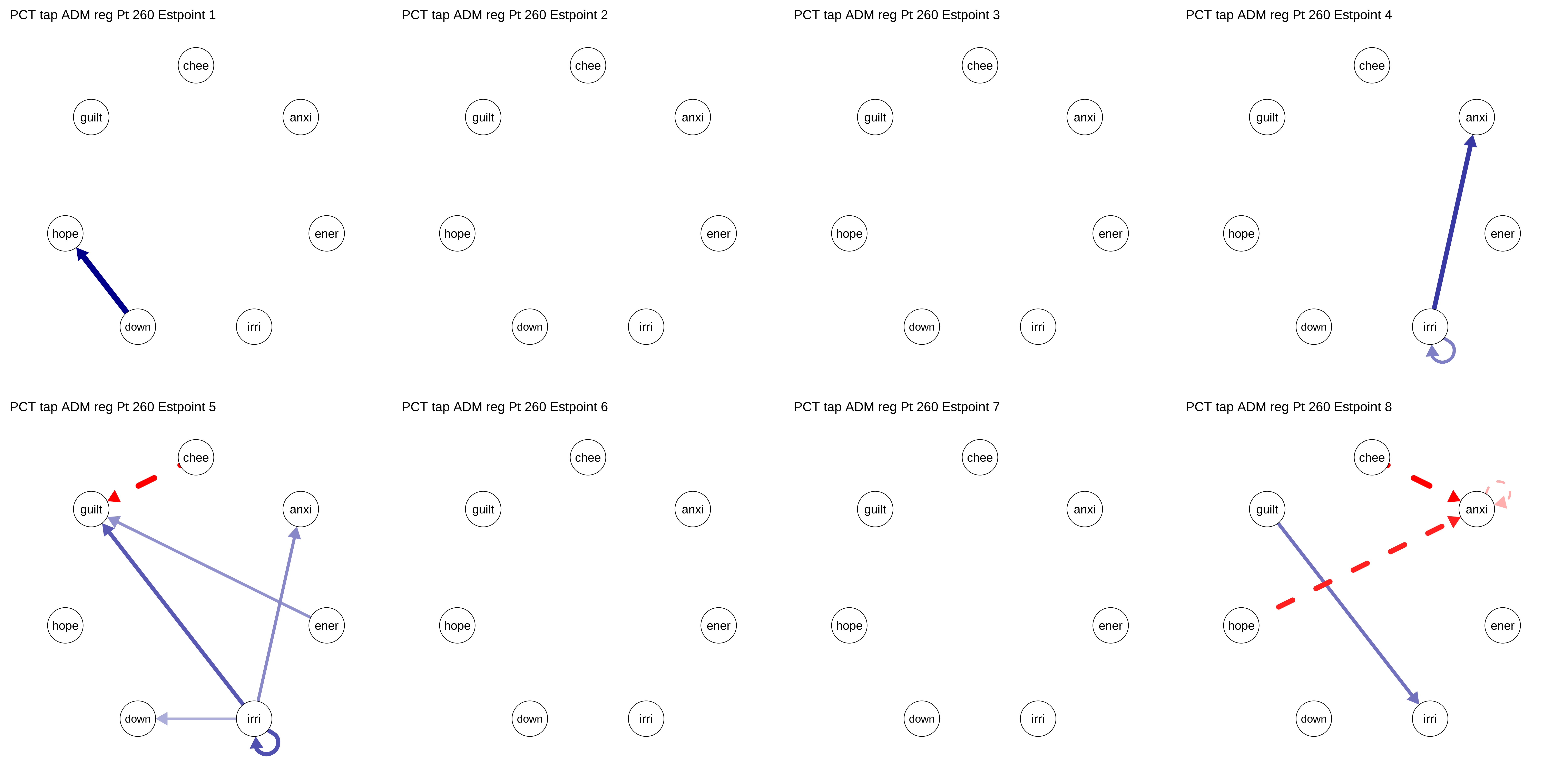
PCT tap ADM reg Pt 260 Estpoint 1
chee
guilt
anxi
hope
ener
down
irri
PCT tap ADM reg Pt 260 Estpoint 2
chee
guilt
anxi
hope
ener
down
irri
PCT tap ADM reg Pt 260 Estpoint 3
chee
guilt
anxi
hope
ener
down
irri
PCT tap ADM reg Pt 260 Estpoint 4
chee
guilt
anxi
hope
ener
down
irri
PCT tap ADM reg Pt 260 Estpoint 5
chee
guilt
anxi
hope
ener
down
irri
PCT tap ADM reg Pt 260 Estpoint 6
chee
guilt
anxi
hope
ener
down
irri
PCT tap ADM reg Pt 260 Estpoint 7
chee
guilt
anxi
hope
ener
down
irri
PCT tap ADM reg Pt 260 Estpoint 8
chee
guilt
anxi
hope
ener
down
irri

PCT tap ADM reg Pt 253 Estpoint 1

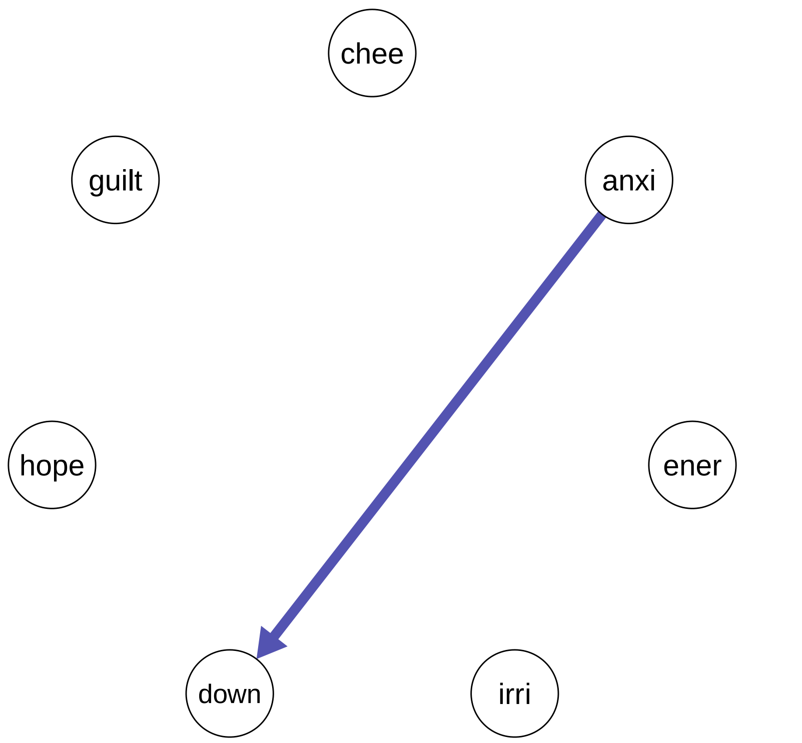

PCT tap ADM reg Pt 253 Estpoint 2

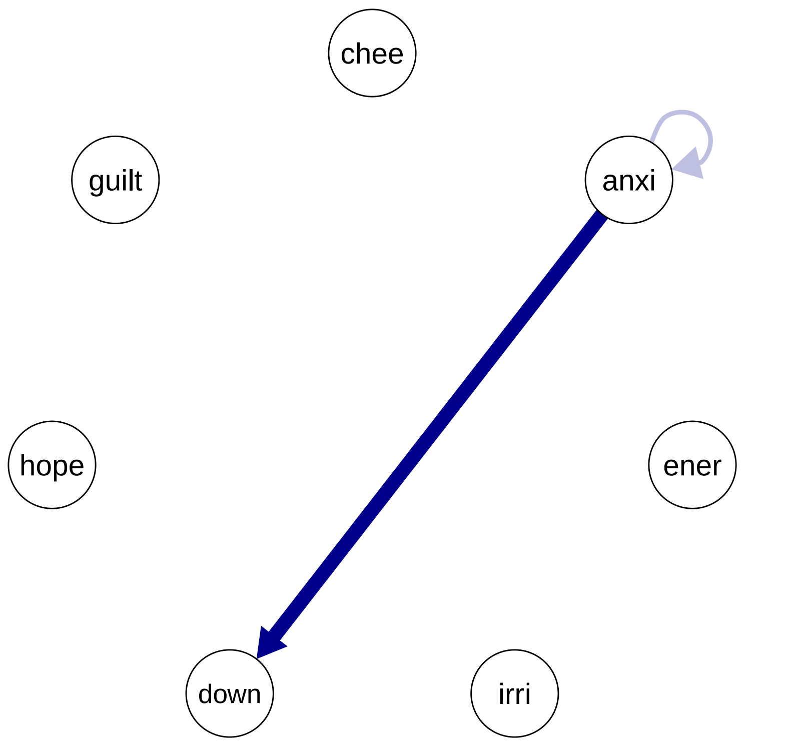

PCT tap ADM reg Pt 253 Estpoint 3

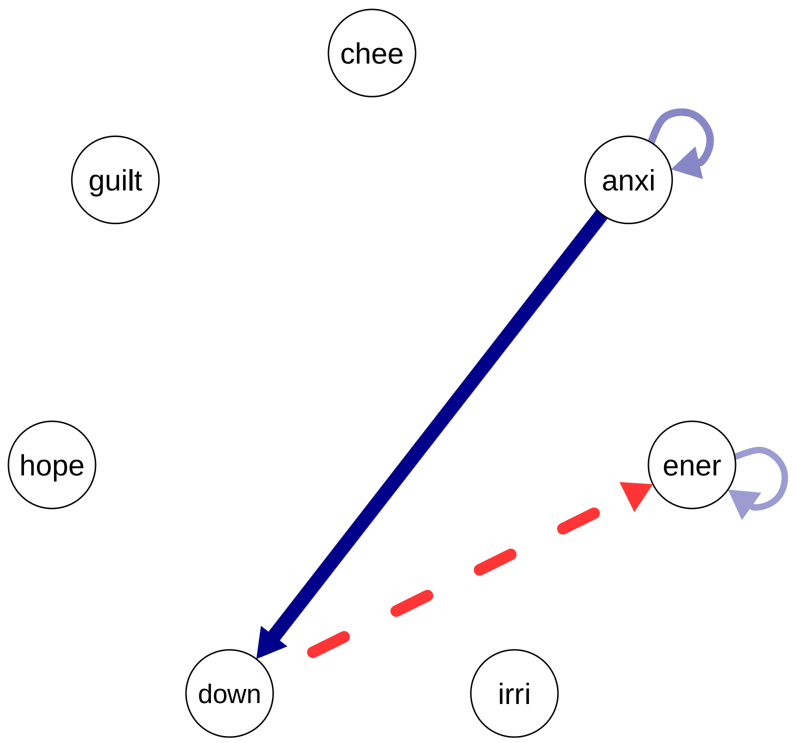

PCT tap ADM reg Pt 253 Estpoint 4

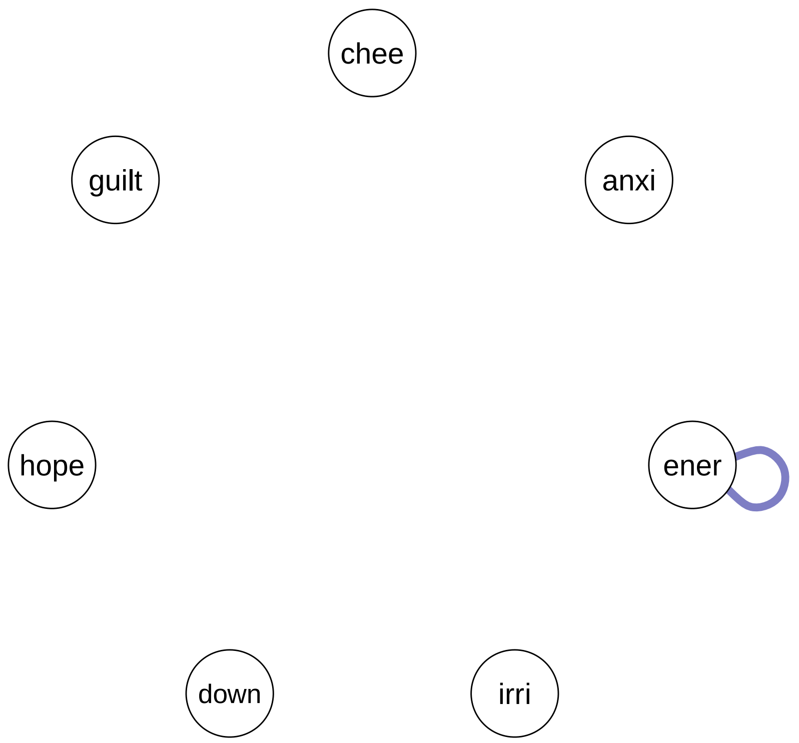

PCT tap ADM reg Pt 253 Estpoint 5

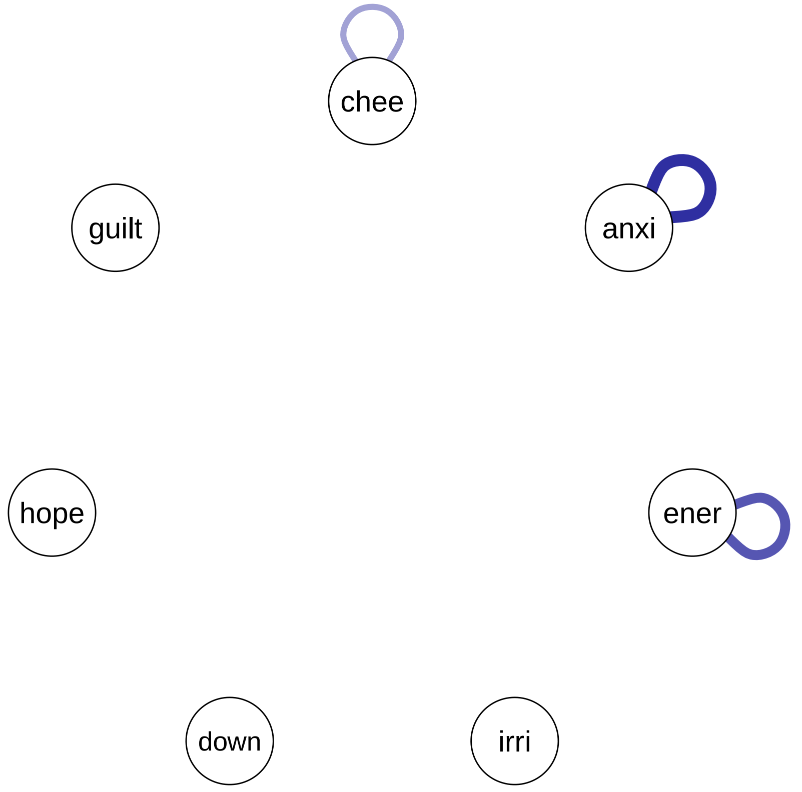

PCT tap ADM reg Pt 253 Estpoint 6

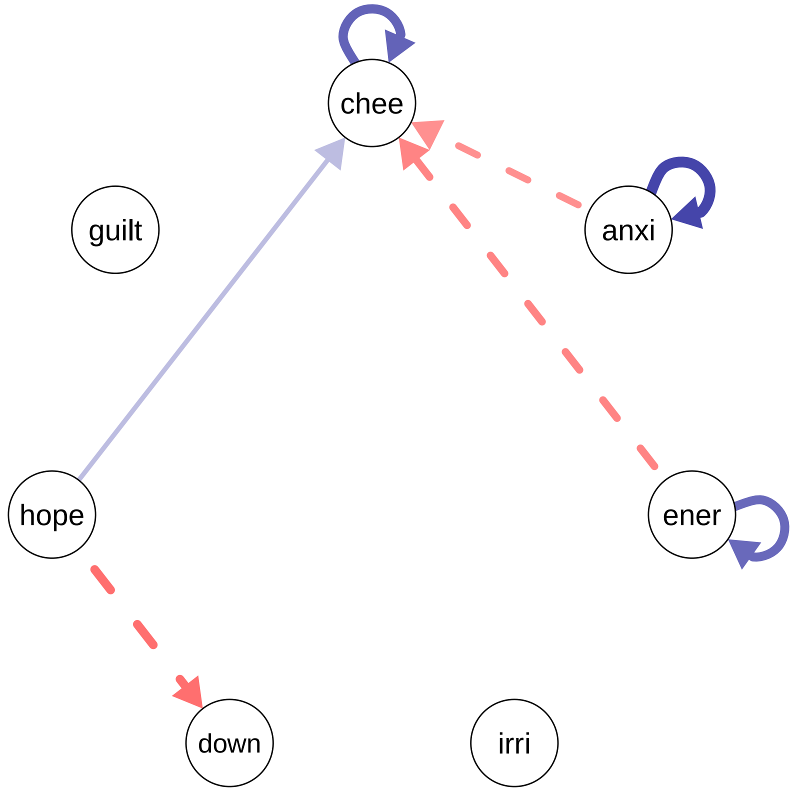

PCT tap ADM reg Pt 253 Estpoint 7

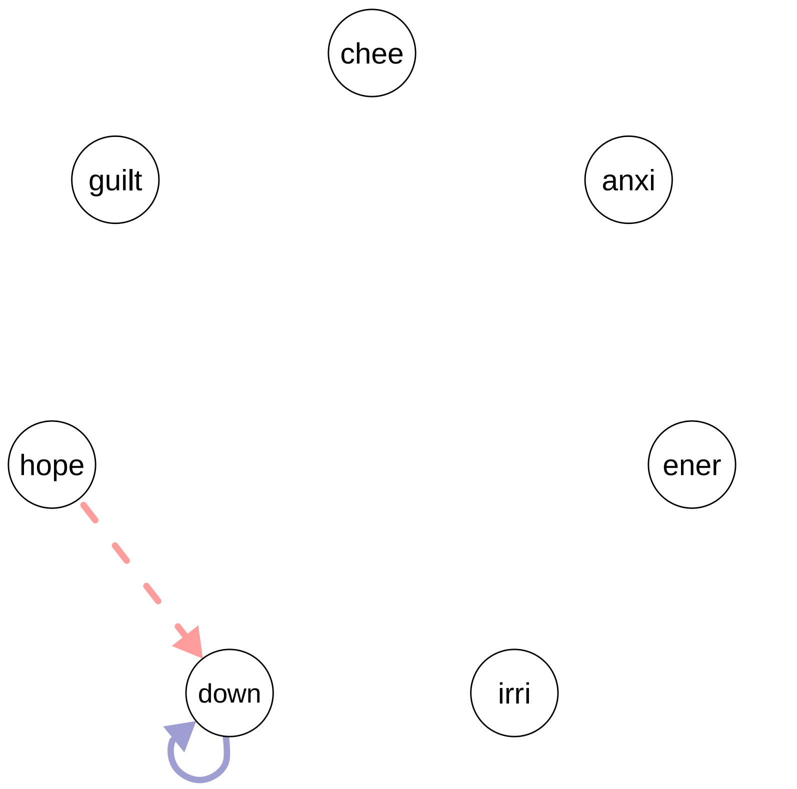

PCT tap ADM reg Pt 253 Estpoint 8

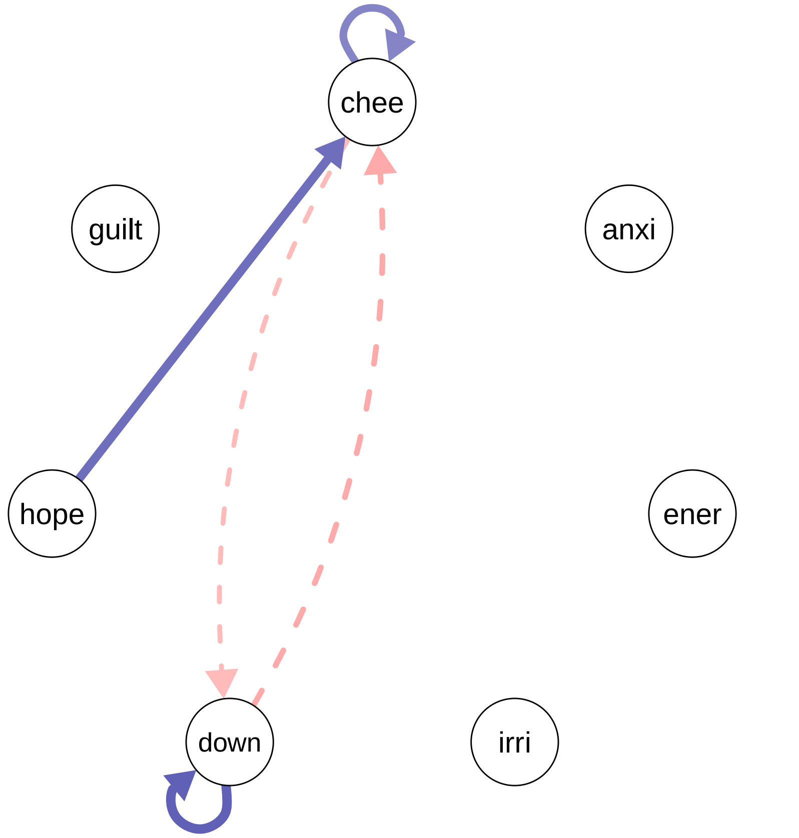

PCT tap ADM reg Pt 245 Estpoint 1

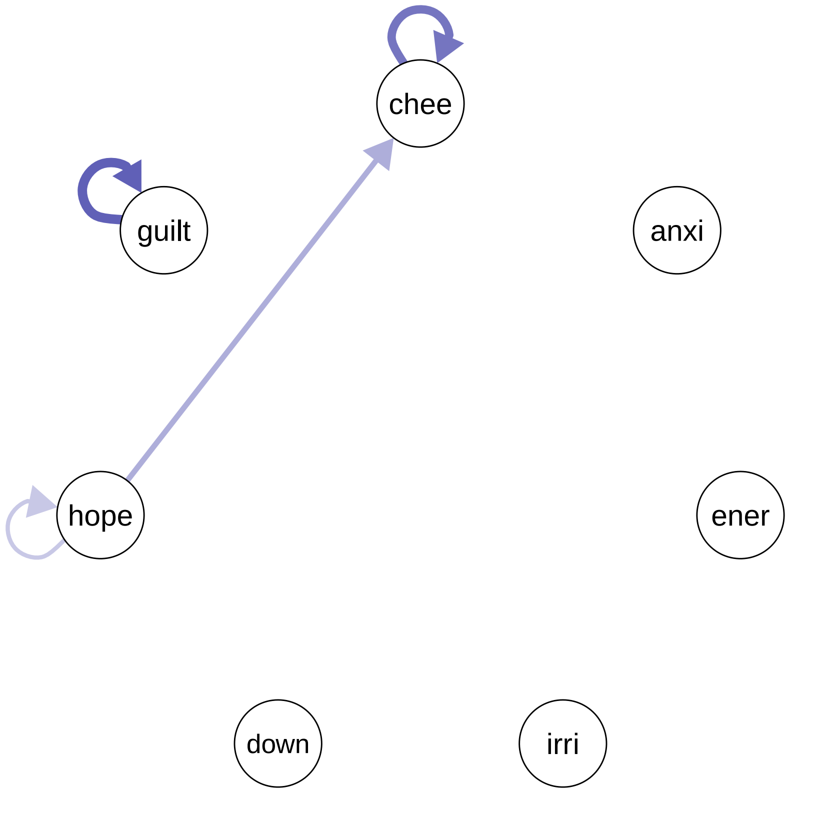

PCT tap ADM reg Pt 245 Estpoint 2

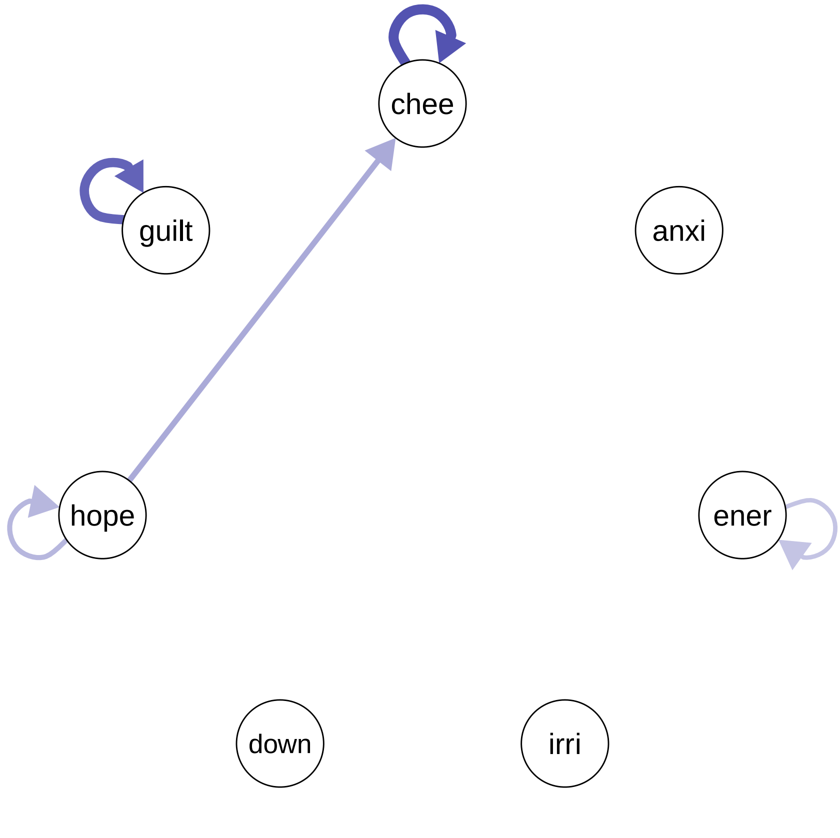

PCT tap ADM reg Pt 245 Estpoint 3

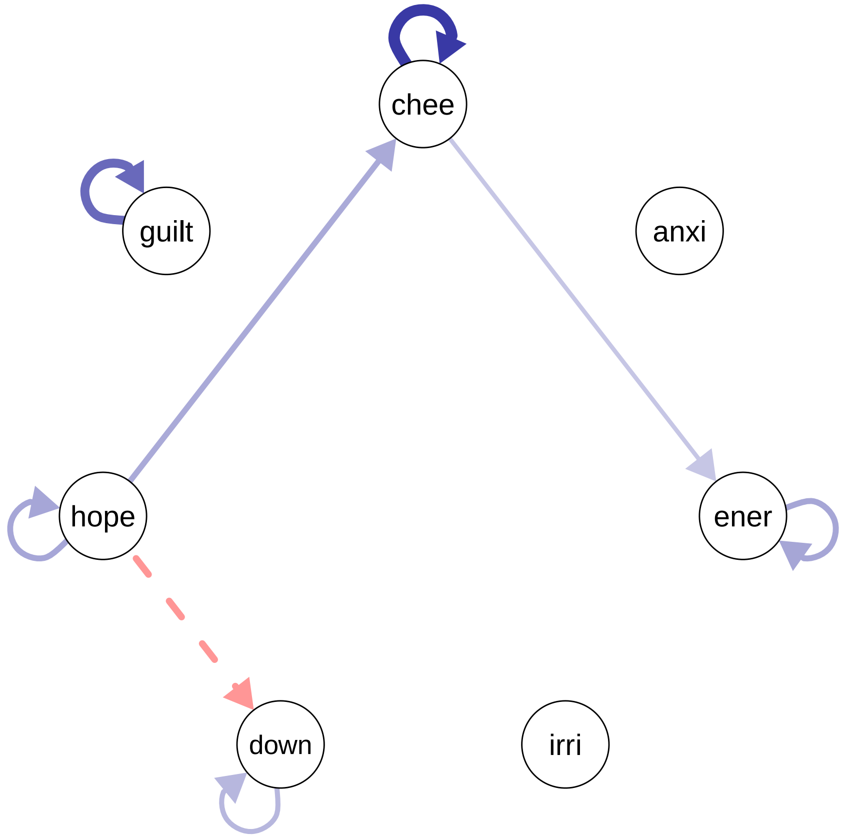

PCT tap ADM reg Pt 245 Estpoint 4

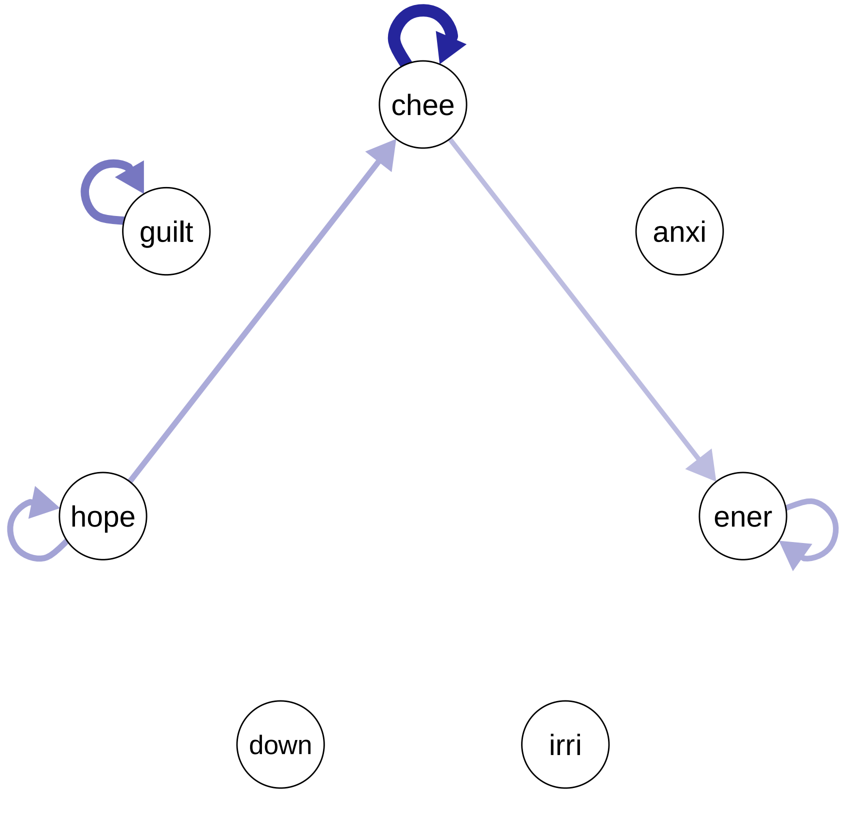

PCT tap ADM reg Pt 245 Estpoint 5

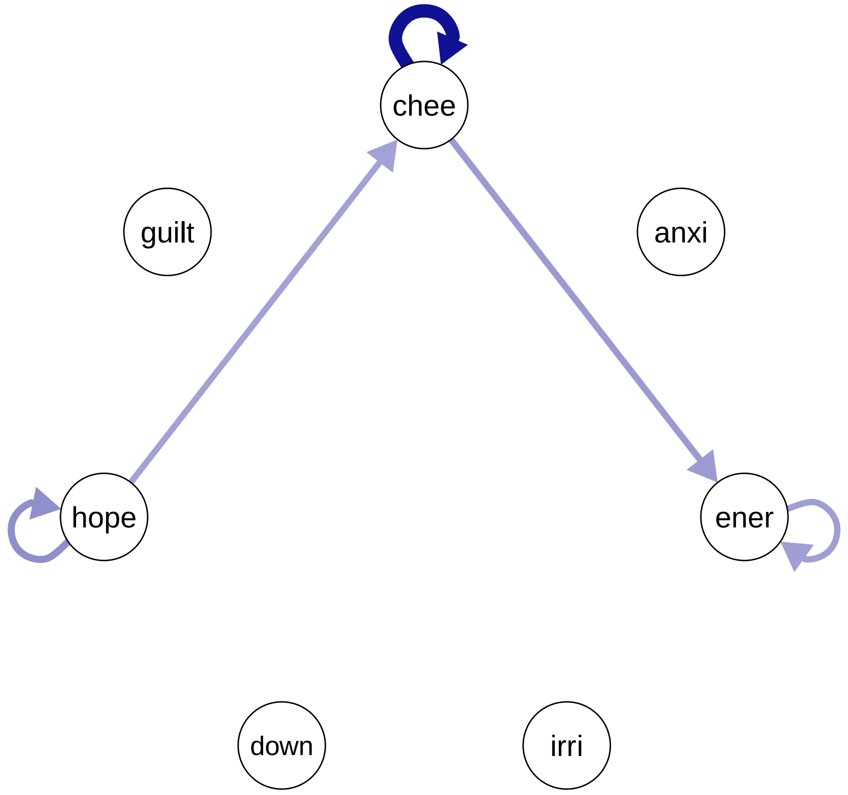

PCT tap ADM reg Pt 245 Estpoint 6

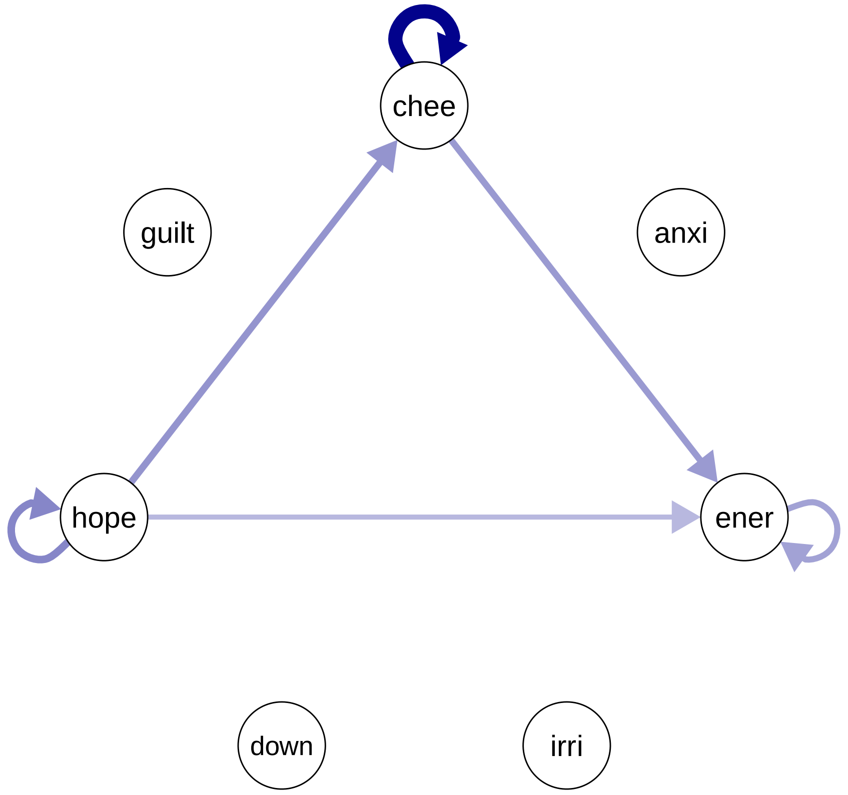

PCT tap ADM reg Pt 245 Estpoint 7

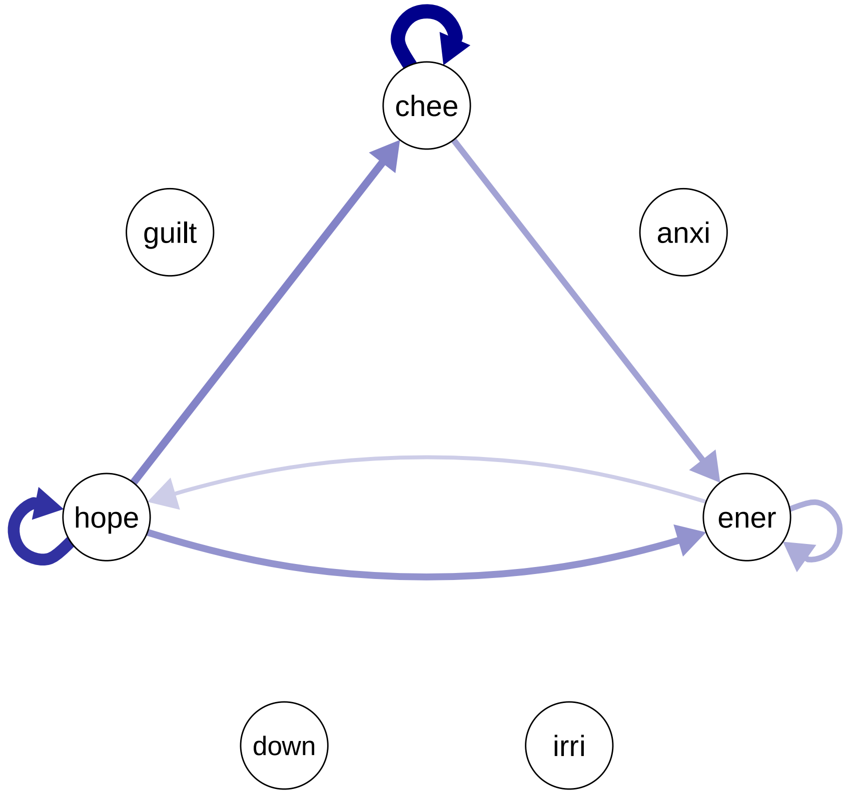

PCT tap ADM reg Pt 245 Estpoint 8

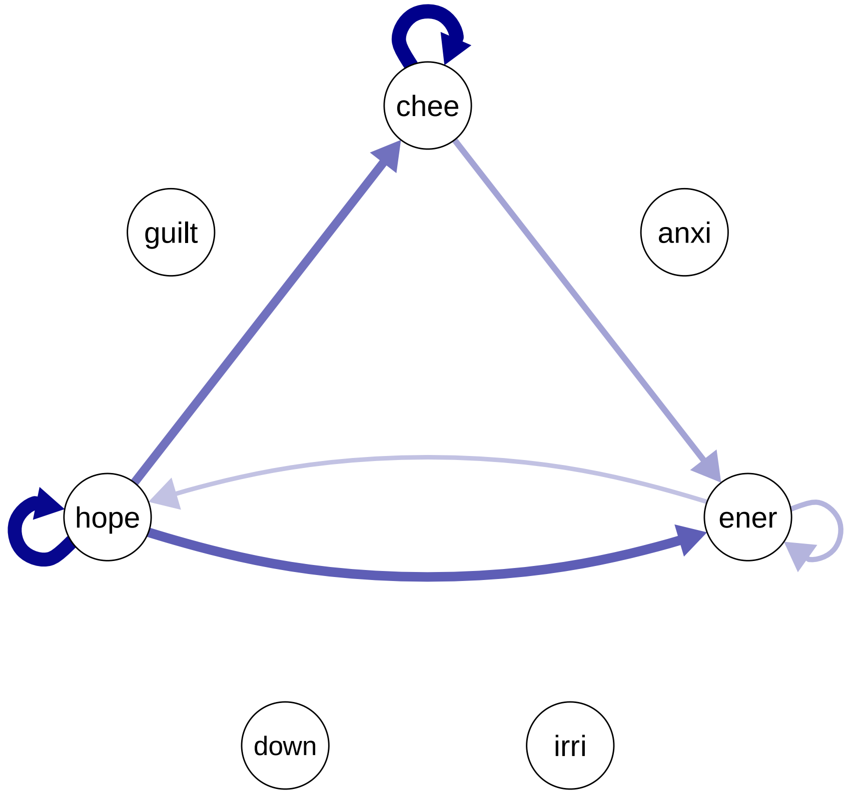

PCT tap ADM reg Pt 286 Estpoint 1

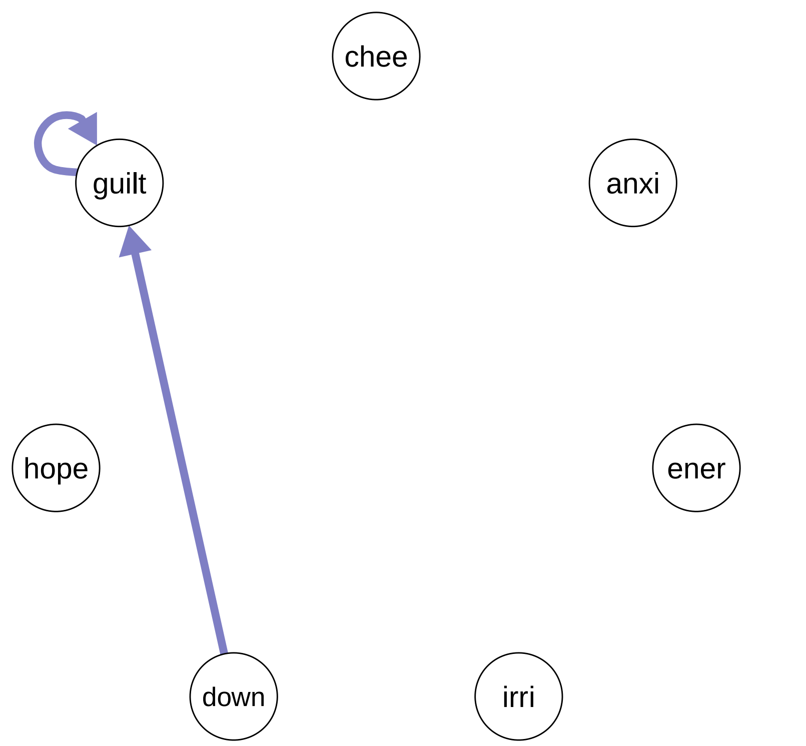

PCT tap ADM reg Pt 286 Estpoint 2

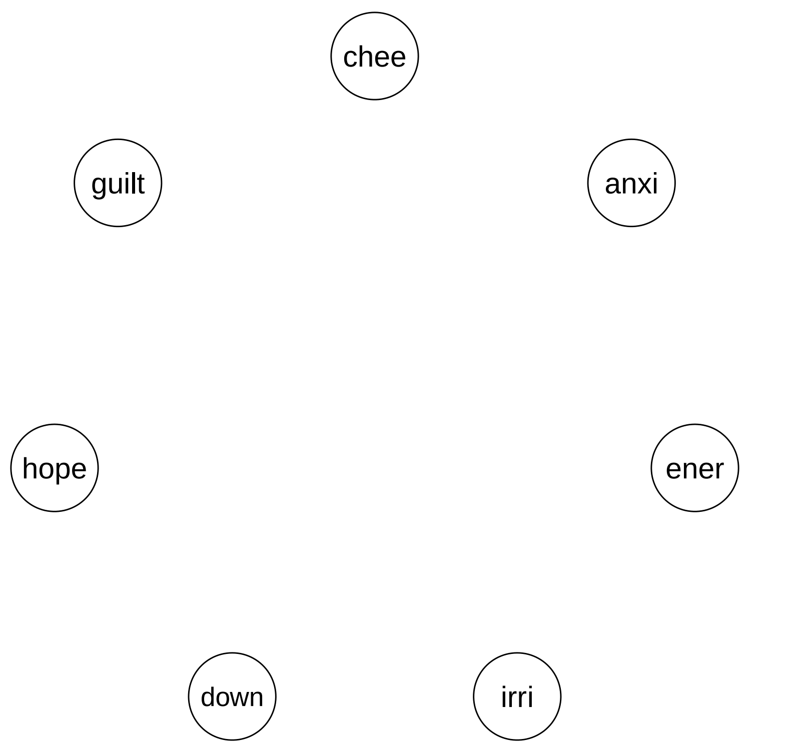

PCT tap ADM reg Pt 286 Estpoint 3

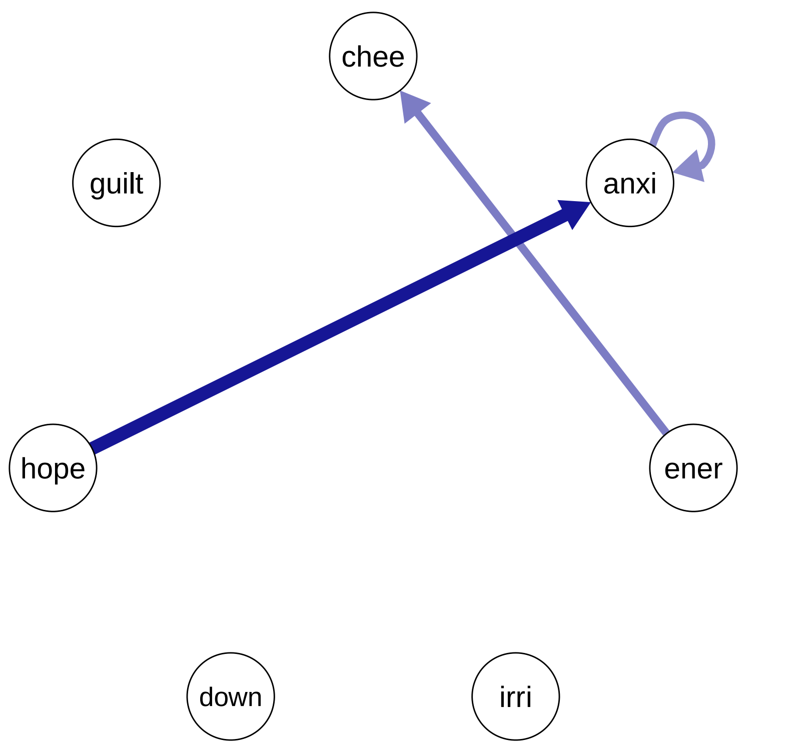

PCT tap ADM reg Pt 286 Estpoint 4

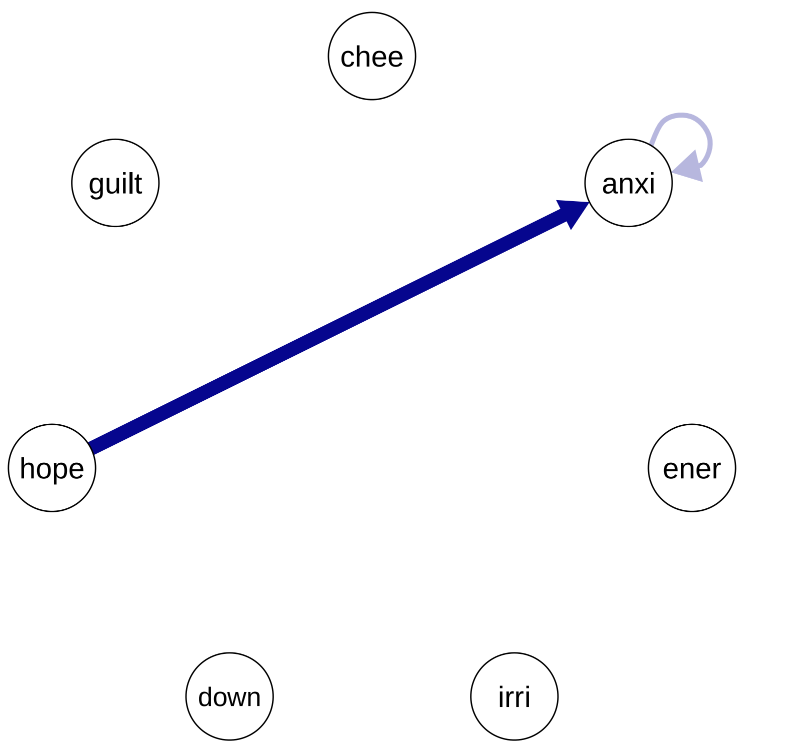

PCT tap ADM reg Pt 286 Estpoint 5

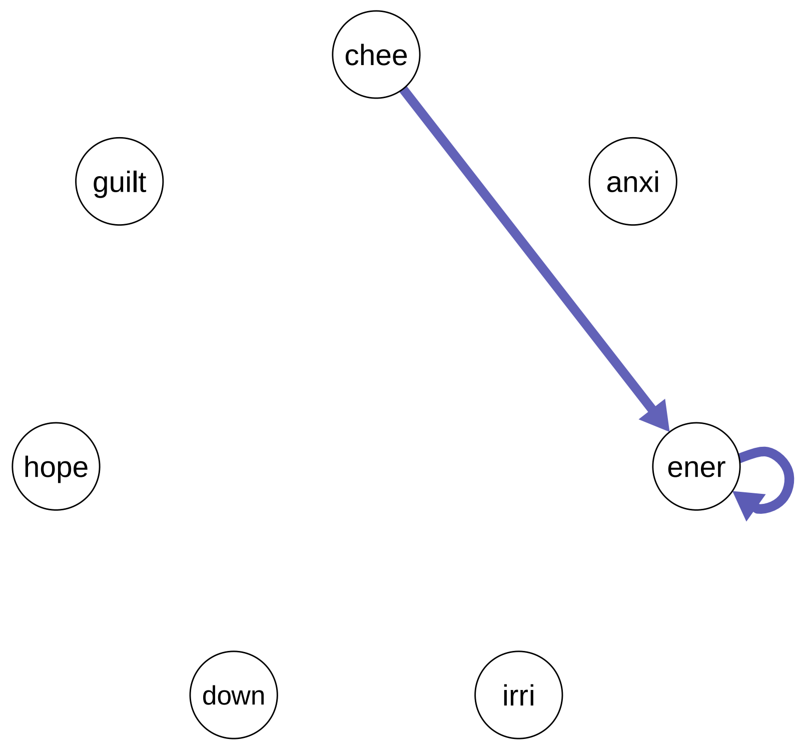

PCT tap ADM reg Pt 286 Estpoint 6

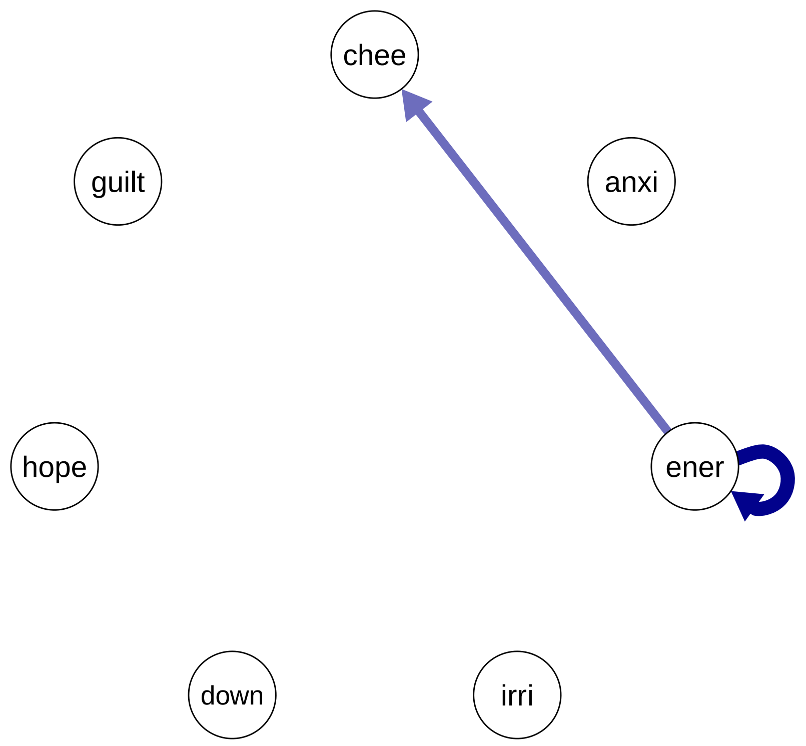

PCT tap ADM reg Pt 286 Estpoint 7

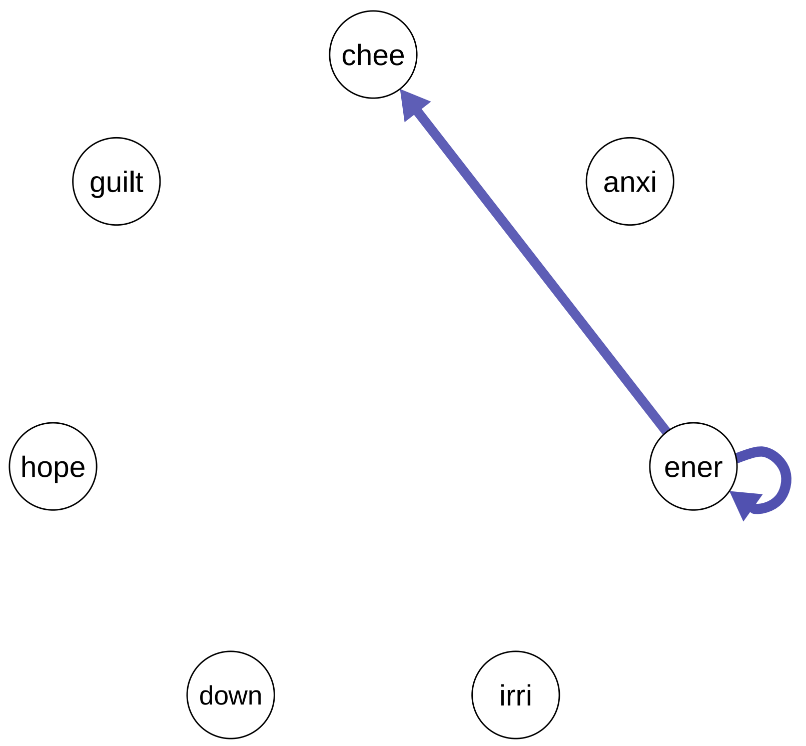

PCT tap ADM reg Pt 286 Estpoint 8

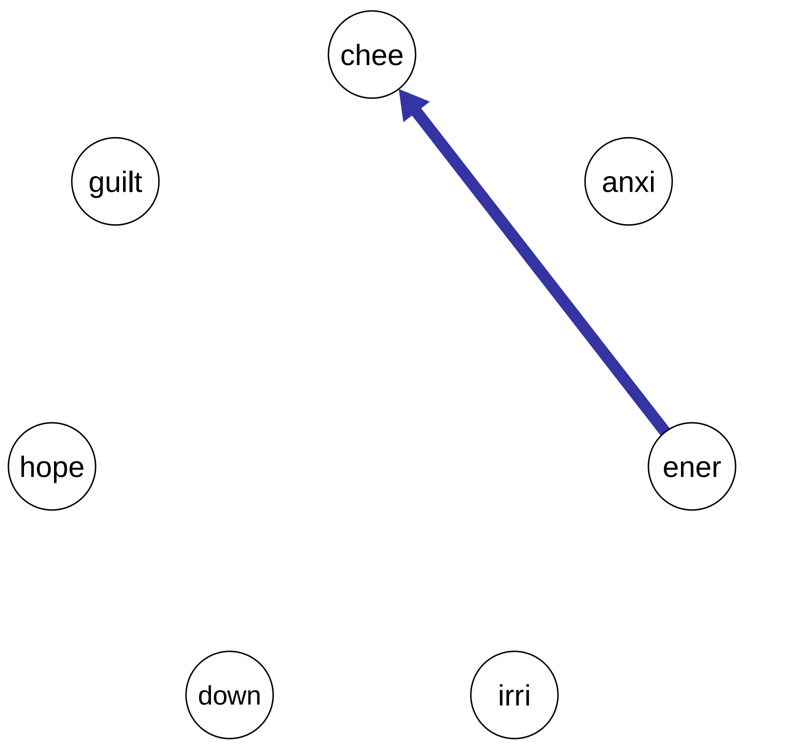

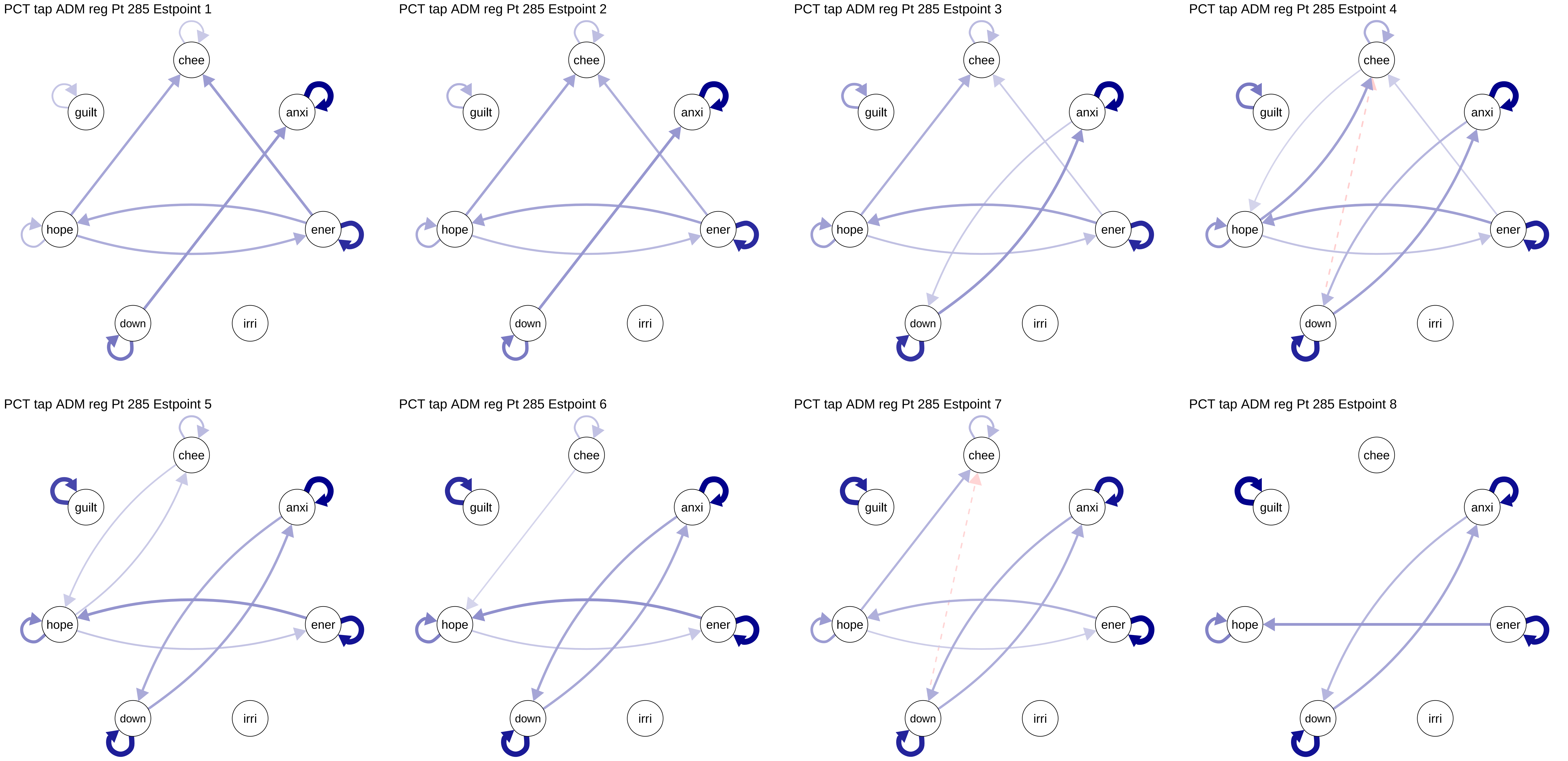
PCT tap ADM reg Pt 285 Estpoint 1
chee
guilt
anxi
hope
ener
down
irri
PCT tap ADM reg Pt 285 Estpoint 2
chee
guilt
anxi
hope
ener
down
irri
PCT tap ADM reg Pt 285 Estpoint 3
chee
guilt
anxi
hope
ener
down
irri
PCT tap ADM reg Pt 285 Estpoint 4
chee
guilt
anxi
hope
ener
down
irri
PCT tap ADM reg Pt 285 Estpoint 5
chee
guilt
anxi
hope
ener
down
irri
PCT tap ADM reg Pt 285 Estpoint 6
chee
guilt
anxi
hope
ener
down
irri
PCT tap ADM reg Pt 285 Estpoint 7
chee
guilt
anxi
hope
ener
down
irri
PCT tap ADM reg Pt 285 Estpoint 8
chee
guilt
anxi
hope
ener
down
irri

PCT tap ADM reg Pt 263 Estpoint 1

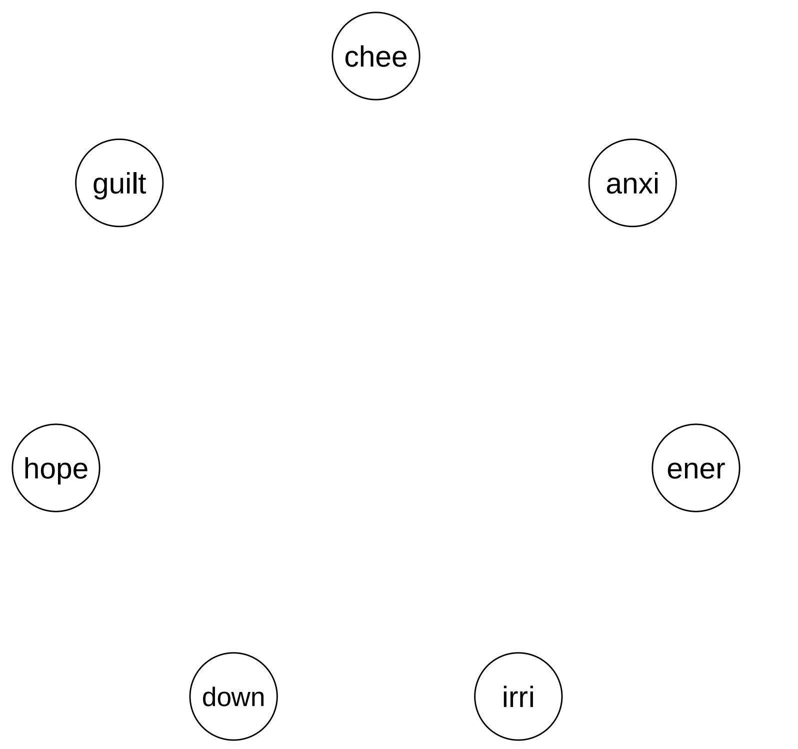

PCT tap ADM reg Pt 263 Estpoint 2

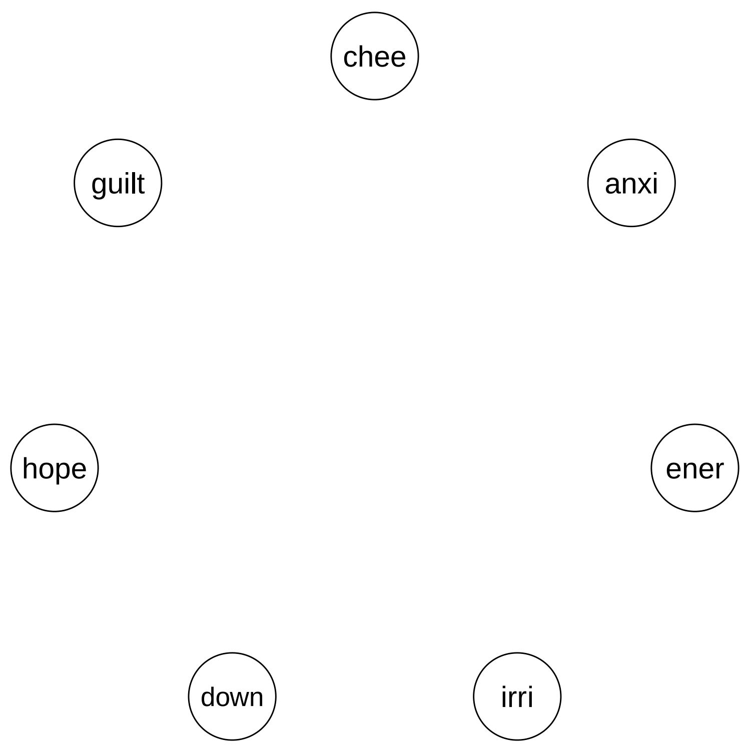

PCT tap ADM reg Pt 263 Estpoint 3

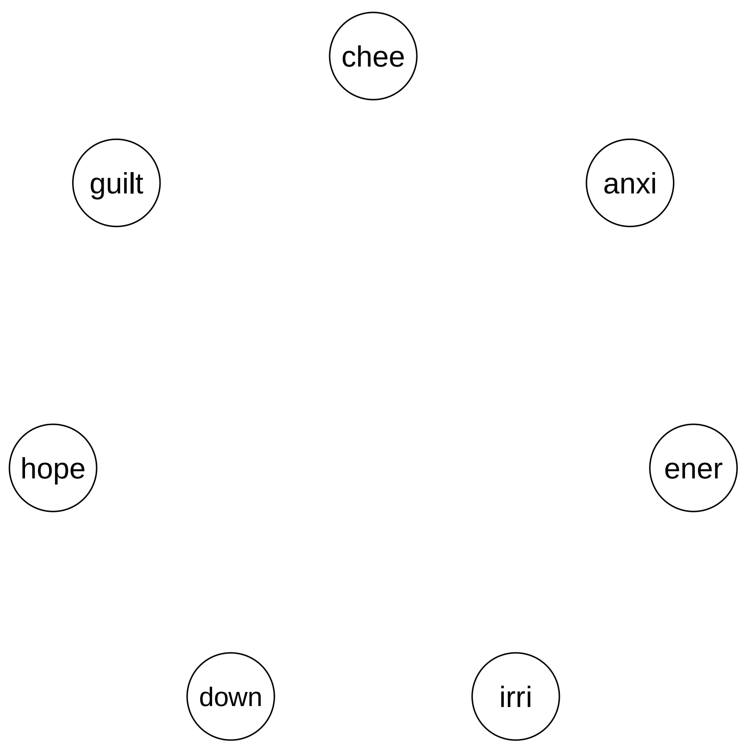

PCT tap ADM reg Pt 263 Estpoint 4

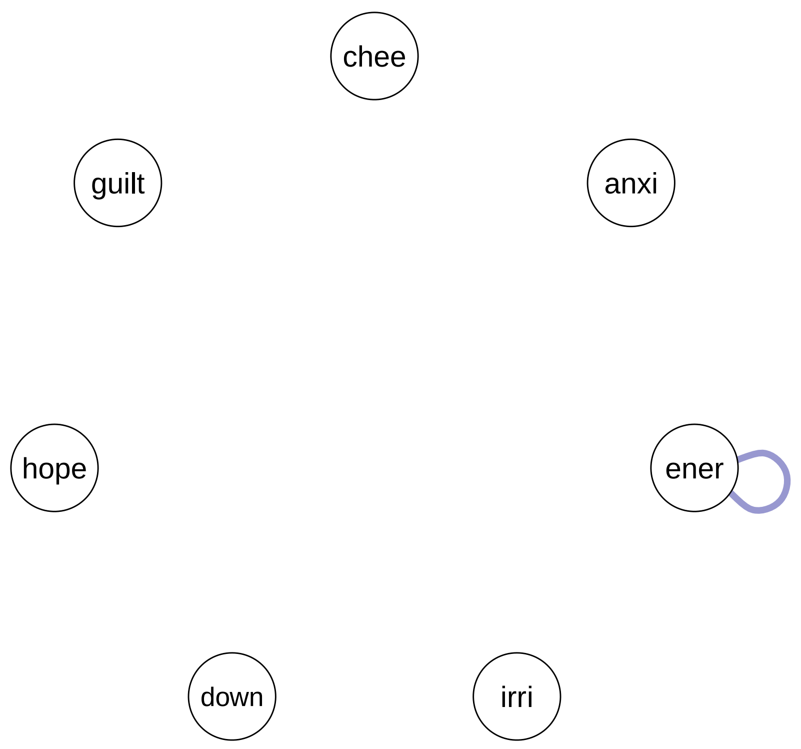

PCT tap ADM reg Pt 263 Estpoint 5

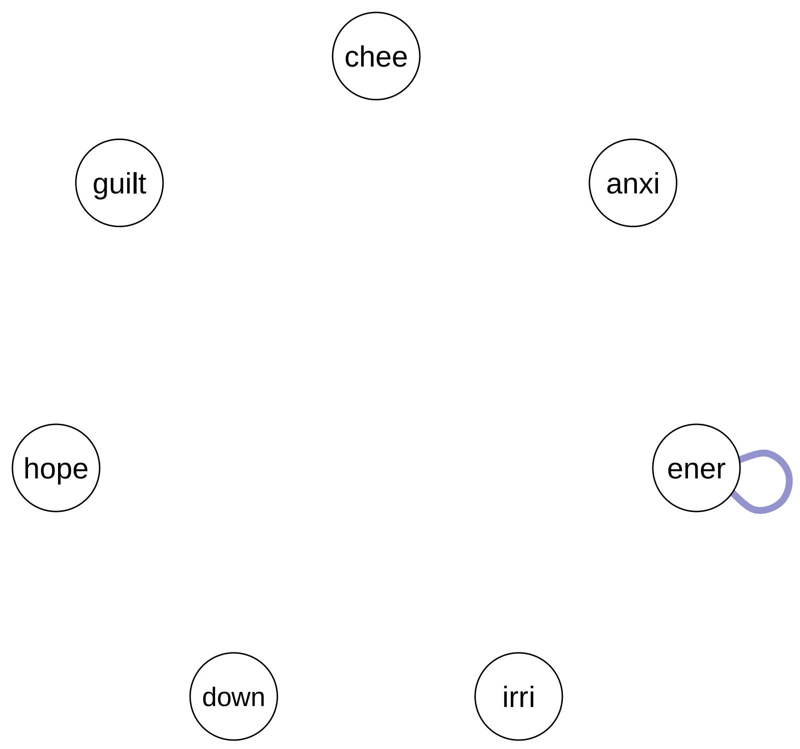

PCT tap ADM reg Pt 263 Estpoint 6

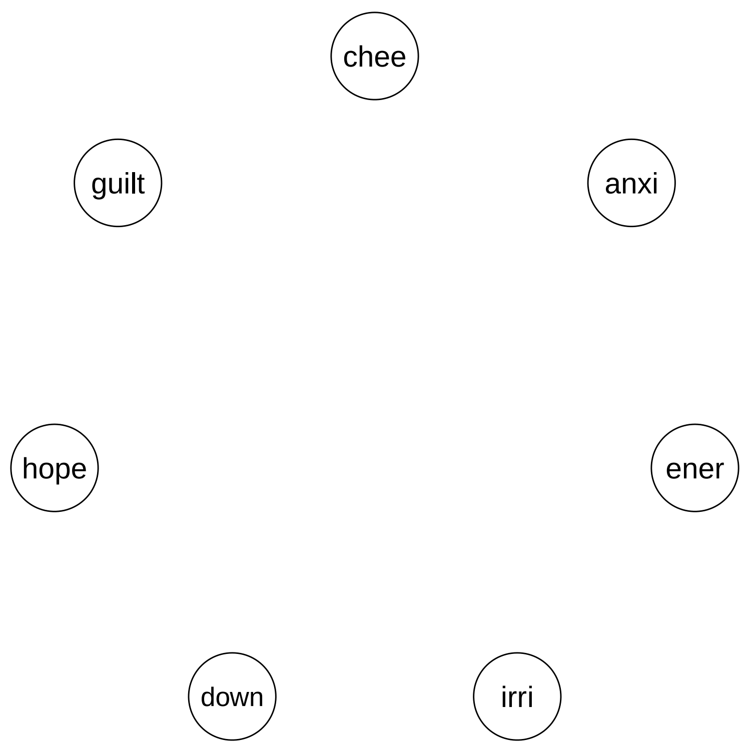

PCT tap ADM reg Pt 263 Estpoint 7

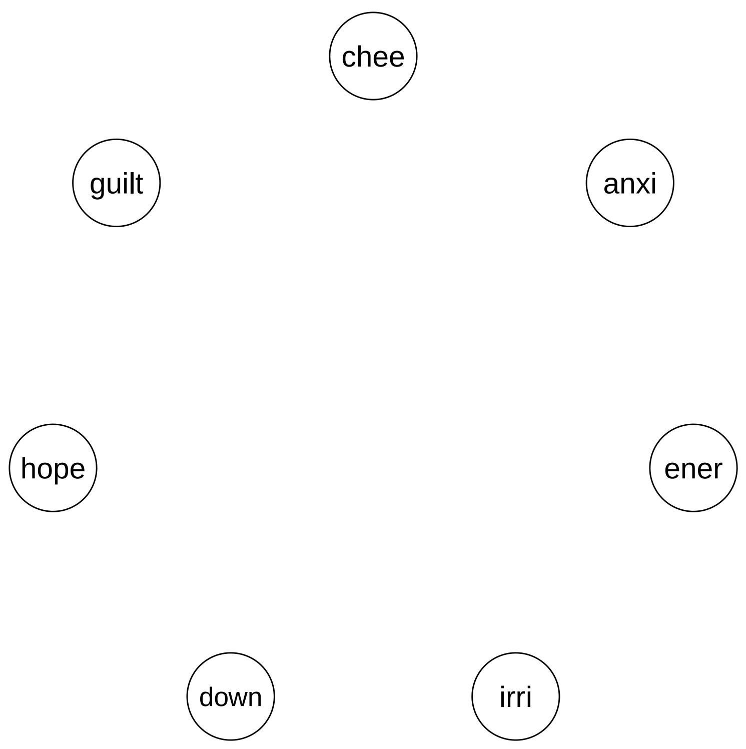

PCT tap ADM reg Pt 263 Estpoint 8

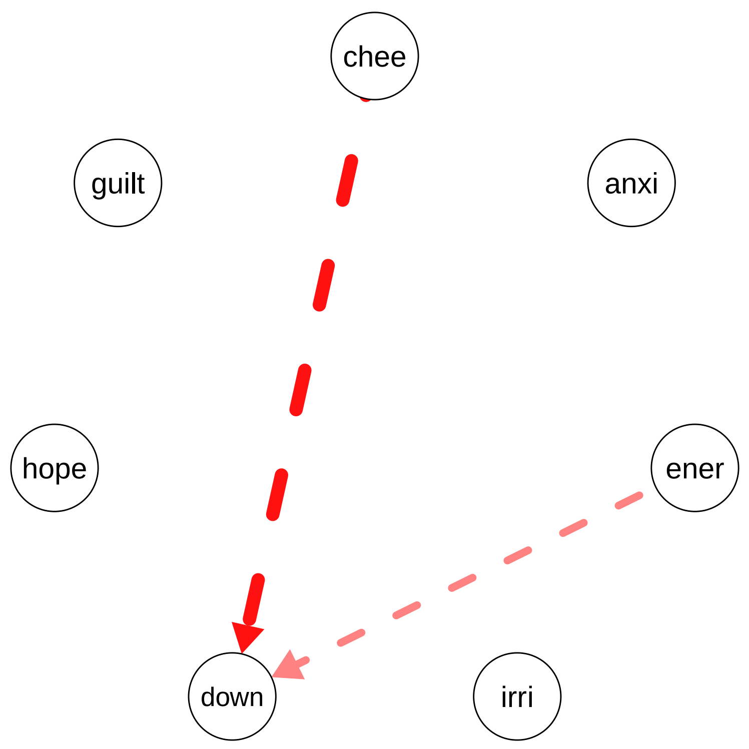

PCT tap ADM reg Pt 284 Estpoint 1

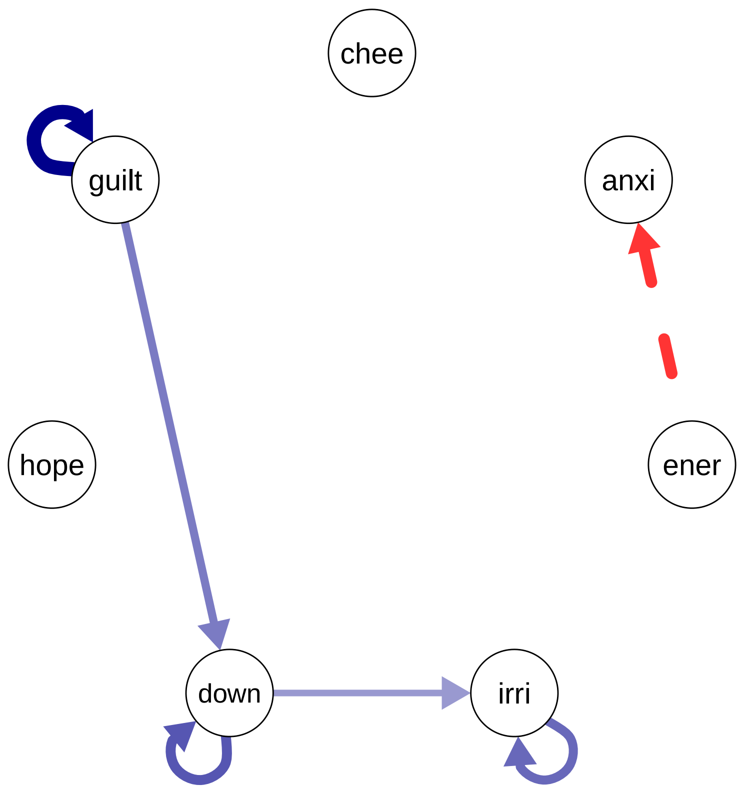

PCT tap ADM reg Pt 284 Estpoint 2

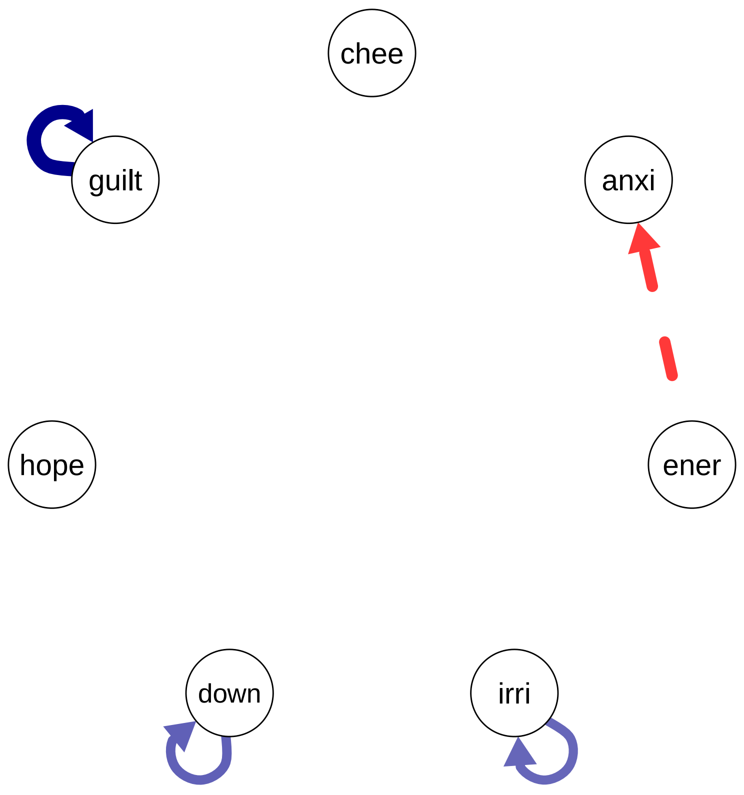

PCT tap ADM reg Pt 284 Estpoint 3

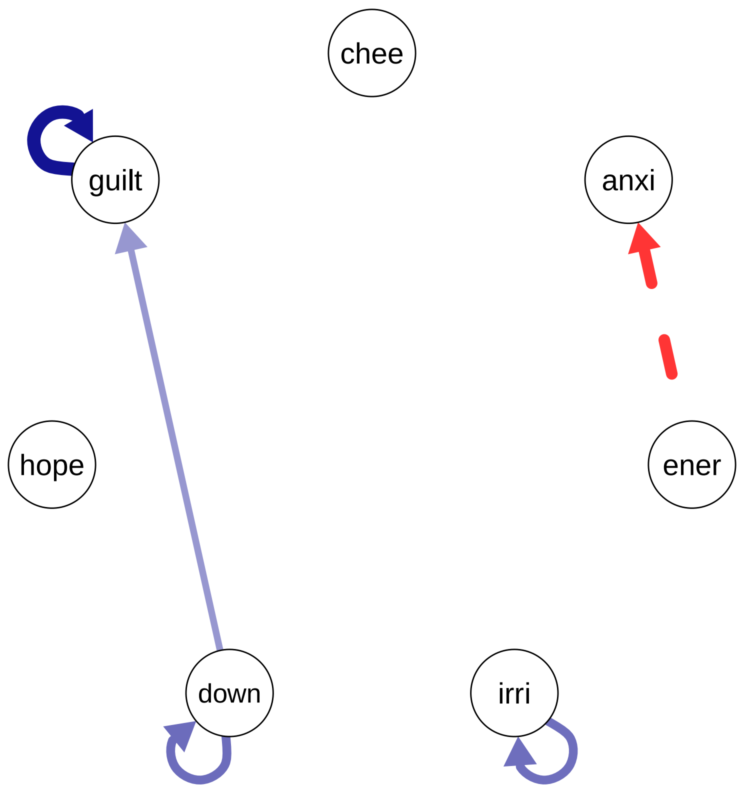

PCT tap ADM reg Pt 284 Estpoint 4

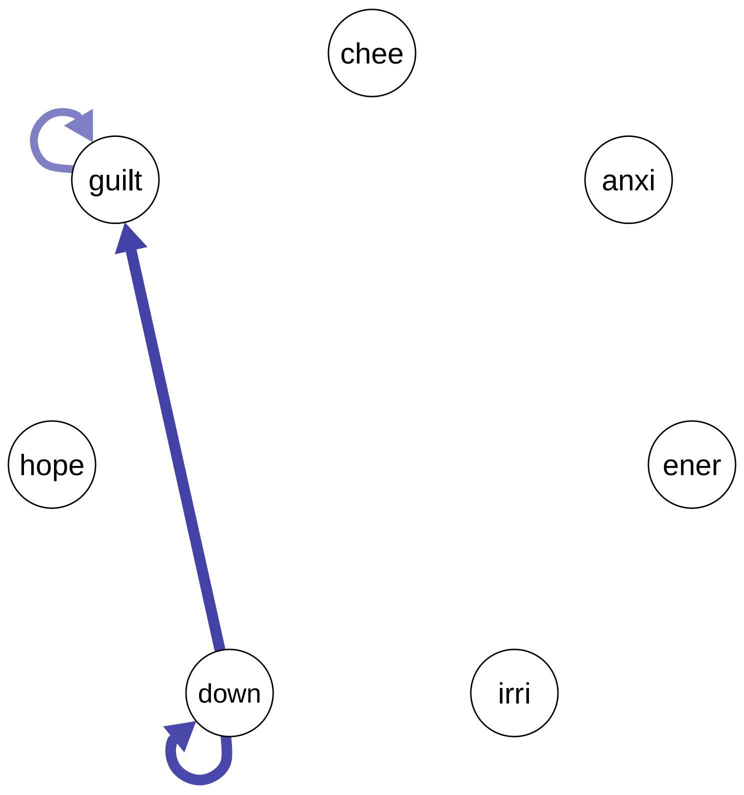

PCT tap ADM reg Pt 284 Estpoint 5

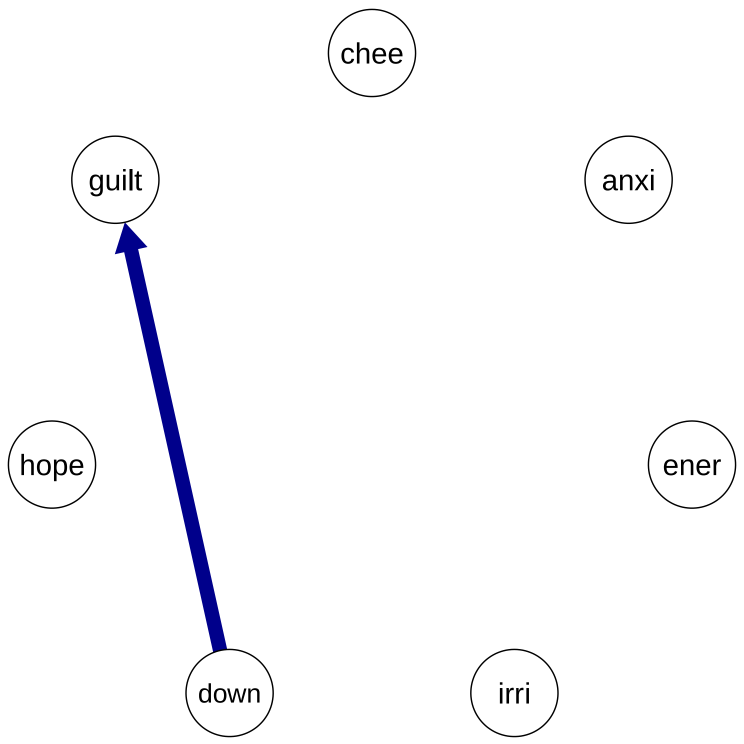

PCT tap ADM reg Pt 284 Estpoint 6

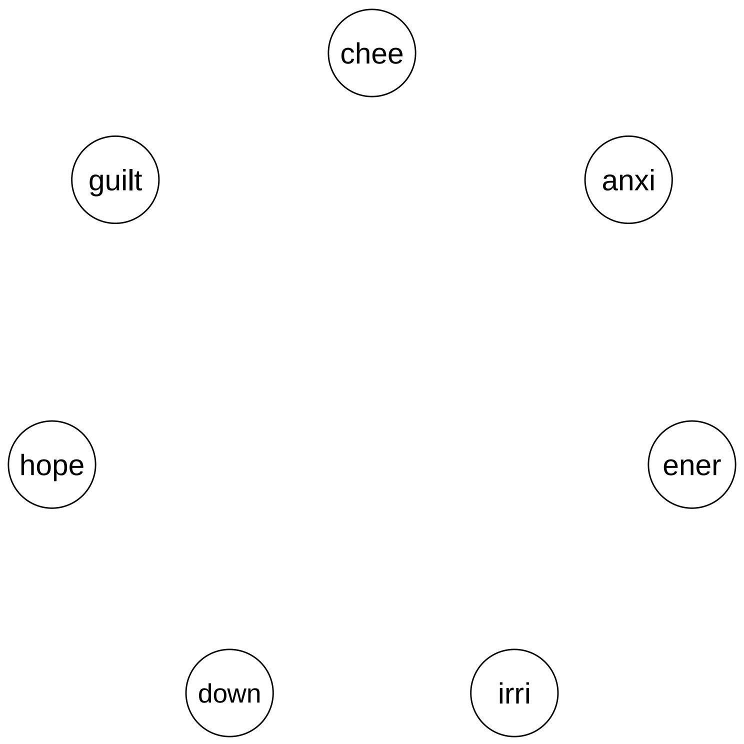

PCT tap ADM reg Pt 284 Estpoint 7

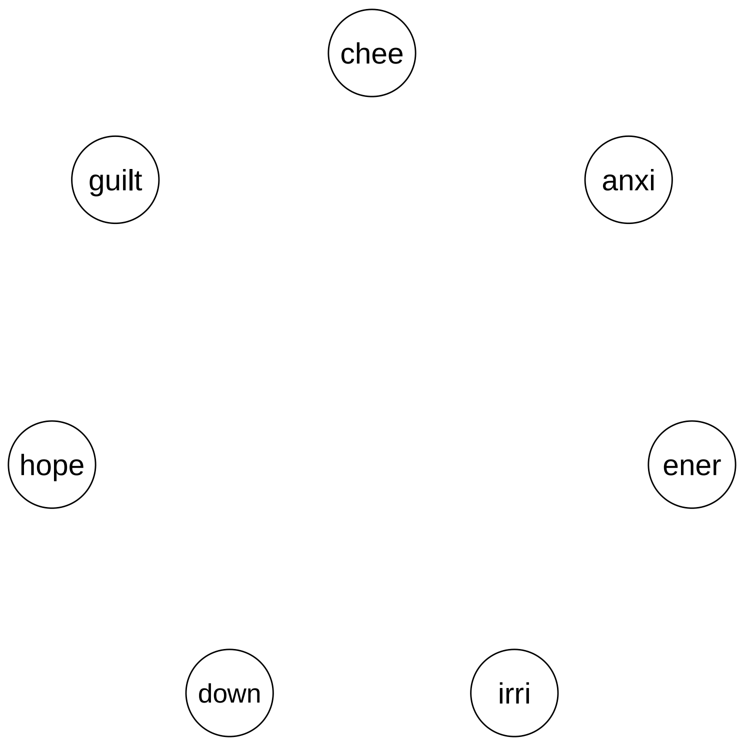

PCT tap ADM reg Pt 284 Estpoint 8

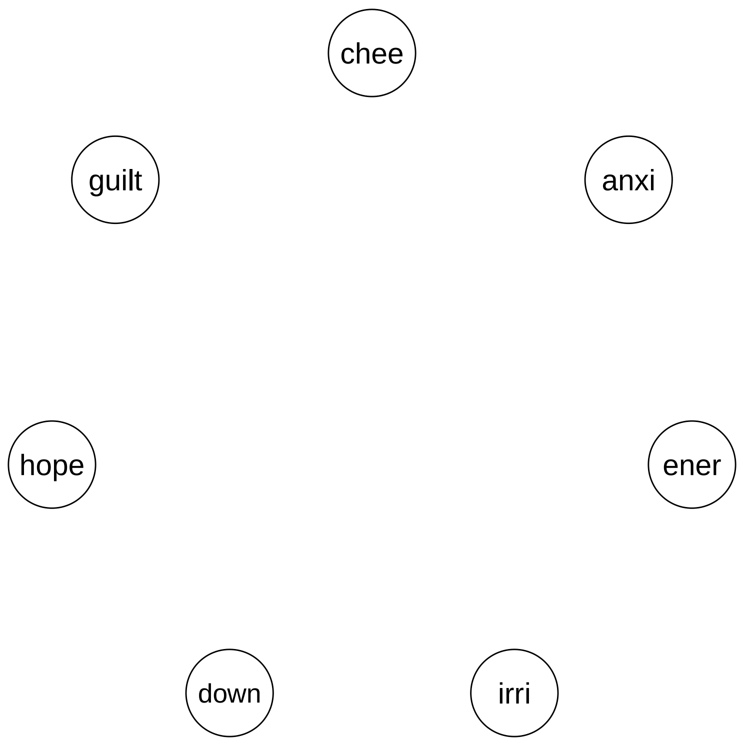

PCT tap ADM reg Pt 68 Estpoint 1

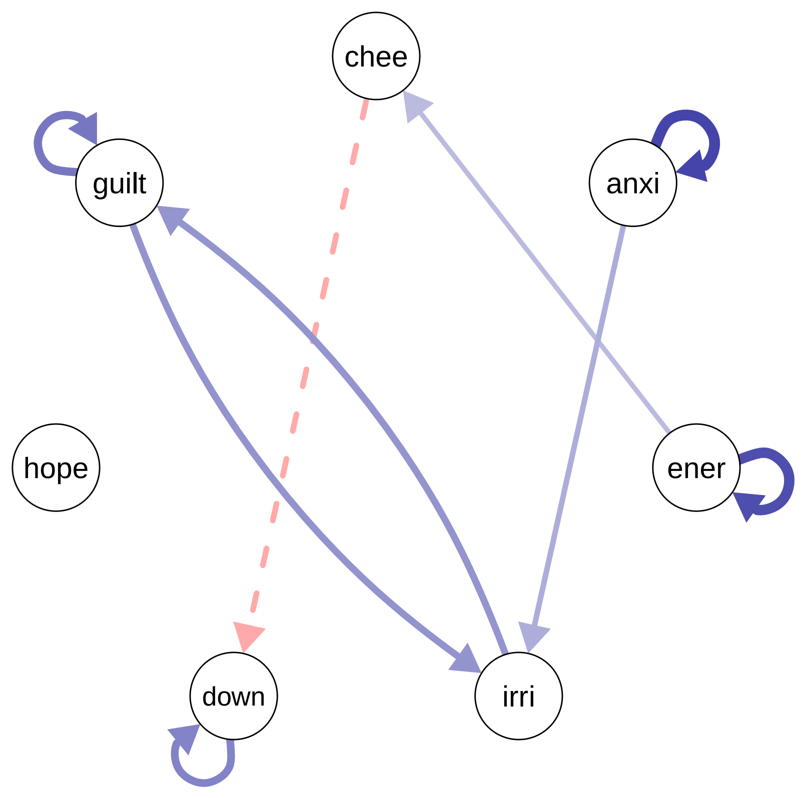

PCT tap ADM reg Pt 68 Estpoint 2

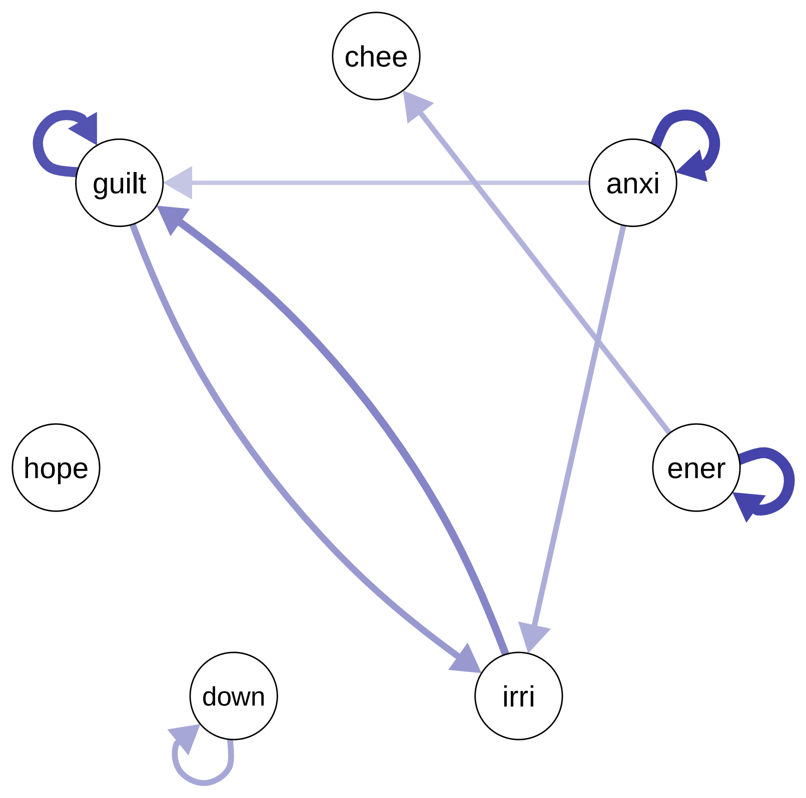

PCT tap ADM reg Pt 68 Estpoint 3

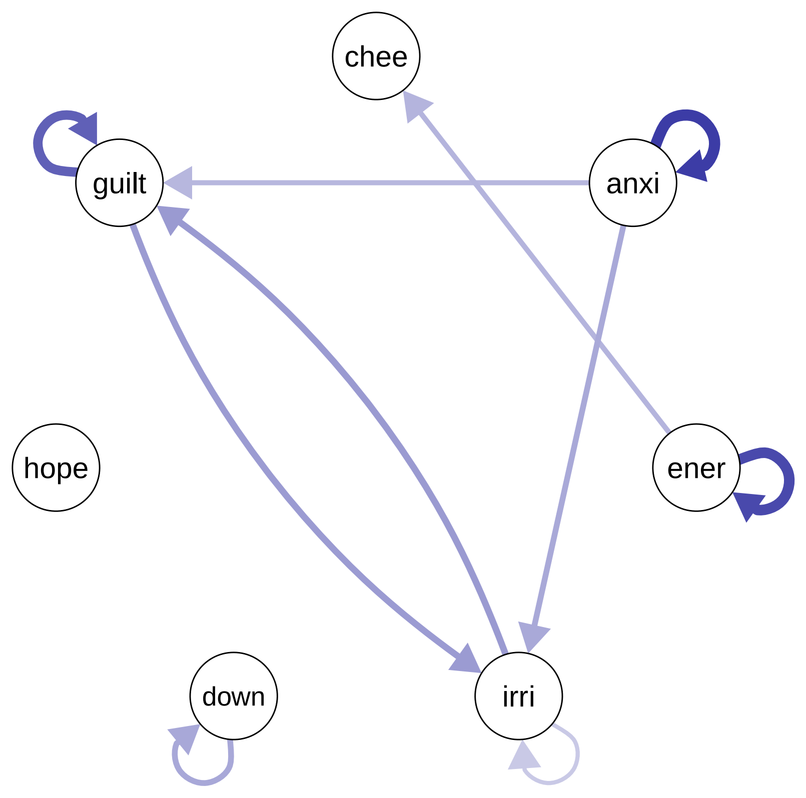

PCT tap ADM reg Pt 68 Estpoint 4

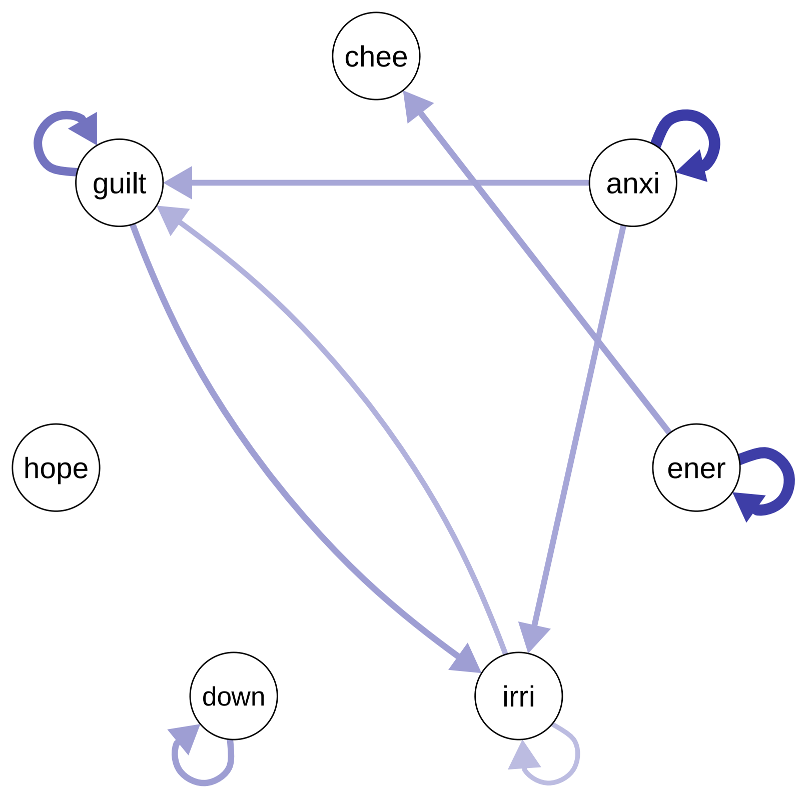

PCT tap ADM reg Pt 68 Estpoint 5

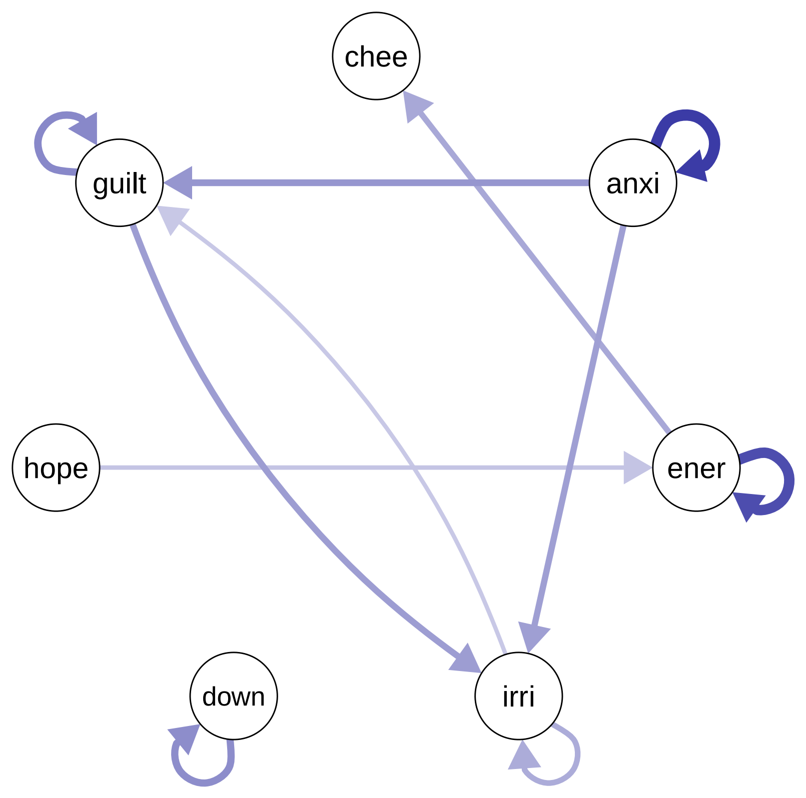

PCT tap ADM reg Pt 68 Estpoint 6

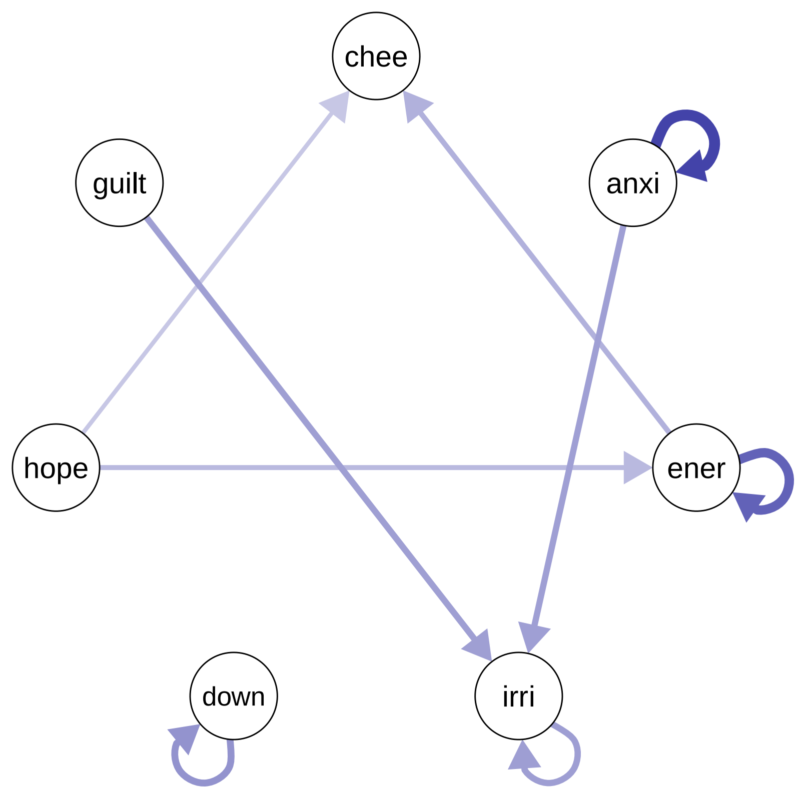

PCT tap ADM reg Pt 68 Estpoint 7

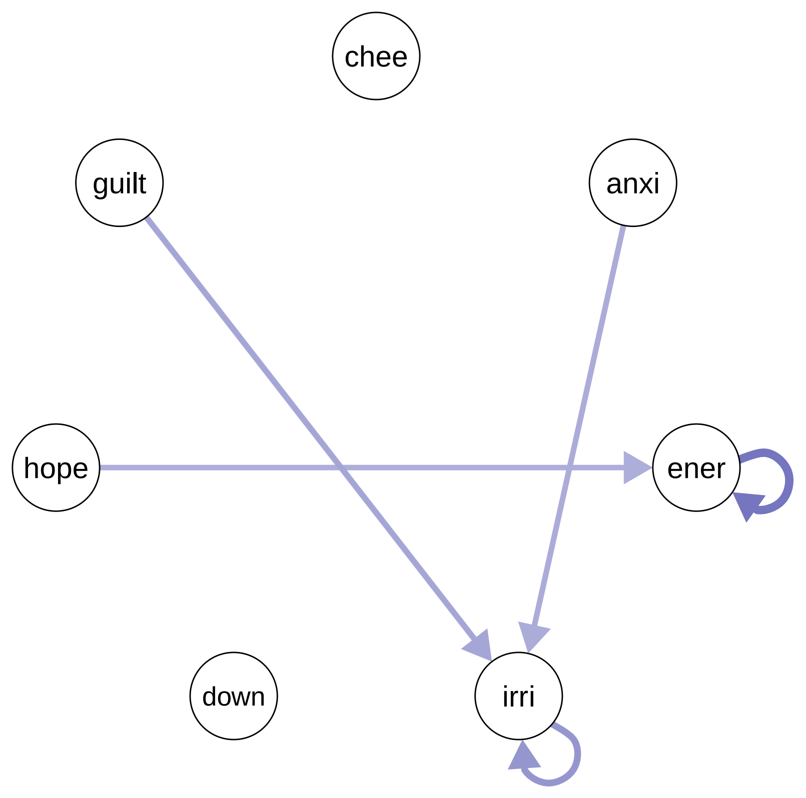

PCT tap ADM reg Pt 68 Estpoint 8

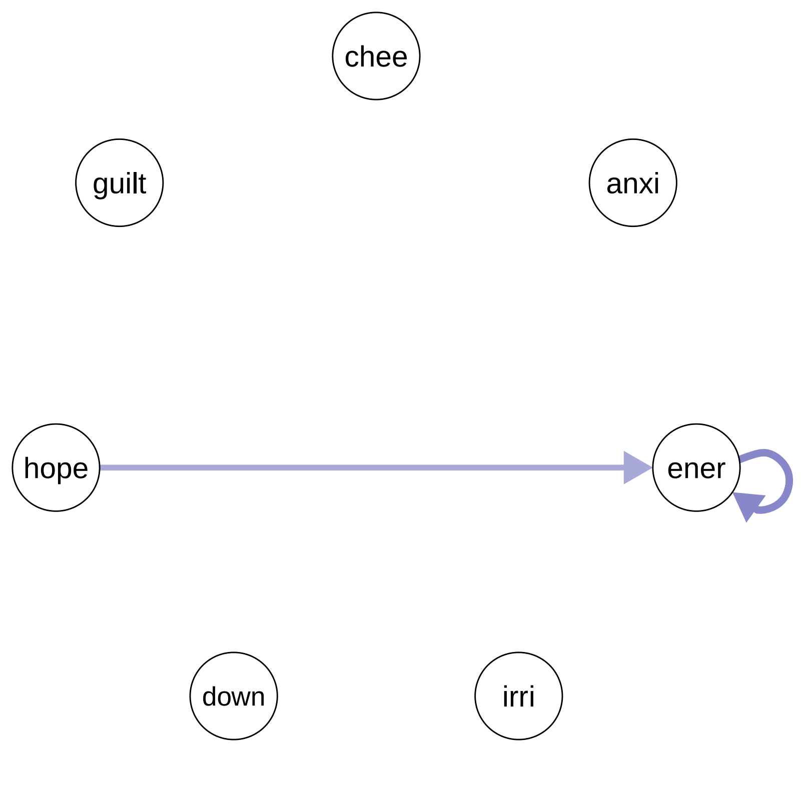

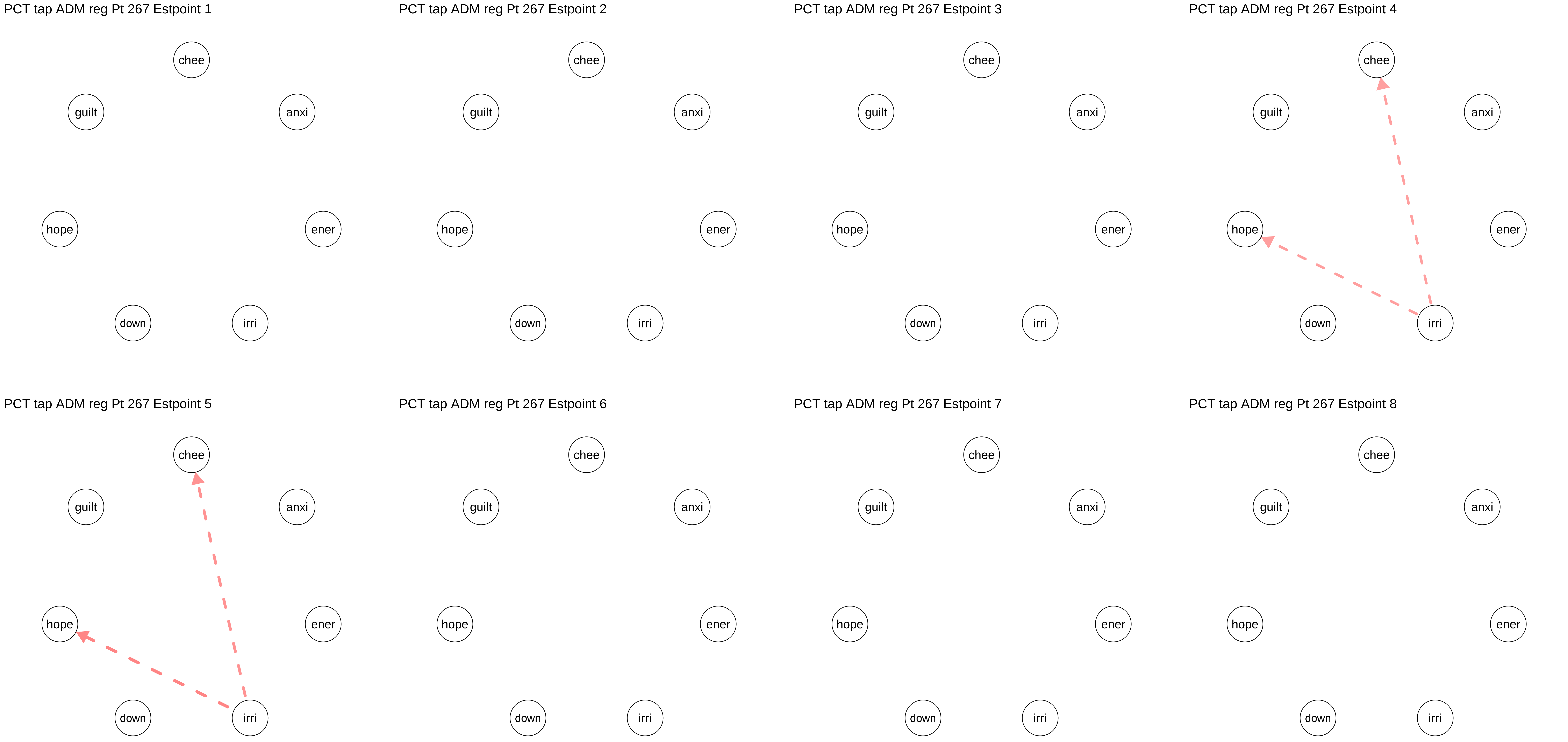
PCT tap ADM reg Pt 267 Estpoint 1
chee
guilt
anxi
hope
ener
down
irri
PCT tap ADM reg Pt 267 Estpoint 2
chee
guilt
anxi
hope
ener
down
irri
PCT tap ADM reg Pt 267 Estpoint 3
chee
guilt
anxi
hope
ener
down
irri
PCT tap ADM reg Pt 267 Estpoint 4
chee
guilt
anxi
hope
ener
down
irri
PCT tap ADM reg Pt 267 Estpoint 5
chee
guilt
anxi
hope
ener
down
irri
PCT tap ADM reg Pt 267 Estpoint 6
chee
guilt
anxi
hope
ener
down
irri
PCT tap ADM reg Pt 267 Estpoint 7
chee
guilt
anxi
hope
ener
down
irri
PCT tap ADM reg Pt 267 Estpoint 8
chee
guilt
anxi
hope
ener
down
irri

PCT tap ADM reg Pt 246 Estpoint 1

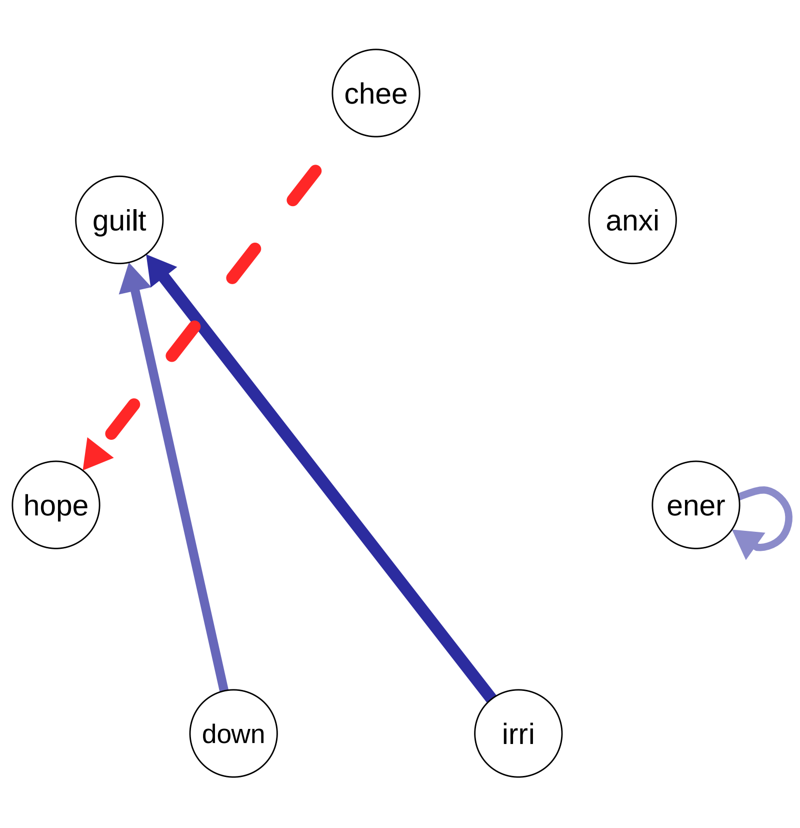

PCT tap ADM reg Pt 246 Estpoint 2

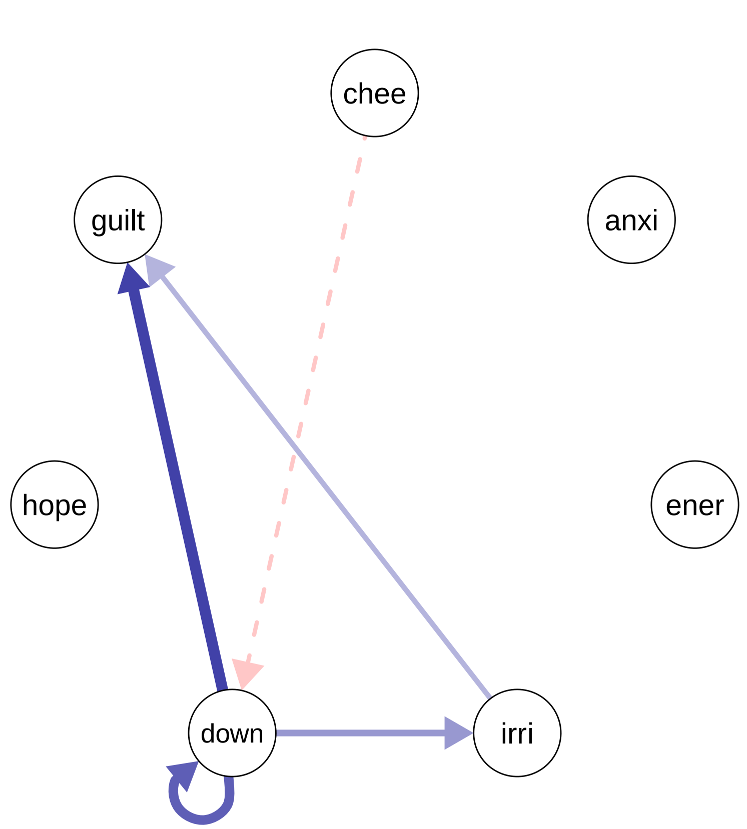

PCT tap ADM reg Pt 246 Estpoint 3

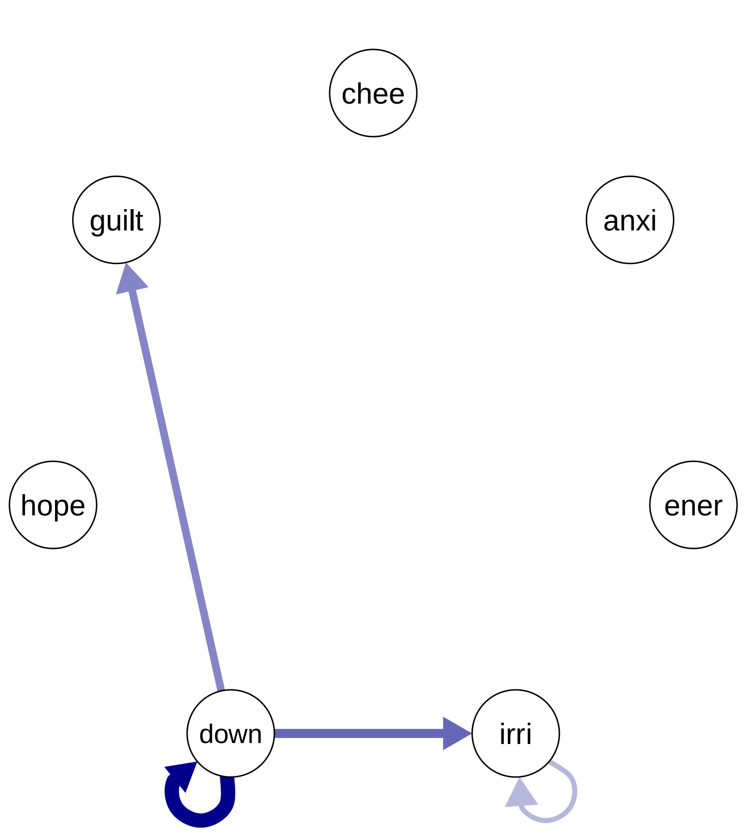

PCT tap ADM reg Pt 246 Estpoint 4

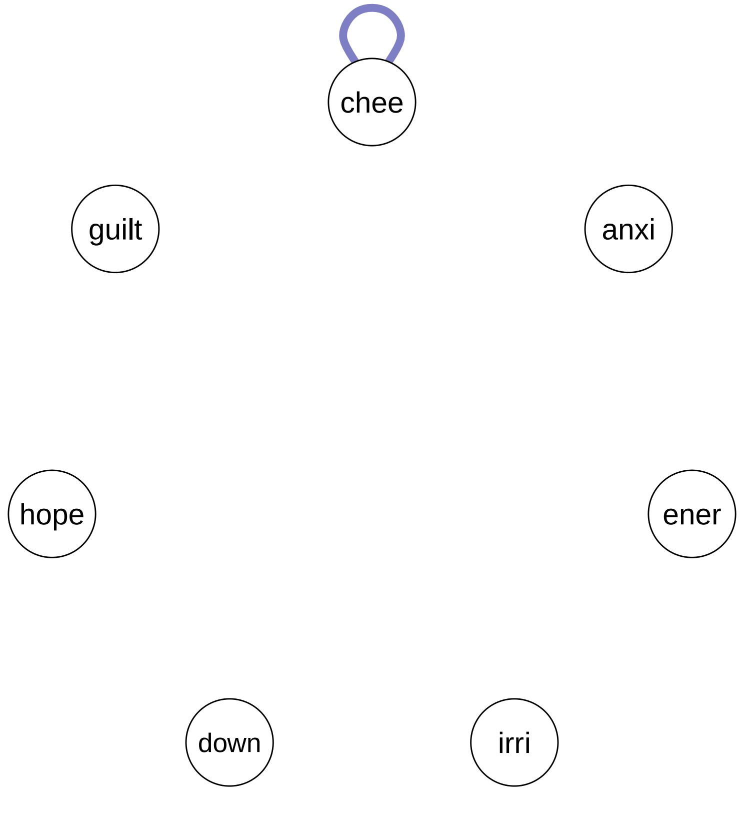

PCT tap ADM reg Pt 246 Estpoint 5

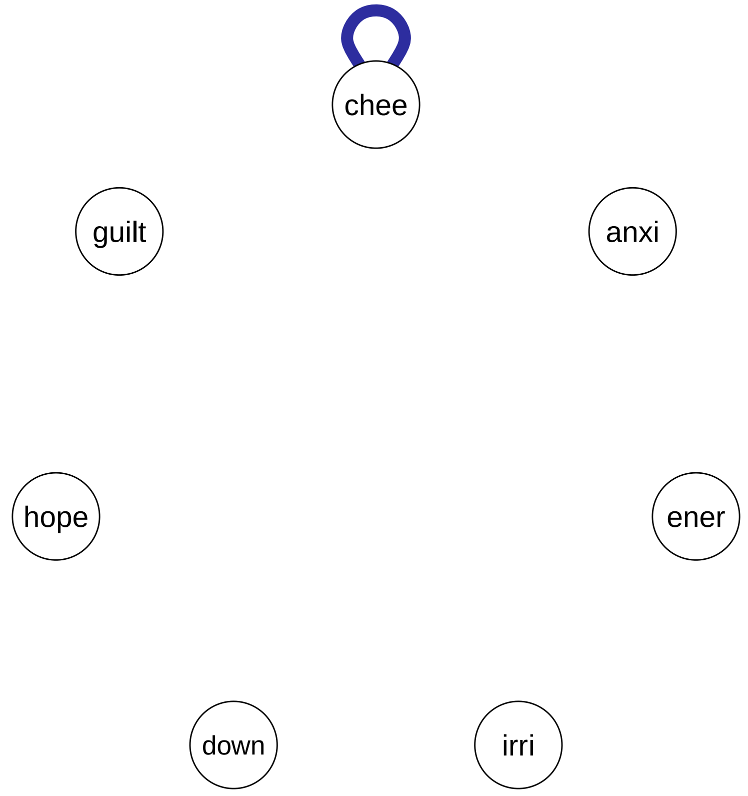

PCT tap ADM reg Pt 246 Estpoint 6

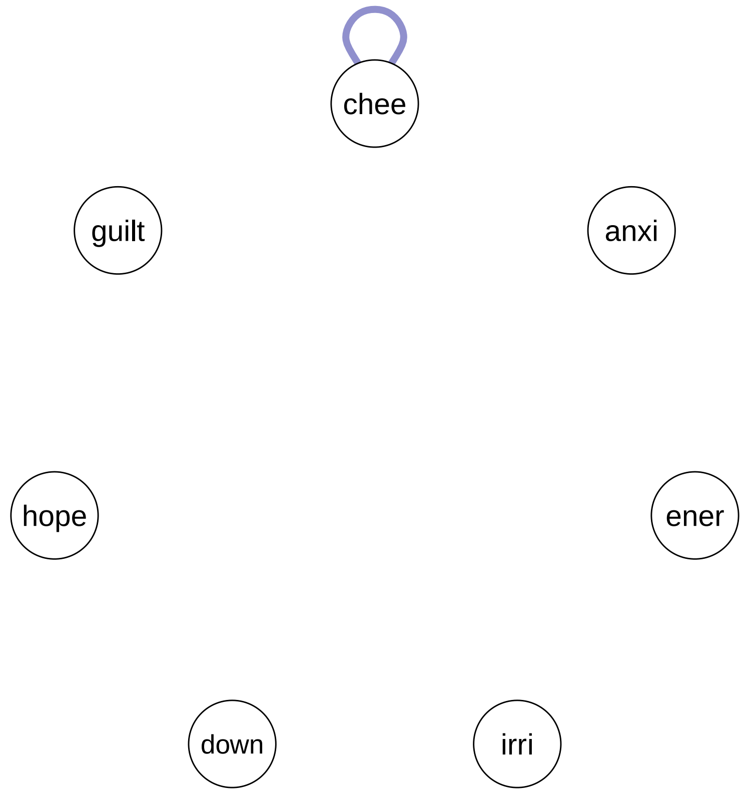

PCT tap ADM reg Pt 246 Estpoint 7

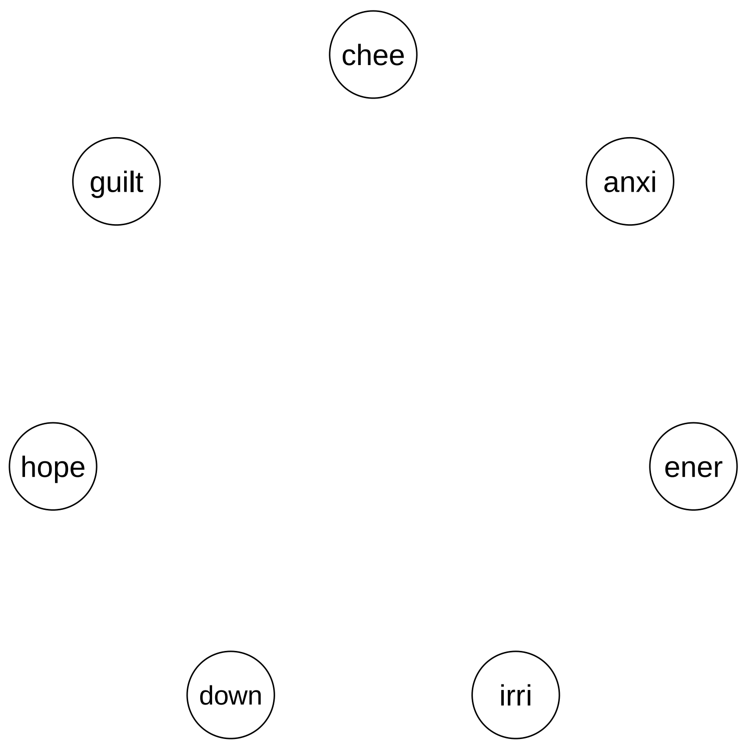

PCT tap ADM reg Pt 246 Estpoint 8

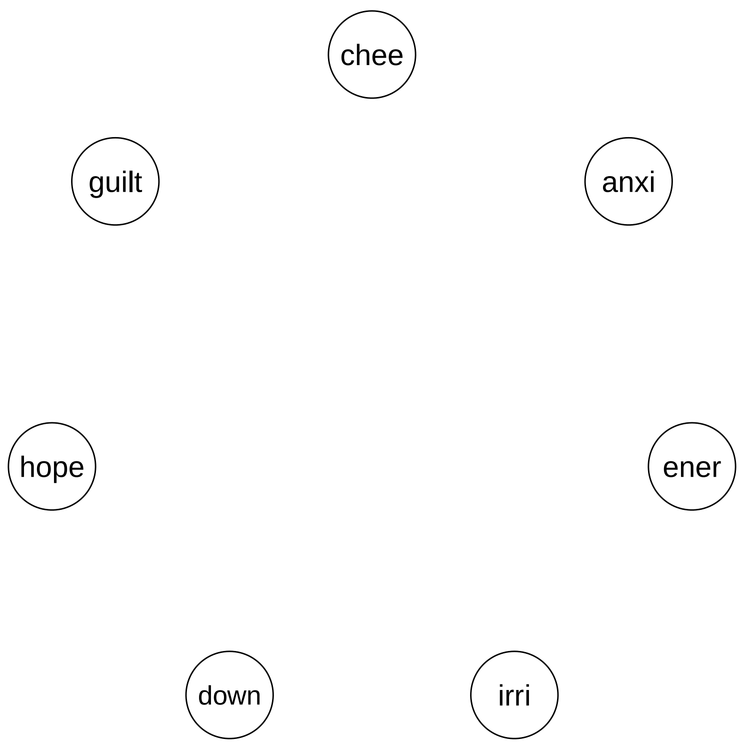

PCT tap ADM reg Pt 272 Estpoint 1

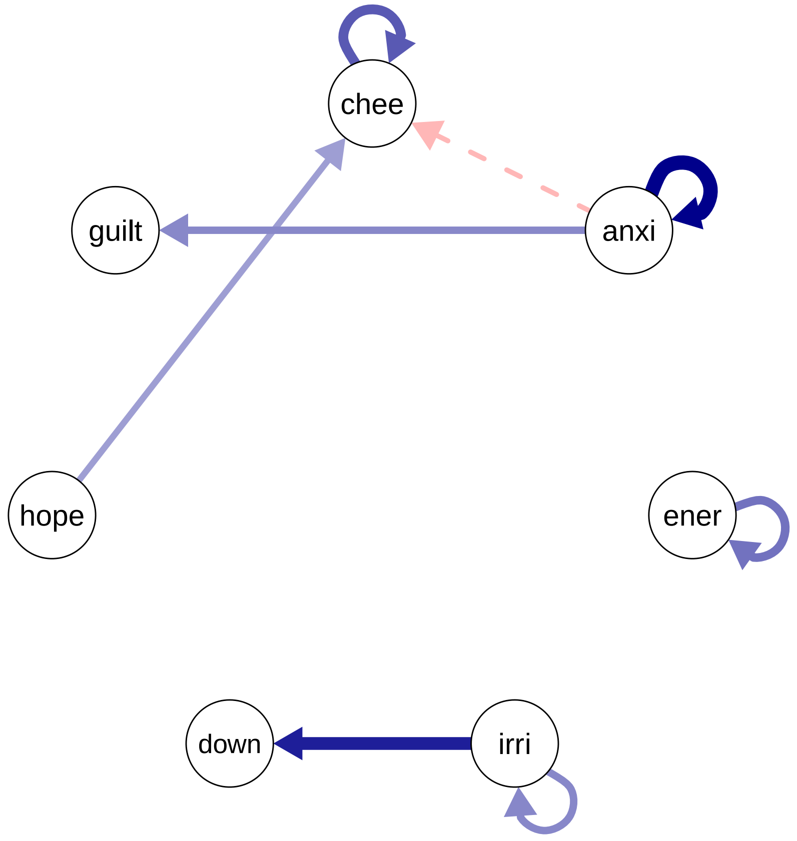

PCT tap ADM reg Pt 272 Estpoint 2

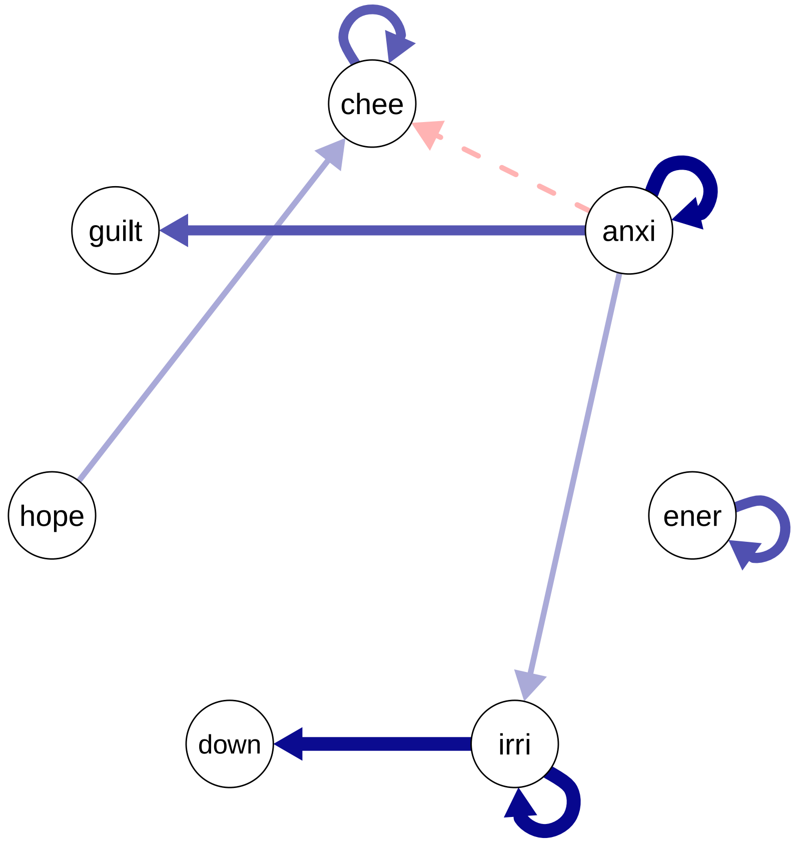

PCT tap ADM reg Pt 272 Estpoint 3

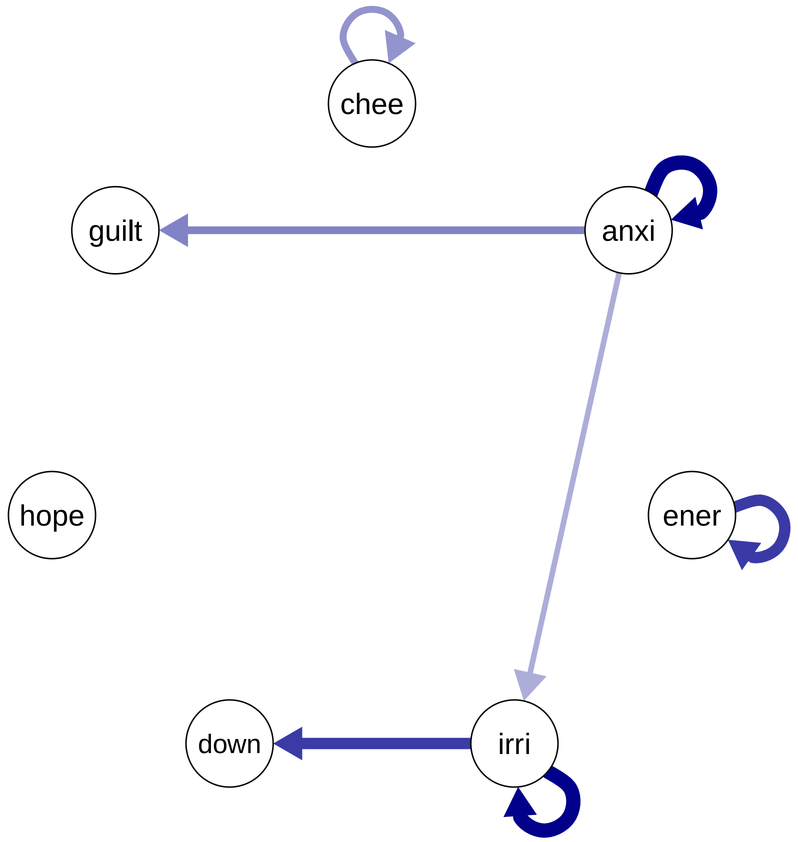

PCT tap ADM reg Pt 272 Estpoint 4

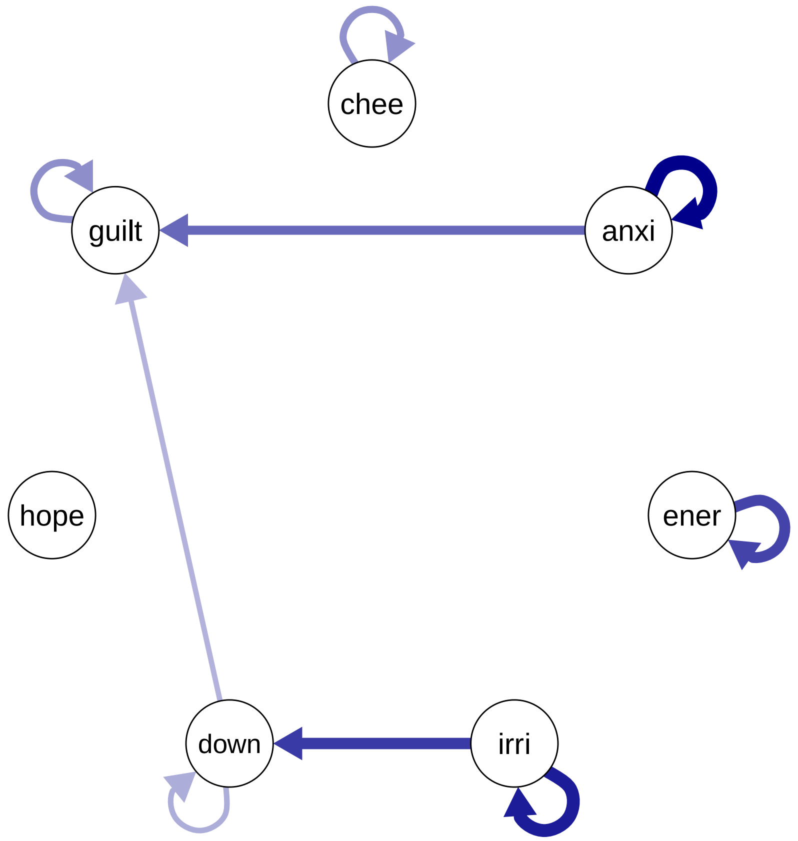

PCT tap ADM reg Pt 272 Estpoint 5

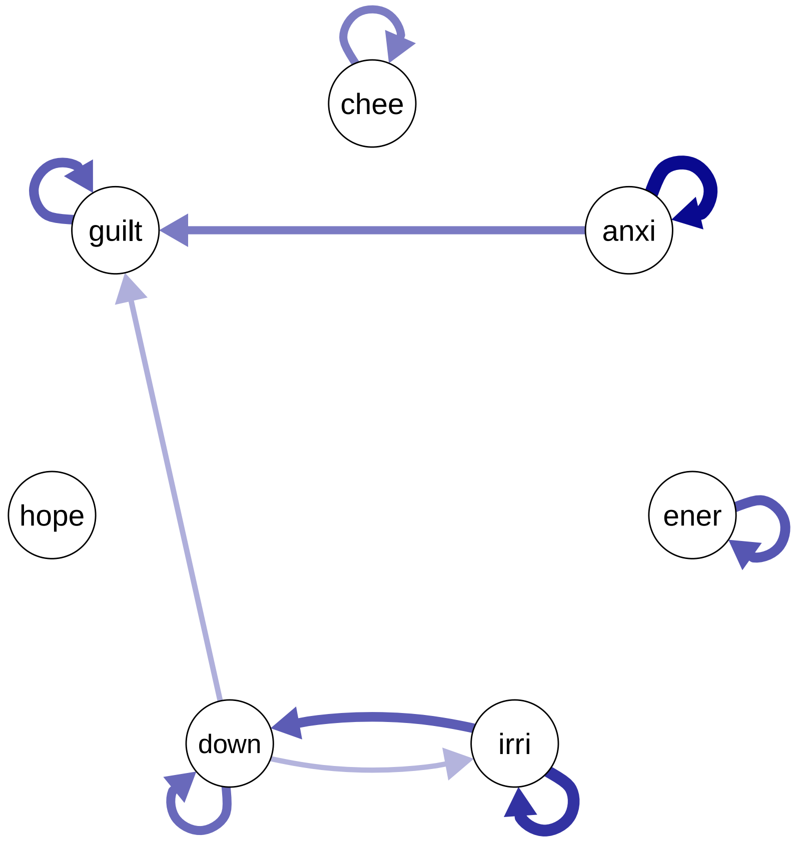

PCT tap ADM reg Pt 272 Estpoint 6

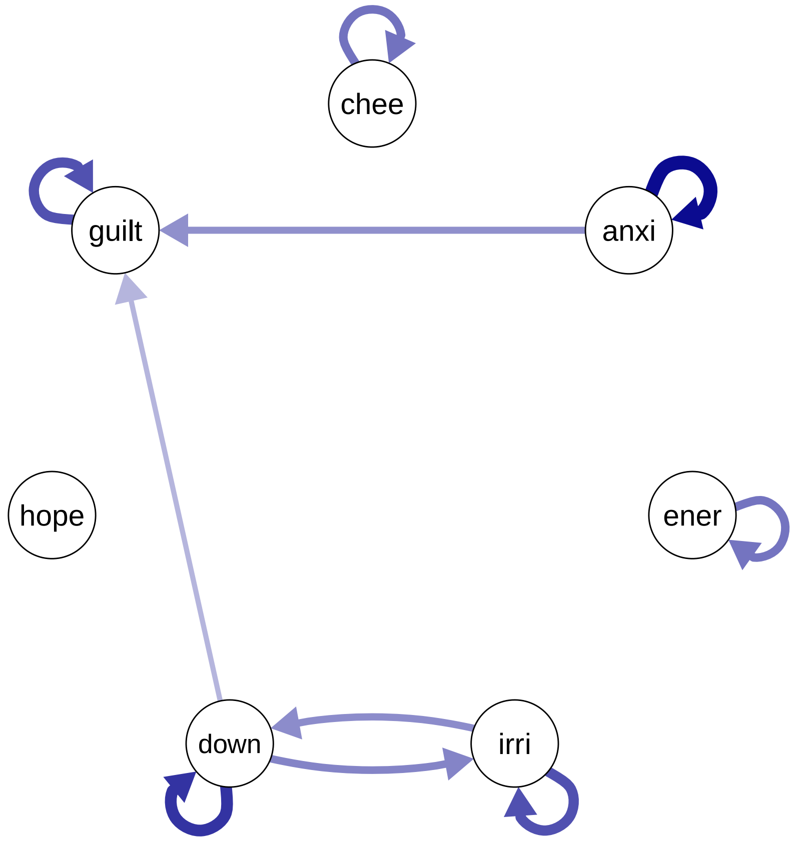

PCT tap ADM reg Pt 272 Estpoint 7

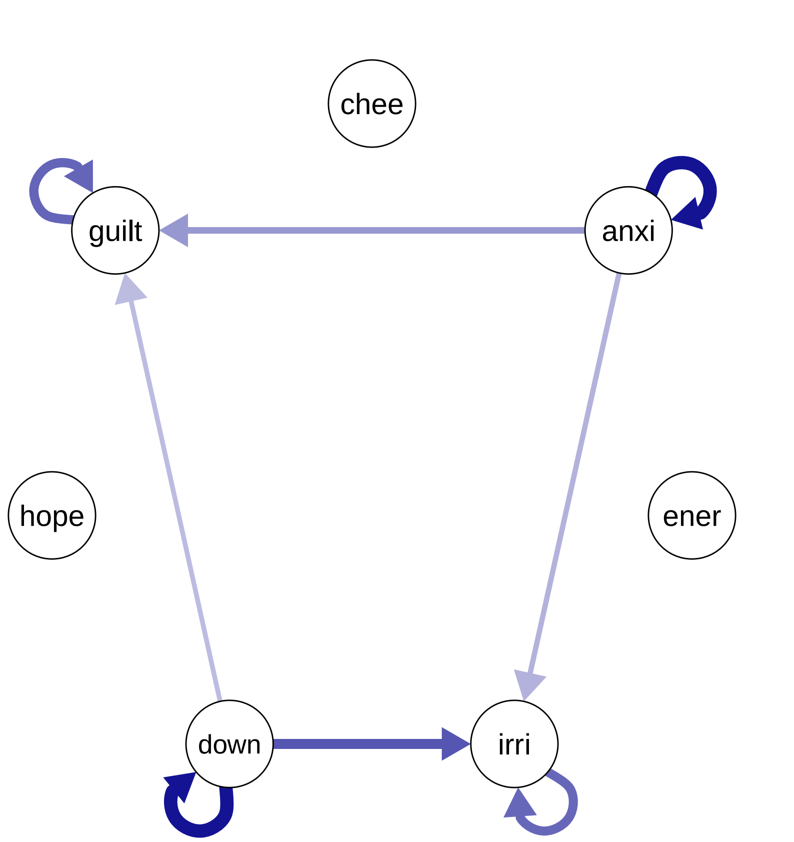

PCT tap ADM reg Pt 272 Estpoint 8

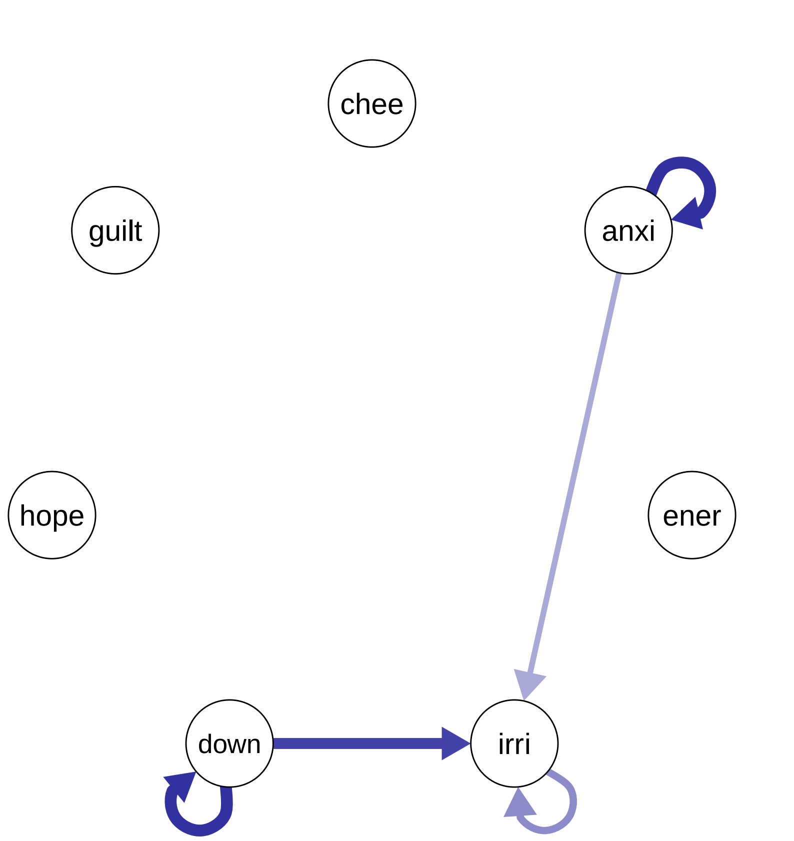

PCT tap ADM reg Pt 254 Estpoint 1

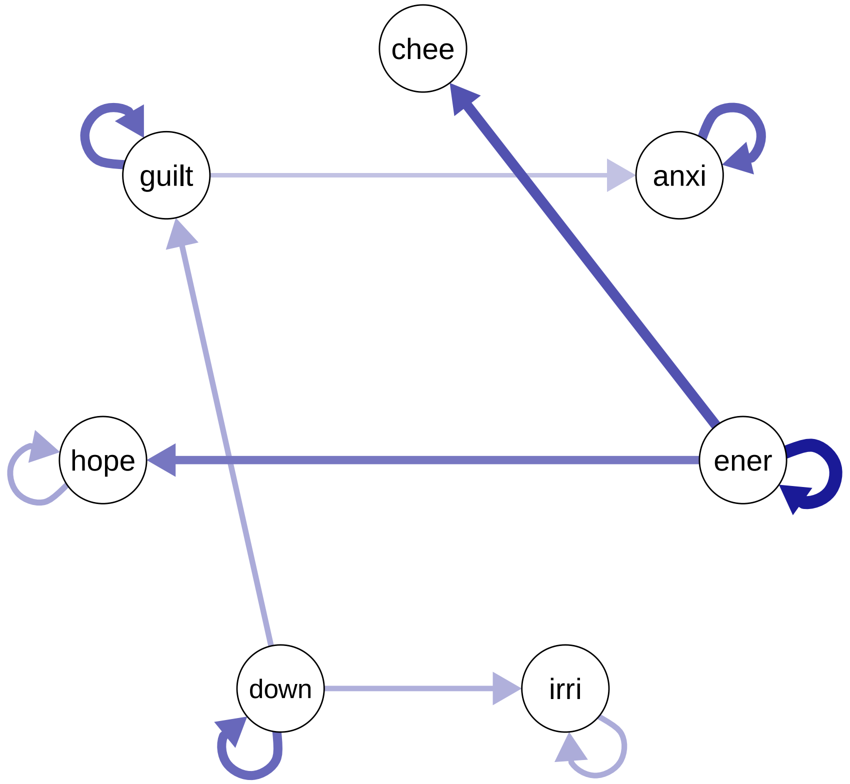

PCT tap ADM reg Pt 254 Estpoint 2

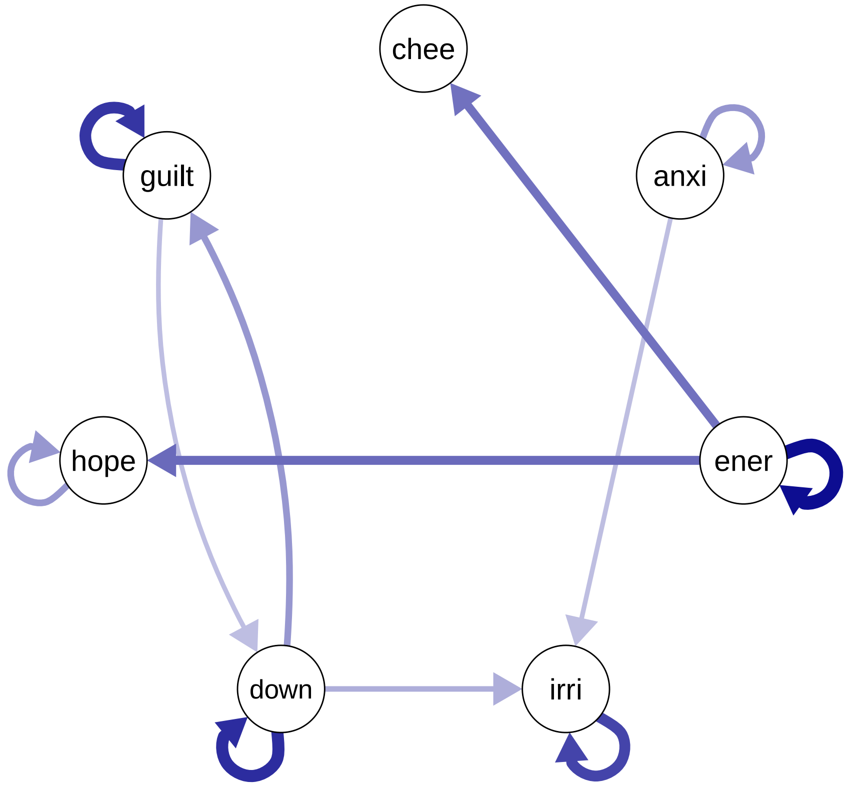

PCT tap ADM reg Pt 254 Estpoint 3

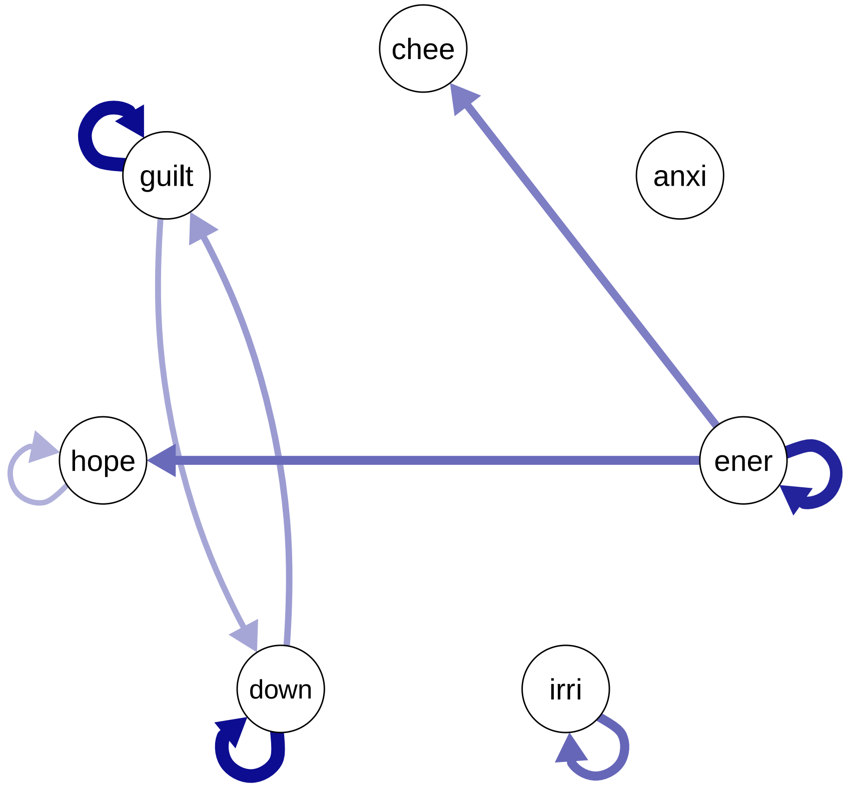

PCT tap ADM reg Pt 254 Estpoint 4

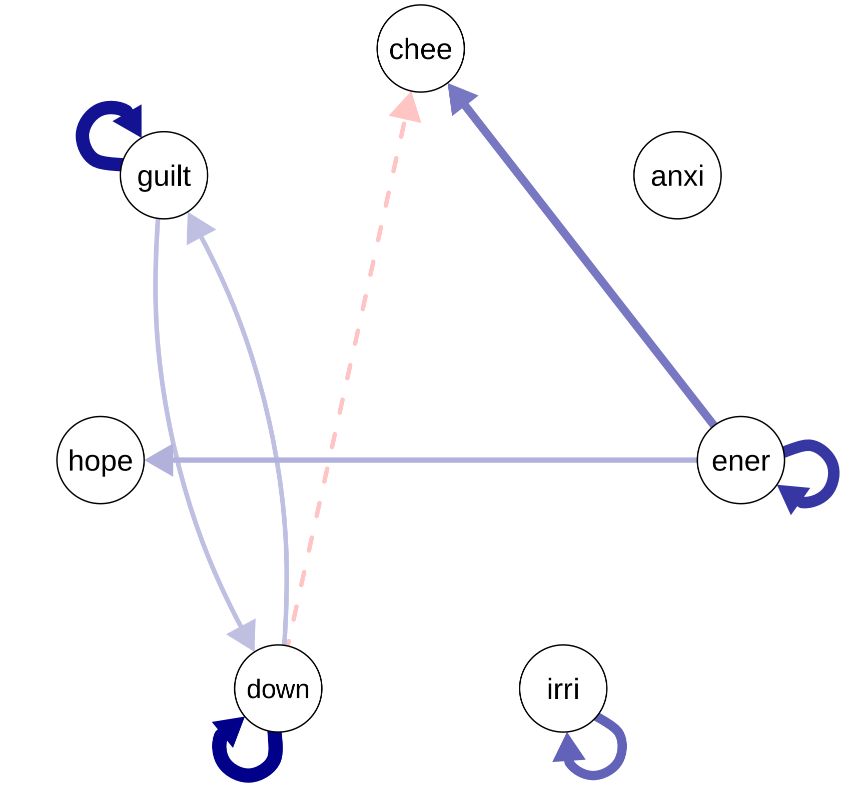

PCT tap ADM reg Pt 254 Estpoint 5

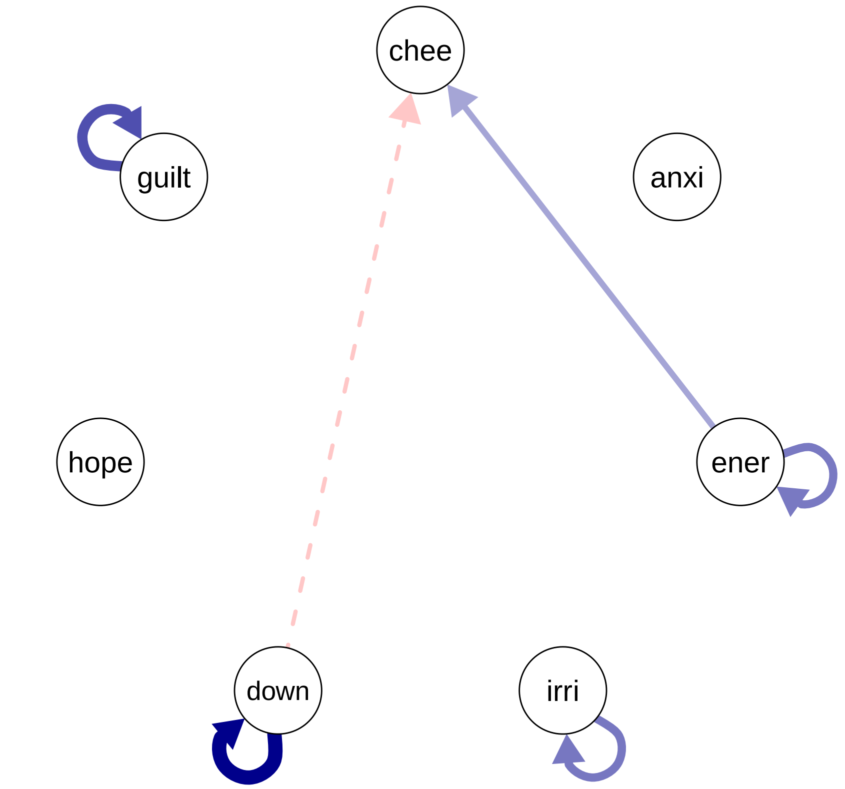

PCT tap ADM reg Pt 254 Estpoint 6

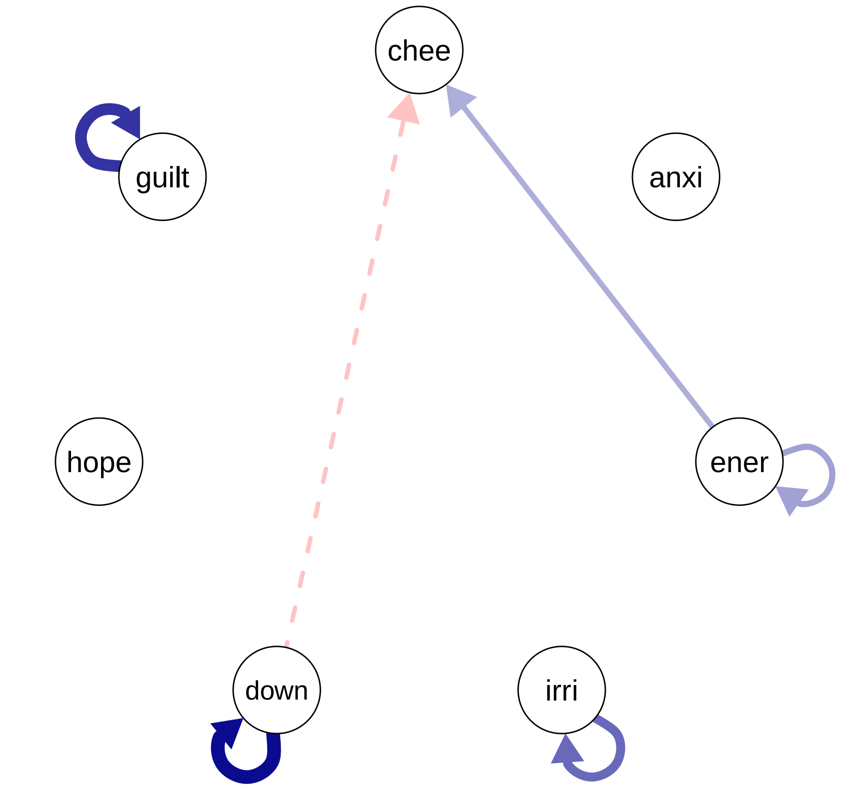

PCT tap ADM reg Pt 254 Estpoint 7

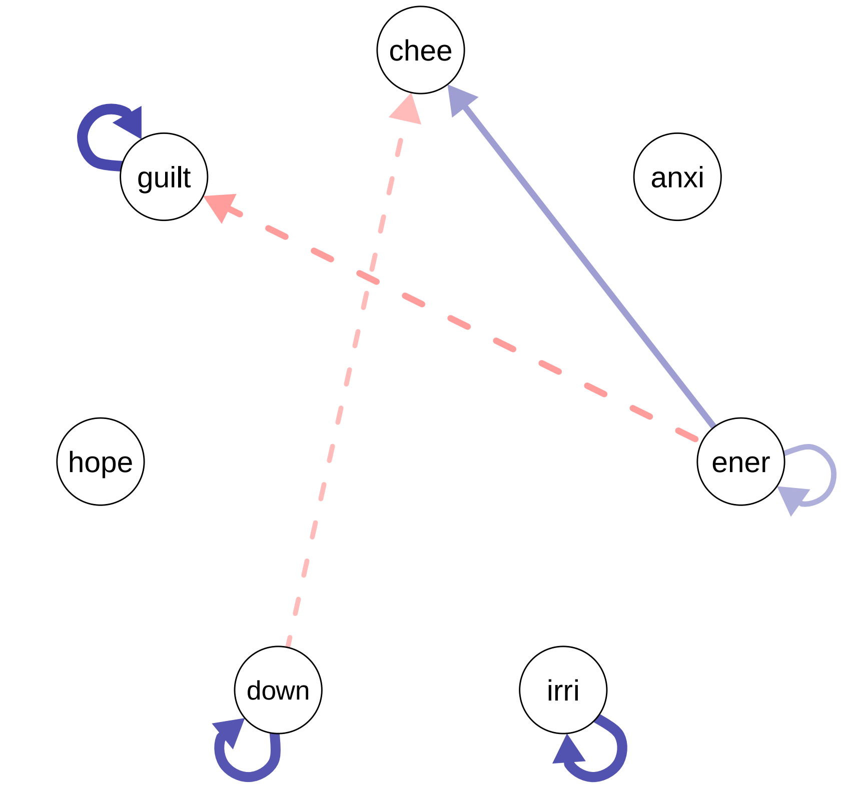

PCT tap ADM reg Pt 254 Estpoint 8

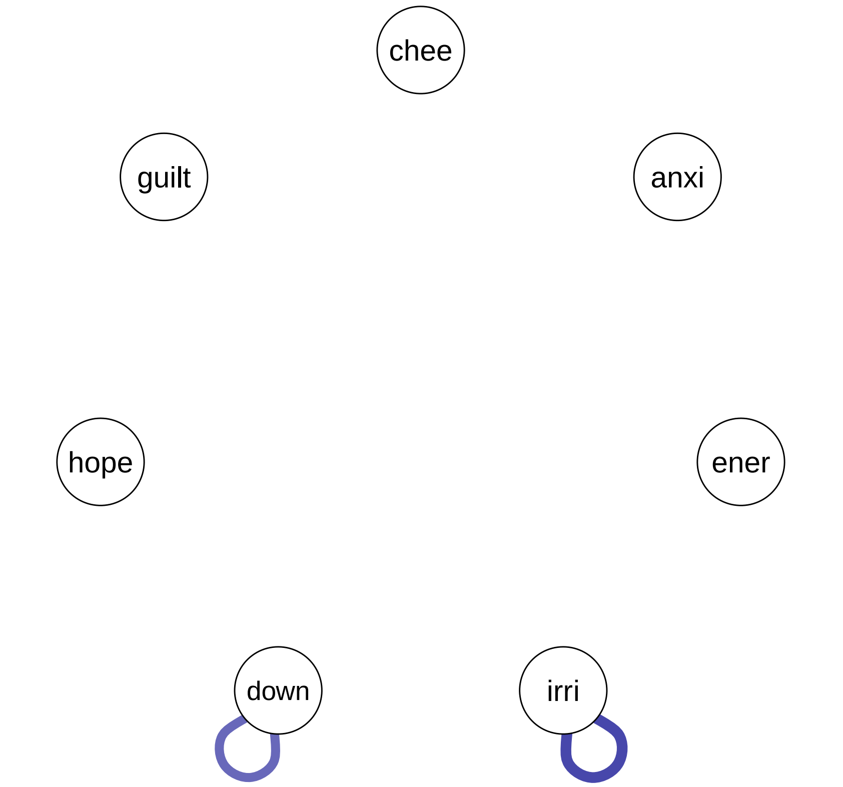

PCT tap ADM reg Pt 262 Estpoint 1

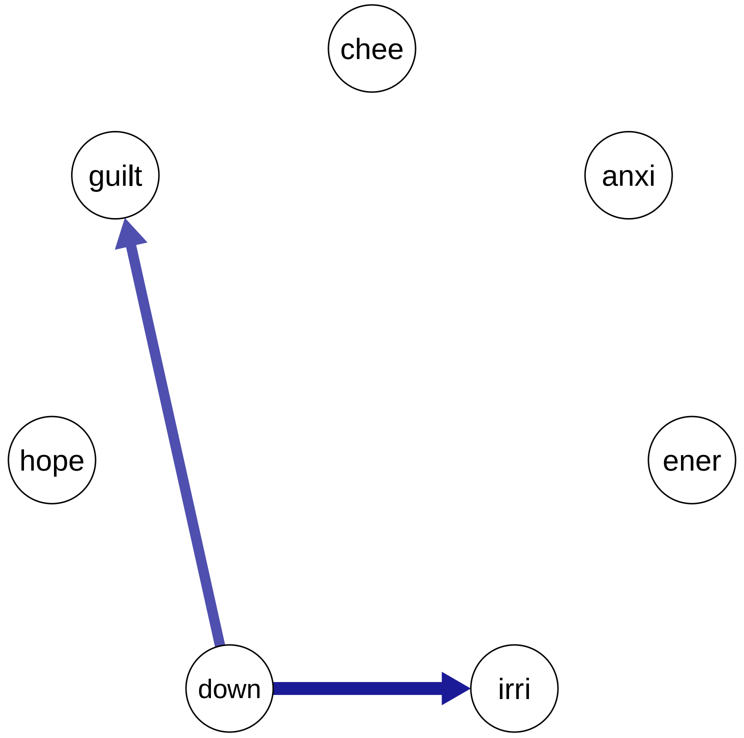

PCT tap ADM reg Pt 262 Estpoint 2

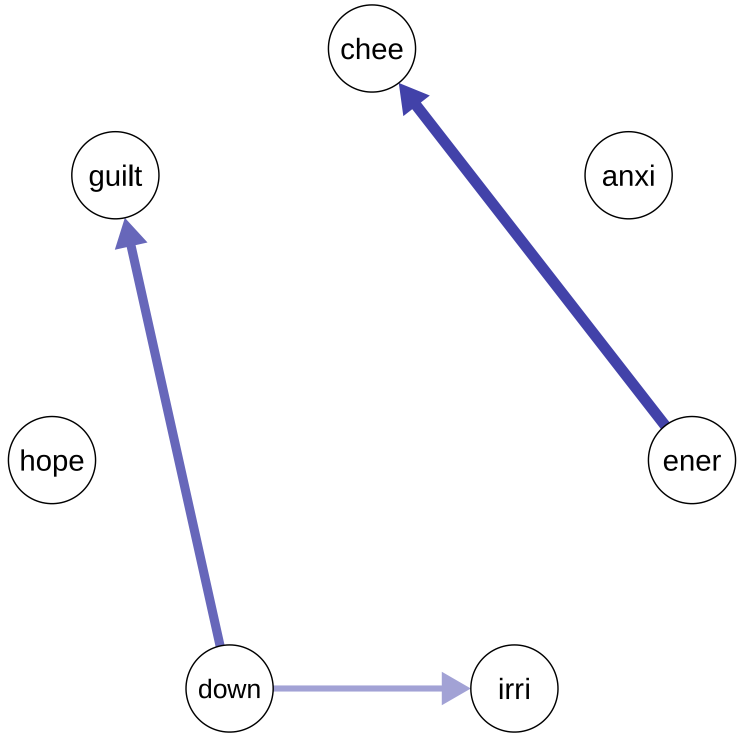

PCT tap ADM reg Pt 262 Estpoint 3

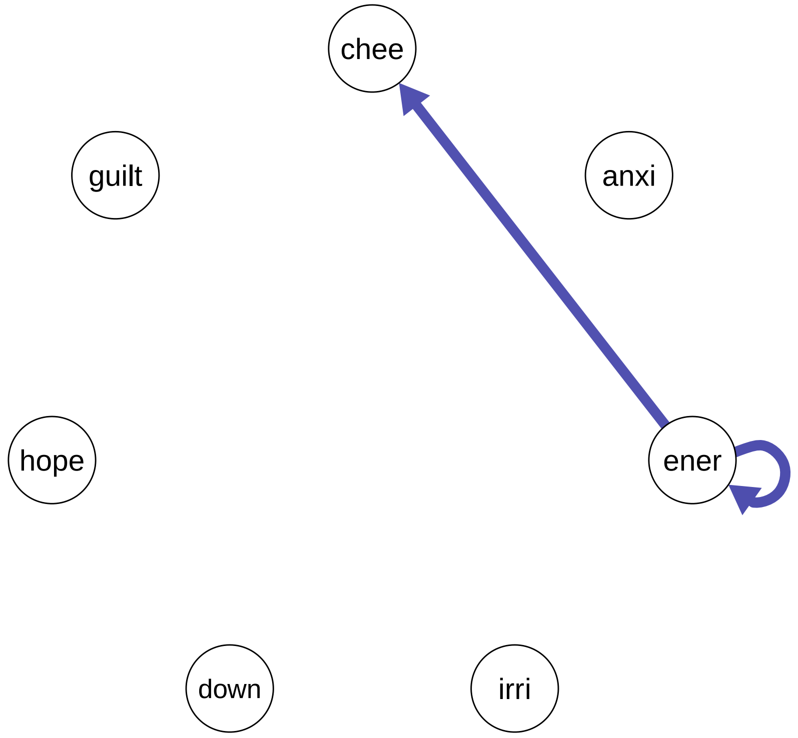

PCT tap ADM reg Pt 262 Estpoint 4

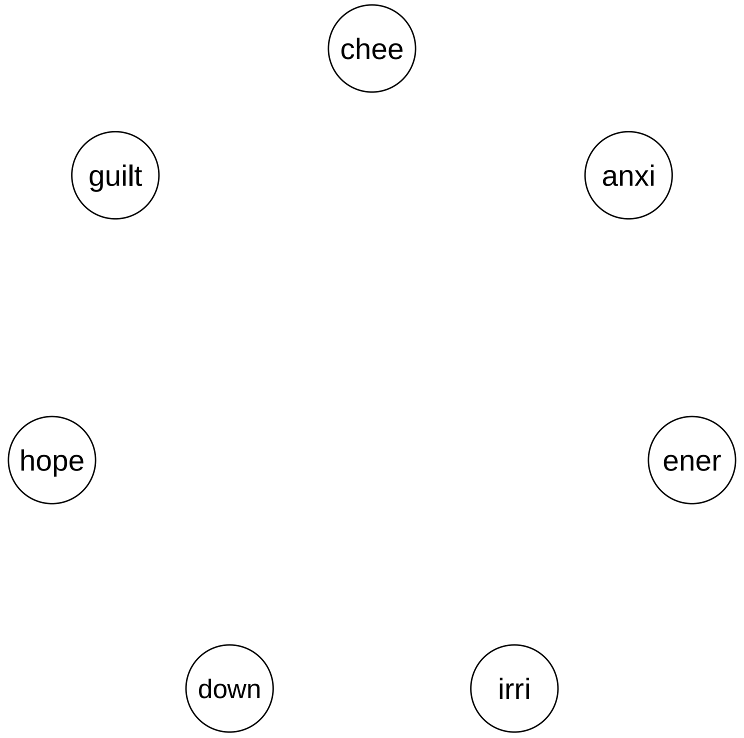

PCT tap ADM reg Pt 262 Estpoint 5

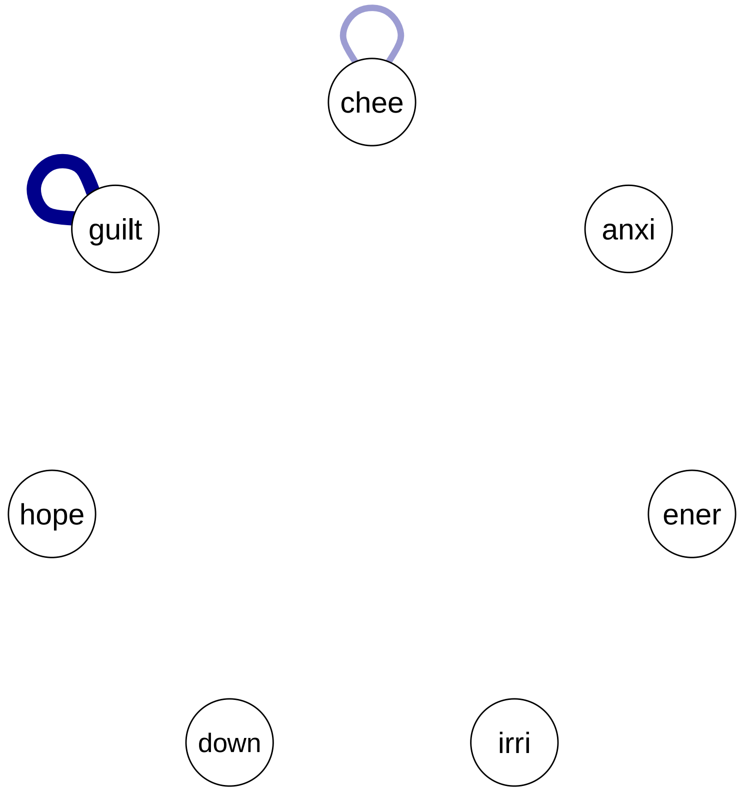

PCT tap ADM reg Pt 262 Estpoint 6

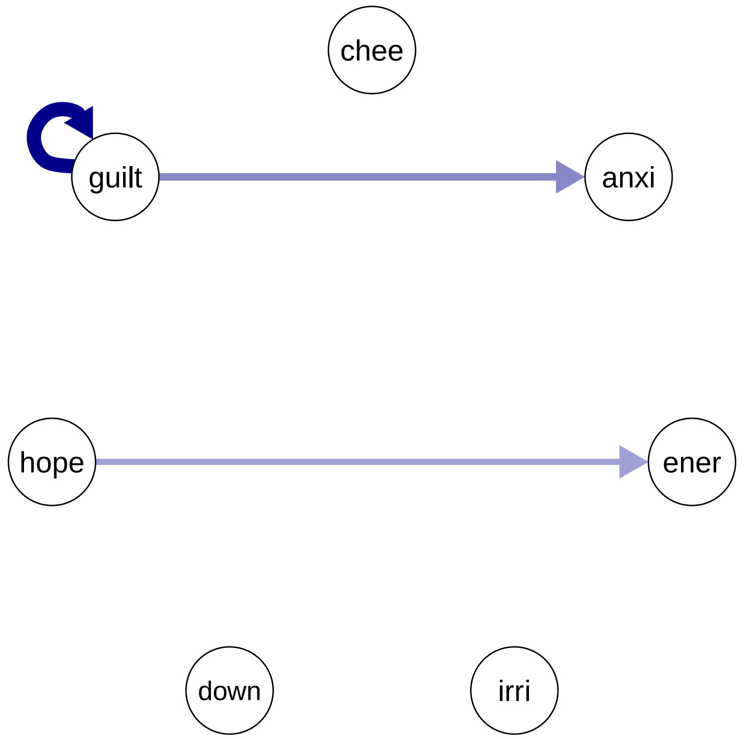

PCT tap ADM reg Pt 262 Estpoint 7

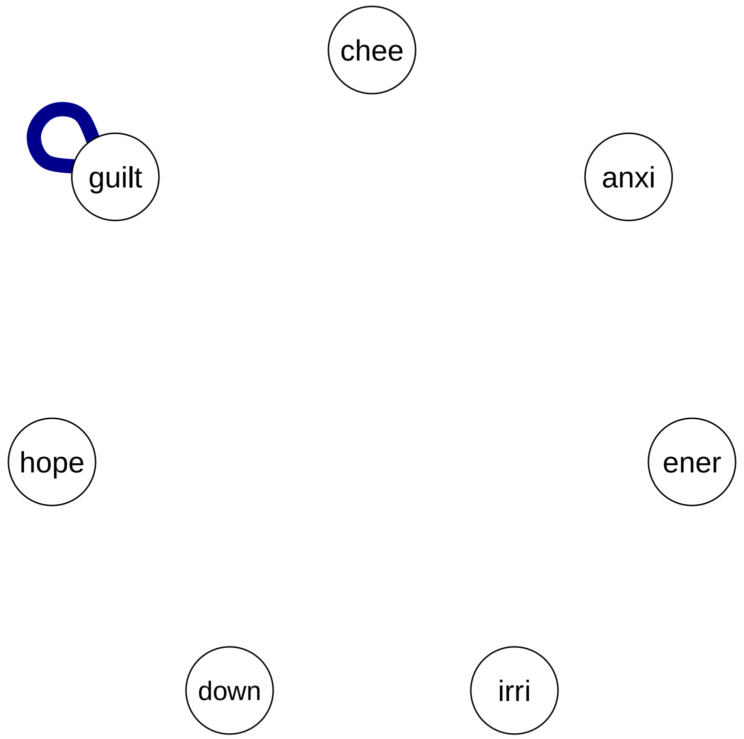

PCT tap ADM reg Pt 262 Estpoint 8

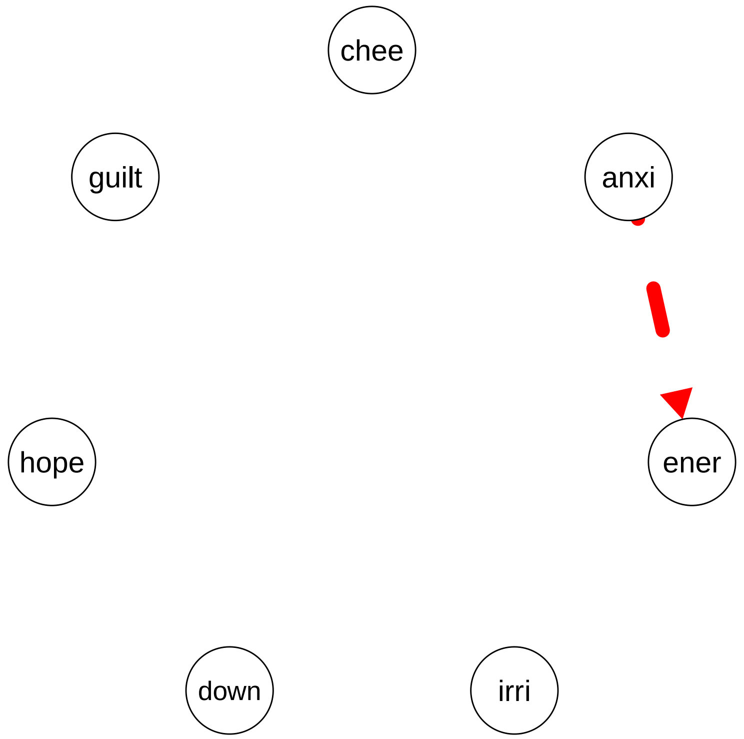

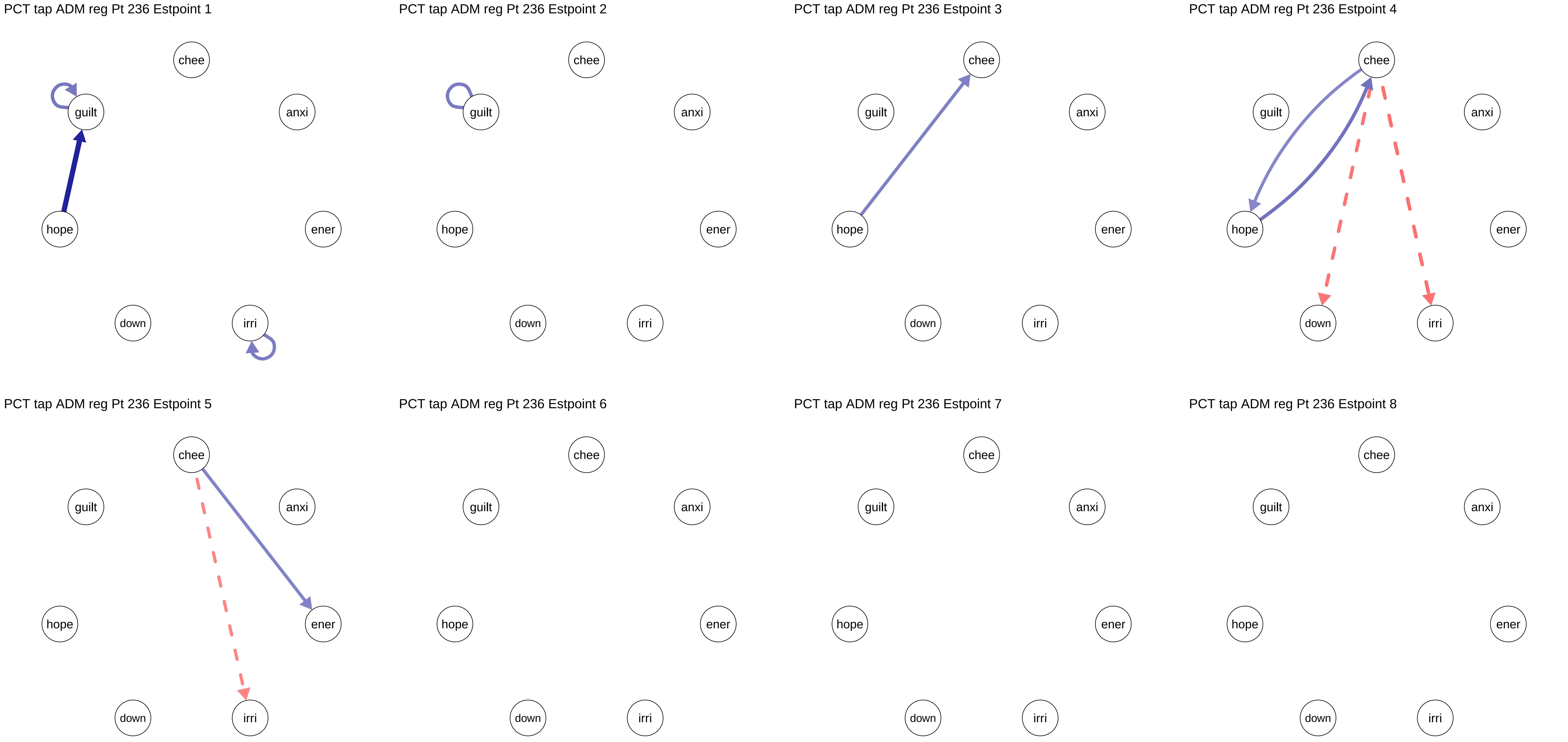
PCT tap ADM reg Pt 236 Estpoint 1
chee
guilt
anxi
hope
ener
down
irri
PCT tap ADM reg Pt 236 Estpoint 2
chee
guilt
anxi
hope
ener
down
irri
PCT tap ADM reg Pt 236 Estpoint 3
chee
guilt
anxi
hope
ener
down
irri
PCT tap ADM reg Pt 236 Estpoint 4
chee
guilt
anxi
hope
ener
down
irri
PCT tap ADM reg Pt 236 Estpoint 5
chee
guilt
anxi
hope
ener
down
irri
PCT tap ADM reg Pt 236 Estpoint 6
chee
guilt
anxi
hope
ener
down
irri
PCT tap ADM reg Pt 236 Estpoint 7
chee
guilt
anxi
hope
ener
down
irri
PCT tap ADM reg Pt 236 Estpoint 8
chee
guilt
anxi
hope
ener
down
irri

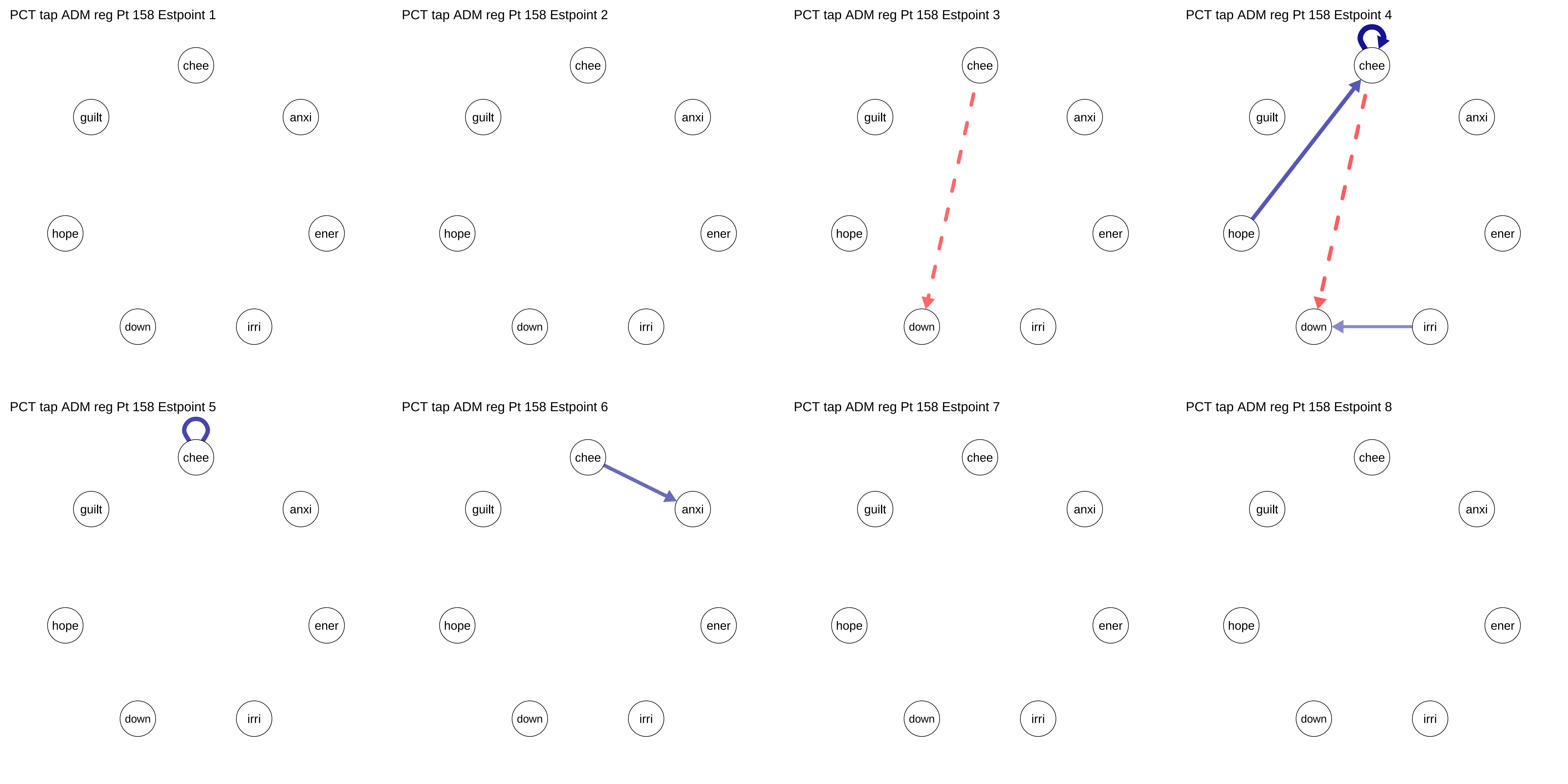
PCT tap ADM reg Pt 158 Estpoint 1
chee
guilt
anxi
hope
ener
down
irri
PCT tap ADM reg Pt 158 Estpoint 2
chee
guilt
anxi
hope
ener
down
irri
PCT tap ADM reg Pt 158 Estpoint 3
chee
guilt
anxi
hope
ener
down
irri
PCT tap ADM reg Pt 158 Estpoint 4
chee
guilt
anxi
hope
ener
down
irri
PCT tap ADM reg Pt 158 Estpoint 5
chee
guilt
anxi
hope
ener
down
irri
PCT tap ADM reg Pt 158 Estpoint 6
chee
guilt
anxi
hope
ener
down
irri
PCT tap ADM reg Pt 158 Estpoint 7
chee
guilt
anxi
hope
ener
down
irri
PCT tap ADM reg Pt 158 Estpoint 8
chee
guilt
anxi
hope
ener
down
irri

ADM only reg Pt 249 Estpoint 1

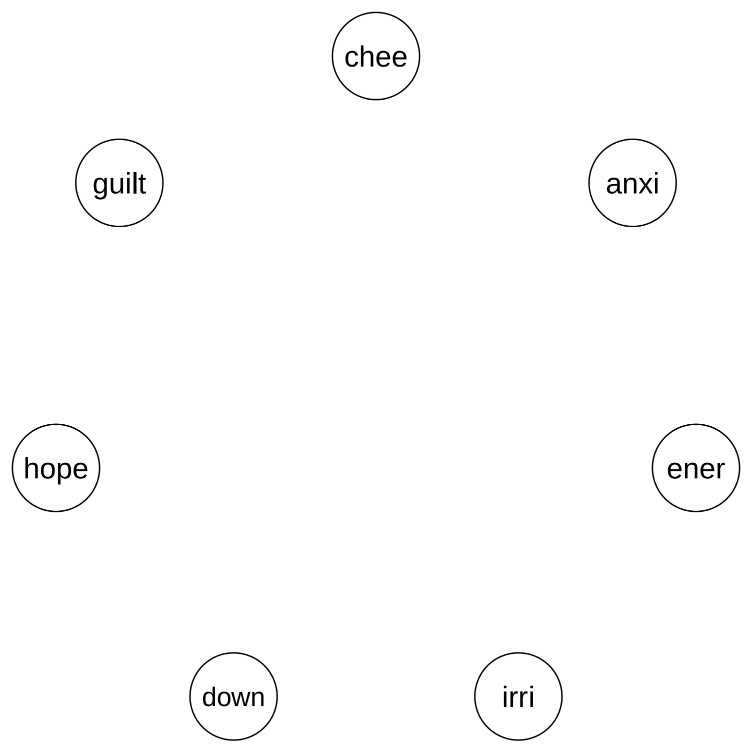

ADM only reg Pt 249 Estpoint 2

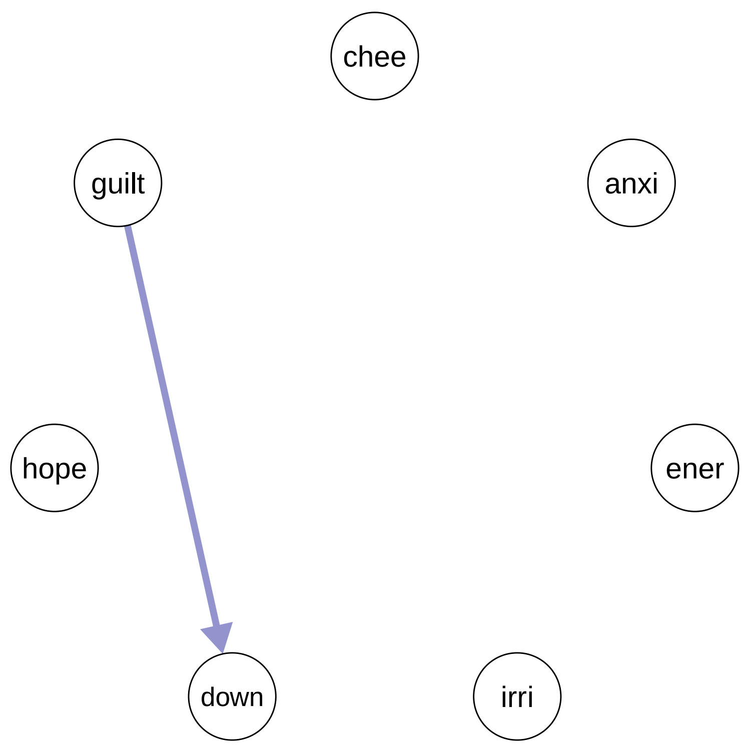

ADM only reg Pt 249 Estpoint 3

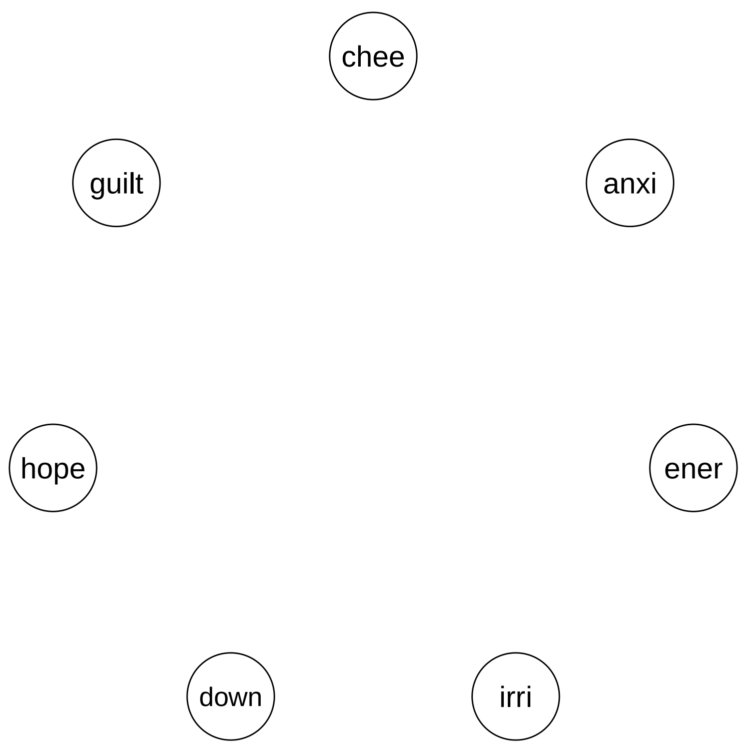

ADM only reg Pt 249 Estpoint 4

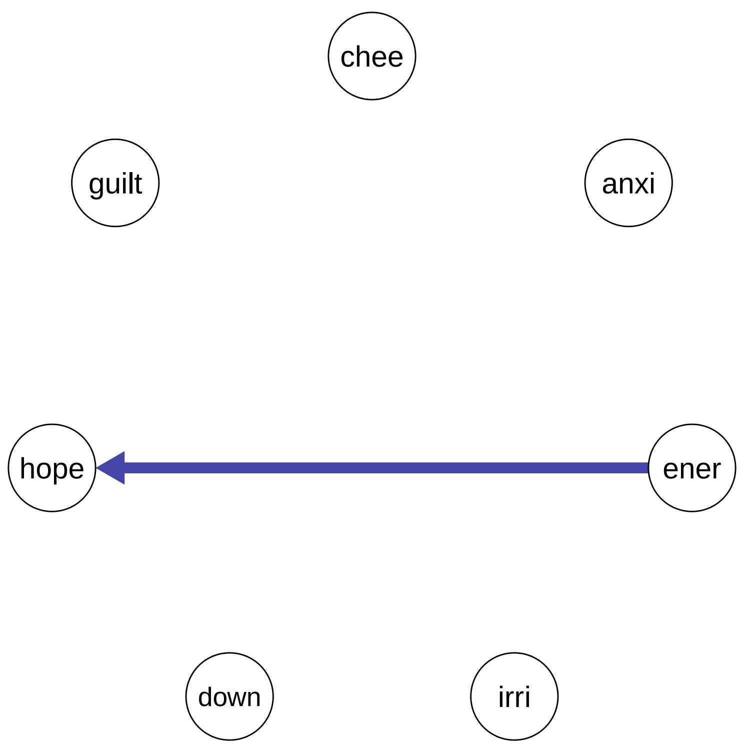

ADM only reg Pt 249 Estpoint 5

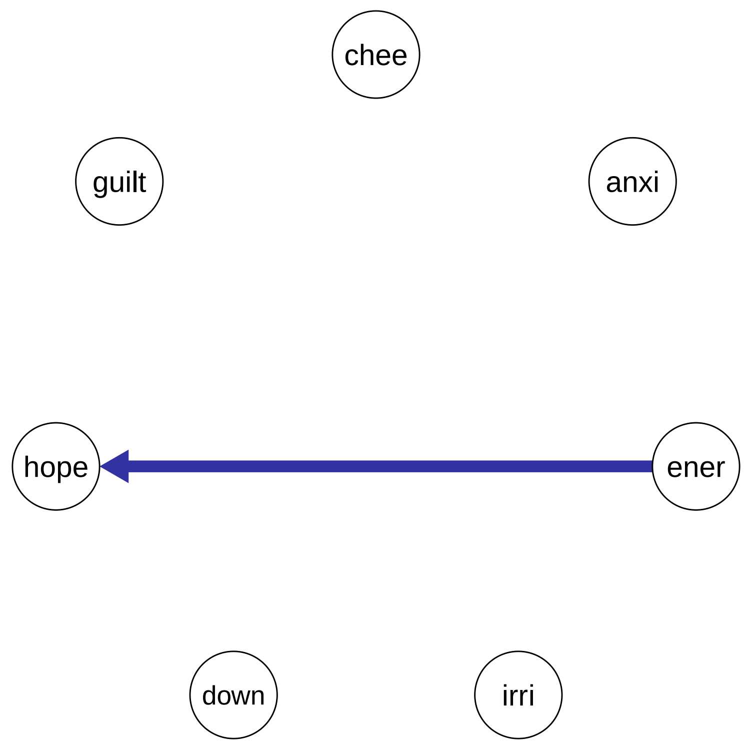

ADM only reg Pt 249 Estpoint 6

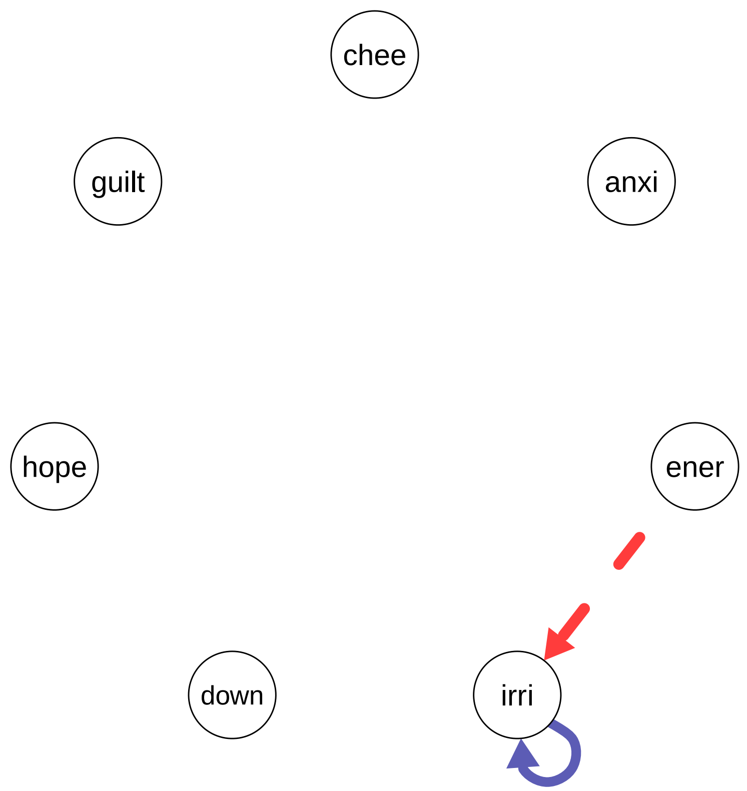

ADM only reg Pt 249 Estpoint 7

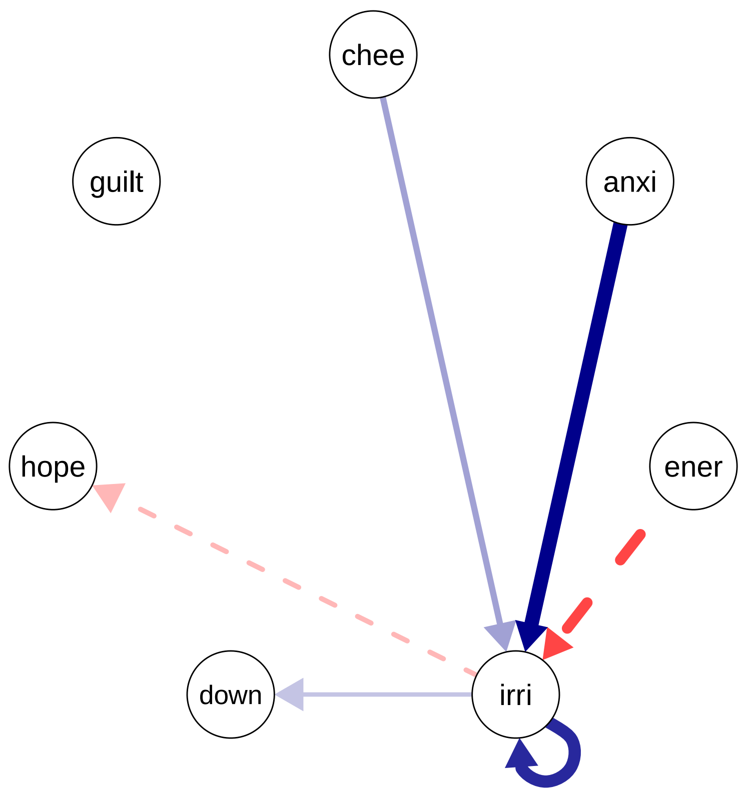

ADM only reg Pt 249 Estpoint 8

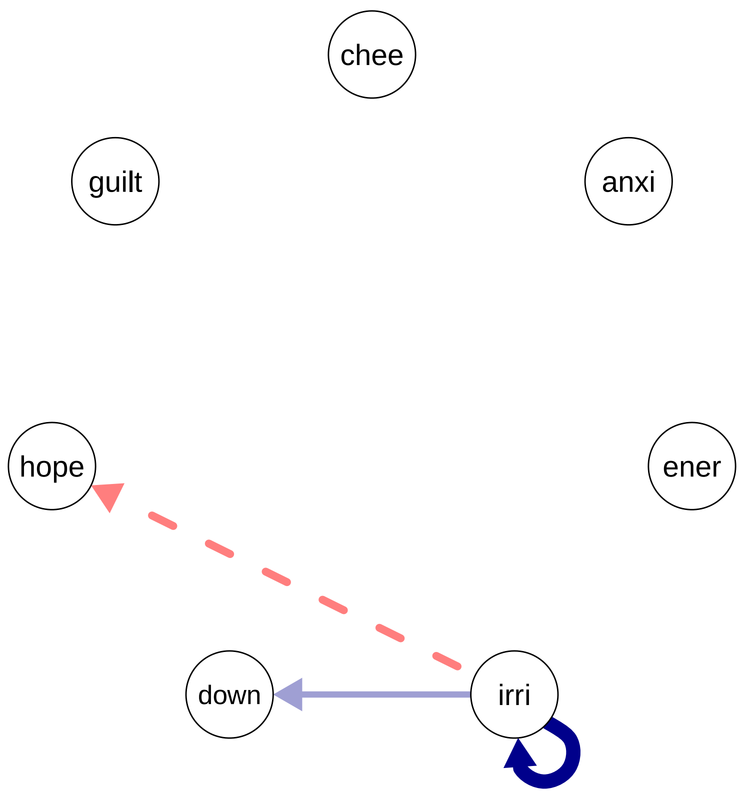

ADM only reg Pt 273 Estpoint 1

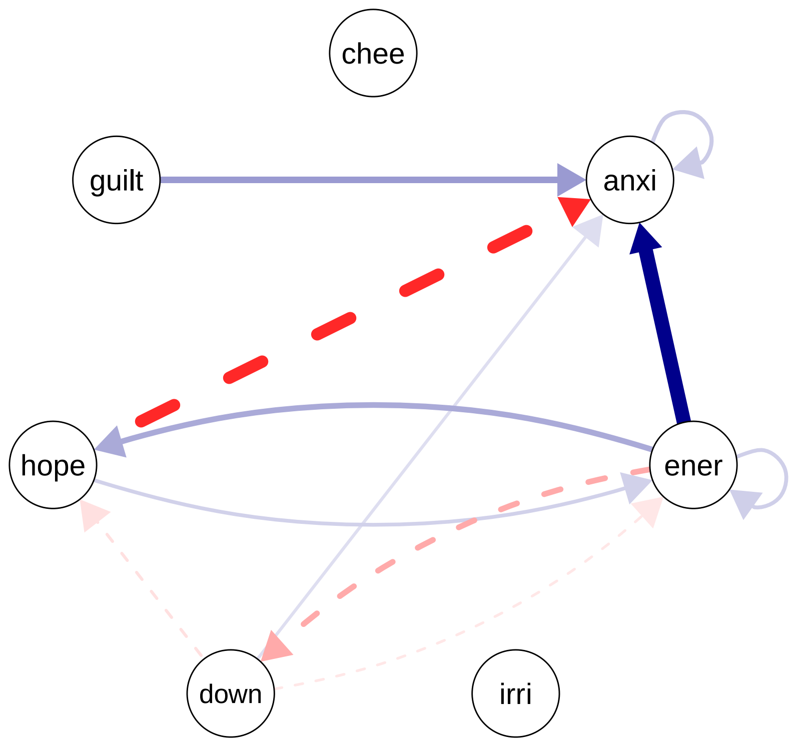

ADM only reg Pt 273 Estpoint 2

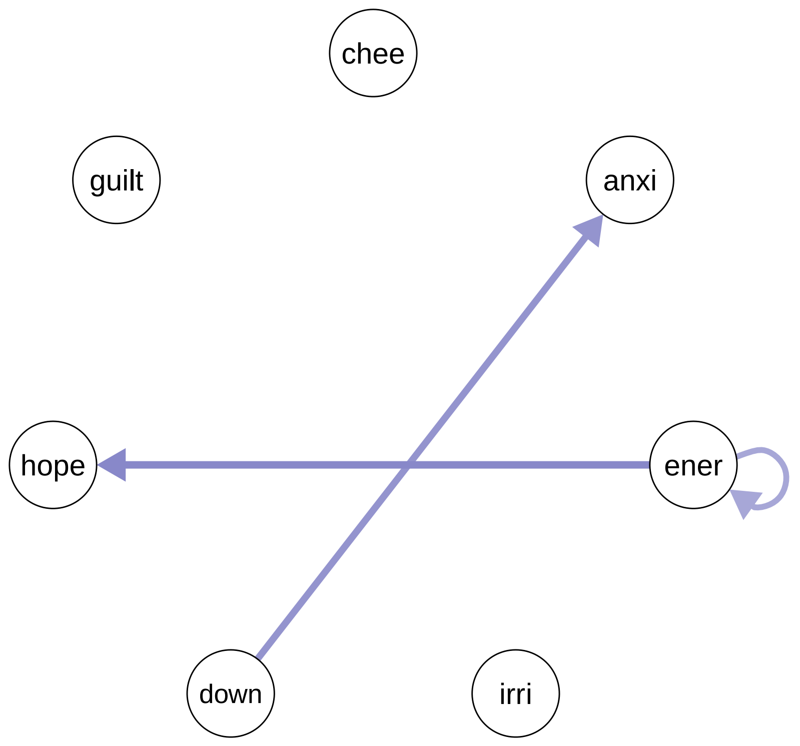

ADM only reg Pt 273 Estpoint 3

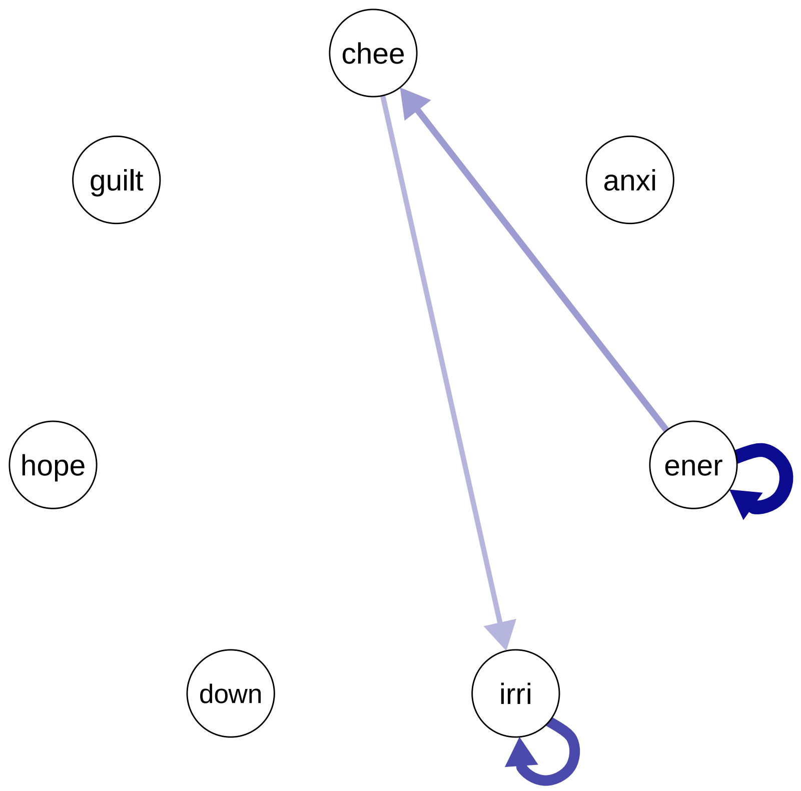

ADM only reg Pt 273 Estpoint 4

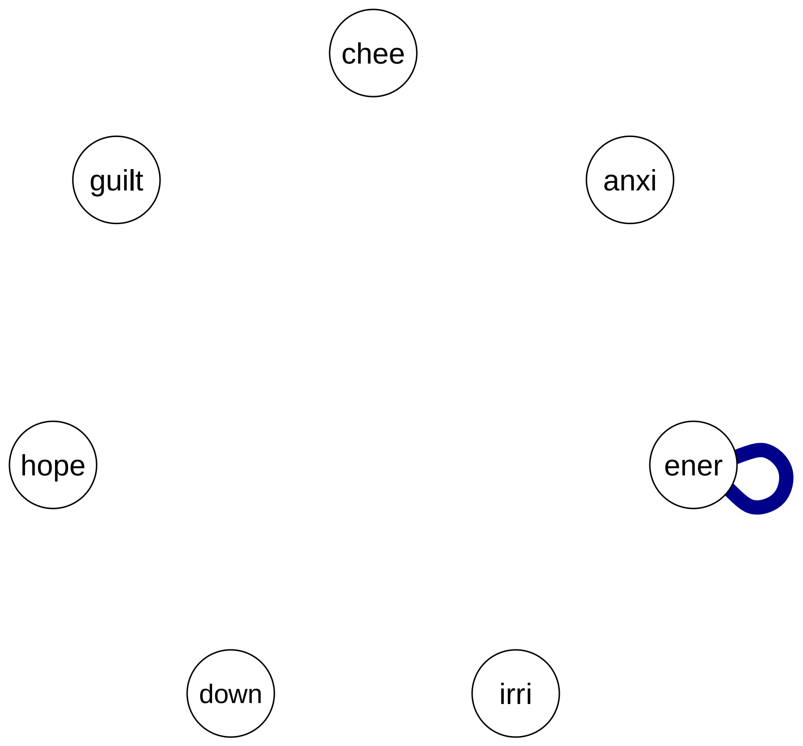

ADM only reg Pt 273 Estpoint 5

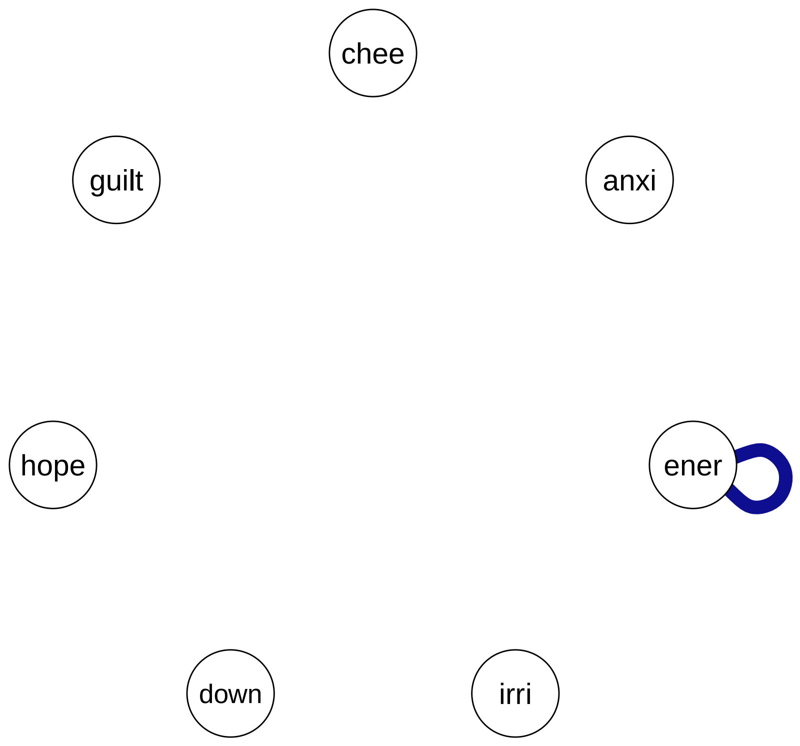

ADM only reg Pt 273 Estpoint 6

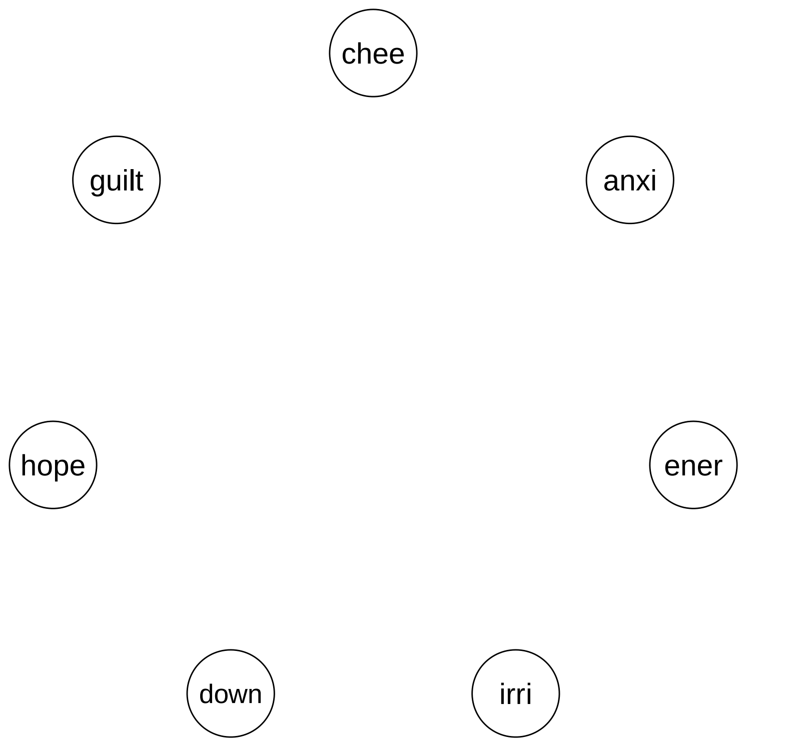

ADM only reg Pt 273 Estpoint 7

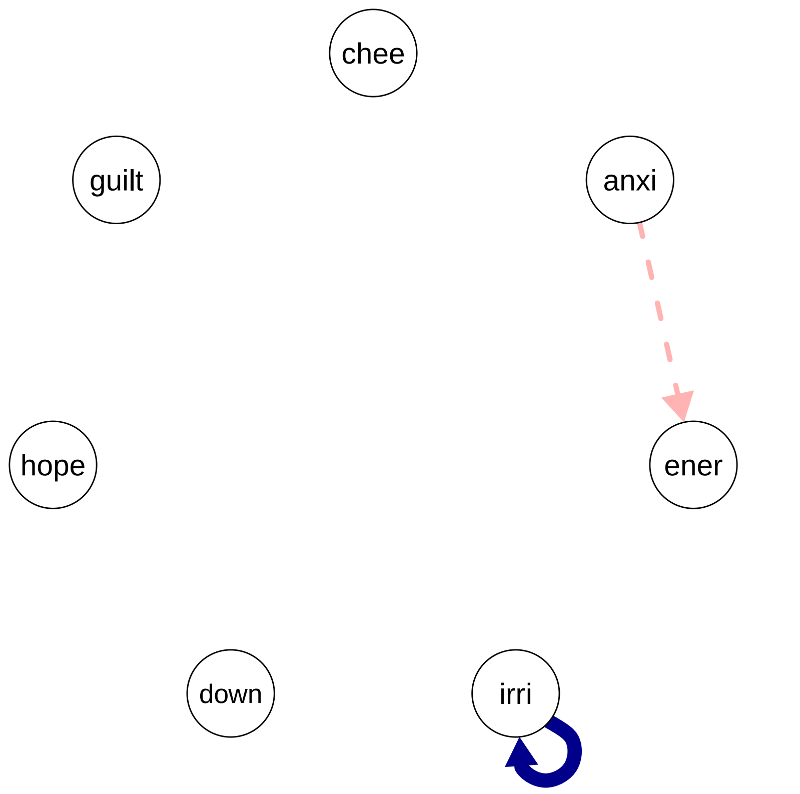

ADM only reg Pt 273 Estpoint 8

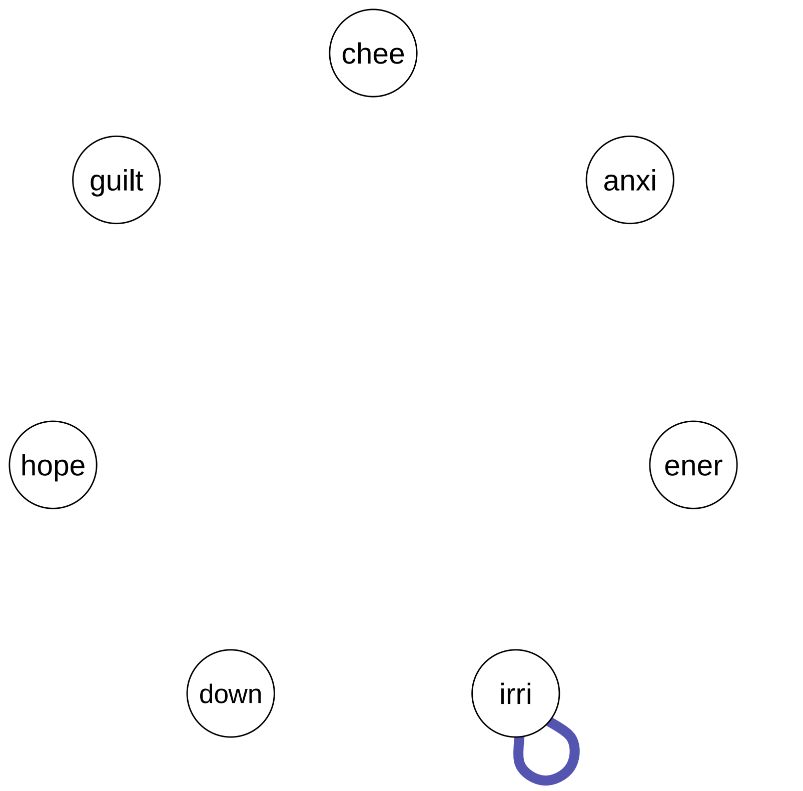

ADM only reg Pt 224 Estpoint 1

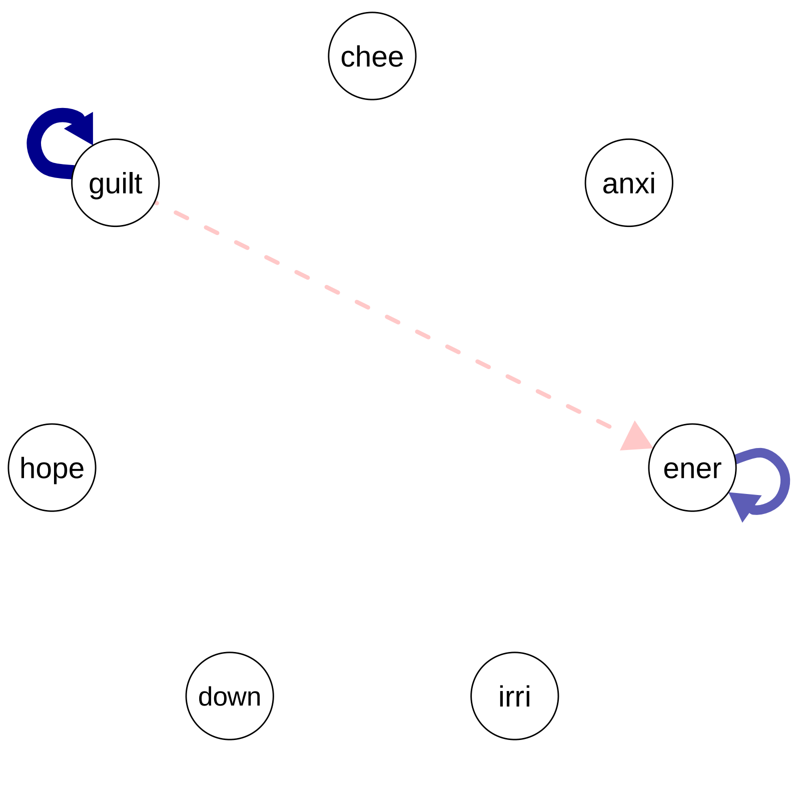

ADM only reg Pt 224 Estpoint 2

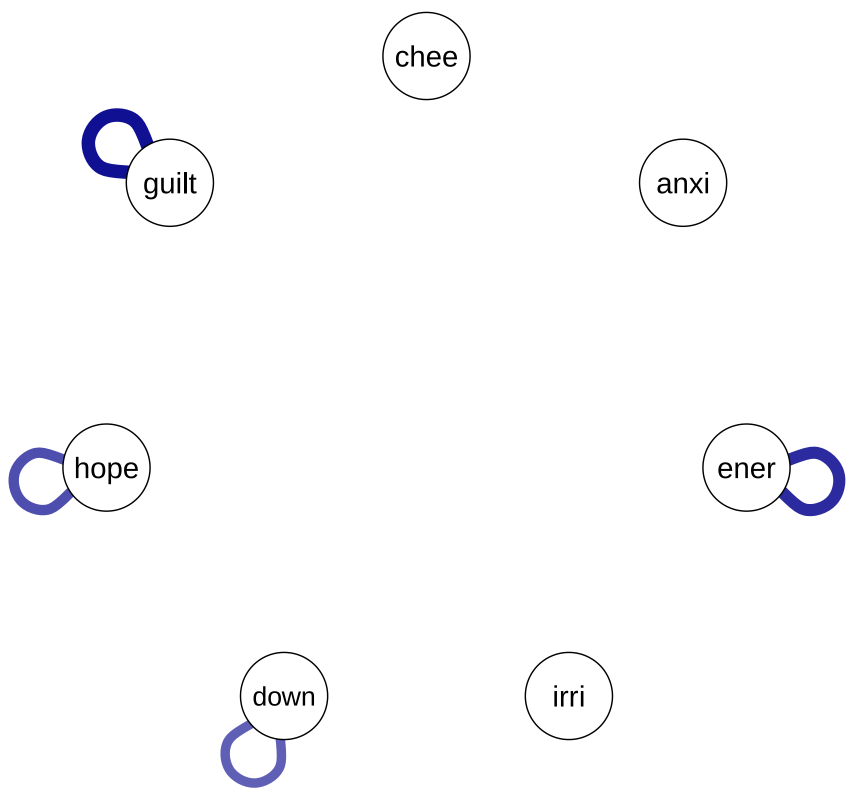

ADM only reg Pt 224 Estpoint 3

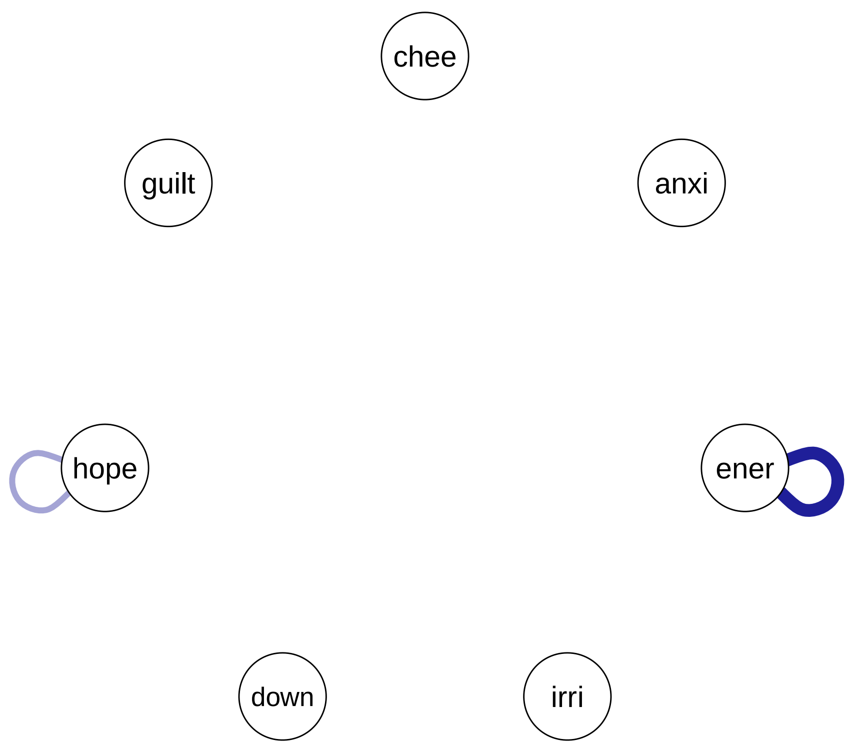

ADM only reg Pt 224 Estpoint 4

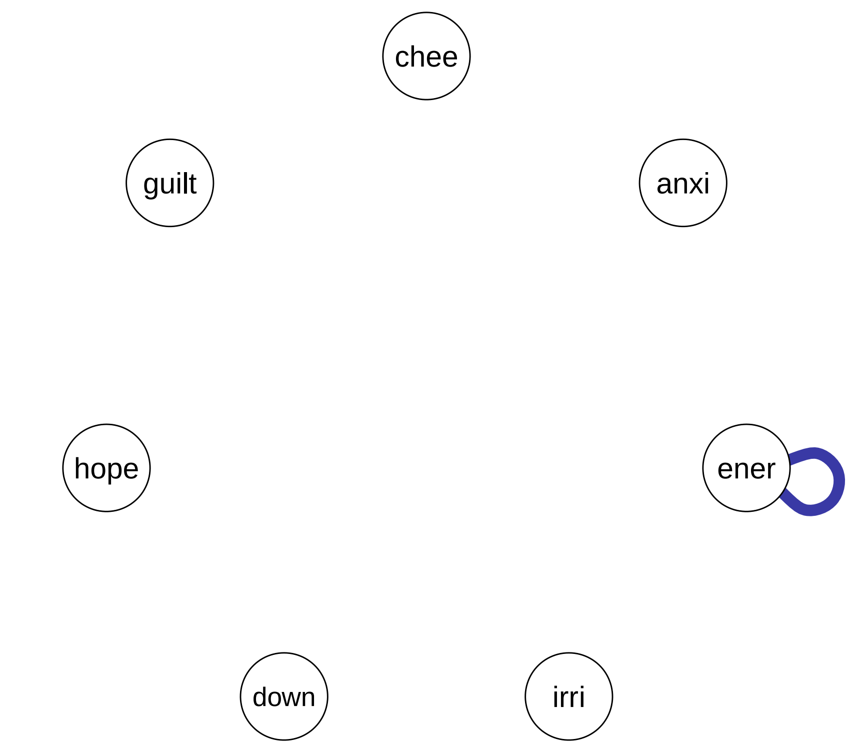

ADM only reg Pt 224 Estpoint 5

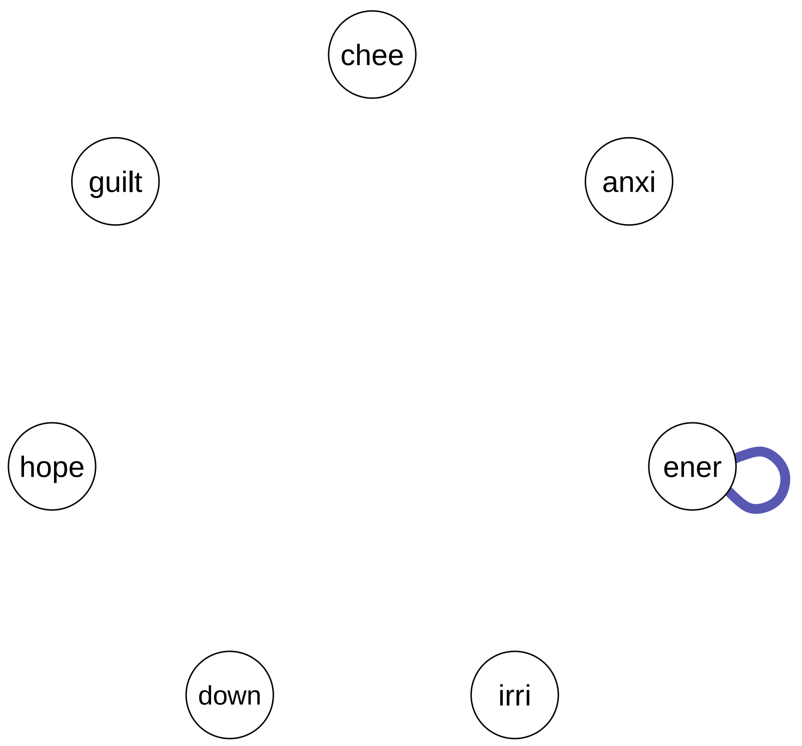

ADM only reg Pt 224 Estpoint 6

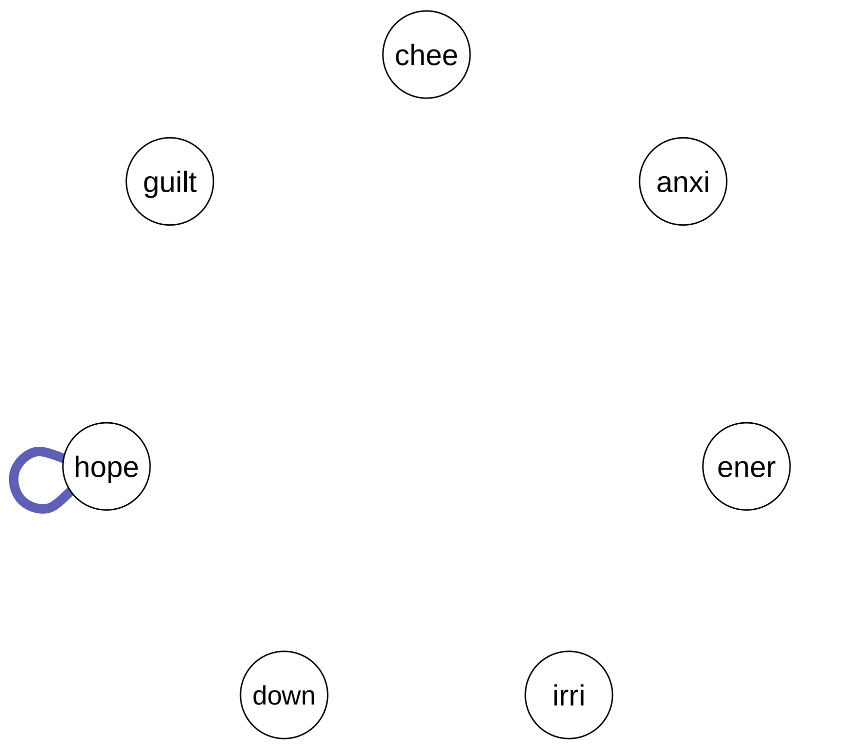

ADM only reg Pt 224 Estpoint 7

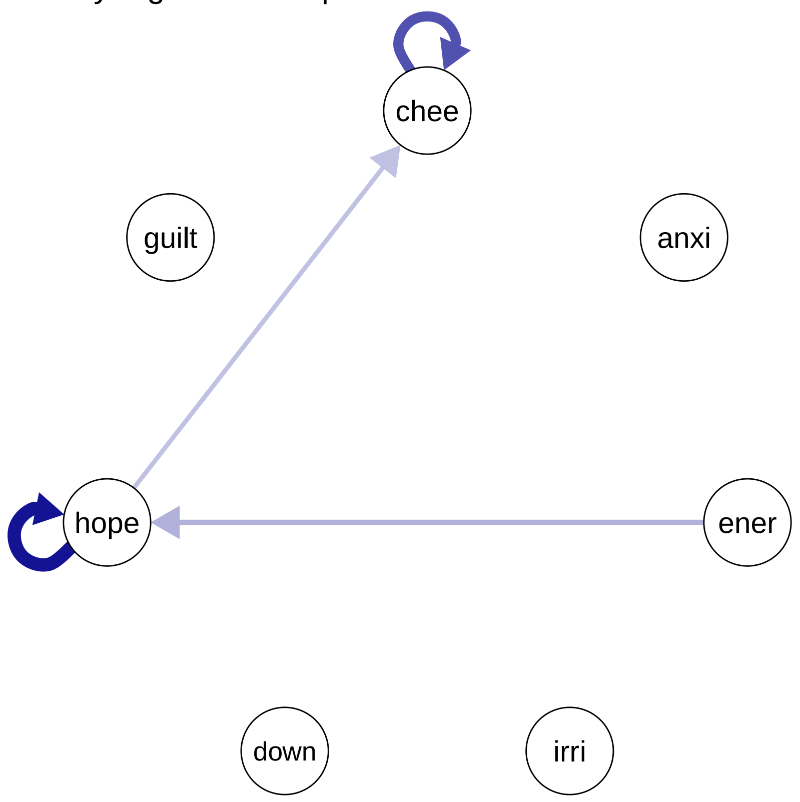

ADM only reg Pt 224 Estpoint 8

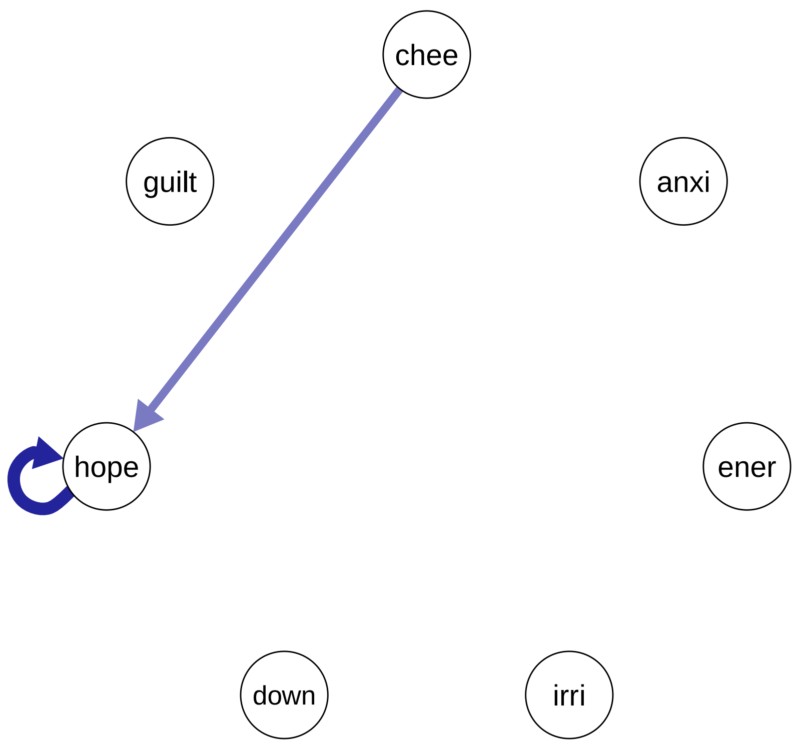

ADM only reg Pt 259 Estpoint 1

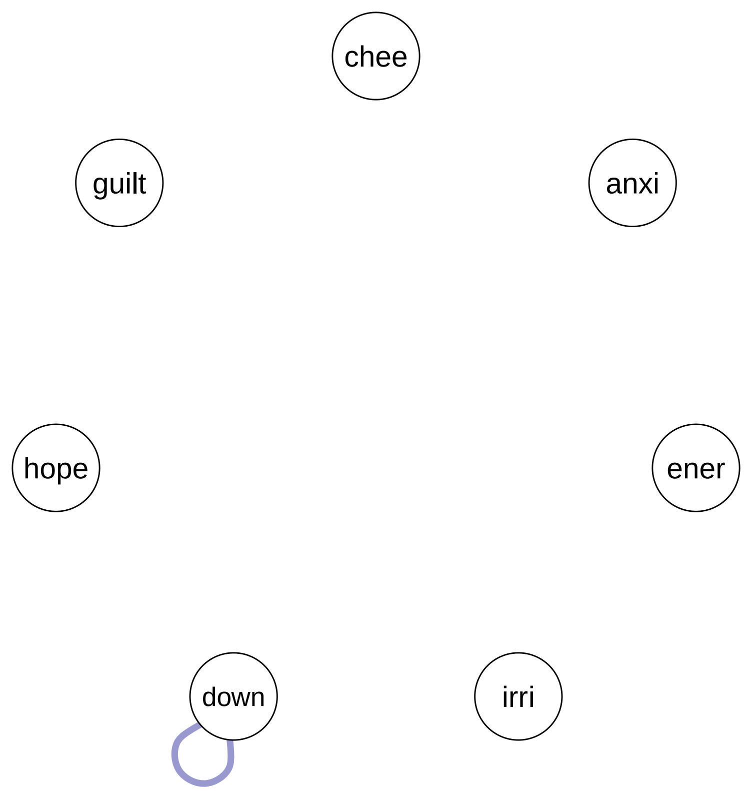

ADM only reg Pt 259 Estpoint 2

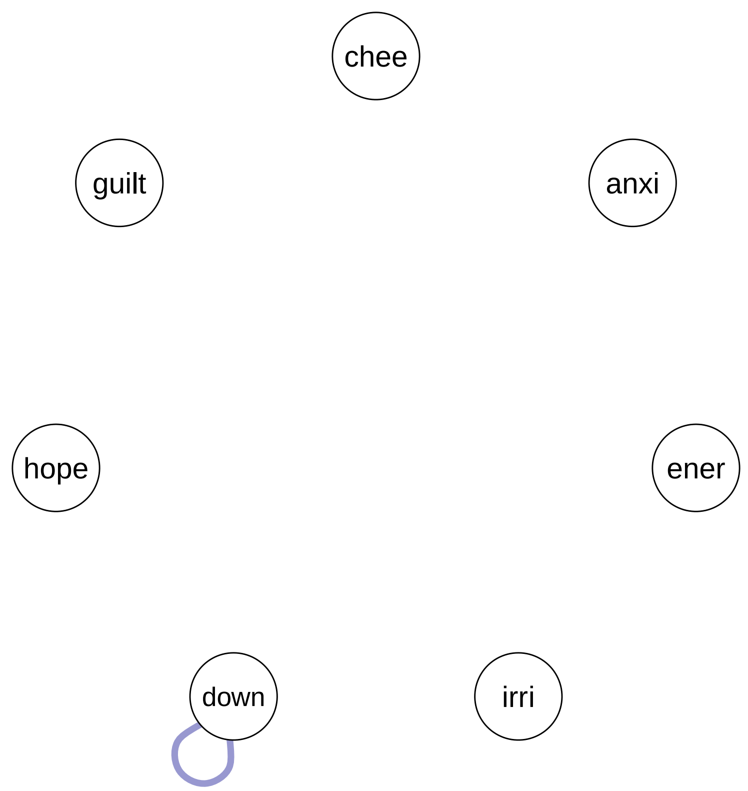

ADM only reg Pt 259 Estpoint 3

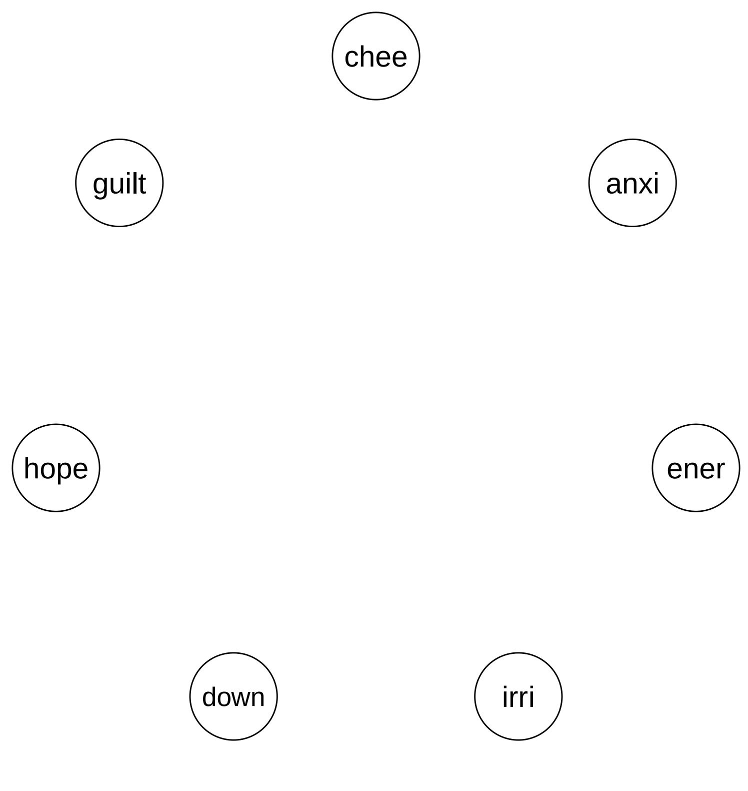

ADM only reg Pt 259 Estpoint 4

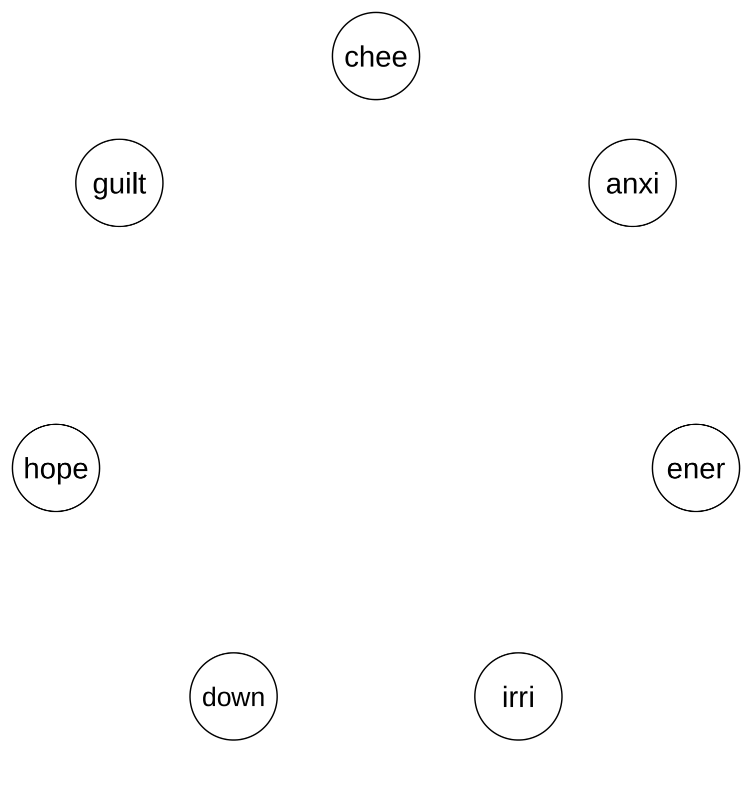

ADM only reg Pt 259 Estpoint 5

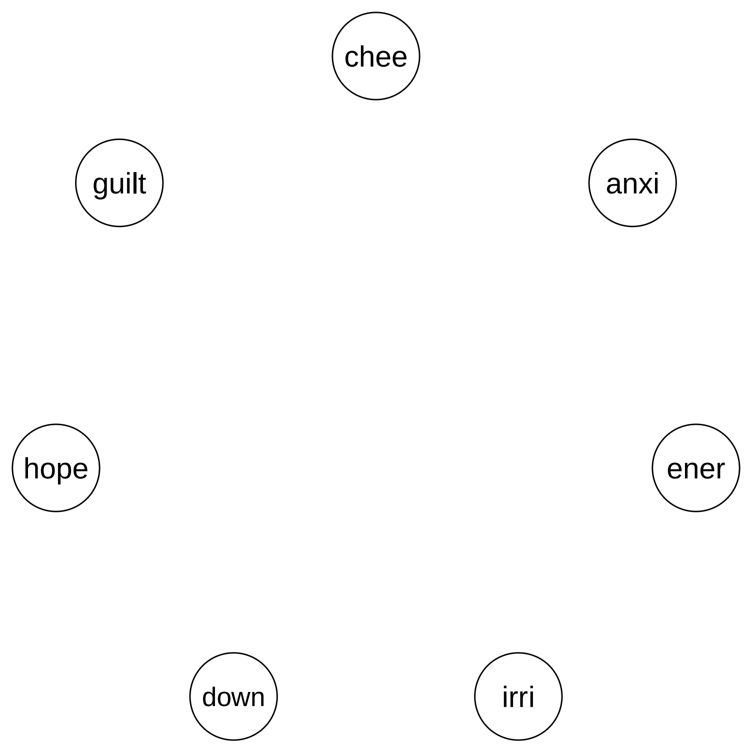

ADM only reg Pt 259 Estpoint 6

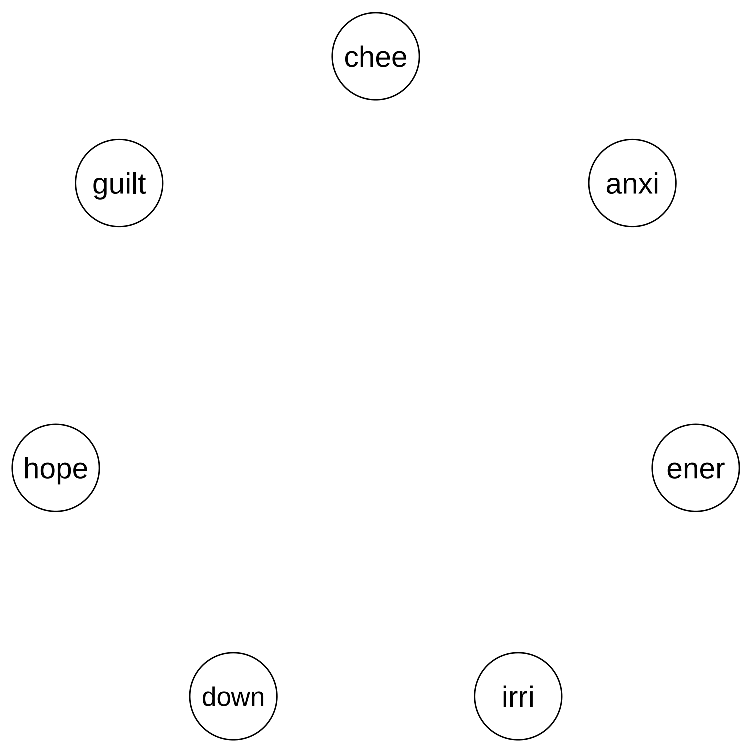

ADM only reg Pt 259 Estpoint 7

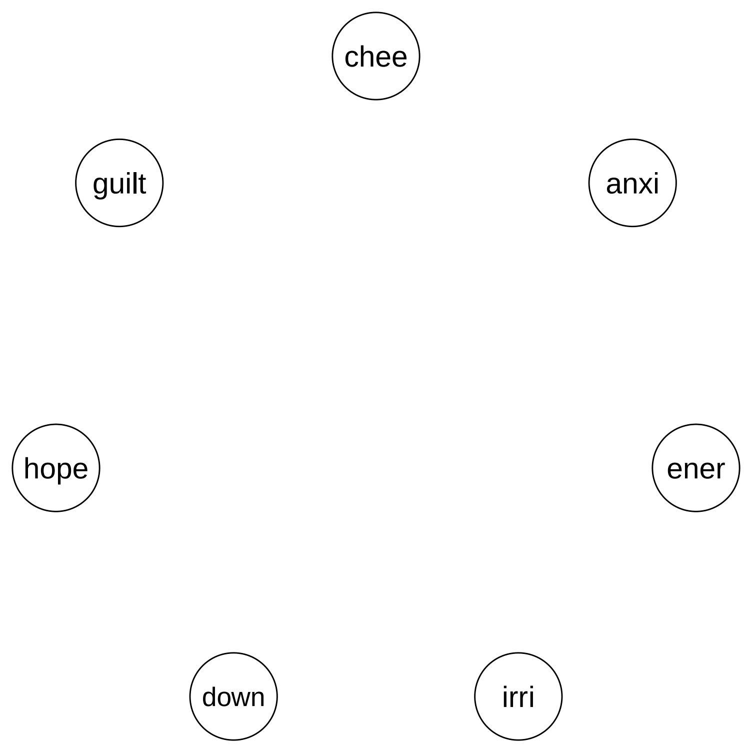

ADM only reg Pt 259 Estpoint 8

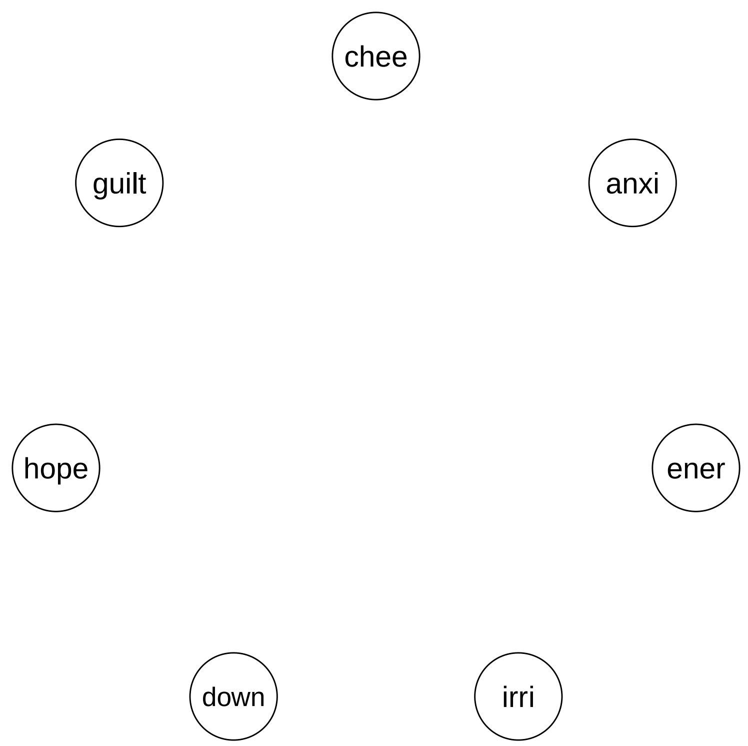

ADM only reg Pt 257 Estpoint 1

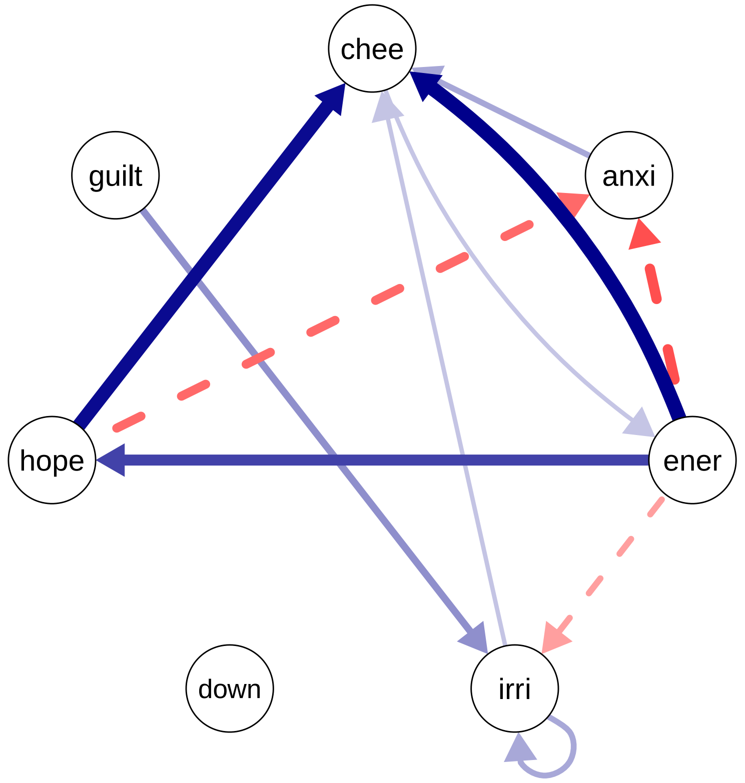

ADM only reg Pt 257 Estpoint 2

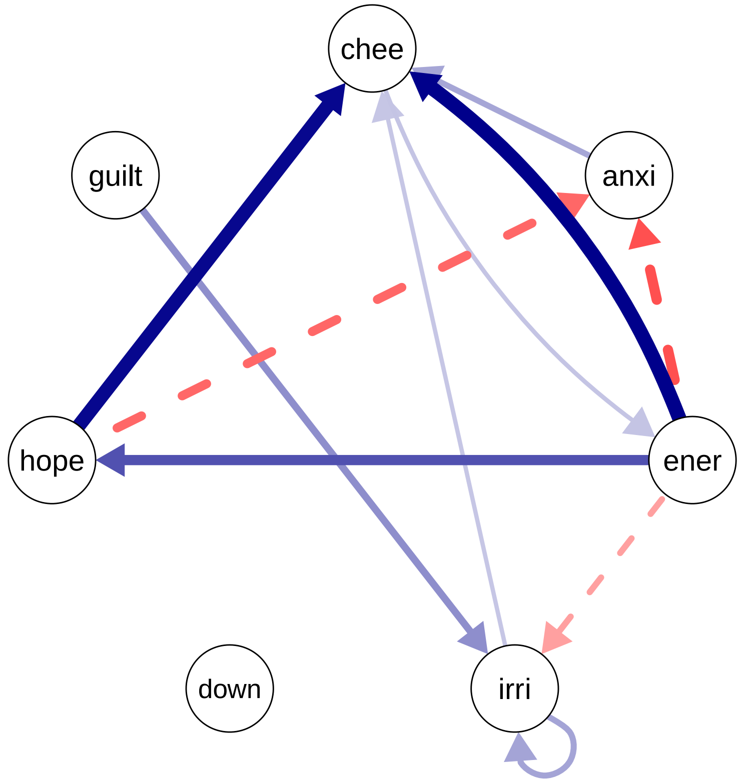

ADM only reg Pt 257 Estpoint 3

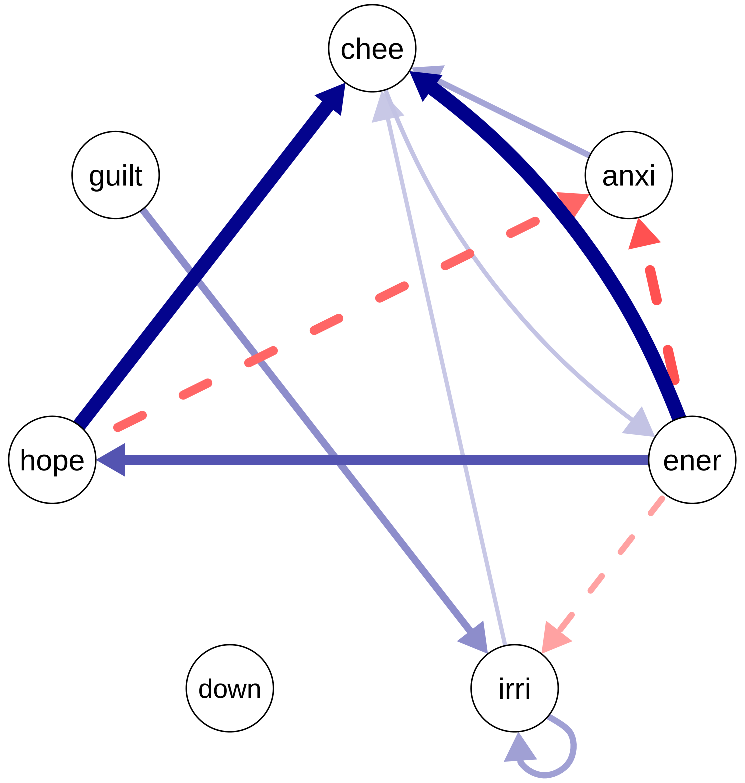

ADM only reg Pt 257 Estpoint 4

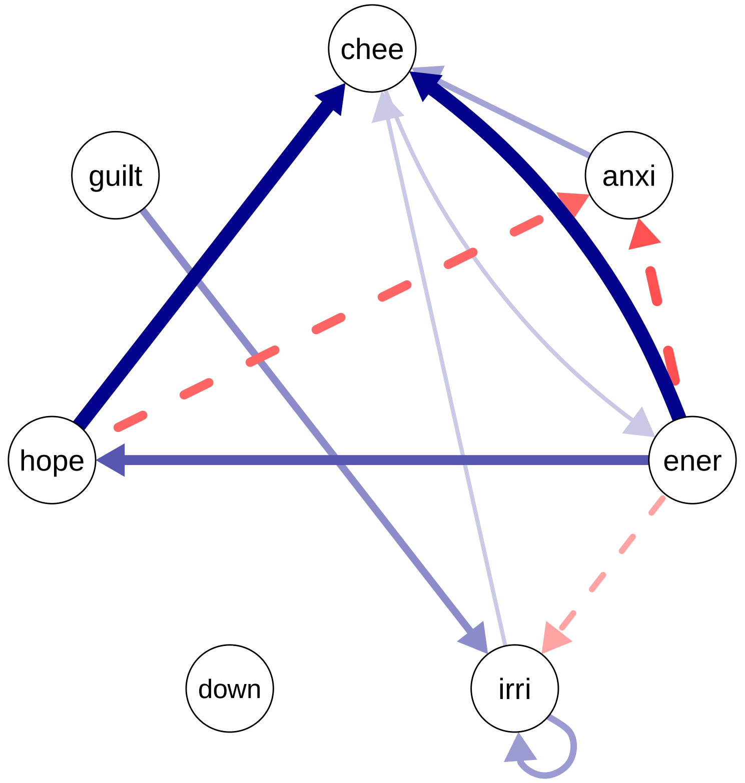

ADM only reg Pt 257 Estpoint 5

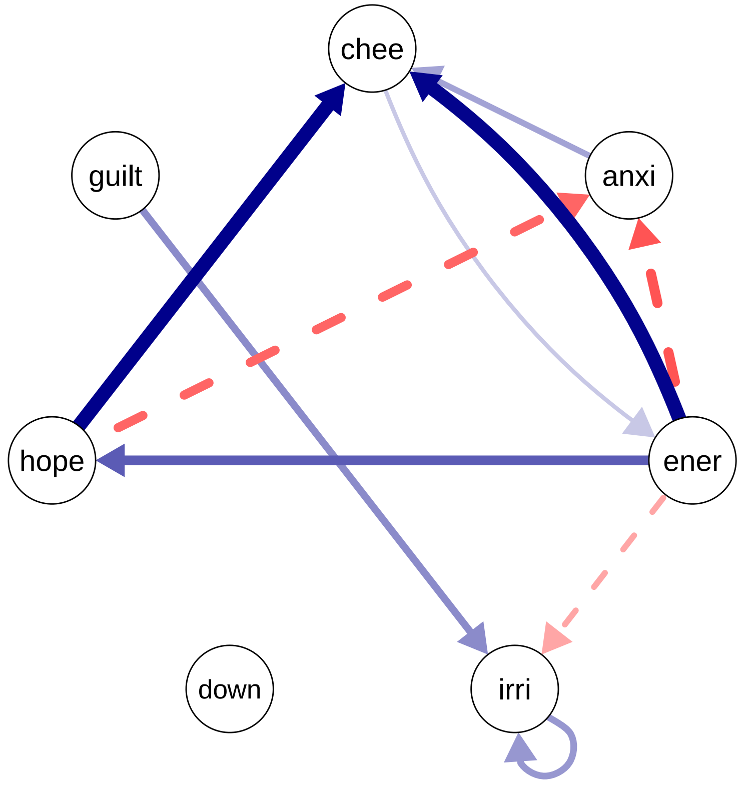

ADM only reg Pt 257 Estpoint 6

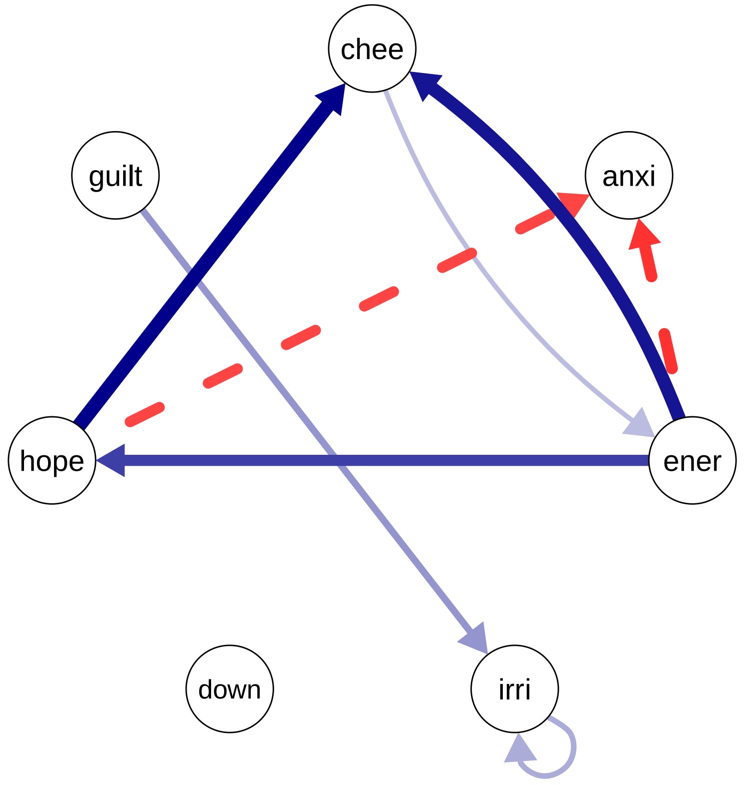

ADM only reg Pt 257 Estpoint 7

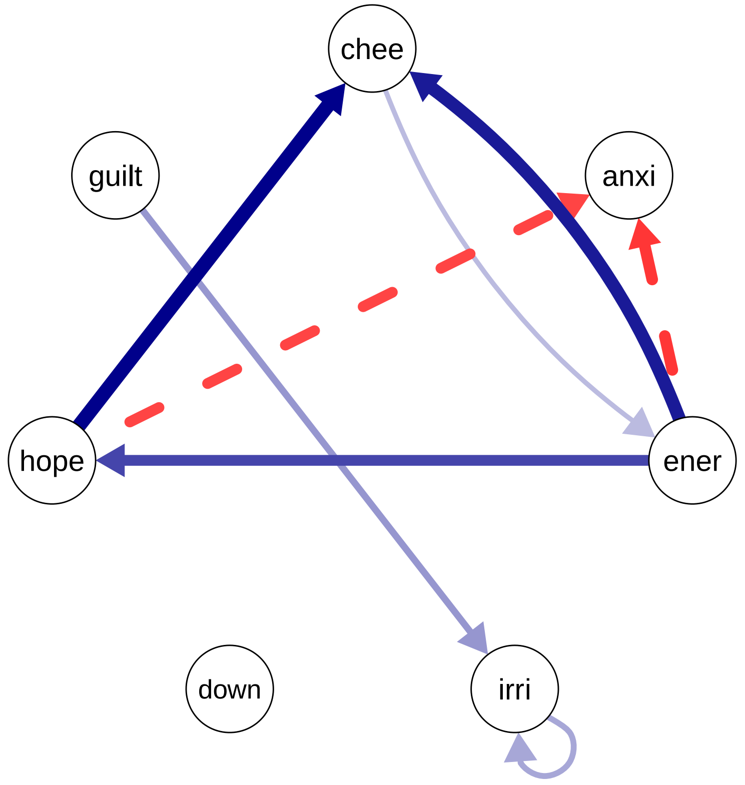

ADM only reg Pt 257 Estpoint 8

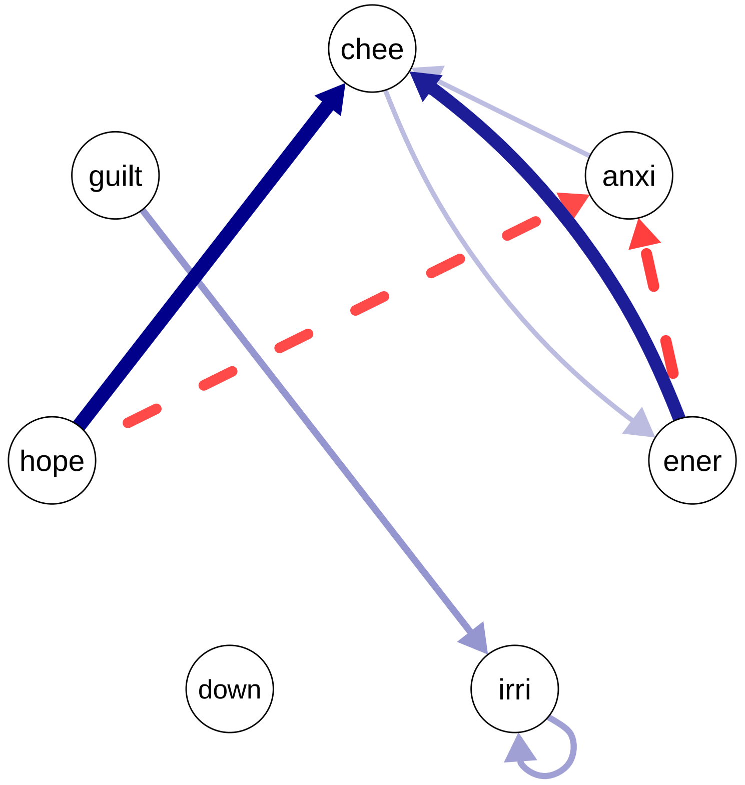

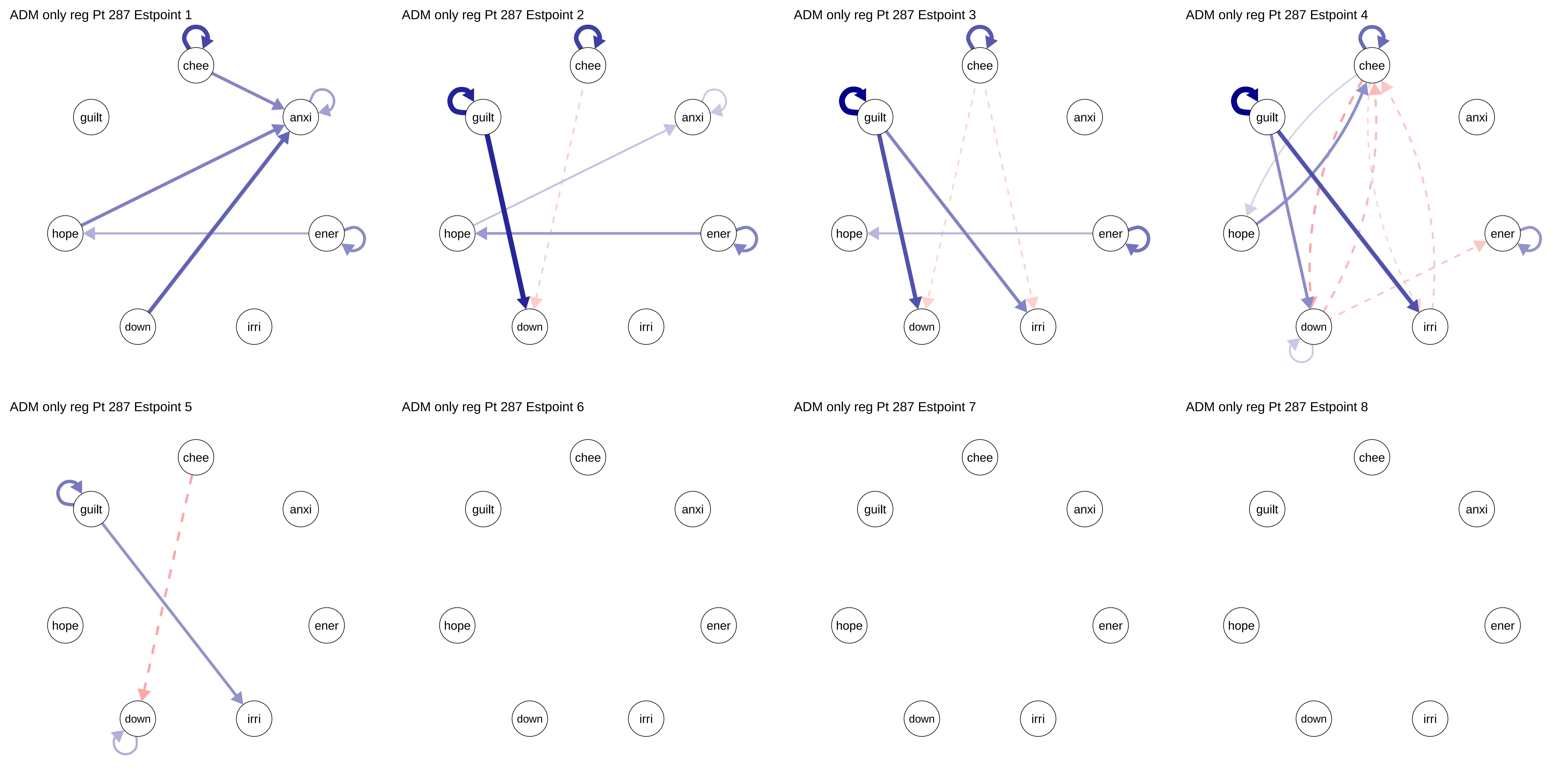
ADM only reg Pt 287 Estpoint 1
chee
guilt
anxi
hope
ener
down
irri
ADM only reg Pt 287 Estpoint 2
chee
guilt
anxi
hope
ener
down
irri
ADM only reg Pt 287 Estpoint 3
chee
guilt
anxi
hope
ener
down
irri
ADM only reg Pt 287 Estpoint 4
chee
guilt
anxi
hope
ener
down
irri
ADM only reg Pt 287 Estpoint 5
chee
guilt
anxi
hope
ener
down
irri
ADM only reg Pt 287 Estpoint 6
chee
guilt
anxi
hope
ener
down
irri
ADM only reg Pt 287 Estpoint 7
chee
guilt
anxi
hope
ener
down
irri
ADM only reg Pt 287 Estpoint 8
chee
guilt
anxi
hope
ener
down
irri

ADM only reg Pt 233 Estpoint 1

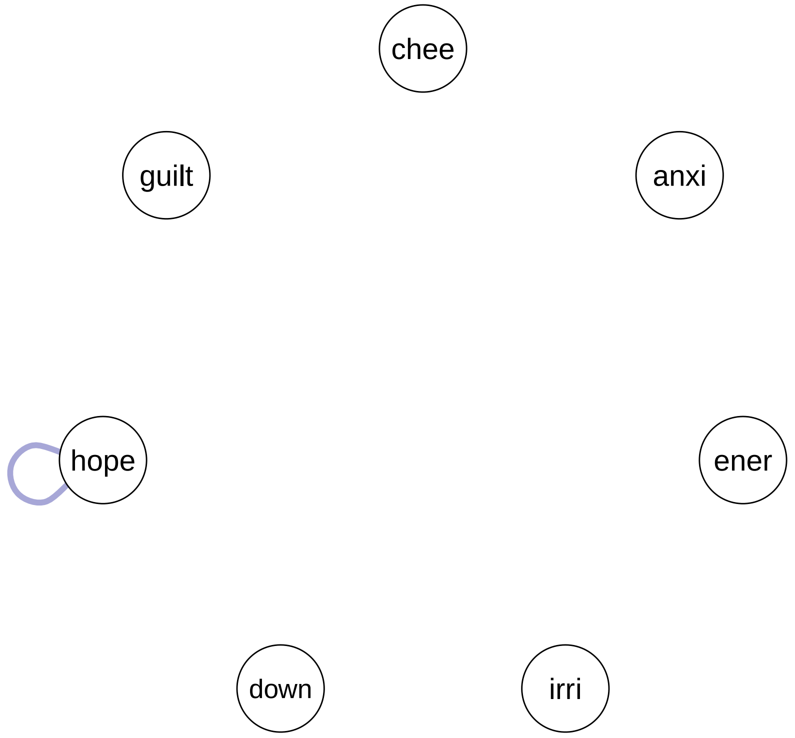

ADM only reg Pt 233 Estpoint 2

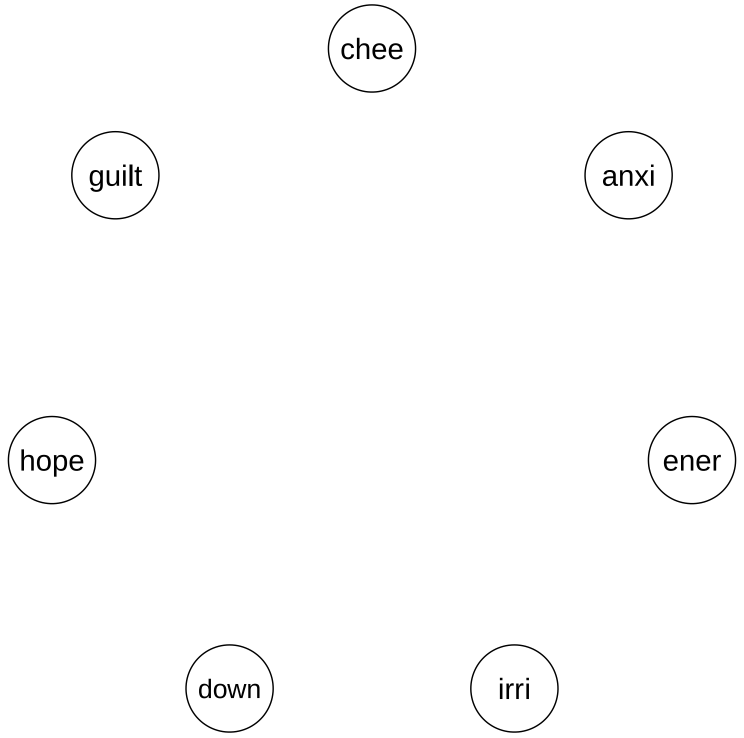

ADM only reg Pt 233 Estpoint 3

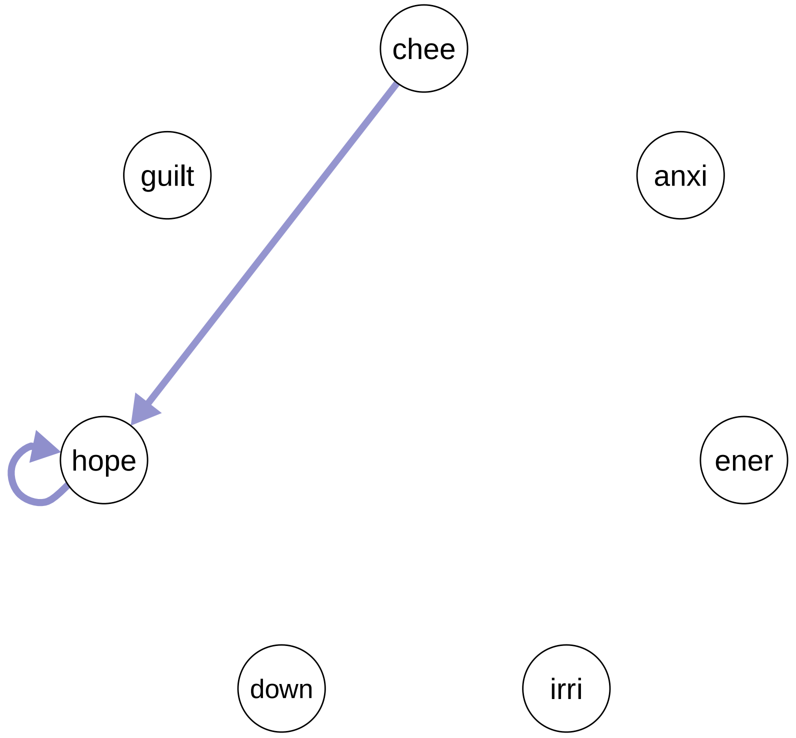

ADM only reg Pt 233 Estpoint 4

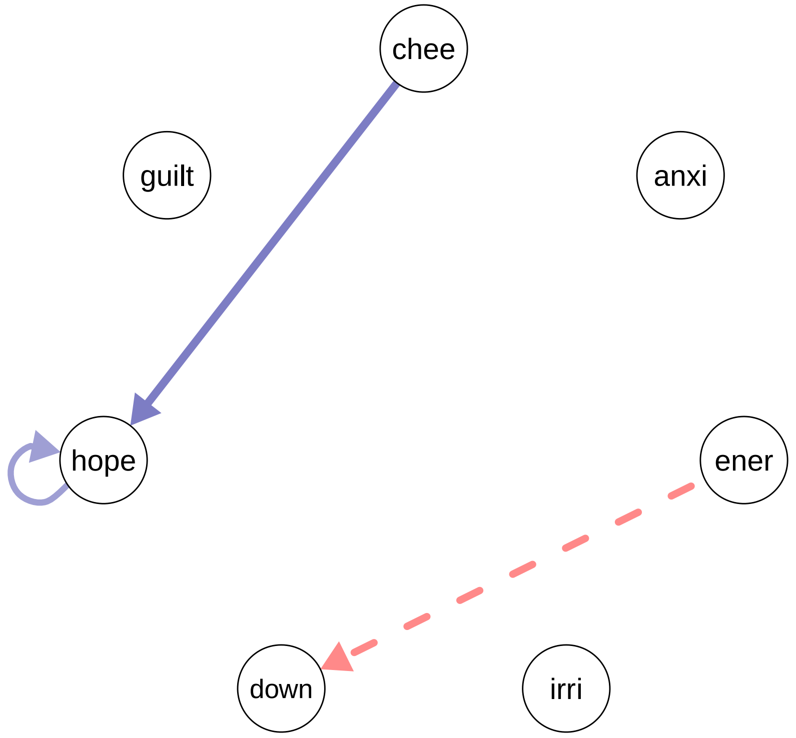

ADM only reg Pt 233 Estpoint 5

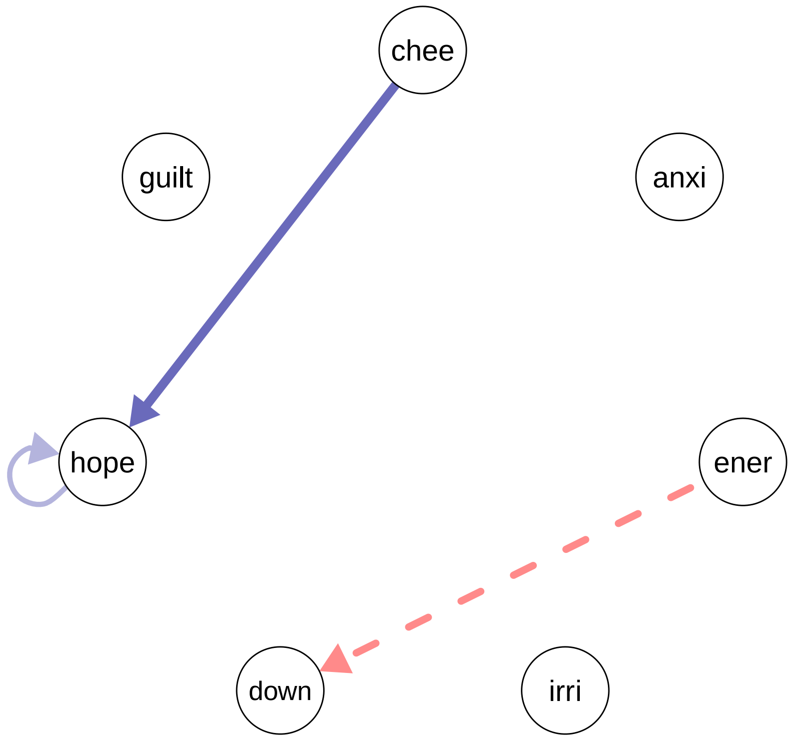

ADM only reg Pt 233 Estpoint 6

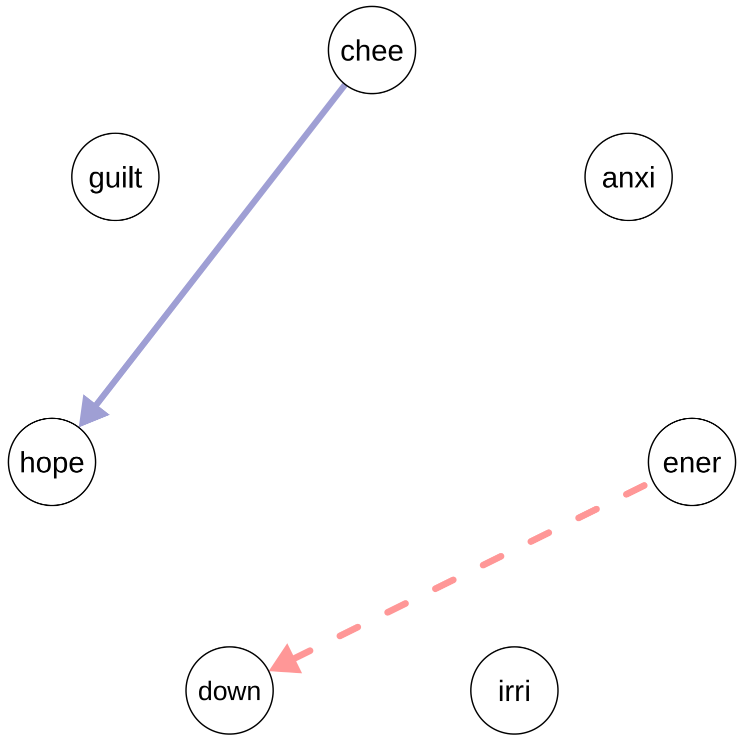

ADM only reg Pt 233 Estpoint 7

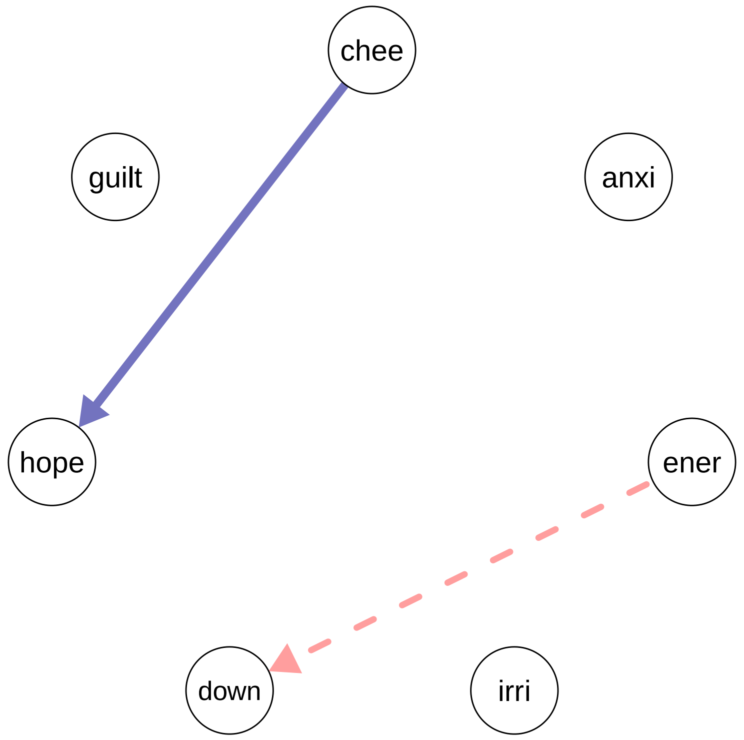

ADM only reg Pt 233 Estpoint 8

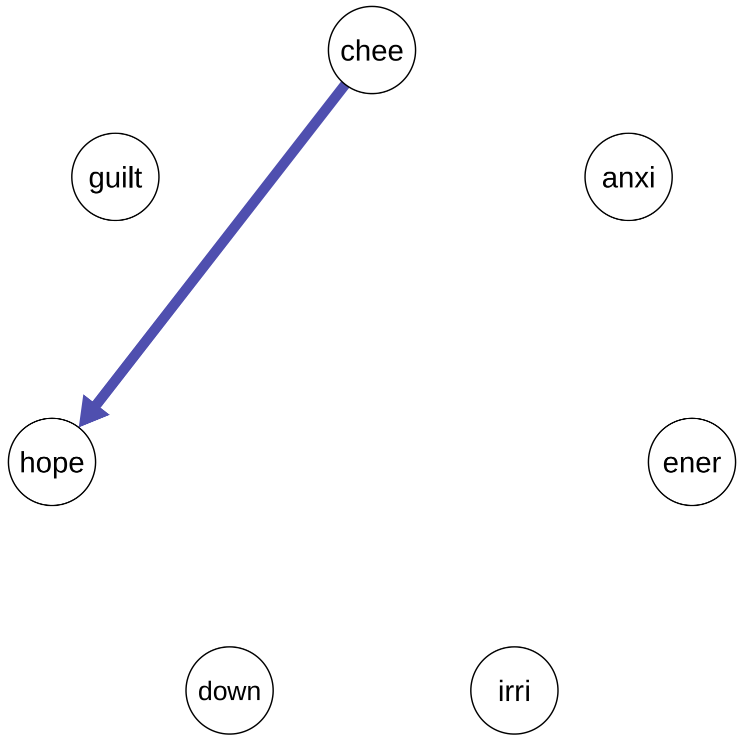

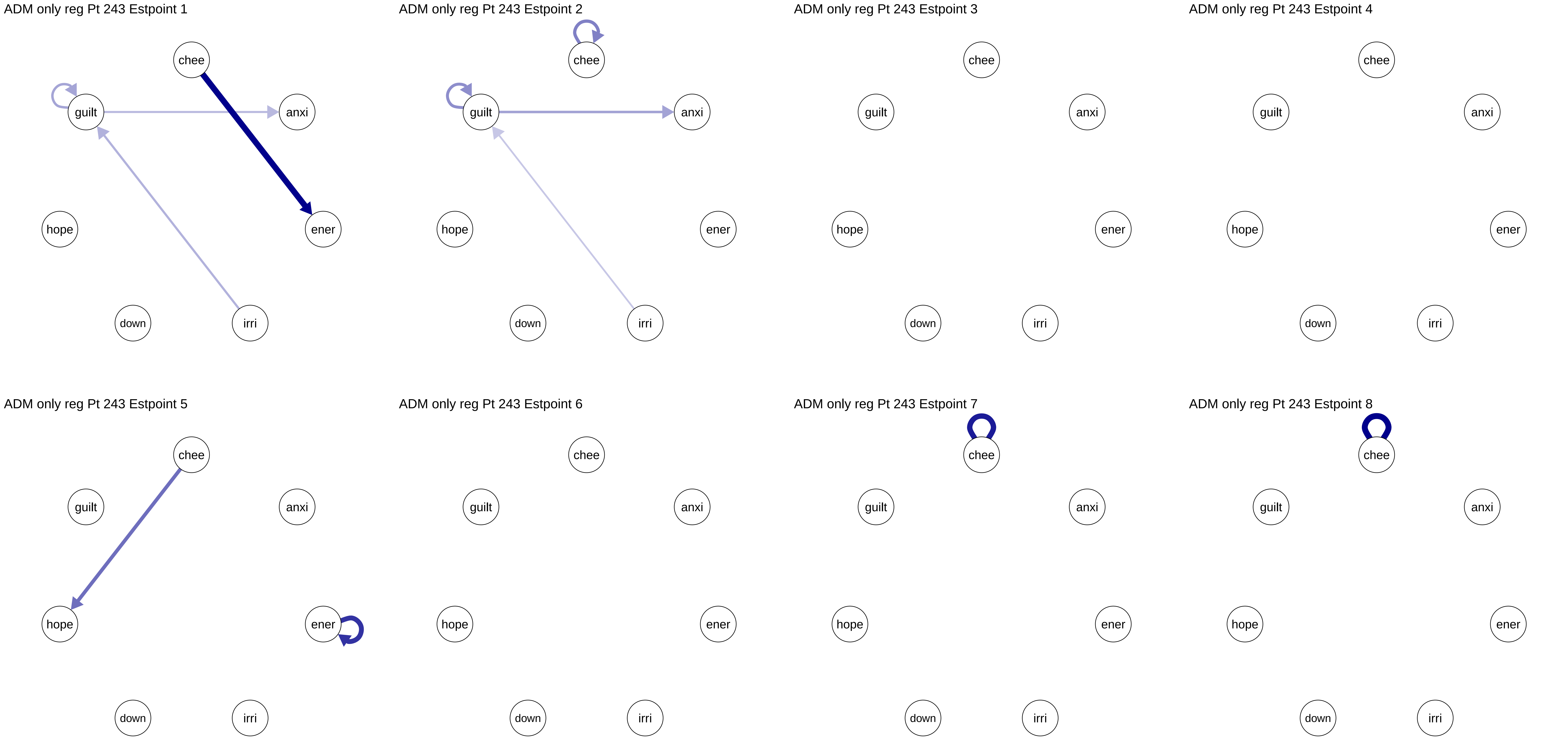
ADM only reg Pt 243 Estpoint 1
chee
guilt
anxi
hope
ener
down
irri
ADM only reg Pt 243 Estpoint 2
chee
guilt
anxi
hope
ener
down
irri
ADM only reg Pt 243 Estpoint 3
chee
guilt
anxi
hope
ener
down
irri
ADM only reg Pt 243 Estpoint 4
chee
guilt
anxi
hope
ener
down
irri
ADM only reg Pt 243 Estpoint 5
chee
guilt
anxi
hope
ener
down
irri
ADM only reg Pt 243 Estpoint 6
chee
guilt
anxi
hope
ener
down
irri
ADM only reg Pt 243 Estpoint 7
chee
guilt
anxi
hope
ener
down
irri
ADM only reg Pt 243 Estpoint 8
chee
guilt
anxi
hope
ener
down
irri

ADM only reg Pt 239 Estpoint 1

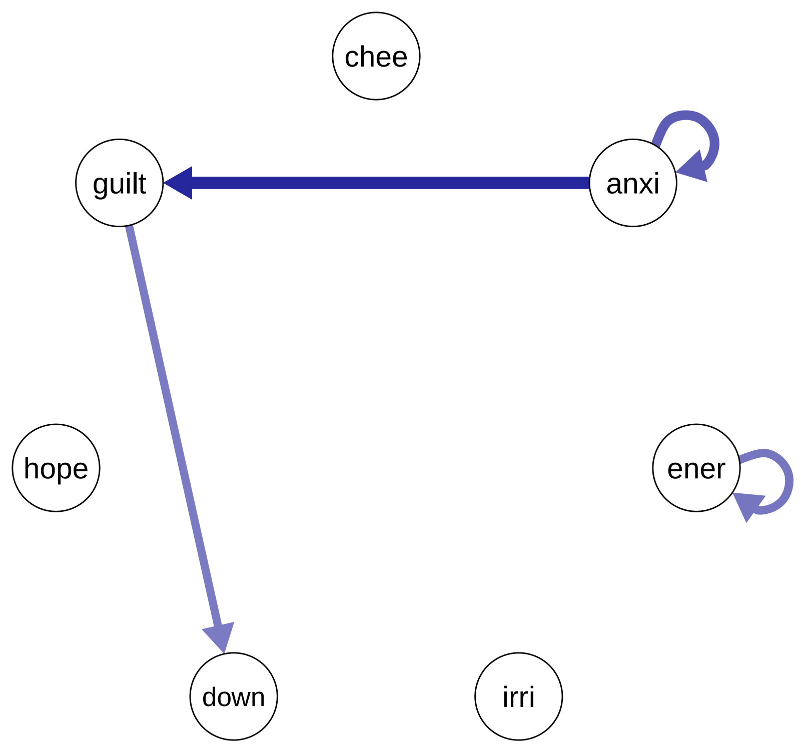

ADM only reg Pt 239 Estpoint 2

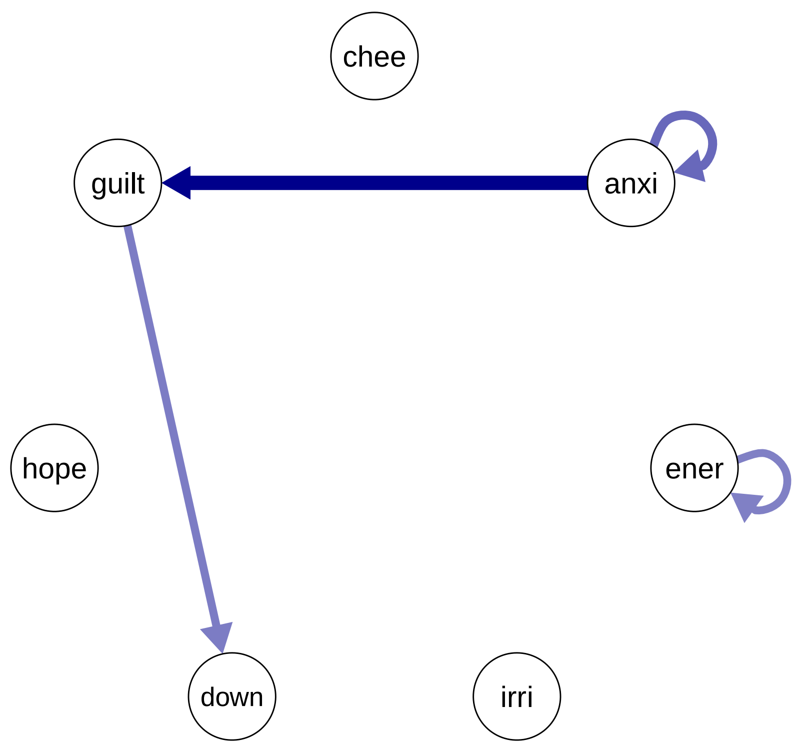

ADM only reg Pt 239 Estpoint 3

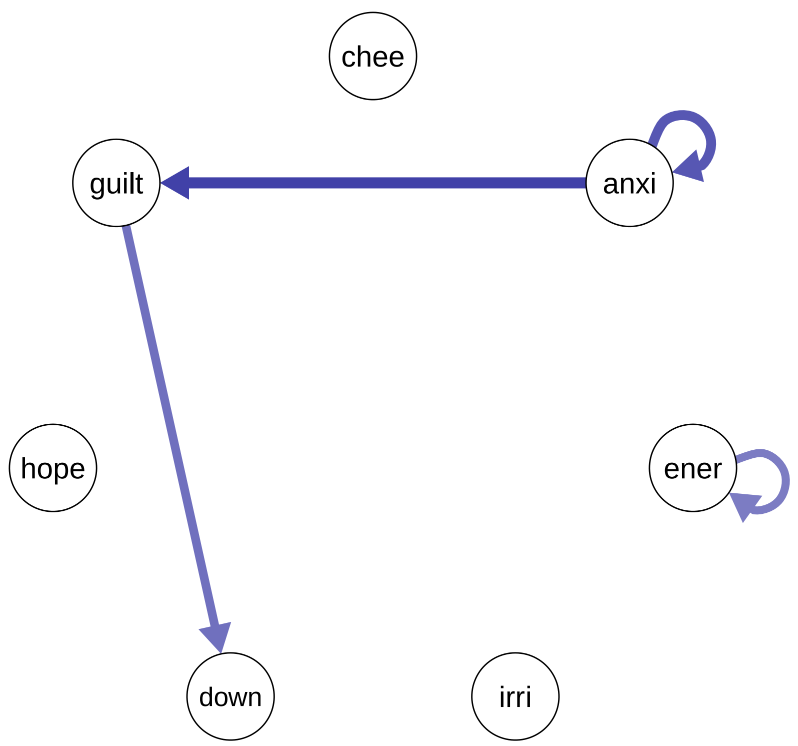

ADM only reg Pt 239 Estpoint 4

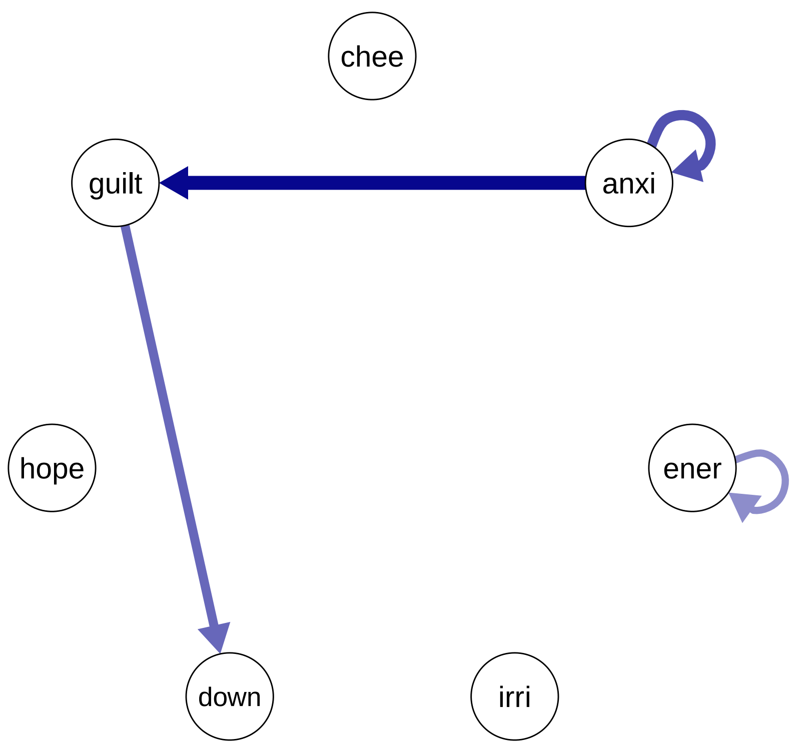

ADM only reg Pt 239 Estpoint 5

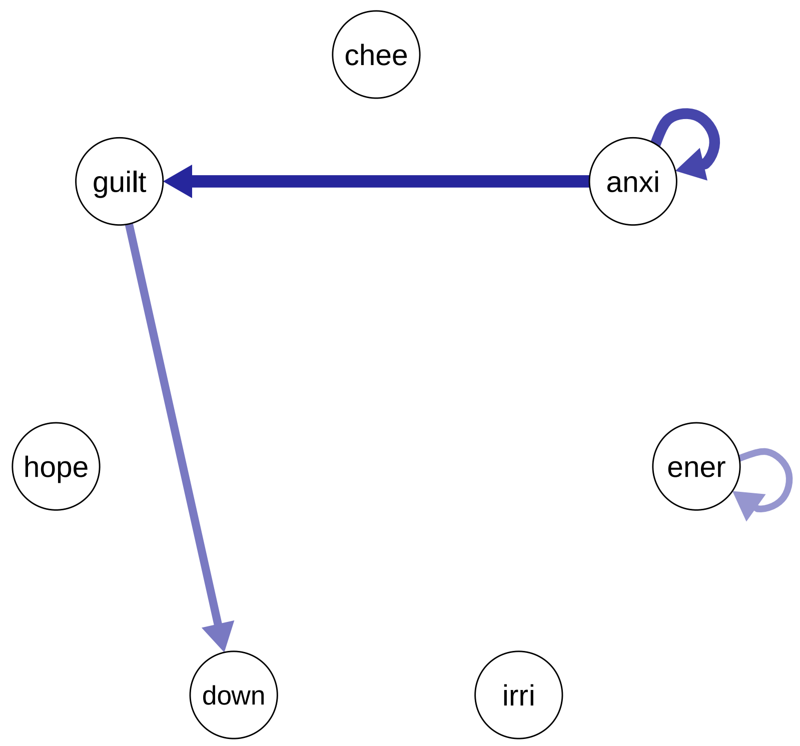

ADM only reg Pt 239 Estpoint 6

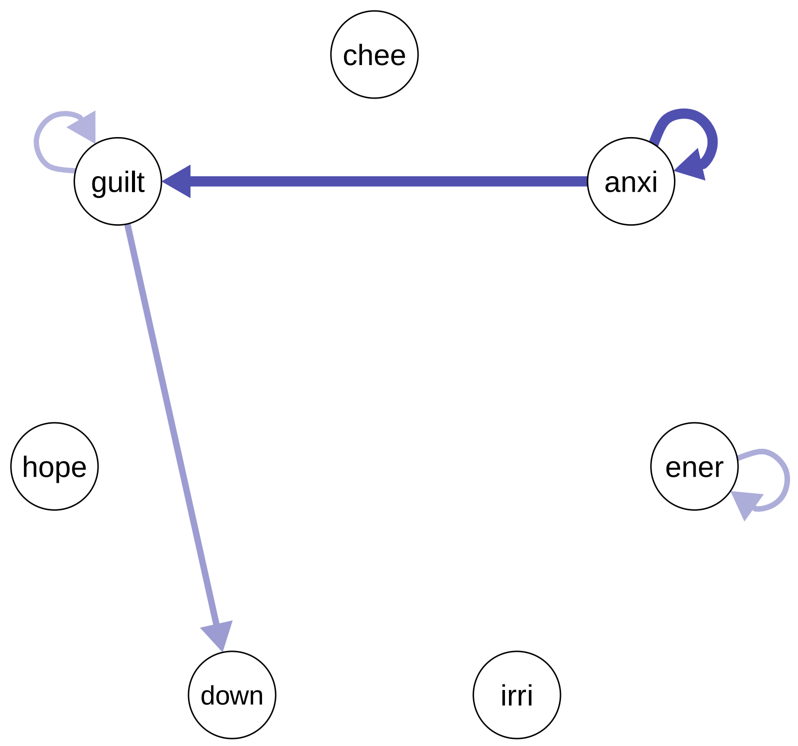

ADM only reg Pt 239 Estpoint 7

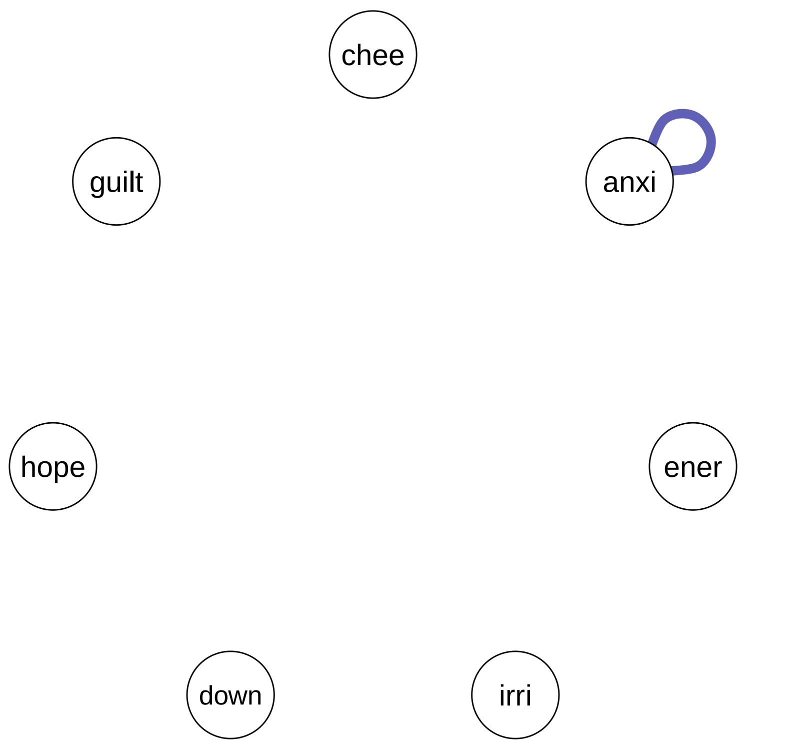

ADM only reg Pt 239 Estpoint 8

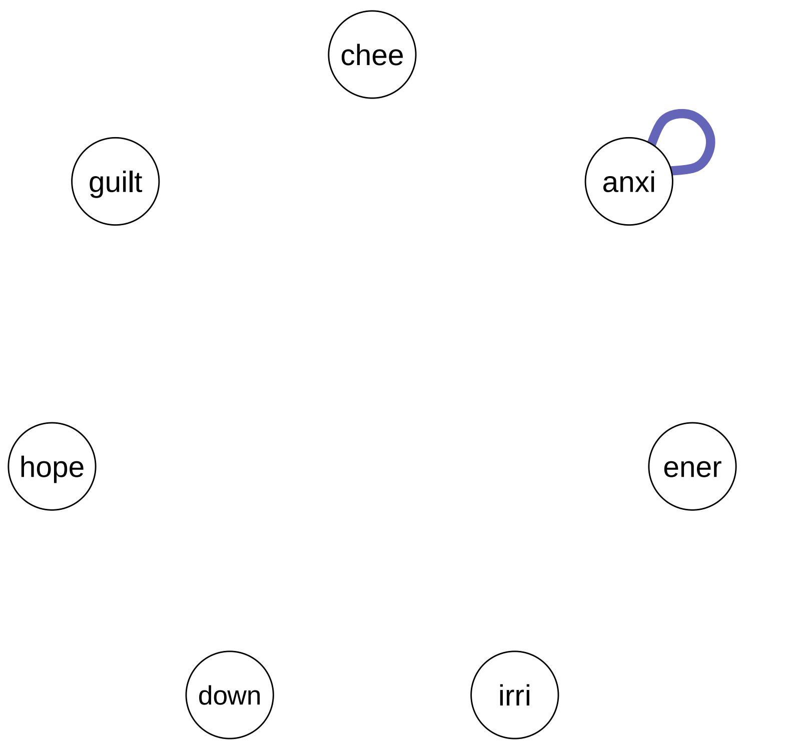

ADM only reg Pt 265 Estpoint 1

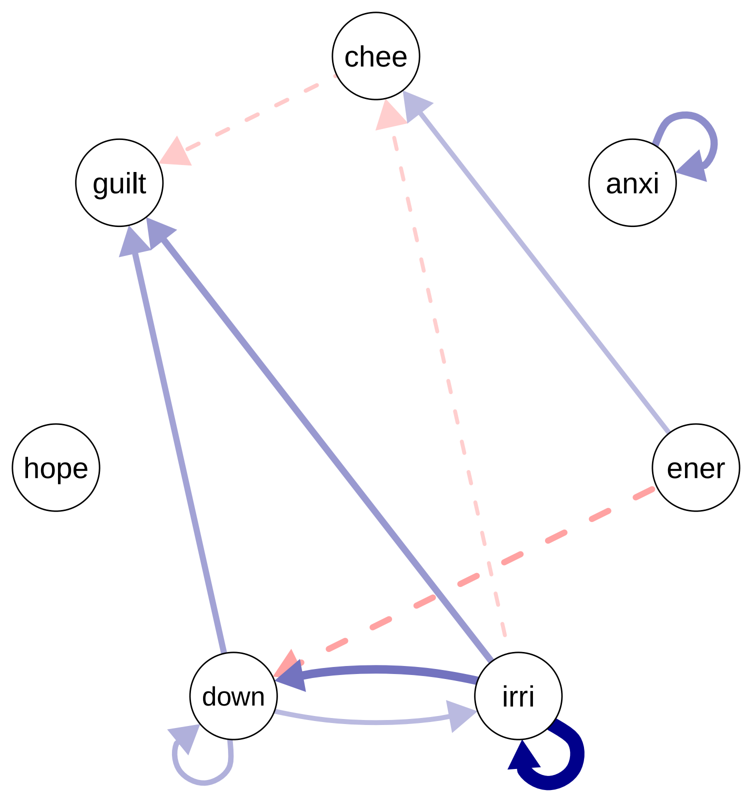

ADM only reg Pt 265 Estpoint 2

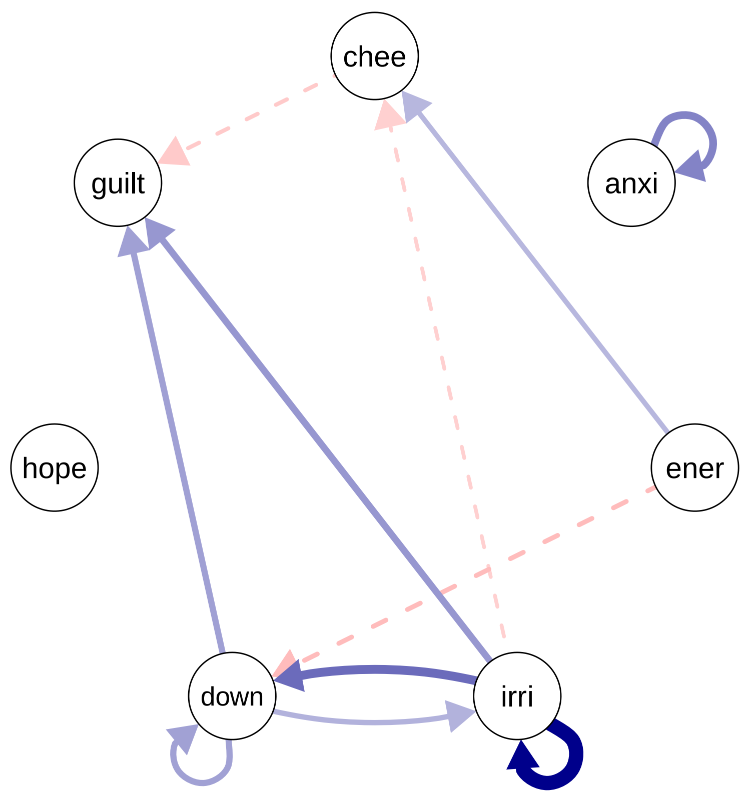

ADM only reg Pt 265 Estpoint 3

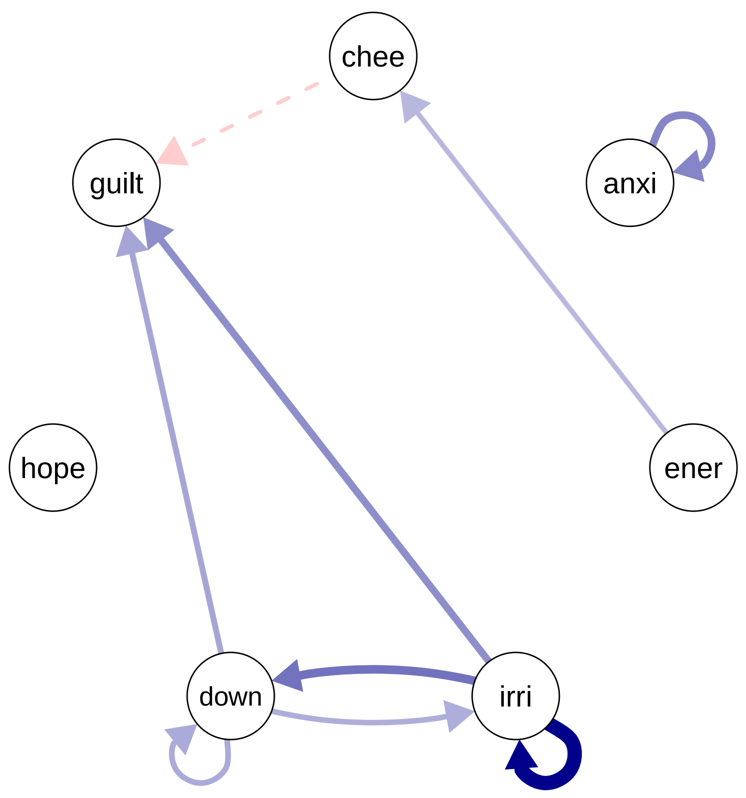

ADM only reg Pt 265 Estpoint 4

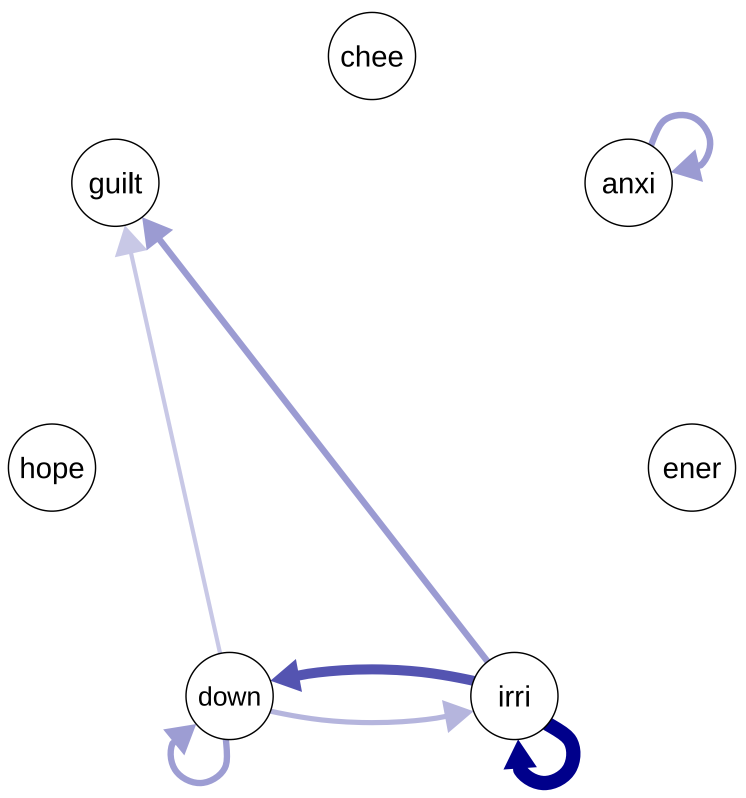

ADM only reg Pt 265 Estpoint 5

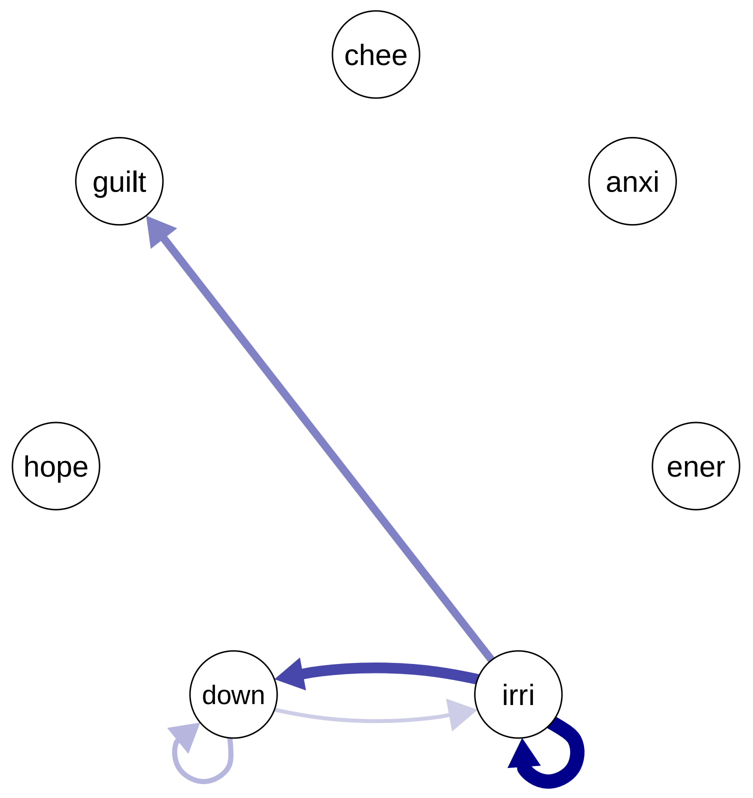

ADM only reg Pt 265 Estpoint 6

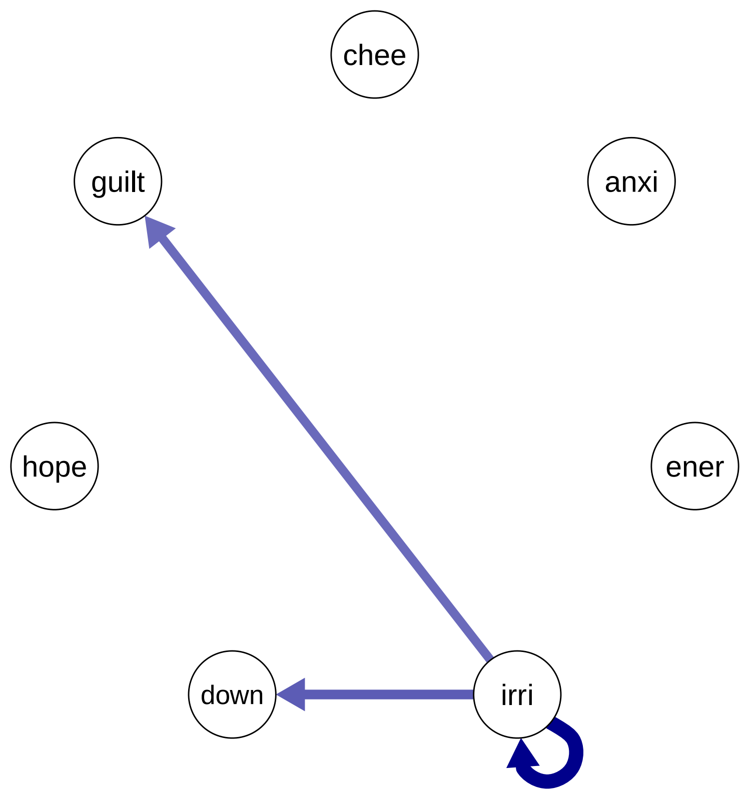

ADM only reg Pt 265 Estpoint 7

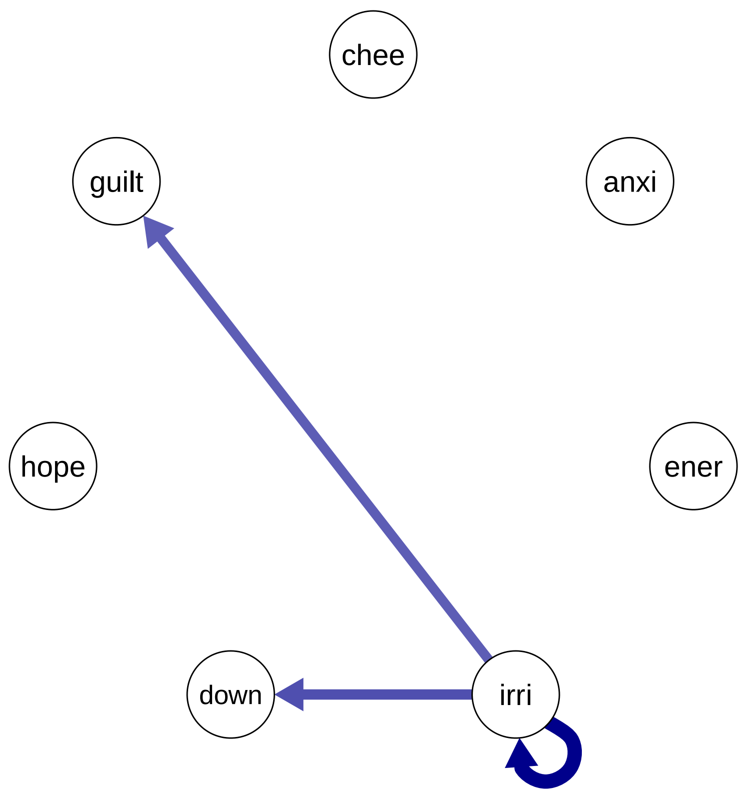

ADM only reg Pt 265 Estpoint 8

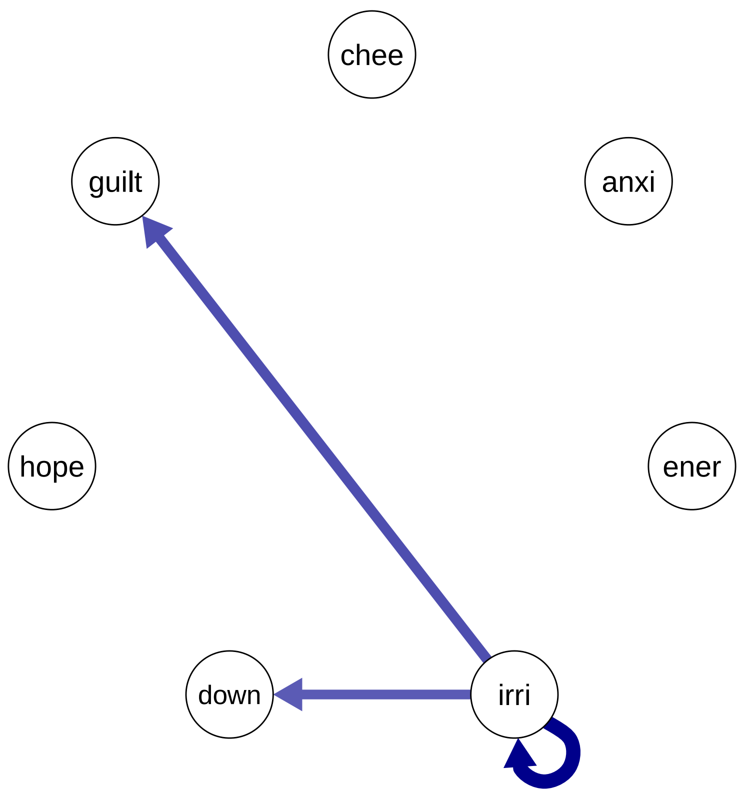

Healthy control non−reg 88MD Estpoint 1

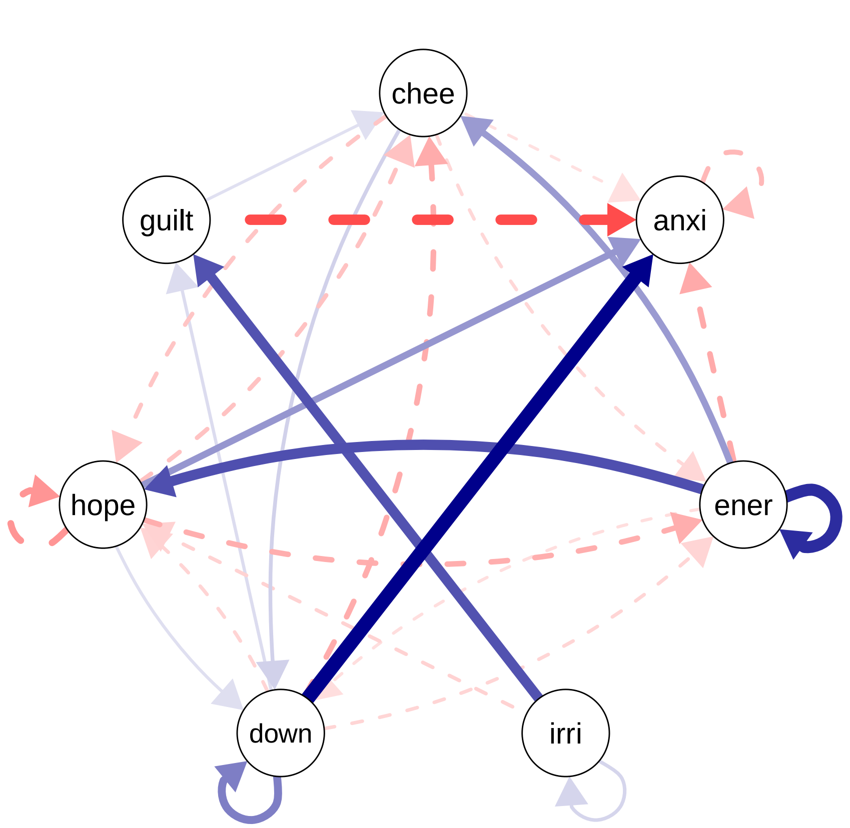

Healthy control non−reg 88MD Estpoint 2

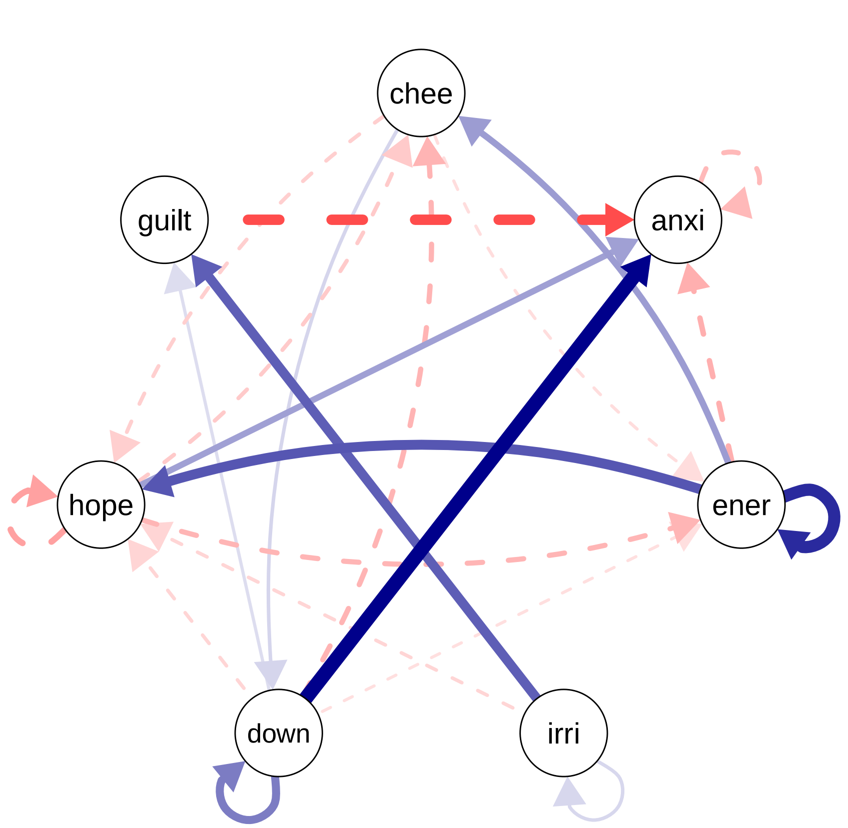

Healthy control non−reg 88MD Estpoint 3

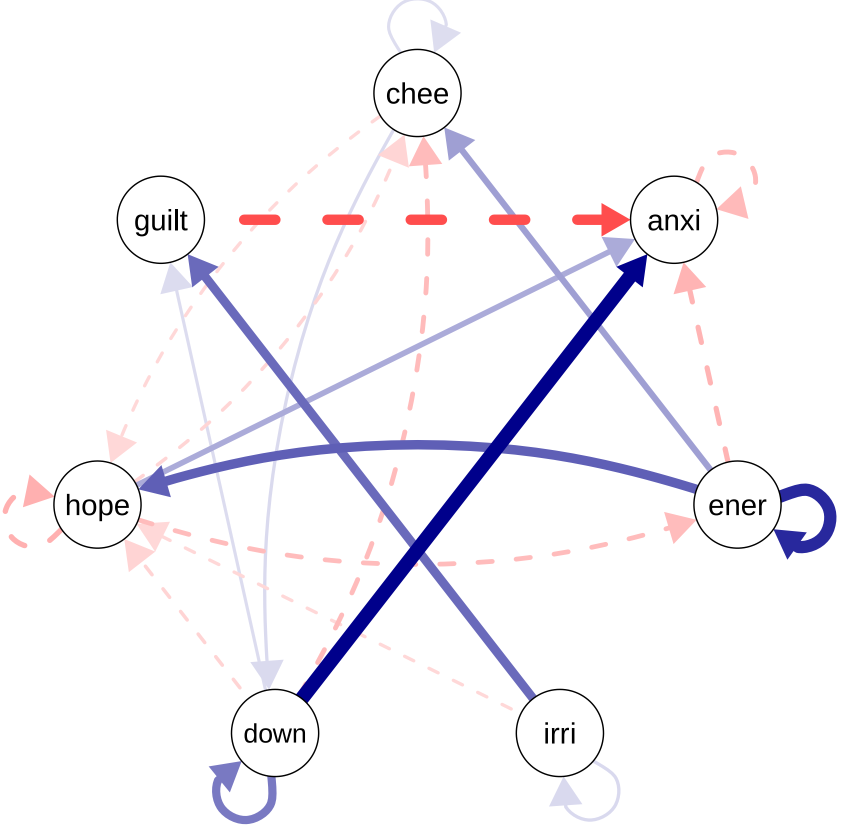

Healthy control non−reg 88MD Estpoint 4

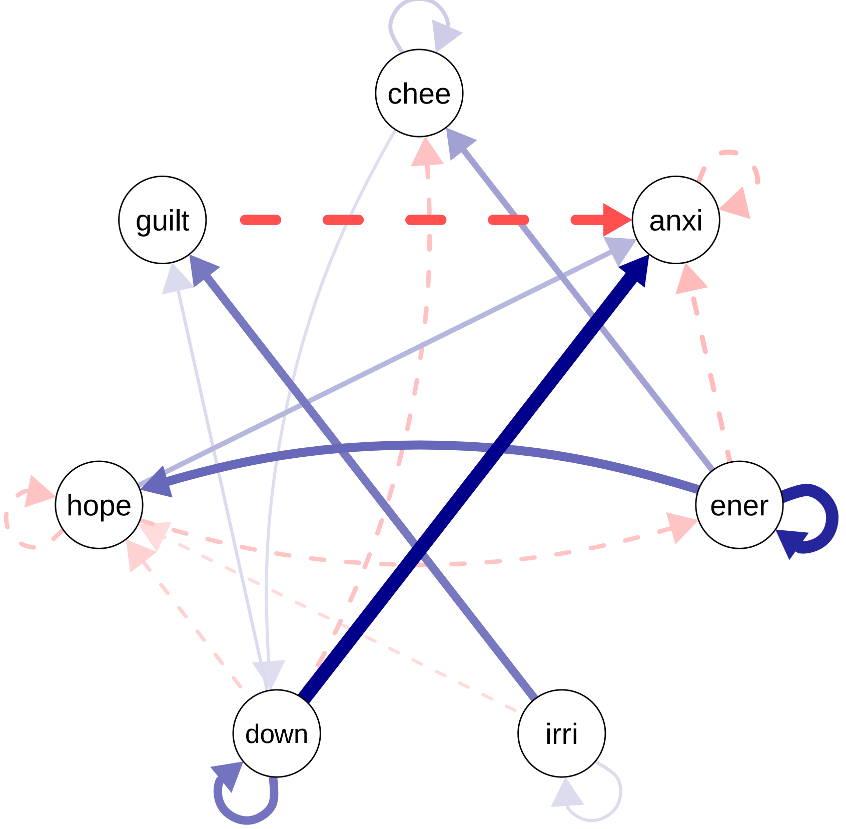

Healthy control non−reg 88MD Estpoint 5

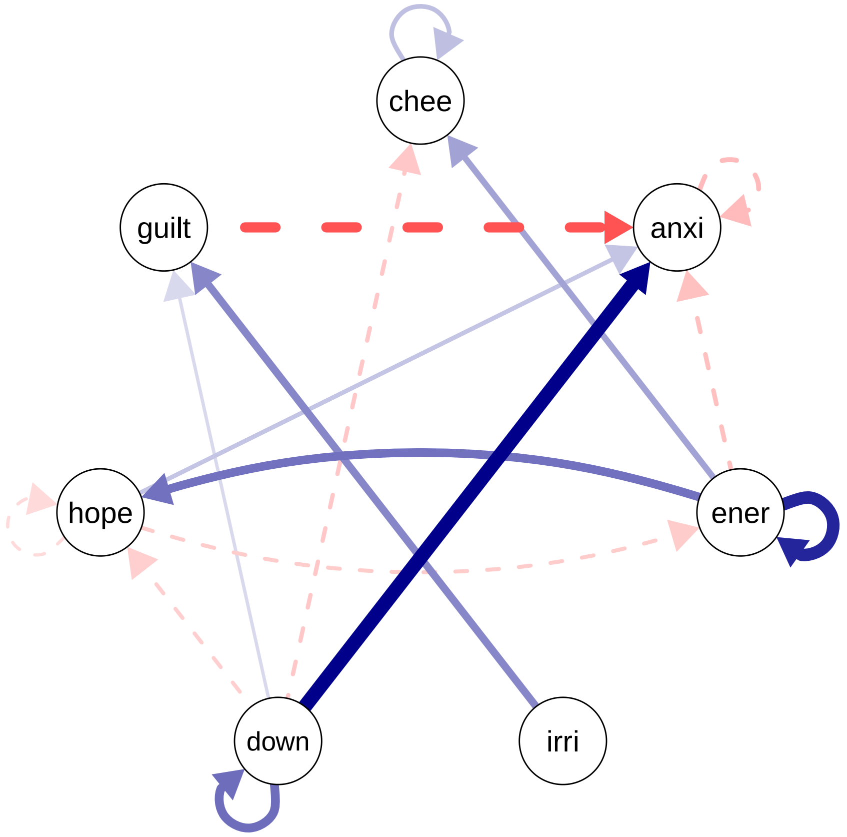

Healthy control non−reg 88MD Estpoint 6

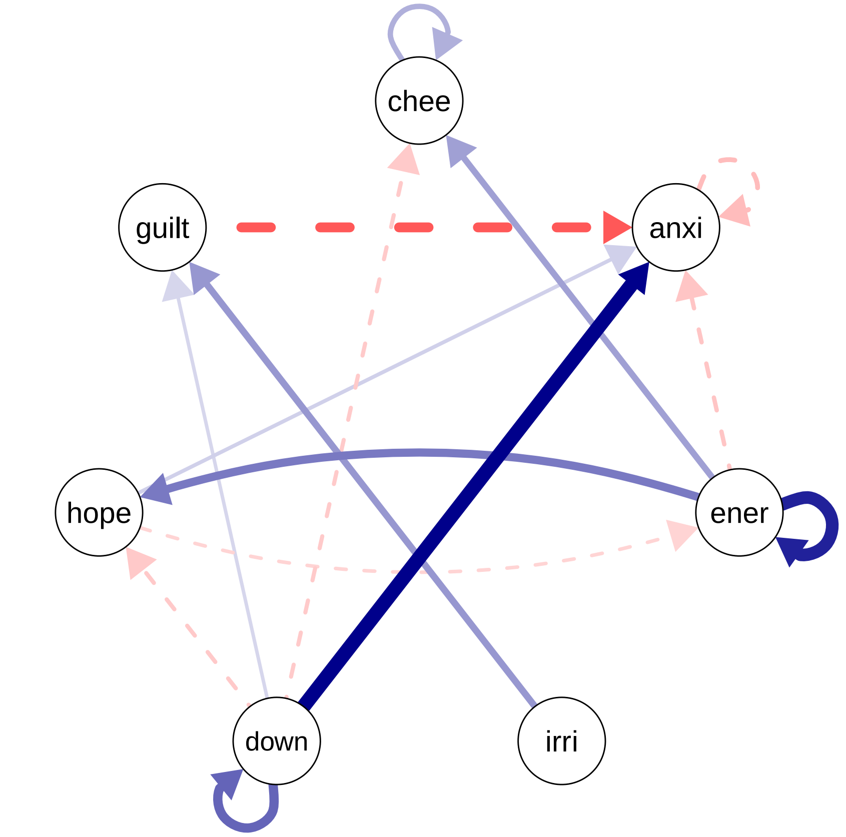

Healthy control non−reg 88MD Estpoint 7

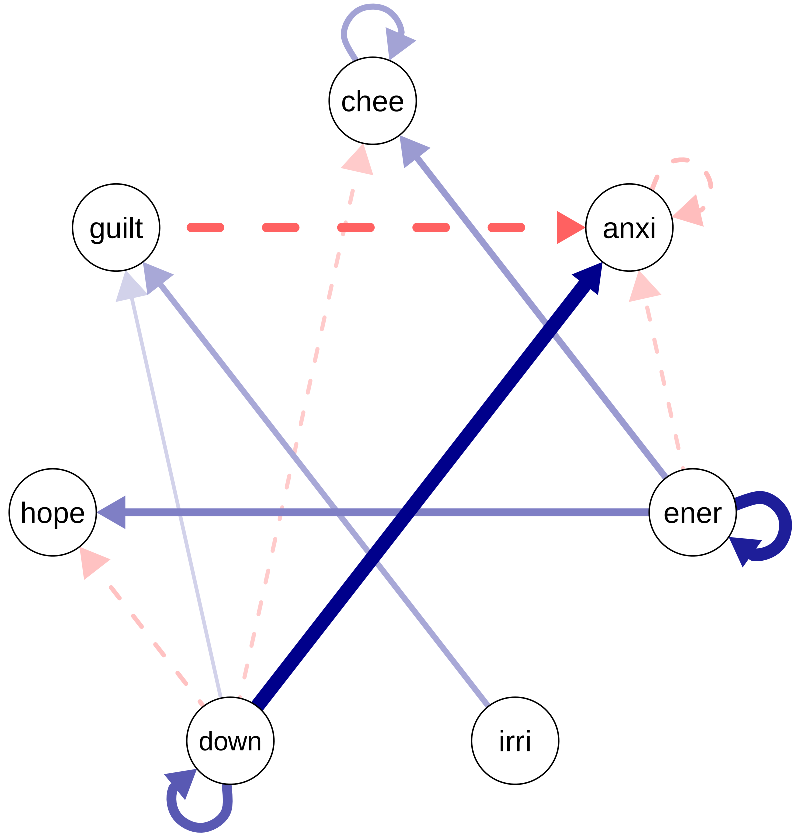

Healthy control non−reg 88MD Estpoint 8

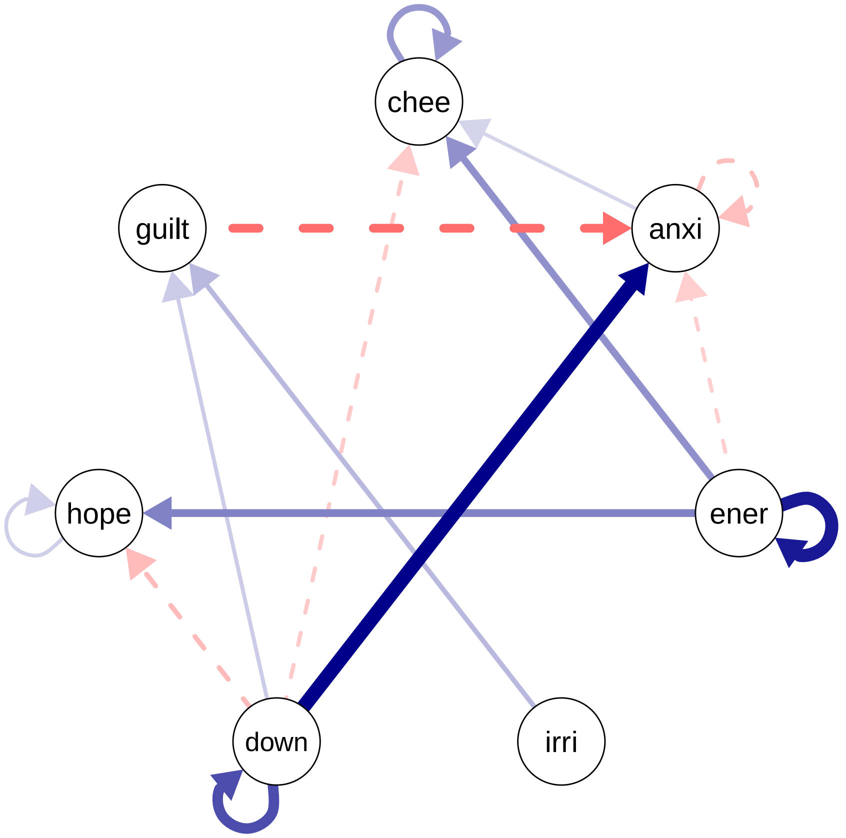

Healthy control non−reg 88JZ Estpoint 1

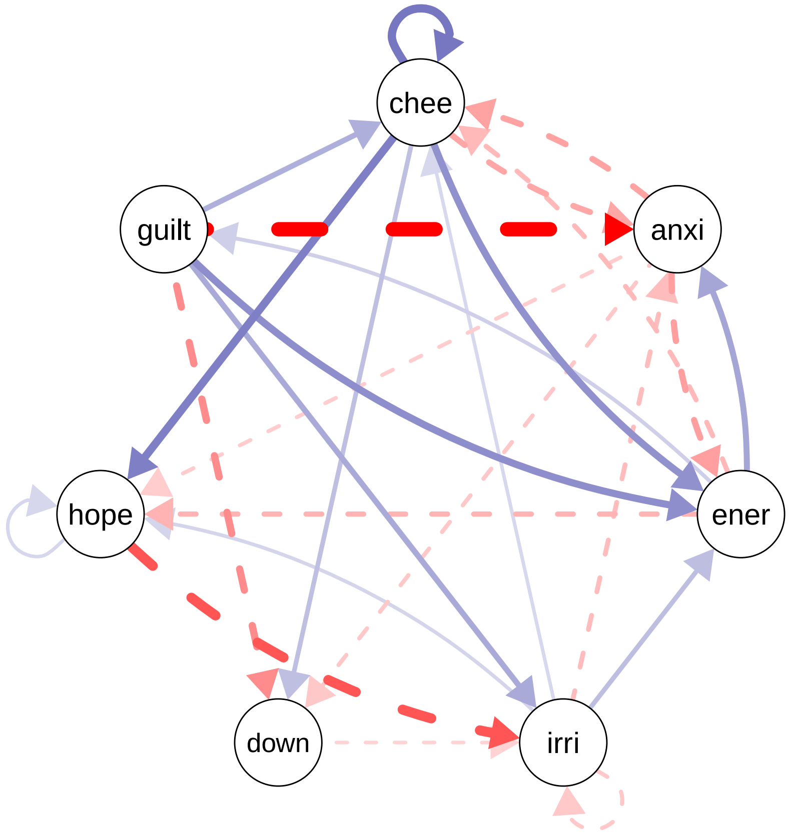

Healthy control non−reg 88JZ Estpoint 2

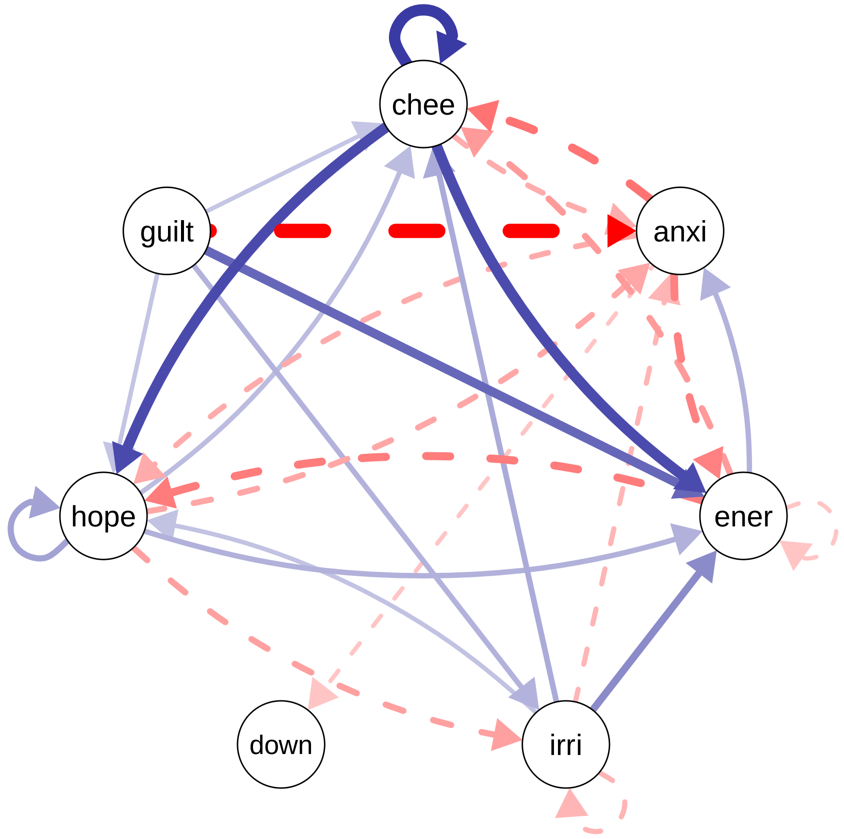

Healthy control non−reg 88JZ Estpoint 3

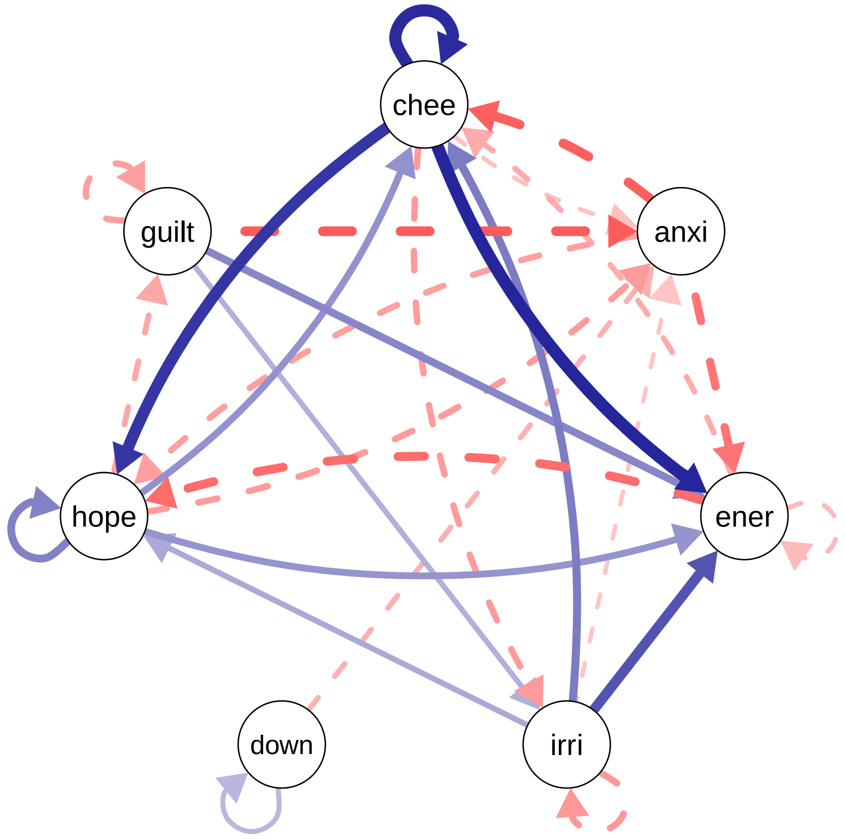

Healthy control non−reg 88JZ Estpoint 4

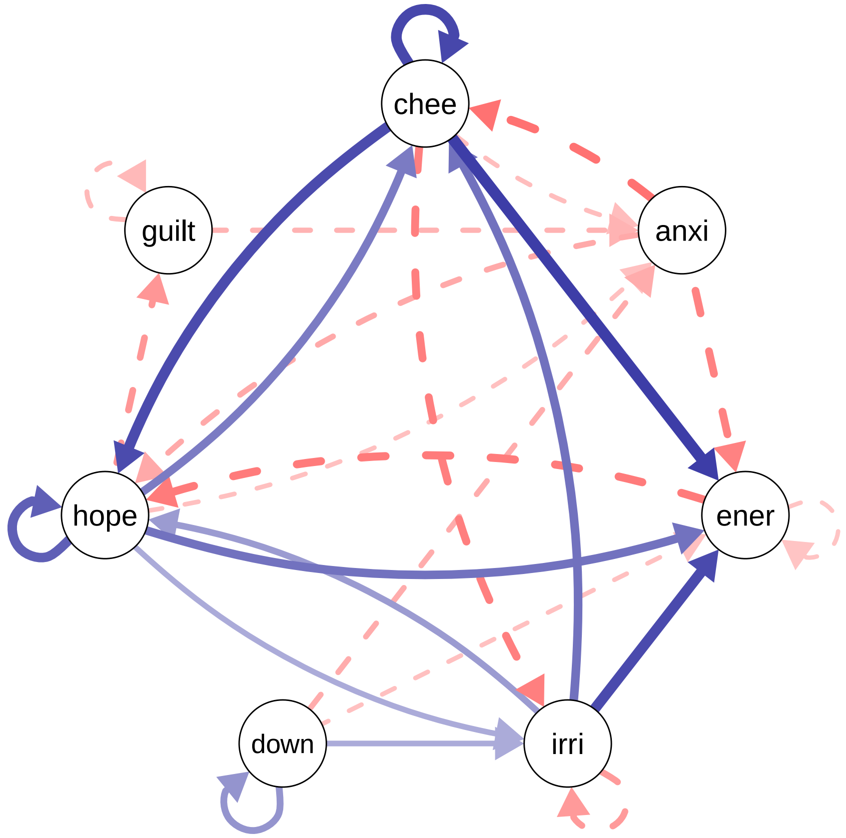

Healthy control non−reg 88JZ Estpoint 5

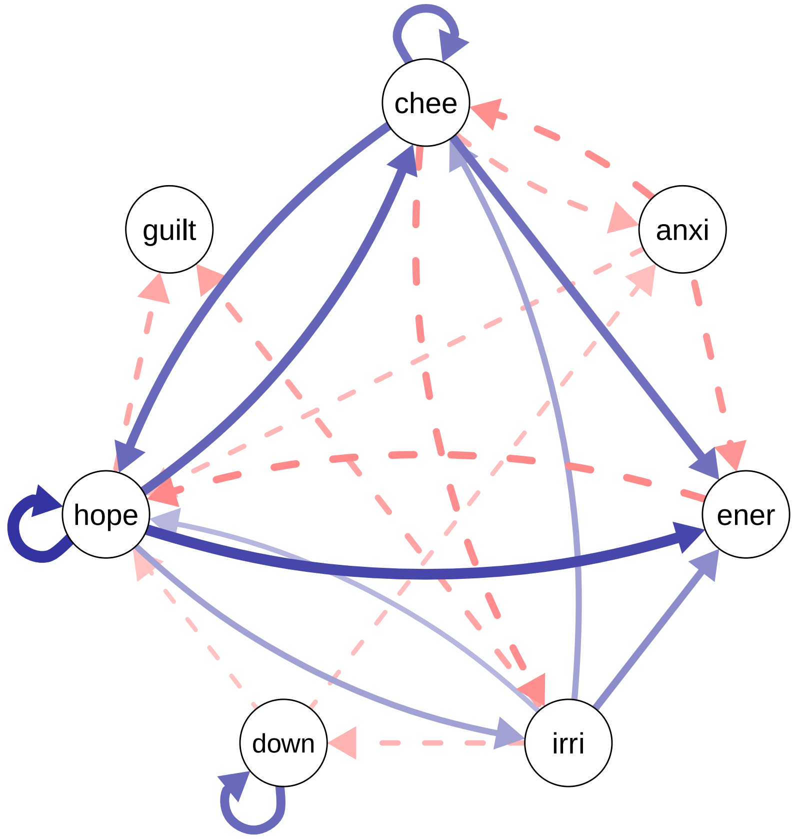

Healthy control non−reg 88JZ Estpoint 6

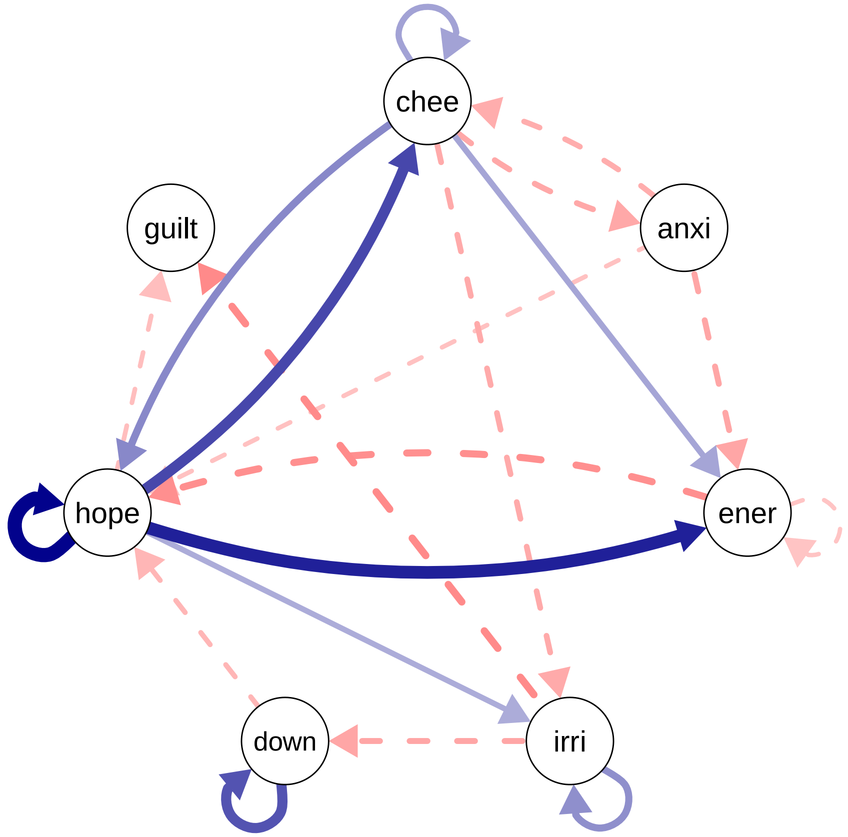

Healthy control non−reg 88JZ Estpoint 7

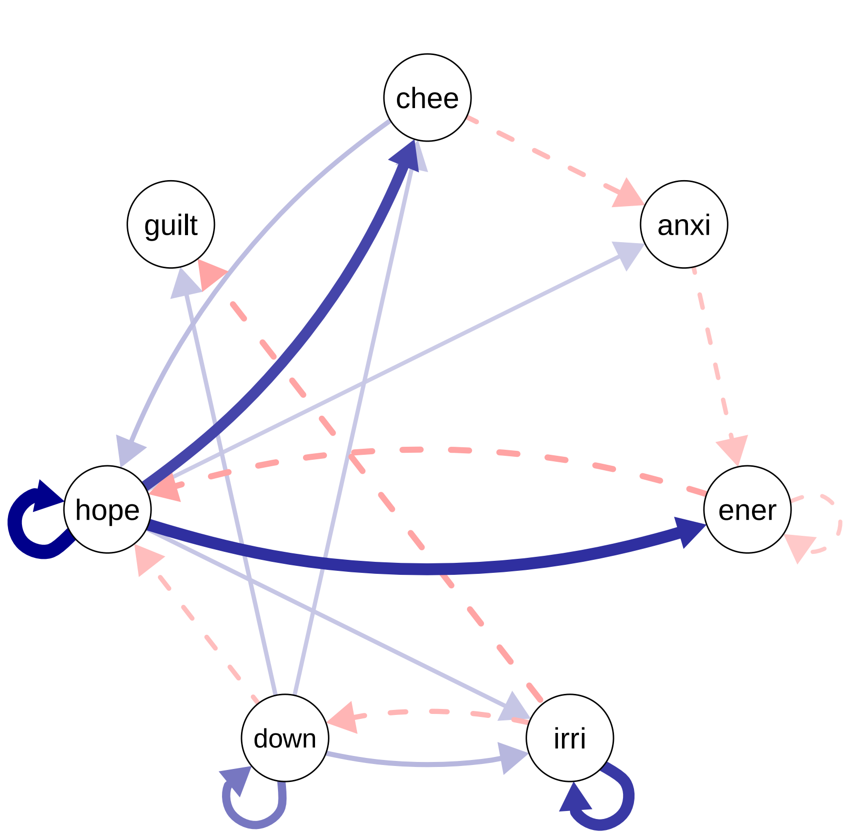

Healthy control non−reg 88JZ Estpoint 8

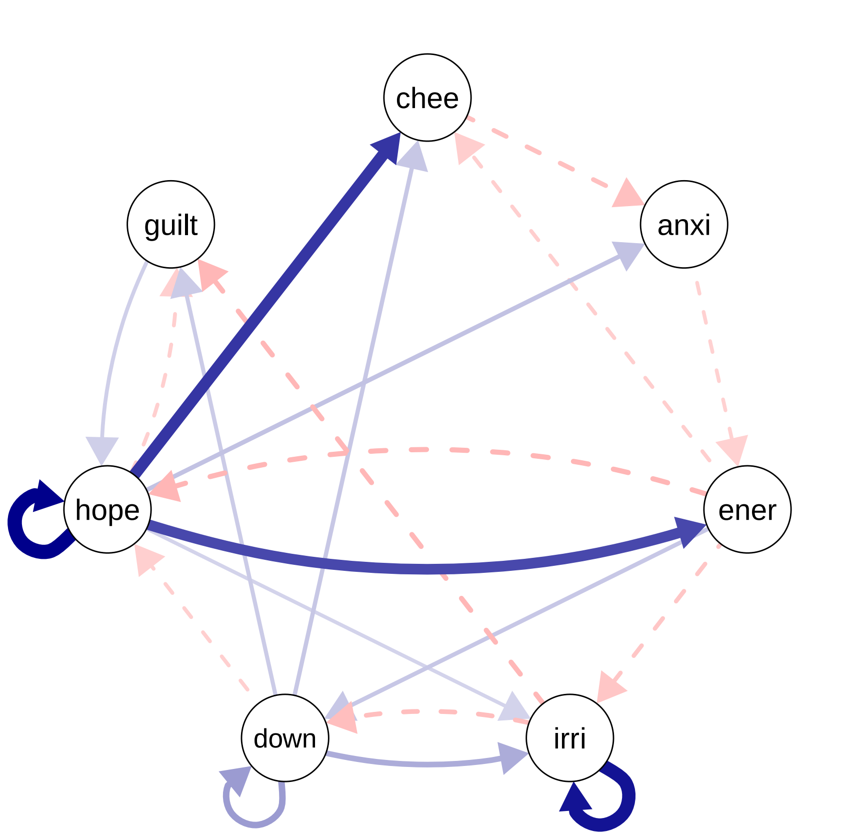

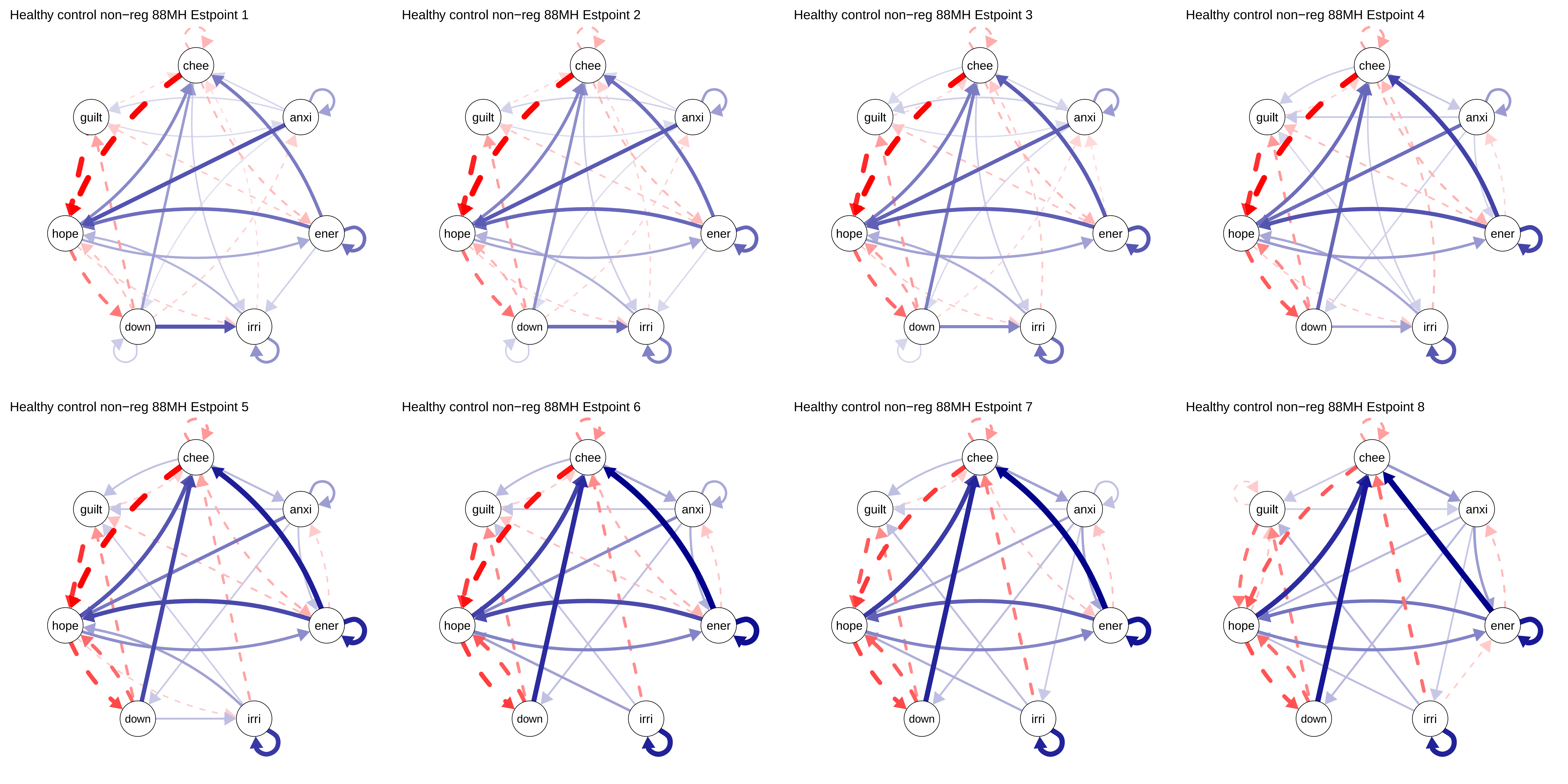
Healthy control non−reg 88MH Estpoint 1
chee
guilt
anxi
hope
ener
down
irri
Healthy control non−reg 88MH Estpoint 2
chee
guilt
anxi
hope
ener
down
irri
Healthy control non−reg 88MH Estpoint 3
chee
guilt
anxi
hope
ener
down
irri
Healthy control non−reg 88MH Estpoint 4
chee
guilt
anxi
hope
ener
down
irri
Healthy control non−reg 88MH Estpoint 5
chee
guilt
anxi
hope
ener
down
irri
Healthy control non−reg 88MH Estpoint 6
chee
guilt
anxi
hope
ener
down
irri
Healthy control non−reg 88MH Estpoint 7
chee
guilt
anxi
hope
ener
down
irri
Healthy control non−reg 88MH Estpoint 8
chee
guilt
anxi
hope
ener
down
irri

Healthy control non−reg 88JB Estpoint 1

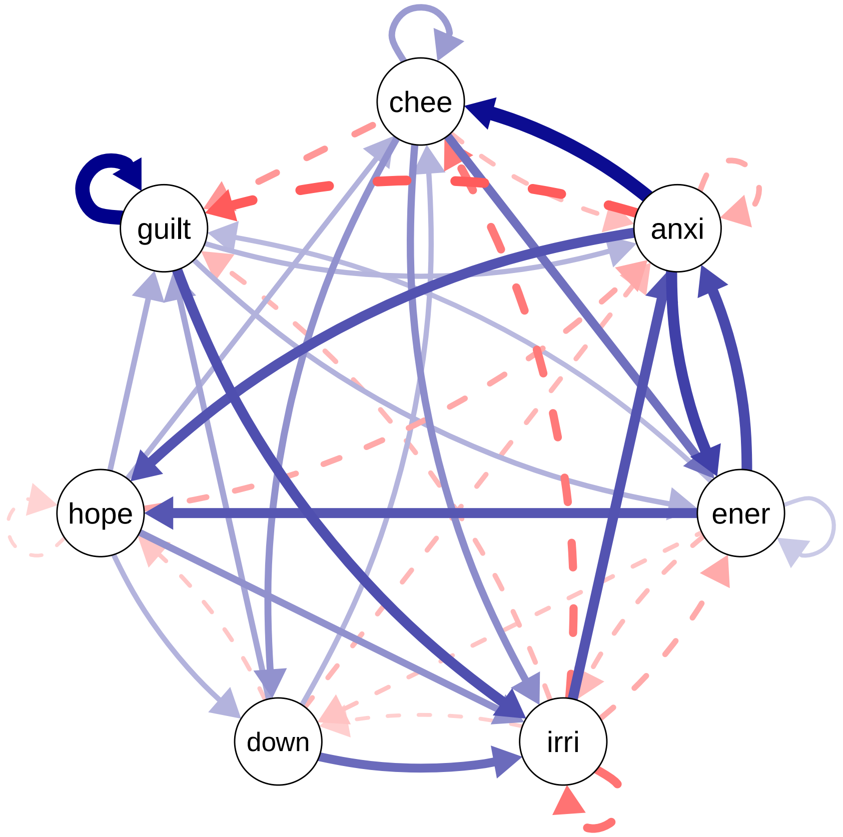

Healthy control non−reg 88JB Estpoint 2

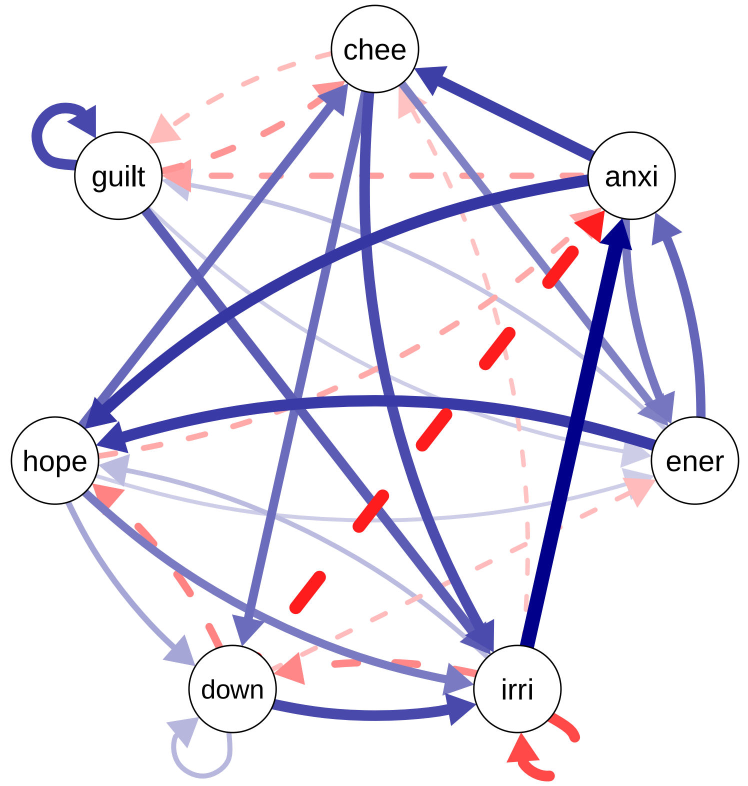

Healthy control non−reg 88JB Estpoint 3

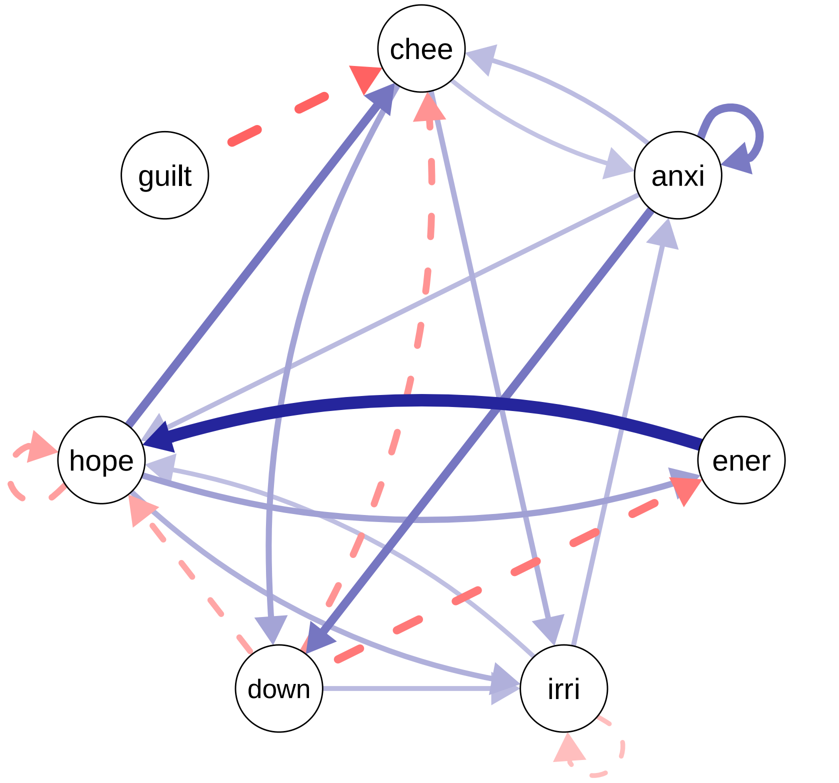

Healthy control non−reg 88JB Estpoint 4

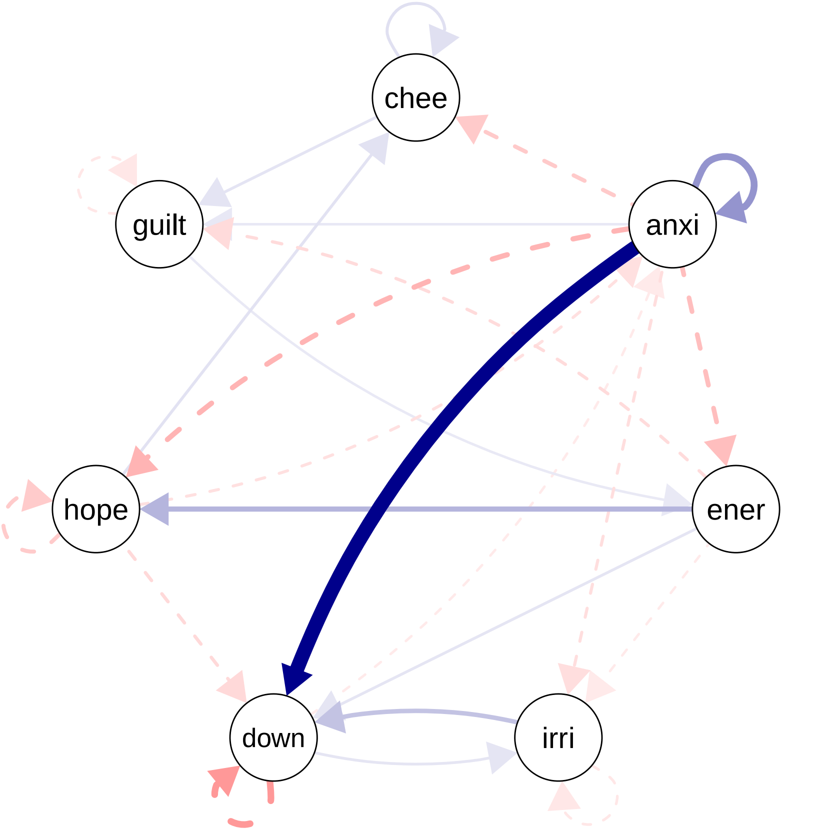

Healthy control non−reg 88JB Estpoint 5

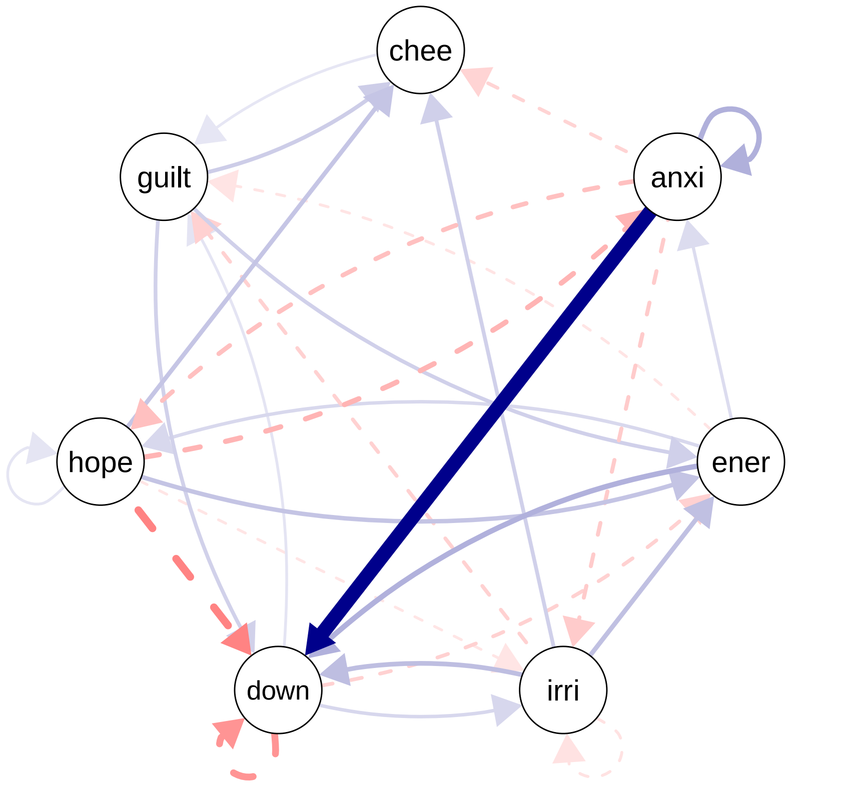

Healthy control non−reg 88JB Estpoint 6

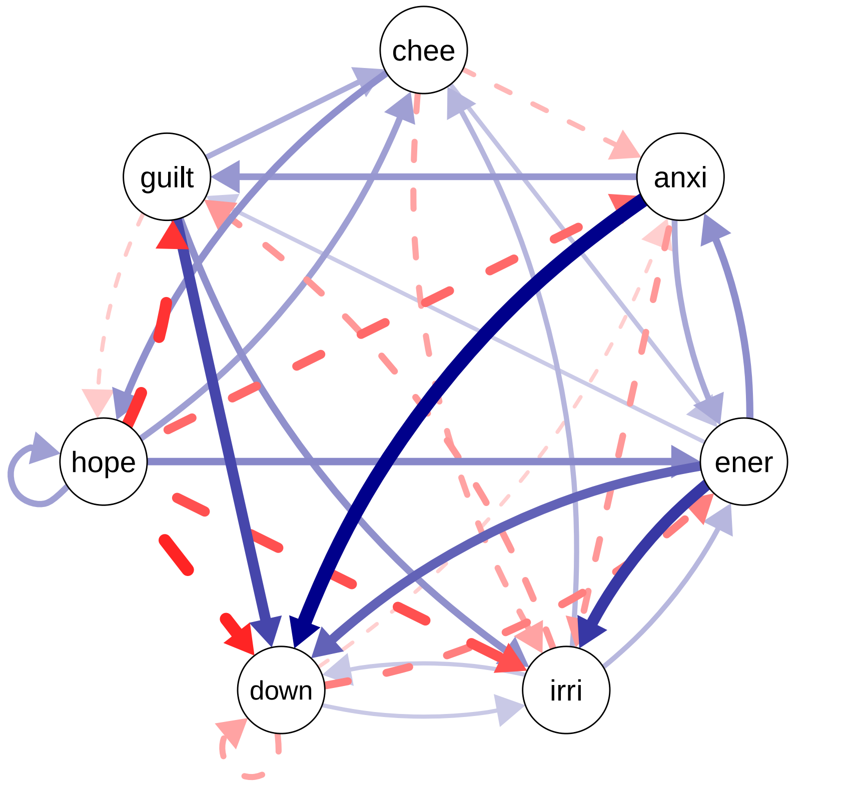

Healthy control non−reg 88JB Estpoint 7

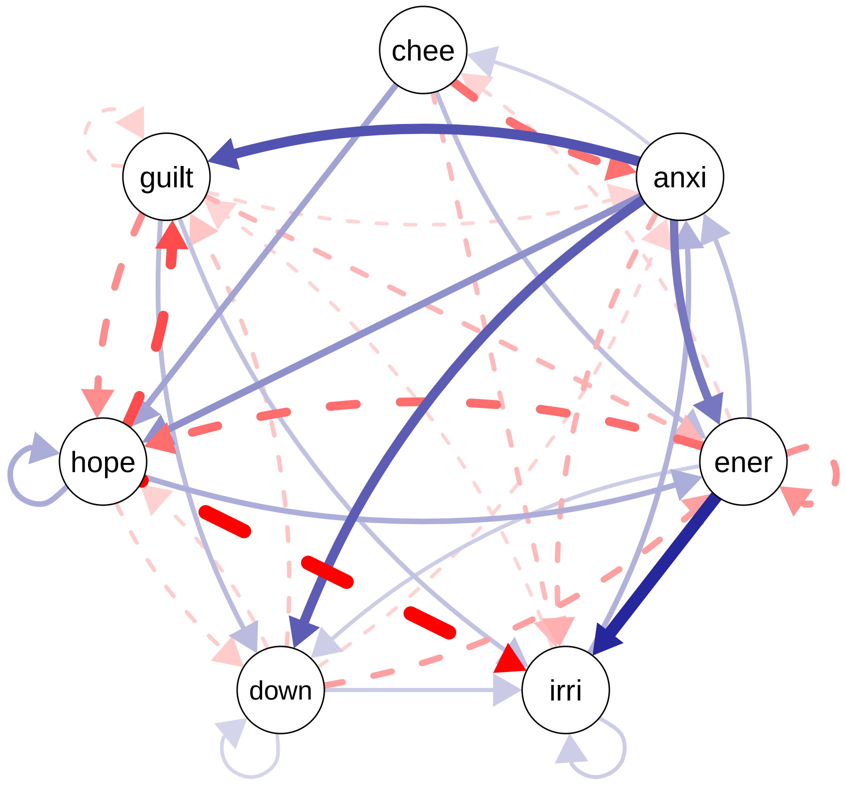

Healthy control non−reg 88JB Estpoint 8

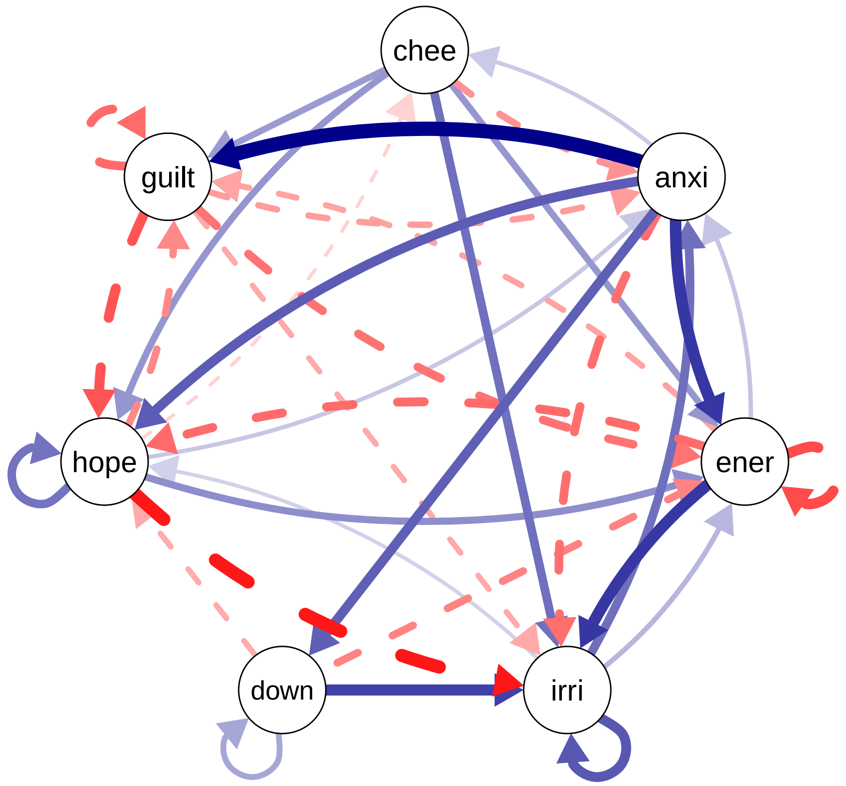

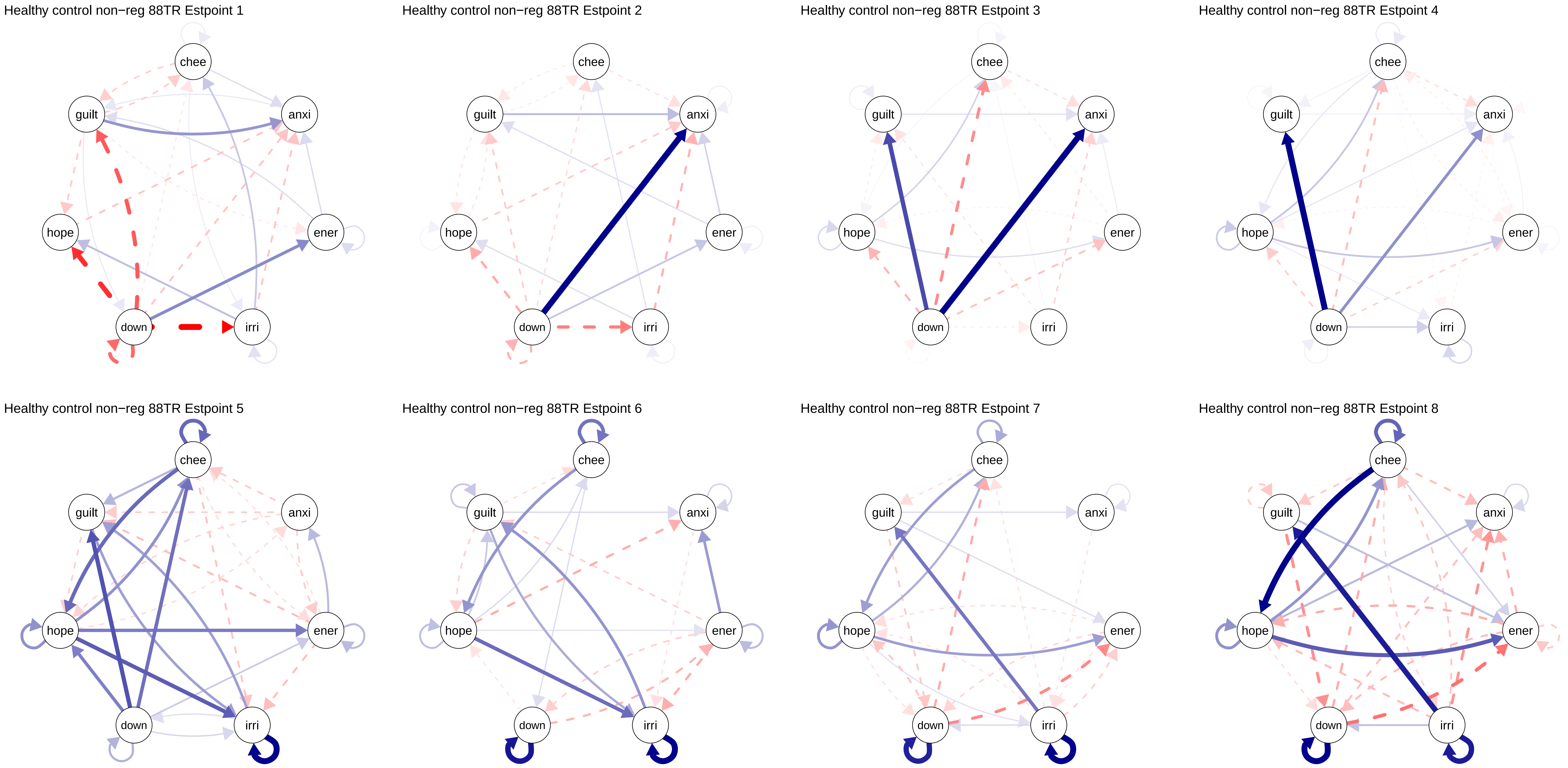
Healthy control non−reg 88TR Estpoint 1
Healthy control non−reg 88TR Estpoint 2
Healthy control non−reg 88TR Estpoint 3
Healthy control non−reg 88TR Estpoint 4
Healthy control non−reg 88TR Estpoint 5
Healthy control non−reg 88TR Estpoint 6
Healthy control non−reg 88TR Estpoint 7
Healthy control non−reg 88TR Estpoint 8
chee
guilt
anxi
hope
ener
down
irri

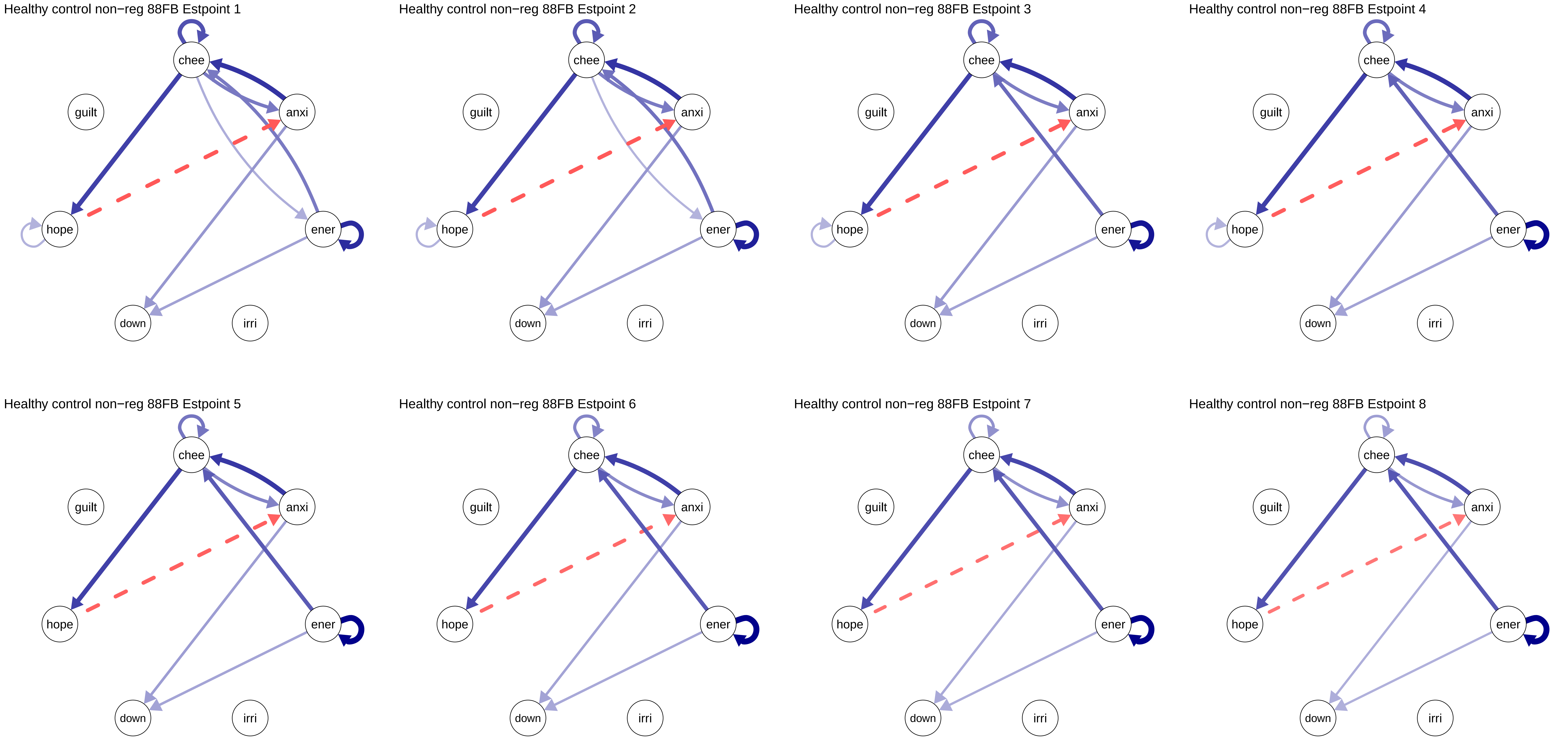
Healthy control non−reg 88FB Estpoint 1
chee guilt anxi hope ener down irri
Healthy control non−reg 88FB Estpoint 2
chee guilt anxi hope ener down irri
Healthy control non−reg 88FB Estpoint 3
chee guilt anxi hope ener down irri
Healthy control non−reg 88FB Estpoint 4
chee guilt anxi hope ener down irri
Healthy control non−reg 88FB Estpoint 5
chee guilt anxi hope ener down irri
Healthy control non−reg 88FB Estpoint 6
chee guilt anxi hope ener down irri
Healthy control non−reg 88FB Estpoint 7
chee guilt anxi hope ener down irri
Healthy control non−reg 88FB Estpoint 8
chee guilt anxi hope ener down irri

Healthy control non−reg 88LJ Estpoint 1

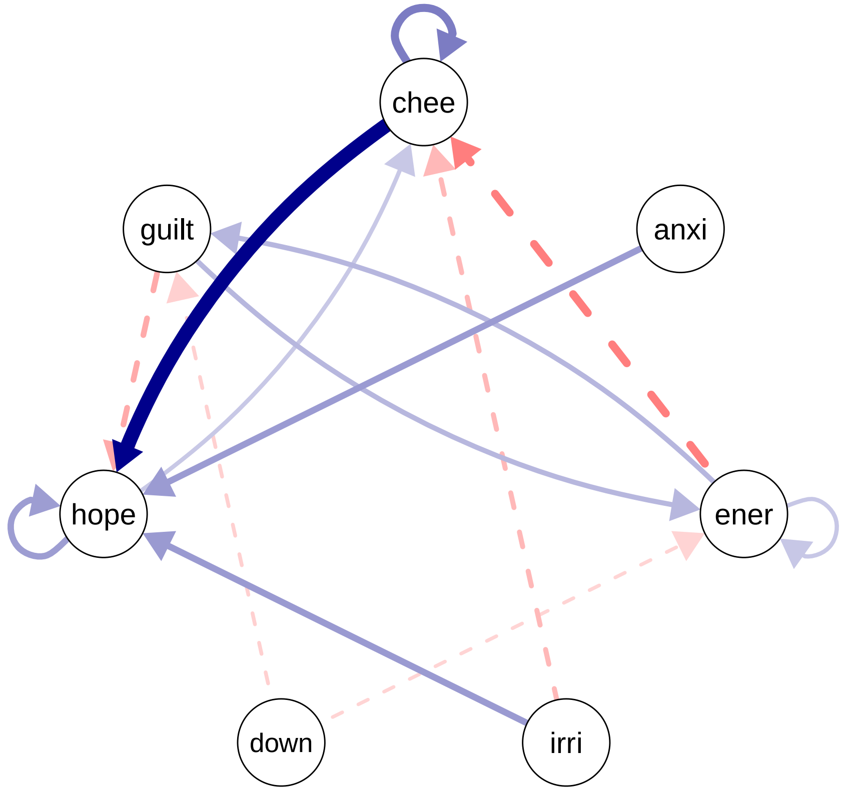

Healthy control non−reg 88LJ Estpoint 2

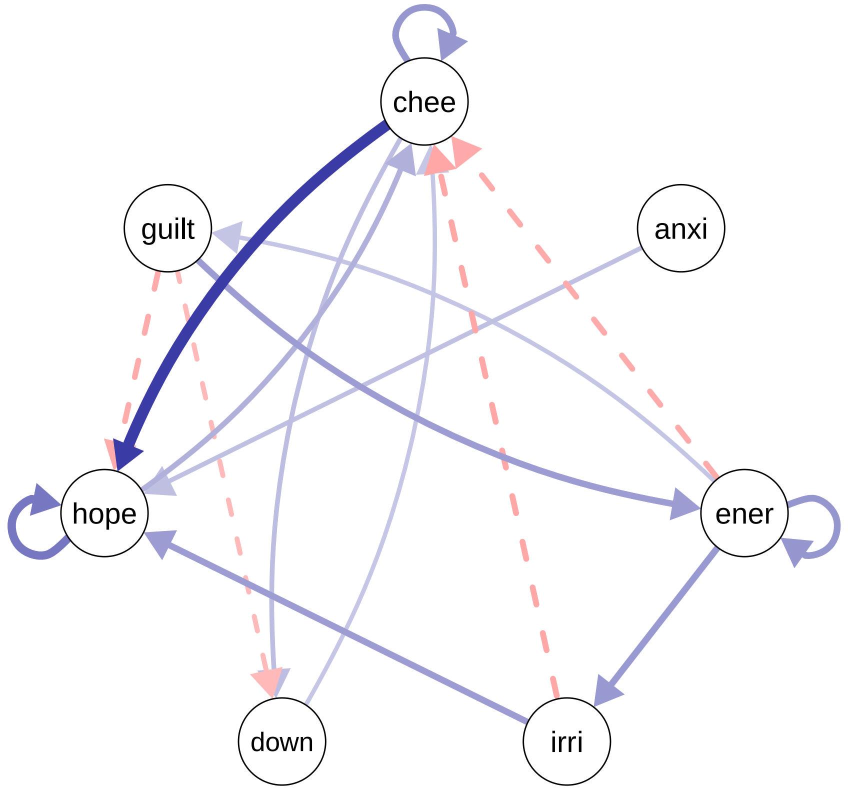

Healthy control non−reg 88LJ Estpoint 3

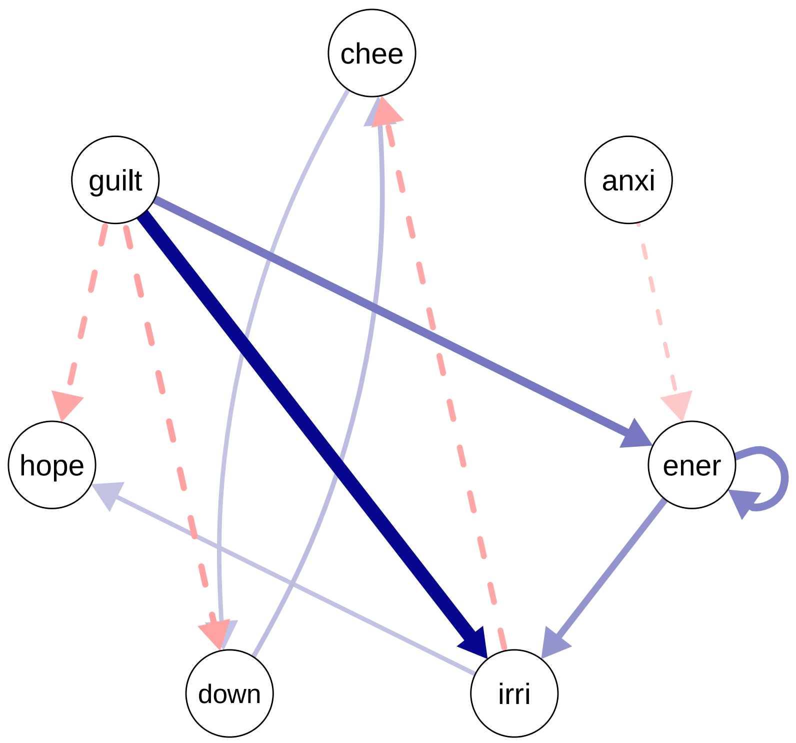

Healthy control non−reg 88LJ Estpoint 4

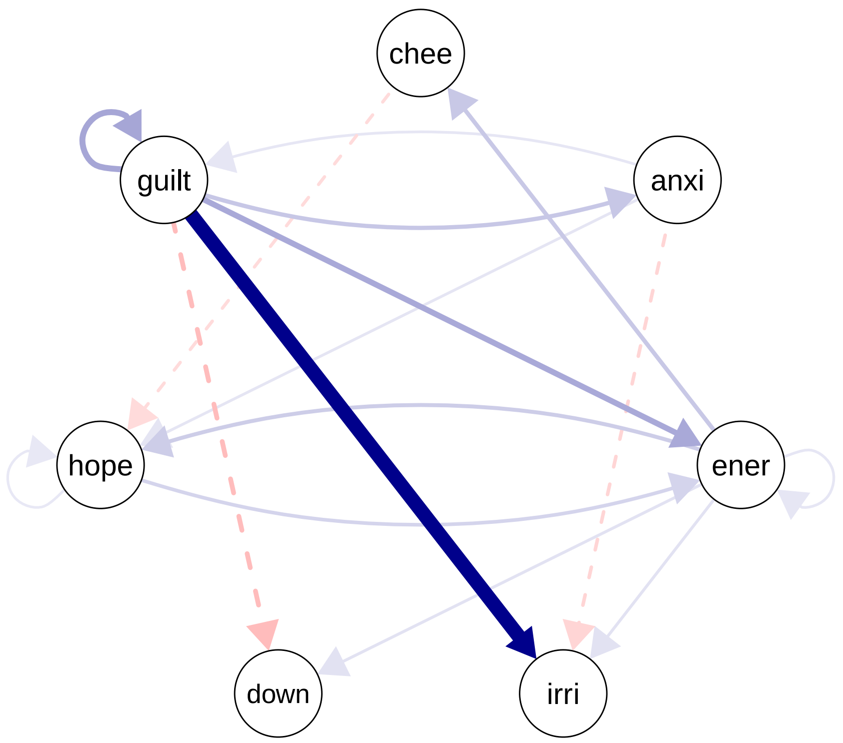

Healthy control non−reg 88LJ Estpoint 5

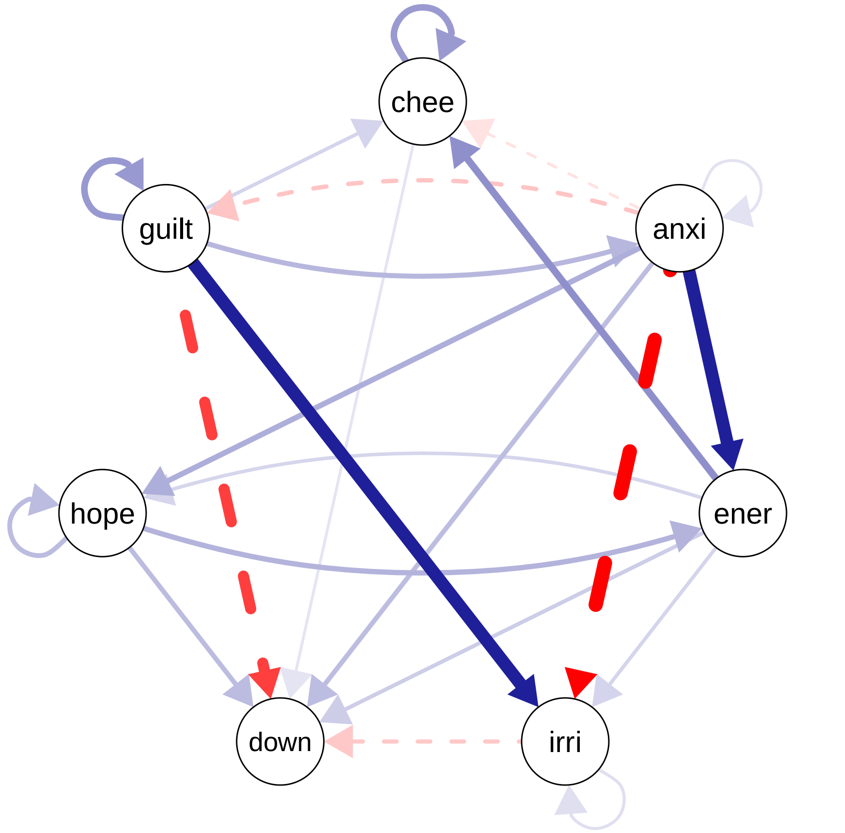

Healthy control non−reg 88LJ Estpoint 6

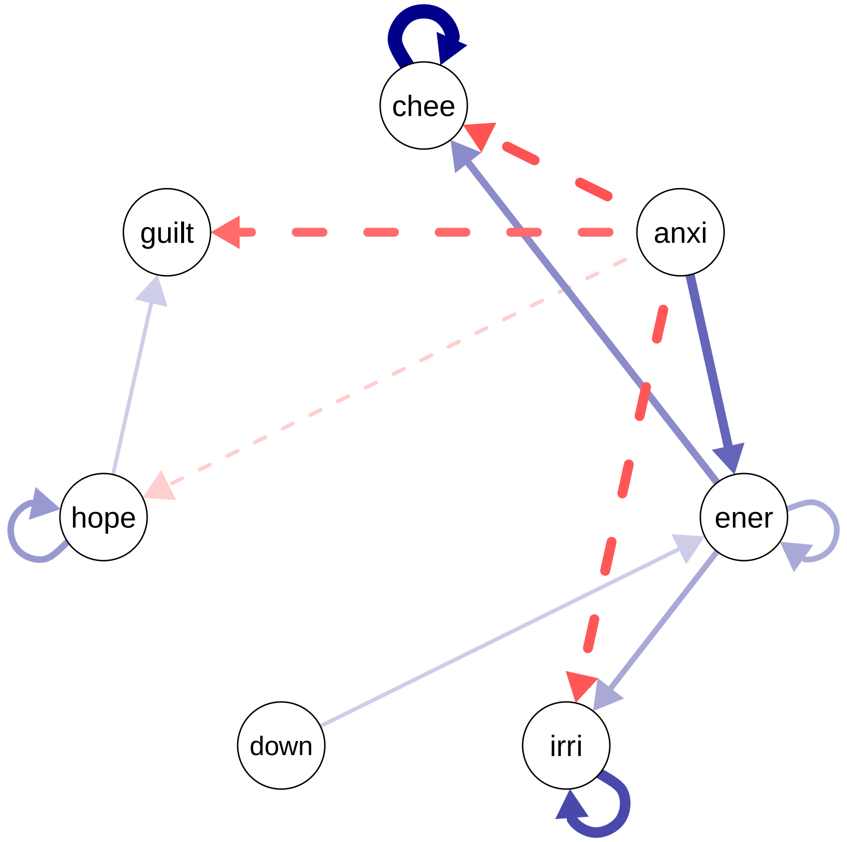

Healthy control non−reg 88LJ Estpoint 7

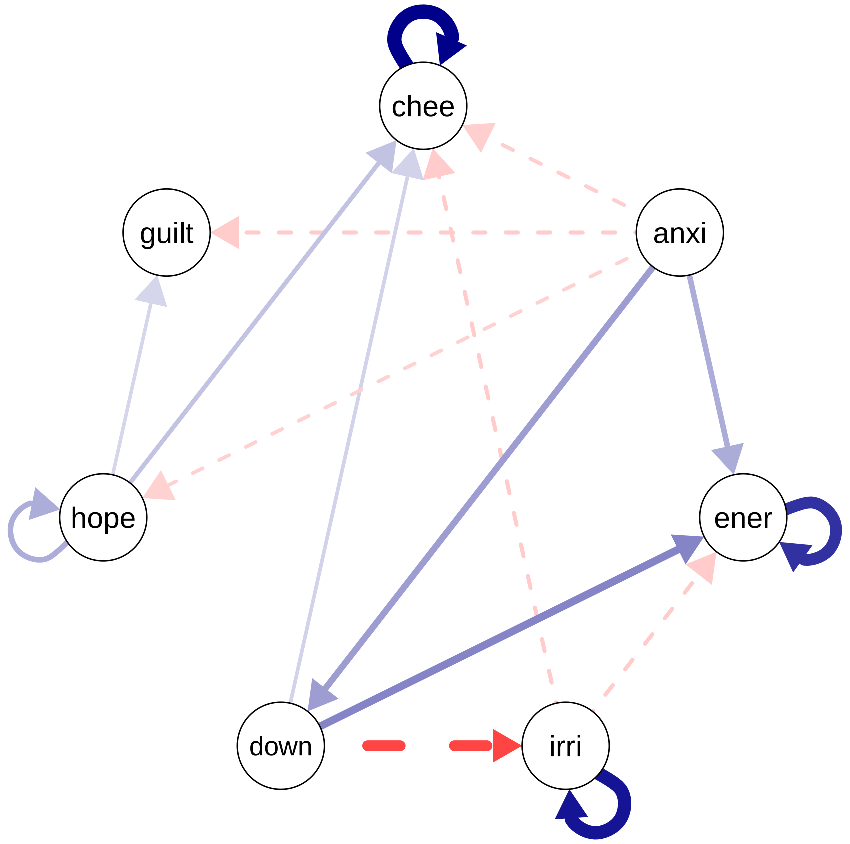

Healthy control non−reg 88LJ Estpoint 8

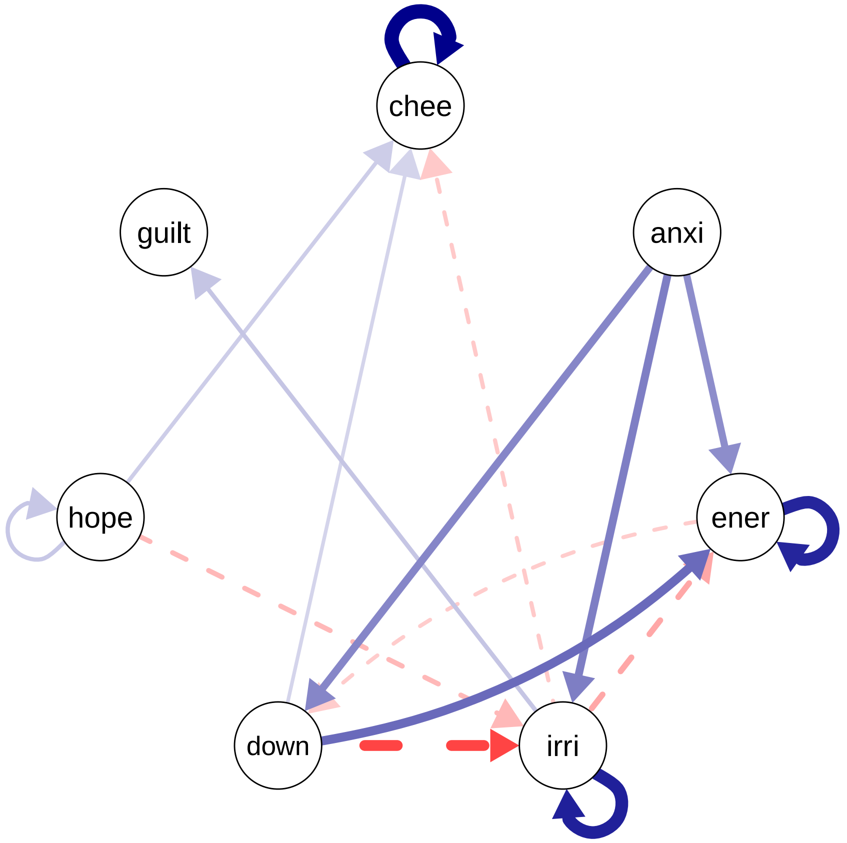

Healthy control non−reg 88MHB Estpoint 1

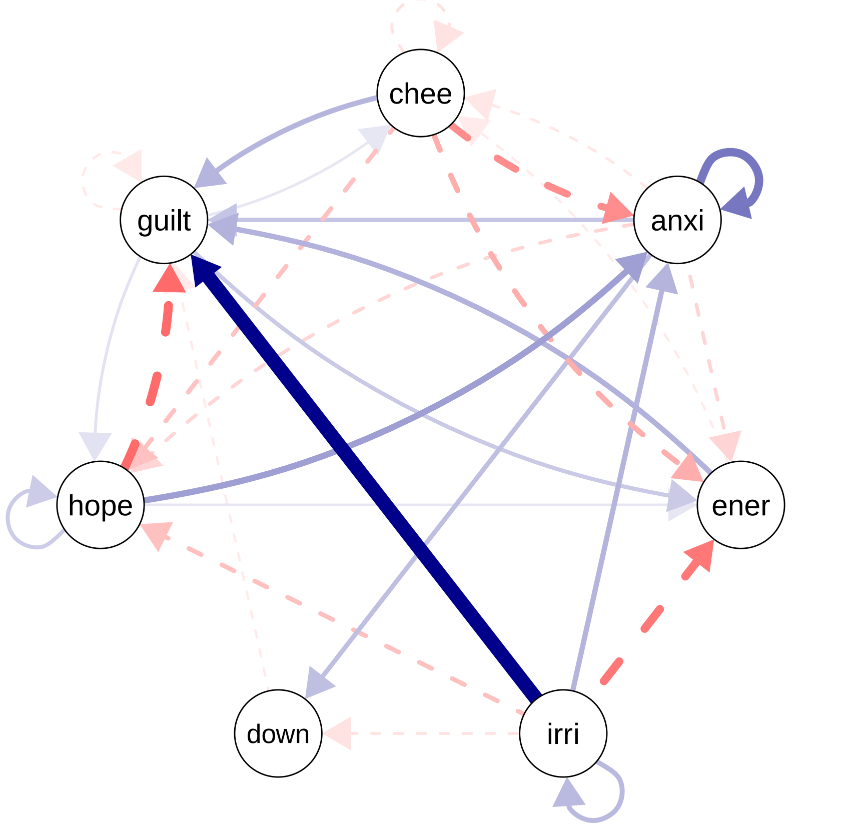

Healthy control non−reg 88MHB Estpoint 2

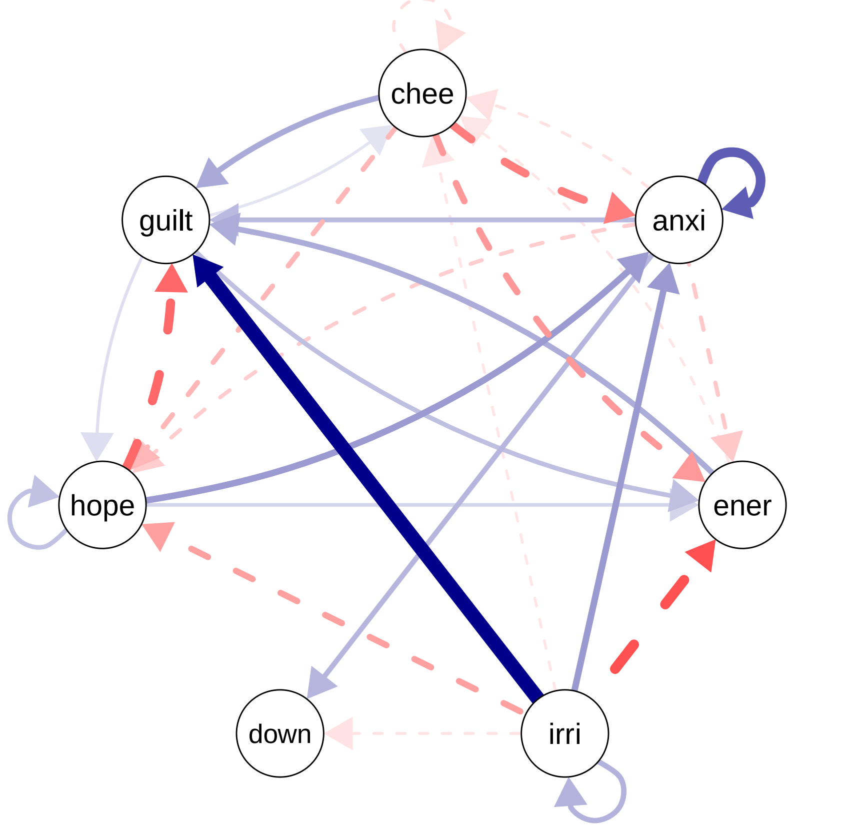

Healthy control non−reg 88MHB Estpoint 3

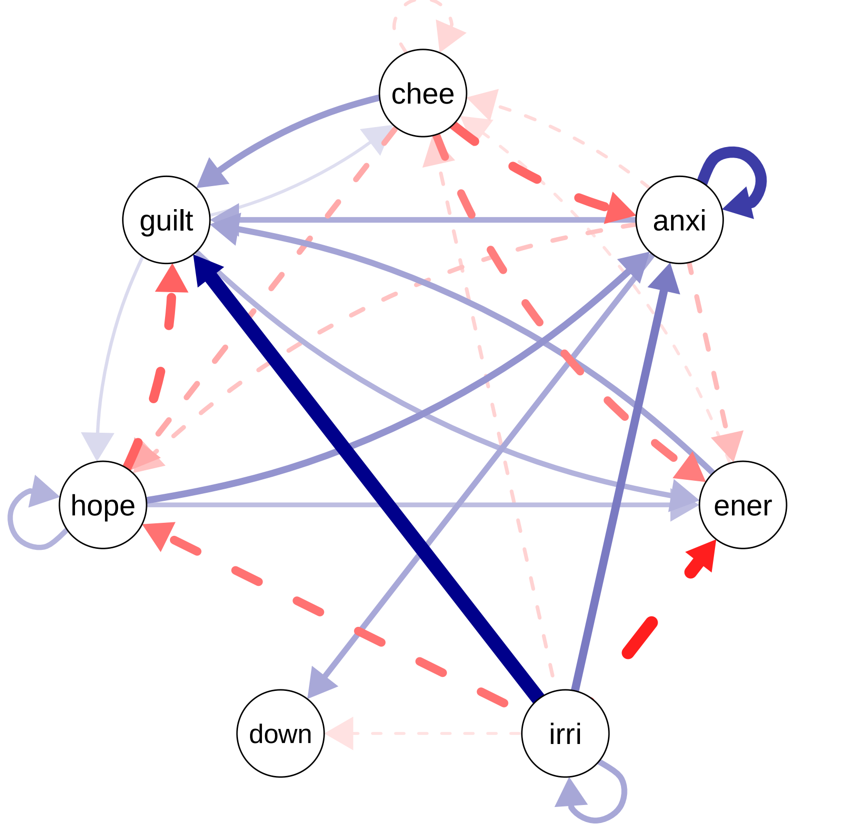

Healthy control non−reg 88MHB Estpoint 4

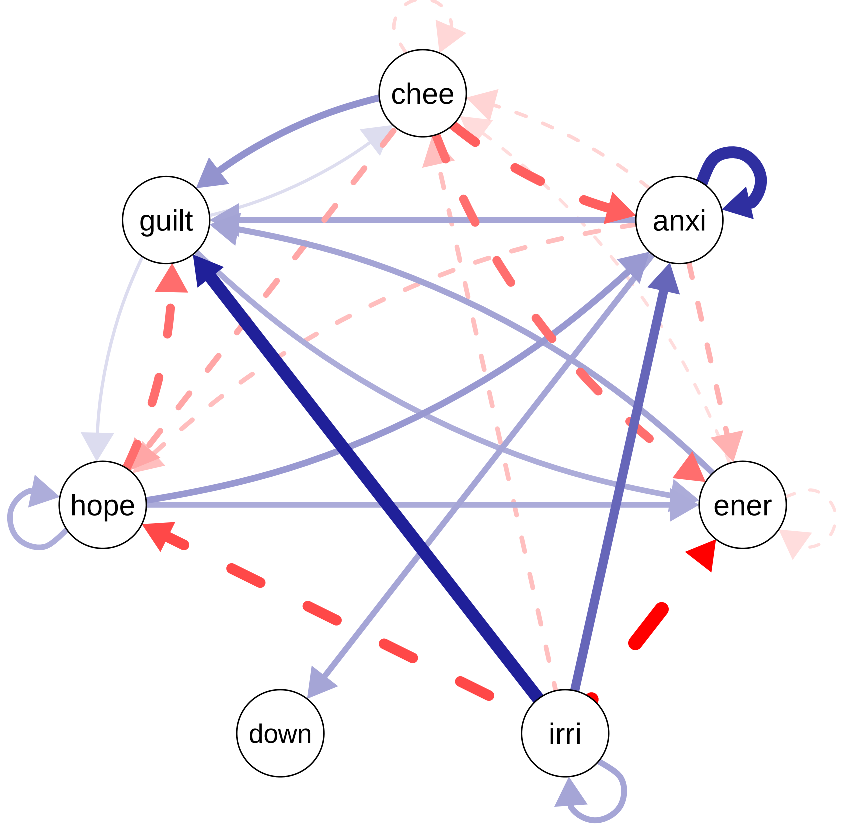

Healthy control non−reg 88MHB Estpoint 5

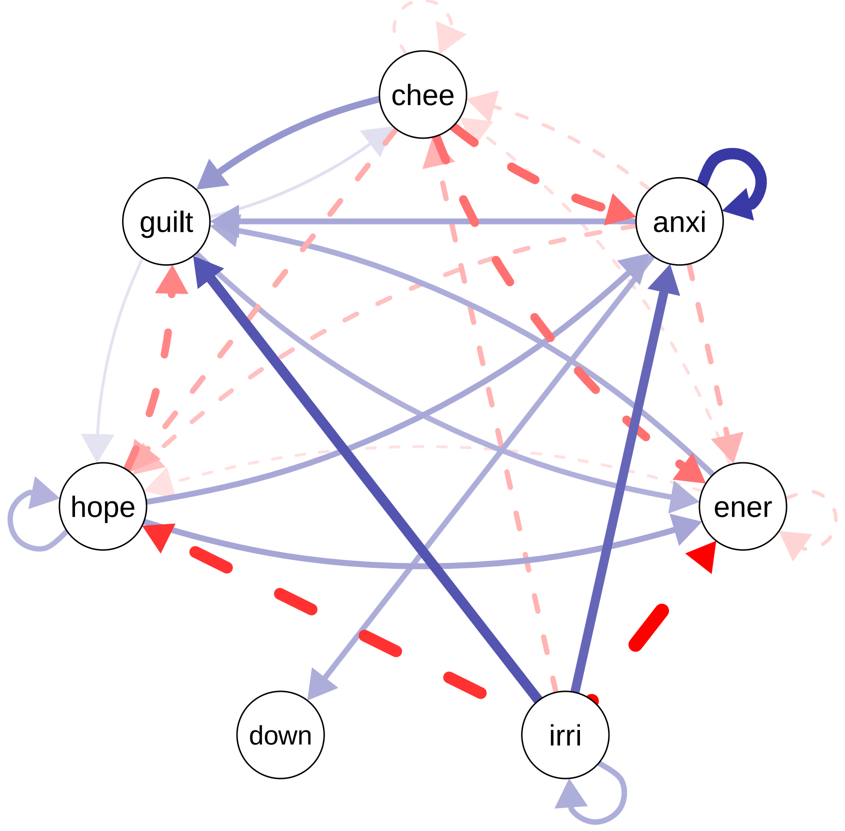

Healthy control non−reg 88MHB Estpoint 6

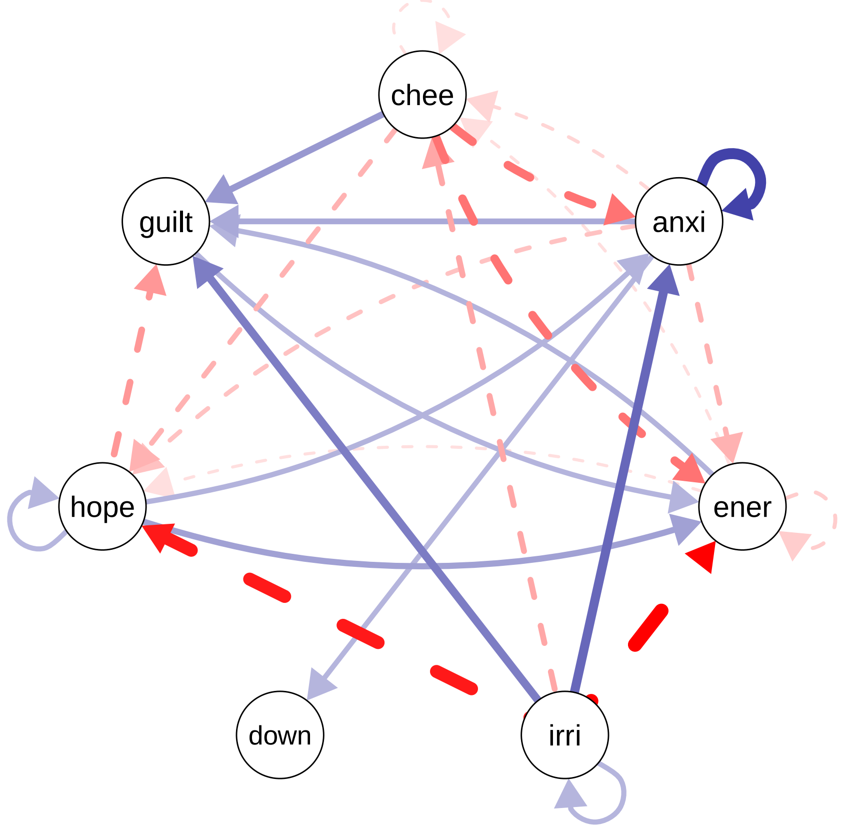

Healthy control non−reg 88MHB Estpoint 7

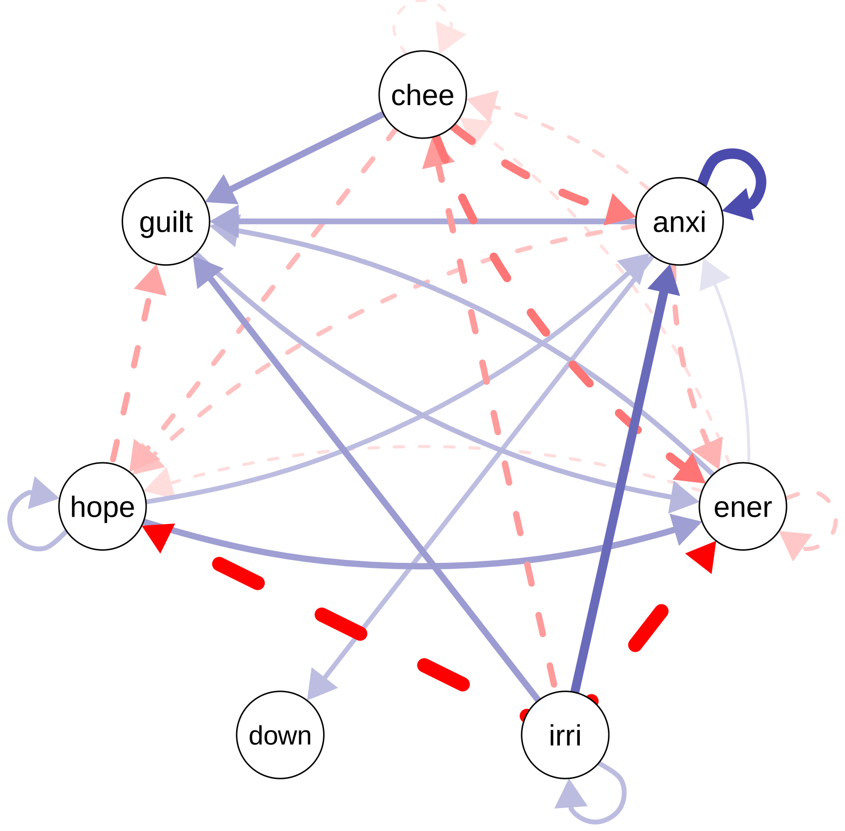

Healthy control non−reg 88MHB Estpoint 8

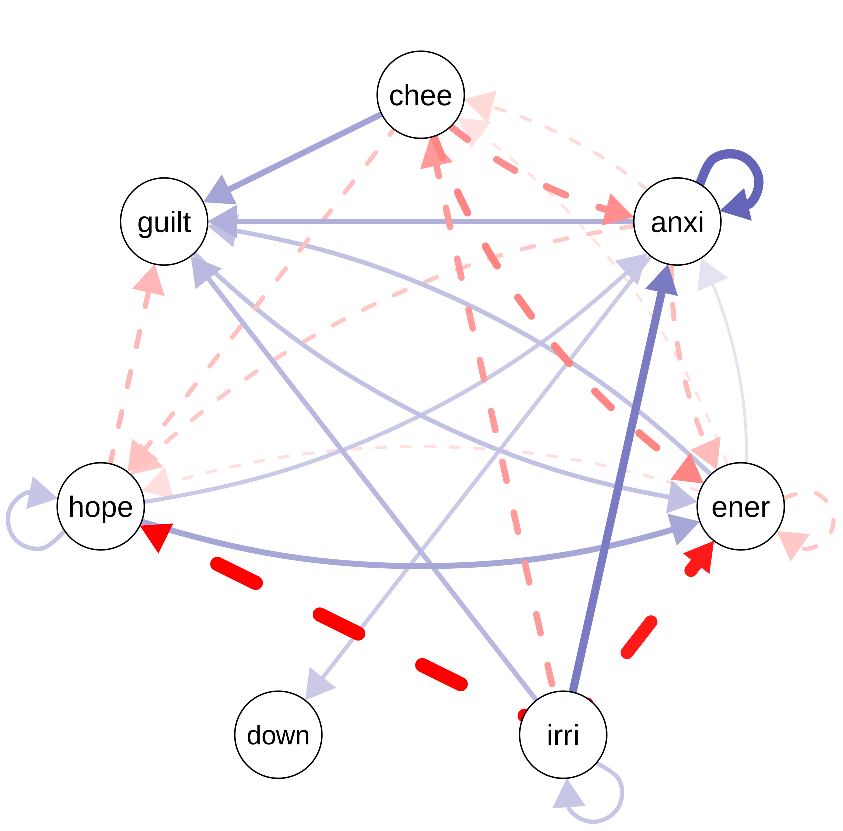

Healthy control non−reg 88SS Estpoint 1

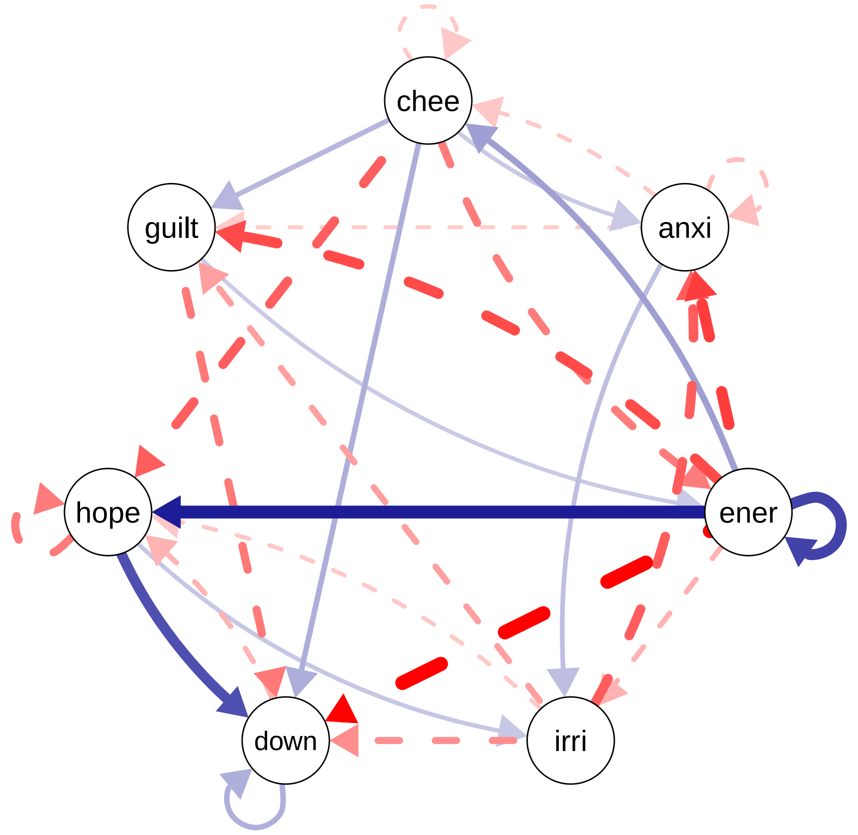

Healthy control non−reg 88SS Estpoint 2

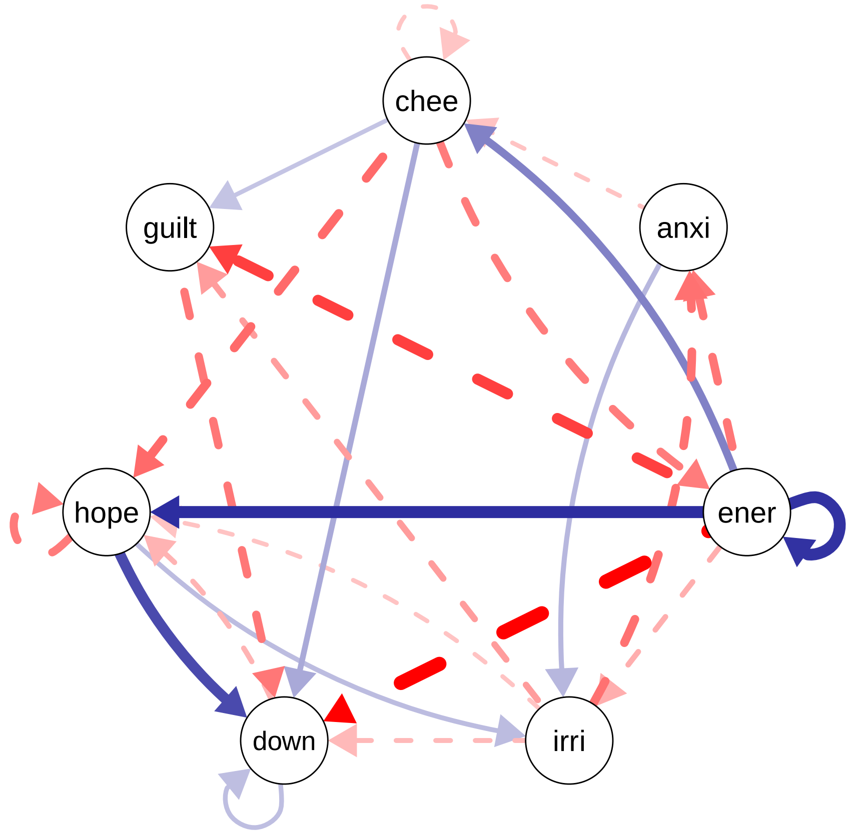

Healthy control non−reg 88SS Estpoint 3

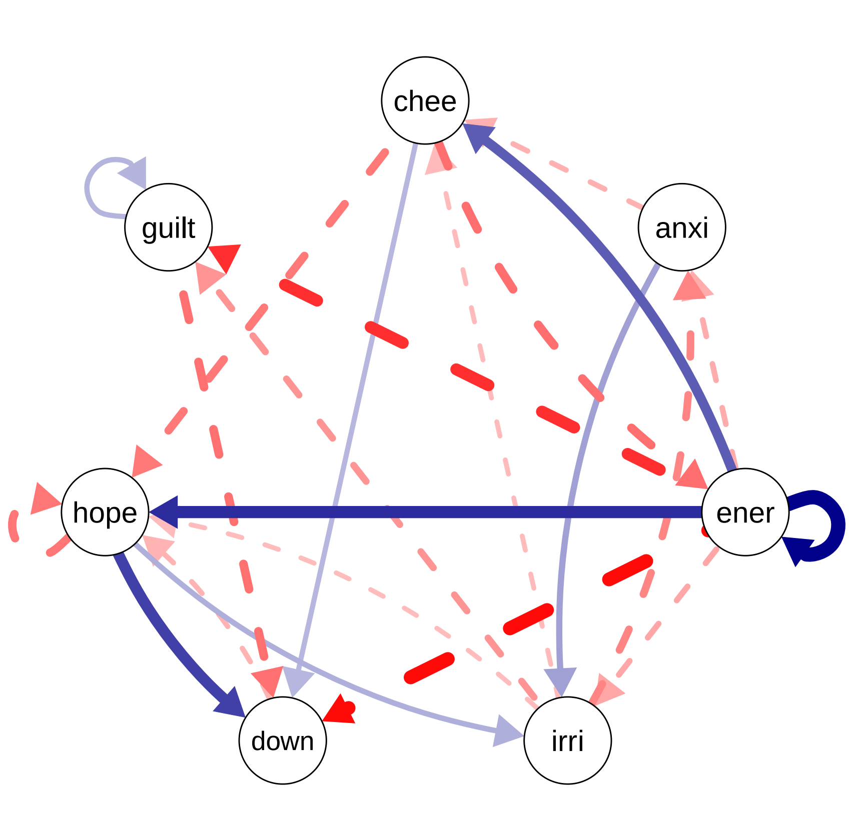

Healthy control non−reg 88SS Estpoint 4

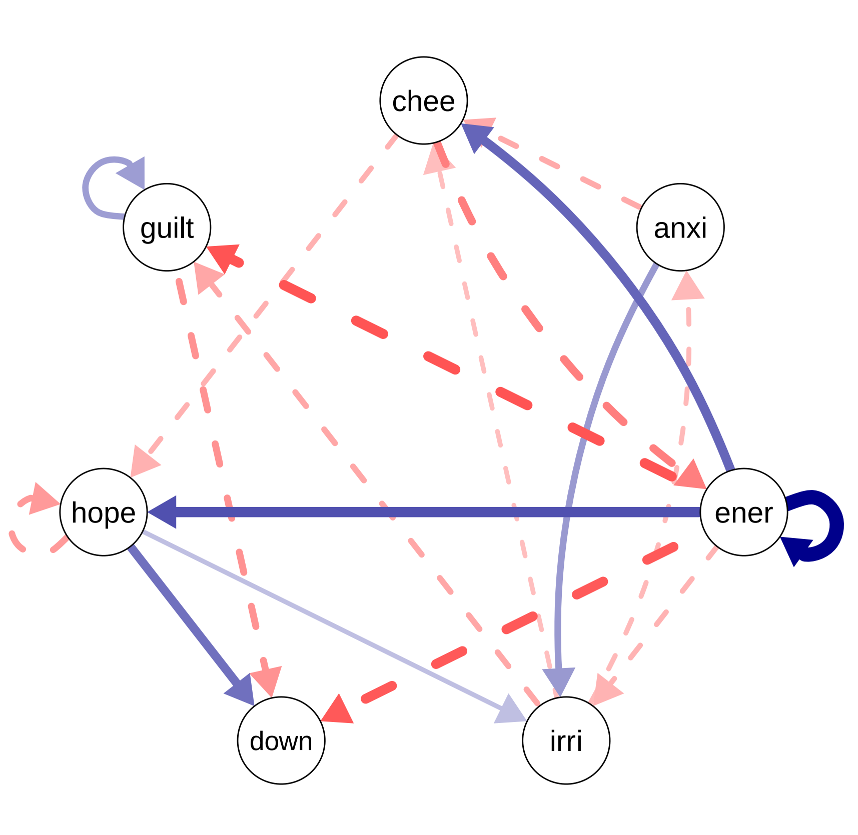

Healthy control non−reg 88SS Estpoint 5

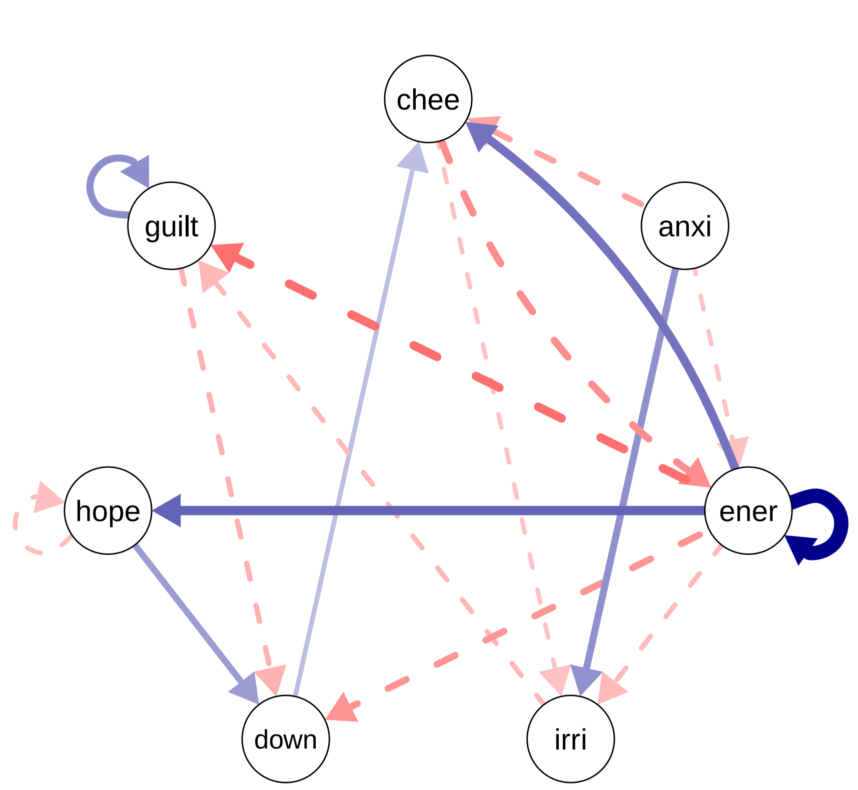

Healthy control non−reg 88SS Estpoint 6

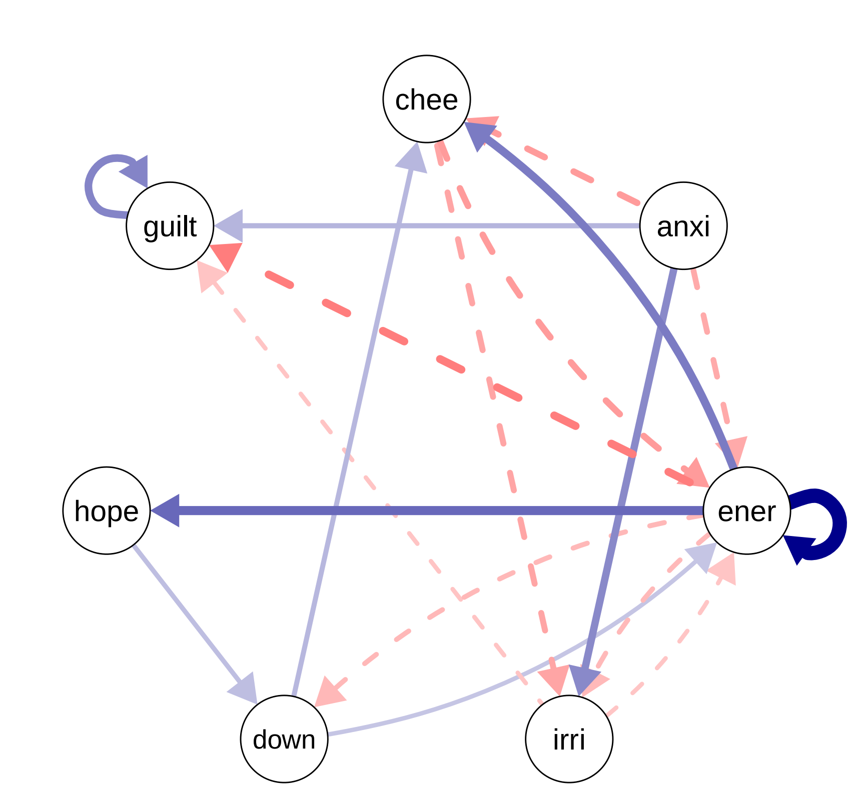

Healthy control non−reg 88SS Estpoint 7

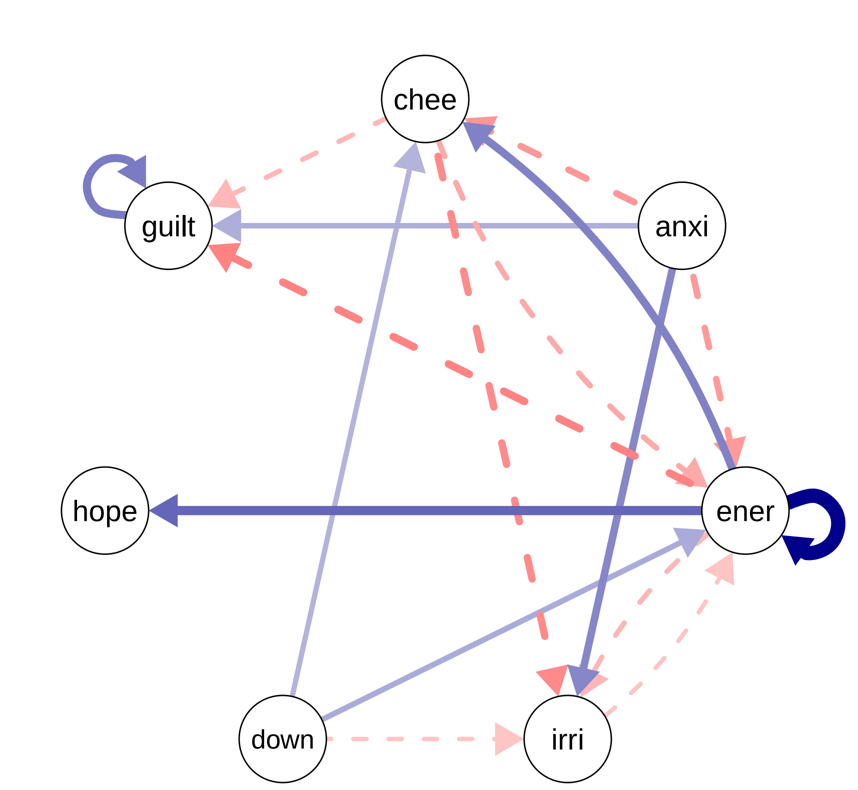

Healthy control non−reg 88SS Estpoint 8

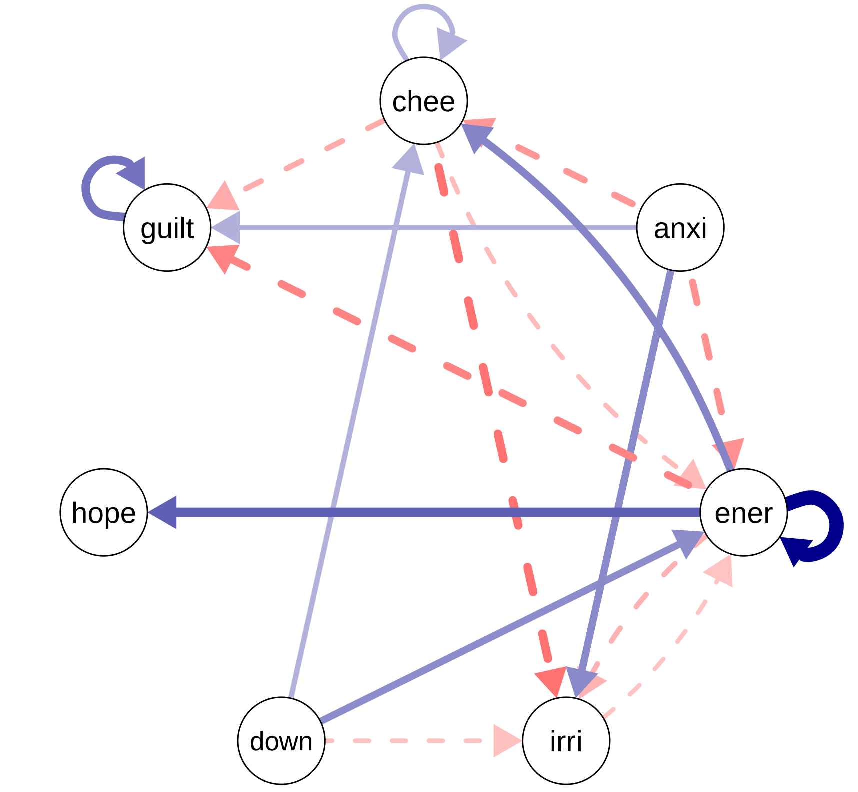

Healthy control non−reg 88JV Estpoint 1

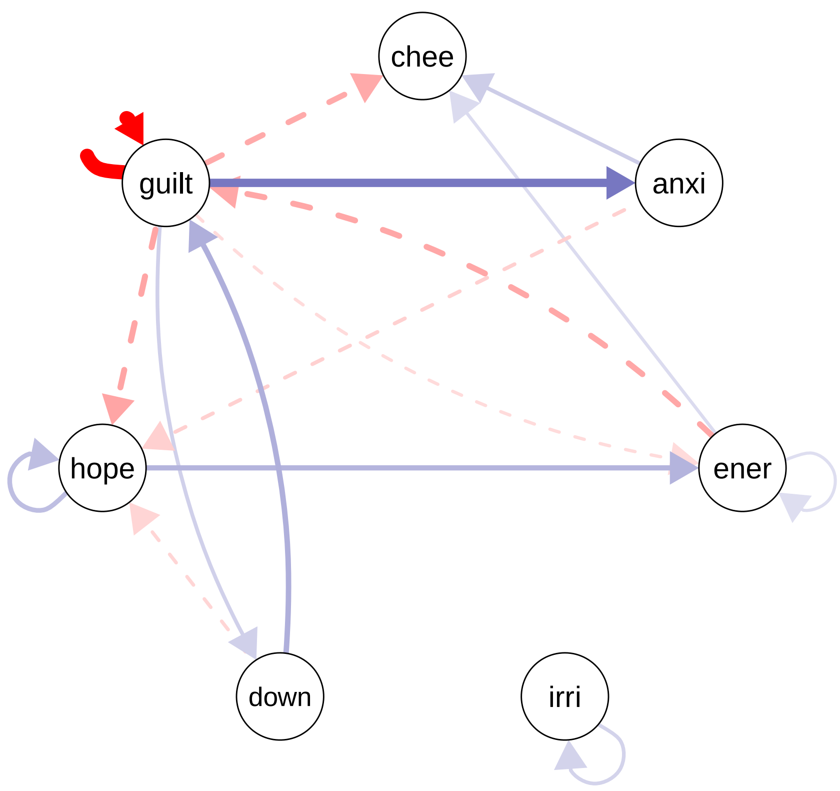

Healthy control non−reg 88JV Estpoint 2

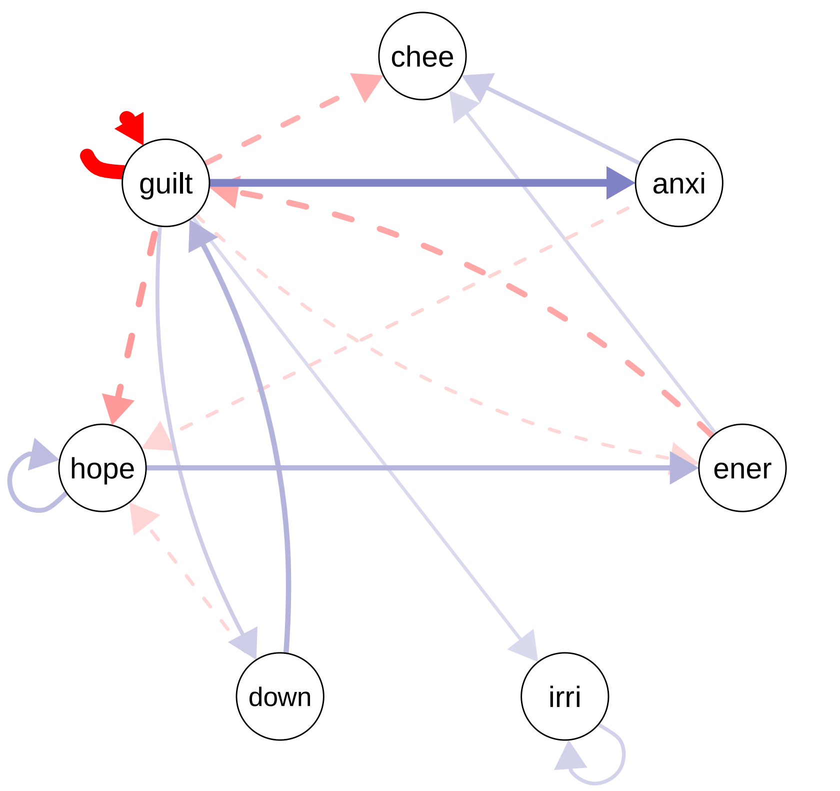

Healthy control non−reg 88JV Estpoint 3

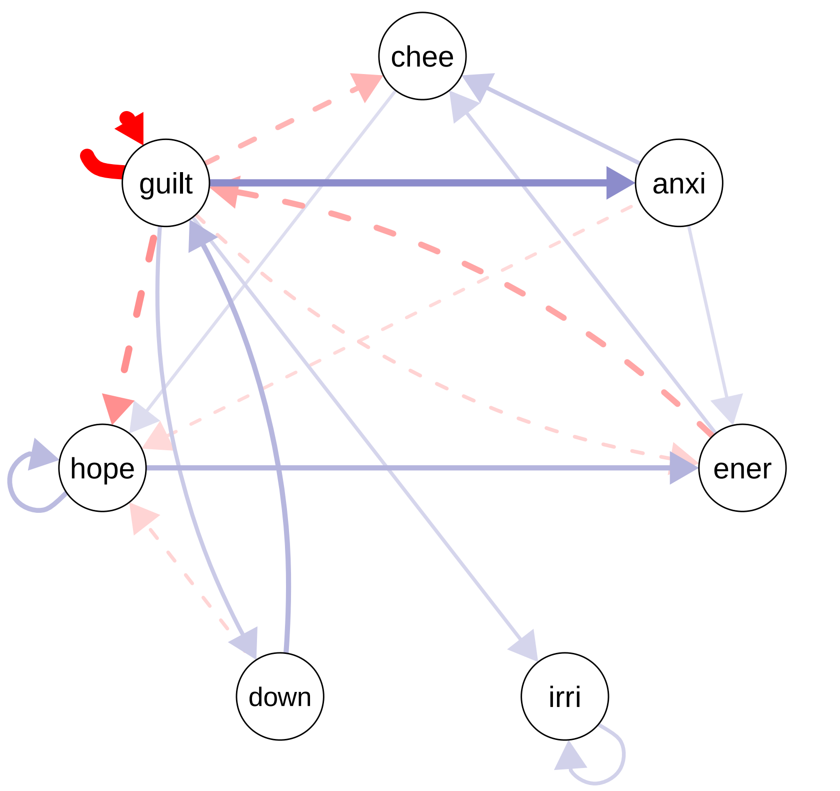

Healthy control non−reg 88JV Estpoint 4

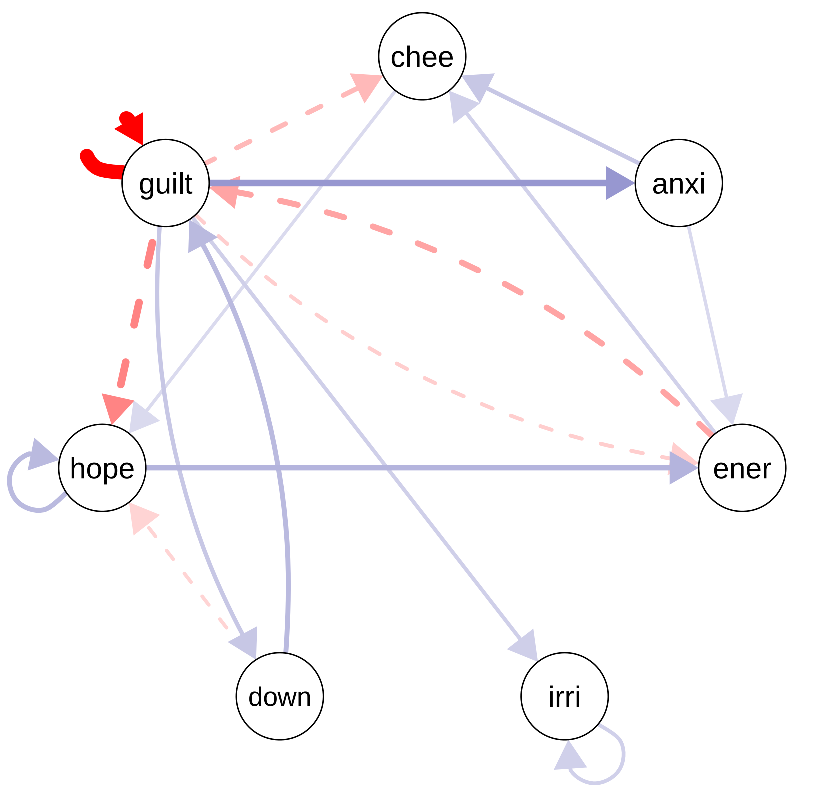

Healthy control non−reg 88JV Estpoint 5

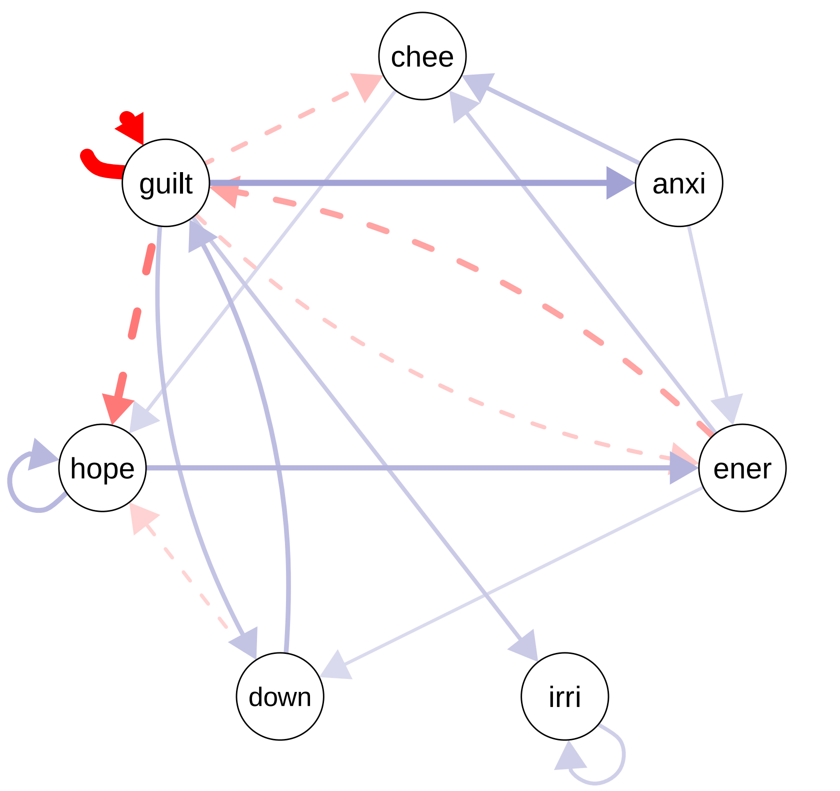

Healthy control non−reg 88JV Estpoint 6

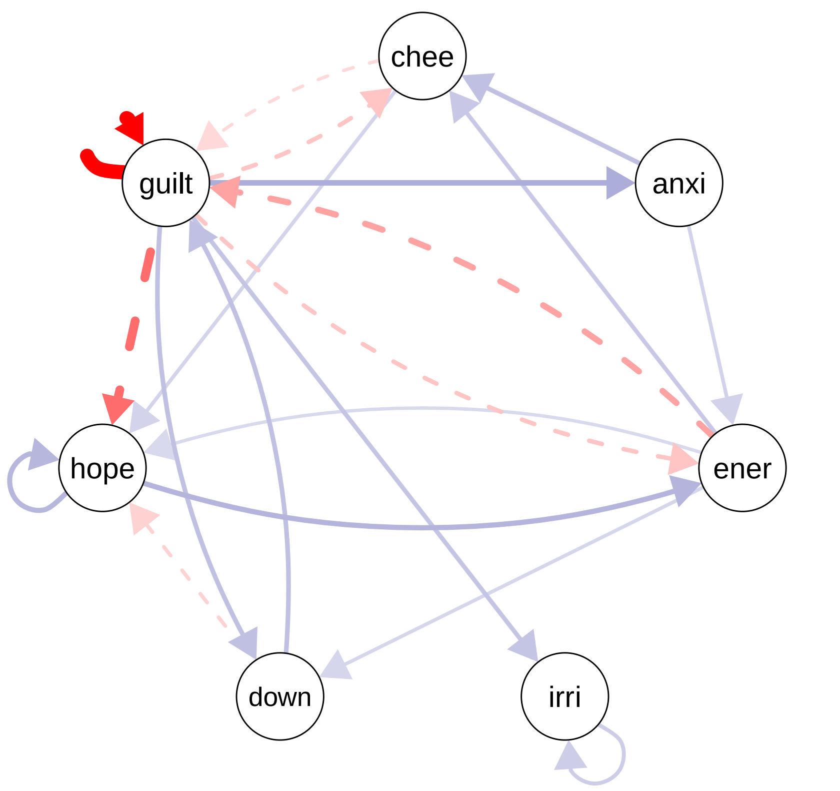

Healthy control non−reg 88JV Estpoint 7

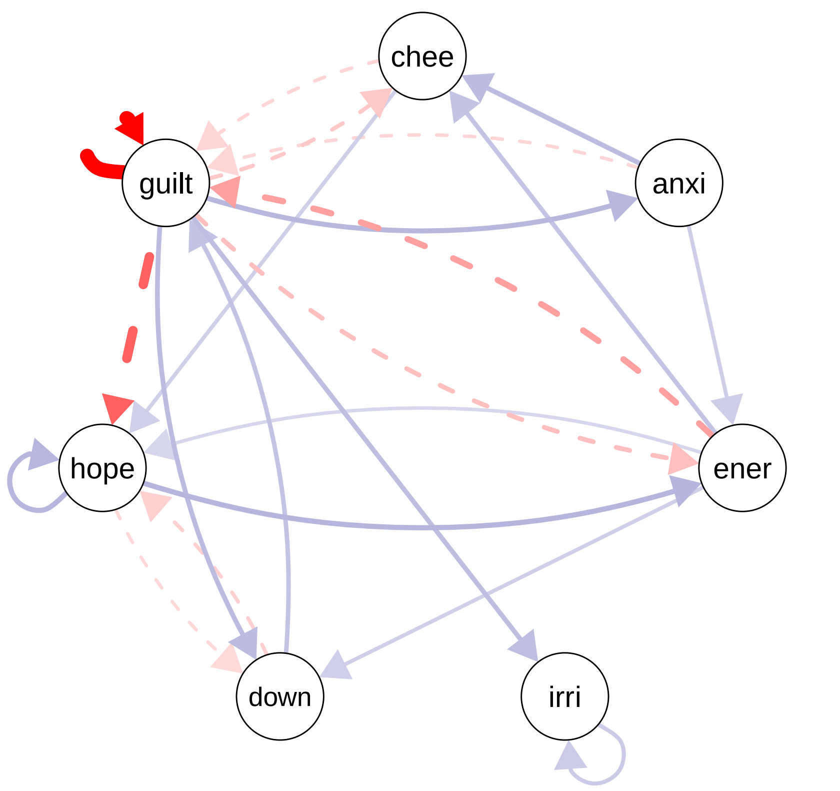

Healthy control non−reg 88JV Estpoint 8

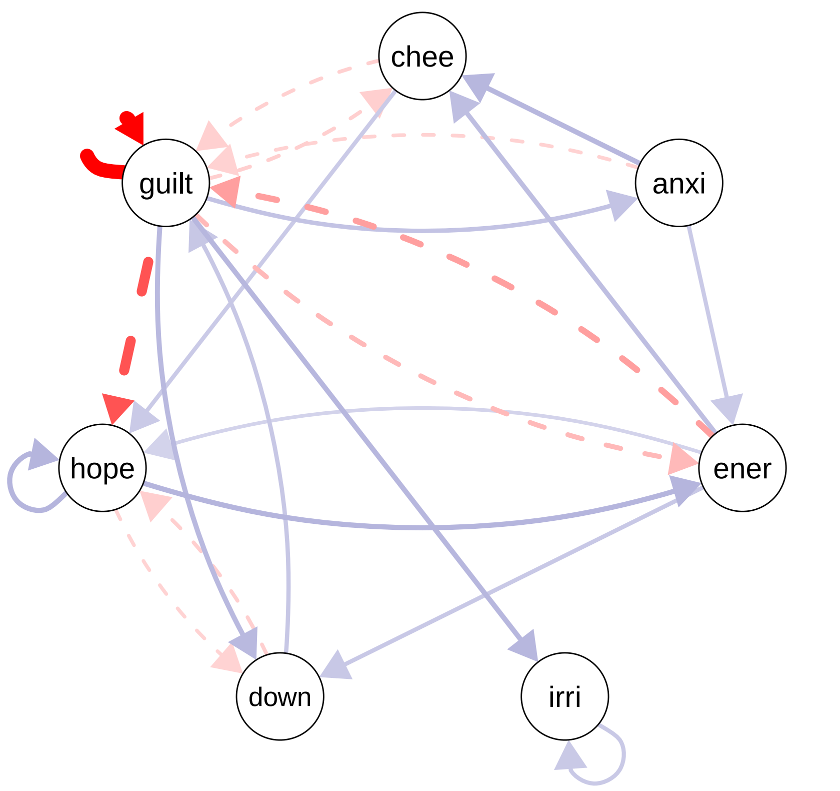

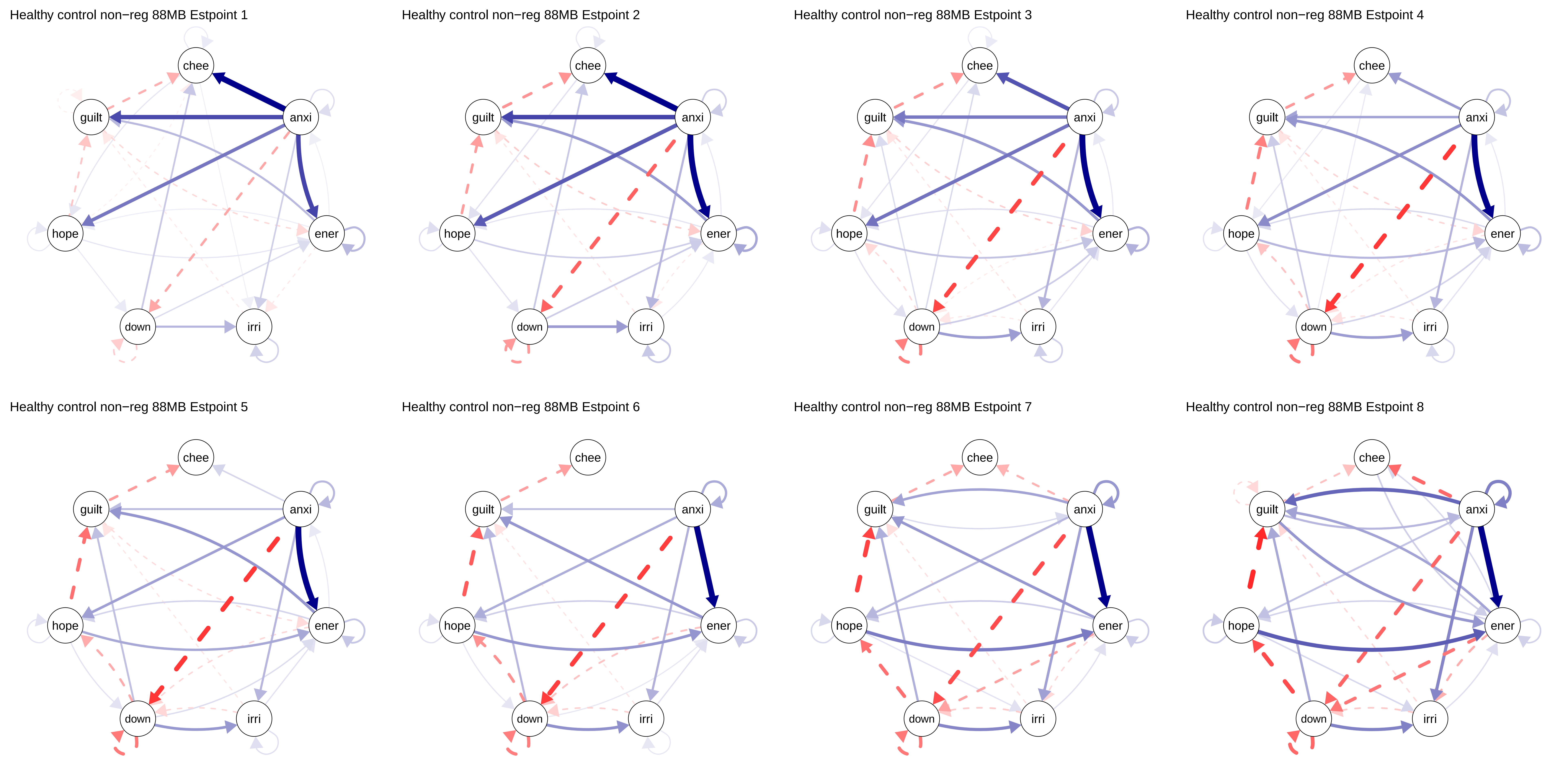
Healthy control non−reg 88MB Estpoint 1
chee guilt anxi hope ener down irri
Healthy control non−reg 88MB Estpoint 2
chee guilt anxi hope ener down irri
Healthy control non−reg 88MB Estpoint 3
chee guilt anxi hope ener down irri
Healthy control non−reg 88MB Estpoint 4
chee guilt anxi hope ener down irri
Healthy control non−reg 88MB Estpoint 5
chee guilt anxi hope ener down irri
Healthy control non−reg 88MB Estpoint 6
chee guilt anxi hope ener down irri
Healthy control non−reg 88MB Estpoint 7
chee guilt anxi hope ener down irri
Healthy control non−reg 88MB Estpoint 8
chee guilt anxi hope ener down irri

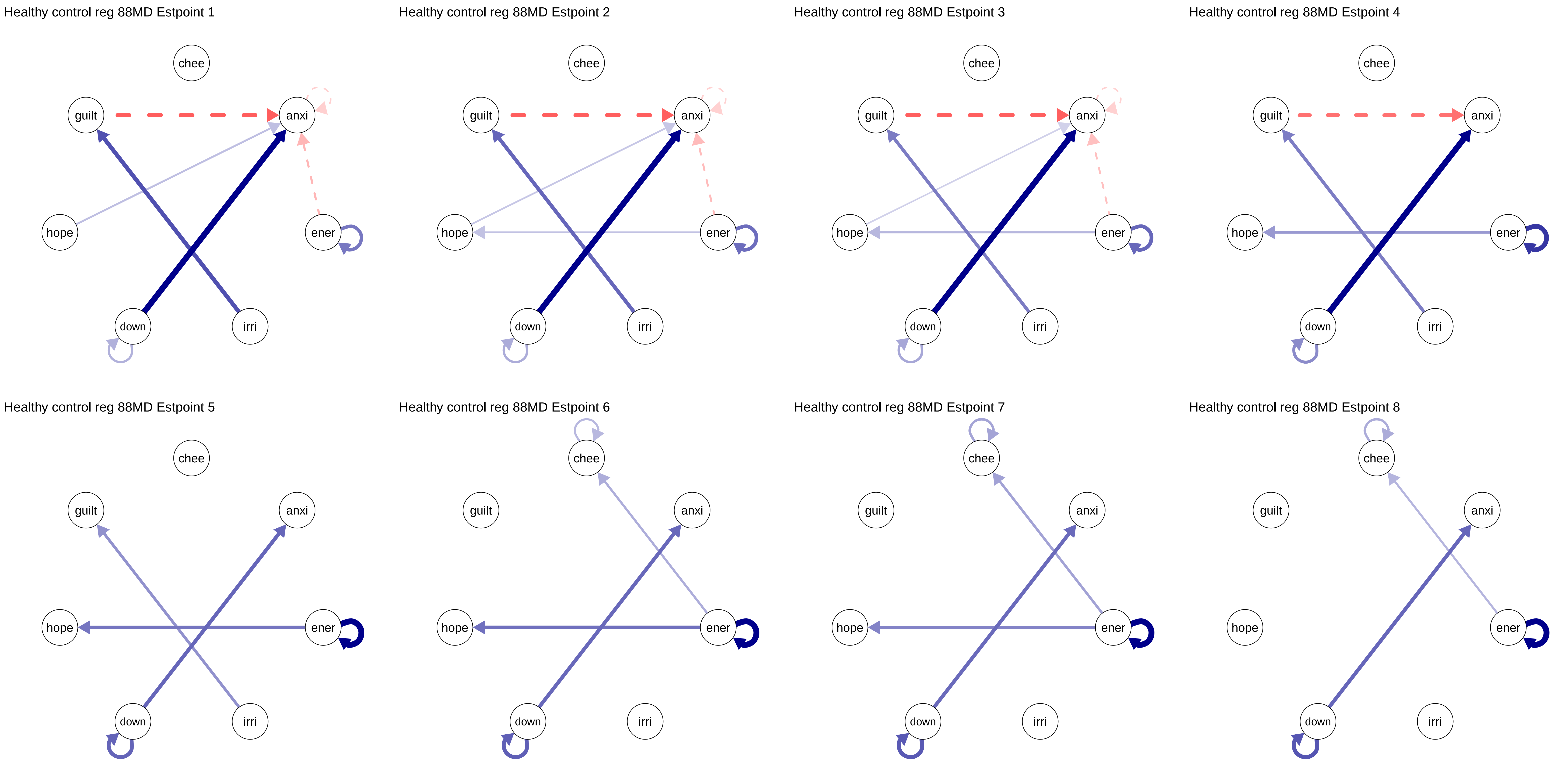
Healthy control reg 88MD Estpoint 1
Healthy control reg 88MD Estpoint 2
Healthy control reg 88MD Estpoint 3
Healthy control reg 88MD Estpoint 4
Healthy control reg 88MD Estpoint 5
Healthy control reg 88MD Estpoint 6
Healthy control reg 88MD Estpoint 7
Healthy control reg 88MD Estpoint 8
chee
guilt
anxi
hope
ener
down
irri

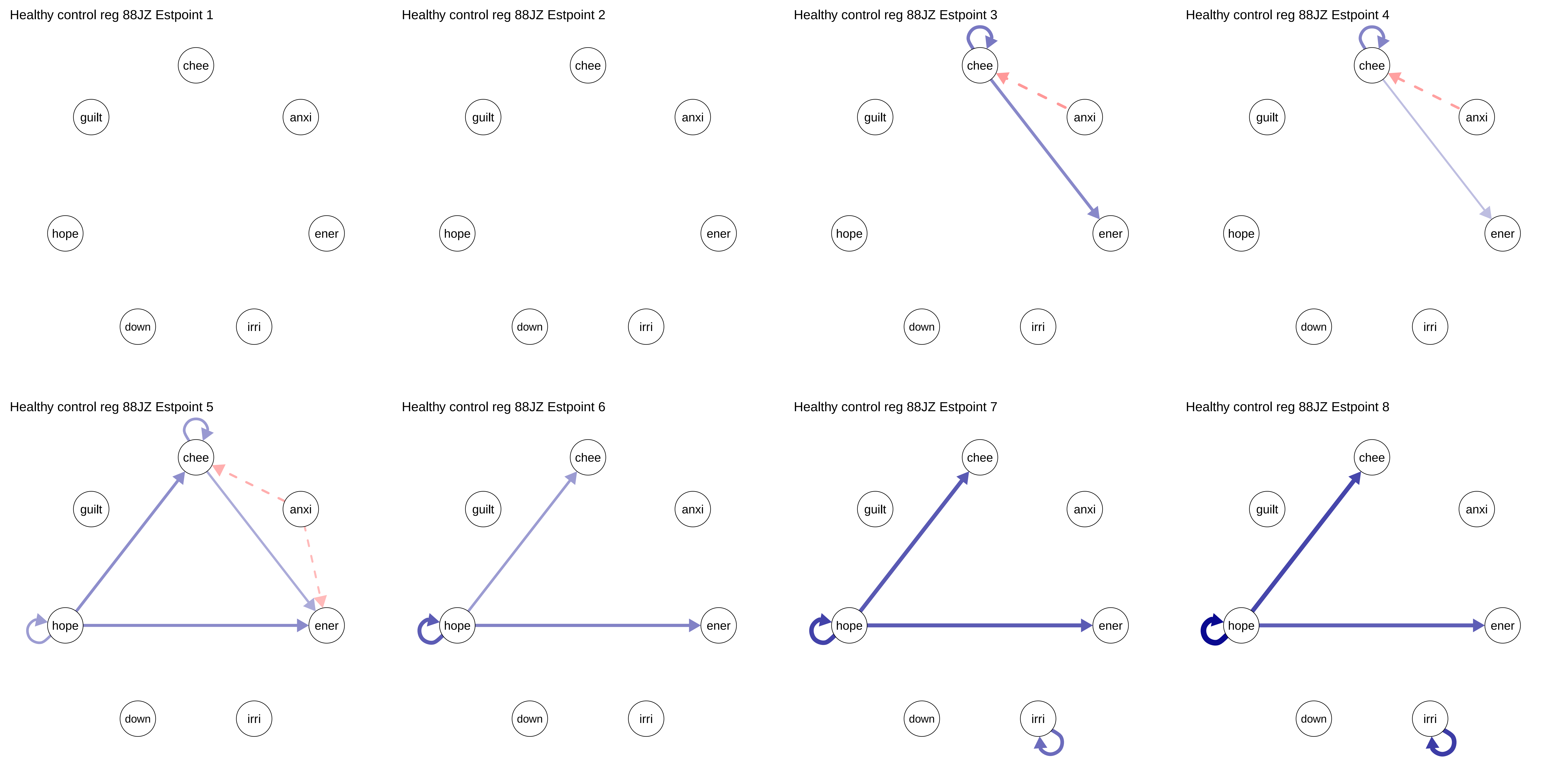
Healthy control reg 88JZ Estpoint 1
chee
guilt
anxi
hope
ener
down
irri
Healthy control reg 88JZ Estpoint 2
chee
guilt
anxi
hope
ener
down
irri
Healthy control reg 88JZ Estpoint 3
chee
guilt
anxi
hope
ener
down
irri
Healthy control reg 88JZ Estpoint 4
chee
guilt
anxi
hope
ener
down
irri
Healthy control reg 88JZ Estpoint 5
chee
guilt
anxi
hope
ener
down
irri
Healthy control reg 88JZ Estpoint 6
chee
guilt
anxi
hope
ener
down
irri
Healthy control reg 88JZ Estpoint 7
chee
guilt
anxi
hope
ener
down
irri
Healthy control reg 88JZ Estpoint 8
chee
guilt
anxi
hope
ener
down
irri

Healthy control reg 88MH Estpoint 1

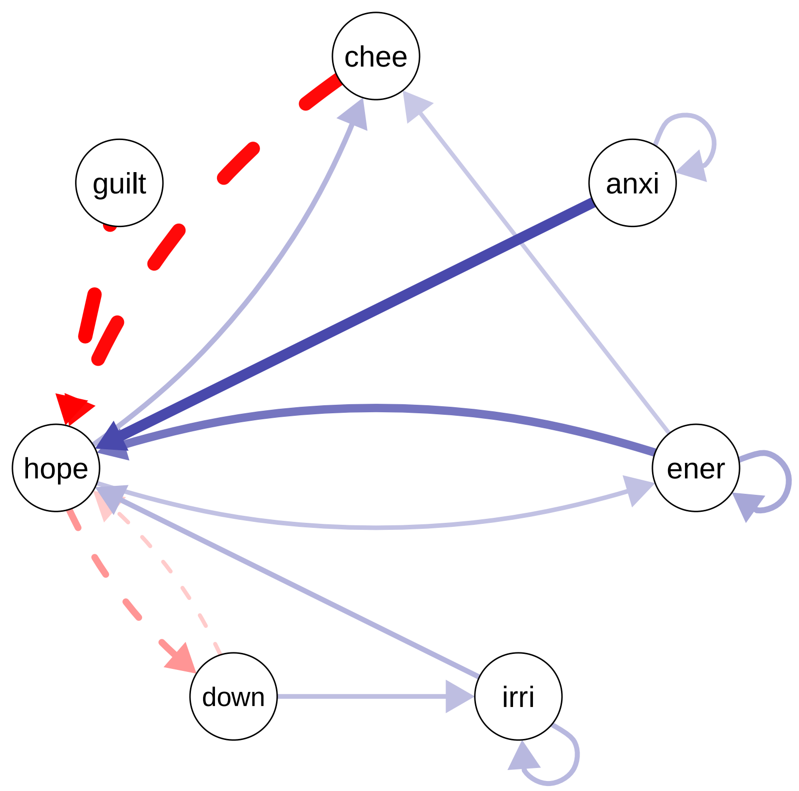

Healthy control reg 88MH Estpoint 2

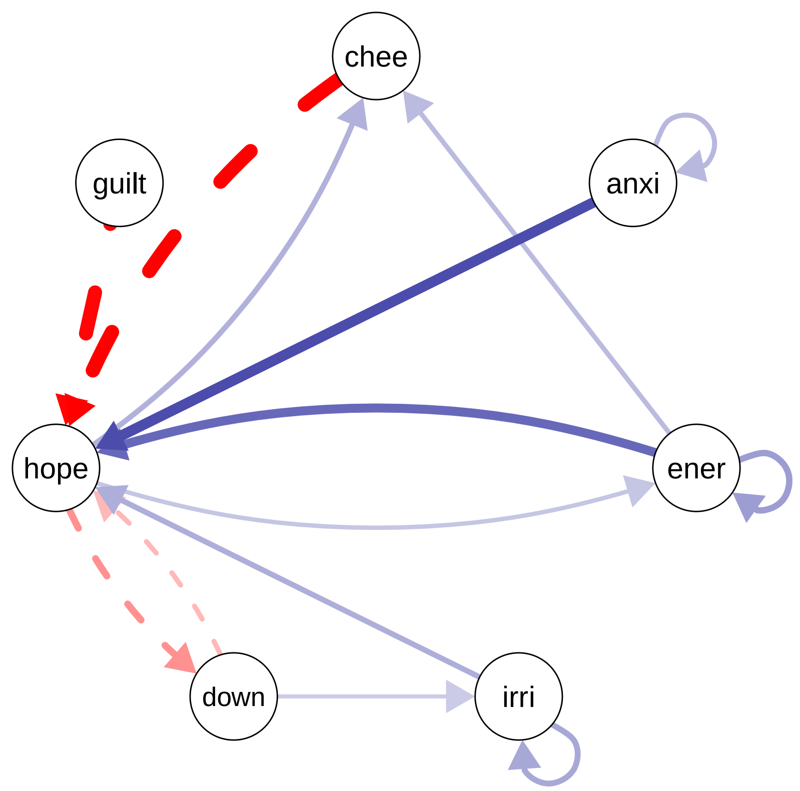

Healthy control reg 88MH Estpoint 3

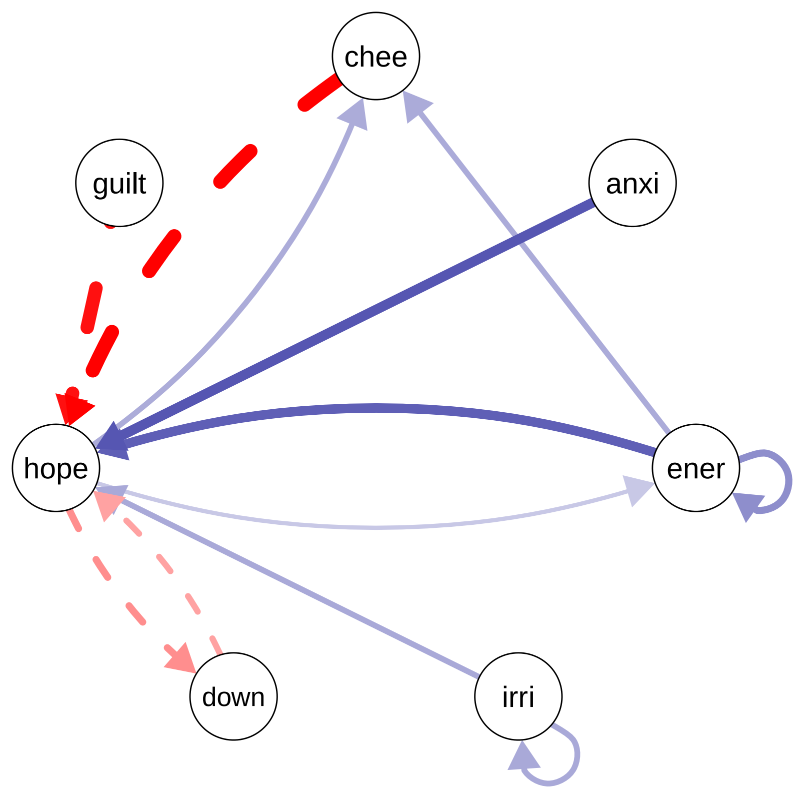

Healthy control reg 88MH Estpoint 4

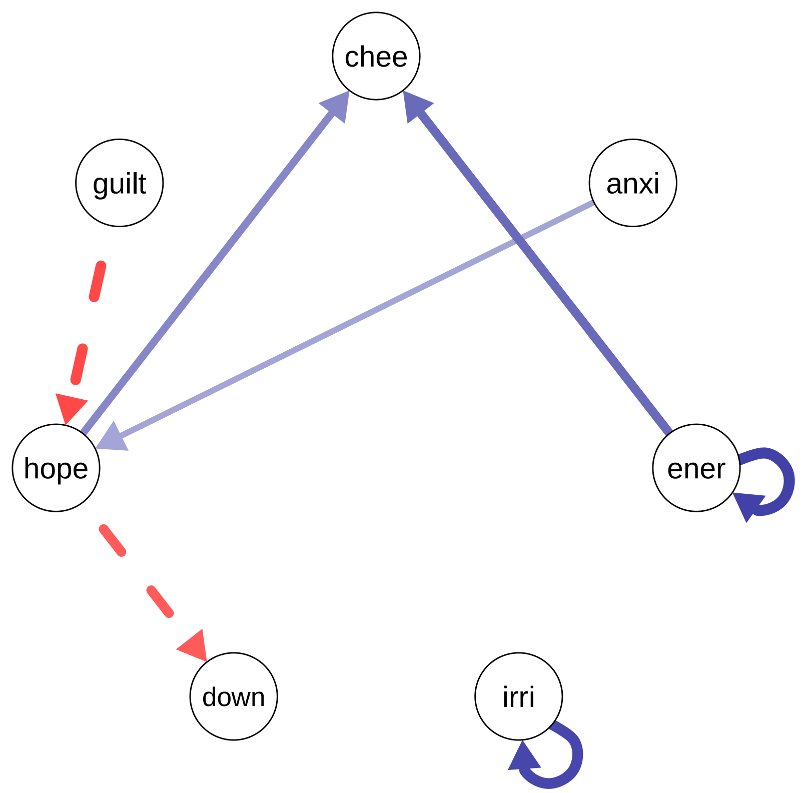

Healthy control reg 88MH Estpoint 5

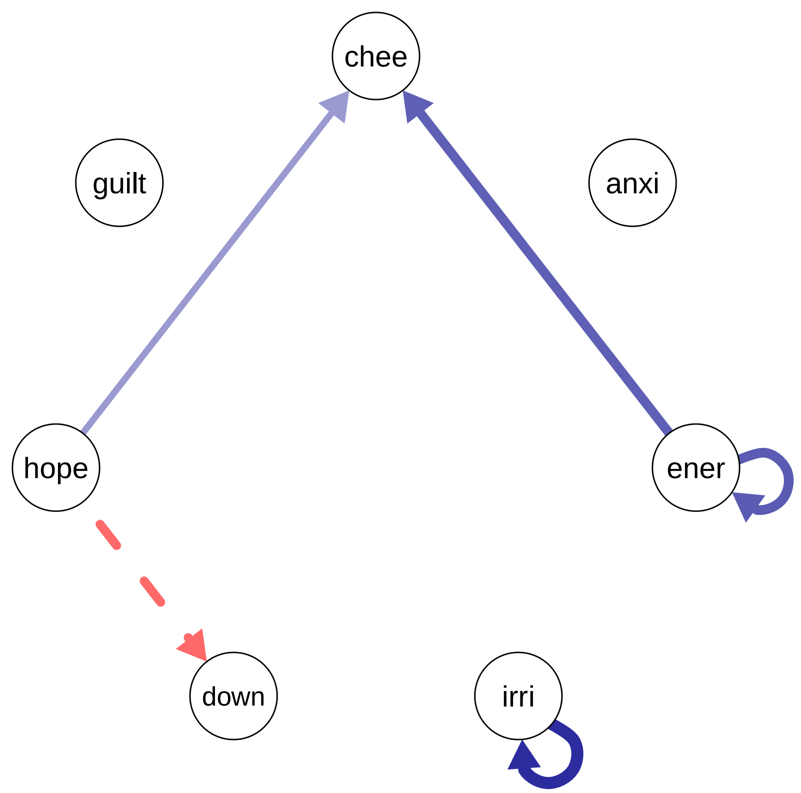

Healthy control reg 88MH Estpoint 6

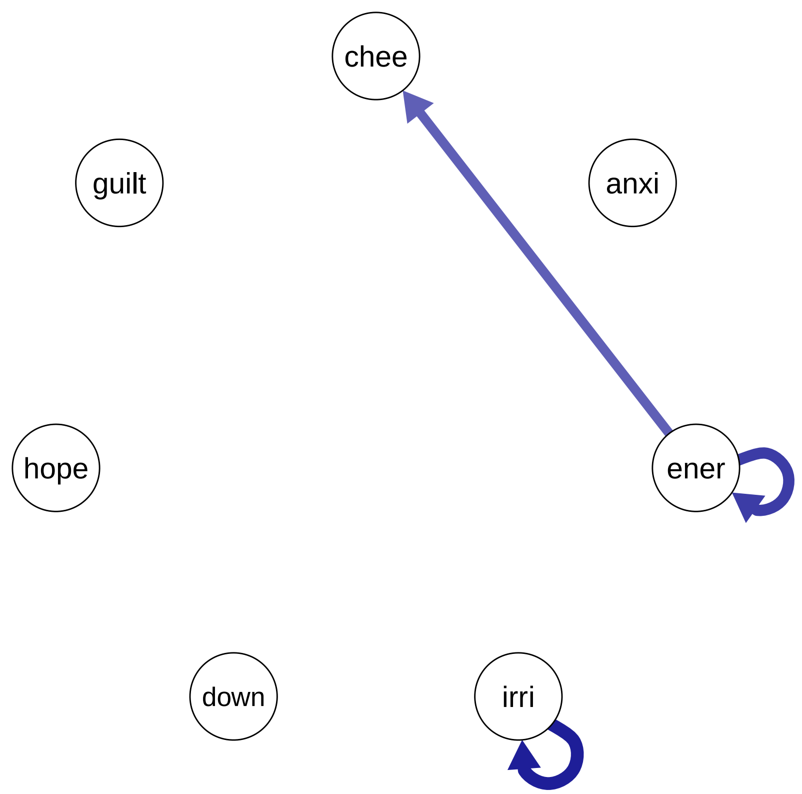

Healthy control reg 88MH Estpoint 7

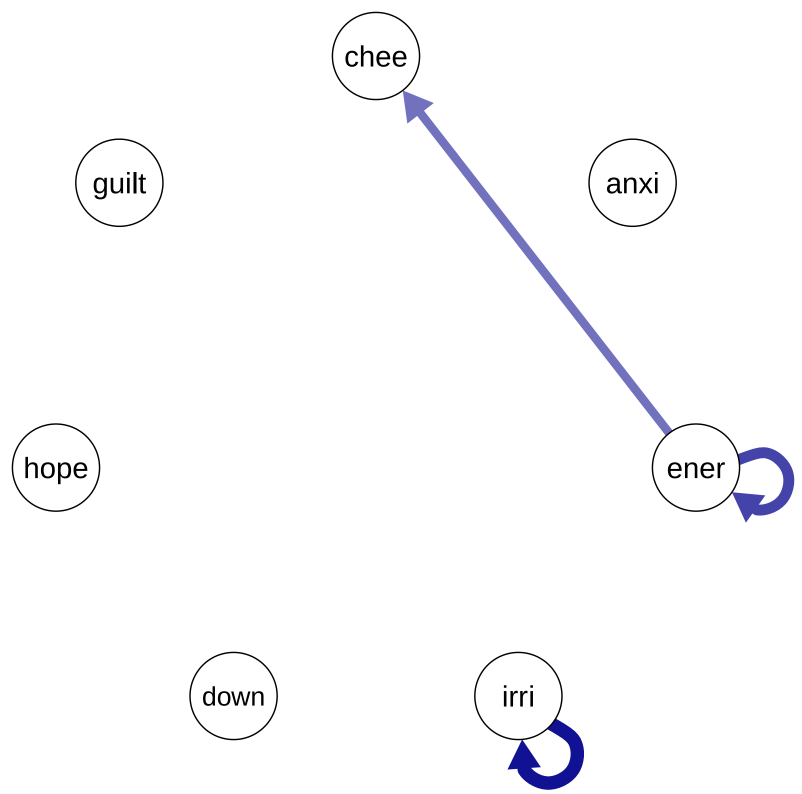

Healthy control reg 88MH Estpoint 8

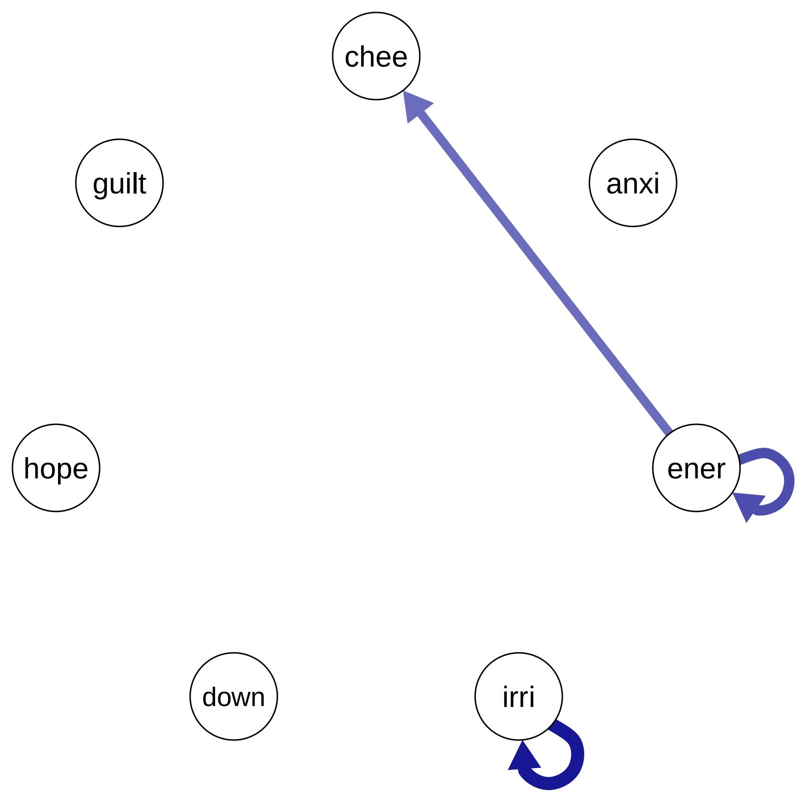

Healthy control reg 88JB Estpoint 1

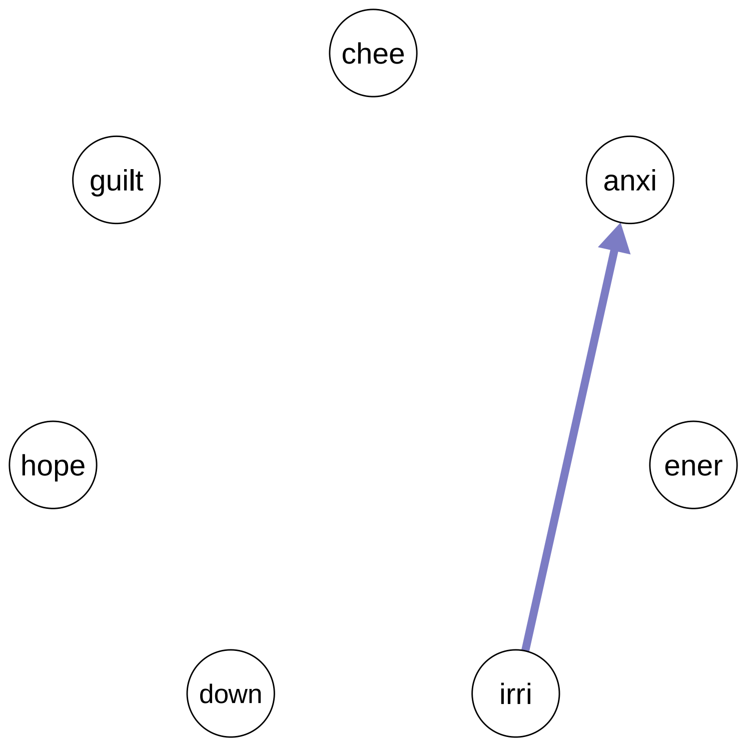

Healthy control reg 88JB Estpoint 2

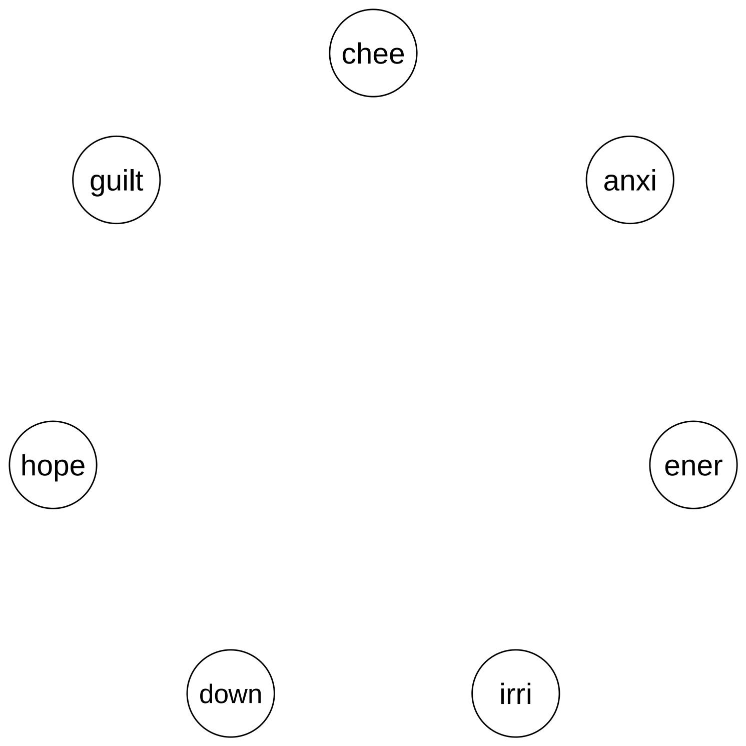

Healthy control reg 88JB Estpoint 3

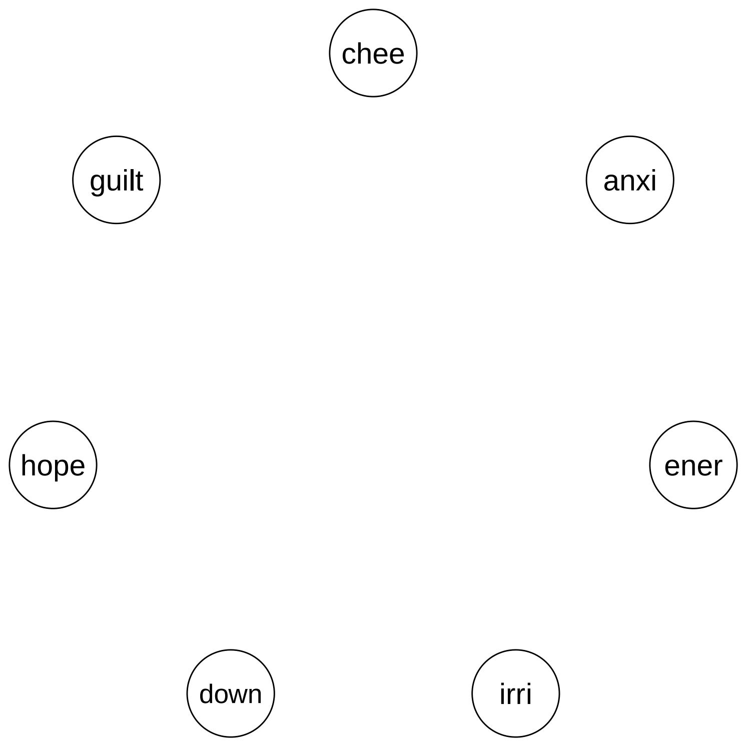

Healthy control reg 88JB Estpoint 4

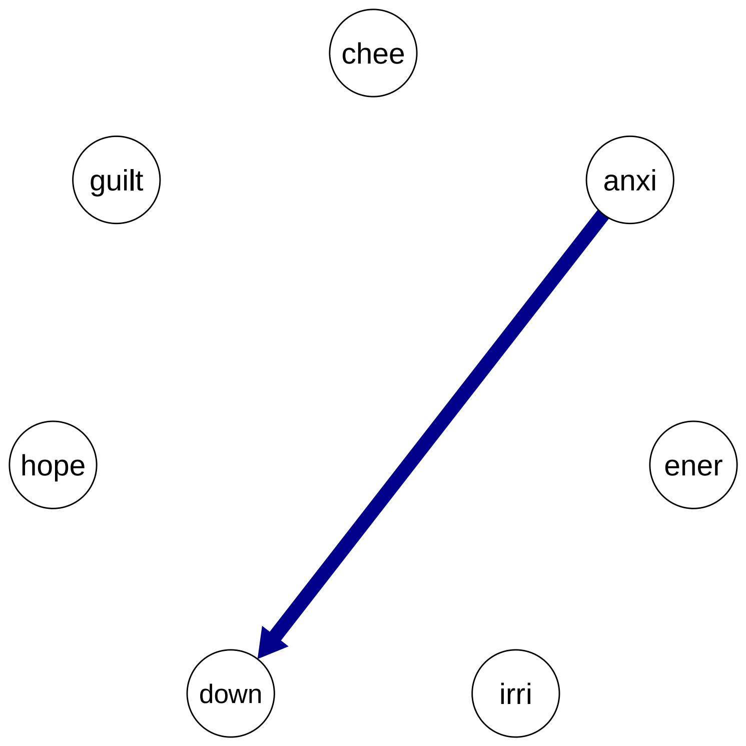

Healthy control reg 88JB Estpoint 5

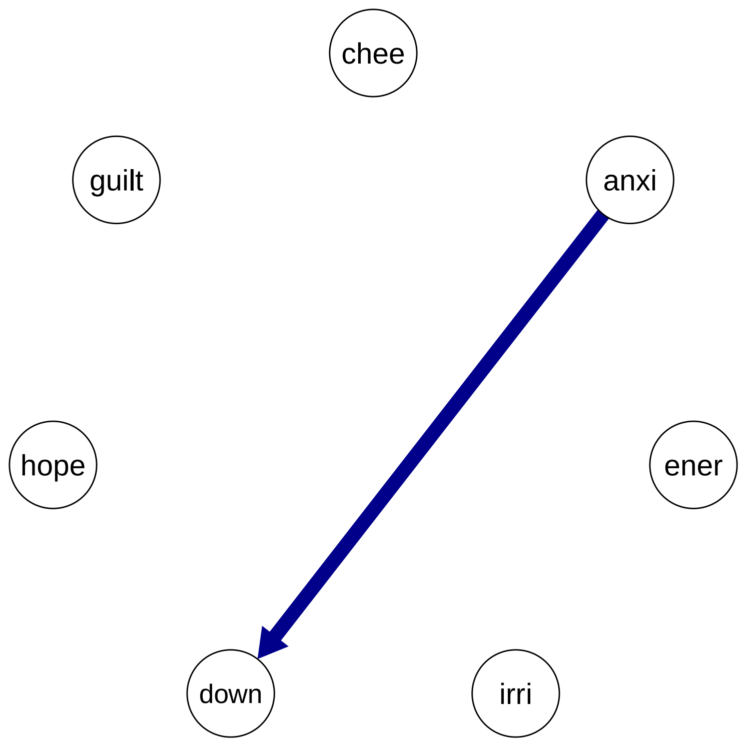

Healthy control reg 88JB Estpoint 6

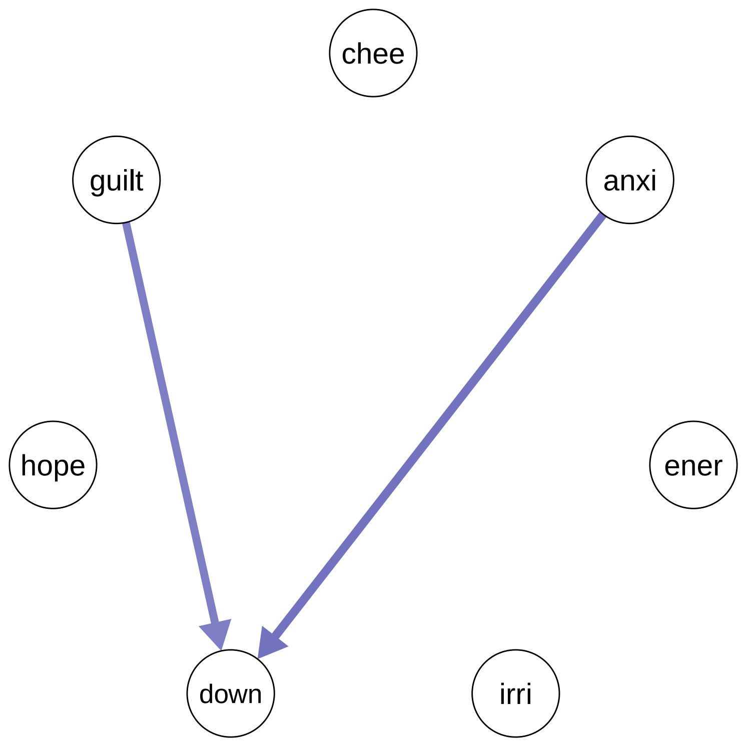

Healthy control reg 88JB Estpoint 7

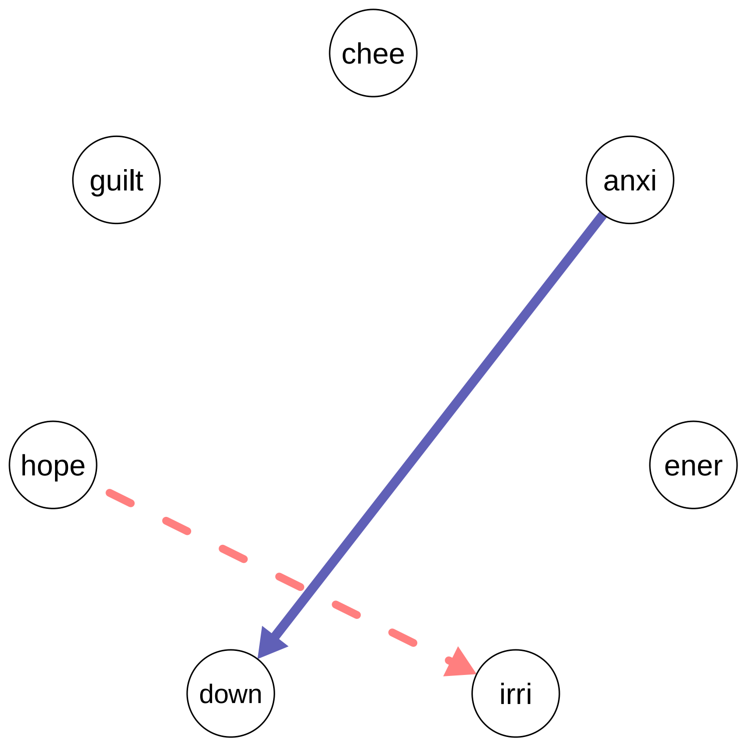

Healthy control reg 88JB Estpoint 8

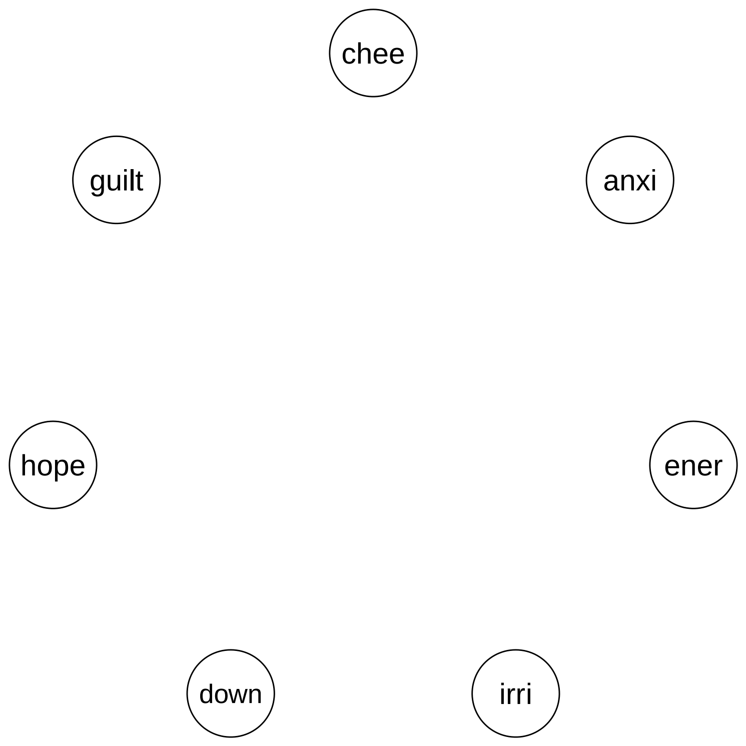

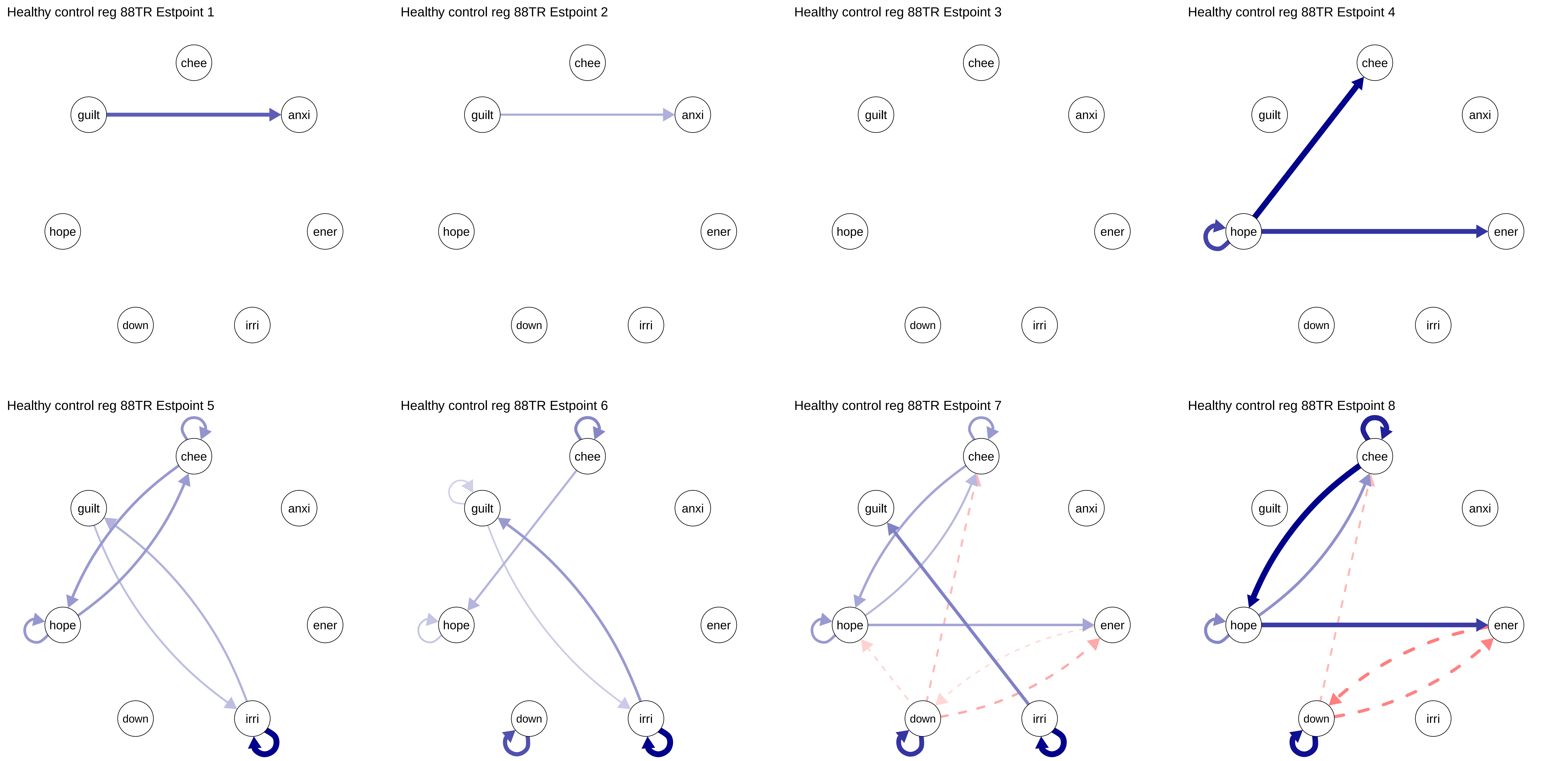
Healthy control reg 88TR Estpoint 1
Healthy control reg 88TR Estpoint 2
Healthy control reg 88TR Estpoint 3
Healthy control reg 88TR Estpoint 4
Healthy control reg 88TR Estpoint 5
Healthy control reg 88TR Estpoint 6
Healthy control reg 88TR Estpoint 7
Healthy control reg 88TR Estpoint 8
chee
guilt
anxi
hope
ener
down
irri

Healthy control reg 88FB Estpoint 1

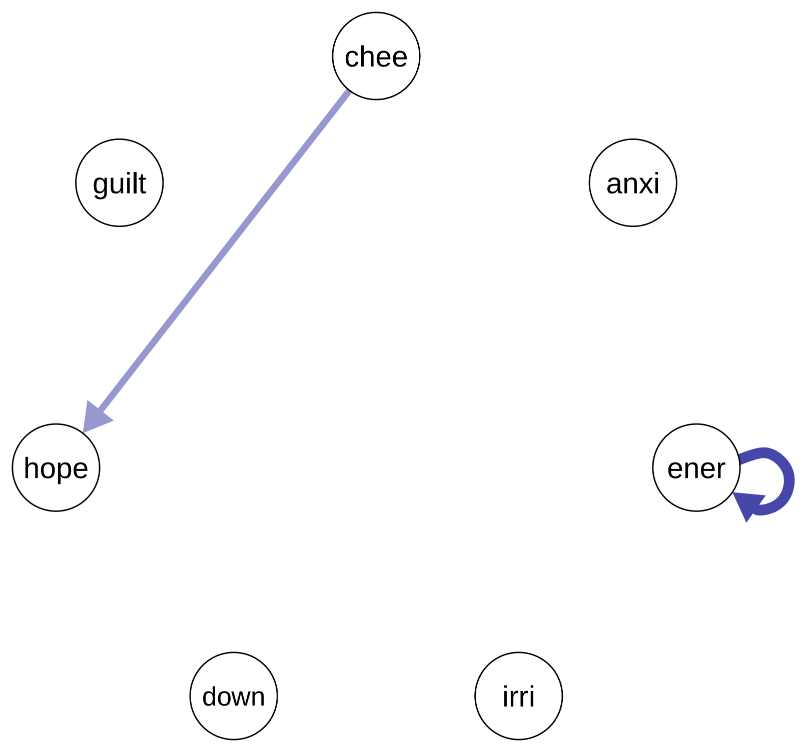

Healthy control reg 88FB Estpoint 2

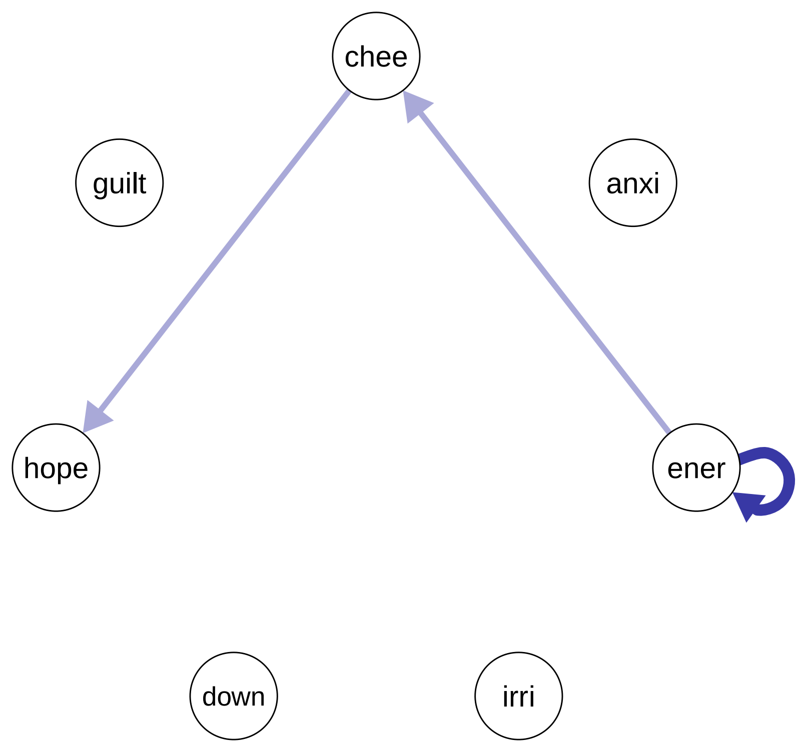

Healthy control reg 88FB Estpoint 3

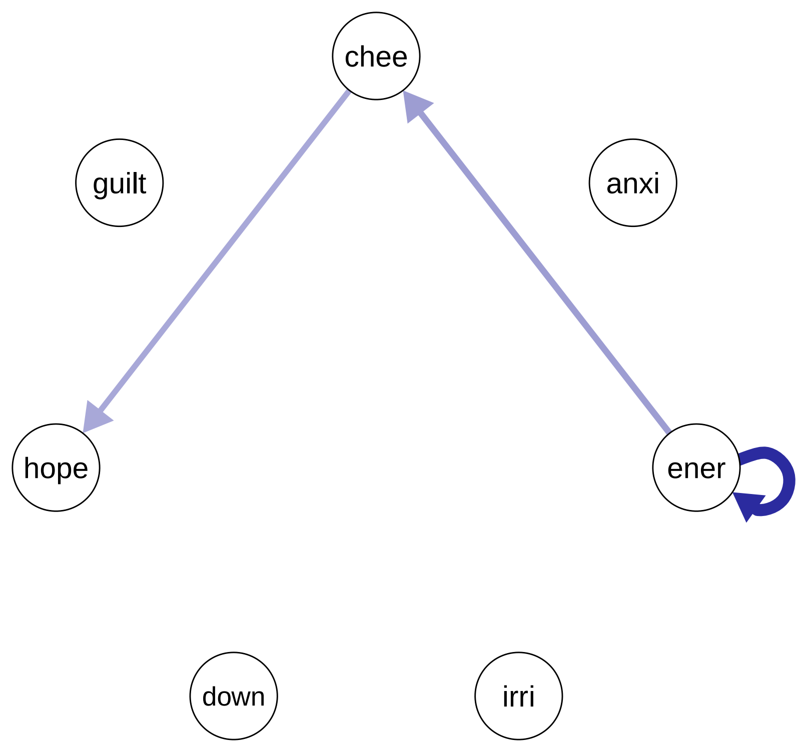

Healthy control reg 88FB Estpoint 4

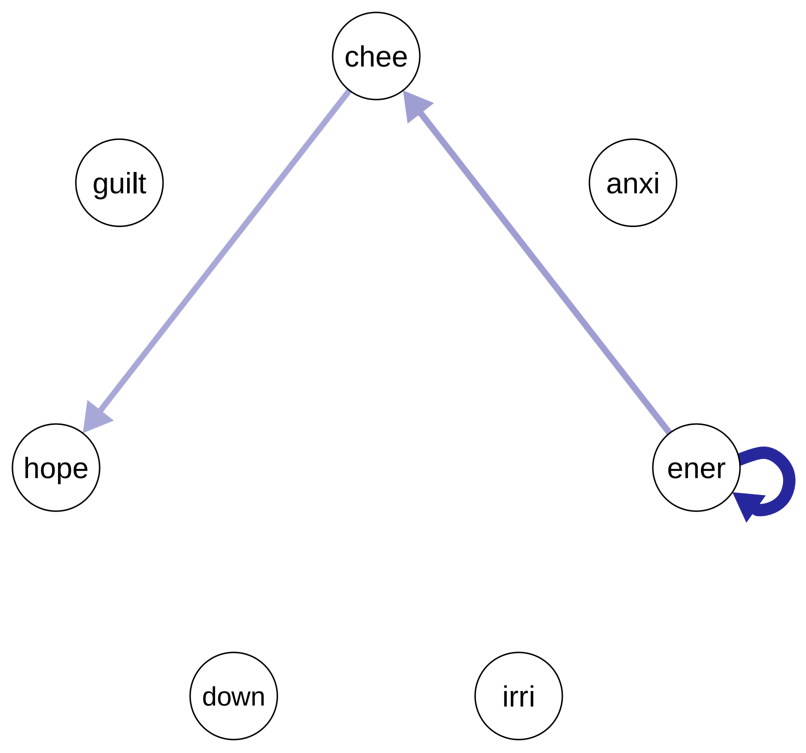

Healthy control reg 88FB Estpoint 5

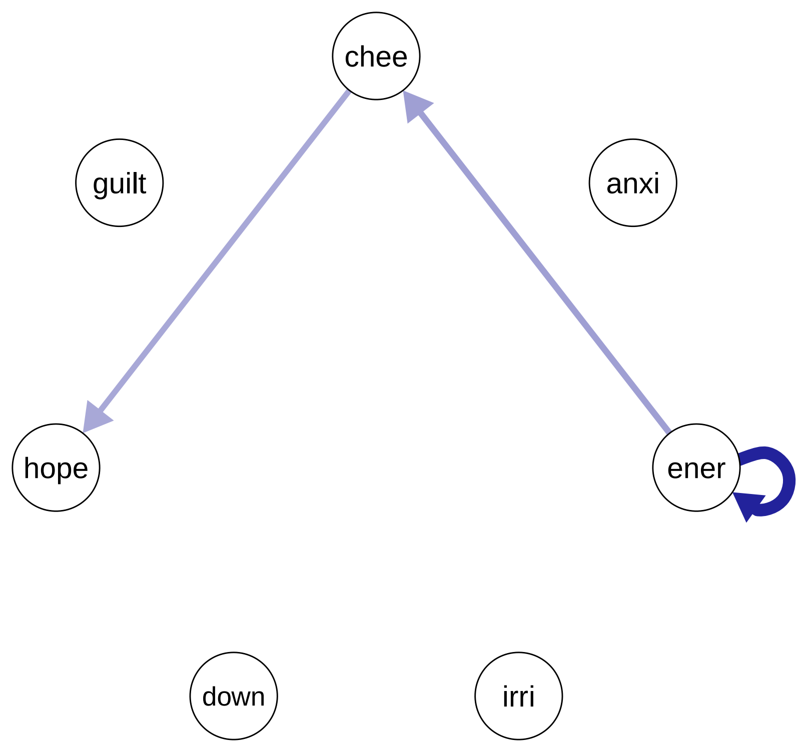

Healthy control reg 88FB Estpoint 6

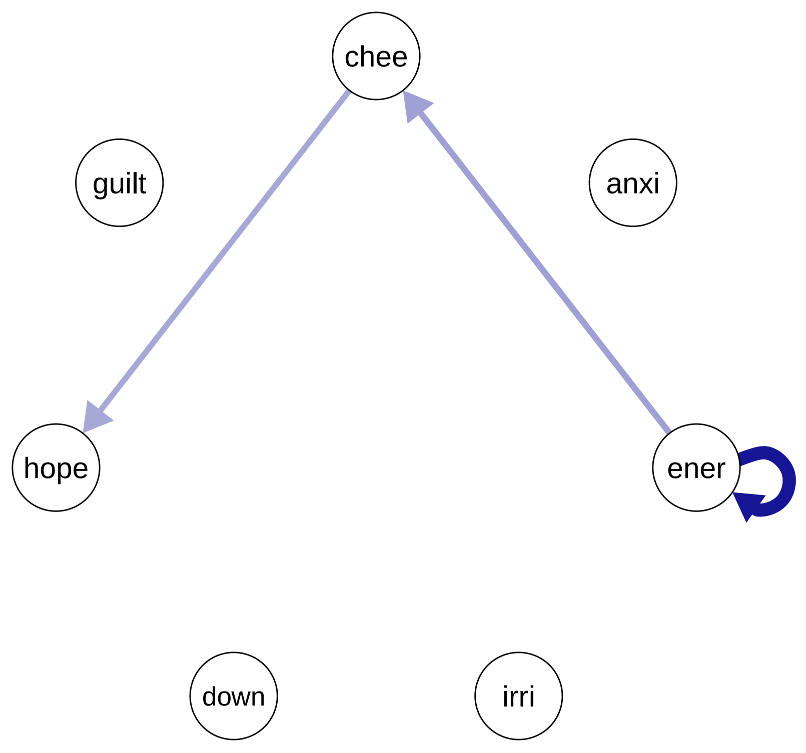

Healthy control reg 88FB Estpoint 7

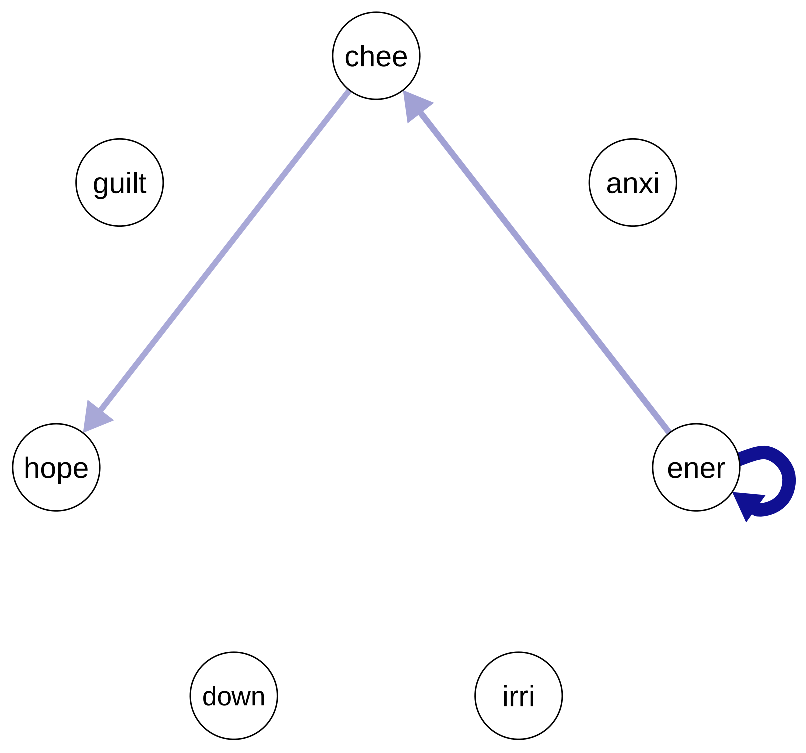

Healthy control reg 88FB Estpoint 8

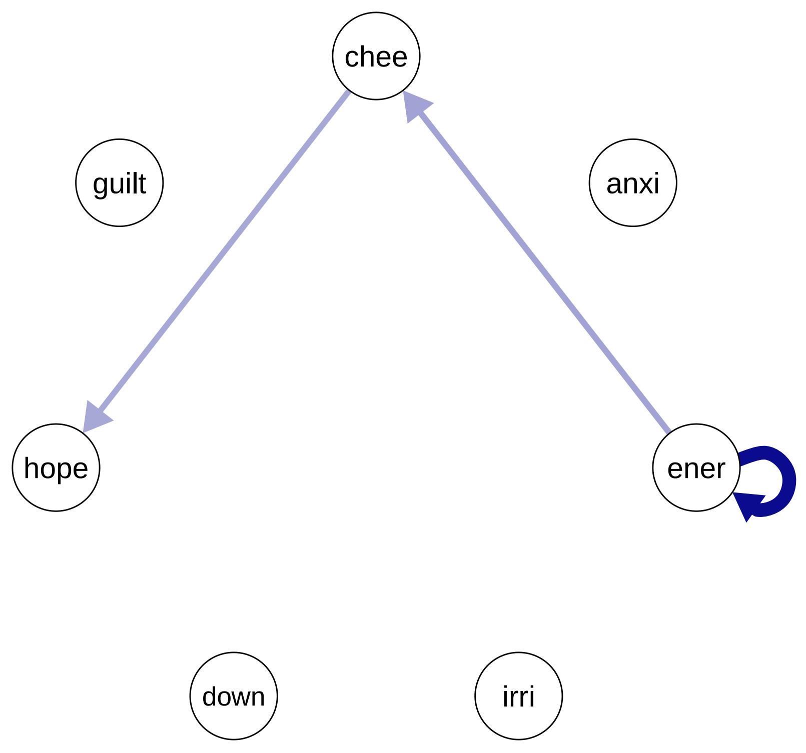

Healthy control reg 88LJ Estpoint 1

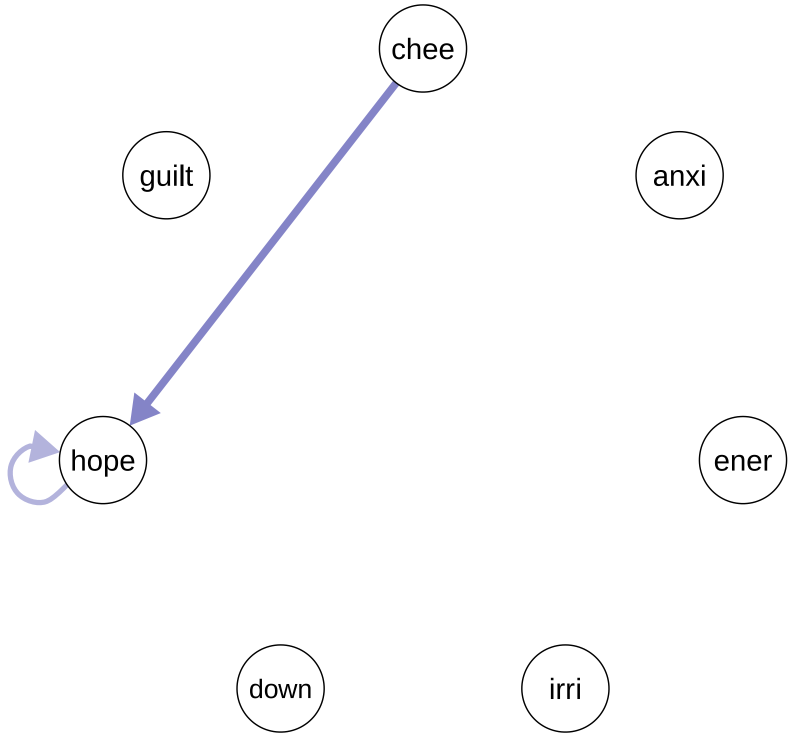

Healthy control reg 88LJ Estpoint 2

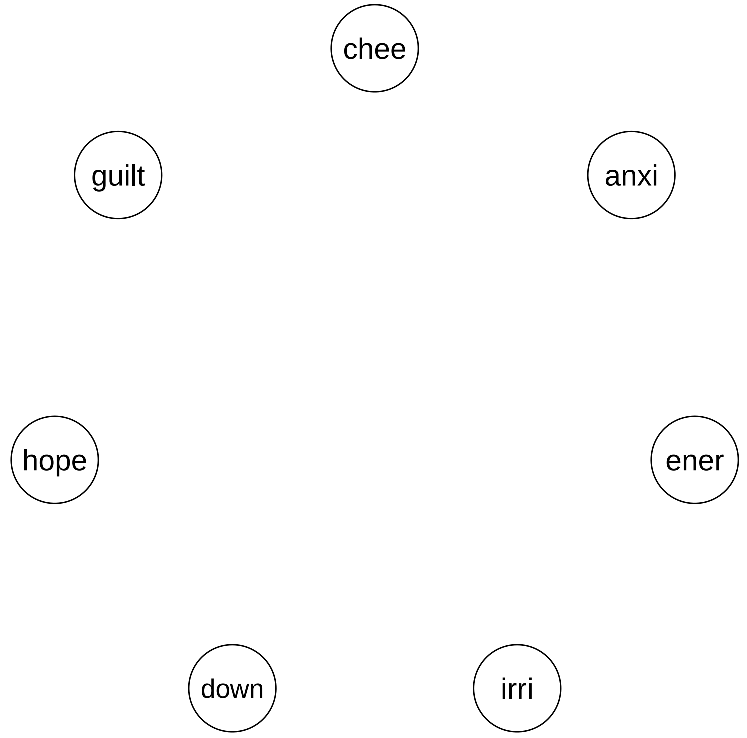

Healthy control reg 88LJ Estpoint 3

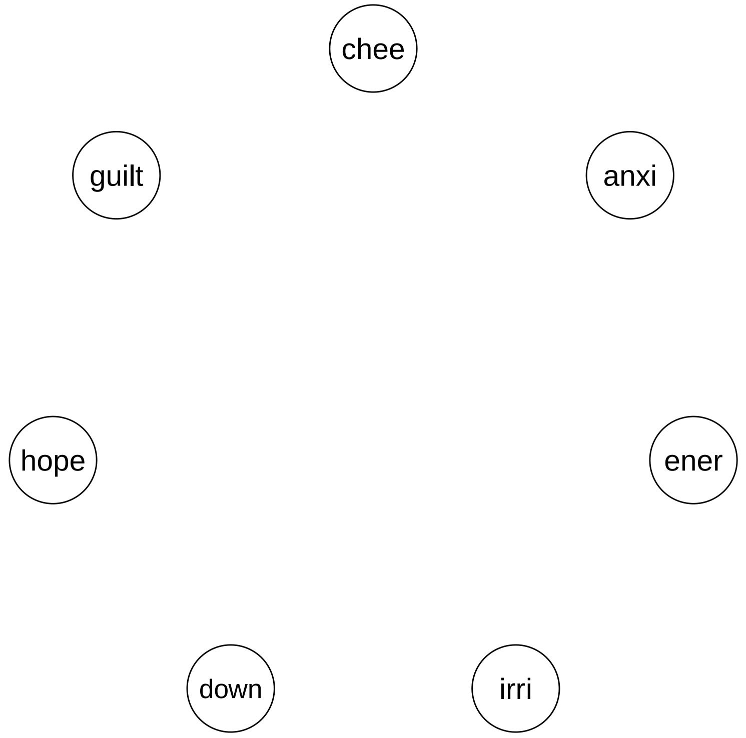

Healthy control reg 88LJ Estpoint 4

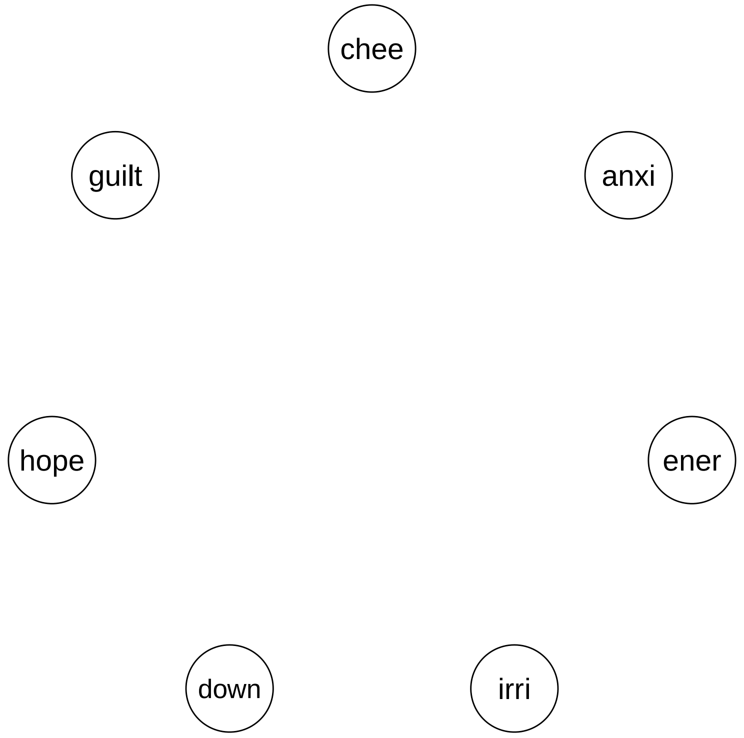

Healthy control reg 88LJ Estpoint 5

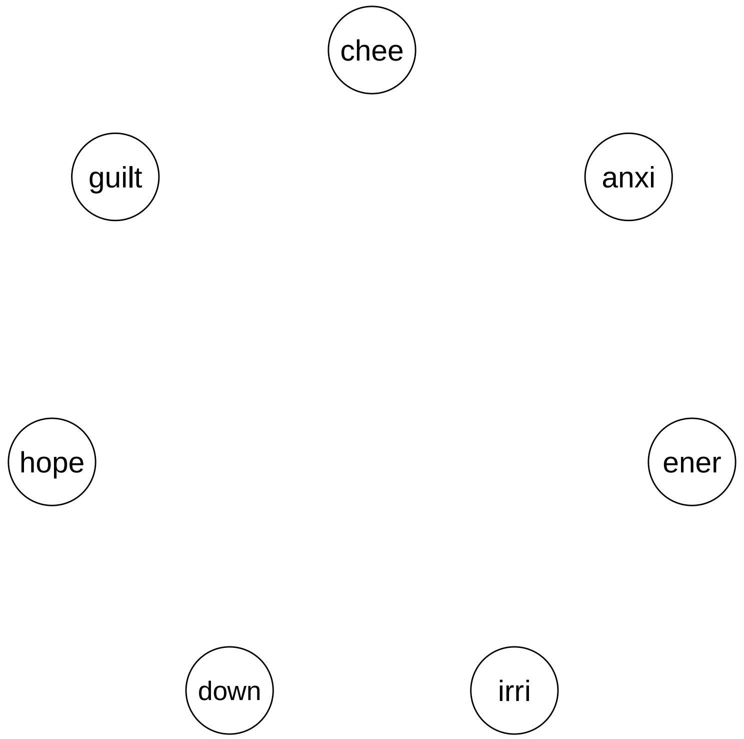

Healthy control reg 88LJ Estpoint 6

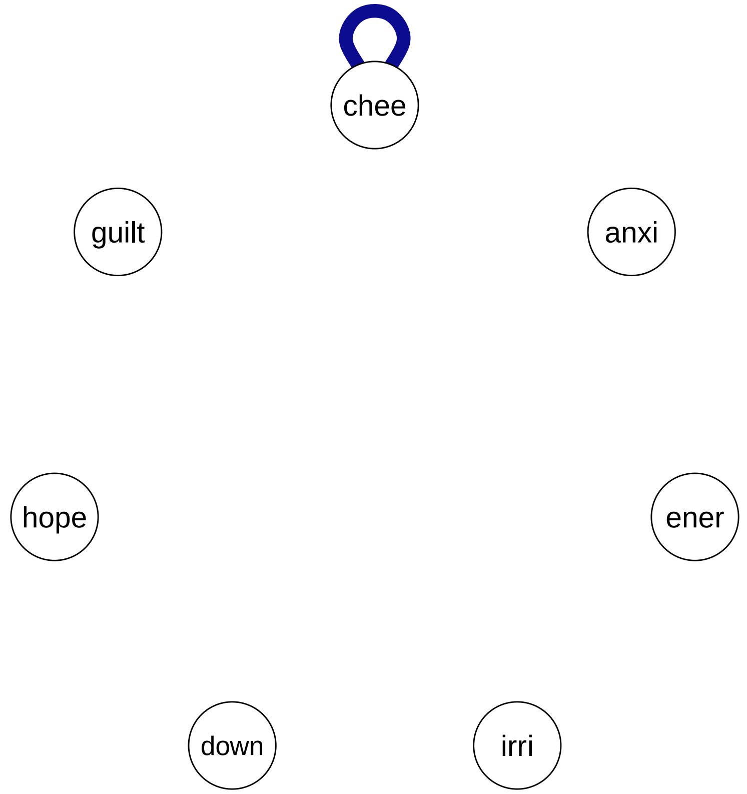

Healthy control reg 88LJ Estpoint 7

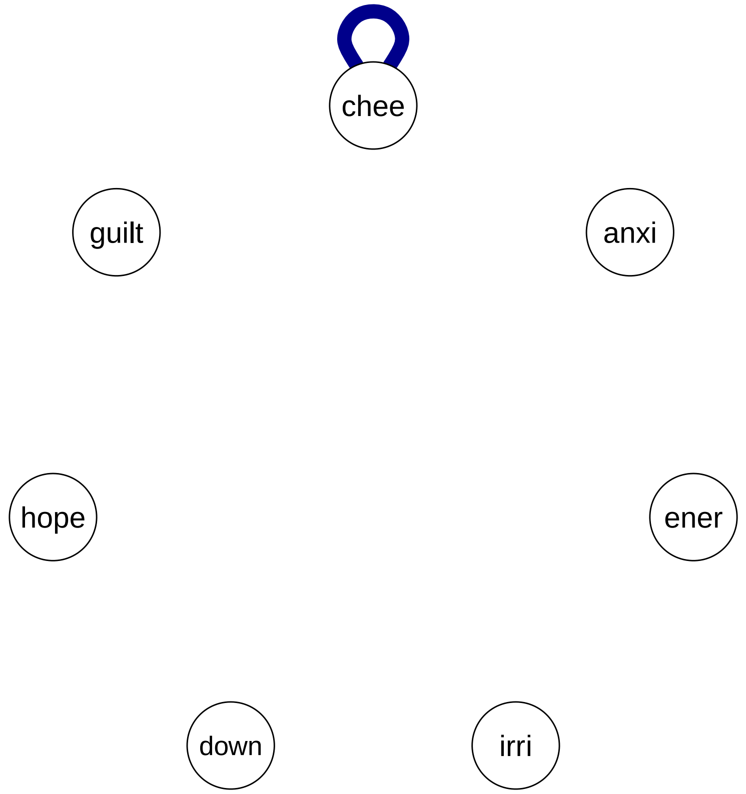

Healthy control reg 88LJ Estpoint 8

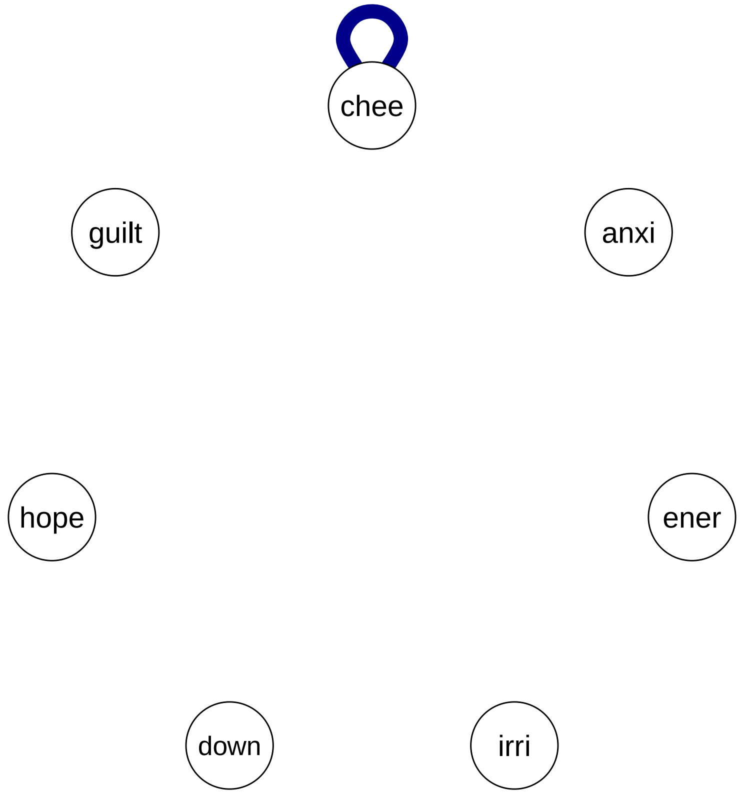

Healthy control reg 88MHB Estpoint 1

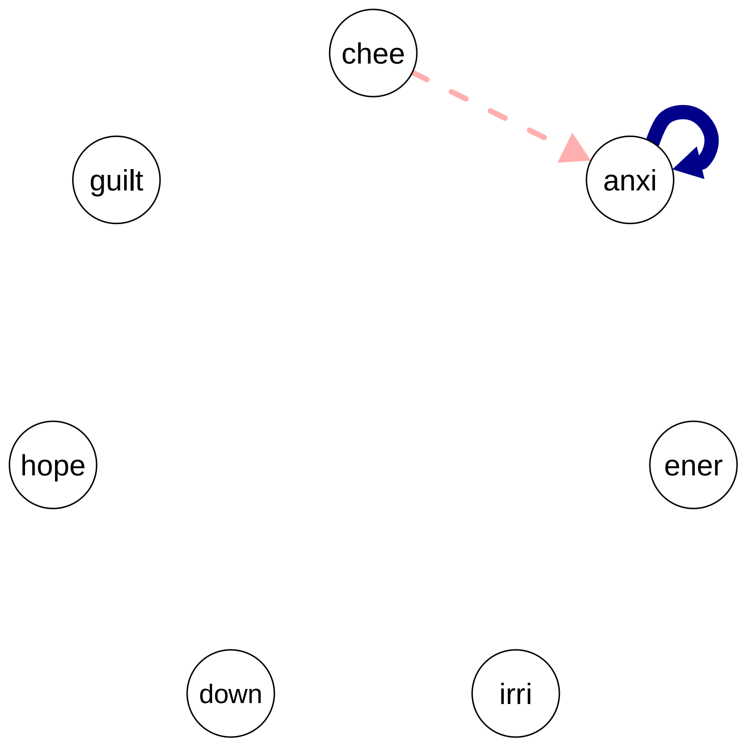

Healthy control reg 88MHB Estpoint 2

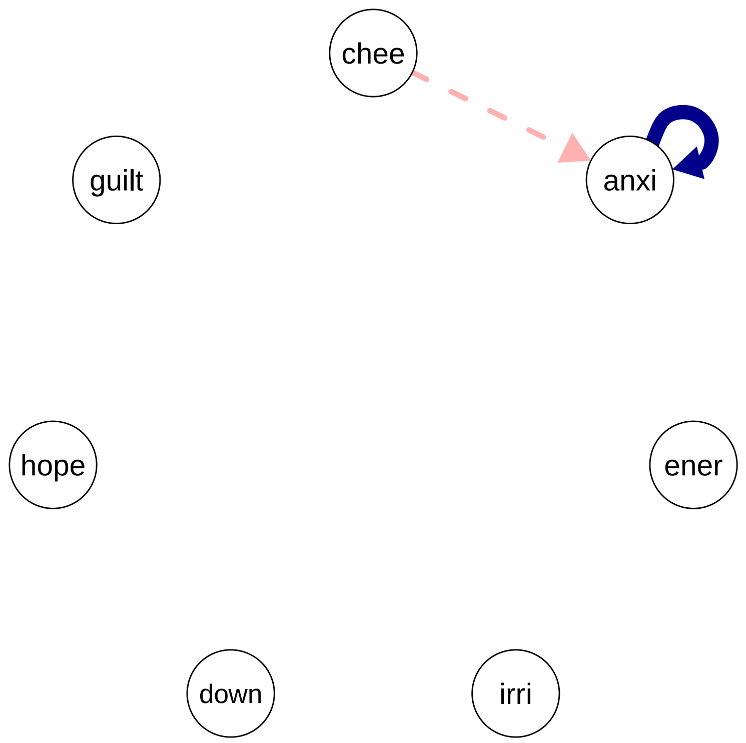

Healthy control reg 88MHB Estpoint 3

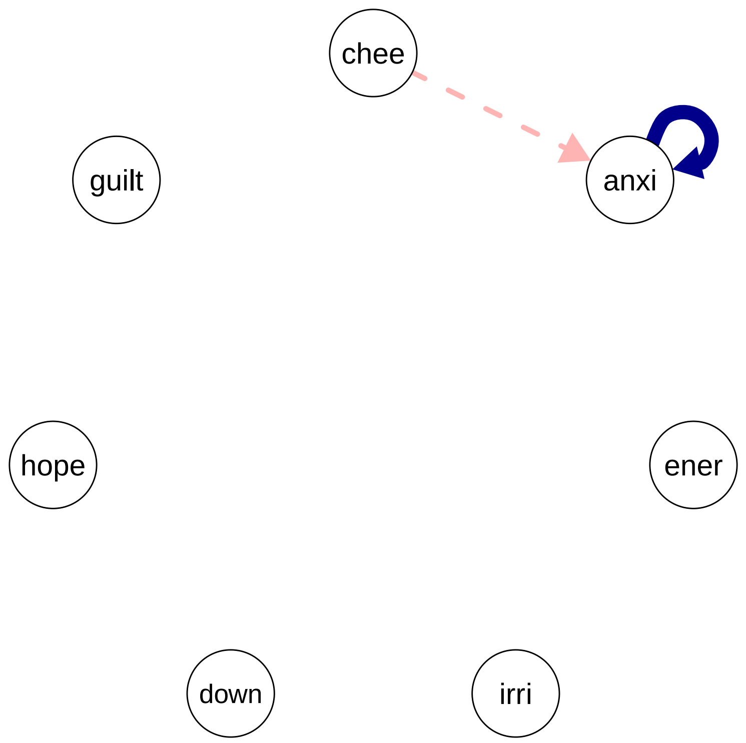

Healthy control reg 88MHB Estpoint 4

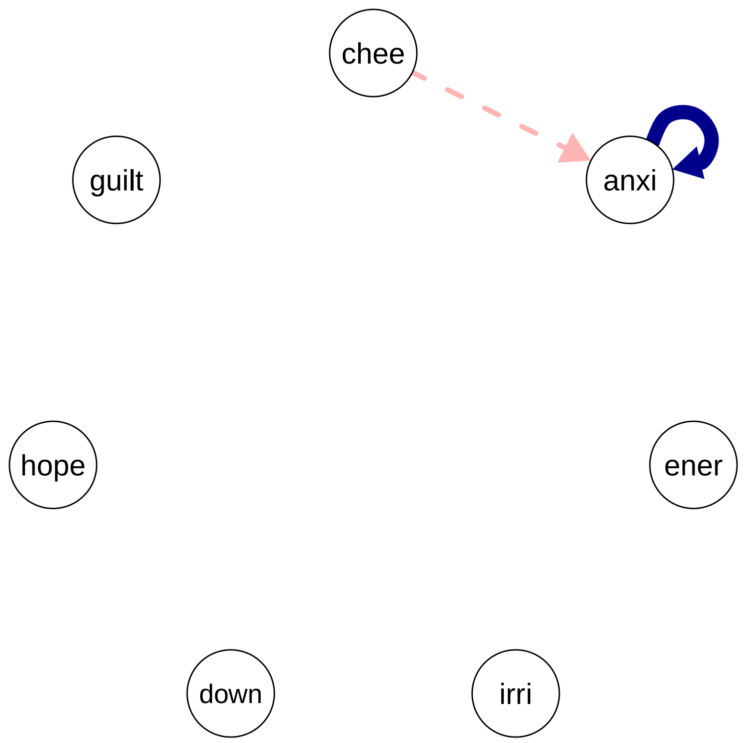

Healthy control reg 88MHB Estpoint 5

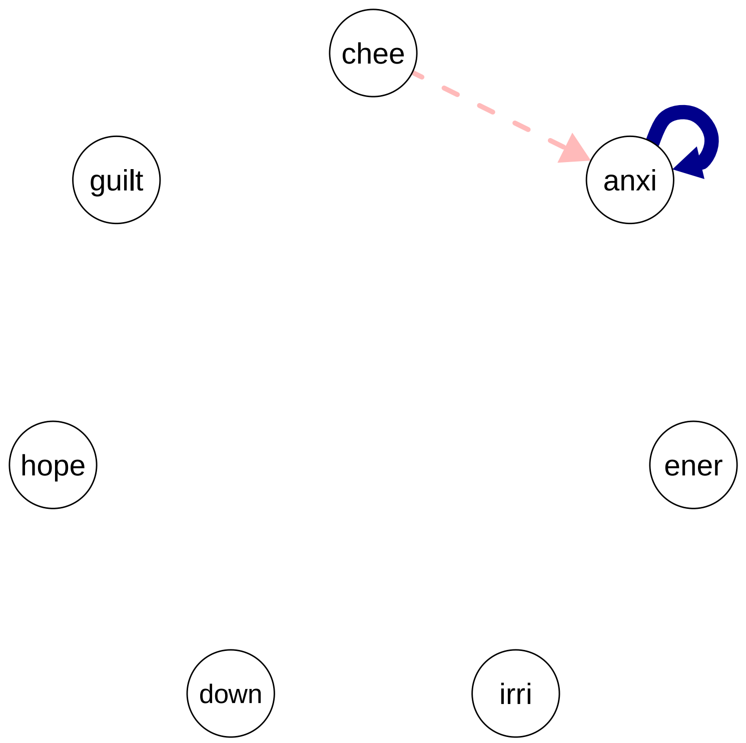

Healthy control reg 88MHB Estpoint 6

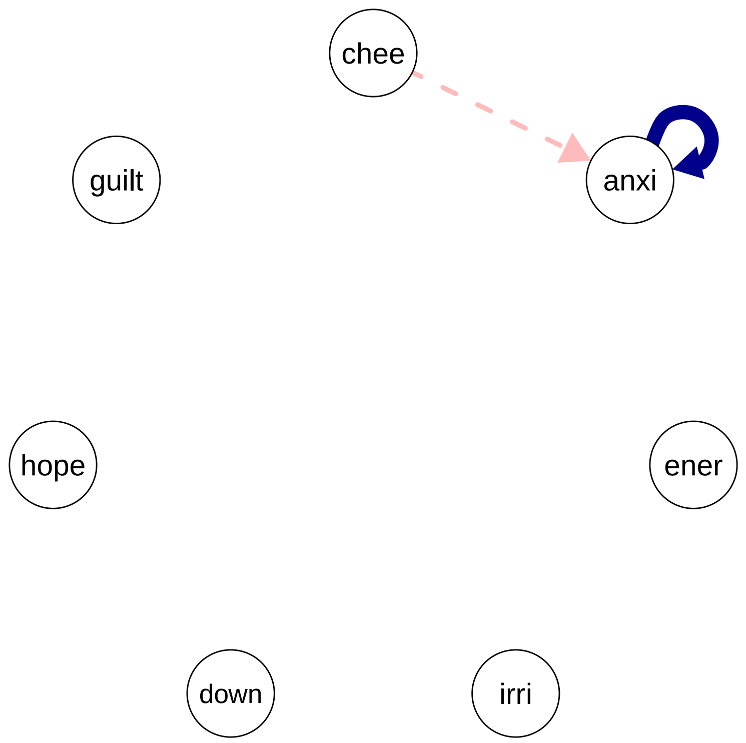

Healthy control reg 88MHB Estpoint 7

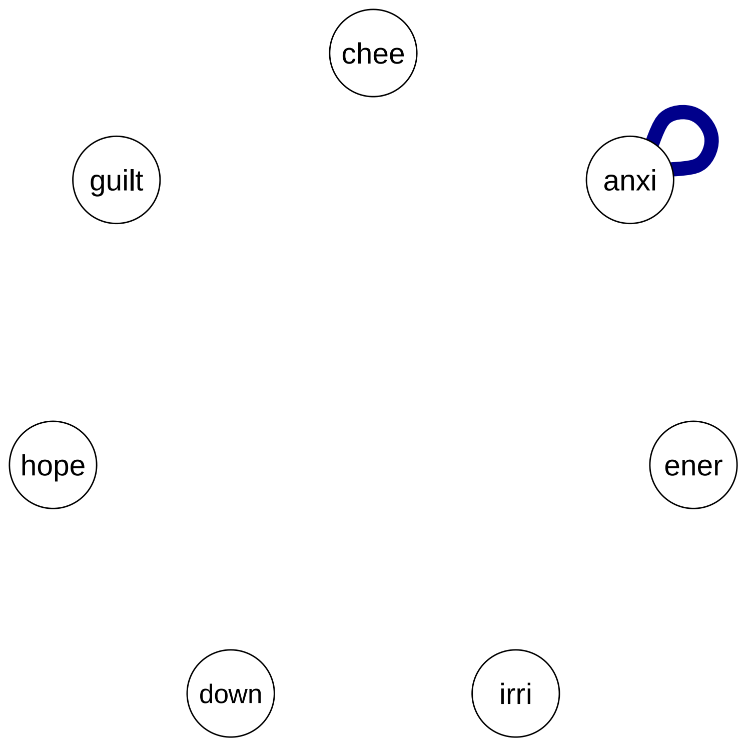

Healthy control reg 88MHB Estpoint 8

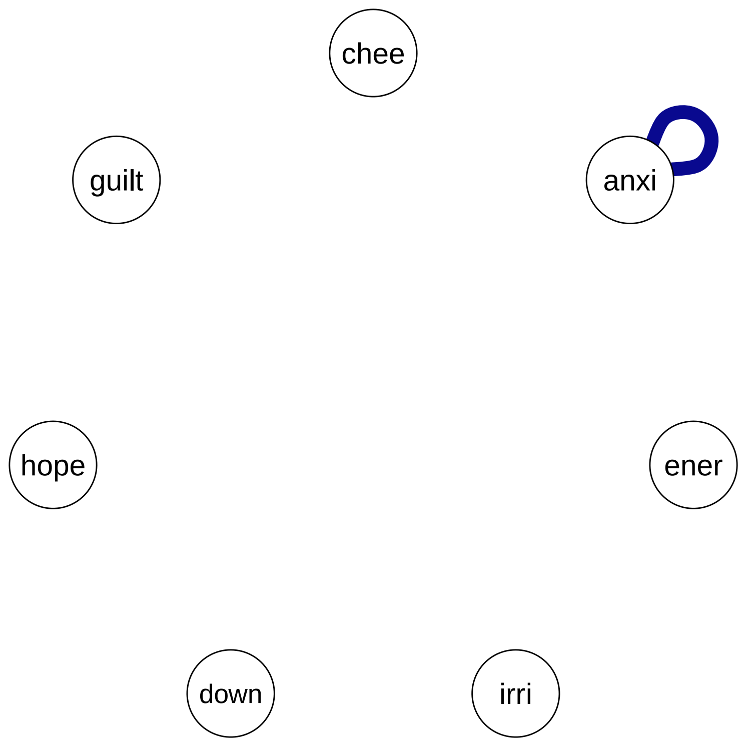

Healthy control reg 88SS Estpoint 1

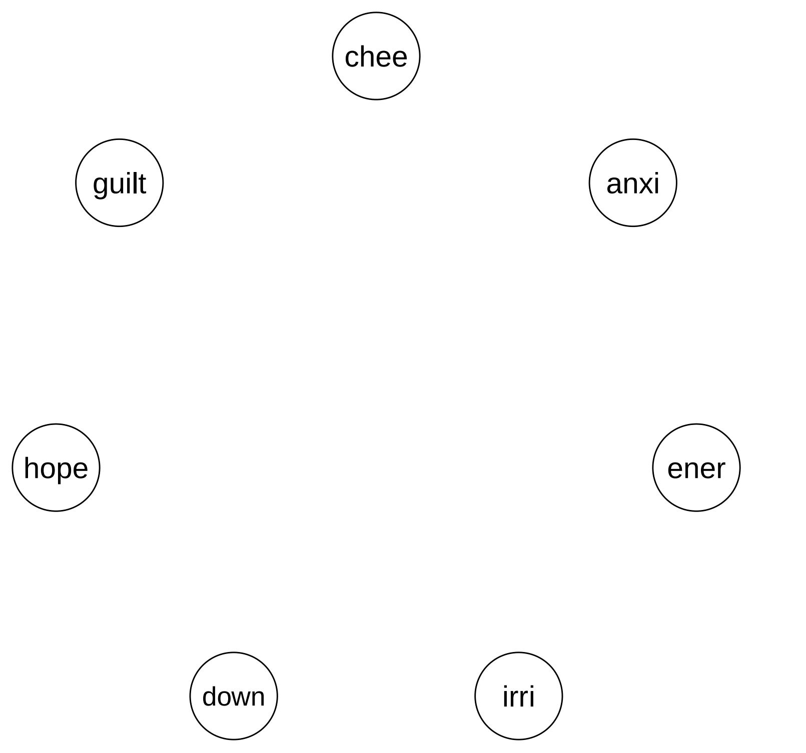

Healthy control reg 88SS Estpoint 2

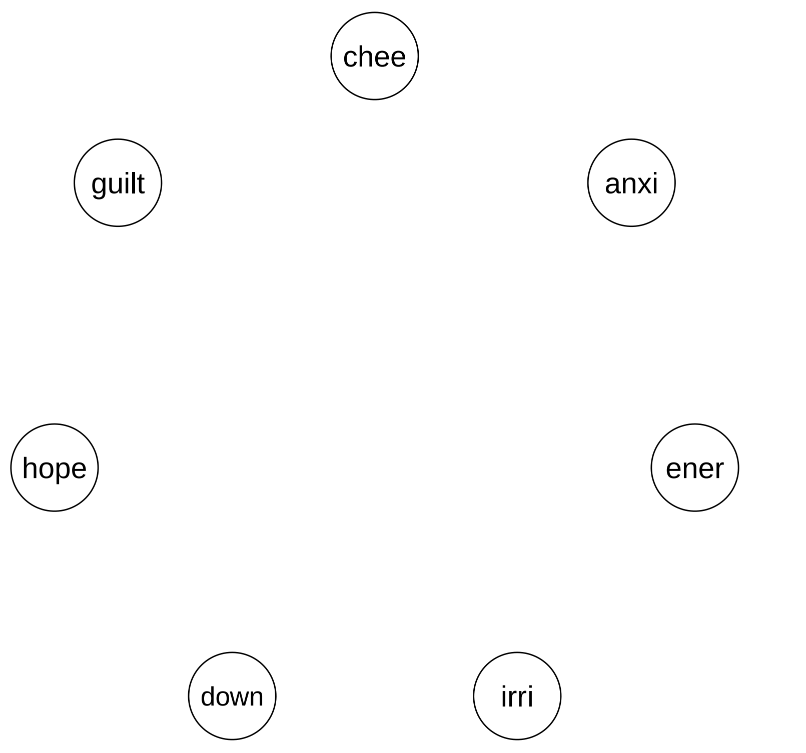

Healthy control reg 88SS Estpoint 3

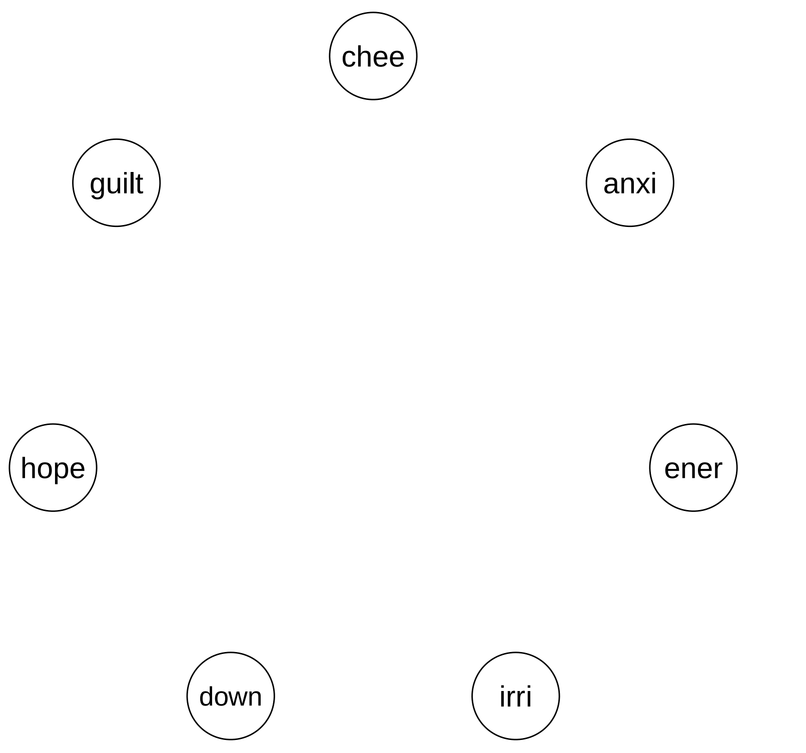

Healthy control reg 88SS Estpoint 4

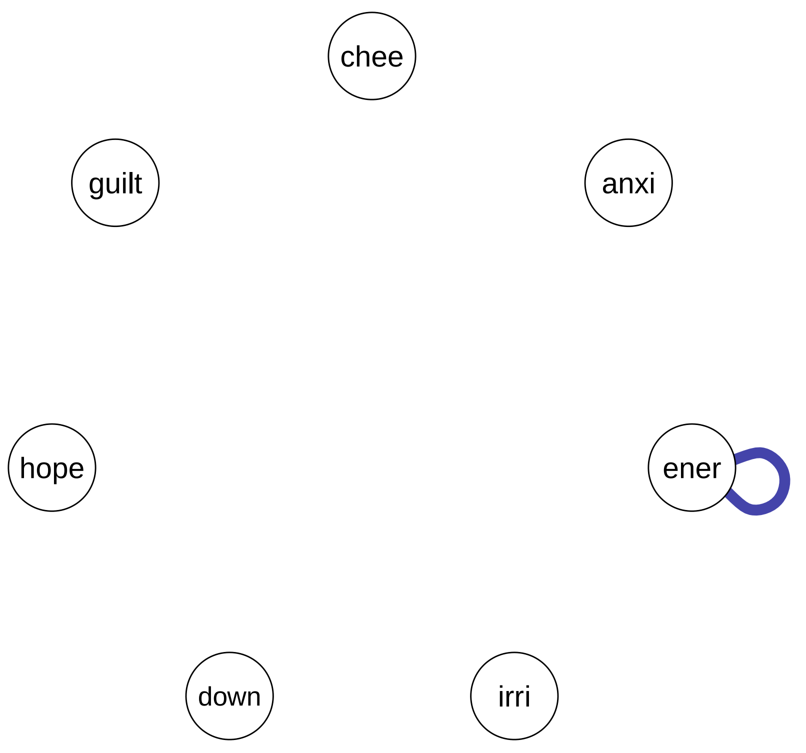

Healthy control reg 88SS Estpoint 5

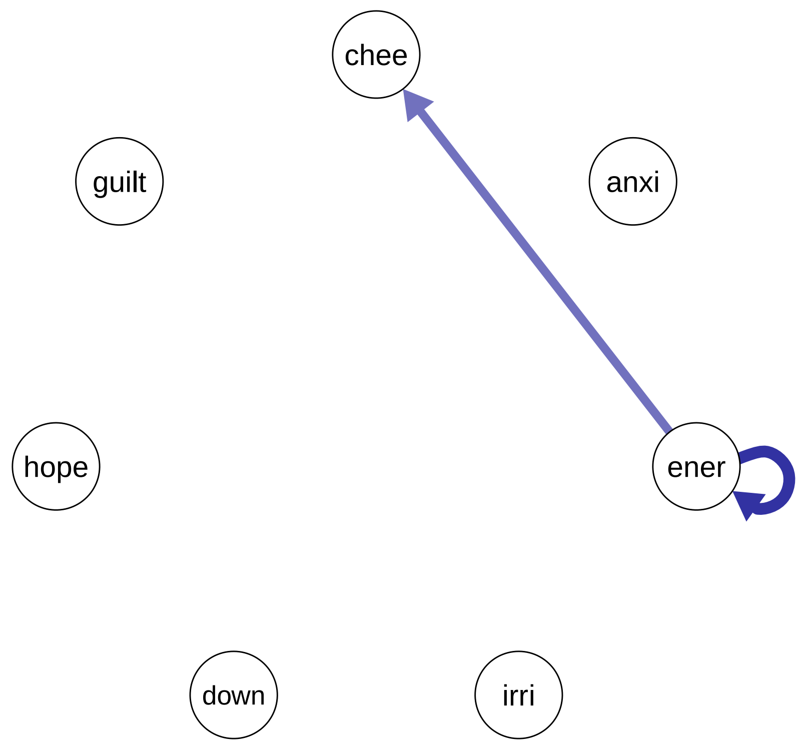

Healthy control reg 88SS Estpoint 6

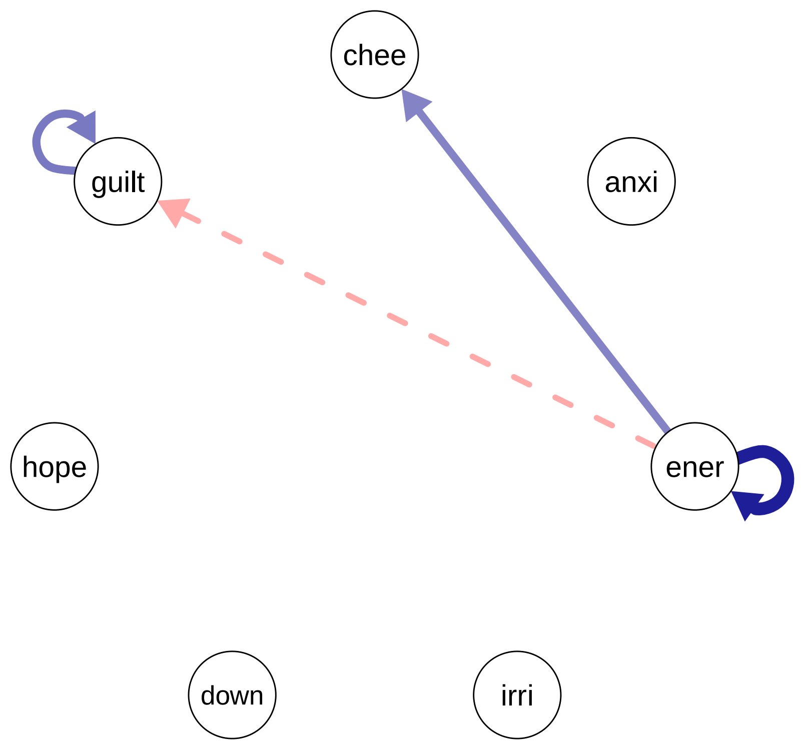

Healthy control reg 88SS Estpoint 7

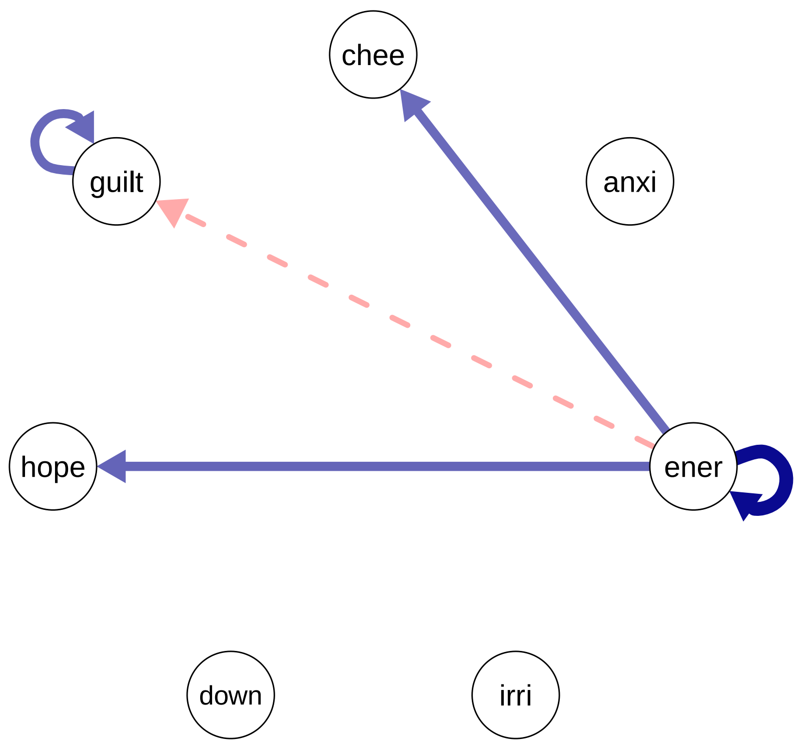

Healthy control reg 88SS Estpoint 8

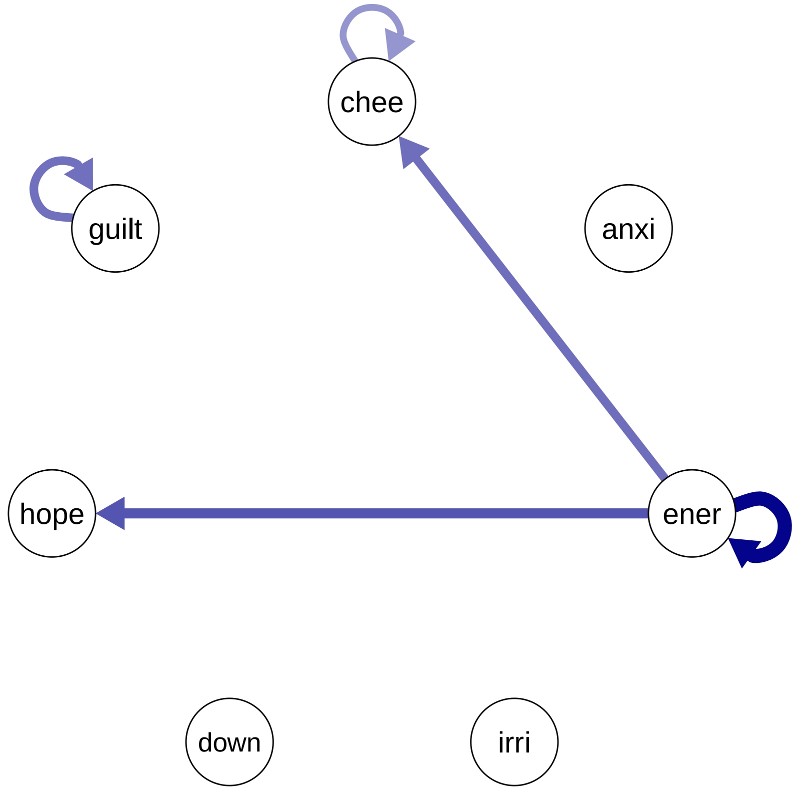

Healthy control reg 88JV Estpoint 1

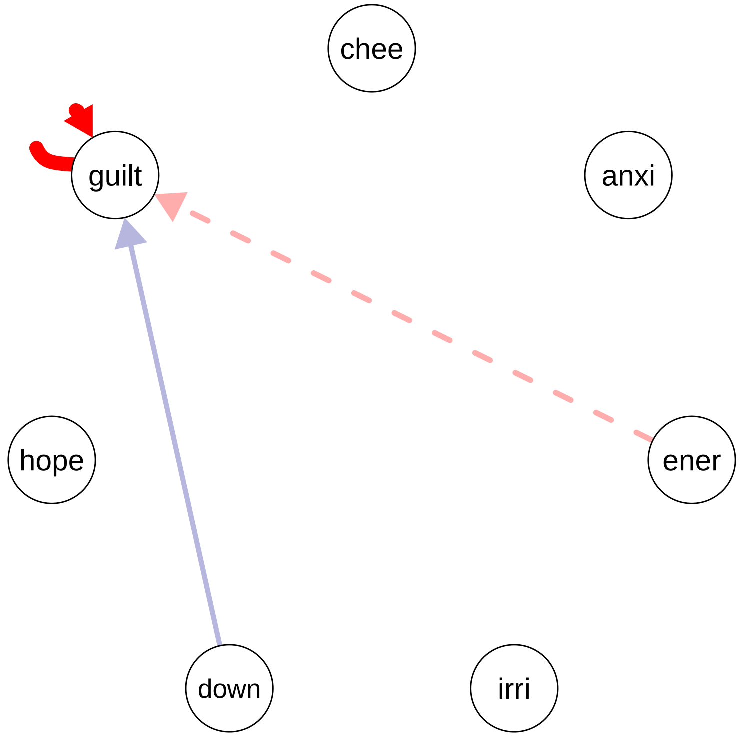

Healthy control reg 88JV Estpoint 2

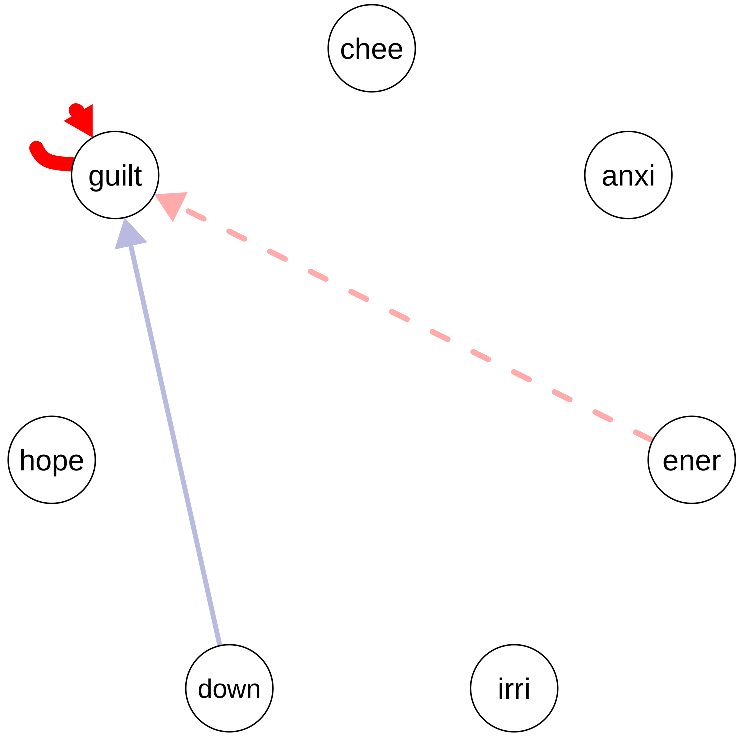

Healthy control reg 88JV Estpoint 3

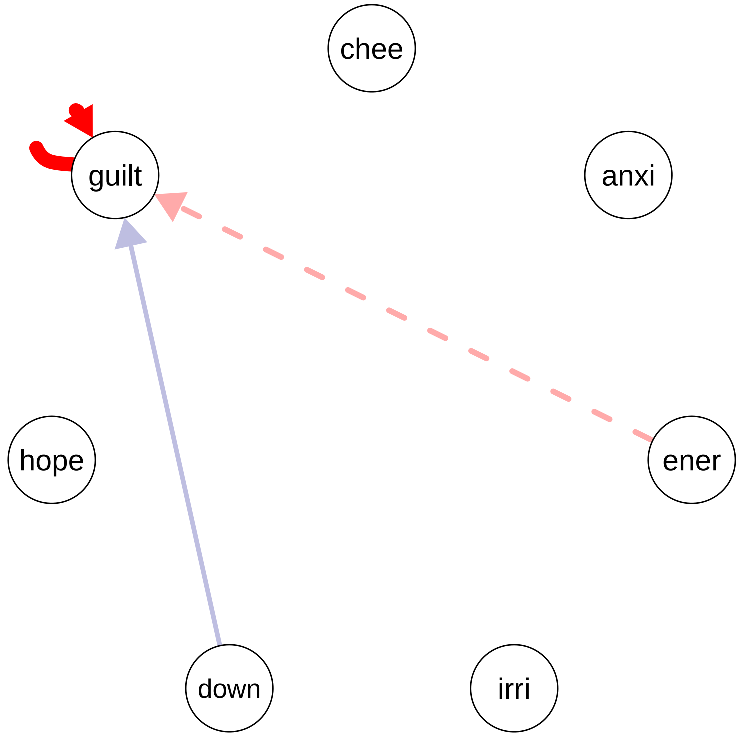

Healthy control reg 88JV Estpoint 4

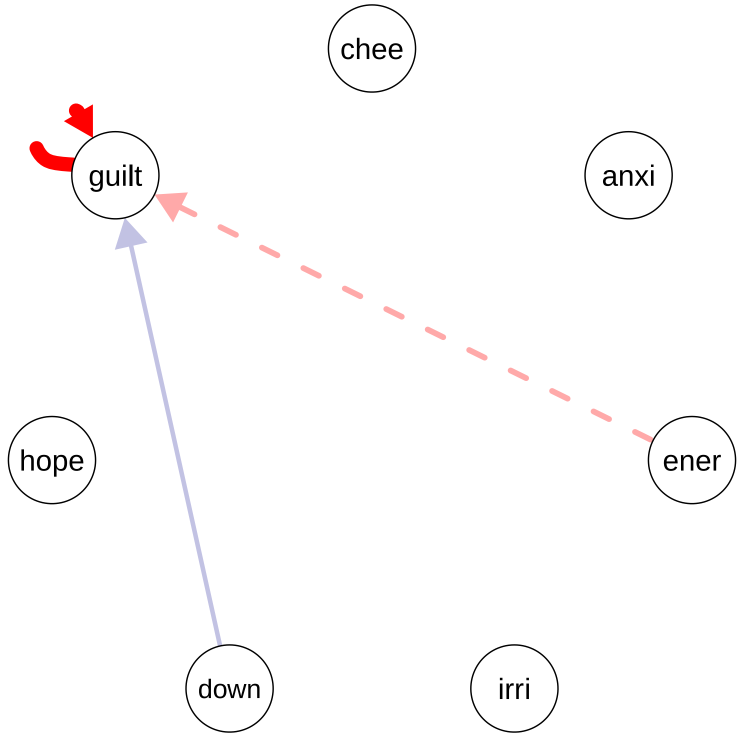

Healthy control reg 88JV Estpoint 5

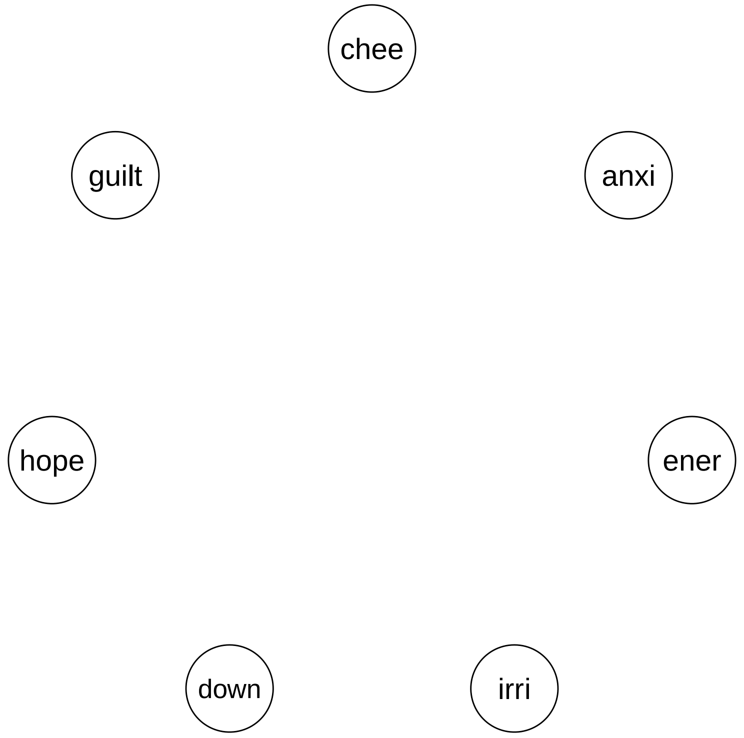

Healthy control reg 88JV Estpoint 6

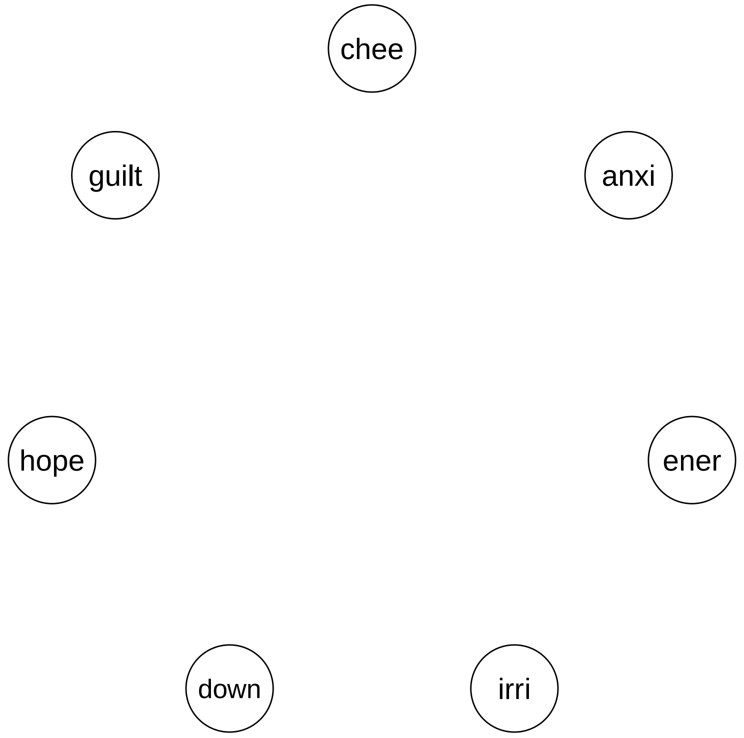

Healthy control reg 88JV Estpoint 7

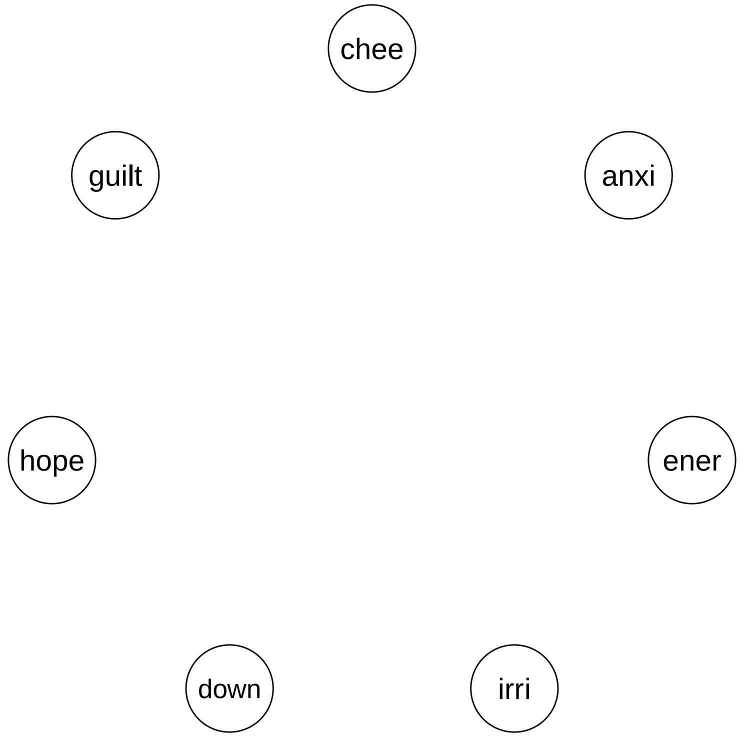

Healthy control reg 88JV Estpoint 8

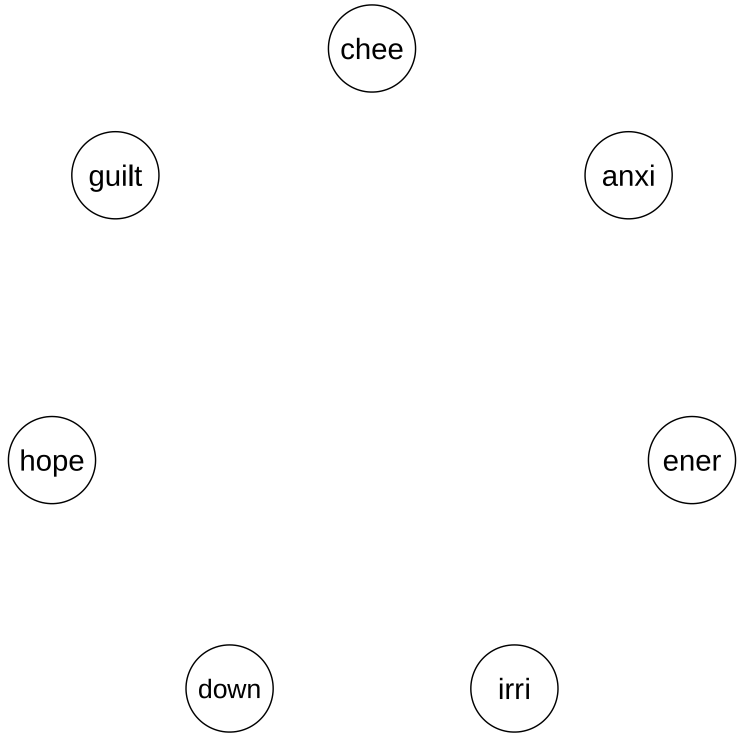

Healthy control reg 88MB Estpoint 1

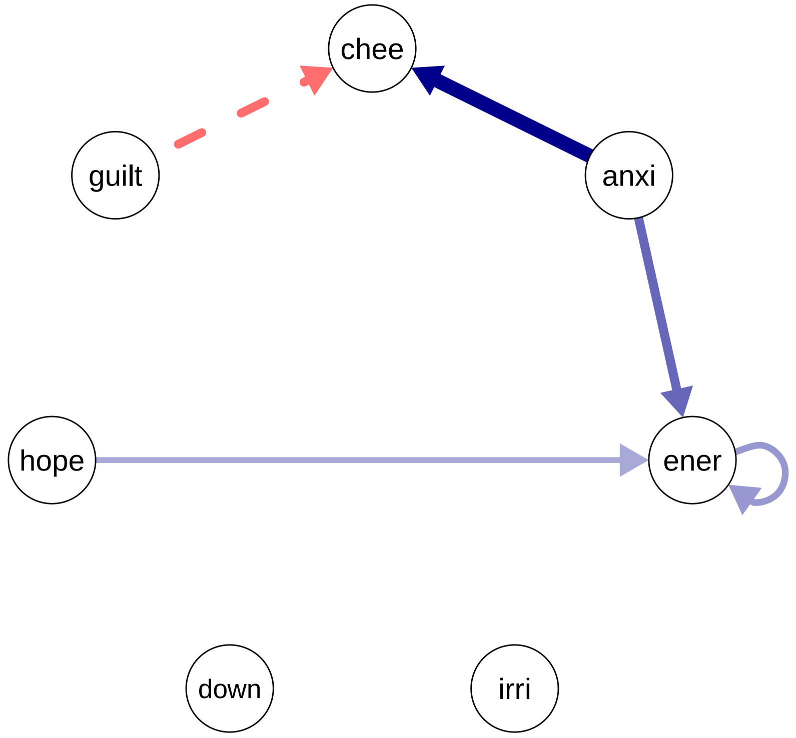

Healthy control reg 88MB Estpoint 2

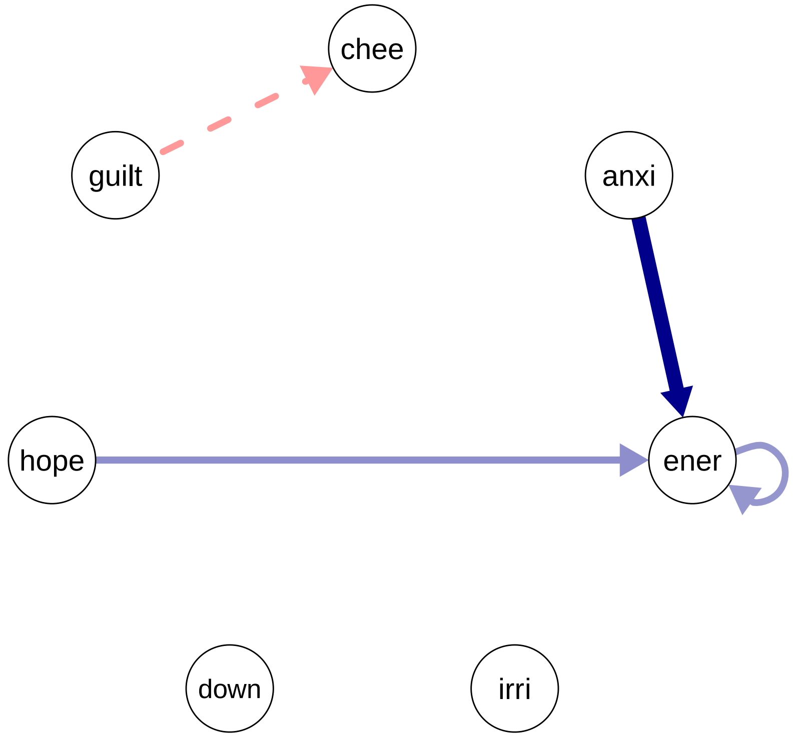

Healthy control reg 88MB Estpoint 3

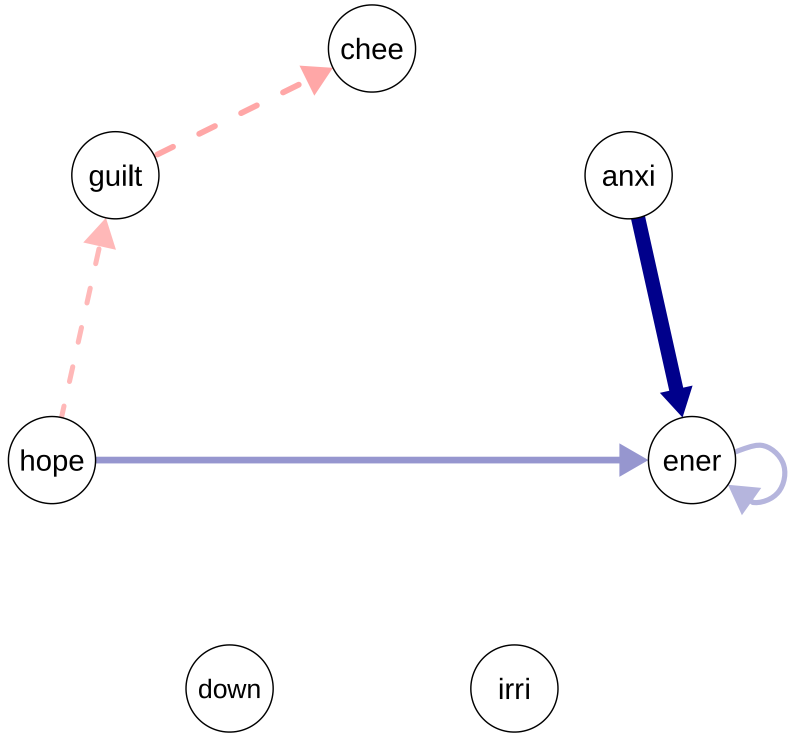

Healthy control reg 88MB Estpoint 4

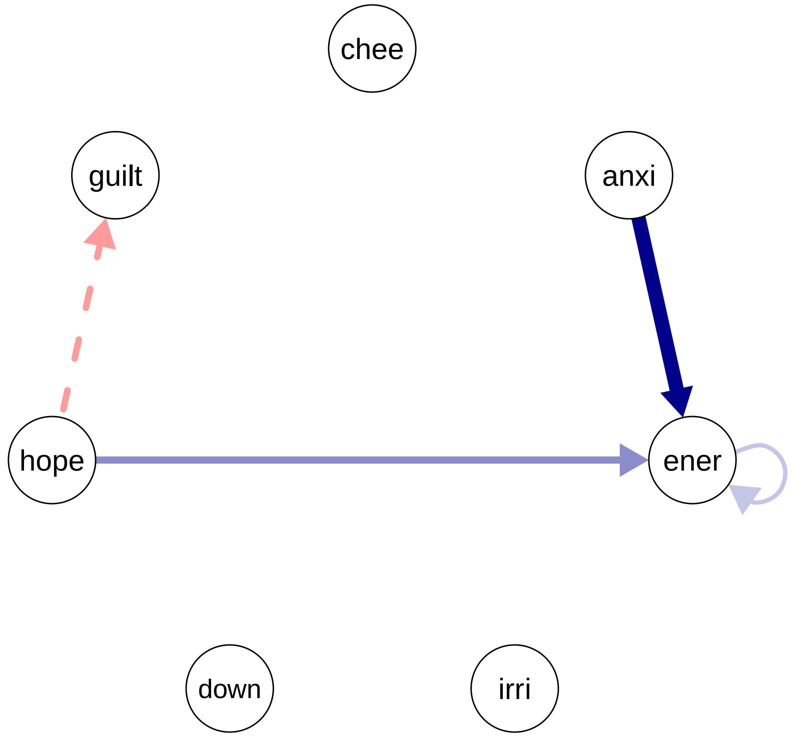

Healthy control reg 88MB Estpoint 5

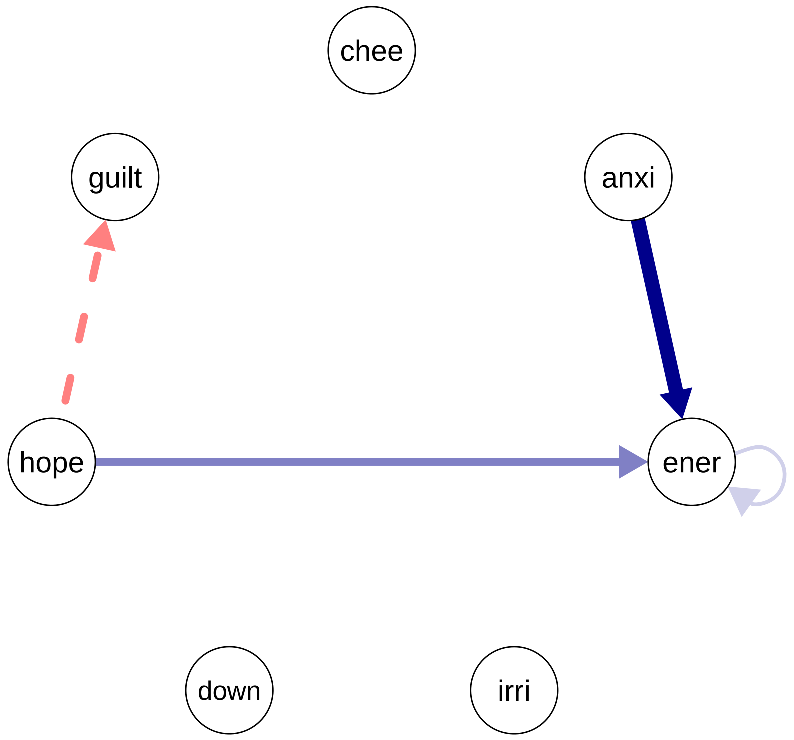

Healthy control reg 88MB Estpoint 6

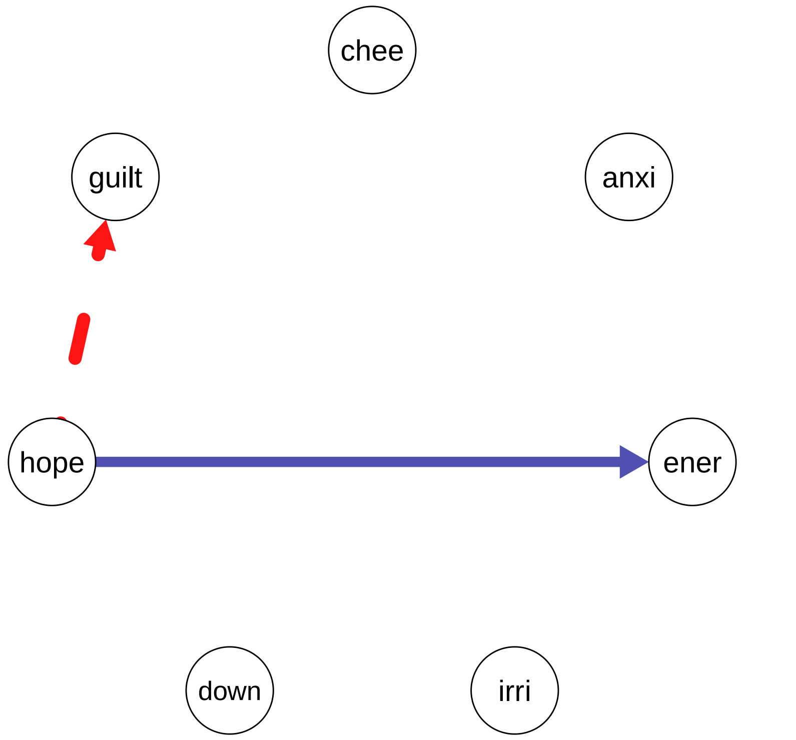

Healthy control reg 88MB Estpoint 7

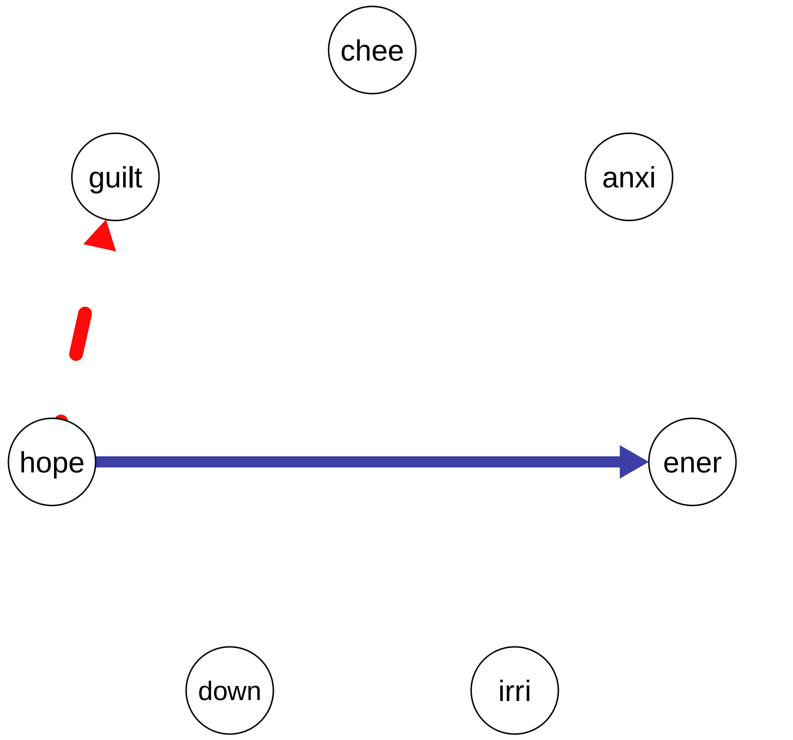

Healthy control reg 88MB Estpoint 8

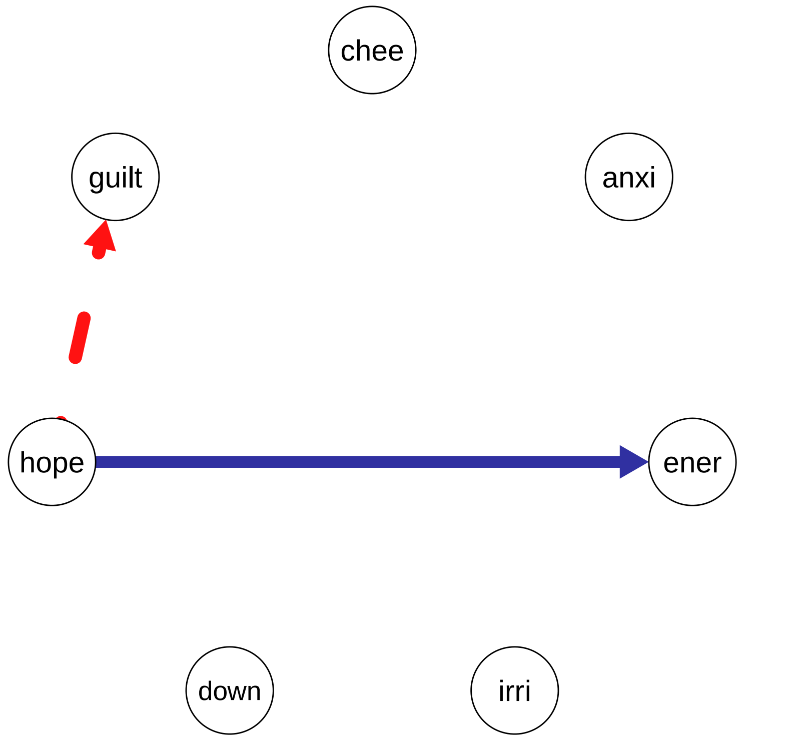

Supplement: Supplementary Figures — “Temporal affect networks of all n = 42 study participants and n = 11 healthy never-depressed controls”. [file mmc1.pdf]
