# Supplementary material for: Differential impact of preventive cognitive therapy while tapering antidepressants versus maintenance antidepressant treatment on affect fluctuations and individual affect networks and impact on relapse: a secondary analysis of a randomised controlled trial
Source: eClinicalMedicine. 2023 Nov 22;66:102329. doi: 10.1016/j.eclinm.2023.102329 (PMC10700372; doi:10.1016/j.eclinm.2023.102329)
Supplement: Supplementary Material [file mmc2.docx]

**Methodological appendix and supplementary tables 1-7 for ‘The differential impact of preventive cognitive therapy while tapering antidepressants versus maintenance antidepressant treatment on affect fluctuations and individual networks and impact on relapse: a secondary analysis of a randomised controlled trial’**

**Table of content supplementary material**

Supplementary material I: methodological appendix p. 2

Supplementary material II: supplementary tables p. 4

Supplementary table 1 p. 4

Supplementary table 2 p. 5

Supplementary table 3 p. 6

Supplementary table 4 p. 6

Supplementary table 5 p. 7

Supplementary table 6 p. 8

Supplementary table 7 p. 10

**Supplementary material I: methodological appendix**

**Time-varying vector autoregressive (TV-VAR) models**

Time-varying VAR (TV-VAR) models were recently developed as an extension of conventional VAR models, to accommodate the time-varying nature of some longitudinal datasets. There are various methods of estimating time-varying model parameters. For example, the Generalized Additive Modeling (GAM) framework estimates time-varying parameters by modeling them as a spline function of time, while penalized kernel-smoothing entails combining estimates of several local models that span the entire time period of the dataset.^1^ While subtle variations of parameters across time likely require time series much longer than the ones available in the present study, a recent simulation study showed that if parameters are highly time-varying, 55 observations can already be enough for a TV-VAR to outperform a standard VAR model.^1^ In addition, the same study showed the KS method outperformed the GAM framework for time series with a moderate sample size (n < 200-300), similar to the number of data points in our study. Therefore, we opted to use the kernel-smoothing method to estimate the TV-VAR models, which is implemented in the R-package *mgm.*^2^ The kernel-smoothing method estimates time-varying model parameters by estimating a number of local models by essentially sliding a window through the time series. The difference to a moving window approach is that it gives the observations within the window the same positive weight, while all other observations get a zero weight. Here, we use a Gaussian kernel function centered on the given estimation point, which gives us a weights for time points that depend on their distance from the given estimation point. Note that the number of estimation points is chosen to properly capture the measured time period but at the same time keep the computation time manageable. Looking at the average number of data points in the participants in our study in relation to the number of data points used in the aforementioned simulation study, we chose eight estimation points for our TV-VAR models thus resulting in eight temporal affect networks per participant. The number of estimated temporal affect networks across the time interval is in principle arbitrary, but in this context adding more would not have created additional meaningful differences between subsequent networks. Equally spaced local models are estimated by weighting all data point in the time series depending on their proximity to the estimation point of the specific local model. In order to account for the real-time time differences between measurements, the proximity from an estimation point is based on a timestamp variable that reflects how much time (in seconds) there is between two subsequent measures.

This package also includes a function that ensures that changes in networks over time do indeed reflect a time-varying process. A model selection procedure is used to select the optimal *bandwidth parameter*, which controls how time-varying parameters can be, using a data driven cross-validation scheme. If the parameters of the true models are not time-varying *or* if there is not enough data to reliably estimate true time-varying parameters, a simpler model with parameters that are not or hardly time-varying is selected. In an effort to have sufficient observations for every participant, and given that missing data imputation is currently only available for non-time-varying (i.e., stationary) psychological time-series^3^, participants with more than 70% missings were excluded from analyses. The number of observations for the n = 42 participants included in the TV-VAR analyses ranged from 34 – 179, with n = 3 participants still providing less than 55 observations (see Suppl. Table 6 for a full overview). We therefore also chose to limit the number of affect items in our networks to seven, which is less than the ten variables in the TV-VAR models in the aforementioned simulation study and thus requires less statistical power to estimate. Nonetheless, this is still enough to select affect items that reflect positive and negative affect dimensions, high and low arousal, and key symptoms of MDD.^4^ The affect items included in our networks are: *feeling* *down*, *anxious*, *irritated*, *guilty*, *cheerful*, *energetic*, and *hopeful*.

**Calculating average change in network structure over time**

We calculated the average change in affect networks over time for each participant in the study. That is, for each possible interaction in the eight individual networks, we computed the mean interaction effect and calculated the standard deviation (SD) from this mean. As such, a higher SD was reflective of higher deviations from this person-specific mean throughout the study, which was interpreted as a measure of the average rate of change in the particular interaction effect over time. We then calculated, per individual participant, the mean of the SDs for all interaction effects in the individual networks (including null-values in case of no interaction effect between certain affect items). These individual mean SDs were used as a quantification of the overall rate of change in the individual network architecture as a whole over time. Finally, we calculated the group-level means by averaging the individual mean SDs within treatment groups (both intention-to-treat and as-treated), as well as within groups according to relapse status at follow-up (i.e., early follow-up [3 months] or complete follow-up [up to 24 months]).

**References**

1 Haslbeck JMB, Bringmann LF, Waldorp LJ. A Tutorial on Estimating Time-Varying Vector Autoregressive Models. *Multivariate Behav Res* 2020; **0**: 1–30.

2 Haslbeck JMB, Waldorp LJ. MGM: Estimating time-varying mixed graphical models in high-dimensional data. *J Stat Softw* 2020; **93**. DOI:10.18637/jss.v093.i08.

3 Mansueto AC, Wiers RW, van Weert JCM, Schouten BC, Epskamp S. Investigating the Feasibility of Idiographic Network Models. *Psychol Methods* 2022. DOI:10.1037/met0000466.

4 Tellegen A, Watson D. Toward a Consensual Structure of Mood. *Psychol Bull* 1985; **98**: 219–35.

| **Supplementary table 1: logistic regression models of affect fluctuations and relapse** | | | | | | | | | |
| --- | --- | --- | --- | --- | --- | --- | --- | --- | --- |
|  | **Within-person variance** | | | **Autocorrelation** | | | **RMSSD** | | |
|  | Beta SE | OR (95% CI) | *P* | Beta SE | OR (95% CI) | *p* | Beta SE | OR (95% CI) | *p* |
| NA | -0.21 (0.32) | 0.81 (0.42 – 1.53) | 0.51 | -0.29 (0.33) | 0.75 (0.80 – 2.62) | 0.37 | -0.10 (0.32) | 0.90 (0.80 – 2.78) | 0.74 |
| PA | -0.27 (0.32) | 0.76 (0.40 – 1.42) | 0.39 | -0.10 (0.32) | 0.90 (0.46 – 1.69) | 0.75 | -0.22 (0.32) | 0.80 (0.42 – 1.51) | 0.49 |
| Angry | -0.10 (0.32) | 0.91 (0.48 – 1.72) | 0.76 | -0.49 (0.33) | 0.61 (0.30 – 1.15) | 0.14 | 0.05 (0.32) | 1.06 (0.56 – 2.04) | 0.87 |
| Anx | - 0.34 (0.32) | 0.71 (0.36 - 1.33) | 0.29 | -0.03 (0.32) | 0.71 (0.36 - 1.33) | 0.93 | - 0.21 (0.32) | 1.03 (0.42 - 1.52) | 0.51 |
| Chee | - 0.22 (0.32) | 0.80 (0.40 - 1.51) | 0.49 | 0.23 (0.32) | 1.26 (0.67 - 2.43) | 0.48 | - 0.28 (0.32) | 0.76 (0.39 - 1.42) | 0.39 |
| Cont | -0.40 (0.33) | 0.67 (0.33 – 1.25) | 0.22 | 0.17 (0.32) | 1.19 (0.63 – 2.27) | 0.59 | -0.53 (0.34) | 0.59 (0.29 – 1.11) | 0.12 |
| Down | - 0.23 (0.32) | 0.79 (0.41 - 1.49) | 0.22 | - 0.27 (0.32) | 0.77 (0.40 - 1.43) | 0.41 | - 0.11 (0.32) | 0.90 (0.47 - 1.68) | 0.73 |
| Ener | - 0.35 (0.32) | 0.71 (0.36 - 1.32) | 0.28 | 0.12 (0.32) | 1.13 (0.60 - 2.14) | 0.71 | - 0.40 (0.33) | 0.67 (0.33 - 1.26) | 0.22 |
| Enth | - 0.19 (0.32) | 1.14 (0.61 – 2.16) | 0.68 | 0.13 (0.32) | 1.14 (0.61 – 2.19) | 0.68 | - 0.17 (0.32) | 0.84 (0.44 – 1.57) | 0.59 |
| Guilt | - 0.68 (0.41) | 0.51 (0.20 - 1.02) | 0.10 | - 0.36 (0.33) | 0.70 (0.36 - 1.31) | 0.27 | - 0.35 (0.33) | 0.71 (0.36 - 1.33) | 0.29 |
| Help | 0.05 (0.32) | 1.05 (0.56 – 2.01) | 0.88 | - 0.09 (0.32) | 1.09 (0.58 – 2.07) | 0.78 | - 0.08 (0.32) | 0.93 (0.49 – 1.74) | 0.81 |
| Hope | - 0.24 (0.32) | 0.79 (0.41 - 1.47) | 0.46 | - 0.12 (0.32) | 0.89 (0.47 - 1.67) | 0.72 | - 0.24 (0.32) | 0.79 (0.41 - 1.47) | 0.46 |
| Insec | -0.26 (0.32) | 0.77 (0.40 – 1.44) | 0.41 | - 0.41 (0.33) | 0.67 (0.33 – 1.26) | 0.22 | - 0.23 (0.32) | 0.80 (0.41 – 1.49) | 0.48 |
| Irrir | - 0.13 (0.32) | 0.87 (0.46 - 1.65) | 0.67 | - 0.17 (0.32) | 0.84 (0.43 - 1.59) | 0.60 | - 0.04 (0.32) | 0.96 (0.50 - 1.81) | 0.89 |
| Lone | -0.23 (0.32) | 0.79 (0.42 – 1.48) | 0.46 | - 0.14 (0.32) | 0.87 (0.45 – 1.63) | 0.65 | - 0.26 (0.32) | 0.77 (0.40 – 1.44) | 0.42 |
| Susp | 0.002 (0.32) | 1.00 (0.54 – 1.94) | 0.99 | - 0.10 (0.32) | 0.91 (0.48 – 1.71) | 0.75 | 0.32 (0.3) | 0.95 (0.51 – 1.80) | 0.87 |

**Supplementary material II: Supplementary tables**

NA = (aggregated) negative affect, PA = (aggregated) positive affect, SE = standard error, OR: odds radio, 95% CI = 95% confidence interval, RMSSD = root mean square of successive differences

| **Supplementary table 2: Cox proportional hazard models of individual affect fluctuations and time to relapse** | | | | | | | | | |
| --- | --- | --- | --- | --- | --- | --- | --- | --- | --- |
|  | **Within-person variance** | | | **Autocorrelation** | | | **RMSSD** | | |
|  | HR | CI 95% | *p* | HR | CI 95% | *p* | HR | CI 95% | *p* |
| NA | 0.93 | 0.62 - 1.41 | 0.74 | 0.91 | 0.63 - 1.32 | 0.62 | 0.99 | 0.67 - 1.46 | 0.95 |
| PA | 0.85 | 0.56 - 1.28 | 0.43 | 1.06 | 0.73 - 1.53 | 0.77 | 0.85 | 0.57 - 1.26 | 0.41 |
| Angry | 0.95 | 0.64 - 1.42 | 0.82 | 0.78 | 0.52 - 1.17 | 0.23 | 1.04 | 0.69 – 1.54 | 0.87 |
| Anx | 0.86 | 0.57 – 1.30 | 0.46 | 1.10 | 0.75 – 1.62 | 0.63 | 0.90 | 0.62 – 1.31 | 0.59 |
| Chee | 0.85 | 0.55 – 1.33 | 0.48 | 1.26 | 0.84 – 1.89 | 0.26 | 0.80 | 0.53 – 1.19 | 0.27 |
| Cont | 0.77 | 0.50 - 1.21 | 0.26 | 1.19 | 0.79 - 1.78 | 0.40 | 0.71 | 0.46 – 1.09 | 0.12 |
| Down | 0.97 | 0.65 - 1.44 | 0.89 | 0.91 | 0.61 - 1.36 | 0.63 | 1.04 | 0.69 - 1.57 | 0.85 |
| Ener | 0.73 | 0.48 - 1.13 | 0.16 | 1.13 | 0.75 - 1.69 | 0.56 | 0.71 | 0.47 - 1.08 | 0.11 |
| Enth | 0.85 | 0.57 - 1.28 | 0.44 | 1.19 | 0.82 - 1.74 | 0.37 | 0.83 | 0.55 - 1.23 | 0.35 |
| Guilt | 0.66 | 0.38 - 1.16 | 0.15 | 0.90 | 0.62 - 1.31 | 0.58 | 0.82 | 0.55 - 1.22 | 0.34 |
| Help | 1.14 | 0.78 - 1.67 | 0.51 | 1.15 | 0.78 - 1.69 | 0.48 | 1.04 | 0.68 - 1.59 | 0.86 |
| Hope | 0.83 | 0.55 - 1.25 | 0.38 | 1.00 | 0.68 - 1.47 | 0.99 | 0.86 | 0.59 - 1.27 | 0.46 |
| Insec | 0.94 | 0.63 - 1.42 | 0.78 | 0.85 | 1.17 - 1.25 | 0.41 | 0.96 | 0.64 - 1.45 | 0.84 |
| Irrir | 0.92 | 0.63 - 1.35 | 0.67 | 0.97 | 0.67 - 1.40 | 0.87 | 0.99 | 0.68 - 1.44 | 0.96 |
| Lone | 0.90 | 0.57 - 1.43 | 0.66 | 0.96 | 0.63 - 1.45 | 0.85 | 0.84 | 0.53 - 1.32 | 0.44 |
| Susp | 1.07 | 0.71 - 1.59 | 0.76 | 0.92 | 0.60 - 1.42 | 0.71 | 1.03 | 0.70 - 1.51 | 0.90 |

NA = (aggregated) negative affect, PA = (aggregated) positive affect, HR = hazard ratio, CI = confidence interval,

RMSSD = root mean square of successive difference

| **Supplementary table 3: Group-level average change in temporal affect networks over the course of the study period measured as mean of individual network parameter SDs; mean SD’s, SD, (range SDs)** | | | | | | | | |
| --- | --- | --- | --- | --- | --- | --- | --- | --- |
|  | **PCT comb. ADM**  (ITT, n = 15) | **PCT tap. ADM**  (ITT, n = 17) | **PCT comb. ADM**  (AT, n = 23) | **PCT tap. ADM**  (AT, n = 9) | **ADM only**  (n = 10) | **Relapse < 3 months**  (n = 5) | **Relapse 2-year** **FU**  (n = 25) | **No relapse 2-year FU**  (n = 17) |
| Reg.  TV-VAR models | 0.0192, 0.0137  (0.0020 - 0.0508) | 0.0231, 0.0113 (0.0039 - 0.0511) | 0.0217, 0.0134  (0.0020 - 0.0511) | 0.0203, 0.0104  (0.0045 - 0.0365) | 0.0207, 0.133  (0.0019 - 0.0465) | 0.0249, 0.0058 (0.0177 - 0.0309) | 0.0213, 0.0131(0.0019 – 0.0508) | 0.0209, 0.0119 (0.0020 - 0.0511) |
| Non-reg.  TV-VAR models | 0.1739, 0.2119 (0.0037 - 0.7006) | 0.1489, 0.0783 (0.0477 - 0.2968) | 0.1750, 0.1770(0.0037 - 0.7006) | 0.1238, 0.0563 (0.0519 - 0.1972) | 0.1091, 0.0689 (0.0191 - 0.2228) | 0.2084, 0.2333  (0.0543 - 0.6158) | 0.1622, 0.1211  (0.0054 - 0.6158) | 0.1280, 0.1641 (0.0037 - 0.7006) |

*No significant differences in group-level mean SD’s according to treatment group or relapse status, ANOVA or Kruskal-Wallis test p-values shown in supplementary table 5.

See methods section and methodology appendix for detailed explanation of how average change in temporal affect network structure (i.e., mean SD) is calculated. SD = standard deviation, PCT = preventive cognitive therapy, ADM = antidepressant medication, comb. = combined with, tap. = while tapering, ITT = intention-to-treat, AT = as-treated, TV-VAR = time-varying vector autoregression.

| **Supplementary table 4: Group-level average change in temporal affect networks over the course of the study period for health never-depressed controls measured as mean of individual network parameter SDs** | | | | |
| --- | --- | --- | --- | --- |
| **Treatment arm** | **With regularization** | | **Without regularization** | |
|  | Average change in temporal affect networks during study period  (mean SD, SD) | Range SDs | average change in temporal affect networks during study period  (mean SD, SD) | Range SDs |
| Healthy never-depressed controls  (n = 11) | 0.0214, 0.0169 | 0.0024 - 0.0515 | 0.0964, 0.0684 | 0.0066 - 0.2223 |

*No significant difference in group-level mean between healthy never-depressed controls (n = 11) and study participants who experienced relapse at follow-up (n =25), ANOVA or Kruskal-Wallis test p-values shown in supplementary table 5. See methods section and methodology appendix for detailed explanation of how average change in temporal affect network structure (i.e., mean SD) is calculated. SD = standard deviation

| **Supplementary table 5: group-level comparisons mean SD’s values as proxy for average change over time in temporal affect network structure** | |
| --- | --- |
| **Comparison** | **Test-statistic (p-value)** |
| Non-regularized TV-VAR models |  |
| Three different treatment conditions (ITT) | Kruskal-Wallis chi-squared = 1.5177 (p = 0.4682) |
| Three different treatment conditions (AT) | Kruskal-Wallis chi-squared = 0.8166 (p = 0.6648) |
| Relapse complete FU (n = 25) vs. non-relapse (n = 17) | Kruskal-Wallis chi-squared = 3.4514 (p = 0.0632) |
| Relapse early FU (n = 5) vs. non-relapse (n = 17) | Kruskal-Wallis chi-squared = 1.1187 (p = 0.2902) |
| Relapse complete FU (n = 25) vs. never depressed healthy controls (n = 11) | Kruskal-Wallis chi-squared = 2.6609 (p = 0.1028) |
|  | |
| Regularized TV-VAR models |  |
| Three different treatment conditions (ITT) | ANOVA F-value = 0.384 (p = 0.684) |
| Three different treatment conditions (AT) | ANOVA F-value = 0.955 (p = 0.955) |
| Relapse complete FU (n = 25) vs. non-relapse (n = 17) | ANOVA F-value = 0.014 (p = 0.905) |
| Relapse early FU (n = 5) vs. non-relapse (n = 17) | ANOVA F-value = 0.527 (p = 0.476) |
| Relapse complete FU (n = 25) vs. never depressed healthy controls (n = 11) | ANOVA F-value = 0 (p = 0.991) |

See methods section and methodology appendix for detailed explanation of how average change in temporal affect network structure (i.e., mean SD) is calculated. SD = standard deviation; TV-VAR = time-varying vector autoregression; ITT = intention-to-treat; AT = as-treated; FU = follow-up

| **Supplementary table 6: number of data points in TV-VAR models, bandwidth parameter used to estimate model, mean SDs (continues on next page)** | | | | |
| --- | --- | --- | --- | --- |
| **Participant no.** | **Data points in**  **TV-VAR model** | **Bandwidth** | **Mean SD (regularized)** | **Mean SD**  **(non-regularized)** |
| PCT tapering ADM  (ITT, n = 17) |  |  |  |  |
| 68 | 141 | 0.45 | 0.04766584 | 0.0231187 |
| 158 | 64 | 0.12 | 0.2048817 | 0.01295731 |
| 236 | 88 | 0.12 | 0.2294699 | 0.01784737 |
| 245 | 133 | 0.34 | 0.05428222 | 0.01772272 |
| 246 | 105 | 0.12 | 0.1783782 | 0.02344898 |
| 253 | 129 | 0.12 | 0.1586257 | 0.03066403 |
| 254 | 151 | 0.23 | 0.09290317 | 0.02907159 |
| 260 | 96 | 0.12 | 0.197218 | 0.03648501 |
| 262 | 78 | 0.12 | 0.2967937 | 0.05105259 |
| 263 | 104 | 0.23 | 0.08471732 | 0.00708009 |
| 266 | 174 | 0.23 | 0.08681255 | 0.02592661 |
| 267 | 89 | 0.12 | 0.2801454 | 0.003926287 |
| 272 | 75 | 0.34 | 0.08790963 | 0.03413807 |
| 277 | 108 | 0.23 | 0.1284951 | 0.01599905 |
| 284 | 63 | 0.23 | 0.1613676 | 0.02432872 |
| 285 | 177 | 0.34 | 0.05073246 | 0.01918767 |
| 286 | 96 | 0.12 | 0.1908228 | 0.01980611 |
| PCT plus ADM  (ITT, n = 15) |  |  |  |  |
| 225 | 106 | 0.12 | 0.2200927 | 0.05080242 |
| 232 | 162 | 0.34 | 0.05188748 | 0.004497402 |
| 234 | 113 | 0.12 | 0.1375675 | 0.006418483 |
| 247 | 179 | 0.01 | 0.6158023 | 0.02700589 |
| 248 | 122 | 1 | 0.005384146 | 0.006605416 |
| 250 | 43 | 0.12 | 0.7005689 | 0.03027959 |
| 255 | 83 | 0.23 | 0.1039681 | 0.01485074 |
| 258 | 116 | 0.12 | 0.09953448 | 0.02970589 |
| 261 | 186 | 0.89 | 0.01072496 | 0.009452997 |
| 264 | 127 | 0.12 | 0.2788338 | 0.03218576 |
| 269 | 63 | 0.23 | 0.08114754 | 0.01122248 |
| 274 | 104 | 0.43 | 0.08816589 | 0.03092801 |
| 288 | 152 | 0.34 | 0.04597147 | 0.01826799 |
| 290 | 102 | 0.89 | 0.003663098 | 0.002023148 |
| 291 | 99 | 0.12 | 0.1657959 | 0.01386109 |
| ADM only  (n = 10) |  |  |  |  |
| 224 | 154 | 0.12 | 0.1318831 | 0.02350591 |
| 233 | 53 | 0.56 | 0.041325 | 0.007021143 |
| 239 | 67 | 0.45 | 0.04246477 | 0.01060503 |
| **Suppl. table 6 continued** | | | | |
| **Participant no.** | **Data points in TV-VAR model** | **Bandwidth** | **Mean SD (regularized)** | **Mean SD (non-reg.)** |
| 243 | 137 | 0.12 | 0.1593681 | 0.01796437 |
| 249 | 95 | 0.12 | 0.1980721 | 0.02480709 |
| 257 | 47 | 1 | 0.01909329 | 0.01865123 |
| 259 | 70 | 0.23 | 0.1128228 | 0.001898939 |
| 265 | 113 | 0.34 | 0.07107489 | 0.01961421 |
| 273 | 120 | 0.12 | 0.2228446 | 0.04651915 |
| 287 | 169 | 0.23 | 0.0921446 | 0.03644603 |

SD = standard deviation; TV-VAR = time-varying vector autoregressive; PCT = protective cognitive therapy; ADM = antidepressant medication;
ITT = intention-to-treat

| **Supplementary table 7: mean (SD) affect scores per week for the entire group and per treatment group (continues on next page)** | | | | | | | | |
| --- | --- | --- | --- | --- | --- | --- | --- | --- |
| **Entire group (n = 42)** | | | | | | | | |
|  | **Week 1** | **Week 2** | **Week 3** | **Week 4** | **Week 5** | **Week 6** | **Week 7** | **Week 8** |
| PA | 48.82 (18.74) | 46.34 (18.59) | 47.92 (19.08) | 48.44 (18.53) | 47.30 (18.42) | 47.74 (18.28) | 46.83 (17.95) | 48.93 (18.27) |
| NA | 17.53 (14.91) | 15.95 (12.98) | 13.30 (11.28) | 15.24 (140.4) | 12.88 (12.70) | 14.51 (13.85) | 14.38 (14.72) | 13.32 (12.90) |
| Cheerful | 51.36 (22.49) | 49.77 (22.01) | 51.32 (22.10) | 52.78 (20.30) | 52.22 (20.53) | 53.88 (19.20) | 51.24 (20.29) | 53.43 (19.64) |
| Anxious | 11.97 (14.27) | 10.32 (12.77) | 9.61 (12.83) | 10.53 (12.79) | 9.34 (12.68) | 10.71 (13.50) | 11.68 (14.98) | 10.30 (13.22) |
| Enthusiastic | 46.77 (23.38) | 43.15 (23.29) | 44.44 (23.40) | 44.58 (22.58) | 44.87 (22.62) | 44.27 (22.61) | 43.71 (21.92) | 44.60 (23.57) |
| Angry | 14.20 (18.48) | 12.98 (17.12) | 11.23 (15.59) | 12.39 (17.05) | 10.68 (15.31) | 11.09 (15.15) | 10.68 (14.83) | 10.63 (14.60) |
| Lonely | 19.64 (21.29) | 17.08 (18.57) | 13.53 (15.66) | 15.24 (17.47) | 14.13 (15.86) | 15.28 (17.79) | 15.44 (18.87) | 13.32 (16.43) |
| Energetic | 45.77 (23.24) | 44.05 (22.56) | 45.72 (21.92) | 44.73 (21.42) | 43.06 (21.09) | 44.19 (21.43) | 45.06 (19.76) | 46.25 (21.02) |
| Irritated | 20.98 (23.81) | 19.84 (21.92) | 17.30 (20.57) | 17.74 (20.90) | 15.28 (18.89) | 17.43 (20.52) | 16.28 (20.32) | 15.97 (19.21) |
| Down | 20.99 (22.67) | 19.05 (20.36) | 15.24 (17.97) | 17.72 (20.10) | 14.85 (17.32) | 16.96 (19.29) | 17.76 (20.15) | 16.15 (19.07) |
| Suspicious | 13.28 (16.21) | 12.17 (14.39) | 9.93 (12.19) | 13.17 (16.44) | 11.01 (14.04) | 11.94 (15.16) | 10.97 (14.61) | 10.15 (13.54) |
| Hopeful | 47.57 (22.98) | 45.16 (22.75) | 46.33 (22.71) | 48.14 (22.59) | 45.05 (23.11) | 44.90 (22.31) | 44.32 (21.71) | 47.21 (22.45) |
| Helpless | 16.22 (18.80) | 13.68 (14.57) | 11.25 (14.09) | 13.70 (15.75) | 12.00 (14.89) | 13.16 (15.90) | 13.81 (17.14) | 11.68 (14.69) |
| Guilty | 20.06 (22.96) | 17.99 (19.37) | 15.14 (19.25) | 16.25 (19.06) | 13.09 (16.28) | 15.08 (18.09) | 12.73 (16.49) | 12.94 (16.22) |
| Content | 52.65 (23.31) | 49.55 (21.31) | 51.81 (21.75) | 51.99 (21.76) | 51.29 (20.80) | 51.49 (20.50) | 49.81 (20.44) | 53.16 (20.73) |
| Insecure | 20.43 (21.44) | 20.43 (20.24) | 16.49 (18.14) | 20.38 (20.01) | 15.51 (16.99) | 18.94 (20.11) | 20.09 (22.59) | 18.73 (21.42) |
| **Group receiving PCT while tapering ADM (n = 17)** | | | | | | | | |
|  | **Week 1** | **Week 2** | **Week 3** | **Week 4** | **Week 5** | **Week 6** | **Week 7** | **Week 8** |
| PA | 49.94 (20.12) | 48.14 (19.41) | 47.33 (21.50) | 48.77 (20.56) | 51.03 (22.30) | 46.09 (18.77) | 45.42 (18.23) | 48.31 (19.46) |
| NA | 22.00 (17.07) | 19.12 (15.47) | 17.72 (13.11) | 21.17 (17.75) | 18.70 (15.39) | 20.72 (15.89) | 23.66 (17.51) | 19.14 (15.23) |
| Cheerful | 50.50 (25.15) | 50.82 (23.42) | 50.80 (24.02) | 52.61 (22.61) | 54.90 (24.80) | 53.02 (19.66) | 50.33 (20.43) | 52.01 (21.22) |
| Anxious | 15.92 (16.64) | 13.94 (14.73) | 11.97 (14.13) | 16.34 (17.32) | 14.91 (17.37) | 13.74 (14.73) | 19.30 (18.99) | 14.04 (15.49) |
| Enthusiastic | 46.38 (24.94) | 45.32 (23.33) | 43.05 (24.45) | 43.74 (24.11) | 49.18 (25.81) | 41.68 (22.17) | 41.13 (21.57) | 42.91 (24.77) |
| Angry | 18.31 (20.68) | 15.75 (19.41) | 16.72 (19.59) | 17.10 (19.67) | 15.00 (18.22) | 15.68 (19.06) | 17.18 (19.35) | 14.25 (17.24) |
| Lonely | 24.69 (23.71) | 18.87 (18.65) | 17.08 (18.10) | 21.89 (21.81) | 20.10 (17.85) | 23.51 (2096) | 26.22 (22.80) | 19.69 (19.33) |
| Energetic | 49.24 (24.43) | 46.08 (22.55) | 45.13 (24.48) | 44.04 (23.84) | 46.55 (25.96) | 42.99 (22.63) | 44.73 (18.94) | 45.96 (23.06) |
| Irritated | 24.76 (25.01) | 21.13 (22.44) | 22.02 (22.29) | 21.47 (21.61) | 19.64 (19.56) | 23.78 (21.74) | 26.04 (23.78) | 21.22 (20.71) |
| Down | 25.88 (25.43) | 21.24 (22.37) | 19.61 (22.03) | 23.30 (23.99) | 20.09 (19.25) | 23.15 (21.70) | 26.60 (22.15) | 21.27 (20.23) |
| Suspicious | 17.28 (18.52) | 14.76 (16.25) | 12.85 (14.79) | 18.89 (21.11) | 17.06 (17.39) | 18.06 (18.50) | 18.86 (19.35) | 14.29 (16.47) |
| Hopeful | 50.89 (23.80) | 47.56 (23.10) | 48.01 (25.14) | 50.82 (24.08) | 50.44 (26.26) | 43.75 (22.31) | 44.54 (22.21) | 48.50 (23.38) |
| Helpless | 18.56 (19.77) | 16.06 (16.29) | 14.74 (16.14) | 18.63 (19.65) | 17.97 (17.65) | 19.08 (18.69) | 22.70 (20.80) | 18.17 (18.86) |
| Guilty | 27.54 (26.96) | 24.78 (22.52) | 22.10 (22.86) | 25.52 (24.30) | 19.86 (19.62) | 23.15 (21.20) | 21.83 (21.06) | 20.31 (19.90) |
| Content | 52.69 (25.47) | 50.92 (22.62) | 49.65 (24.48) | 52.65 (23.85) | 54.06 (24.38) | 48.99 (22.22) | 46.39 (21.69) | 52.15 (22.70) |
| Insecure | 25.03 (23.01) | 25.52 (22.15) | 22.35 (20.84) | 27.41 (22.20) | 23.71 (20.09) | 26.34 (20.53) | 34.26 (25.14) | 29.03 (24.05) |
| **Group receiving PCT while continuing ADM (n = 15)** | | | | | | | | |
|  | **Week 1** | **Week 2** | **Week 3** | **Week 4** | **Week 5** | **Week 6** | **Week 7** | **Week 8** |
| PA | 50.57 (17.87) | 43.54 (17.54) | 49.65 (15.59) | 48.11 (16.43) | 45.79 (16.28) | 49.956 (16.14) | 50.17 (15.08) | 49.02 (15.33) |
| NA | 13.42 (12.26) | 12.50 (9.69) | 10.42 (9.08) | 12.17 (9.52) | 10.08 (9.45) | 10.79 (10.19) | 10.26 (9.73) | 11.10 (10.01) |
| Cheerful | 54.08 (20.79) | 49.80 (20.46) | 53.94 (18.87) | 53.60 (18.26) | 52.83 (17.21) | 56.67 (16.12) | 53.64 (18.91) | 55.22 (16.06) |
| Anxious | 9.46 (12.57) | 8.03 (9.66) | 8.06 (10.86) | 8.38 (7.73) | 6.74 (7.74) | 9.02 (11.54) | 9.11 (11.09) | 9.63 (12.68) |
| Enthusiastic | 51.01 (22.06) | 40.88 (22.01) | 48.59 (20.48) | 47.01 (19.97) | 44.98 (20.18) | 49.21 (20.04) | 49.68 (18.21) | 46.75 (21.36) |
| Angry | 11.64 (16.83) | 10.58 (14.90) | 8.33 (11.25) | 10.86 (15.07) | 9.42 (14.21) | 8.84 (11.95) | 7.74 (9.81) | 10.06 (13.65) |
| **Suppl. table 7 continued: mean (SD) affect scores per week for the entire group and per treatment group** | | | | | | | | |
|  | **Week 1** | **Week 2** | **Week 3** | **Week 4** | **Week 5** | **Week 6** | **Week 7** | **Week 8** |
| Lonely | 12.71 (17.07) | 12.67 (16.01) | 10.94 (13.16) | 11.51 (12.71) | 11.25 (13.35) | 10.74 (14.28) | 9.26 (10.59) | 9.98 (11.90) |
| Energetic | 44.51 (22.28) | 39.68 (21.28) | 47.07 (18.18) | 44.54 (18.42) | 41.35 (18.51) | 45.62 (18.56) | 48.21 (16.55) | 46.84 (16.47) |
| Irritated | 18.51 (22.87) | 18.99 (21.84) | 13.72 (17.90) | 17.24 (20.59) | 14.47 (19.47.) | 13.11 (16.92) | 11.30 (15.61) | 14.93 (19.07) |
| Down | 15.79 (18.69) | 16.48 (17.71) | 13.76 (16.24) | 15.87 (16.68) | 12.51 (15.23) | 14.74 (17.79) | 15.10 (18.28) | 15.16 (18.76) |
| Suspicious | 9.88 (14.09) | 9.16 (12.57) | 7.54 (9.36) | 9.66 (12.05) | 7.36 (9.88) | 7.76 (10.72) | 6.95 (7.85) | 8.41 (11.91) |
| Hopeful | 48.23 (21.49) | 40.46 (21.24) | 45.11 (18.96) | 44.87 (21.15) | 41.15 (20.14) | 45.74 (20.98) | 47.27 (18.43) | 45.29 (19.31) |
| Helpless | 15.33 (19.33) | 11.18 (11.96) | 9.82 (12.77) | 10.90 (11.17) | 9.11 (11.97) | 8.68 (10.83) | 10.13 (12.50) | 8.16 (9.36) |
| Guilty | 11.50 (15.90) | 10.78 (14.05) | 9.41 (12.42) | 9.90 (11.00) | 8.86 (11.94) | 8.58 (11.15) | 7.94 (9.76) | 8.78 (11.29) |
| Content | 55.02 (22.51) | 46.88 (20.23) | 53.56 (18.37) | 50.50 (19.91) | 48.62 (18.95) | 52.53 (18.14) | 52.03 (18.13) | 51.01 (18.65) |
| Insecure | 15.92 (19.37) | 14.63 (16.54) | 12.20 (14.11) | 15.26 (16.48) | 11 (13.20) | 15.63 (18.86) | 14.80 (17.96) | 14.78 (18.80) |
| **Group receiving ADM only without PCT (n = 10)** | | | | | | | | |
|  | **Week 1** | **Week 2** | **Week 3** | **Week 4** | **Week 5** | **Week 6** | **Week 7** | **Week 8** |
| PA | 43.58 (16.26) | 47.26 (18.06) | 46.19 (20.09) | 48.47 (18.48) | 43.80 (13.22) | 47.19 (20.27) | 42.70 (21.30) | 49.83 (21.09) |
| NA | 15.32 (11.41) | 15.11 (10.28) | 11.51 (9.53) | 11.21 (10.28) | 8.26 (8.83) | 9.40 (10.44) | 6.46 (7.64) | 7.46 (8.80) |
| Cheerful | 48.52 (18.79) | 47.53 (21.42) | 48.19 (23.38) | 51.80 (19.69) | 46.43 (17.04) | 51.01 (22.18) | 48.12 (22.15) | 52.53 (22.55) |
| Anxious | 8.09 (8.65) | 6.58 (10.88) | 8.68 (13.22) | 5.36 (6.24) | 4.80 (5.32) | 8.01 (13.11) | 3.61 (3.55) | 5.11 (6.20) |
| Enthusiastic | 40.41 (20.70) | 42.45 (24.95) | 40.20 (25.03) | 42.21 (23.68) | 37.21 (19.09) | 41.04 (25.77) | 36.46 (25.91) | 43.49 (25.19) |
| Angry | 10.08 (14.24) | 11.21 (14.47) | 8.00 (12.65) | 7.86 (13.90) | 5.65 (8.41) | 6.51 (8.12) | 5.26 (8.85) | 5.46 (8.47) |
| Lonely | 21.02 (19.29) | 20.75 (20.94) | 12.51 (14.59) | 11.17 (13.16) | 9.43 (13.45) | 7.90 (8.54) | 8.95 (15.39) | 8.63 (14.95) |
| Energetic | 40.75 (21.23) | 47.13 (23.69) | 44.52 (23.20) | 46.02 (21.93) | 40.34 (14.83) | 44.03 (23.38) | 39.44 (25.08) | 45.65 (24.75) |
| Irritated | 17.41 (21.76) | 18.55 (20.88) | 16.12 (20.67) | 13.11 (19.35) | 9.34 (14.26) | 13.01 (20.67) | 9.23 (14.94) | 8.87 (13.40) |
| Down | 19.76 (20.69) | 18.78 (19.68) | 11.45 (12.13) | 12.42 (16.39) | 10.37 (15.36) | 9.50 (12.57) | 7.70 (12.42) | 9.19 (14.69) |
| Suspicious | 10.81 (12.28) | 11.80 (11.95) | 9.46 (11.13) | 10.13 (11.43) | 7.66 (10.55) | 7.74 (10.17) | 5.23 (7.95) | 6.28 (7.86) |
| Hopeful | 39.63 (21.90) | 48.03 (23.32) | 45.85 (24.21) | 49.09 (21.96) | 43.34 (21.00) | 45.59 (24.31) | 38.14 (25.35) | 48.57 (25.93) |
| Helpless | 12.91 (14.89) | 12.92 (14.05) | 8.57 (11.86) | 10.74 (13.34) | 7.31 (11.07) | 9.79 (13.92) | 5.70 (9.94) | 7.10 (10.03) |
| Guilty | 19.21 (18.63) | 15.88 (14.53) | 14.04 (19.20) | 12.28 (14.07) | 9.64 (13.21) | 11.08 (15.36) | 6.43 (9.81) | 8.05 (11.85) |
| Content | 48.58 (19.18) | 51.17 (19.88) | 52.19 (22.24) | 53.24 (21.22) | 51.69 (16.63) | 54.28 (20.48) | 51.37 (21.48) | 58.93 (19.97) |
| Insecure | 18.59 (19.56) | 19.52 (18.99) | 14.80 (17.39) | 17.81 (18.54) | 10.13 (11.47) | 11.07 (16.68) | 6.05 (8.65) | 8.41 (11.69) |

SD = standard deviation; NA = (aggregated) negative affect; PA = (aggregated) positive affect; PCT = protective cognitive therapy; ADM = antidepressant medication
