# Supplementary material for: Differential impact of preventive cognitive therapy while tapering antidepressants versus maintenance antidepressant treatment on affect fluctuations and individual affect networks and impact on relapse: a secondary analysis of a randomised controlled trial
Source: eClinicalMedicine. 2023 Nov 22;66:102329. doi: 10.1016/j.eclinm.2023.102329 (PMC10700372; doi:10.1016/j.eclinm.2023.102329)
Supplement: Study protocol [file mmc3.pdf]

ISRCTN15472145 https://doi.org/10.1186/ISRCTN15472145

Imagine your mood: a step towards personalized relapse prevention in depression

Submission date	Recruitment status		
14/11/2016	No longer recruiting	✓	Retrospectively registered
		✓	Protocol added
**Registration date**	**Overall study status**	?	SAP not yet added
17/11/2016	Completed	✓	Results added
**Last Edited**	**Condition category**	?	Raw data not yet added
13/12/2018	Mental and Behavioural Disorders	✓	Study completed

Plain English Summary
Background and study aims
Major Depressive Disorder (depression) is a mood disorder that causes a persistent feeling of sadness and loss of interest. One of the options to reduce the burden of Major Depressive Disorder is to prevent it from coming back (relapse after remission). People diagnosed with recurrent Major Depressive Disorder have a very high risk of relapse after remission. Relapse prevention strategies for previously depressed people include continuing to take antidepressant medication and preventive psychological treatment. However, it is unclear what relapse prevention strategy works best for whom. The effect may be improved when interventions are tailored to specific people. Such personalization of treatment may be based on repeated assessments of a patient. More information is needed about differences in the risk of relapse, response to treatment, and how different relapse prevention strategies may target vulnerability for relapse. The aim of this study is to collect this information to inform personalized relapse prevention strategies. Repeated measurements in daily life using a smartphone application will be used to explore individual differences in vulnerability for relapse, and to explore what may work for whom to prevent relapse. The results may give new insights into individual differences in depression and ultimately may pave the way to tailored relapse prevention strategies.

Who can participate?
People aged 18 – 65 who have previously had depression and are taking antidepressant medication, and healthy volunteers who have never had depression

What does the study involve?
Previously depressed participants are randomly allocated to one of the three groups. Participants assigned to the first group continue taking antidepressant medication. Participants assigned to the second group taper (reduce) their antidepressant medication and attend eight sessions of preventive cognitive therapy. Participants assigned to the third group receive preventive cognitive therapy and continue taking antidepressant medication. All previously depressed participants are monitored over a 24-month follow-up period. The smartphone application “Imagine your mood” has been developed specifically for this study to assess the participants’ emotions and behavior in daily life. A trained research assistant installs the “Imagine your mood” application on the smartphone of the participants. Participants are

instructed to fill out questionnaires in response to triggers that are set at 10 random moments throughout the day, for 3 days a week during the first 8 weeks of the study (parallel to the 8-week preventive cognitive therapy period). The same data is also collected from the healthy volunteers during an 8-week period.

What are possible benefits and risks of participating?
All previously depressed participants will be monitored for depressive relapse and informed in case of a depressive relapse, so that they can contact their GP or psychiatrist. Participants who receive preventive cognitive therapy may potentially benefit from it in terms of reduced risk of relapse. Participants who are allocated to tapering of antidepressant medication may potentially benefit from a reduction of side-effects of the antidepressant medication. How tapering of antidepressant medication influences the risk of relapse is unclear. For all participants, taking part in the study will cost time and effort.

Where is the study run from?
University of Groningen (Netherlands)

When is the study starting and how long is it expected to run for?
September 2012 to June 2017

Who is the main contact?
Prof. Claudi Bockting
c.l.h.bockting@uu.nl

Contact information

Type(s)
Scientific

Contact name
Prof Claudi Bockting

ORCID ID
http://orcid.org/0000-0002-9220-9244

Contact details
Department of Clinical & Health Psychology
Utrecht University
PO Box 80140
Utrecht
Netherlands
3508 TC
+31 (0)30 2531470
c.l.h.bockting@uu.nl

Additional identifiers

EudraCT/CTIS number

IRAS number

ClinicalTrials.gov number

Protocol/serial number
NL 24000.097.08

Study information

Using app-based Experience Sampling Methodology to explore processes of change in a randomized controlled (micro) trial on relapse prevention interventions for recurrent depression

Study hypothesis
The aim of this study is to gain understanding of individual (affective) differences and processes of change that may inform personalized relapse prevention. To this end, Experience Sampling Methodology (ESM) will be used to explore affect in the daily life of previously depressed individuals randomized to different relapse prevention strategies. Additionally, a group of matched never depressed controls will be included.

Research questions:
1. Can individual differences in affect and within-individual affective change be discerned and do these signal future relapse?
2. How are (cognitive) mechanisms related to (affective) vulnerability?
3. Do early affective responses predict who will be able to taper antidepressant medication (ADM)?
4. Do relapse prevention strategies may differentially alter vulnerability for relapse?

Ethics approval required
Old ethics approval format

Ethics approval(s)
This micro-trial was conducted in a subset of patients that were included in the RCT “Disrupting the rhythm of depression” (Netherlands Trial Register: NTR1907). The addition of ESM to this RCT “Disrupting the rhythm of depression” was approved by the medical ethical board of University Medical Center Groningen, Groningen, 22/11/2013, ref: METc 2009/158. In addition, a group of matched healthy volunteers was included. Ethical approval for including these never-depressed individuals was obtained from the University of Groningen Ethical Committee of the Psychology Department, 22/10/2014, ref: ppo-014-043.

Study design
Single-blind randomised controlled trial

Primary study design
Interventional

Secondary study design
Randomised controlled trial

Study setting(s)
Other

Study type(s)

Treatment

Participant information sheet
Not available in web format, please use contact details to request a participant information sheet

Condition
Recurrent major depressive disorder

Interventions
Previously depressed participants will be randomly assigned to:
1. Preventive cognitive therapy (PCT) while continuing ADM (PCT + ADM)
2. PCT while tapering ADM (PCT – ADM)
3. Continuation ADM (ADM) control group

Participants in the PCT + ADM and PCT – ADM groups will receive eight weekly sessions of individual PCT. In the PCT – ADM group, participants will be instructed to gradually taper ADM in four weeks guided by their GP or psychiatrist, who will also be informed and advised. In the continuation ADM group and in the ADM + PCT group, participants and their GPs or psychiatrists will be advised to continue ADM.

Intervention Type
Mixed

Primary outcome measure
Given that this is an exploratory study on processes of change, there are no primary outcome measures defined.
To examine the research questions, the following measures will be used:
1. Affect, mood and mental imagery, collected using ESM. The experience sampling app "Imagine your mood" has been developed and programmed using TEMPEST software. The application has been programmed to set triggers for questionnaires 10 times a day, 3 days a week (on Thursdays, Fridays and Saturdays), for 8 weeks, resulting in a maximum of 240 completed responses. The triggers are set semi-randomly, between the hours of 7.30 and 22.30, with a maximum of one trigger per 90-minute interval and a minimum of 30 minutes between triggers. The ESM procedure will take place during the first 8 weeks of the study (parallel to the 8-week therapy period for previously depressed individuals who have been randomly assigned to receiving preventive cognitive therapy)
2. Relapse/recurrence over a follow-up period of minimal 15 months, using DSM-IV-TR criteria as assessed by the Structural Interview for DSM-IV (SCID, telephonic version) at 3 months, 9 months, at 15 months and at 24 months (current depressive symptomatology and previous 3, 6 or 9 months)
3. The ADM dose, assessed weekly during the ESM study period by telephone interviews

Secondary outcome measures
The following measures will be included for hypothesis-generating purposes:
1. Qualitative information on the use of the app, assessed in weekly phone calls during the 8-week ESM study period
2. Additional questions in the app questionnaire, assessed during the 8-week ESM study period (10 times a day, 3 days a week) using the experience sampling app "Imagine your mood":
2.1. Questions on the impact of ESM
2.2. Further assessment of participants mental representation

2.3. Physical complaints
2.4. Current and previous activities and future events
3. Additional measures assessed in the trial ‘Disrupting the rhythm of depression’ as described in the study design and protocol (Netherlands Trial Register: NTR1907).

Overall study start date
01/09/2012

Overall study end date
01/06/2017

Eligibility

Participant inclusion criteria
Previously depressed participants:
1. A diagnosis of remitted MDD, with at least two previous depressive episodes in the past five years.
2. A current score of <10 on the Hamilton Rating Scale for Depression (HRSD)
3. Use of continuation ADM in the last six months before the start of the study
4. Age between 18 and 65

Healthy volunteers:
1. Age between 18 and 65
2. Match the previously depressed sample on age and gender

Participant type(s)
Mixed

Age group
Adult

Lower age limit
18 Years

Sex
Both

Target number of participants
Total previously depressed: N = 45, equally distributed over the three groups (15 per group).
Total matched healthy volunteers: N = 15

Participant exclusion criteria
Previously depressed participants:
1. Current mania or hypomania or a history of bipolar illness
2. Any psychotic disorder (current and previous)
3. Organic brain damage
4. Alcohol or drug misuse
5. Predominant anxiety disorder
6. Current psychotherapy (> twice a month).

Healthy volunteers:
1. Current or previous depressive episodes
2. Use of ADM.

Recruitment start date
01/01/2014

Recruitment end date
31/01/2015

Locations

Countries of recruitment
Netherlands

Study participating centre
University of Groningen - Clinical Psychology Department
Grote Kruisstraat 2/1
Groningen
Netherlands
9712TS

Study participating centre
GGZ Drenthe
Stationsstraat 20
Emmen
Netherlands
7801 CG

Study participating centre
University Center of Psychiatry Groningen
Hanzeplein 1
Groningen
Netherlands
9700 RB

Sponsor information

Organisation
NWO - Netherlands Organisation for Scientific Research, Dutch National Scientific Foundation, Dutch National Science Foundation

Sponsor details
PO Box 93138
The Hague
Netherlands
2509 AC
+31 (0)70 344 06 40
nwo@nwo.nl

Sponsor type
Research organisation

Website
http://www.nwo.nl/en

Organisation
ZonMW - The Netherlands Organisation for Health Research and Development

Sponsor details
Postbox 93 245
The Hague
Netherlands
2509 AE
+31 (0)70 349 51 11
nfo@zonmw.nl

Sponsor type
Research organisation

Website
http://www.zonmw.nl/en/

Organisation
Netherlands Organisation for Scientific Research

Sponsor details

Sponsor type
Government

Website
http://www.nwo.nl/

ROR
https://ror.org/04jsz6e67

Funder(s)

Funder type
Research organisation

Funder Name
Nederlandse Organisatie voor Wetenschappelijk Onderzoek

Alternative Name(s)
Dutch Research Council, Dutch National Scientific Foundation, Dutch National Science Foundation, Netherlands Organisation for Scientific Research, Dutch Research Council, Netherlands, NWO

Funding Body Type
Government organisation

Funding Body Subtype
National government

Location
Netherlands

Funder Name
ZonMw

Alternative Name(s)
Netherlands Organisation for Health Research and Development

Funding Body Type
Private sector organisation

Funding Body Subtype
Other non-profit organizations

Location
Netherlands

Results and Publications

Publication and dissemination plan
To be confirmed at a later date

Intention to publish date
01/06/2018

Individual participant data (IPD) sharing plan

The datasets generated during and/or analysed during the current study are/will be available upon request from Prof. Dr Claudi Bockting (C.L.H.Bockting@uu.nl)

IPD sharing plan summary

Available on request

Study outputs

Output type	Details	Date created	Date added	Peer reviewed?	Patient-facing?
Protocol article	protocol	12/07/2017		Yes	No
Results article	results	01/07/2018		Yes	No
Results article	results	01/08/2018		Yes	No
