# Supplementary material for: Differential impact of preventive cognitive therapy while tapering antidepressants versus maintenance antidepressant treatment on affect fluctuations and individual affect networks and impact on relapse: a secondary analysis of a randomised controlled trial
Source: eClinicalMedicine. 2023 Nov 22;66:102329. doi: 10.1016/j.eclinm.2023.102329 (PMC10700372; doi:10.1016/j.eclinm.2023.102329)
Supplement: Trial Registration DRD study [file mmc5.pdf]

Gepubliceerd: 14-07-2009 Laatst bijgewerkt: 13-12-2022

Research questions: 1. What are the effects of the addition of preventive CT to AD compared to AD alone in remitted patients with recurrent MDD with a follow-up of 15 months-24 months on: A. Time related proportion of depression recurrence; B. The...

**Ethische beoordeling**	Goedgekeurd
**Status**	Werving nog niet gestart
**Type aandoening**	-
**Onderzoekstype**	Interventie onderzoek

Samenvatting

Verkorte titel

N/A

Health condition

Major depressive disorder, relapse, recurrence, cognitive therapy, prevention, cost-effectiveness

Depressie, terugval, cognitive therapie, preventie, kosteneffectiviteit

Ondersteuning

Primaire sponsor :	N/A
Overige ondersteuning :	ZON MW, OOG, Doelmatigheid, K&E

Onderzoeksproduct en/of interventie

Uitkomstmaten

Primaire uitkomstmaten

Time related proportion of depression relapse/recurrence during 15-24 months using DSM-IV-R criteria as assessed by the Structural Clinical Interview for Depression.

Secundaire uitkomstmaten

1. Cost-effectiveness;
2. Number, duration and severity of relapses/recurrences;
3. Potential mediators of the effect.

Toelichting onderzoek

Achtergrond van het onderzoek

Objective: Maintenance treatment with antidepressants is the leading strategy to prevent relapse and recurrence in patients with recurrent major depressive disorder (MDD) who have responded to acute treatment with AD (Dutch Multidisciplinary Guideline for Depression, 2005).

However, previous studies, including ours, indicated that most patients (up to 70-80%) in clinical practice are not willing to take this medication after remission or take too low dosages. Moreover, as patients need to take medication for several years (if not for the rest of their lives), it may not be the most cost-effective strategy.

The best established effective and available alternative is brief cognitive therapy (CT). However, it is unclear whether the combination of AD to brief CT is beneficial. In addition, it is unclear whether brief CT while tapering antidepressants (AD) is an effective alternative for long term use of AD in recurrent depression. Therefore, we will compare the effectiveness and cost-effectiveness of brief CT added to maintenance AD versus maintenance AD alone versus guided tapering of AD with brief CT.
In addition, we examine whether the prophylactic effect of CT was due to CT tackling illness related risk factors for recurrence such as residual symptoms or to its efficacy to modify presumed (psychological) vulnerability factors of recurrence, e.g. rigid dysfunctional attitudes, using self report and indirect measures (i.e., implicit measures).

Study design: A multicenter RCT comparing brief CT with guided tapering of AD versus continuation of maintenance treatment with AD versus brief CT combined with continuation of AD.

Study population: Remitted patients on AD with at least two previous depressive episodes in the past five years.

Interventions: Brief CT (while tapering AD) versus continuation of AD use versus brief CT combined continuation of AD.

Outcome measures: Primary outcome; time related proportion of depression

relapse/recurrence during 15-24 months using DSM-IV-R criteria as assessed by the Structural Clinical Interview for Depression.

Secondary outcome: Number, duration and severity of relapses/recurrences.

Doel van het onderzoek

Research questions:
1. What are the effects of the addition of preventive CT to AD compared to AD alone in remitted patients with recurrent MDD with a follow-up of 15 months-24 months on:
A. Time related proportion of depression recurrence;
B. The number, duration and severity of recurrences;
C. Costs of subsequent medical consumption associated with relapses and work absenteeism as assessed by TIC-P. Other aspects like the impact of depression (including severity of depression) on health related quality of life will be assessed by various self-report measures, including preference based outcomes (EQ-5D) to be applied in the economic analysis.
2. What are the effects of either of preventive CT with tapering of AD compared to continuation on AD with a follow-up of 15 months-24 months on:
A. Time related proportion of depression recurrence;
B. The number, duration and severity of recurrences;
C. Costs of subsequent medical consumption associated with relapses and work absenteeism as assessed by TIC-P. Ultimately, the balance between costs and effects of CT alone will be compared against AD alone.

3. Identifying differential illness-, stress-, and cognitive related predictors of response to CT versus AD to examine what works for whom and thereby accomplish better future treatment-patient matching.

4. Does preventive CT reduce:
A. Implicit and/or explicit dysfunctional attitudes;
B. Difficulty to disengage from negative material;
C. Residual symptoms;
D. Daily hassles;
E. Non-adherence to AD.
5. Is the reduction of these factors (4A t/m 4E) related to the reduction of relapse/recurrence?

Onderzoeksopzet

Primary outcome: Relapse/recurrence over a follow-up period of minimal 15 months using DSM-IV-TR criteria as assessed by the Structural Interview for DSM-IV (SCID, telephonic version) at three months, 9 months and at least 15 months-24 months (current depressive symptomatology and previous 3 and 6 months).

Onderzoeksproduct en/of interventie

Brief CT (while tapering AD) versus continuation of AD use versus brief CT combined continuation of AD.

Contactpersonen

Publiek

Rijksuniversiteit Groningen
Klinische Psychologie
Grote Kruisstraat 2/1
C.L.H. Bockting
Rijksuniversiteit Groningen
Klinische Psychologie
Grote Kruisstraat 2/1
Groningen 9712 TS
The Netherlands
+31 (0)50-3636479

Rijksuniversiteit Groningen
Klinische Psychologie
Grote Kruisstraat 2/1
C.L.H. Bockting
Rijksuniversiteit Groningen
Klinische Psychologie
Grote Kruisstraat 2/1
Groningen 9712 TS
The Netherlands
+31 (0)50-3636479

Deelname eisen

Belangrijkste voorwaarden om deel te mogen nemen (Inclusiecriteria)

Remitted patients on AD with at least two previous depressive episodes in the past five years.

Belangrijkste redenen om niet deel te kunnen nemen (Exclusiecriteria)

1. Current mania or hypomania or a history of bipolar illness;
2. Any psychotic disorder (current and previous);
3. Organic brain damage;
4. Alcohol or drug misuse;
5. Predominant anxiety disorder.

Onderzoeksopzet

Opzet

Type :	Interventie onderzoek
Onderzoeksmodel :	Parallel
Toewijzing :	Gerandomiseerd
Blindering :	Enkelblind
Controle :	Geneesmiddel

Deelname

Nederland

Status :	Werving nog niet gestart
(Verwachte) startdatum :	01-08-2009
Aantal proefpersonen :	276
Type :	Gerealiseerd

Ethische beoordeling

Goedgekeurd

Datum :	14-07-2009
Soort :	Eerste indiening

Registraties

In dit register bekende (historische) registraties

In overige registers

Register	ID
NTR-new	NL1797
NTR-old	NTR1907
Ander register	MEtIGG : 8220
ISRCTN	ISRCTN wordt niet meer aangevraagd.

Resultaten

Samenvatting resultaten

N/A
